# One-step multicomponent synthesis of chiral oxazolinyl-zinc complexes

**DOI:** 10.1186/s13065-017-0305-1

**Published:** 2017-08-09

**Authors:** Mei Luo, Jing Cheng Zhang, Wen Min Pang, King Kuok Hii

**Affiliations:** 1 College of Chemistry and Chemical Engineering, Hefei University of Technology, Hefei, 230009 People’s Republic of China; 2 Department of Chemistry, University of Science and Technology of China, Hefei, 230009 People’s Republic of China; 3 Department of Chemistry, Imperial College London, Exhibition Road, South Kensington, London, SW7 2AZ UK

**Keywords:** Chiral organozinc complexes, A single step, Nitriles, Chiral D/L amino alcohols,

## Abstract

**Background:**

Typically, oxazolinyl metal complexes are synthesized in two steps, where the free ligand is prepared by the condensation reaction between a functionalized nitrile and an amino alcohol in the presence of a Lewis or Brønsted acid catalyst, followed by a further reaction with metal salts to obtain the corresponding metal complexes. Very often, the yield afforded by the two-step procedure is not high, and very few oxazolinyl zinc complexes have been prepared by this route. Given that metal-oxazoline complexes often contain Lewis acidic metals, it is conceivable that the two steps may be telescoped.

**Results:**

A series of novel chiral organozinc complexes **1**–**15** were assembled in a single step, All crystalline compounds were fully characterized, including the report of 15 X-ray crystal structures, including a wide structural diversity.

**Conclusions:**

A series of novel chiral organozinc complexes were assembled in a single step, from nitriles, chiral D/L amino alcohols, and a stoichiometric amount of ZnCl_2_, with moderate to high yields (20–90%).

**Electronic supplementary material:**

The online version of this article (doi:10.1186/s13065-017-0305-1) contains supplementary material, which is available to authorized users.

**Graphical abstract:**
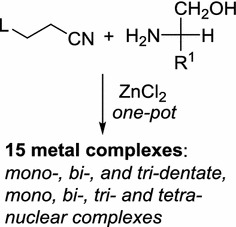
A series of novel chiral organozinc complexes can be assembled in moderate to high yields (20–90%), from a mixture of nitriles, chiral amino alcohols and ZnCl2. All crystalline compounds were fully characterized, including the report of 15 X-ray crystal structures, including a wide structural diversity.

## Background

Chiral oxazolines constitute an important class of ‘privileged’ ligands in asymmetric catalysis [1–3]. Chiral zinc complexes containing these ligands exhibit a broad range of catalytic activities, including the asymmetric Mukaiyama-aldol reactions of α-ketoesters [4], the Henry reaction [5], isoselective ring-opening polymerization of rac-lactide [6], and asymmetric co-polymerisation of cyclohexene oxide with CO_2_ [7]. More recently, a chiral boxmi-Zn catalyst has been reported to be highly effective for the enantioselective alkylation of oxindoles and α-ketoesters, thought to proceed through an usual radical pathway [8].

**Scheme 1:**
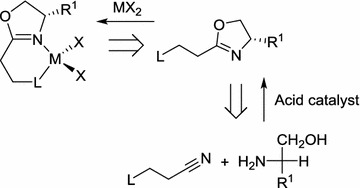
Two-step synthesis of oxazolinyl-metal complexes

Typically, oxazolinyl metal complexes are synthesized in two steps, where the free ligand is prepared by the condensation reaction between a functionalized nitrile and an amino alcohol in the presence of a Lewis or Brønsted acid catalyst, followed by a further reaction with metal salts to obtain the corresponding metal complexes (Scheme 1) [9, 10]. Very often, the yield afforded by the two-step procedure is not high, and very few oxazolinyl zinc complexes have been prepared by this route. Given that metal-oxazoline complexes often contain Lewis acidic metals, it is conceivable that the two steps may be telescoped. Herein, we will report a simple, one-pot procedure for the preparation of oxazolinyl-zinc complexes by the atom-efficient assembly of three reactive components: a nitrile, an amino alcohol and a zinc salt. In all cases, the complexes were isolated, purified and characterized; their structures were further confirmed by X-ray crystallography.

## Results and discussion

**Scheme 2:**
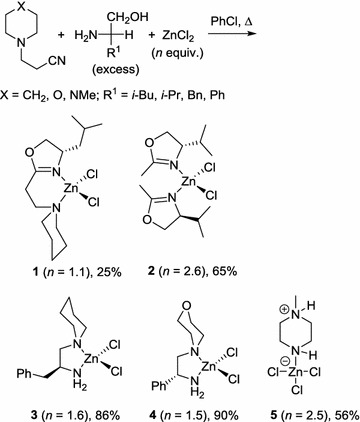
Effect of reaction stoichiometry (metal precursor) ZnCl_2_

The one-pot procedure was initially tested by refluxing a mixture of 1-piperidinepropionitrile with 2–3 eq of amino alcohol in the presence of ZnCl_2_ (1–2.5 eq) in chlorobenzene. Following the reaction, excess ZnCl_2_ can be removed by an aqueous wash, and the metal complexes were isolated and purified by column chromatography. During the preliminary work, it became quickly apparent that the reaction outcome is highly dependent upon the amount of ZnCl_2_ used (Scheme 2): Using 1.1 eq of the metal salt, the desired amino-oxazolidinyl complex 1 can be obtained from l-leucinol, but only in a low yield (25%). While the use of an excess (2.5 eq) of the zinc salt with l-valinol led to the formation of the bis-oxazolidinyl zinc complex **2**, containing two monodentate ligands.

The nature of the side chain (R^1^) also influenced the reaction outcome: using l-phenylalaninol with ZnCl_2_ (1.6 eq) led to the cleavage of the propionitrile to give the unsymmetrical diamine complex **3** in a very good yield (86%). Similarly, addition of the 1.5 eq of ZnCl_2_ to 1-morpholinepropionitrile (X=O) and d-phenylglycinol furnished complex **4** in 90% yield. Interestingly, using 1-(2-cyanoethyl)-4-methylpiperazine (Z=NMe) as a precursor with 2.5 eq of the ZnCl_2_ led only to the formation of the zwitterionic piperazine-complex **5**, irrespective of the amino alcohol used.

**Scheme 3:**
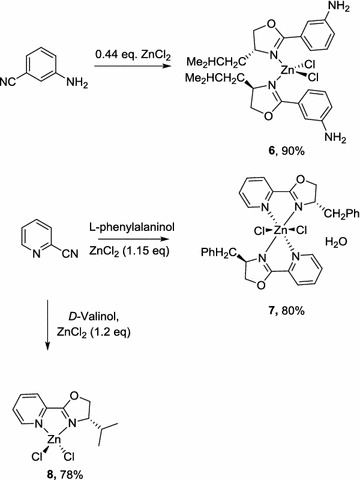
Zinc complexes derived from 3-aminobenzonitrile and 2-cyanopyridine

The formation of complexes **2**–**5** indicates that the propionitrile precursors are unstable under the reaction conditions in the presence of excess ZnCl_2_, which can decompose into acetonitrile (affording **2**) or the parent cyclic amines (**3**–**5**). With this in mind, a number of nitrile precursors were chosen which are more robust against degradation under the reaction conditions. Consequently, a number of aromatic nitrile precursors containing additional N-donors were examined as precursors in these 3-component reactions. In these reactions, the amount of ZnCl_2_ was carefully optimizedto ensure a specific outcome. The use of 3-aminobenzonitrile and D-leucinol in the presence of 0.44 eq of ZnCl_2_ led to the formation of complex **6** containing two monodentate ligands coordinating via the oxazoline nitrogen (Scheme 3). The use of 2-cyanopyridine with 1.2 eq of ZnCl_2_, on the other hand, led to different outcomes with different amino alcohols: the formation of a bis-chelated complex **7** was obtained with l-phenylalaninol, while the mono-chelated complex **8** was obtained from d-valinol. This result highlights the importance of the sidechain present in the amino alcohol precursor; presumably, the sterically bulky isopropyl group prevented the formation of the bis-chelate complex.

**Scheme 4:**
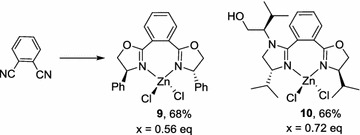
Complexes derives from isophathalonitrile

It was anticipated that oxazolines derived from 1, 2-dicyanobezene will provide C2-symmetricalbis-oxazolines that form 7-membered chelate rings, which can only form a 1:1 adduct with zinc dichloride. Indeed, the condensation of isophthalonitrile with d-phenylglycinol (0.56 eq) afforded the predicted mono-chelated complex **9** [11] in a good yield (Scheme 4). However, the presence of a slight excess of l-valinol (0.72 eq) caused the condensation of three amino alcohols in complex **10**.

**Scheme 5:**
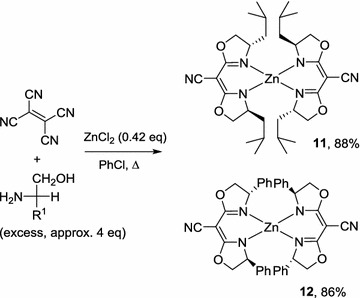
Neutral zinc complexes derived from tetracyanoethylene

Condensation of l-leucinol and phenyl glycinol with tetracyanoethylene in the presence of 0.42 eq of ZnCl_2_ provided neutral bis[bis(oxazoline)]zinc (II) complexes **11** and **12**, respectively, in good yields (Scheme 5). The formation of these methylene-bis(oxazoline) structures indicates disproportionation-rearrangement of the tetracyanoethylene precursor (to tricyanomethane), although the precise mechanism of this is unclear. During the preparation of this manuscript, the synthesis complex **12** (by a different route) was reported by Kögel et al. [12] Interestingly, compound **12** was reported to display intense Cotton effect as a result of exciton coupling. Indeed, a comparison of their X-ray crystal structures revealed that the isobutyl-substituted complex **11** possesses a fairly symmetrical tetrahedral coordination environment; while, in contrast, complex **12** is highly distorted (See Figs. 11 and 12 in Additional file 1). We speculate this may be due to the favourable intramolecular π-interaction between one of the phenyl substituent with the semicorrin structure of the adjacent ligand within 3.5 Å, effectively bringing the two chiral chromophores into close proximity to facilitate exciton coupling [13].

**Scheme 6:**
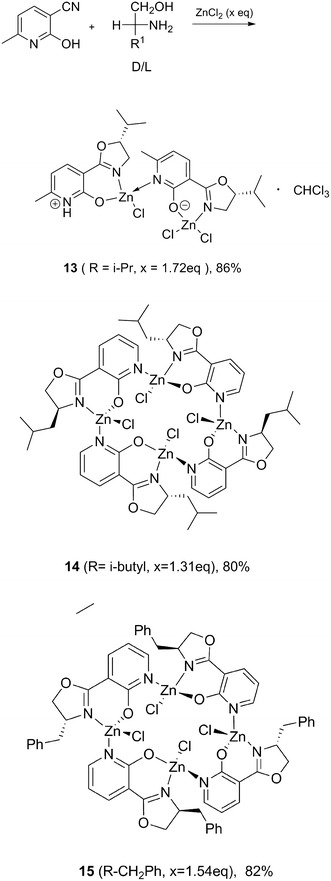
Multinuclear zinc complexes

In the final part of this study, 2-hydroxy-6-methylnicotinonitrile was employed as a precursor, to test the utility of the one-pot methodology in assembling complex multinuclear structures. Condensation product with valinol furnished the binuclear zwitterionic complex **13** (Scheme 6). Presumably, the formation of higher aggregates is prevented by the sterically demanding isopropyl substituent.

Highly symmetrical tetramers **14** and **15** (Scheme 6) were formed when leucinol or phenylalaninol were used as precursors in the presence of 1.5 eq of ZnCl_2_ (See Figs. 14 and 15 in Additional file 1). A six-membered N, O-chelate is formed preferentially at each metal centre, and the pendant pyridine acting as a bridging donor ligand to another metal centre. With each zinc occupying a corner of a square grid, the planar N,O,N-ligands are oriented perpendicularly to one another with diagonal Zn···Zn distance of ca. 6 Å.

The X-ray crystal structures of all the complexes are determined and reported in the supporting information. In all cases, a distorted tetrahedral geometry is found at the zinc(II), and the C=N double bond character of the oxazolindinyl ligand is largely retained in the metal complexes.

## General remarks

Unless otherwise stated, all chemical reagents were purchased from Acros, Aldrich, or Fluka USA. Flash column chromatography was performed using Merck silica gel (60, particle size 0.02–0.03 mm). ^1^H and ^13^C NMR spectra were recoZrded using Bruker AM-500 or AM-600 spectrometers. Chemical shifts are reported in ppm (δ) with the solvent relative to tetramethylsilane (TMS) employed as the internal standard (residual CHCl_3_, δ_H_ 7.26 ppm; CDCl_3_, δ_c_ 77 ppm). The following abbreviations were used to δ designate multiplicities: s = singlet, d = doublet, t = triplet, m = multiplet. Infrared spectra were recorded on a Mattson Galaxy Series FTIR 3000 spectrometer; peaks are reported in cm^−1^. Elemental analyses were obtained on Elemental Analyzer AE-3000. High-resolution mass spectra (HRMS) were obtained on a Micro GCT-MS equipped with an EI ion source. Optical rotations were measured on a WZZ-1 automatic polarimeter with a 2-cm cell, recorded at the sodium d-line.

### The procedure for the synthesis of the complexes 1–15

#### 1-[2-(4-isobutyl-4,5-dihydro-oxazol-2-yl)-ethyl]-piperidine zinc(II) dichloride, 1

A dry 100 mL Schlenk flask was purged with N_2_ and charged with anhydrous ZnCl_2_ (2.515 g, 18.45 mmol), 3-piperidin 1-yl propionitrile (2.462 g, 17.81 mmol) and l-leucinol (4.824 g, 41.16 mmol). 40 mL of chlorobenzene was added, and the reaction mixture was refluxed for 72 h. After cooling to room temperature, the solvent was removed under reduced pressure, and the residue was dissolved in 15 mL of H_2_O and extracted with CH_2_Cl_2_ (3 × 20 mL). The combined organic extracts were evaporated to give a crude red oil, which was purified by column chromatography (petroleum ether/CH_2_Cl_2_, 4/1) to afford the title compound as colourless crystals in 25% yield, m.p. 50–52 °C $\left[ {\alpha } \right]_{\text{D}}^{25}$ = +67.5° (c = 0.02, MeOH); δ_H_ (600 MHz, CDCl_3_, 27 °C) 4.52–4.56 (m, 1H), 4.12–4.16 (m, 1H), 4.01–4.03 (m, 1H), 2.89–2.92 (m, 1H), 2.73–2.75 (m, 1H), 2.59–2.64 (m, 3H), 2.31 (t, *J* = 12.4 Hz, 1H), 1.62–1.78 (m, 6H), 1.43–1.44 (m, 2H), 1.29–1.35 (m, 2H), 1.15–1.20 (m, 1H), 0.84–0.90 (m, 6H); δ_C_ (150 MHz, DMSO-d_6_), 170.0, 73.7 (×2), 65.5, 62.9 (×2), 54.7, 46.1, 44.4, 43.3, 25.1, 24.0, 23.4, 22.6. ν_max_(cm^−1^) 3274, 2954, 2869, 1648, 1587, 1468, 1387, 1368, 1319, 1283, 1169, 1076 1041, 979, 956, 949, 904, 864, 839, 780, 607, 493. Found C: 45.36, H: 7.19, N: 7.75%; C_14_H_26_Cl_2_N_2_OZn requires C: 44.88, H: 7.00, N: 7.48%.

#### Bis-[4-isopropyl-2-methyl-4,5-dihydro-oxazole]zinc(II) dichloride, 2

Prepared using the same procedure described above for complex **1**, from a mixture of ZnCl_2_ (5.401 g, 39.63 mmol), 3-piperidin1-yl propionitrile (2.321 g, 16.79 mmol) and l-valinol (5.319 g, 51.56 mmol) in chlorobenzene (80 mL). The product was obtained as colourless crystals in 65% yield after column chromatography (petroleum ether/CH_2_Cl_2_, 2/1), m.p. 60–62 °C, $\left[ {\alpha } \right]_{\text{D}}^{25}$ = +9.97° (c = 0.35, MeOH); δ_H_ (600 MHz, CDCl_3_, 27 °C) 3.64–3.71 (m, 6H), 2.03 (s, 6H), 1.85–1.88 (m, 2H), 0.92 (d, *J* = 6.8 Hz, 6H), 0.96 (d, *J* = 7.8 Hz, 6 H); δ_C_ (150 MHz, CDCl_3_), 171.2(×2), 63.7, 63.6, 57.1, 53.5, 31.7, 28.9, 23.4, 22.9, 19.3, 19.0, 18.8, 17.9; ν_max_(cm^−1^) 3436, 3284, 3145, 2954, 2864, 1725, 1659, 1585, 1458, 1420, 1393, 1319, 1278, 1280, 1219, 1092, 1038, 974, 952, 905, 765. Found C: 43.49, H: 7.19, N: 7.23%; C_14_H_26_Cl_2_N_2_O_2_Zn requires C: 43.04, H: 6.71, N: 7.17%.

#### α-Phenyl-1-hexahydropyridyl ethylamine zinc(II), 3

Prepared using the same procedure described above, using anhydrous ZnCl_2_ (3.5002 g, 25.68 mmol), 3-piperidin1-yl propionitrile (2.4590 g, 17.79 mmol), and l-phenylalaninol (5.5420 g, 40.40 mmol) in 80 mL of dry chlorobenzene. The product was obtained as colorless crystals after column chromatography (petroleum ether/dichloromethane, 1/2) in 86% yield, m.p: 168–172 °C; $\left[ {{\text{a}}} \right]_{\text{D}^{25}}$ = −29.30º (c = 0.016, CH_3_OH): δ_H_ (600 MHz, CDCl_3_, 27 °C) 7.32–7.43 (m, 5H), 4.23–4.32 (m, 1H), 3.42–3.64 (m, 3H), 2.96–3.05 (m, 2H), 2.61–2.65 (m, 1H), 2.19–2.41 (t, J = 912.4 Hz, 1H), 1.62–1.78 (m, 6H), 1.43–2.41 (m, 3H), 1.69–1.98 (m, 6H), 1.24–1.31 (m, 1H), δ_C_ (150 MHz, DMSO-d_6_), 169.2, 141.8, 128.9, 128.4, 127.4, 127.3, 127.1 65.2, 65.1, 55.4, 55.2, 53.8, 51.9, 25.3, 23.2(×2); ν_max_: 3447, 3027,2943, 2860, 1648, 1603, 1496, 1455, 1132, 1043, 1060,1040, 1030, 762, 705. Elemental analysis: Found C: C:47.20%, H, 6.05%, N, 7.60%; C_14_H_22_Cl_2_N_2_Zn requires C: 47.42, H: 6.25, N: 7.90%.

#### 2-Morpholin-4-(R)-yl-1-phenyl-ethylamine zinc(II) dichloride complex, 4

Prepared as described above, from a mixture of anhydrous ZnCl_2_ (1.780 g, 13.06 mmol), *N*-cyanoacetylmorpholine (1.501 g, 9.74 mmol), d-phenylglycinol (4.097 g, 29.87 mmol) and dry chlorobenzene (40 mL). The reaction mixture was refluxed for 60 h. The product was purified by column chromatography (petroleum ether/CH_2_Cl_2_, 1/100) to afford the title compound as colourless crystals in 90% yield, m.p. 196–198 °C, $\left[ {\alpha } \right]_{\text{D}}^{25}$ = −42.43° (c = 0.13, THF); δ_H_ (500 MHz, DMSO-d_6_, 27 °C), 7.48 (d, *J* = 8.8 Hz, 2H), 7.36 (t, *J* = 7.5 Hz, 2H), 7.30 (t, *J* = 7.3 Hz, 1H), 4.95 (br s, 2H), 4.14 (t, *J* = 11.9 Hz, 1H), 3.88-3.91 (m, 2H), 3.78–3.81 (m, 2H), 2.97–3.00 (m, 2H), 2.67–2.82 (m, 4H); δ_C_ (125 MHz, DMSO-d_6_) 139.9, 128.5 (×2), 127.9, 127.0 (×2), 65.6, 65.2 (×2), 54.6 (×2), 51.2; ν_max_(cm^−1^) 3435, 3271, 3228, 3145, 2974, 2928, 2904, 2865, 1591, 1499, 1458, 1447, 1290, 1263, 1146, 1126, 1094, 1072, 1060, 1037, 988, 899, 874, 749, 694. Found C: 42.39, H: 5.24, N: 8.05%; C_12_H_18_N_2_Cl_2_OZn requires C: 42.07, H: 5.30, N: 8.18%.

#### (1-Methyl-piperazine)zinc(II) trichloride, 5

Prepared using the same procedure described for complex 5, from a mixture of anhydrous ZnCl_2_ (5.008 g, 36.75 mmol), 1-(2-cyanoethyl)-4-methylpiperazine (2.313 g, 15.09 mmol) and d-phenylglycinol (10.696 g, 77.97 mmol) in 40 mL of dry chlorobenzene. The product was recrystallized from ethanol/CH_2_Cl_2_, to furnish colourless crystals in 56% yield; m.p. 148–152 °C; δ_H_ (600 MHz, CDCl_3_ and DMSO-d_6_, 27 °C) 4.09–4.12 (m, 1H), 3.64–3.67 (m, 1H), 3.56–3.59 (m, 1H), 2.86–2.88 (m, 4H), 2.37–2.40 (m, 3H), 2.16 (s, 3H); δ_C_ (150 MHz, CDCl_3_ and DMSO-d_6_) 62.1, 54.7, 51.4, 44.0, 42.5. ν_max_(cm^−1^) 3491, 3455, 3189, 3006, 2956, 2771, 1585, 1458, 1387, 1128, 1099, 1058, 1035, 998, 976, 870, 701. Found: C: 22.20, H: 4.56, N: 10.10%; C_5_H_13_Cl_3_N_2_Zn requires C: 22.01, H: 4.80, N: 10.27%.

#### 2-[4R-4,5-dihydro-4-(1′,1′-dimethylethyl)-3-oxazolinyl]aniline zinc(II) dichloride, 6

Prepared using the same procedure described for complex **1**, from a mixture of anhydrous ZnCl_2_ (3.002 g, 22.02 mmol), 3-amino-benzonitrile (6.702 g, 56.73 mmol), and d-leucinol (10.008 g, 85.40 mmol) in 80 mL of dry chlorobenzene. The reaction mixture was refluxed for 72 h. After evaporation, the residue was dissolved in 15 mL of H_2_O and extracted with CH_2_Cl_2_ (3 × 10 mL). The organic layer was evaporated under vacuum, and the red oily residue was purified by column chromatography over silica gel (petroleum ether/CH_2_Cl_2_, 1/4), yield: 90%; m.p. 168–170 °C, $\left[ {\alpha } \right]_{\text{D}}^{25}$ = −54.9° (c = 0.0364, EtOH). δ_H_ (600 MHz, CDCl_3_ and DMSO-d_6_, 27 °C) 7.77–7.90 (m, 1H), 6.98–7.19 (m, 5H), 6.63–6.76 (m, 2H), 5.19–5.33 (m, 1H), 4.26–4.58 (m, 4H), 3.92–3.93 (m, 1H), 3.21–3.25 (m, 4H), 1.70–1.82 (m, 4H), 1.35–1.44 (m, 2H), 0.89–0.96 (m, 12H); δ_C_ (150 MHz, CDCl_3_ and DMSO-d_6_) 167.0, 161.3, 146.9, 146.4, 134.2, 126.9, 115.0, 113.9, 113.0, 112.8, 111.5(×2), 109.9(×2), 70.9, 63.0, 46.5, 45.3, 43.7, 39.5, 23.5, 23.2, 21.5, 21.1, 21.0, 20.3; ν_max_(cm^−1^) 3353, 2957, 2928, 2870, 1625, 1498, 1467, 1386, 1333, 1290, 1171, 1135, 1108, 996, 966, 948, 882, 797, 750, 688, 576, 537. Found C: 54.65, H: 6.24, N: 10.16%; C_26_H_36_N_4_Cl_2_O_2_Zn requires C: 54.51, H: 6.33, N: 9.78%.

#### Bis-[2-(4R-benzyl-4,5-dihydro-oxazol-2-yl)-pyridine]zinc(II) tetrachlorozincate, 7

Prepared using the same procedure described for compound **1**, using anhydrous ZnCl_2_ (3.340 g, 24.51 mmol), 2-cyanopyridine (2.095 g, 20.13 mmol) and l-phenylalaninol (3.992 g, 26.40 mmol) in 40 mL of dry chlorobenzene, and the reaction mixture was refluxed for 60 h. The product was extracted into CH_2_Cl_2_ as described above, and purified by column chromatography (petroleum ether/CH_2_Cl_2_, 1/4) to give colourless crystals in 80% yield; m.p. 134–136 °C, $\left[ {\alpha } \right]_{\text{D}}^{25}$ = +51.4° (c = 0.0272, MeOH); δ_H_ (600 MHz, DMSO-d_6_, 27 °C) 8.78–8.81 (m, 2H), 7.95–8.03 (m, 4H), 7.63–7.68 (m, 2H), 7.22–7.30 (m, 10H), 4.64–4.67 (m, 4H), 4.49–4.51 (m, 2H), 3.30–3.39 (m, 2H), 3.35 (s, 2H), 2.82–2.86 (m, 2H); δ_C_ (150 MHz, DMSO-d_6_) 163.8, 148.5, 137.7, 135.5, 128.2, 127.6, 126.7, 125.7, 122.4, 73.6, 64.5, 39.2; ν_max_(cm^−1^) 3493, 3061, 3027, 2955, 2920, 2853, 1660, 1590, 1571, 1492, 1469, 1452, 1440, 1404, 1388, 1325, 1293, 1244, 1223, 1154, 1143, 1088, 1045, 1014, 947, 847, 801, 746, 703, 681, 632; Found C: 57.43, H: 5.03, N: 8.67%; C_30_H_30_Cl_2_N_4_O_3_Zn_2_ requires C: 57.12, H: 4.79, N: 8.88%.

#### [2-(4S-isopropyl-4,5-dihydro-oxazol-2-yl)-pyridine] zinc (II) dichloride, 8

Prepared using the procedure described above for compound **1**, refluxing a mixture of anhydrous ZnCl_2_ (3.423 g, 25.12 mmol), 2-cyanopyridine (2.128 g, 20.44 mmol), and l-valinol (3.386 g, 32.82 mmol) in 40 mL of dry chlorobenzene for 60 h. The product was purified by column chromatography (petroleum ether/CH_2_Cl_2_, 1/8). Colourless crystals were obtained in 85% yield; m.p. 178–180 °C, $\left[ {\alpha } \right]_{\text{D}}^{25}$ = +23.1° (c = 0.17, MeOH); δ_H_ (600 MHz,CDCl_3_, 27 °C) 8.78–8.80 (m, 1H), 8.20–8.23 (m, 1H), 8.05 (d, *J* = 7.7 Hz, 1H), 7.86–7.88 (m, 1H), 4.96 (t, *J* = 9.5 Hz, 1H), 4.65 (t, *J* = 8.9 Hz, 1H), 4.42–4.45 (m, 1H), 2.10–2.14 (m, 1H), 1.04–1.14 (m, 6H); δ_C_ (150 MHz, CDCl_3_) 166.1, 149.8, 141.2, 140.2, 129.7, 124.1, 75.4, 69.3, 31.6, 18.4, 17.8; ν_max_(cm^−1^) 3223, 3188, 2962, 2875, 1662, 1587, 1470, 1392, 1372, 1320, 1251, 1129, 1046, 878, 836, 791, 752, 690, 539. Found C: 40.81, H: 3.85, N: 8.47%; C_11_H_14_Cl_2_N_2_OZn requires C: 40.46, H: 4.32, N: 8.58%.

#### [1, 2-bis–(4R-phenyl-4,5-dihydro-oxazol-2-yl)phenyl] zinc(II) dichloride, 9

Prepared using the same procedure described above for compound **1**, using anhydrous ZnCl_2_ (2.590 g, 19.01 mmol), isophthalonitrile (3.353 g, 26.17 mmol), and d-phenylglycinol (8.492 g, 61.91 mmol) in 80 mL of dry chlorobenzene, and refluxing for 72 h. The product was purified by column chromatography (petroleum ether/CH_2_Cl_2_, 1/8). Yield = 86%; m.p. >250 °C (dec), $\left[ {\alpha } \right]_{\text{D}}^{25}$ = −54.9° (c = 0.0364, EtOH).δ_H_ (600 MHz, CDCl_3_, 27 °C) 7.77–7.79 (m, 2H), 7.55–7.56 (m, 2H), 7.18–7.28 (m, 10H), 5.28 (t, *J* = 9.2 Hz, 2H), 4.68 (t, *J* = 9.2 Hz, 2H), 4.10 (t, *J* = 8.4 Hz, 2H),δ_C_ (150 MHz, CDCl_3_) 163.5, 140.3, 129.4 (×2), 128.4, 127.0 (×2), 126.0,125.3 (×2), 73.9, 68.3. ν_max_(cm^−1^) 3447, 3058, 2965, 2907, 1650, 1639, 1592, 1495,1473, 1455, 1379, 1363, 1318, 1308, 1278, 1238, 1207, 1153, 1120, 1067, 1020, 991, 945, 760, 704, 648, 594, 556. Found C: 56.92, H: 3.92, N: 5.41%; C_24_H_20_Cl_2_N_2_O_2_Zn requires C: 57.11, H: 3.99, N: 5.55%.

#### 2-{(4*S*-isopropyl-4,5-dihydro-oxazol-2-yl)-phenyl-4,5-dihydro-imidazol-1-yl}-3-methyl-butan-1-ol zinc(II), 10

Prepared using the same procedure described for compound **1**, refluxing a mixture of anhydrous ZnCl_2_ (4.000 g, 29.35 mmol), isophthalonitrile (6.700 g, 52.29 mmol), and l-valinol (16.000 g, 15.51 mmol) in 80 mL of dry chlorobenzene for 72 h. The product was purified by column chromatography (petroleum ether/CH_2_Cl_2_, 1/4). Yield: 90%; m.p. >250 °C (dec), $\left[ {\alpha } \right]_{\text{D}}^{25}$ = +34.4° (c = 0.0436, CHCl_3_),δ_H_ (600 MHz, DMSO-d_6_, 27 °C), 7.78–7.81 (m, 2H), 7.69–7.71 (m, 1H), 7.63–7.66 (m, 1H), 4.90–4.93 (m, 1H), 4.65 (t, *J* = 9.4 Hz, 1H), 4.51–4.55 (m, 1H), 4.45 (t, *J* = 7.8 Hz, 2H), 4.27 (t, *J* = 5.0 Hz, 1H), 3.75 (d, *J* = 11.2 Hz, 1H), 3.62–3.65 (m 2H), 3.47–3.50 (m, 1H), 2.21–2.24 (m, 1H), 1.70–1.74 (m, 1H), 0.94–0.99 (m, 8H), 0.85–0.86 (m, 4H), 0.72 (d, *J* = 6.6 Hz, 3H), 0.59 (d, *J* = 6.6 Hz, 3H); δ_C_ (150 MHz, CDCl_3_ and DMSO-d_6_) 165.6, 163.1, 130.2, 129.4, 128.7 (×2), 125.4, 123.5, 68.4, 67.7, 64.2, 61.1, 57.5, 42.5, 29.9, 29.4, 27.0, 25.1, 17.9, 17.5, 16.9, 16.2, 13.9, 13.0. Found C: 53.55, H: 6.87, N: 7.78%; C_23_H_35_N_3_Cl_2_O_2_Zn requires C: 52.94, H: 6.76, N: 8.05%. ν_max_(cm^−1^) 3436, 2961, 2923, 2874, 1635, 1604, 1571, 1520, 1464, 1377, 1317, 1300, 1138, 1074, 1047, 1026, 946, 784, 766.

#### Bis-[(4S-isobutyl-4,5-dihydro-oxazol-2-yl)-acetonitrile] zinc(II), 11 [11]

Prepared using the procedure described above for compound **1**, by refluxing a mixture of anhydrous ZnCl_2_ (0.450 g, 3.30 mmol), tetracyanoethylene (1.000 g, 7.81 mmol), and l-leucinol (4.029 g, 34.38 mmol) in 40 mL of dry chlorobenzene for 60 h. The product was obtained in 88% yield as colourless crystals after column chromatography (petroleum ether/dichlormethane, 4/1). m.p. > 220 °C (dec); $\left[ {\alpha } \right]_{\text{D}}^{25}$ = +166.33° (c = 0.30, CH_2_Cl_2_): δ_H_ (500 MHz, CDCl_3_, 27 °C) 4.60 (t, *J* = 7.3 Hz, 4H), 3.94–4.05 (m, 8H), 1.29–1.72 (m, 12H), 0.89–0.93 (m, 24H); δ_C_ (125 MHz, CDCl_3_) 170.1, 118.3, 73.0, 61.6, 45.6, 25.0, 22.3, 21.8. ν_max_(cm^−1^) 3439, 2955, 2927, 2871, 2201, 1611, 1530, 1430, 1386, 1368, 1342, 1281, 1260, 1239 1218, 1133, 1068, 1048, 951, 746. Found: C: 59.32, H: 7.46, N: 13.77%; C_32_H_48_N_6_O_4_Zn requires C: 59.48, H: 7.49, N: 13.01%.

#### Bis-[(4S-phenyl-4,5-dihydro-oxazol-2-yl)-acetonitrile] zinc(II), 12 [12]

Prepared using the procedure described above for compound **1**, by refluxing a mixture of anhydrous ZnCl_2_ (0.450 g, 3.30 mmol), tetracyanoethylene (1.000 g, 7.81 mmol), and *L*-phenylglycinol (10.089 g, 7.35 mmol) in 40 mL of dry chlorobenzene for 60 h. The product was obtained in 86% yield as colourless crystals after column chromatography (petroleum ether/CH_2_Cl_2_, 2/1) m.p. > 220 °C (dec), $\left[ {\alpha } \right]_{\text{D}}^{25}$ = +306.6° (c = 0.17, CH_2_Cl_2_). δ_H_ (500 MHz, CDCl3, 27 °C) 7.22–7.26 (m, 12H), 6.82 (d, *J* = 6.9 Hz, 8H), 4.50–4.60 (m, 8H), 3.95 (t, *J* = 7.2 Hz, 4H); δ_C_ (125 MHz, CDCl_3_) 171.3, 138.6, 129.3 (×2), 129.1, 126.8 (×2), 118.5, 74.6, 67.4; ν_max_(cm^−1^) 3032, 2903, 2202, 1608, 1526, 1429, 1455, 1362, 1264, 1220, 1075, 1051,911, 734, 701. Found C: 65.99, H: 4.20, N: 11.28%; C_40_H_32_N_6_O_4_Zn requires C: 66.17, H: 4.44, N: 11.57%.

#### [3-(4S-isopropyl-4,5-dihydro-oxazol-2-yl)-6-methyl-2-ol]zinc(II) chloride dimer, 13

Prepared using the procedure described above for compound **1**, by refluxing a mixture of anhydrous ZnCl_2_ (3.502 g, 25.70 mmol), 2-hydro-6-methyl-nicotinonitrile (2.002 g, 14.92 mmol) and l-valinol (8.025 g, 77.79 mmol) in 40 mL of dry chlorobenzene for 60 h. The product was obtained in 80% yield as colourless crystals after column chromatography (petroleum ether/CH_2_Cl_2_, 4/1) m.p. 168–170 °C, $\left[ {\alpha } \right]_{\text{D}}^{25}$ = +162.8° (c = 0.181, MeOH); δ_H_ (600 MHz, CDCl_3_ and DMSO-d_6_, 27 °C) 12.36(br s, 1H), 8.27 (d, *J* = 7.7 Hz, 2H), 6.57 (d, *J* = 7.7 Hz, 2H), 4.56–4.58 (m, 2H), 4.50–4.53 (m, 2H), 4.37–4.39 (m, 2H), 2.68 (s, 6H), 2.16–2.18 (m, 2H), 0.99 (d, *J* = 6.9 Hz, 6H), 0.93 (d, *J* = 6.7 Hz, 6H); δ_C_ (150 MHz, DMSO-d_6_)164.0, 162.5, 155.1 (×2), 146.7 (x4), 110.1 (×2), 108.5 (×2), 69.6 (×2), 68.2 (×2), 30.0 (×2), 20.1 (×2), 18.9 (×2), 15.0 (×2); ν_max_(cm^−1^) 3420, 2962, 2928, 2874, 1726, 1660, 1612, 1564, 1388, 1325, 1214, 1150, 1084, 986, 953, 790, 750, 701, 597, 469. Found C: 37.52, H: 4.22, N: 7.28%; C_25_H_32_Cl_6_N_4_O_4_Zn_2_ (CHCl_3_ solvate) requires C: 37.72, H: 4.05, N: 7.04%.

#### Tetra-[3-(4S-isobutyl-4,5-dihydro-oxazol-2-yl)-6-methyl-2-ol] zinc(II) chloride, 14

Prepared using the procedure described above for compound **1**, by refluxing a mixture of anhydrous ZnCl_2_ (1.500 g, 11.01 mmol), 2-hydro-6-methyl-nicotinonitrile (1.002 g, 7.47 mmol) and *L*-leucinol (4.022 g, 34.32 mmol) in 40 mL of dry chlorobenzene for 60 h. The product was obtained in 86% yield as colourless crystals after column chromatography (petroleum ether/CH_2_Cl_2_, 1/1). m.p. 120–124 °C, $\left[ {\alpha } \right]_{\text{D}}^{25}$ =+30.0° (c = 0.08, THF). δ_H_ (600 MHz, CDCl_3_, 27 °C), 8.02 (d, *J* = 7.8 Hz, 2H), 7.98 (d, *J* = 7.8 Hz, 2H), 6.15 (d, *J* = 5.5 Hz, 2H), 6.14 (d, *J* = 5.4 Hz, 2H), 4.86 (t, *J* = 8.7 Hz, 2H), 4.48–4.56 (m, 6H), 4.29 (d, *J* = 7.8 Hz, 1H), 4.28 (d, *J* = 7.9 Hz, 1H), 3.94 (t, *J* = 8.6 Hz, 2H), 2.41(s, 6H), 2.44(s, 6H), 1.90–1.94 (m, 2H), 1.57–1.69 (m, 6H), 1.21–1.43 (m, 4H), 0.82 (t, *J* = 7.5 Hz, 12H), 0.74 (d, *J* = 6.6 Hz, 6H), 0.57 (d, *J* = 6.6 Hz, 6H). δ_C_ (150 MHz, CDCl_3_) 167.7, 167.5, 165.3, 164.8, 163.4, 163.2, 143.4, 143.3, 111.7, 111.5, 105.3, 105.1, 73.0, 72.8, 63.8, 63.4, 43.9, 43.1, 26.1 (×2), 25.3, 25.2, 22.7, 22.6, 22.5 (×2); ν_max_(cm^−1^) 2957, 2929, 2870, 1648, 1579, 1490, 1386, 1322, 1284, 1250, 1205, 1153, 1077, 1060, 953, 883, 787, 749, 707, 620, 595, 419. Found C: 46.98, H: 5.12, N: 7.99%; C_52_H_68_Cl_4_N_8_O_8_Zn_4_ requires C: 46.73, H: 5.13, N: 8.38%.

#### Tetra-{3-[4(R)-benzyl-4,5-dihydro-oxazol-2-yl]-6-methyl-2-ol}zinc complex, 15

Prepared using the procedure described above for compound **1**, by refluxing a mixture of anhydrous ZnCl_2_ (1.562 g, 11.46 mmol), 2-hydro-6-methyl-nicotinonitrile (1.000 g, 7.46 mmol), and *D*-phenylalaninol (4.008 g, 26.51 mmol) in 40 mL of dry chlorobenzene for 60 h. The product was obtained in 82% yield as colourless crystals after column chromatography (petroleum ether/CH_2_Cl_2_, 1/2). m.p. 120–124 °C, $\left[ {\alpha } \right]_{\text{D}}^{25}$ = −109.0° (c = 0.164, THF); δ_H_ (600 MHz, DMSO-d_6_, 27 °C) 12.36–12.41 (m, 3H), 9.78 (d, *J* = 8.0 Hz, 4H), 8.11 (d, *J* = 7.2 Hz, 2H), 7.13–7.22 (m, 17H), 6.23(d, *J* = 7.2 Hz, 2H), 4.90 (s, 3H), 4.08 (d, *J* = 5.2 Hz, 3H), 3.34–3.40 (m, 6H), 2.84–2.88 (m, 4H), 2.69–2.73 (m, 4H), 2.23 (s, 12H),δ_C_ (150 MHz, DMSO-d_6_) 162.7, 162.4, 150.3, 143.5, 138.5, 128.9 (×2), 127.8 (×2), 125.7, 116.7, 105.4, 61.6, 51.8, 36.6, 18.3. ν_max_(cm^−1^) 3435, 3061, 2922, 1644, 1581, 1488, 1454, 1385, 1323, 1245, 1206, 1152, 1085, 1059, 1031, 986, 968, 786, 784, 704, 619, 510. Found C: 52.03, H: 4.38, N: 7.25%; for C_64_H_60_N_8_O_8_Zn_4_Cl_4_ requires C: 52.20, H: 4.11, N: 7.61%.

## Conclusions

One-pot synthesis of oxazolinyl-zinc(II) complexes from three-component reactions between ZnCl_2_, amino alcohols and a variety of nitrile precursors has been demonstrated. The reaction outcome is highly dependent upon the presence of additional donor atoms, reaction stoichiometry and nature of the δ-substituent at the stereogenic centre, giving rise to a variety of coordination modes, including mono- and bis-chelate complexes. Using excess of zinc salt led to the formation of multinuclear complexes.

## Additional files

**Additional file 1.** Table, figures, crystal data and structure determination, general remarks, and procedure for the synthesis of the complexes 1-15.

**Additional file 2.** Copies of NMR spectra.
