# Erratum to: One-step multicomponent synthesis of chiral oxazolinyl-zinc complexes

**DOI:** 10.1186/s13065-017-0315-z

**Published:** 2017-09-20

**Authors:** Mei Luo, Jing Cheng Zhang, Wen Min Pang, King Kuok Hii

**Affiliations:** 1 College of Chemistry and Chemical Engineering, Hefei University of Technology, Hefei, 230009 People’s Republic of China; 2 Department of Chemistry, University of Science and Technology of China, Hefei, 230009 People’s Republic of China; 3 Department of Chemistry, Imperial College London, Exhibition Road, South Kensington, London, SW7 2AZ UK

## Erratum to: Chem Central J (2017) 11:81 DOI 10.1186/s13065-017-0305-1

After the publication of this work [1] it was noticed that in the legend for scheme 5: ‘(left 11, right 12)’ was accidentally included and should be removed. The drawing for scheme 6 is incorrect and needs to be replaced with the correct version.

We apologise for these errors.

The original article was corrected.
