# Supplementary material for: One-step multicomponent synthesis of chiral oxazolinyl-zinc complexes
Source: Chem Cent J. 2017 Aug 9;11:81. doi: 10.1186/s13065-017-0305-1 (PMC5549684; doi:10.1186/s13065-017-0305-1)
Supplement: Supplementary file 1 — Additional file 1. Table, figures, crystal data and structure determination, general remarks, and procedure for the synthesis of the complexes 1-15. [file 13065_2017_305_MOESM1_ESM.pdf]

Supporting Information

One-step multicomponent synthesis of chiral oxazolinyl-zinc complexes

Mei Luo,[a*] Jing Cheng Zhang,[a] Wen Min Pang [b], King Kuok (Mimi) Hii[c*]

Contents of the supporting information:

Table 1 S1

Figures S2

Crystal data and structure detrmination S3

Thr crstal data for the complex 1-15 S4

General remarks S5

The procedure for the synthesis of the complexes 1-15 S6

S1

Table 1 One-pot Synthesis of Oxazolinyl Zinc Complexes

$ZnCl_2$ (%)	Products	Yield (%)
114.2	1	25
259.8	2	65
152.8	3	86
144.6	4	90
245.8-256.0	5	56
44.1	6	90
121.8	7	80
122.9	8	85
56.1	9	86
72.6	10	90
42.2	11	88
42.2	12	86
172.1	13	86
130.7	14	80
153.6	15	82`

[a]Isolated yield from silica ge9

S2 Figures

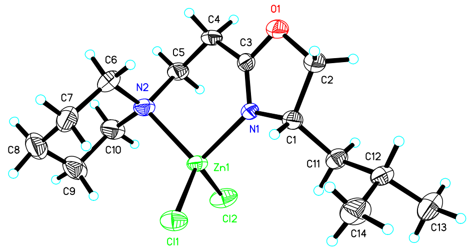

Figure 1.ORTEP diagram of 1.

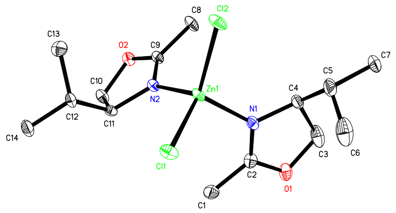

Figure 2.ORTEP diagram of 2.

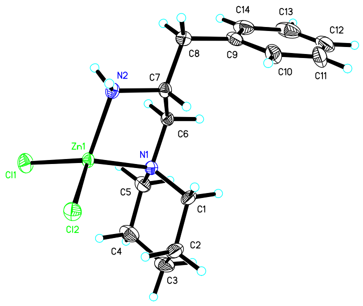

Figure 3.ORTEP diagram of 3

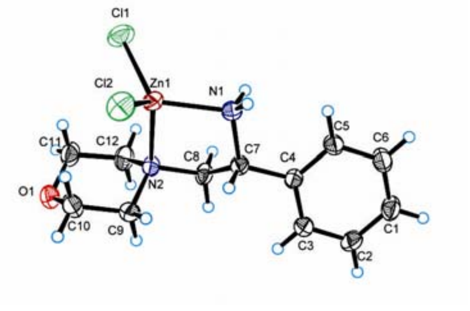

Figure 4.ORTEP diagram of 4.

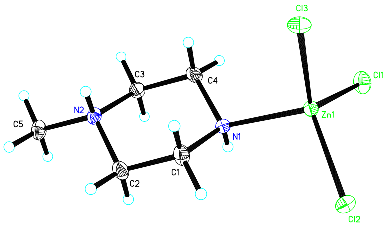

Figure 5.ORTEP diagram of 5

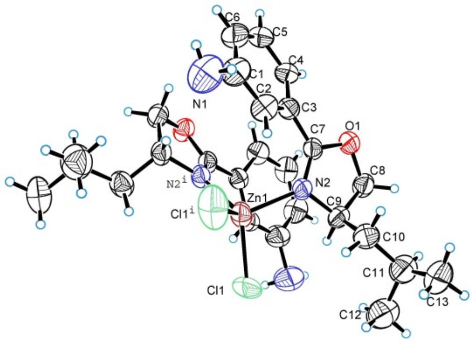

Figure 6.ORTEP diagram of 6

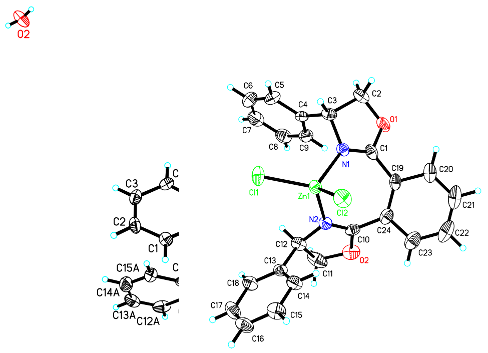

Figure 7.ORTEP diagram of 7

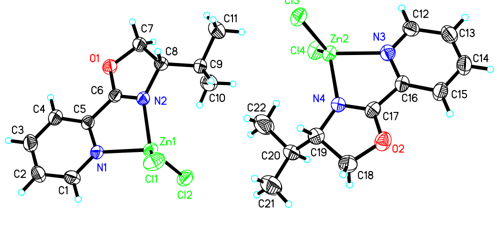

Figure 8.ORTE diagram of 8.

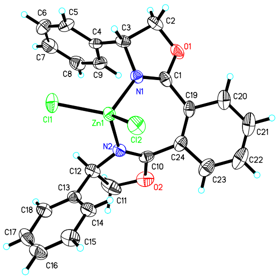

Figure 9.ORTEP diagram of 9.

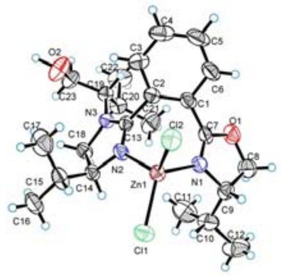

Figure 10.ORTEP diagram of 10

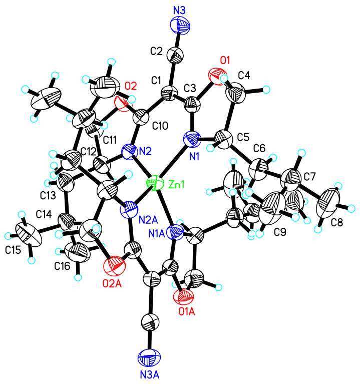

Figure 11.ORTEP diagram of 11.[**cited from scheme 5]**

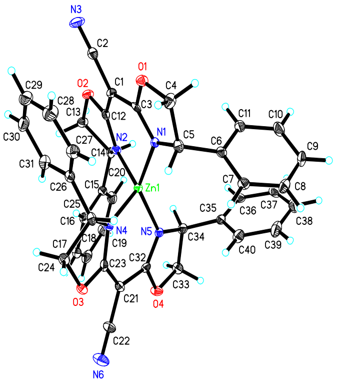

Figure 12.ORTEP diagram of 12. [**cited from scheme 5**]

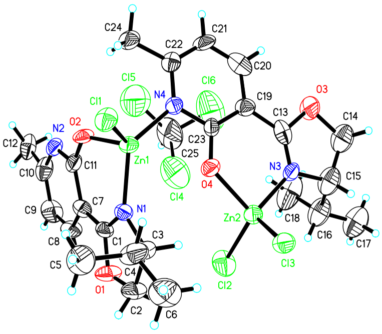

Figure 13.ORTEP diagram of 13.

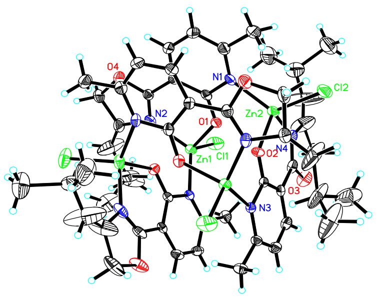

Figure 14.ORTEP diagram of 14. [**cited from scheme 6**]

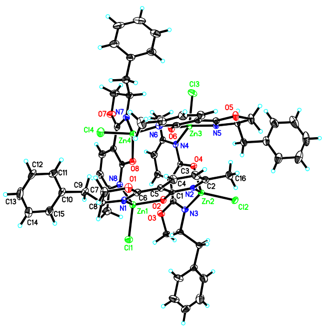

Figure 15.ORTEP diagram of 15. [**cited from scheme 6**]

S3

Crystal data and structure detrmination

The data was collected on Bruker SMART diffractometer equipped with graphite-monochromatic Mo*K*α(o.71073 Å) or Cu*k*α(1.54178 Å).The structure was solved by full-matrix least-squares on F^2 using the SHELXTL program. All non-H atoms were refined with anisotropic displance parameters. All-hydrogen atoms were located theoretically and refined with riding medel position parameters and fixed isotropic thermal parameters.

S4

Thr crstal data for the complex 1-15

The colorless plate crystal of the title compound **1** of approximately 0.176x 0.145 x 0.037 mm was selected for the data collection on a “graphite” diffractometer with mirror monochromated MoK/αradiation (λ=71073Å). A total of 10642 reflections were collected in the range of 1.83 < θ < 26.00° by using “phi and omega scans” techniques at 293(2) K, $C_{14}H_{26}Cl_2N_2OZn$, M = 374.64, orthorhomboic, $P2_12_12_1$, a = 7.3809(16)Å, α= 90º , b = 10.831(2) Å, = 90 º, c = 22.238(5) Å, γ= 90º , V = 1777.8(7), Z = 4, Dcalc. = 1.400 mg/m3 , the final R factor was R1 = 0.0812, for reflections with I> 2 sigma, R=0.0478 for all data. The structure was solved by full-matrix least-squares on F2 using the SHELXTL PROGREM .

The colorless plate crystal of the title compound **2** of approximately 0.22 x 0.16 x 0.10 mm was selected for the data collection on a “graphite” diffractometer with mirror monochromated MoK/αradiation (λ=0.71073Å). A total of 8700 reflections were collected in the range of 2.36 <θ< 30.59° by using “phi and omega scans” techniques at 140(2) K, $C_{14}H_{26}Cl_2N_2O_2Zn$, M = 390.64, monoclinic, P2(1), a = 6.494(2)Å, b = 16.648(5) Å, c = 9.129(3) Å, α= 90º, β=109.082(5)º, γ= 90º , V = 932.6(5), Z = 2, Dcalc. = 1.391 mg/m^3 , the final R factor was R1 = 0.0455 for reflections with I> 2 sigma, R=0.0551 for all data. The structure was solved by full-matrix least-squares on F2 using the SHELXTL

A colorless plate crystal of the title compound **3** of approximately 0.50 x 0.15 x 0.10 mm was selected for the data collection on a “graphite” diffractometer with mirror monochromated MoK/αradiation (λ=0.71073 Å) A total of 7602 reflections were collected in the range of 1.45 <θ<26.98° by using “phi and omega scans” techniques at 133(2) K, $C_{15}H_{23}Cl_5N_2Zn$, M = 473.97, Tetragonal, I4, a = 19.852(3)Å, α= 90º , b = 19.852(3) Å, β= 90º, c = 10.7639(16) Å, γ= 90º , V = 4241.9(11), Z = 8, Dcalc. = 1.484 mg/m3 , the final R factor was R1 = 0.0282, for reflections with I> 2 sigma, R=0.0299 for all data.

The colorless plate crystal of the title compound **4** of approximately 0.36 x 0.32 x 0.30mm was selected for the data collection on a “graphite” diffractometer with mirror monochromated CuK/αradiation (λ=1.54184Å). A total of 10362 reflections were collected in the range of 5.26 < θ < 69.80° by using “phi and omega scans” techniques at 291(2)K, $C_{12}H_{18}OZnCl_2N_2$, M =342.55,

Orthorhombic, a =9.65410(10) Å, b =10.24400(10) Å, c =14.76100(10) Å, α=90°, β=90°, γ=90°, U =1459.81(2) Å^3, space group $P2_12_12_1$, Z = 4, μ(Mo-Kα) = 5.620, 10362 reflections measured, 2724 unique (R_{int} = 0.0240) which were used in all calculations. The final $wR(F_2)$ was 0.0611 (all data).

The colorless plate crystal of the title compound 5 of approximately 0.22 x 0.17 x 0.10mm was selected for the data collection on a “graphite” diffractometer with mirror monochromated MoK/αradiation (λ=Å). A total of 10387 reflections were collected in the range of 2.37< θ < 30.50° by using “phi and omega scans” techniques at 173(2)K, $C_5H_{13}ZnCl_3N_2$, M =271.89, Monoclinic, a =7.3936(11) Å, b =17.204(3) Å, c =8.4147(13) Å, α=90°, β=90°, γ=90°, U =1069.6(3) Å^3, T =173(2)K, space group $P2_1/n$, Z = 4, μ(Mo-Kα) = 5.620, 10387 reflections measured, 3263 unique (R_{int} = 0.0463) which were used in all calculations. The final $wR(F_2)$ was 0.0694 (all data).

The colorless plate crystal of the title compound 6 of approximately 0.42 x 0.38 x 0.35mm was selected for the data collection on a “graphite” diffractometer with mirror monochromated CuK/αradiation (λ=1.54184Å). A total of 20763 reflections were collected in the range of 4.35 < θ < 69.85° by using “phi and omega scans” techniques at 293(2)K, $C_{13}H_{18}N_2ZnClO$, M =572.86,

Tetragonal, a=11.3057(1), b=11.3057(1) ,c = 23.1376(5) Å, α=90°, β=90°, γ=90°, U = 2957.42(7) Å^3, space group $P4_12_12$ (no. 92), Z = 4, μ(Mo-Kα) = 3.034, 20763 reflections measured, 2786 unique (R_{int} = 0.0283) which were used in all calculations. The final $wR(F_2)$ was 0.1602 (all data).

The colorless plate crystal of the title compound 7 of approximately 0.20 x 0.16 x 0.12mm was selected for the data collection on a “graphite” diffractometer with mirror monochromated MoK/αradiation (λ=0.71073 Å). A total of 20763 reflections were collected in the range of 2.151< θ < 25.997° by using “phi and omega scans” techniques at 293(2)K, $C_{30}H_{30}N_4Zn_2Cl_2O_3$, M =630.85, Monoclinic, a=14.361(2), b=10.2938(14), c=9.4877(14) Å, α=90°, β=93.739(4)°, γ=90°, U =1399.6(4)Å^3, space group C2, Z =2, μ(Mo-Kα) = 1.108, 4216 reflections measured, 2600 unique (R_{int} = 0.0393) which were used in all calculations. The final $wR(F_2)$ was 0.1217 (all data).

The colorless plate crystal of the title compound 8 of approximately 0.212 x 0.167 x 0.132 mm was selected for the data collection on a “graphite” diffractometer with mirror monochromated

MoK/αradiation (λ=0.71073Å). A total of 8481 reflections were collected in the range of 2.19< θ < 25.97° by using "phi and omega scans" techniques at 293(2)K, $C_{11}H_{14}N_2ZnCl2O$, *M* =326.51,Monoclinic, a=9.3099(9),b=9.4799(9), c=15.8234(14) Å,

α=90°,*β* = 90°, *γ*= 90°, U =1395.1(2)Å^3, space group P2$_1$, *Z* = 4, μ(Mo-Kα) = 1.555, 8481 reflections measured, 4331 unique (R_{int} = 0.0272) which were used in all calculations. The final $wR(F_2)$ was 0.0882 (all data).

The colorless plate crystal of the title compound 9 of approximately 0.112 x 0.087 x 0.069 mm was selected for the data collection on a "graphite" diffractometer with mirror monochromated MoK/αradiation (λ=0.71073Å). A total of 6889 reflections were collected in the range of 1.99 < θ < 26.00° by using "phi and omega scans" techniques at 293(2)K, $C_{24}H_{20}N_2ZnCl_2O_2$, *M* =504.69, Monoclinic, P2(1), a=10.4326(8) Å, b=7.5507(6)Å, c=14.5952(13) Å, α=90°,*β* = 90°, *γ*= 90°, U =1130.49(16) Å^3, space group P2$_1$, *Z* = 2, μ(Mo-Kα) = 1.346 6889 reflections measured,4365 unique (R_{int} = 0.0556 which were used in all calculations. The final $wR(F_2)$ was 0.1070 (all data).

The colorless plate crystal of the title compound 10 of approximately 0.40 x 0.38 x 0.32mm was selected for the data collection on a "graphite" diffractometer with mirror monochromated CuK/αradiation (λ=1.54184Å). A total of 13197 reflections were collected in the range of 3.76 < θ < 62.69° by using "phi and omega scans" techniques at 291(2)K, $C_{23}H_{35}O_2ZnCl_2N_3$, *M* =521.81 , Monoclinic, *a* = 9.78320(10) Å, *b* = 11.33510(10) Å, *c* = 11.81900(10) Å, α=90°,*β* = 96.3280(10)°, *γ*= 90°, *U* = 1302.67(2) Å^3, *T* = 291(2), space group P2$_1$, *Z* = 2, μ(Mo-Kα) = 3.376, 13197 reflections measured, 4158 unique (R_{int} = 0.0207) which were used in all calculations. The final $wR(F_2)$ was 0.0645 (all data)

The colorless plate crystal of the title compound 11 of approximately 0.321 x 0.225 x 0.168 mm was selected for the data collection on a "graphite" diffractometer with mirror monochromated MoK/αradiation (λ=0.71073Å). A total of 16046 reflections were collected in the range of 1.95 < θ < 25.99 ° by using "phi and omega scans" techniques at 293 2)K, $C_{32}H_{48}O_4ZnN_6$, *M* =646.13,Trigonal, *a* = 12.0352(4) Å, *b* = 12.0352(4) Å, *c* =20.9216 (10) Å, α=90°, β=90°, *γ*= 120°, *U* = 2624.41(18) Å^3, 'space group trigonal P3$_2$2$_1$, *Z* = 3, μ(Mo-Kα) = 0.744, 16046 reflections measured, 3450 unique (R_{int} = 0.0323) which were used in all calculations. The final $wR(F_2)$ was 0.0935 (all data).

The colorless plate crystal of the title compound 12 of approximately 0.25 x 0.08 x 0.04 mm was selected for the data collection on a "graphite" diffractometer with mirror monochromated MoK/αradiation (λ=0.71073Å). A total of 16046 reflections were collected in the range of 1.65 < θ < 27.51° by using "phi and omega scans" techniques at 133 2)K, $C_{40}H_{32}O_4ZnN_6$, *M* =726.09,

Orthorhombic, *a* = 10.5470(15) Å, *b* = 15.264(2) Å, *c* =21.146 (3) Å, α=90°, β=90°, *γ*= 90°,

U = 3404.3(8) Å^3, 'space group P2$_1$2$_1$ 2$_1$, *Z* = 4, μ(Mo-Kα) = 0.774, 27153 reflections measured, 7824 unique (R_{int} = 0.0734) which were used in all calculations. The final $wR(F_2)$ was 0.0953 (all data).

The colorless plate crystal of the title compound 13 of approximately 0.165 x 0.134 x 0.058 mm was selected for the data collection on a "graphite" diffractometer with mirror monochromated

MoK/αradiation (λ=0.71073Å). A total of 9654 reflections were collected in the range of 2.15 < θ < 25.50° by using "phi and omega scans" techniques at 293 2)K, $C_{25}H_{32}O_4ZnCl_6N_4$, M =794.98, Orthorhombic, a = 18.253(3) Å, b = 22.150(4) Å, c =9.1847 (17) Å, α=90°,β=90°,γ= 90°, U =3713.3(12) $Å^3$,P'space group $P2_12_12_1$, Z = 4, μ(Mo-Kα) = 1.756, 6915 reflections measured, 6915 unique (R_{int} = 0.0000) which were used in all calculations. The final $wR(F_2)$ was 0.1649 (all data)

The colorless plate crystal of the title compound 14 of approximately 0.212 x 0.145 x 0.113 mm was selected for the data collection on a "graphite" diffractometer with mirror monochromated MoK/αradiation (λ=0.71073Å). A total of 9654 reflections were collected in the range of 0.91 < θ < 26.00° by using "phi and omega scans" techniques at 293 2)K, $C_{26}H_{34}O_4Zn_2Cl_2N_4$, M =668.21,Monoclinic, a = 18.656(13) Å, b = 16.055(11) Å, c =23.275 (16) Å, α=90°,β=105.774(16)°,γ= 90°, U = 6709(8) $Å^3$, 'space group C2, Z = 8, μ(Mo-Kα) = 1.622, 9654 reflections measured, 9654 unique (R_{int} = 0.0000) which were used in all calculations. The final $wR(F_2)$ was 0.1561 (all data).

The colorless plate crystal of the title compound 15 of approximately 0.28 x 0.15 x 0.10 mm was selected for the data collection on a "graphite" diffractometer with mirror monochromated MoK/αradiation (λ=0.71073Å). A total of 45019 reflections were collected in the range of 1.79 < θ < 28.71° by using "phi and omega scans" techniques at 143(2) K, $C_{64}H_{60}O_8Zn_4Cl_4N_8$, M =1472.48, Ttetragonal, a = 16.1089(14) Å, b = 16.1089(14) Å, c =25.737 (2) Å, α=90°, β=90°, γ= 90°, U =6678.7(10) $Å^3$, 'space group $P4_1$ Z = 4, μ(Mo-Kα) = 1.638, 45019 reflections measured, 17062 unique (R_{int} = 0.0452) which were used in all calculation.

S5

General remarks

Unless otherwise stated, all chemical reagents were purchased from Acros, Aldrich, or Fluka USA. Flash column chromatography was performed using Merck silica gel (60, particle size 0.02-0.03 mm). ^{1}H and ^{13}C NMR spectra were recoZrded using Bruker AM-500 or AM-600 spectrometers. Chemical shifts are reported in ppm (δ) with the solvent relative to tetramethylsilane (TMS) employed as the internal standard (residual $CHCl_3$, $δ_H$ 7.26 ppm; $CDCl_3$, $_c$ 77 ppm). The following abbreviations were used to designate multiplicities: s = singlet, d = doublet, t = triplet, m = multiplet. Infrared spectra were recorded on a Mattson Galaxy Series FTIR 3000 spectrometer; peaks are reported in cm^{-1}. Elemental analyses were obtained on Elemental Analyzer AE-3000. High-resolution mass spectra (HRMS) were obtained on a Micro GCT-MS equipped with an EI ion source. Optical rotations were measured on a WZZ-1 automatic polarimeter with a 2-cm cell, recorded at the sodium D-line.

S6

The procedure for the synthesis of the complexes 1-15

1-[2-(4-isobutyl-4,5-dihydro-oxazol-2-yl)-ethyl]-piperidine zinc(II) dichloride, 1. A dry 100 mL Schlenk flask was purged with N_2 and charged with anhydrous $ZnCl_2$ (2.515 g, 18.45 mmol),

3-piperidin 1-yl propionitrile (2.462 g, 17.81 mmol) and *L*-leucinol (4.824 g, 41.16 mmol). 40 mL of chlorobenzene was added, and the reaction mixture was refluxed for 72 h. After cooling to room temperature, the solvent was removed under reduced pressure, and the residue was dissolved in 15 mL of H_2O and extracted with CH_2Cl_2 (3 x 20 mL). The combined organic extracts were evaporated to give a crude red oil, which was purified by column chromatography (petroleum ether/CH_2Cl_2, 4/1) to afford the title compound as colourless crystals in 25% yield, m.p. 50-52°C $[\alpha]_D^{25}$=+67.5° (c = 0.02, MeOH); δ_H (600 MHz, $CDCl_3$, 27 °C) 4.52-4.56 (m, 1H), 4.12-4.16 (m, 1H), 4.01-4.03 (m, 1H), 2.89-2.92 (m, 1H), 2.73-2.75 (m, 1H), 2.59-2.64 (m, 3H), 2.31 (t, *J* =12.4 Hz, 1H), 1.62-1.78 (m, 6H), 1.43-1.44 (m, 2H), 1.29-1.35 (m, 2H), 1.15-1.20 (m, 1H), 0.84-0.90 (m, 6H); $_C$ (150 MHz, DMSO-d_6), 170.0, 73.7 (x2), 65.5, 62.9 (x2), 54.7, 46.1, 44.4, 43.3, 25.1, 24.0, 23.4, 22.6. ν_{max}(cm^{-1}) 3274, 2954, 2869, 1648, 1587, 1468, 1387, 1368, 1319, 1283, 1169, 1076 1041, 979, 956, 949, 904, 864, 839, 780, 607, 493. Found C: 45.36, H: 7.19, N: 7.75%; $C_{14}H_{26}Cl_2N_2OZn$ requires C: 44.88, H: 7.00, N: 7.48%.

***Bis*-[4-isopropyl-2-methyl-4,5-dihydro-oxazole]zinc(II) dichloride, 2.** Prepared using the same procedure described above for complex **1**, from a mixture of $ZnCl_2$ (5.401 g, 39.63 mmol), 3-piperidin1-yl propionitrile (2.321 g, 16.79 mmol) and *L*-valinol (5.319 g, 51.56 mmol) in chlorobenzene (80 mL). The product was obtained as colourless crystals in 65% yield after column chromatography (petroleum ether/CH_2Cl_2, 2/1), m.p. 60-62°C, $[\alpha]_D^{25}$=+9.97° (c = 0.35, MeOH);δ_H (600 MHz, $CDCl_3$, 27 °C) 3.64-3.71 (m, 6H), 2.03 (s, 6H), 1.85-1.88 (m, 2H), 0.92 (d, *J* = 6.8 Hz, 6H), 0.96 (d, *J* = 7.8Hz, 6 H); δ_C (150 MHz, $CDCl_3$), 171.2(x2), 63.7, 63.6, 57.1, 53.5, 31.7, 28.9, 23.4, 22.9, 19.3, 19.0, 18.8, 17.9; ν_{max}(cm^{-1}) 3436, 3284, 3145, 2954, 2864, 1725, 1659, 1585, 1458, 1420, 1393, 1319, 1278, 1280, 1219, 1092, 1038, 974, 952, 905, 765. Found C: 43.49, H: 7.19, N: 7.23%; $C_{14}H_{26}Cl_2N_2O_2Zn$ requires C: 43.04, H: 6.71, N: 7.17%.

α-Phenyl-1-hexahydropyridyl ethylamine zinc(II), 3. Prepared using the same procedure described above, using anhydrous $ZnCl_2$ (3.5002 g, 25.68 mmol), 3-piperidin1-yl propionitrile (2.4590 g, 17.79mmol), and L-phenylalaninol (5.5420 g, 40.40 mmol) in 80 mL of dry chlorobenzene. The product was obtained as colorless crystals after column chromatography (petroleum ether/dichloromethane, 1/2) in 86% yield,.m.p：168-172°C; $[a]^5{}_D$=-29.30º (c=0.016, CH_3OH)：δ_H (600 MHz, $CDCl_3$, 27 °C) 7.32-7.43 (m, 5H), 4.23-4.32 (m, 1H), 3.42-3.64 (m, 3H), 2.96-3.05 (m, 2H), 2.61 -2.65(m, 1H), 2.19-2.41 (t, J =912.4 Hz, 1H), 1.62-1.78 (m, 6H), 1.43-2.41 (m, 3H), 1.69-1.98 (m, 6H) , 1.24-1.31 (m, 1H), δ_C (150 MHz, DMSO-d_6), 169.2, 141.8, 128.9, 128.4, 127.4, 127.3, 127.1 65.2, 65.1, 55.4, 55.2, 53.8, 51.9, 25.3, 23.2(x2); ν_{max} :3447, 3027，2943, 2860, 1648, 1603，1496, 1455, 1132, 1043, 1060,1040, 1030, 762, 705. Elemental analysis: Found C: C:47. 20%, H, 6. 05%, N, 7. 60%; $C_{14}H_{22}Cl_2N_2Zn$ requires C: 47.42, H: 6.25, N: 7.90%.

2-Morpholin-4-(R)-yl-1-phenyl-ethylamine zinc(II) dichloride complex, 4. Prepared as described above, from a mixture of anhydrous $ZnCl_2$ (1.780 g, 13.06 mmol), *N*-cyanoacetylmorpholine (1.501 g, 9.74 mmol), *D*-phenylglycinol (4.097 g, 29.87mmol) and dry chlorobenzene (40 mL). The reaction mixture was refluxed for 60 h. The product was purified by column chromatography (petroleum ether/CH_2Cl_2, 1/100) to afford the title compound as colourless crystals in 90% yield, m.p.196–198 °C, $[\alpha]^{25}_D$ = –42.43° (c = 0.13, THF); δ_H (500 MHz, DMSO-d_6, 27℃), 7.48 (d, *J* = 8.8 Hz, 2H), 7.36 (t, *J* = 7.5 Hz, 2H), 7.30 (t, *J* = 7.3 Hz, 1H), 4.95 (br s, 2H), 4.14 (t, *J* = 11.9 Hz, 1H), 3.88-3.91 (m, 2H), 3.78-3.81 (m, 2H), 2.97-3.00 (m, 2H), 2.67-2.82 (m, 4H); δ_C (125 MHz, DMSO-d_6) 139.9, 128.5 (x2), 127.9, 127.0 (x2), 65.6, 65.2 (x2), 54.6 (x2), 51.2; ν_{max}(cm^{-1}) 3435, 3271, 3228, 3145, 2974, 2928, 2904, 2865, 1591, 1499, 1458,

1447, 1290, 1263, 1146, 1126, 1094, 1072, 1060, 1037, 988, 899, 874, 749, 694. Found C: 42.39, H: 5.24, N: 8.05%; $C_{12}H_{18}N_2Cl_2OZn$ requires C: 42.07, H: 5.30, N: 8.18%.

(1-Methyl-piperazine)zinc(II) trichloride, 5. Prepared using the same procedure described for complex **4**, from a mixture of anhydrous $ZnCl_2$ (5.008 g, 36.75 mmol), 1-(2-cyanoethyl)-4-methylpiperazine (2.313 g, 15.09mmol) and *D*-phenylglycinol (10.696 g, 77.97 mmol) in 40 mL of dry chlorobenzene. The product was recrystallized from ethanol/CH_2Cl_2, to furnish colourless crystals in 56% yield; m.p. 148–152°C; δ_H (600 MHz, $CDCl_3$ and DMSO-d_6, 27℃) 4.09-4.12 (m, 1H), 3.64-3.67 (m, 1H), 3.56-3.59 (m, 1H), 2.86-2.88 (m, 4H), 2.37-2.40 (m, 3H), 2.16 (s, 3H); δ_C (150 MHz, $CDCl_3$ and DMSO-d_6) 62.1, 54.7, 51.4, 44.0, 42.5. ν_{max}(cm^{-1}) 3491, 3455, 3189, 3006, 2956, 2771, 1585, 1458, 1387, 1128, 1099, 1058, 1035, 998, 976, 870, 701. Found: C: 22.20, H: 4.56, N: 10.10%; $C_5H_{13}Cl_3N_2Zn$ requires C: 22.01, H: 4.80, N: 10.27%.

2-[4*R*-4,5-dihydro-4-(1',1'-dimethylethyl)-3-oxazolinyl]aniline zinc(II) dichloride, 6. Prepared using the same procedure described for complex **1**, from a mixture of anhydrous $ZnCl_2$ (3.002 g, 22.02 mmol), 3-amino-benzonitrile (6.702 g. 56.73 mmol), and D-leucinol (10.008 g, 85.40 mmol) in 80 mL of dry chlorobenzene. The reaction mixture was refluxed for 72 h. After evaporation, the residue was dissolved in 15 mL of H_2O and extracted with CH_2Cl_2 (3 x 10 mL). The organic layer was evaporated under vacuum, and the red oily residue was purified by column chromatography over silica gel (petroleum ether/CH_2Cl_2, 1/4), yield: 90%; m.p. 168–170°C, $[\alpha]^{25}_D$ = –54.9° (c = 0.0364, EtOH). δ_H (600 MHz, $CDCl_3$ and DMSO-d_6, 27℃) 7.77-7.90 (m, 1H), 6.98-7.19 (m, 5H), 6.63-6.76 (m, 2H), 5.19-5.33 (m, 1H), 4.26-4.58 (m, 4H), 3.92-3.93 (m, 1H), 3.21-3.25 (m, 4H), 1.70-1.82 (m, 4H), 1.35-1.44 (m, 2H), 0.89-0.96 (m, 12H); δ_C (150 MHz, $CDCl_3$ and DMSO-d_6) 167.0, 161.3, 146.9, 146.4, 134.2, 126.9, 115.0, 113.9, 113.0, 112.8, 111.5(x2), 109.9(x2), 70.9, 63.0, 46.5, 45.3, 43.7, 39.5, 23.5, 23.2, 21.5, 21.1, 21.0, 20.3; ν_{max}(cm^{-1}) 3353, 2957, 2928, 2870, 1625, 1498, 1467, 1386, 1333, 1290, 1171, 1135, 1108, 996, 966, 948, 882, 797, 750, 688, 576, 537. Found C: 54.65, H: 6.24, N: 10.16%; $C_{26}H_{36}N_4Cl_2O_2Zn$ requires C: 54.51, H: 6.33, N: 9.78%.

***Bis*-[2-(4R-benzyl-4,5dihydro-oxazol-2-yl)-pyridine]zinc(II) tetrachlorozincate, 7**. Prepared using the same procedure described for compound **1**, using anhydrous $ZnCl_2$ (3.340 g, 24.51 mmol), 2-cyanopyridine (2.095 g, 20.13 mmol) and *L*-phenylalaninol (3.992 g, 26.40 mmol) in 40 mL of dry chlorobenzene, and the reaction mixture was refluxed for 60 h. The product was extracted into CH_2Cl_2 as described above, and purified by column chromatography (petroleum ether/CH_2Cl_2, 1/4) to give colourless crystals in 80% yield; m.p. 134–136°C, $[\alpha]^{25}_D$= +51.4° (c = 0.0272, MeOH); δ_H (600 MHz, DMSO-d_6, 27 ℃) 8.78-8.81 (m, 2H), 7.95-8.03 (m, 4H) , 7.63-7.68 (m, 2H), 7.22-7.30 (m, 10H), 4.64-4.67 (m, 4H), 4.49-4.51 (m, 2H), 3.30-3.39 (m, 2H), 3.35 (s,2H), 2.82-2.86 (m, 2H); δ_C (150 MHz, DMSO-d_6) 163.8, 148.5, 137.7, 135.5, 128.2, 127.6, 126.7, 125.7, 122.4, 73.6, 64.5, 39.2; ν_{max}(cm^{-1}) 3493, 3061, 3027, 2955, 2920, 2853, 1660, 1590, 1571, 1492, 1469, 1452, 1440, 1404, 1388, 1325, 1293, 1244, 1223, 1154, 1143, 1088, 1045, 1014, 947, 847, 801, 746, 703, 681, 632; Found C: 57.43, H: 5.03, N: 8.67%; $C_{30}H_{30}Cl_2N_4O_3Zn_2$ requires C: 57.12, H: 4.79, N: 8.88 %.

[2-(4*S*-isopropyl-4,5dihydro-oxazol-2-yl)-pyridine] zinc (II) dichloride, 8. Prepared using the procedure described above for compound **1**, refluxing a mixture of anhydrous $ZnCl_2$ (3.423 g, 25.12 mmol), 2-cyanopyridine (2.128 g, 20.44 mmol), and *L*-valinol (3.386 g, 32.82 mmol) in 40

mL of dry chlorobenzene for 60 h. The product was purified by column chromatography (petroleum ether/CH_2Cl_2, 1/8). Colourless crystals were obtained in 85% yield; m.p. 178–180°C, $[\alpha]^{25}_D$ = +23.1° (c = 0.17, MeOH); δ_H (600 MHz,$CDCl_3$, 27 °C) 8.78-8.80 (m, 1H), 8.20-8.23 (m, 1H), 8.05 (d, *J* = 7.7Hz, 1H), 7.86-7.88 (m, 1H), 4.96 (t, *J* = 9.5 Hz, 1H), 4.65 (t, *J* = 8.9 Hz, 1H), 4.42-4.45 (m, 1H), 2.10-2.14 (m, 1H), 1.04-1.14 (m, 6H); δ_C (150 MHz, $CDCl_3$) 166.1, 149.8, 141.2, 140.2, 129.7, 124.1, 75.4, 69.3, 31.6, 18.4, 17.8;ν_{max}(cm^{-1}) 3223, 3188, 2962, 2875, 1662, 1587, 1470, 1392, 1372, 1320, 1251, 1129, 1046, 878, 836, 791, 752, 690, 539. Found C: 40.81, H: 3.85, N: 8.47%; $C_{11}H_{14}Cl_2N_2OZn$ requires C: 40.46, H: 4.32, N: 8.58%.

[1, 2-*bis*–(4*R*-phenyl-4,5-dihydro-oxazol-2-yl)phenyl] zinc(II) dichloride, 9. Prepared using the same procedure described above for compound **1**, using anhydrous $ZnCl_2$ (2.590 g, 19.01 mmol), isophthalonitrile (3.353 g, 26.17mmol), and *D*-phenylglycinol (8.492 g, 61.91 mmol) in 80 mL of dry chlorobenzene, and refluxing for 72 h. The product was purified by column chromatography (petroleum ether/CH_2Cl_2, 1/8). Yield = 86%; m.p. > 250°C (dec), $[\alpha]^{25}_D$ = -54.9° (c = 0.0364, EtOH).δ_H (600 MHz, $CDCl_3$, 27°C) 7.77-7.79 (m, 2H), 7.55-7.56 (m, 2H), 7.18-7.28 (m, 10H), 5.28 (t, *J* = 9.2 Hz, 2H), 4.68 (t, *J* = 9.2 Hz, 2H), 4.10 (t, *J* = 8.4Hz, 2H),δ_C (150 MHz, $CDCl_3$) 163.5, 140.3, 129.4 (x2), 128.4, 127.0 (x2), 126.0,125.3 (x2), 73.9, 68.3.ν_{max}(cm^{-1}) 3447, 3058, 2965, 2907, 1650, 1639, 1592, 1495,1473, 1455, 1379，1363，1318，1308，1278，1238，1207, 1153, 1120, 1067, 1020, 991, 945, 760, 704, 648, 594, 556. Found C: 56.92, H: 3.92, N: 5.41%; $C_{24}H_{20}Cl_2N_2O_2Zn$ requires C: 57.11, H: 3.99, N: 5.55%.

2-{(4*S*-isopropyl-4,5-dihydro-oxazol-2-yl)-phenyl-4,5-dihydro-imidazol-1-yl}-3-methyl-butan -1-ol zinc(II), 10. Prepared using the same procedure described for compound **1**, refluxing a mixture of anhydrous $ZnCl_2$ (4.000 g, 29.35 mmol), isophthalonitrile (6.700 g, 52.29 mmol), and *L*-valinol (16.000 g, 15.51 mmol) in 80 mL of dry chlorobenzene for 72 h. The product was purified by column chromatography (petroleum ether/CH_2Cl_2, 1/4). Yield: 90%; m.p. >250°C (dec), $[\alpha]^{25}_D$ = +34.4° (c = 0.0436, $CHCl_3$),δ_H (600 MHz, DMSO-d_6, 27 °C), 7.78-7.81 (m, 2H), 7.69-7.71 (m, 1H), 7.63-7.66 (m, 1H), 4.90-4.93 (m, 1H), 4.65 (t, *J* = 9.4Hz, 1H), 4.51-4.55 (m, 1H), 4.45 (t, *J* = 7.8 Hz, 2H), 4.27 (t, *J* = 5.0 Hz, 1H), 3.75 (d, *J* = 11.2 Hz, 1H), 3.62-3.65 (m 2H), 3.47-3.50 (m, 1H), 2.21-2.24 (m, 1H), 1.70-1.74 (m, 1H), 0.94-0.99 (m, 8H), 0.85-0.86 (m, 4H), 0.72 (d, *J* = 6.6 Hz, 3H), 0.59 (d, *J* = 6.6 Hz, 3H); δ_C (150 MHz, $CDCl_3$ and DMSO-d_6) 165.6, 163.1, 130.2, 129.4, 128.7 (x2), 125.4, 123.5, 68.4, 67.7, 64.2, 61.1, 57.5, 42.5, 29.9, 29.4, 27.0, 25.1, 17.9, 17.5, 16.9, 16.2, 13.9, 13.0. Found C: 53.55, H: 6.87, N: 7.78%; $C_{23}H_{35}N_3Cl_2O_2Zn$ requires C: 52.94, H: 6.76, N: 8.05%. ν_{max}(cm^{-1}) 3436, 2961, 2923, 2874, 1635, 1604, 1571, 1520, 1464, 1377, 1317, 1300, 1138, 1074, 1047, 1026, 946, 784, 766.

***Bis*-[(4S-isobutyl-4,5-dihydro-oxazol-2-yl)-acetonitrile] zinc(II), 11**.[11] Prepared using the procedure described above for compound **1**, by refluxing a mixture of anhydrous $ZnCl_2$ (0.450 g, 3.30 mmol), tetracyanoethylene (1.000 g, 7.81 mmol), and L-leucinol (4.029 g, 34.38 mmol) in 40 mL of dry chlorobenzene for 60 h. The product was obtained in 88% yield as colourless crystals after column chromatography (petroleum ether/dichlormethane, 4/1). m.p. >220°C (dec); $[\alpha]^{25}_D$ = +166.33° (c=0.30, CH_2Cl_2): δ_H (500 MHz, $CDCl_3$, 27°C) 4.60 (t, *J* = 7.3 Hz, 4H), 3.94-4.05 (m, 8H), 1.29-1.72 (m, 12H), 0.89-0.93 (m, 24H); δ_C (125 MHz, $CDCl_3$) 170.1, 118.3, 73.0, 61.6, 45.6, 25.0, 22.3, 21.8. ν_{max}(cm^{-1}) 3439, 2955, 2927, 2871, 2201, 1611, 1530, 1430, 1386, 1368, 1342, 1281, 1260, 1239 1218, 1133, 1068, 1048, 951, 746. Found: C: 59.32, H: 7.46, N: 13.77%; $C_{32}H_{48}N_6O_4Zn$ requires C: 59.48, H: 7.49, N: 13.01%.

***Bis*-[(4S-phenyl-4,5-dihydro-oxazol-2-yl)-acetonitrile] zinc(II), 12.**[12] Prepared using the

procedure described above for compound **1**, by refluxing a mixture of anhydrous $ZnCl_2$ (0.450 g, 3.30 mmol), tetracyanoethylene (1.000 g, 7.81 mmol), and *L*-phenylglycinol (10.089 g, 7.35 mmol) in 40 mL of dry chlorobenzene for 60 h. The product was obtained in 86% yield as colourless crystals after column chromatography (petroleum ether/CH_2Cl_2, 2/1) m.p. >220 °C (dec), $[\alpha]^{25}_D$ = +306.6° (c = 0.17, CH_2Cl_2). δ_H (500 MHz, CDCl3, 27 ℃) 7.22-7.26 (m, 12H), 6.82 (d, *J* = 6.9 Hz, 8H), 4.50-4.60 (m, 8H), 3.95 (t, *J* = 7.2Hz, 4H); δ_C (125 MHz, $CDCl_3$) 171.3, 138.6, 129.3 (x2), 129.1, 126.8 (x2), 118.5, 74.6, 67.4; ν_{max}(cm^{-1}) 3032, 2903, 2202, 1608, 1526, 1429, 1455, 1362, 1264, 1220, 1075, 1051， 911, 734, 701. Found C: 65.99, H: 4.20, N: 11.28%; $C_{40}H_{32}N_6O_4Zn$ requires C: 66.17, H: 4.44, N: 11.57%.

[3-(4*S*-isopropyl-4,5-dihydro-oxazol-2-yl)-6-methyl-2-ol]zinc(II) chloride dimer, 13. Prepared using the procedure described above for compound **1**, by refluxing a mixture of anhydrous $ZnCl_2$ (3.502 g, 25.70 mmol), 2-hydro-6-methyl-nicotinonitrile (2.002 g, 14.92 mmol) and *L*-valinol (8.025 g, 77.79 mmol) in 40 mL of dry chlorobenzene for 60 h. The product was obtained in 80% yield as colourless crystals after column chromatography (petroleum ether/CH_2Cl_2, 4/1) m.p. 168–170 °C, $[\alpha]^{25}_D$ = +162.8° (c = 0.181, MeOH); δ_H (600 MHz, $CDCl_3$ and DMSO-d_6, 27°C) 12.36(br s, 1H), 8.27 (d, *J* = 7.7Hz, 2H), 6.57 (d, *J* = 7.7Hz, 2H), 4.56-4.58 (m, 2H), 4.50-4.53 (m, 2H), 4.37-4.39 (m, 2H), 2.68 (s, 6H), 2.16-2.18 (m, 2H), 0.99 (d, *J* = 6.9Hz, 6H), 0.93 (d, *J* = 6.7Hz, 6H); δ_C (150 MHz, DMSO-d_6)164.0, 162.5, 155.1 (x2), 146.7 (x4), 110.1 (x2), 108.5 (x2), 69.6 (x2), 68.2 (x2), 30.0 (x2), 20.1 (x2), 18.9 (x2), 15.0 (x2); ν_{max}(cm^{-1}) 3420, 2962, 2928, 2874, 1726, 1660, 1612, 1564, 1388, 1325, 1214, 1150, 1084, 986, 953, 790, 750, 701, 597, 469. Found C: 37.52, H: 4.22, N: 7.28%; $C_{25}H_{32}Cl_6N_4O_4Zn_2$ ($CHCl_3$ solvate) requires C: 37.72, H: 4.05, N: 7.04%.

Tetra-[3-(4*S*-isobutyl-4,5-dihydro-oxazol-2-yl)-6-methyl-2-ol] zinc(II) chloride, 14. Prepared using the procedure described above for compound **1**, by refluxing a mixture of anhydrous $ZnCl_2$ (1.500 g, 11.01 mmol), 2-hydro-6-methyl-nicotinonitrile (1.002 g, 7.47 mmol) and *L*-leucinol (4.022 g, 34.32 mmol) in 40 mL of dry chlorobenzene for 60 h. The product was obtained in 86% yield as colourless crystals after column chromatography (petroleum ether/CH_2Cl_2, 1/1). m.p. 120–124 °C, $[\alpha]^{25}_D$ =+30.0° (c= 0.08, THF). δ_H (600 MHz, $CDCl_3$, 27°C), 8.02 (d, *J* = 7.8 Hz, 2H), 7.98 (d, *J* = 7.8 Hz, 2H), 6.15 (d, *J* = 5.5 Hz, 2H), 6.14 (d, *J* = 5.4 Hz, 2H), 4.86 (t, *J* = 8.7 Hz, 2H), 4.48-4.56 (m, 6H), 4.29 (d, *J* = 7.8 Hz, 1H), 4.28 (d, *J* = 7.9 Hz, 1H), 3.94 (t, *J* = 8.6 Hz, 2H), 2.41(s, 6H), 2.44(s, 6H), 1.90-1.94 (m, 2H), 1.57-1.69 (m, 6H), 1.21-1.43 (m, 4H), 0.82 (t, *J* = 7.5 Hz, 12H), 0.74 (d, *J* = 6.6 Hz, 6H), 0.57 (d, *J* = 6.6 Hz, 6H).δ_C (150 MHz, $CDCl_3$) 167.7, 167.5, 165.3, 164.8, 163.4, 163.2, 143.4, 143.3, 111.7, 111.5, 105.3, 105.1, 73.0, 72.8, 63.8, 63.4, 43.9, 43.1, 26.1 (x2), 25.3, 25.2, 22.7, 22.6, 22.5 (x2); ν_{max}(cm^{-1}) 2957, 2929, 2870, 1648, 1579, 1490, 1386, 1322, 1284, 1250, 1205, 1153, 1077, 1060, 953, 883, 787, 749, 707, 620, 595, 419. Found C: 46.98, H: 5.12, N: 7.99%; $C_{52}H_{68}Cl_4N_8O_8Zn_4$ requires C: 46.73, H: 5.13, N: 8.38%.

Tetra-{3-[4(*R*)-benzyl-4,5-dihydro-oxazol-2-yl]-6-methyl-2-ol}zinc complex, 15. Prepared using the procedure described above for compound **1**, by refluxing a mixture of anhydrous $ZnCl_2$ (1.562 g, 11.46 mmol), 2-hydro-6-methyl-nicotinonitrile (1.000 g, 7.46 mmol), and *D*-phenylalaninol (4.008 g, 26.51 mmol) in 40 mL of dry chlorobenzene for 60 h. The product was obtained in 82% yield as colourless crystals after column chromatography (petroleum ether/CH_2Cl_2, 1/2). m.p. 120–124 °C, $[\alpha]^{25}_D$ =-109.0° (c=0.164, THF); δ_H (600 MHz, DMSO-d_6, 27°C) 12.36-12.41 (m, 3H), 9.78 (d, *J* = 8.0 Hz, 4H), 8.11 (d, *J* = 7.2 Hz, 2H), 7.13-7.22 (m, 17H), 6.23(d, *J* = 7.2 Hz, 2H), 4.90 (s, 3H), 4.08 (d, *J* = 5.2 Hz, 3H), 3.34-3.40 (m, 6H), 2.84-2.88 (m,

4H), 2.69-2.73 (m, 4H), 2.23 (s, 12H), δ_C (150 MHz, DMSO-d$_6$) 162.7, 162.4, 150.3, 143.5, 138.5, 128.9 (x2), 127.8 (x2), 125.7, 116.7, 105.4, 61.6, 51.8, 36.6, 18.3. ν_{max}(cm^{-1}) 3435, 3061, 2922, 1644, 1581, 1488, 1454, 1385, 1323, 1245, 1206, 1152, 1085, 1059, 1031, 986, 968, 786, 784, 704, 619, 510. Found C: 52.03, H: 4.38, N: 7.25%; for $C_{64}H_{60}N_8O_8Zn_4Cl_4$ requires C: 52.20, H: 4.11, N: 7.61%.

```
data_compound_1

_audit_creation_method            SHELXL-97
_chemical_name_systematic
;
 ?
;
_chemical_name_common             ?
_chemical_melting_point           ?
_chemical_formula_moiety          ?
_chemical_formula_sum
 'C14 H26 Cl2 N2 O Zn'
_chemical_formula_weight          374.64

loop_
 _atom_type_symbol
 _atom_type_description
 _atom_type_scat_dispersion_real
 _atom_type_scat_dispersion_imag
 _atom_type_scat_source
 'C'  'C'   0.0033   0.0016
 'International Tables Vol C Tables 4.2.6.8 and 6.1.1.4'
 'H'  'H'   0.0000   0.0000
 'International Tables Vol C Tables 4.2.6.8 and 6.1.1.4'
 'N'  'N'   0.0061   0.0033
 'International Tables Vol C Tables 4.2.6.8 and 6.1.1.4'
 'O'  'O'   0.0106   0.0060
 'International Tables Vol C Tables 4.2.6.8 and 6.1.1.4'
 'Cl'  'Cl'   0.1484   0.1585
 'International Tables Vol C Tables 4.2.6.8 and 6.1.1.4'
 'Zn'  'Zn'   0.2839   1.4301
 'International Tables Vol C Tables 4.2.6.8 and 6.1.1.4'

_symmetry_cell_setting            Orthorhombic
_symmetry_space_group_name_H-M    P2(1)2(1)2(1)

loop_
 _symmetry_equiv_pos_as_xyz
```

```
 'x, y, z'
 '-x+1/2, -y, z+1/2'
 '-x, y+1/2, -z+1/2'
 'x+1/2, -y+1/2, -z'

_cell_length_a                    7.3809(16)
_cell_length_b                    10.831(2)
_cell_length_c                    22.238(5)
_cell_angle_alpha                 90.00
_cell_angle_beta                  90.00
_cell_angle_gamma                 90.00
_cell_volume                      1777.8(7)
_cell_formula_units_Z             4
_cell_measurement_temperature     293(2)
_cell_measurement_reflns_used     1219
_cell_measurement_theta_min       5.251
_cell_measurement_theta_max       51.134

_exptl_crystal_description        prismatic
_exptl_crystal_colour             colorless
_exptl_crystal_size_max           0.176
_exptl_crystal_size_mid           0.145
_exptl_crystal_size_min           0.037
_exptl_crystal_density_meas       ?
_exptl_crystal_density_diffrn     1.400
_exptl_crystal_density_method     'not measured'
_exptl_crystal_F_000              784
_exptl_absorpt_coefficient_mu     1.680
_exptl_absorpt_correction_type    empirical
_exptl_absorpt_correction_T_min   0.0985
_exptl_absorpt_correction_T_max   1.0000
_exptl_absorpt_process_details    sadabs

_exptl_special_details
;
 ?
;

_diffrn_ambient_temperature       293(2)
_diffrn_radiation_wavelength      0.71073
_diffrn_radiation_type            MoK\a
_diffrn_radiation_source          'fine-focus sealed tube'
_diffrn_radiation_monochromator   graphite
_diffrn_measurement_device_type   'CCD area detector'
```

```
_diffrn_measurement_method        'phi and omega scans'
_diffrn_detector_area_resol_mean  ?
_diffrn_standards_number          ?
_diffrn_standards_interval_count  ?
_diffrn_standards_interval_time   ?
_diffrn_standards_decay_%         ?
_diffrn_reflns_number             10642
_diffrn_reflns_av_R_equivalents   0.0728
_diffrn_reflns_av_sigmaI/netI     0.0830
_diffrn_reflns_limit_h_min        -7
_diffrn_reflns_limit_h_max        9
_diffrn_reflns_limit_k_min        -13
_diffrn_reflns_limit_k_max        13
_diffrn_reflns_limit_l_min        -27
_diffrn_reflns_limit_l_max        26
_diffrn_reflns_theta_min          1.83
_diffrn_reflns_theta_max          26.00
_reflns_number_total              3492
_reflns_number_gt                 2462
_reflns_threshold_expression      >2sigma(I)

_computing_data_collection        'Bruker SMART'
_computing_cell_refinement        'Bruker SMART'
_computing_data_reduction         'Bruker SHELXTL'
_computing_structure_solution     'SHELXS-97 (Sheldrick, 1990)'
_computing_structure_refinement   'SHELXL-97 (Sheldrick, 1997)'
_computing_molecular_graphics     'Bruker SHELXTL'
_computing_publication_material   'Bruker SHELXTL'

_refine_special_details
;
 Refinement of F^2^ against ALL reflections.  The weighted R-factor wR and
 goodness of fit S are based on F^2^, conventional R-factors R are based
 on F, with F set to zero for negative F^2^. The threshold expression of
 F^2^ > 2sigma(F^2^) is used only for calculating R-factors(gt) etc. and is
 not relevant to the choice of reflections for refinement.  R-factors based
 on F^2^ are statistically about twice as large as those based on F, and R-
 factors based on ALL data will be even larger.
;

_refine_ls_structure_factor_coef  Fsqd
_refine_ls_matrix_type            full
_refine_ls_weighting_scheme       calc
_refine_ls_weighting_details
```

```
 'calc w=1/[\s^2^(Fo^2^)+(0.0320P)^2^+0.0000P] where P=(Fo^2^+2Fc^2^)/3'
_atom_sites_solution_primary      direct
_atom_sites_solution_secondary    difmap
_atom_sites_solution_hydrogens    geom
_refine_ls_hydrogen_treatment     constr
_refine_ls_extinction_method      none
_refine_ls_extinction_coef        ?
_refine_ls_abs_structure_details
 'Flack H D (1983), Acta Cryst. A39, 876-881'
_refine_ls_abs_structure_Flack    0.05(2)
_chemical_absolute_configuration  ad
_refine_ls_number_reflns          3492
_refine_ls_number_parameters      183
_refine_ls_number_restraints      0
_refine_ls_R_factor_all           0.0812
_refine_ls_R_factor_gt            0.0478
_refine_ls_wR_factor_ref          0.0915
_refine_ls_wR_factor_gt           0.0812
_refine_ls_goodness_of_fit_ref    0.987
_refine_ls_restrained_S_all       0.987
_refine_ls_shift/su_max           0.000
_refine_ls_shift/su_mean          0.000

loop_
 _atom_site_label
 _atom_site_type_symbol
 _atom_site_fract_x
 _atom_site_fract_y
 _atom_site_fract_z
 _atom_site_U_iso_or_equiv
 _atom_site_adp_type
 _atom_site_occupancy
 _atom_site_symmetry_multiplicity
 _atom_site_calc_flag
 _atom_site_refinement_flags
 _atom_site_disorder_assembly
 _atom_site_disorder_group
Zn1 Zn 0.55191(7) 0.06371(5) 0.10148(3) 0.03994(17) Uani 1 1 d . . .
Cl1 Cl 0.37243(17) -0.09630(12) 0.12141(7) 0.0564(4) Uani 1 1 d . . .
Cl2 Cl 0.41237(18) 0.24568(12) 0.09360(8) 0.0651(4) Uani 1 1 d . . .
N1 N 0.7012(5) 0.0199(3) 0.02905(17) 0.0367(10) Uani 1 1 d . . .
N2 N 0.7821(5) 0.0982(4) 0.15587(19) 0.0458(11) Uani 1 1 d . . .
O1 O 0.9583(5) -0.0258(3) -0.01849(15) 0.0500(9) Uani 1 1 d . . .
C1 C 0.6438(6) -0.0622(5) -0.0204(2) 0.0416(12) Uani 1 1 d . . .
```

```
H1 H 0.6025 -0.1410 -0.0036 0.050 Uiso 1 1 calc R . .
C2 C 0.8192(6) -0.0831(6) -0.0551(2) 0.0520(15) Uani 1 1 d . . .
H2A H 0.8428 -0.1706 -0.0601 0.062 Uiso 1 1 calc R . .
H2B H 0.8133 -0.0447 -0.0945 0.062 Uiso 1 1 calc R . .
C3 C 0.8723(6) 0.0345(5) 0.0254(2) 0.0389(13) Uani 1 1 d . . .
C4 C 0.9907(6) 0.1070(5) 0.0651(2) 0.0496(15) Uani 1 1 d . . .
H4A H 1.0536 0.1676 0.0408 0.060 Uiso 1 1 calc R . .
H4B H 1.0816 0.0519 0.0817 0.060 Uiso 1 1 calc R . .
C5 C 0.9006(7) 0.1740(5) 0.1170(2) 0.0486(15) Uani 1 1 d . . .
H5A H 0.9946 0.2102 0.1419 0.058 Uiso 1 1 calc R . .
H5B H 0.8291 0.2413 0.1006 0.058 Uiso 1 1 calc R . .
C6 C 0.8806(7) -0.0094(5) 0.1791(3) 0.0574(16) Uani 1 1 d . . .
H6A H 0.9897 0.0182 0.1993 0.069 Uiso 1 1 calc R . .
H6B H 0.9165 -0.0617 0.1457 0.069 Uiso 1 1 calc R . .
C7 C 0.7660(8) -0.0842(6) 0.2227(3) 0.0700(19) Uani 1 1 d . . .
H7A H 0.6634 -0.1191 0.2014 0.084 Uiso 1 1 calc R . .
H7B H 0.8377 -0.1520 0.2385 0.084 Uiso 1 1 calc R . .
C8 C 0.6979(10) -0.0060(7) 0.2745(3) 0.082(2) Uani 1 1 d . . .
H8A H 0.6130 -0.0535 0.2985 0.099 Uiso 1 1 calc R . .
H8B H 0.7988 0.0171 0.3001 0.099 Uiso 1 1 calc R . .
C9 C 0.6055(9) 0.1091(7) 0.2510(3) 0.076(2) Uani 1 1 d . . .
H9A H 0.5747 0.1626 0.2845 0.092 Uiso 1 1 calc R . .
H9B H 0.4941 0.0863 0.2307 0.092 Uiso 1 1 calc R . .
C10 C 0.7280(8) 0.1784(6) 0.2075(3) 0.0619(17) Uani 1 1 d . . .
H10A H 0.6649 0.2506 0.1924 0.074 Uiso 1 1 calc R . .
H10B H 0.8356 0.2062 0.2285 0.074 Uiso 1 1 calc R . .
C11 C 0.4937(6) -0.0069(4) -0.0582(2) 0.0453(14) Uani 1 1 d . . .
H11A H 0.5397 0.0661 -0.0783 0.054 Uiso 1 1 calc R . .
H11B H 0.3961 0.0190 -0.0318 0.054 Uiso 1 1 calc R . .
C12 C 0.4178(6) -0.0949(4) -0.1051(2) 0.0462(13) Uani 1 1 d . . .
H12 H 0.5195 -0.1275 -0.1285 0.055 Uiso 1 1 calc R . .
C13 C 0.2915(9) -0.0266(6) -0.1480(3) 0.076(2) Uani 1 1 d . . .
H13A H 0.1932 0.0091 -0.1257 0.113 Uiso 1 1 calc R . .
H13B H 0.2443 -0.0835 -0.1772 0.113 Uiso 1 1 calc R . .
H13C H 0.3574 0.0376 -0.1682 0.113 Uiso 1 1 calc R . .
C14 C 0.3234(8) -0.2020(5) -0.0753(3) 0.0722(19) Uani 1 1 d . . .
H14A H 0.4116 -0.2538 -0.0560 0.108 Uiso 1 1 calc R . .
H14B H 0.2592 -0.2490 -0.1050 0.108 Uiso 1 1 calc R . .
H14C H 0.2395 -0.1716 -0.0458 0.108 Uiso 1 1 calc R . .

loop_
 _atom_site_aniso_label
 _atom_site_aniso_U_11
 _atom_site_aniso_U_22
```

```
 _atom_site_aniso_U_33
 _atom_site_aniso_U_23
 _atom_site_aniso_U_13
 _atom_site_aniso_U_12
Zn1 0.0284(3) 0.0452(3) 0.0462(3) -0.0046(3) 0.0009(3) -0.0025(3)
Cl1 0.0451(8) 0.0478(9) 0.0763(11) 0.0009(7) 0.0035(7) -0.0092(6)
Cl2 0.0458(8) 0.0490(8) 0.1005(12) 0.0001(9) -0.0080(9) 0.0054(6)
N1 0.029(2) 0.049(3) 0.033(2) -0.004(2) -0.0026(18) -0.0046(19)
N2 0.035(2) 0.052(3) 0.051(3) -0.005(2) 0.000(2) -0.004(2)
O1 0.0394(19) 0.061(2) 0.049(2) -0.0071(18) 0.0016(19) 0.010(2)
C1 0.037(3) 0.046(3) 0.042(3) -0.003(3) 0.001(2) 0.000(3)
C2 0.045(3) 0.064(4) 0.047(3) -0.006(3) 0.001(3) 0.009(3)
C3 0.037(3) 0.043(3) 0.037(3) 0.013(3) -0.001(2) -0.005(3)
C4 0.026(3) 0.072(4) 0.050(3) -0.001(3) 0.003(2) -0.008(2)
C5 0.036(3) 0.055(3) 0.055(4) -0.003(3) -0.002(2) -0.014(3)
C6 0.046(3) 0.062(4) 0.064(4) 0.003(3) -0.016(3) 0.007(3)
C7 0.066(4) 0.083(5) 0.061(4) 0.016(4) -0.019(3) -0.004(4)
C8 0.084(5) 0.117(6) 0.045(4) 0.003(4) 0.003(4) -0.029(5)
C9 0.075(5) 0.106(6) 0.049(4) -0.021(4) 0.012(3) -0.021(4)
C10 0.052(4) 0.076(4) 0.057(4) -0.025(4) 0.006(3) -0.011(3)
C11 0.043(3) 0.040(3) 0.053(4) 0.005(3) -0.007(2) -0.001(2)
C12 0.035(3) 0.050(3) 0.054(3) -0.005(3) -0.006(3) 0.000(2)
C13 0.073(4) 0.083(5) 0.071(5) -0.008(4) -0.024(4) 0.004(4)
C14 0.066(4) 0.064(4) 0.086(5) -0.010(4) -0.007(4) -0.024(4)

_geom_special_details
;
 All esds (except the esd in the dihedral angle between two l.s. planes)
 are estimated using the full covariance matrix.  The cell esds are taken
 into account individually in the estimation of esds in distances, angles
 and torsion angles; correlations between esds in cell parameters are only
 used when they are defined by crystal symmetry.  An approximate (isotropic)
 treatment of cell esds is used for estimating esds involving l.s. planes.
;

loop_
 _geom_bond_atom_site_label_1
 _geom_bond_atom_site_label_2
 _geom_bond_distance
 _geom_bond_site_symmetry_2
 _geom_bond_publ_flag
Zn1 N1 2.008(4) . ?
Zn1 N2 2.119(4) . ?
Zn1 Cl1 2.2260(14) . ?
```

Zn1 Cl2 2.2307(15) . ?
N1 C3 1.276(5) . ?
N1 C1 1.477(6) . ?
N2 C6 1.468(6) . ?
N2 C5 1.479(6) . ?
N2 C10 1.494(6) . ?
O1 C3 1.335(5) . ?
O1 C2 1.450(6) . ?
C1 C11 1.514(6) . ?
C1 C2 1.523(6) . ?
C1 H1 0.9800 . ?
C2 H2A 0.9700 . ?
C2 H2B 0.9700 . ?
C3 C4 1.470(6) . ?
C4 C5 1.516(6) . ?
C4 H4A 0.9700 . ?
C4 H4B 0.9700 . ?
C5 H5A 0.9700 . ?
C5 H5B 0.9700 . ?
C6 C7 1.521(7) . ?
C6 H6A 0.9700 . ?
C6 H6B 0.9700 . ?
C7 C8 1.515(8) . ?
C7 H7A 0.9700 . ?
C7 H7B 0.9700 . ?
C8 C9 1.514(8) . ?
C8 H8A 0.9700 . ?
C8 H8B 0.9700 . ?
C9 C10 1.522(8) . ?
C9 H9A 0.9700 . ?
C9 H9B 0.9700 . ?
C10 H10A 0.9700 . ?
C10 H10B 0.9700 . ?
C11 C12 1.519(6) . ?
C11 H11A 0.9700 . ?
C11 H11B 0.9700 . ?
C12 C14 1.507(7) . ?
C12 C13 1.526(7) . ?
C12 H12 0.9800 . ?
C13 H13A 0.9600 . ?
C13 H13B 0.9600 . ?
C13 H13C 0.9600 . ?
C14 H14A 0.9600 . ?
C14 H14B 0.9600 . ?

C14 H14C 0.9600 . ?

loop_
 _geom_angle_atom_site_label_1
 _geom_angle_atom_site_label_2
 _geom_angle_atom_site_label_3
 _geom_angle
 _geom_angle_site_symmetry_1
 _geom_angle_site_symmetry_3
 _geom_angle_publ_flag
N1 Zn1 N2 93.42(16) . . ?
N1 Zn1 Cl1 107.60(12) . . ?
N2 Zn1 Cl1 120.06(12) . . ?
N1 Zn1 Cl2 113.51(12) . . ?
N2 Zn1 Cl2 105.02(12) . . ?
Cl1 Zn1 Cl2 115.39(6) . . ?
C3 N1 C1 108.1(4) . . ?
C3 N1 Zn1 124.4(3) . . ?
C1 N1 Zn1 125.6(3) . . ?
C6 N2 C5 110.7(4) . . ?
C6 N2 C10 108.9(4) . . ?
C5 N2 C10 106.5(4) . . ?
C6 N2 Zn1 117.2(3) . . ?
C5 N2 Zn1 103.8(3) . . ?
C10 N2 Zn1 109.1(3) . . ?
C3 O1 C2 106.5(4) . . ?
N1 C1 C11 112.6(4) . . ?
N1 C1 C2 102.9(4) . . ?
C11 C1 C2 113.6(4) . . ?
N1 C1 H1 109.2 . . ?
C11 C1 H1 109.2 . . ?
C2 C1 H1 109.2 . . ?
O1 C2 C1 104.7(4) . . ?
O1 C2 H2A 110.8 . . ?
C1 C2 H2A 110.8 . . ?
O1 C2 H2B 110.8 . . ?
C1 C2 H2B 110.8 . . ?
H2A C2 H2B 108.9 . . ?
N1 C3 O1 117.2(4) . . ?
N1 C3 C4 128.0(5) . . ?
O1 C3 C4 114.7(4) . . ?
C3 C4 C5 116.9(4) . . ?
C3 C4 H4A 108.1 . . ?
C5 C4 H4A 108.1 . . ?

C3 C4 H4B 108.1 . . ?
C5 C4 H4B 108.1 . . ?
H4A C4 H4B 107.3 . . ?
N2 C5 C4 116.0(4) . . ?
N2 C5 H5A 108.3 . . ?
C4 C5 H5A 108.3 . . ?
N2 C5 H5B 108.3 . . ?
C4 C5 H5B 108.3 . . ?
H5A C5 H5B 107.4 . . ?
N2 C6 C7 111.8(5) . . ?
N2 C6 H6A 109.2 . . ?
C7 C6 H6A 109.2 . . ?
N2 C6 H6B 109.2 . . ?
C7 C6 H6B 109.2 . . ?
H6A C6 H6B 107.9 . . ?
C8 C7 C6 111.8(6) . . ?
C8 C7 H7A 109.3 . . ?
C6 C7 H7A 109.3 . . ?
C8 C7 H7B 109.3 . . ?
C6 C7 H7B 109.3 . . ?
H7A C7 H7B 107.9 . . ?
C9 C8 C7 110.3(5) . . ?
C9 C8 H8A 109.6 . . ?
C7 C8 H8A 109.6 . . ?
C9 C8 H8B 109.6 . . ?
C7 C8 H8B 109.6 . . ?
H8A C8 H8B 108.1 . . ?
C8 C9 C10 111.0(6) . . ?
C8 C9 H9A 109.4 . . ?
C10 C9 H9A 109.4 . . ?
C8 C9 H9B 109.4 . . ?
C10 C9 H9B 109.4 . . ?
H9A C9 H9B 108.0 . . ?
N2 C10 C9 111.2(5) . . ?
N2 C10 H10A 109.4 . . ?
C9 C10 H10A 109.4 . . ?
N2 C10 H10B 109.4 . . ?
C9 C10 H10B 109.4 . . ?
H10A C10 H10B 108.0 . . ?
C1 C11 C12 113.7(4) . . ?
C1 C11 H11A 108.8 . . ?
C12 C11 H11A 108.8 . . ?
C1 C11 H11B 108.8 . . ?
C12 C11 H11B 108.8 . . ?

```
H11A C11 H11B 107.7 . . ?
C14 C12 C11 110.6(4) . . ?
C14 C12 C13 111.5(5) . . ?
C11 C12 C13 110.5(4) . . ?
C14 C12 H12 108.0 . . ?
C11 C12 H12 108.0 . . ?
C13 C12 H12 108.0 . . ?
C12 C13 H13A 109.5 . . ?
C12 C13 H13B 109.5 . . ?
H13A C13 H13B 109.5 . . ?
C12 C13 H13C 109.5 . . ?
H13A C13 H13C 109.5 . . ?
H13B C13 H13C 109.5 . . ?
C12 C14 H14A 109.5 . . ?
C12 C14 H14B 109.5 . . ?
H14A C14 H14B 109.5 . . ?
C12 C14 H14C 109.5 . . ?
H14A C14 H14C 109.5 . . ?
H14B C14 H14C 109.5 . . ?

loop_
 _geom_torsion_atom_site_label_1
 _geom_torsion_atom_site_label_2
 _geom_torsion_atom_site_label_3
 _geom_torsion_atom_site_label_4
 _geom_torsion
 _geom_torsion_site_symmetry_1
 _geom_torsion_site_symmetry_2
 _geom_torsion_site_symmetry_3
 _geom_torsion_site_symmetry_4
 _geom_torsion_publ_flag
N2 Zn1 N1 C3 -9.0(4) . . . . ?
Cl1 Zn1 N1 C3 -132.1(4) . . . . ?
Cl2 Zn1 N1 C3 99.0(4) . . . . ?
N2 Zn1 N1 C1 153.6(4) . . . . ?
Cl1 Zn1 N1 C1 30.5(4) . . . . ?
Cl2 Zn1 N1 C1 -98.4(4) . . . . ?
N1 Zn1 N2 C6 -76.7(4) . . . . ?
Cl1 Zn1 N2 C6 35.9(4) . . . . ?
Cl2 Zn1 N2 C6 167.9(4) . . . . ?
N1 Zn1 N2 C5 45.7(3) . . . . ?
Cl1 Zn1 N2 C5 158.3(3) . . . . ?
Cl2 Zn1 N2 C5 -69.7(3) . . . . ?
N1 Zn1 N2 C10 159.0(4) . . . . ?
```

Cl1 Zn1 N2 C10 -88.4(3) . . . . ?
Cl2 Zn1 N2 C10 43.6(4) . . . . ?
C3 N1 C1 C11 -126.5(4) . . . . ?
Zn1 N1 C1 C11 68.6(5) . . . . ?
C3 N1 C1 C2 -3.8(5) . . . . ?
Zn1 N1 C1 C2 -168.8(3) . . . . ?
C3 O1 C2 C1 -8.0(6) . . . . ?
N1 C1 C2 O1 7.1(5) . . . . ?
C11 C1 C2 O1 129.0(4) . . . . ?
C1 N1 C3 O1 -1.4(6) . . . . ?
Zn1 N1 C3 O1 163.8(3) . . . . ?
C1 N1 C3 C4 -179.2(5) . . . . ?
Zn1 N1 C3 C4 -14.0(8) . . . . ?
C2 O1 C3 N1 6.3(6) . . . . ?
C2 O1 C3 C4 -175.7(4) . . . . ?
N1 C3 C4 C5 -0.4(8) . . . . ?
O1 C3 C4 C5 -178.2(4) . . . . ?
C6 N2 C5 C4 54.7(6) . . . . ?
C10 N2 C5 C4 173.0(4) . . . . ?
Zn1 N2 C5 C4 -71.9(4) . . . . ?
C3 C4 C5 N2 51.9(6) . . . . ?
C5 N2 C6 C7 176.0(5) . . . . ?
C10 N2 C6 C7 59.2(6) . . . . ?
Zn1 N2 C6 C7 -65.2(6) . . . . ?
N2 C6 C7 C8 -56.2(7) . . . . ?
C6 C7 C8 C9 51.9(7) . . . . ?
C7 C8 C9 C10 -52.8(7) . . . . ?
C6 N2 C10 C9 -60.2(6) . . . . ?
C5 N2 C10 C9 -179.7(5) . . . . ?
Zn1 N2 C10 C9 68.9(5) . . . . ?
C8 C9 C10 N2 57.8(7) . . . . ?
N1 C1 C11 C12 -173.6(4) . . . . ?
C2 C1 C11 C12 69.9(6) . . . . ?
C1 C11 C12 C14 65.8(6) . . . . ?
C1 C11 C12 C13 -170.3(4) . . . . ?

_diffrn_measured_fraction_theta_max   1.000
_diffrn_reflns_theta_full   26.00
_diffrn_measured_fraction_theta_full   1.000
_refine_diff_density_max   0.299
_refine_diff_density_min   -0.317
_refine_diff_density_rms   0.065

data_compound_2

```
_audit_creation_method            SHELXL-97
_chemical_name_systematic
;
 ?
;
_chemical_name_common             ?
_chemical_melting_point           ?
_chemical_formula_moiety          ?
_chemical_formula_sum
 'C14 H26 Cl2 N2 O2 Zn'
_chemical_formula_weight          390.64

loop_
 _atom_type_symbol
 _atom_type_description
 _atom_type_scat_dispersion_real
 _atom_type_scat_dispersion_imag
 _atom_type_scat_source
 'C'  'C'   0.0033   0.0016
 'International Tables Vol C Tables 4.2.6.8 and 6.1.1.4'
 'H'  'H'   0.0000   0.0000
 'International Tables Vol C Tables 4.2.6.8 and 6.1.1.4'
 'N'  'N'   0.0061   0.0033
 'International Tables Vol C Tables 4.2.6.8 and 6.1.1.4'
 'Cl'  'Cl'   0.1484   0.1585
 'International Tables Vol C Tables 4.2.6.8 and 6.1.1.4'
 'Zn'  'Zn'   0.2839   1.4301
 'International Tables Vol C Tables 4.2.6.8 and 6.1.1.4'
 'O'  'O'   0.0106   0.0060
 'International Tables Vol C Tables 4.2.6.8 and 6.1.1.4'

_symmetry_cell_setting            Monoclinic
_symmetry_space_group_name_H-M    P2(1)

loop_
 _symmetry_equiv_pos_as_xyz
 'x, y, z'
 '-x, y+1/2, -z'

_cell_length_a                    6.494(2)
_cell_length_b                    16.648(5)
_cell_length_c                    9.129(3)
_cell_angle_alpha                 90.00
```

```
_cell_angle_beta                  109.082(5)
_cell_angle_gamma                 90.00
_cell_volume                      932.6(5)
_cell_formula_units_Z             2
_cell_measurement_temperature     140(2)
_cell_measurement_reflns_used     4218
_cell_measurement_theta_min       2.36
_cell_measurement_theta_max       30.29

_exptl_crystal_description        block
_exptl_crystal_colour             colourless
_exptl_crystal_size_max           0.22
_exptl_crystal_size_mid           0.16
_exptl_crystal_size_min           0.10
_exptl_crystal_density_meas       ?
_exptl_crystal_density_diffrn     1.391
_exptl_crystal_density_method     'not measured'
_exptl_crystal_F_000              408
_exptl_absorpt_coefficient_mu     1.608
_exptl_absorpt_correction_type    multi-scan
_exptl_absorpt_correction_T_min   0.7187
_exptl_absorpt_correction_T_max   0.8558
_exptl_absorpt_process_details    sadabs

_exptl_special_details
;
 ?
;

_diffrn_ambient_temperature       140(2)
_diffrn_radiation_wavelength      0.71073
_diffrn_radiation_type            MoK\a
_diffrn_radiation_source          'fine-focus sealed tube'
_diffrn_radiation_monochromator   graphite
_diffrn_measurement_device_type   'Bruker APEX-II CCD'
_diffrn_measurement_method        '\f and \w scans'
_diffrn_detector_area_resol_mean  ?
_diffrn_reflns_number             8700
_diffrn_reflns_av_R_equivalents   0.0456
_diffrn_reflns_av_sigmaI/netI     0.0804
_diffrn_reflns_limit_h_min        -8
_diffrn_reflns_limit_h_max        9
_diffrn_reflns_limit_k_min        -23
_diffrn_reflns_limit_k_max        20
```

```
_diffrn_reflns_limit_l_min        -13
_diffrn_reflns_limit_l_max        13
_diffrn_reflns_theta_min          2.36
_diffrn_reflns_theta_max          30.59
_reflns_number_total              4982
_reflns_number_gt                 4283
_reflns_threshold_expression      >2sigma(I)

_computing_data_collection        'Bruker APEX2'
_computing_cell_refinement        'Bruker SAINT'
_computing_data_reduction         'Bruker SAINT'
_computing_structure_solution     'SHELXS-97 (Sheldrick, 2008)'
_computing_structure_refinement   'SHELXL-97 (Sheldrick, 2008)'
_computing_molecular_graphics     'Bruker SHELXTL'
_computing_publication_material   'Bruker SHELXTL'

_refine_special_details
;
 Refinement of F^2^ against ALL reflections.  The weighted R-factor wR and
 goodness of fit S are based on F^2^, conventional R-factors R are based
 on F, with F set to zero for negative F^2^. The threshold expression of
 F^2^ > 2sigma(F^2^) is used only for calculating R-factors(gt) etc. and is
 not relevant to the choice of reflections for refinement.  R-factors based
 on F^2^ are statistically about twice as large as those based on F, and R-
 factors based on ALL data will be even larger.
;

_refine_ls_structure_factor_coef  Fsqd
_refine_ls_matrix_type            full
_refine_ls_weighting_scheme       calc
_refine_ls_weighting_details
 'calc w=1/[\s^2^(Fo^2^)+(0.0705P)^2^+0.3114P] where P=(Fo^2^+2Fc^2^)/3'
_atom_sites_solution_primary      direct
_atom_sites_solution_secondary    difmap
_atom_sites_solution_hydrogens    geom
_refine_ls_hydrogen_treatment     constr
_refine_ls_extinction_method      none
_refine_ls_extinction_coef        ?
_refine_ls_abs_structure_details
 'Flack H D (1983), Acta Cryst. A39, 876-881'
_refine_ls_abs_structure_Flack    0.059(16)
_chemical_absolute_configuration  ad
_refine_ls_number_reflns          4982
_refine_ls_number_parameters      196
```

```
_refine_ls_number_restraints      1
_refine_ls_R_factor_all           0.0551
_refine_ls_R_factor_gt            0.0455
_refine_ls_wR_factor_ref          0.1329
_refine_ls_wR_factor_gt           0.1212
_refine_ls_goodness_of_fit_ref    0.992
_refine_ls_restrained_S_all       0.992
_refine_ls_shift/su_max           0.027
_refine_ls_shift/su_mean          0.001

loop_
 _atom_site_label
 _atom_site_type_symbol
 _atom_site_fract_x
 _atom_site_fract_y
 _atom_site_fract_z
 _atom_site_U_iso_or_equiv
 _atom_site_adp_type
 _atom_site_occupancy
 _atom_site_symmetry_multiplicity
 _atom_site_calc_flag
 _atom_site_refinement_flags
 _atom_site_disorder_assembly
 _atom_site_disorder_group
Zn1 Zn 0.19500(6) 0.24677(3) 0.22209(4) 0.02056(12) Uani 1 1 d . . .
Cl1 Cl 0.52949(16) 0.19528(7) 0.33251(12) 0.0289(2) Uani 1 1 d . . .
Cl2 Cl 0.17201(18) 0.32396(8) 0.01700(12) 0.0332(3) Uani 1 1 d . . .
N1 N -0.0258(6) 0.1550(2) 0.1724(4) 0.0241(7) Uani 1 1 d . . .
N2 N 0.0979(5) 0.3087(2) 0.3800(4) 0.0208(7) Uani 1 1 d . . .
O1 O -0.1935(5) 0.0451(2) 0.2193(4) 0.0347(8) Uani 1 1 d . . .
O2 O -0.1159(4) 0.3844(2) 0.4728(4) 0.0258(6) Uani 1 1 d . . .
C1 C 0.1018(9) 0.0932(3) 0.4349(6) 0.0334(10) Uani 1 1 d . . .
H1A H 0.2325 0.1265 0.4546 0.050 Uiso 1 1 calc R . .
H1B H 0.1448 0.0369 0.4581 0.050 Uiso 1 1 calc R . .
H1C H 0.0185 0.1111 0.5010 0.050 Uiso 1 1 calc R . .
C2 C -0.0360(7) 0.1007(3) 0.2682(5) 0.0270(9) Uani 1 1 d . . .
C3 C -0.3245(8) 0.0666(4) 0.0623(6) 0.0478(15) Uani 1 1 d . . .
H3A H -0.4757 0.0797 0.0565 0.057 Uiso 1 1 calc R . .
H3B H -0.3280 0.0222 -0.0108 0.057 Uiso 1 1 calc R . .
C4 C -0.2082(8) 0.1410(3) 0.0257(5) 0.0342(10) Uani 1 1 d . . .
H4 H -0.3102 0.1877 0.0085 0.041 Uiso 1 1 calc R . .
C5 C -0.1367(13) 0.1325(4) -0.1154(6) 0.0535(17) Uani 1 1 d . . .
H5 H -0.0616 0.1833 -0.1275 0.064 Uiso 1 1 calc R . .
C6 C 0.0258(10) 0.0624(5) -0.0964(8) 0.060(2) Uani 1 1 d . . .
```

```
H6A H 0.1036 0.0535 0.0140 0.090 Uiso 1 1 calc R . .
H6B H 0.1306 0.0757 -0.1493 0.090 Uiso 1 1 calc R . .
H6C H -0.0537 0.0136 -0.1419 0.090 Uiso 1 1 calc R . .
C7 C -0.3408(15) 0.1222(5) -0.2607(7) 0.078(3) Uani 1 1 d . . .
H7A H -0.4076 0.0697 -0.2570 0.117 Uiso 1 1 calc R . .
H7B H -0.2983 0.1254 -0.3541 0.117 Uiso 1 1 calc R . .
H7C H -0.4459 0.1648 -0.2630 0.117 Uiso 1 1 calc R . .
C8 C -0.2382(6) 0.3678(3) 0.1987(5) 0.0315(10) Uani 1 1 d . . .
H8A H -0.1747 0.3570 0.1169 0.047 Uiso 1 1 calc R . .
H8B H -0.2824 0.4242 0.1940 0.047 Uiso 1 1 calc R . .
H8C H -0.3658 0.3333 0.1837 0.047 Uiso 1 1 calc R . .
C9 C -0.0744(6) 0.3508(3) 0.3519(5) 0.0217(8) Uani 1 1 d . . .
C10 C 0.0483(7) 0.3525(3) 0.6109(5) 0.0265(9) Uani 1 1 d . . .
H10A H -0.0157 0.3117 0.6621 0.032 Uiso 1 1 calc R . .
H10B H 0.1134 0.3959 0.6858 0.032 Uiso 1 1 calc R . .
C11 C 0.2179(6) 0.3149(3) 0.5494(4) 0.0204(7) Uani 1 1 d . . .
H11 H 0.2541 0.2598 0.5937 0.025 Uiso 1 1 calc R . .
C12 C 0.4311(6) 0.3636(3) 0.5794(5) 0.0224(8) Uani 1 1 d . . .
H12 H 0.5248 0.3340 0.5295 0.027 Uiso 1 1 calc R . .
C13 C 0.3930(7) 0.4465(3) 0.5108(6) 0.0323(10) Uani 1 1 d . . .
H13A H 0.3376 0.4813 0.5761 0.048 Uiso 1 1 calc R . .
H13B H 0.2861 0.4440 0.4064 0.048 Uiso 1 1 calc R . .
H13C H 0.5304 0.4683 0.5053 0.048 Uiso 1 1 calc R . .
C14 C 0.5539(8) 0.3670(4) 0.7538(6) 0.0358(11) Uani 1 1 d . . .
H14A H 0.7019 0.3871 0.7715 0.054 Uiso 1 1 calc R . .
H14B H 0.5611 0.3130 0.7981 0.054 Uiso 1 1 calc R . .
H14C H 0.4774 0.4030 0.8035 0.054 Uiso 1 1 calc R . .

loop_
 _atom_site_aniso_label
 _atom_site_aniso_U_11
 _atom_site_aniso_U_22
 _atom_site_aniso_U_33
 _atom_site_aniso_U_23
 _atom_site_aniso_U_13
 _atom_site_aniso_U_12
Zn1 0.01856(18) 0.0310(2) 0.01348(17) 0.0032(2) 0.00712(13) 0.0018(2)
Cl1 0.0224(4) 0.0394(6) 0.0277(5) 0.0073(4) 0.0121(4) 0.0090(4)
Cl2 0.0269(5) 0.0511(7) 0.0207(4) 0.0140(5) 0.0068(4) -0.0042(5)
N1 0.0280(17) 0.032(2) 0.0150(14) -0.0019(13) 0.0099(13) 0.0020(14)
N2 0.0177(14) 0.0261(18) 0.0188(14) 0.0010(13) 0.0061(11) 0.0003(12)
O1 0.0254(15) 0.042(2) 0.0349(17) -0.0079(16) 0.0076(13) -0.0119(14)
O2 0.0162(12) 0.0333(17) 0.0285(15) -0.0017(13) 0.0081(11) 0.0025(12)
C1 0.045(3) 0.030(2) 0.028(2) 0.0065(19) 0.0161(19) -0.004(2)
```

```
C2 0.0256(19) 0.030(2) 0.029(2) -0.0029(18) 0.0139(16) -0.0057(16)
C3 0.030(2) 0.078(5) 0.034(3) -0.018(3) 0.008(2) -0.013(3)
C4 0.040(2) 0.033(2) 0.0213(19) -0.0050(18) -0.0005(17) 0.013(2)
C5 0.098(5) 0.041(3) 0.018(2) -0.005(2) 0.015(3) -0.016(3)
C6 0.039(3) 0.097(6) 0.050(3) -0.032(4) 0.021(3) -0.001(3)
C7 0.125(7) 0.047(4) 0.027(3) -0.009(3) -0.023(4) 0.020(4)
C8 0.0117(15) 0.051(3) 0.025(2) 0.004(2) -0.0032(14) 0.0017(17)
C9 0.0112(15) 0.030(2) 0.0228(18) 0.0021(16) 0.0039(13) -0.0005(14)
C10 0.0219(18) 0.041(3) 0.0181(17) 0.0007(17) 0.0089(14) 0.0002(16)
C11 0.0221(17) 0.024(2) 0.0168(16) 0.0044(14) 0.0082(13) 0.0028(14)
C12 0.0166(16) 0.031(2) 0.0179(17) -0.0060(16) 0.0034(13) 0.0020(15)
C13 0.026(2) 0.035(3) 0.039(2) 0.004(2) 0.0145(18) -0.0054(18)
C14 0.027(2) 0.041(3) 0.030(2) -0.010(2) -0.0031(18) 0.006(2)

_geom_special_details
;
 All esds (except the esd in the dihedral angle between two l.s. planes)
 are estimated using the full covariance matrix.  The cell esds are taken
 into account individually in the estimation of esds in distances, angles
 and torsion angles; correlations between esds in cell parameters are only
 used when they are defined by crystal symmetry.  An approximate (isotropic)
 treatment of cell esds is used for estimating esds involving l.s. planes.
;

loop_
 _geom_bond_atom_site_label_1
 _geom_bond_atom_site_label_2
 _geom_bond_distance
 _geom_bond_site_symmetry_2
 _geom_bond_publ_flag
Zn1 N2 2.033(3) . ?
Zn1 N1 2.043(4) . ?
Zn1 Cl2 2.2352(12) . ?
Zn1 Cl1 2.2443(12) . ?
N1 C2 1.274(6) . ?
N1 C4 1.487(5) . ?
N2 C9 1.274(5) . ?
N2 C11 1.490(5) . ?
O1 C2 1.343(5) . ?
O1 C3 1.453(7) . ?
O2 C9 1.341(5) . ?
O2 C10 1.459(5) . ?
C1 C2 1.497(7) . ?
C1 H1A 0.9800 . ?
```

C1 H1B 0.9800 . ?
C1 H1C 0.9800 . ?
C3 C4 1.543(9) . ?
C3 H3A 0.9900 . ?
C3 H3B 0.9900 . ?
C4 C5 1.512(8) . ?
C4 H4 1.0000 . ?
C5 C6 1.544(11) . ?
C5 C7 1.548(8) . ?
C5 H5 1.0000 . ?
C6 H6A 0.9800 . ?
C6 H6B 0.9800 . ?
C6 H6C 0.9800 . ?
C7 H7A 0.9800 . ?
C7 H7B 0.9800 . ?
C7 H7C 0.9800 . ?
C8 C9 1.481(6) . ?
C8 H8A 0.9800 . ?
C8 H8B 0.9800 . ?
C8 H8C 0.9800 . ?
C10 C11 1.525(6) . ?
C10 H10A 0.9900 . ?
C10 H10B 0.9900 . ?
C11 C12 1.549(6) . ?
C11 H11 1.0000 . ?
C12 C13 1.503(7) . ?
C12 C14 1.531(6) . ?
C12 H12 1.0000 . ?
C13 H13A 0.9800 . ?
C13 H13B 0.9800 . ?
C13 H13C 0.9800 . ?
C14 H14A 0.9800 . ?
C14 H14B 0.9800 . ?
C14 H14C 0.9800 . ?

loop_
 _geom_angle_atom_site_label_1
 _geom_angle_atom_site_label_2
 _geom_angle_atom_site_label_3
 _geom_angle
 _geom_angle_site_symmetry_1
 _geom_angle_site_symmetry_3
 _geom_angle_publ_flag
N2 Zn1 N1 100.32(14) . . ?

N2 Zn1 Cl2 110.56(11) . . ?
N1 Zn1 Cl2 113.22(10) . . ?
N2 Zn1 Cl1 110.74(10) . . ?
N1 Zn1 Cl1 108.67(11) . . ?
Cl2 Zn1 Cl1 112.68(4) . . ?
C2 N1 C4 107.6(4) . . ?
C2 N1 Zn1 124.6(3) . . ?
C4 N1 Zn1 127.7(3) . . ?
C9 N2 C11 107.3(3) . . ?
C9 N2 Zn1 126.3(3) . . ?
C11 N2 Zn1 126.4(3) . . ?
C2 O1 C3 107.1(4) . . ?
C9 O2 C10 105.8(3) . . ?
C2 C1 H1A 109.5 . . ?
C2 C1 H1B 109.5 . . ?
H1A C1 H1B 109.5 . . ?
C2 C1 H1C 109.5 . . ?
H1A C1 H1C 109.5 . . ?
H1B C1 H1C 109.5 . . ?
N1 C2 O1 117.8(4) . . ?
N1 C2 C1 127.5(4) . . ?
O1 C2 C1 114.7(4) . . ?
O1 C3 C4 103.9(4) . . ?
O1 C3 H3A 111.0 . . ?
C4 C3 H3A 111.0 . . ?
O1 C3 H3B 111.0 . . ?
C4 C3 H3B 111.0 . . ?
H3A C3 H3B 109.0 . . ?
N1 C4 C5 113.9(5) . . ?
N1 C4 C3 103.5(4) . . ?
C5 C4 C3 114.7(4) . . ?
N1 C4 H4 108.1 . . ?
C5 C4 H4 108.1 . . ?
C3 C4 H4 108.1 . . ?
C4 C5 C6 111.3(5) . . ?
C4 C5 C7 109.0(6) . . ?
C6 C5 C7 112.0(5) . . ?
C4 C5 H5 108.1 . . ?
C6 C5 H5 108.1 . . ?
C7 C5 H5 108.1 . . ?
C5 C6 H6A 109.5 . . ?
C5 C6 H6B 109.5 . . ?
H6A C6 H6B 109.5 . . ?
C5 C6 H6C 109.5 . . ?

H6A C6 H6C 109.5 . . ?
H6B C6 H6C 109.5 . . ?
C5 C7 H7A 109.5 . . ?
C5 C7 H7B 109.5 . . ?
H7A C7 H7B 109.5 . . ?
C5 C7 H7C 109.5 . . ?
H7A C7 H7C 109.5 . . ?
H7B C7 H7C 109.5 . . ?
C9 C8 H8A 109.5 . . ?
C9 C8 H8B 109.5 . . ?
H8A C8 H8B 109.5 . . ?
C9 C8 H8C 109.5 . . ?
H8A C8 H8C 109.5 . . ?
H8B C8 H8C 109.5 . . ?
N2 C9 O2 117.6(3) . . ?
N2 C9 C8 127.4(4) . . ?
O2 C9 C8 115.0(4) . . ?
O2 C10 C11 104.1(3) . . ?
O2 C10 H10A 110.9 . . ?
C11 C10 H10A 110.9 . . ?
O2 C10 H10B 110.9 . . ?
C11 C10 H10B 110.9 . . ?
H10A C10 H10B 109.0 . . ?
N2 C11 C10 102.4(3) . . ?
N2 C11 C12 110.7(3) . . ?
C10 C11 C12 115.6(4) . . ?
N2 C11 H11 109.3 . . ?
C10 C11 H11 109.3 . . ?
C12 C11 H11 109.3 . . ?
C13 C12 C14 111.0(4) . . ?
C13 C12 C11 113.0(3) . . ?
C14 C12 C11 109.6(4) . . ?
C13 C12 H12 107.7 . . ?
C14 C12 H12 107.7 . . ?
C11 C12 H12 107.7 . . ?
C12 C13 H13A 109.5 . . ?
C12 C13 H13B 109.5 . . ?
H13A C13 H13B 109.5 . . ?
C12 C13 H13C 109.5 . . ?
H13A C13 H13C 109.5 . . ?
H13B C13 H13C 109.5 . . ?
C12 C14 H14A 109.5 . . ?
C12 C14 H14B 109.5 . . ?
H14A C14 H14B 109.5 . . ?

```
C12 C14 H14C 109.5 . . ?
H14A C14 H14C 109.5 . . ?
H14B C14 H14C 109.5 . . ?

loop_
 _geom_torsion_atom_site_label_1
 _geom_torsion_atom_site_label_2
 _geom_torsion_atom_site_label_3
 _geom_torsion_atom_site_label_4
 _geom_torsion
 _geom_torsion_site_symmetry_1
 _geom_torsion_site_symmetry_2
 _geom_torsion_site_symmetry_3
 _geom_torsion_site_symmetry_4
 _geom_torsion_publ_flag
N2 Zn1 N1 C2 -66.2(4) . . . . ?
Cl2 Zn1 N1 C2 176.0(3) . . . . ?
Cl1 Zn1 N1 C2 50.0(4) . . . . ?
N2 Zn1 N1 C4 110.1(4) . . . . ?
Cl2 Zn1 N1 C4 -7.7(4) . . . . ?
Cl1 Zn1 N1 C4 -133.7(3) . . . . ?
N1 Zn1 N2 C9 -72.0(4) . . . . ?
Cl2 Zn1 N2 C9 47.8(4) . . . . ?
Cl1 Zn1 N2 C9 173.4(3) . . . . ?
N1 Zn1 N2 C11 109.7(3) . . . . ?
Cl2 Zn1 N2 C11 -130.5(3) . . . . ?
Cl1 Zn1 N2 C11 -4.9(3) . . . . ?
C4 N1 C2 O1 1.7(6) . . . . ?
Zn1 N1 C2 O1 178.6(3) . . . . ?
C4 N1 C2 C1 -175.0(5) . . . . ?
Zn1 N1 C2 C1 2.0(7) . . . . ?
C3 O1 C2 N1 -3.3(6) . . . . ?
C3 O1 C2 C1 173.8(4) . . . . ?
C2 O1 C3 C4 3.2(5) . . . . ?
C2 N1 C4 C5 -124.6(5) . . . . ?
Zn1 N1 C4 C5 58.5(6) . . . . ?
C2 N1 C4 C3 0.5(5) . . . . ?
Zn1 N1 C4 C3 -176.3(3) . . . . ?
O1 C3 C4 N1 -2.2(5) . . . . ?
O1 C3 C4 C5 122.5(5) . . . . ?
N1 C4 C5 C6 58.5(6) . . . . ?
C3 C4 C5 C6 -60.5(7) . . . . ?
N1 C4 C5 C7 -177.4(5) . . . . ?
C3 C4 C5 C7 63.6(6) . . . . ?
```

C11 N2 C9 O2 -4.4(5) . . . . ?
Zn1 N2 C9 O2 177.0(3) . . . . ?
C11 N2 C9 C8 175.5(4) . . . . ?
Zn1 N2 C9 C8 -3.0(7) . . . . ?
C10 O2 C9 N2 -6.8(5) . . . . ?
C10 O2 C9 C8 173.3(4) . . . . ?
C9 O2 C10 C11 14.3(4) . . . . ?
C9 N2 C11 C10 13.0(4) . . . . ?
Zn1 N2 C11 C10 -168.5(3) . . . . ?
C9 N2 C11 C12 -110.8(4) . . . . ?
Zn1 N2 C11 C12 67.7(4) . . . . ?
O2 C10 C11 N2 -16.2(4) . . . . ?
O2 C10 C11 C12 104.3(4) . . . . ?
N2 C11 C12 C13 57.3(5) . . . . ?
C10 C11 C12 C13 -58.6(5) . . . . ?
N2 C11 C12 C14 -178.4(3) . . . . ?
C10 C11 C12 C14 65.7(5) . . . . ?

_diffrn_measured_fraction_theta_max 0.989
_diffrn_reflns_theta_full 30.59
_diffrn_measured_fraction_theta_full 0.989
_refine_diff_density_max 1.060
_refine_diff_density_min -0.716
_refine_diff_density_rms 0.110

data_compound_3

_audit_creation_method SHELXL-97
_chemical_name_systematic
;
 ?
;
_chemical_name_common ?
_chemical_melting_point ?
_chemical_formula_moiety ?
_chemical_formula_sum
 'C15 H23 Cl5 N2 Zn'
_chemical_formula_weight 473.97

loop_
 _atom_type_symbol
 _atom_type_description
 _atom_type_scat_dispersion_real
 _atom_type_scat_dispersion_imag

```
 _atom_type_scat_source
 'C'  'C'   0.0033   0.0016
 'International Tables Vol C Tables 4.2.6.8 and 6.1.1.4'
 'H'  'H'   0.0000   0.0000
 'International Tables Vol C Tables 4.2.6.8 and 6.1.1.4'
 'N'  'N'   0.0061   0.0033
 'International Tables Vol C Tables 4.2.6.8 and 6.1.1.4'
 'Zn'  'Zn'   0.2839   1.4301
 'International Tables Vol C Tables 4.2.6.8 and 6.1.1.4'
 'Cl'  'Cl'   0.1484   0.1585
 'International Tables Vol C Tables 4.2.6.8 and 6.1.1.4'

_symmetry_cell_setting            Tetragonal
_symmetry_space_group_name_H-M    I4

loop_
 _symmetry_equiv_pos_as_xyz
 'x, y, z'
 '-y, x, z'
 'y, -x, z'
 '-x, -y, z'
 'x+1/2, y+1/2, z+1/2'
 '-y+1/2, x+1/2, z+1/2'
 'y+1/2, -x+1/2, z+1/2'
 '-x+1/2, -y+1/2, z+1/2'

_cell_length_a                    19.852(3)
_cell_length_b                    19.852(3)
_cell_length_c                    10.7639(16)
_cell_angle_alpha                 90.00
_cell_angle_beta                  90.00
_cell_angle_gamma                 90.00
_cell_volume                      4241.9(11)
_cell_formula_units_Z             8
_cell_measurement_temperature     133(2)
_cell_measurement_reflns_used     6384
_cell_measurement_theta_min       2.90
_cell_measurement_theta_max       27.47

_exptl_crystal_description        prismatic
_exptl_crystal_colour             colorless
_exptl_crystal_size_max           0.50
_exptl_crystal_size_mid           0.15
_exptl_crystal_size_min           0.10
```

```
_exptl_crystal_density_meas       ?
_exptl_crystal_density_diffrn     1.484
_exptl_crystal_density_method     'not measured'
_exptl_crystal_F_000              1936
_exptl_absorpt_coefficient_mu     1.787
_exptl_absorpt_correction_type    multi-scan
_exptl_absorpt_correction_T_min   0.4686
_exptl_absorpt_correction_T_max   0.8415
_exptl_absorpt_process_details    sadabs

_exptl_special_details
;
 ?
;

_diffrn_ambient_temperature       133(2)
_diffrn_radiation_wavelength      0.71073
_diffrn_radiation_type            MoK\a
_diffrn_radiation_source          'fine-focus sealed tube'
_diffrn_radiation_monochromator   graphite
_diffrn_measurement_device_type   'Bruker APEX-II CCD'
_diffrn_measurement_method        '\f and \w scans'
_diffrn_detector_area_resol_mean  ?
_diffrn_standards_number          0
_diffrn_standards_interval_count  ?
_diffrn_standards_interval_time   ?
_diffrn_standards_decay_%         ?
_diffrn_reflns_number             7602
_diffrn_reflns_av_R_equivalents   0.0140
_diffrn_reflns_av_sigmaI/netI     0.0377
_diffrn_reflns_limit_h_min        -23
_diffrn_reflns_limit_h_max        22
_diffrn_reflns_limit_k_min        -7
_diffrn_reflns_limit_k_max        25
_diffrn_reflns_limit_l_min        -13
_diffrn_reflns_limit_l_max        13
_diffrn_reflns_theta_min          1.45
_diffrn_reflns_theta_max          26.98
_reflns_number_total              4529
_reflns_number_gt                 4300
_reflns_threshold_expression      >2sigma(I)

_computing_data_collection        'Bruker APEX2'
_computing_cell_refinement        'Bruker SAINT'
```

```
_computing_data_reduction         'Bruker SAINT'
_computing_structure_solution     'SHELXS-97 (Sheldrick, 1990)'
_computing_structure_refinement   'SHELXL-97 (Sheldrick, 1997)'
_computing_molecular_graphics     'Bruker SHELXTL'
_computing_publication_material   'Bruker SHELXTL'

_refine_special_details
;
 Refinement of F^2^ against ALL reflections.  The weighted R-factor wR and
 goodness of fit S are based on F^2^, conventional R-factors R are based
 on F, with F set to zero for negative F^2^. The threshold expression of
 F^2^ > 2sigma(F^2^) is used only for calculating R-factors(gt) etc. and is
 not relevant to the choice of reflections for refinement.  R-factors based
 on F^2^ are statistically about twice as large as those based on F, and R-
 factors based on ALL data will be even larger.
;

_refine_ls_structure_factor_coef  Fsqd
_refine_ls_matrix_type            full
_refine_ls_weighting_scheme       calc
_refine_ls_weighting_details
 'calc w=1/[\s^2^(Fo^2^)+(0.0471P)^2^+1.9547P] where P=(Fo^2^+2Fc^2^)/3'
_atom_sites_solution_primary      direct
_atom_sites_solution_secondary    difmap
_atom_sites_solution_hydrogens    geom
_refine_ls_hydrogen_treatment     constr
_refine_ls_extinction_method      none
_refine_ls_extinction_coef        ?
_refine_ls_abs_structure_details
 'Flack H D (1983), Acta Cryst. A39, 876-881'
_refine_ls_abs_structure_Flack    0.026(12)
_chemical_absolute_configuration  ad
_refine_ls_number_reflns          4529
_refine_ls_number_parameters      235
_refine_ls_number_restraints      25
_refine_ls_R_factor_all           0.0299
_refine_ls_R_factor_gt            0.0282
_refine_ls_wR_factor_ref          0.0850
_refine_ls_wR_factor_gt           0.0837
_refine_ls_goodness_of_fit_ref    1.088
_refine_ls_restrained_S_all       1.112
_refine_ls_shift/su_max           0.001
_refine_ls_shift/su_mean          0.000
```

```
loop_
 _atom_site_label
 _atom_site_type_symbol
 _atom_site_fract_x
 _atom_site_fract_y
 _atom_site_fract_z
 _atom_site_U_iso_or_equiv
 _atom_site_adp_type
 _atom_site_occupancy
 _atom_site_symmetry_multiplicity
 _atom_site_calc_flag
 _atom_site_refinement_flags
 _atom_site_disorder_assembly
 _atom_site_disorder_group
Zn1 Zn 0.087953(12) 0.425967(11) 0.25596(5) 0.02428(9) Uani 1 1 d . . .
Cl1 Cl 0.12972(4) 0.47958(4) 0.41950(7) 0.04073(19) Uani 1 1 d . . .
Cl2 Cl 0.11847(4) 0.48277(4) 0.08641(6) 0.03545(17) Uani 1 1 d . . .
N1 N 0.10513(10) 0.32197(9) 0.2644(3) 0.0281(4) Uani 1 1 d . . .
N2 N -0.01345(9) 0.40510(9) 0.2592(3) 0.0274(4) Uani 1 1 d . . .
H2A H -0.0317 0.4166 0.3348 0.033 Uiso 1 1 calc R . .
H2D H -0.0355 0.4287 0.1978 0.033 Uiso 1 1 calc R . .
C1 C 0.12238(16) 0.29256(15) 0.1402(3) 0.0378(7) Uani 1 1 d . . .
H1A H 0.0895 0.3085 0.0776 0.045 Uiso 1 1 calc R . .
H1B H 0.1192 0.2428 0.1445 0.045 Uiso 1 1 calc R . .
C2 C 0.19370(19) 0.31266(17) 0.0996(4) 0.0535(10) Uani 1 1 d . . .
H2B H 0.2042 0.2909 0.0191 0.064 Uiso 1 1 calc R . .
H2C H 0.1956 0.3621 0.0874 0.064 Uiso 1 1 calc R . .
C3 C 0.24591(17) 0.29223(18) 0.1945(5) 0.0617(12) Uani 1 1 d . . .
H3A H 0.2907 0.3091 0.1691 0.074 Uiso 1 1 calc R . .
H3B H 0.2482 0.2425 0.1998 0.074 Uiso 1 1 calc R . .
C4 C 0.22717(17) 0.32126(19) 0.3203(4) 0.0520(9) Uani 1 1 d . . .
H4A H 0.2304 0.3710 0.3170 0.062 Uiso 1 1 calc R . .
H4B H 0.2595 0.3050 0.3836 0.062 Uiso 1 1 calc R . .
C5 C 0.15571(15) 0.30118(15) 0.3587(3) 0.0393(7) Uani 1 1 d . . .
H5A H 0.1536 0.2517 0.3700 0.047 Uiso 1 1 calc R . .
H5B H 0.1447 0.3225 0.4393 0.047 Uiso 1 1 calc R . .
C6 C 0.03834(13) 0.29683(12) 0.3057(2) 0.0264(5) Uani 1 1 d . . .
H6A H 0.0334 0.3048 0.3960 0.032 Uiso 1 1 calc R . .
H6B H 0.0359 0.2476 0.2912 0.032 Uiso 1 1 calc R . .
C7 C -0.01941(12) 0.33111(11) 0.2375(2) 0.0258(5) Uani 1 1 d . . .
H7A H -0.0139 0.3225 0.1466 0.031 Uiso 1 1 calc R . .
C8 C -0.08791(13) 0.30377(14) 0.2774(3) 0.0324(6) Uani 1 1 d . . .
H8A H -0.0943 0.3122 0.3673 0.039 Uiso 1 1 calc R . .
H8B H -0.1238 0.3280 0.2319 0.039 Uiso 1 1 calc R . .
```

C9 C -0.09415(11) 0.22869(12) 0.2522(4) 0.0349(5) Uani 1 1 d . . .
C10 C -0.09420(18) 0.20600(19) 0.1292(3) 0.0475(8) Uani 1 1 d . . .
H10A H -0.0927 0.2375 0.0628 0.057 Uiso 1 1 calc R . .
C11 C -0.09647(19) 0.1369(2) 0.1042(5) 0.0636(12) Uani 1 1 d . . .
H11A H -0.0953 0.1214 0.0207 0.076 Uiso 1 1 calc R . .
C12 C -0.10030(19) 0.0919(2) 0.1991(6) 0.0658(13) Uani 1 1 d . . .
H12A H -0.1031 0.0451 0.1815 0.079 Uiso 1 1 calc R . .
C13 C -0.1001(2) 0.1132(2) 0.3190(6) 0.0671(15) Uani 1 1 d . . .
H13A H -0.1023 0.0812 0.3845 0.080 Uiso 1 1 calc R . .
C14 C -0.09681(17) 0.18267(19) 0.3471(4) 0.0481(9) Uani 1 1 d . . .
H14A H -0.0964 0.1974 0.4311 0.058 Uiso 1 1 calc R . .
C15 C 0.02297(17) 0.21653(17) 0.7356(4) 0.0482(8) Uani 1 1 d . . .
H15 H 0.0045 0.2623 0.7489 0.058 Uiso 1 1 d R A .
Cl3 Cl 0.06792(7) 0.20409(7) 0.59574(11) 0.0656(3) Uani 0.85 1 d PU A 1
Cl4 Cl 0.07278(10) 0.20027(9) 0.86146(13) 0.0843(5) Uani 0.85 1 d PU A 1
Cl5 Cl -0.04599(7) 0.15926(7) 0.7289(2) 0.0885(6) Uani 0.85 1 d PU A 1
Cl6 Cl -0.0073(5) 0.1755(5) 0.8662(11) 0.104(3) Uani 0.15 1 d PU A 2
Cl7 Cl 0.0051(9) 0.1950(5) 0.6154(8) 0.110(4) Uani 0.15 1 d PU A 2
Cl8 Cl 0.1054(3) 0.2435(5) 0.7962(14) 0.105(4) Uani 0.15 1 d PU A 2

loop_
 _atom_site_aniso_label
 _atom_site_aniso_U_11
 _atom_site_aniso_U_22
 _atom_site_aniso_U_33
 _atom_site_aniso_U_23
 _atom_site_aniso_U_13
 _atom_site_aniso_U_12
Zn1 0.02936(14) 0.01782(12) 0.02566(14) -0.00002(14) 0.00100(14) -0.00012(8)
Cl1 0.0518(5) 0.0404(4) 0.0301(4) -0.0053(3) -0.0075(3) -0.0066(3)
Cl2 0.0461(4) 0.0328(4) 0.0275(3) 0.0057(3) 0.0041(3) -0.0037(3)
N1 0.0283(9) 0.0196(8) 0.0365(11) -0.0008(11) 0.0052(11) -0.0005(7)
N2 0.0302(9) 0.0209(8) 0.0311(9) 0.0009(11) -0.0012(12) 0.0039(7)
C1 0.0401(16) 0.0273(13) 0.0461(16) -0.0056(12) 0.0171(13) 0.0036(12)
C2 0.056(2) 0.0314(15) 0.073(2) -0.0051(15) 0.036(2) 0.0003(14)
C3 0.0324(17) 0.0318(16) 0.121(4) 0.0050(19) 0.0229(19) 0.0061(13)
C4 0.0300(15) 0.0380(17) 0.088(3) 0.0140(18) -0.0030(16) 0.0023(13)
C5 0.0316(15) 0.0291(14) 0.0573(18) 0.0075(13) -0.0041(14) 0.0029(12)
C6 0.0296(13) 0.0213(11) 0.0284(11) 0.0014(9) 0.0027(10) -0.0008(9)
C7 0.0315(11) 0.0207(10) 0.0252(13) 0.0004(9) 0.0004(10) -0.0013(8)
C8 0.0298(12) 0.0331(12) 0.0344(16) 0.0001(11) 0.0033(10) -0.0010(9)
C9 0.0218(10) 0.0322(12) 0.0506(14) 0.0044(16) -0.0008(15) -0.0047(8)
C10 0.0413(18) 0.046(2) 0.055(2) -0.0101(16) -0.0089(14) -0.0062(14)
C11 0.047(2) 0.048(2) 0.096(3) -0.032(2) -0.009(2) -0.0094(16)

C12 0.0290(17) 0.0358(19) 0.133(4) -0.014(2) 0.004(2) -0.0046(14)
C13 0.036(2) 0.045(2) 0.120(4) 0.031(3) 0.008(2) -0.0025(16)
C14 0.0335(16) 0.047(2) 0.064(2) 0.0145(17) 0.0125(15) -0.0022(14)
C15 0.0438(16) 0.0488(17) 0.052(2) 0.0154(16) 0.0041(15) 0.0126(13)
Cl3 0.0633(7) 0.0913(9) 0.0421(5) 0.0190(6) 0.0080(6) 0.0242(6)
Cl4 0.1105(12) 0.0963(11) 0.0460(6) -0.0111(7) -0.0246(7) 0.0492(10)
Cl5 0.0570(7) 0.0681(7) 0.1405(18) -0.0061(9) 0.0203(9) -0.0040(5)
Cl6 0.088(5) 0.098(6) 0.125(7) 0.071(5) 0.065(5) 0.043(5)
Cl7 0.199(11) 0.079(6) 0.051(4) 0.001(4) -0.032(6) 0.017(7)
Cl8 0.031(3) 0.072(4) 0.211(12) -0.018(6) -0.024(5) 0.016(3)

_geom_special_details
;
 All esds (except the esd in the dihedral angle between two l.s. planes)
 are estimated using the full covariance matrix. The cell esds are taken
 into account individually in the estimation of esds in distances, angles
 and torsion angles; correlations between esds in cell parameters are only
 used when they are defined by crystal symmetry. An approximate (isotropic)
 treatment of cell esds is used for estimating esds involving l.s. planes.
;

loop_
 _geom_bond_atom_site_label_1
 _geom_bond_atom_site_label_2
 _geom_bond_distance
 _geom_bond_site_symmetry_2
 _geom_bond_publ_flag
Zn1 N2 2.0554(19) . ?
Zn1 N1 2.0944(18) . ?
Zn1 Cl1 2.2178(9) . ?
Zn1 Cl2 2.2292(9) . ?
N1 C6 1.485(3) . ?
N1 C5 1.487(4) . ?
N1 C1 1.498(4) . ?
N2 C7 1.492(3) . ?
N2 H2A 0.9200 . ?
N2 H2D 0.9200 . ?
C1 C2 1.535(4) . ?
C1 H1A 0.9900 . ?
C1 H1B 0.9900 . ?
C2 C3 1.511(7) . ?
C2 H2B 0.9900 . ?
C2 H2C 0.9900 . ?
C3 C4 1.518(6) . ?

```
C3 H3A 0.9900 . ?
C3 H3B 0.9900 . ?
C4 C5 1.530(4) . ?
C4 H4A 0.9900 . ?
C4 H4B 0.9900 . ?
C5 H5A 0.9900 . ?
C5 H5B 0.9900 . ?
C6 C7 1.522(3) . ?
C6 H6A 0.9900 . ?
C6 H6B 0.9900 . ?
C7 C8 1.526(3) . ?
C7 H7A 1.0000 . ?
C8 C9 1.520(4) . ?
C8 H8A 0.9900 . ?
C8 H8B 0.9900 . ?
C9 C14 1.372(5) . ?
C9 C10 1.398(5) . ?
C10 C11 1.399(5) . ?
C10 H10A 0.9500 . ?
C11 C12 1.359(8) . ?
C11 H11A 0.9500 . ?
C12 C13 1.359(7) . ?
C12 H12A 0.9500 . ?
C13 C14 1.412(6) . ?
C13 H13A 0.9500 . ?
C14 H14A 0.9500 . ?
C15 Cl7 1.408(10) . ?
C15 Cl4 1.708(4) . ?
C15 Cl6 1.732(8) . ?
C15 Cl3 1.767(4) . ?
C15 Cl5 1.781(4) . ?
C15 Cl8 1.841(8) . ?
C15 H15 0.9900 . ?

loop_
 _geom_angle_atom_site_label_1
 _geom_angle_atom_site_label_2
 _geom_angle_atom_site_label_3
 _geom_angle
 _geom_angle_site_symmetry_1
 _geom_angle_site_symmetry_3
 _geom_angle_publ_flag
N2 Zn1 N1 87.71(7) . . ?
N2 Zn1 Cl1 116.71(9) . . ?
```

N1 Zn1 Cl1 112.20(8) . . ?
N2 Zn1 Cl2 112.45(8) . . ?
N1 Zn1 Cl2 119.33(8) . . ?
Cl1 Zn1 Cl2 107.78(3) . . ?
C6 N1 C5 107.8(2) . . ?
C6 N1 C1 109.9(2) . . ?
C5 N1 C1 110.3(2) . . ?
C6 N1 Zn1 101.47(14) . . ?
C5 N1 Zn1 114.40(18) . . ?
C1 N1 Zn1 112.50(19) . . ?
C7 N2 Zn1 105.88(14) . . ?
C7 N2 H2A 110.6 . . ?
Zn1 N2 H2A 110.6 . . ?
C7 N2 H2D 110.6 . . ?
Zn1 N2 H2D 110.6 . . ?
H2A N2 H2D 108.7 . . ?
N1 C1 C2 111.3(3) . . ?
N1 C1 H1A 109.4 . . ?
C2 C1 H1A 109.4 . . ?
N1 C1 H1B 109.4 . . ?
C2 C1 H1B 109.4 . . ?
H1A C1 H1B 108.0 . . ?
C3 C2 C1 111.7(3) . . ?
C3 C2 H2B 109.3 . . ?
C1 C2 H2B 109.3 . . ?
C3 C2 H2C 109.3 . . ?
C1 C2 H2C 109.3 . . ?
H2B C2 H2C 107.9 . . ?
C2 C3 C4 109.5(3) . . ?
C2 C3 H3A 109.8 . . ?
C4 C3 H3A 109.8 . . ?
C2 C3 H3B 109.8 . . ?
C4 C3 H3B 109.8 . . ?
H3A C3 H3B 108.2 . . ?
C3 C4 C5 111.7(3) . . ?
C3 C4 H4A 109.3 . . ?
C5 C4 H4A 109.3 . . ?
C3 C4 H4B 109.3 . . ?
C5 C4 H4B 109.3 . . ?
H4A C4 H4B 107.9 . . ?
N1 C5 C4 111.7(3) . . ?
N1 C5 H5A 109.3 . . ?
C4 C5 H5A 109.3 . . ?
N1 C5 H5B 109.3 . . ?

C4 C5 H5B 109.3 . . ?
H5A C5 H5B 107.9 . . ?
N1 C6 C7 112.2(2) . . ?
N1 C6 H6A 109.2 . . ?
C7 C6 H6A 109.2 . . ?
N1 C6 H6B 109.2 . . ?
C7 C6 H6B 109.2 . . ?
H6A C6 H6B 107.9 . . ?
N2 C7 C6 107.8(2) . . ?
N2 C7 C8 112.2(2) . . ?
C6 C7 C8 112.1(2) . . ?
N2 C7 H7A 108.2 . . ?
C6 C7 H7A 108.2 . . ?
C8 C7 H7A 108.2 . . ?
C9 C8 C7 111.8(2) . . ?
C9 C8 H8A 109.3 . . ?
C7 C8 H8A 109.3 . . ?
C9 C8 H8B 109.3 . . ?
C7 C8 H8B 109.3 . . ?
H8A C8 H8B 107.9 . . ?
C14 C9 C10 119.4(3) . . ?
C14 C9 C8 121.6(3) . . ?
C10 C9 C8 119.0(3) . . ?
C9 C10 C11 119.9(4) . . ?
C9 C10 H10A 120.1 . . ?
C11 C10 H10A 120.1 . . ?
C12 C11 C10 120.1(5) . . ?
C12 C11 H11A 119.9 . . ?
C10 C11 H11A 119.9 . . ?
C13 C12 C11 120.5(4) . . ?
C13 C12 H12A 119.7 . . ?
C11 C12 H12A 119.7 . . ?
C12 C13 C14 120.6(4) . . ?
C12 C13 H13A 119.7 . . ?
C14 C13 H13A 119.7 . . ?
C9 C14 C13 119.5(4) . . ?
C9 C14 H14A 120.3 . . ?
C13 C14 H14A 120.3 . . ?
Cl7 C15 Cl4 144.8(6) . . ?
Cl7 C15 Cl6 121.0(8) . . ?
Cl4 C15 Cl6 57.9(4) . . ?
Cl7 C15 Cl3 45.7(7) . . ?
Cl4 C15 Cl3 110.91(19) . . ?
Cl6 C15 Cl3 143.2(4) . . ?

```
C17 C15 C15 64.8(7) . . ?
C14 C15 C15 110.9(2) . . ?
C16 C15 C15 57.7(5) . . ?
C13 C15 C15 105.3(2) . . ?
C17 C15 C18 129.6(9) . . ?
C14 C15 C18 42.2(4) . . ?
C16 C15 C18 99.1(7) . . ?
C13 C15 C18 83.9(5) . . ?
C15 C15 C18 152.0(4) . . ?
C17 C15 H15 108.6 . . ?
C14 C15 H15 105.8 . . ?
C16 C15 H15 100.7 . . ?
C13 C15 H15 116.0 . . ?
C15 C15 H15 107.9 . . ?
C18 C15 H15 90.6 . . ?

loop_
 _geom_hbond_atom_site_label_D
 _geom_hbond_atom_site_label_H
 _geom_hbond_atom_site_label_A
 _geom_hbond_distance_DH
 _geom_hbond_distance_HA
 _geom_hbond_distance_DA
 _geom_hbond_angle_DHA
 _geom_hbond_site_symmetry_A
N2 H2A Cl2  0.92 2.81 3.554(3) 138.6 7_455
N2 H2A Cl1  0.92 2.98 3.681(2) 134.5 4_565
N2 H2D Cl2  0.92 2.69 3.572(2) 160.6 4_565

_diffrn_measured_fraction_theta_max    0.996
_diffrn_reflns_theta_full              26.98
_diffrn_measured_fraction_theta_full   0.996
_refine_diff_density_max    0.768
_refine_diff_density_min   -0.414
_refine_diff_density_rms    0.067

data_compound_4

_audit_creation_method            SHELXL-97
_chemical_name_systematic
;
 ?
;
_chemical_name_common             ?
```

```
_chemical_melting_point           ?
_chemical_formula_moiety          ?
_chemical_formula_sum
 'C12 H18 Cl2 N2 O Zn'
_chemical_formula_weight          342.55
_chemical_absolute_configuration  'ad'

loop_
 _atom_type_symbol
 _atom_type_description
 _atom_type_scat_dispersion_real
 _atom_type_scat_dispersion_imag
 _atom_type_scat_source
 'C'  'C'   0.0181   0.0091
 'International Tables Vol C Tables 4.2.6.8 and 6.1.1.4'
 'H'  'H'   0.0000   0.0000
 'International Tables Vol C Tables 4.2.6.8 and 6.1.1.4'
 'O'  'O'   0.0492   0.0322
 'International Tables Vol C Tables 4.2.6.8 and 6.1.1.4'
 'Zn'  'Zn'  -1.5491   0.6778
 'International Tables Vol C Tables 4.2.6.8 and 6.1.1.4'
 'Cl'  'Cl'   0.3639   0.7018
 'International Tables Vol C Tables 4.2.6.8 and 6.1.1.4'
 'N'  'N'   0.0311   0.0180
 'International Tables Vol C Tables 4.2.6.8 and 6.1.1.4'

_symmetry_cell_setting            orthorhombic
_symmetry_space_group_name_H-M    'P 21 21 21'

loop_
 _symmetry_equiv_pos_as_xyz
 'x, y, z'
 '-x+1/2, -y, z+1/2'
 'x+1/2, -y+1/2, -z'
 '-x, y+1/2, -z+1/2'

_cell_length_a                    9.65410(10)
_cell_length_b                    10.24400(10)
_cell_length_c                    14.76100(10)
_cell_angle_alpha                 90.00
_cell_angle_beta                  90.00
_cell_angle_gamma                 90.00
_cell_volume                      1459.81(2)
_cell_formula_units_Z             4
```

```
_cell_measurement_temperature     291(2)
_cell_measurement_reflns_used     9663
_cell_measurement_theta_min       2.9912
_cell_measurement_theta_max       69.6755

_exptl_crystal_description        block
_exptl_crystal_colour             colorless
_exptl_crystal_size_max           0.36
_exptl_crystal_size_mid           0.32
_exptl_crystal_size_min           0.30
_exptl_crystal_density_meas       ?
_exptl_crystal_density_diffrn     1.559
_exptl_crystal_density_method     'not measured'
_exptl_crystal_F_000              704
_exptl_absorpt_coefficient_mu     5.620
_exptl_absorpt_correction_type    'multi-scan'
_exptl_absorpt_correction_T_min   0.2368
_exptl_absorpt_correction_T_max   0.2834
_exptl_absorpt_process_details
;
CrysAlisPro, Oxford Diffraction Ltd.,
Version 1.171.34.44 (release 25-10-2010 CrysAlis171 .NET)
(compiled Oct 25 2010,18:11:34)
Empirical absorption correction using spherical harmonics,
 implemented in SCALE3 ABSPACK scaling algorithm.
;

_exptl_special_details
;
 ?
;

_diffrn_ambient_temperature       291(2)
_diffrn_radiation_wavelength      1.54184
_diffrn_radiation_type            CuK\a
_diffrn_radiation_source          'Enhance Ultra (Cu) X-ray Source'
_diffrn_radiation_monochromator   'mirror'
_diffrn_measurement_device_type   'Gemini S Ultra, Oxford Diffraction'
_diffrn_measurement_method        '\w scans'
_diffrn_detector_area_resol_mean  15.9149
_diffrn_standards_number          ?
_diffrn_standards_interval_count  ?
_diffrn_standards_interval_time   ?
_diffrn_standards_decay_%         ?
```

```
_diffrn_reflns_number             10362
_diffrn_reflns_av_R_equivalents   0.0240
_diffrn_reflns_av_sigmaI/netI     0.0179
_diffrn_reflns_limit_h_min        -11
_diffrn_reflns_limit_h_max        11
_diffrn_reflns_limit_k_min        -12
_diffrn_reflns_limit_k_max        12
_diffrn_reflns_limit_l_min        -16
_diffrn_reflns_limit_l_max        17
_diffrn_reflns_theta_min          5.26
_diffrn_reflns_theta_max          69.80
_reflns_number_total              2724
_reflns_number_gt                 2705
_reflns_threshold_expression      >2sigma(I)

_computing_data_collection        'CrysAlisPro (Oxford Diffraction Ltd.)'
_computing_cell_refinement        'CrysAlisPro (Oxford Diffraction Ltd.)'
_computing_data_reduction         'CrysAlisPro (Oxford Diffraction Ltd.)'
_computing_structure_solution     'SHELXS-97 (Sheldrick, 1997)'
_computing_structure_refinement   'SHELXL-97 (Sheldrick, 1997)'
_computing_molecular_graphics     'Ortep-3 (L. J. Farrugia, 2001)'
_computing_publication_material   SHELXL-97

_refine_special_details
;
 Refinement of F^2^ against ALL reflections.  The weighted R-factor wR and
 goodness of fit S are based on F^2^, conventional R-factors R are based
 on F, with F set to zero for negative F^2^. The threshold expression of
 F^2^ > 2sigma(F^2^) is used only for calculating R-factors(gt) etc. and is
 not relevant to the choice of reflections for refinement.  R-factors based
 on F^2^ are statistically about twice as large as those based on F, and R-
 factors based on ALL data will be even larger.
;

_refine_ls_structure_factor_coef  Fsqd
_refine_ls_matrix_type            full
_refine_ls_weighting_scheme       calc
_refine_ls_weighting_details
 'calc w=1/[\s^2^(Fo^2^)+(0.0425P)^2^+0.1948P] where P=(Fo^2^+2Fc^2^)/3'
_atom_sites_solution_primary      direct
_atom_sites_solution_secondary    difmap
_atom_sites_solution_hydrogens    geom
_refine_ls_hydrogen_treatment     constr
_refine_ls_extinction_method      none
```

```
_refine_ls_extinction_coef        ?
_refine_ls_abs_structure_details
 'Flack H D (1983), Acta Cryst. A39, 876-881'
_refine_ls_abs_structure_Flack    -0.001(15)
_refine_ls_number_reflns          2724
_refine_ls_number_parameters      163
_refine_ls_number_restraints      0
_refine_ls_R_factor_all           0.0230
_refine_ls_R_factor_gt            0.0229
_refine_ls_wR_factor_ref          0.0611
_refine_ls_wR_factor_gt           0.0610
_refine_ls_goodness_of_fit_ref    1.045
_refine_ls_restrained_S_all       1.045
_refine_ls_shift/su_max           0.001
_refine_ls_shift/su_mean          0.000

loop_
 _atom_site_label
 _atom_site_type_symbol
 _atom_site_fract_x
 _atom_site_fract_y
 _atom_site_fract_z
 _atom_site_U_iso_or_equiv
 _atom_site_adp_type
 _atom_site_occupancy
 _atom_site_symmetry_multiplicity
 _atom_site_calc_flag
 _atom_site_refinement_flags
 _atom_site_disorder_assembly
 _atom_site_disorder_group
Zn1 Zn 0.26939(3) -0.10272(2) 0.489654(16) 0.03447(9) Uani 1 1 d . . .
Cl2 Cl 0.07153(6) -0.06344(6) 0.56333(4) 0.05233(15) Uani 1 1 d . . .
Cl1 Cl 0.45017(6) -0.07852(7) 0.58033(4) 0.05903(17) Uani 1 1 d . . .
N2 N 0.30416(16) -0.00953(15) 0.36264(11) 0.0312(3) Uani 1 1 d . . .
O1 O 0.30342(19) 0.26913(14) 0.38823(11) 0.0482(4) Uani 1 1 d . . .
N1 N 0.25192(18) -0.27258(14) 0.41573(10) 0.0342(3) Uani 1 1 d . . .
H1B H 0.3318 -0.3178 0.4172 0.041 Uiso 1 1 calc R . .
H1A H 0.1839 -0.3232 0.4381 0.041 Uiso 1 1 calc R . .
C12 C 0.4323(2) 0.0710(2) 0.35765(16) 0.0430(5) Uani 1 1 d . . .
H12A H 0.4495 0.0955 0.2952 0.052 Uiso 1 1 calc R . .
H12B H 0.5106 0.0199 0.3786 0.052 Uiso 1 1 calc R . .
C4 C 0.2205(2) -0.34072(17) 0.25217(12) 0.0305(4) Uani 1 1 d . . .
C3 C 0.1913(2) -0.3112(2) 0.16207(14) 0.0409(5) Uani 1 1 d . . .
H3 H 0.1781 -0.2247 0.1451 0.049 Uiso 1 1 calc R . .
```

```
C2 C 0.1815(3) -0.4088(2) 0.09745(14) 0.0475(5) Uani 1 1 d . . .
H2 H 0.1624 -0.3878 0.0374 0.057 Uiso 1 1 calc R . .
C6 C 0.2324(3) -0.56743(19) 0.21042(15) 0.0463(5) Uani 1 1 d . . .
H6 H 0.2475 -0.6540 0.2267 0.056 Uiso 1 1 calc R . .
C7 C 0.2191(2) -0.23130(17) 0.32183(11) 0.0306(4) Uani 1 1 d . . .
H7 H 0.1257 -0.1939 0.3223 0.037 Uiso 1 1 calc R . .
C10 C 0.1807(3) 0.1949(2) 0.39400(17) 0.0468(5) Uani 1 1 d . . .
H10A H 0.1028 0.2487 0.3759 0.056 Uiso 1 1 calc R . .
H10B H 0.1661 0.1688 0.4564 0.056 Uiso 1 1 calc R . .
C5 C 0.2426(3) -0.46980(18) 0.27545(13) 0.0397(5) Uani 1 1 d . . .
H5 H 0.2643 -0.4914 0.3350 0.048 Uiso 1 1 calc R . .
C8 C 0.3199(2) -0.12194(18) 0.29874(13) 0.0353(4) Uani 1 1 d . . .
H8B H 0.4140 -0.1548 0.3019 0.042 Uiso 1 1 calc R . .
H8A H 0.3031 -0.0924 0.2373 0.042 Uiso 1 1 calc R . .
C11 C 0.4191(3) 0.1921(2) 0.41471(17) 0.0468(5) Uani 1 1 d . . .
H11B H 0.4090 0.1673 0.4778 0.056 Uiso 1 1 calc R . .
H11A H 0.5029 0.2436 0.4091 0.056 Uiso 1 1 calc R . .
C9 C 0.1854(2) 0.07440(19) 0.33486(14) 0.0391(4) Uani 1 1 d . . .
H9B H 0.0995 0.0261 0.3409 0.047 Uiso 1 1 calc R . .
H9A H 0.1958 0.0997 0.2719 0.047 Uiso 1 1 calc R . .
C1 C 0.2001(3) -0.5371(2) 0.12263(16) 0.0462(5) Uani 1 1 d . . .
H1 H 0.1908 -0.6032 0.0799 0.055 Uiso 1 1 calc R . .

loop_
 _atom_site_aniso_label
 _atom_site_aniso_U_11
 _atom_site_aniso_U_22
 _atom_site_aniso_U_33
 _atom_site_aniso_U_23
 _atom_site_aniso_U_13
 _atom_site_aniso_U_12
Zn1 0.04064(15) 0.03685(13) 0.02591(12) -0.00219(9) -0.00152(10) 0.00303(10)
Cl2 0.0455(3) 0.0643(3) 0.0472(3) -0.0035(2) 0.0115(2) 0.0003(2)
Cl1 0.0525(3) 0.0757(4) 0.0488(3) -0.0092(3) -0.0202(2) 0.0151(3)
N2 0.0352(8) 0.0287(7) 0.0298(8) -0.0031(6) 0.0019(6) -0.0035(6)
O1 0.0666(10) 0.0297(6) 0.0483(9) -0.0017(6) -0.0043(8) -0.0029(7)
N1 0.0432(9) 0.0323(7) 0.0272(7) 0.0018(6) -0.0002(7) 0.0002(7)
C12 0.0403(11) 0.0416(11) 0.0469(12) -0.0078(9) 0.0054(9) -0.0103(9)
C4 0.0326(9) 0.0299(8) 0.0289(9) -0.0016(7) 0.0005(8) -0.0029(8)
C3 0.0545(13) 0.0326(9) 0.0356(10) 0.0015(8) -0.0068(9) -0.0017(9)
C2 0.0663(14) 0.0465(11) 0.0297(9) -0.0011(9) -0.0095(9) -0.0052(10)
C6 0.0685(15) 0.0302(9) 0.0403(10) -0.0012(7) 0.0048(11) -0.0008(10)
C7 0.0363(9) 0.0292(8) 0.0262(8) -0.0006(7) 0.0014(8) -0.0003(7)
C10 0.0556(14) 0.0338(10) 0.0509(13) 0.0001(9) 0.0008(11) 0.0066(9)
```

```
C5 0.0579(13) 0.0324(9) 0.0289(9) 0.0018(7) 0.0029(10) 0.0007(10)
C8 0.0466(11) 0.0312(9) 0.0279(8) -0.0055(7) 0.0071(8) -0.0056(8)
C11 0.0537(13) 0.0381(11) 0.0484(12) -0.0084(9) -0.0039(10) -0.0095(9)
C9 0.0449(11) 0.0339(9) 0.0384(10) 0.0032(8) -0.0071(8) -0.0012(8)
C1 0.0616(14) 0.0388(10) 0.0382(10) -0.0107(9) 0.0029(10) -0.0052(9)

_geom_special_details
;
 All esds (except the esd in the dihedral angle between two l.s. planes)
 are estimated using the full covariance matrix.  The cell esds are taken
 into account individually in the estimation of esds in distances, angles
 and torsion angles; correlations between esds in cell parameters are only
 used when they are defined by crystal symmetry.  An approximate (isotropic)
 treatment of cell esds is used for estimating esds involving l.s. planes.
;

loop_
 _geom_bond_atom_site_label_1
 _geom_bond_atom_site_label_2
 _geom_bond_distance
 _geom_bond_site_symmetry_2
 _geom_bond_publ_flag
Zn1 N1 2.0608(15) . ?
Zn1 N2 2.1305(16) . ?
Zn1 Cl1 2.2134(6) . ?
Zn1 Cl2 2.2346(6) . ?
N2 C12 1.488(2) . ?
N2 C9 1.490(3) . ?
N2 C8 1.496(2) . ?
O1 C10 1.410(3) . ?
O1 C11 1.422(3) . ?
N1 C7 1.483(2) . ?
N1 H1B 0.9000 . ?
N1 H1A 0.9000 . ?
C12 C11 1.505(3) . ?
C12 H12A 0.9700 . ?
C12 H12B 0.9700 . ?
C4 C5 1.383(3) . ?
C4 C3 1.393(3) . ?
C4 C7 1.521(2) . ?
C3 C2 1.385(3) . ?
C3 H3 0.9300 . ?
C2 C1 1.378(3) . ?
C2 H2 0.9300 . ?
```

```
C6 C1 1.369(3) . ?
C6 C5 1.390(3) . ?
C6 H6 0.9300 . ?
C7 C8 1.522(2) . ?
C7 H7 0.9800 . ?
C10 C9 1.512(3) . ?
C10 H10A 0.9700 . ?
C10 H10B 0.9700 . ?
C5 H5 0.9300 . ?
C8 H8B 0.9700 . ?
C8 H8A 0.9700 . ?
C11 H11B 0.9700 . ?
C11 H11A 0.9700 . ?
C9 H9B 0.9700 . ?
C9 H9A 0.9700 . ?
C1 H1 0.9300 . ?

loop_
 _geom_angle_atom_site_label_1
 _geom_angle_atom_site_label_2
 _geom_angle_atom_site_label_3
 _geom_angle
 _geom_angle_site_symmetry_1
 _geom_angle_site_symmetry_3
 _geom_angle_publ_flag
N1 Zn1 N2 85.71(6) . . ?
N1 Zn1 Cl1 118.64(5) . . ?
N2 Zn1 Cl1 110.96(5) . . ?
N1 Zn1 Cl2 109.86(5) . . ?
N2 Zn1 Cl2 118.84(5) . . ?
Cl1 Zn1 Cl2 111.07(2) . . ?
C12 N2 C9 107.81(15) . . ?
C12 N2 C8 108.12(15) . . ?
C9 N2 C8 110.40(15) . . ?
C12 N2 Zn1 115.01(13) . . ?
C9 N2 Zn1 112.29(12) . . ?
C8 N2 Zn1 103.05(10) . . ?
C10 O1 C11 110.10(16) . . ?
C7 N1 Zn1 105.76(10) . . ?
C7 N1 H1B 110.6 . . ?
Zn1 N1 H1B 110.6 . . ?
C7 N1 H1A 110.6 . . ?
Zn1 N1 H1A 110.6 . . ?
H1B N1 H1A 108.7 . . ?
```

N2 C12 C11 111.02(17) . . ?
N2 C12 H12A 109.4 . . ?
C11 C12 H12A 109.4 . . ?
N2 C12 H12B 109.4 . . ?
C11 C12 H12B 109.4 . . ?
H12A C12 H12B 108.0 . . ?
C5 C4 C3 118.44(17) . . ?
C5 C4 C7 122.55(16) . . ?
C3 C4 C7 118.93(16) . . ?
C2 C3 C4 120.99(19) . . ?
C2 C3 H3 119.5 . . ?
C4 C3 H3 119.5 . . ?
C1 C2 C3 119.6(2) . . ?
C1 C2 H2 120.2 . . ?
C3 C2 H2 120.2 . . ?
C1 C6 C5 120.44(18) . . ?
C1 C6 H6 119.8 . . ?
C5 C6 H6 119.8 . . ?
N1 C7 C4 114.81(14) . . ?
N1 C7 C8 106.41(15) . . ?
C4 C7 C8 112.64(14) . . ?
N1 C7 H7 107.6 . . ?
C4 C7 H7 107.6 . . ?
C8 C7 H7 107.6 . . ?
O1 C10 C9 112.33(19) . . ?
O1 C10 H10A 109.1 . . ?
C9 C10 H10A 109.1 . . ?
O1 C10 H10B 109.1 . . ?
C9 C10 H10B 109.1 . . ?
H10A C10 H10B 107.9 . . ?
C4 C5 C6 120.37(18) . . ?
C4 C5 H5 119.8 . . ?
C6 C5 H5 119.8 . . ?
N2 C8 C7 111.13(15) . . ?
N2 C8 H8B 109.4 . . ?
C7 C8 H8B 109.4 . . ?
N2 C8 H8A 109.4 . . ?
C7 C8 H8A 109.4 . . ?
H8B C8 H8A 108.0 . . ?
O1 C11 C12 111.73(18) . . ?
O1 C11 H11B 109.3 . . ?
C12 C11 H11B 109.3 . . ?
O1 C11 H11A 109.3 . . ?
C12 C11 H11A 109.3 . . ?

H11B C11 H11A 107.9 . . ?
N2 C9 C10 109.58(16) . . ?
N2 C9 H9B 109.8 . . ?
C10 C9 H9B 109.8 . . ?
N2 C9 H9A 109.8 . . ?
C10 C9 H9A 109.8 . . ?
H9B C9 H9A 108.2 . . ?
C6 C1 C2 120.14(19) . . ?
C6 C1 H1 119.9 . . ?
C2 C1 H1 119.9 . . ?

loop_
 _geom_torsion_atom_site_label_1
 _geom_torsion_atom_site_label_2
 _geom_torsion_atom_site_label_3
 _geom_torsion_atom_site_label_4
 _geom_torsion
 _geom_torsion_site_symmetry_1
 _geom_torsion_site_symmetry_2
 _geom_torsion_site_symmetry_3
 _geom_torsion_site_symmetry_4
 _geom_torsion_publ_flag
N1 Zn1 N2 C12 -126.79(14) . . . . ?
Cl1 Zn1 N2 C12 -7.72(14) . . . . ?
Cl2 Zn1 N2 C12 122.86(12) . . . . ?
N1 Zn1 N2 C9 109.42(13) . . . . ?
Cl1 Zn1 N2 C9 -131.50(11) . . . . ?
Cl2 Zn1 N2 C9 -0.92(13) . . . . ?
N1 Zn1 N2 C8 -9.37(12) . . . . ?
Cl1 Zn1 N2 C8 109.70(11) . . . . ?
Cl2 Zn1 N2 C8 -119.72(11) . . . . ?
N2 Zn1 N1 C7 -20.99(12) . . . . ?
Cl1 Zn1 N1 C7 -132.57(11) . . . . ?
Cl2 Zn1 N1 C7 98.16(12) . . . . ?
C9 N2 C12 C11 56.0(2) . . . . ?
C8 N2 C12 C11 175.40(18) . . . . ?
Zn1 N2 C12 C11 -70.1(2) . . . . ?
C5 C4 C3 C2 1.3(3) . . . . ?
C7 C4 C3 C2 -175.3(2) . . . . ?
C4 C3 C2 C1 0.4(4) . . . . ?
Zn1 N1 C7 C4 172.15(13) . . . . ?
Zn1 N1 C7 C8 46.82(16) . . . . ?
C5 C4 C7 N1 3.7(3) . . . . ?
C3 C4 C7 N1 -179.84(18) . . . . ?

C5 C4 C7 C8 125.7(2) . . . . ?
C3 C4 C7 C8 -57.8(3) . . . . ?
C11 O1 C10 C9 -58.9(2) . . . . ?
C3 C4 C5 C6 -1.5(4) . . . . ?
C7 C4 C5 C6 175.0(2) . . . . ?
C1 C6 C5 C4 -0.1(4) . . . . ?
C12 N2 C8 C7 160.90(18) . . . . ?
C9 N2 C8 C7 -81.38(19) . . . . ?
Zn1 N2 C8 C7 38.73(17) . . . . ?
N1 C7 C8 N2 -59.8(2) . . . . ?
C4 C7 C8 N2 173.53(15) . . . . ?
C10 O1 C11 C12 57.6(2) . . . . ?
N2 C12 C11 O1 -57.6(3) . . . . ?
C12 N2 C9 C10 -56.0(2) . . . . ?
C8 N2 C9 C10 -173.95(16) . . . . ?
Zn1 N2 C9 C10 71.67(18) . . . . ?
O1 C10 C9 N2 59.2(2) . . . . ?
C5 C6 C1 C2 1.9(4) . . . . ?
C3 C2 C1 C6 -2.0(4) . . . . ?

loop_
 _geom_hbond_atom_site_label_D
 _geom_hbond_atom_site_label_H
 _geom_hbond_atom_site_label_A
 _geom_hbond_distance_DH
 _geom_hbond_distance_HA
 _geom_hbond_distance_DA
 _geom_hbond_angle_DHA
 _geom_hbond_site_symmetry_A
N1 H1B Cl2 0.90 2.63 3.5267(18) 174.1 3_546
N1 H1A Cl1 0.90 2.49 3.2888(18) 148.8 3_446

_diffrn_measured_fraction_theta_max   0.994
_diffrn_reflns_theta_full   69.80
_diffrn_measured_fraction_theta_full   0.994
_refine_diff_density_max   0.234
_refine_diff_density_min   -0.452
_refine_diff_density_rms   0.064

data_compound_5

_audit_creation_method   SHELXL-97
_chemical_name_systematic
;

```
 ?
;
_chemical_name_common             ?
_chemical_melting_point           ?
_chemical_formula_moiety          ?
_chemical_formula_sum
 'C5 H12 Cl3 N2 Zn'
_chemical_formula_weight          271.89

loop_
 _atom_type_symbol
 _atom_type_description
 _atom_type_scat_dispersion_real
 _atom_type_scat_dispersion_imag
 _atom_type_scat_source
 'C'  'C'   0.0033   0.0016
 'International Tables Vol C Tables 4.2.6.8 and 6.1.1.4'
 'H'  'H'   0.0000   0.0000
 'International Tables Vol C Tables 4.2.6.8 and 6.1.1.4'
 'N'  'N'   0.0061   0.0033
 'International Tables Vol C Tables 4.2.6.8 and 6.1.1.4'
 'Cl'  'Cl'   0.1484   0.1585
 'International Tables Vol C Tables 4.2.6.8 and 6.1.1.4'
 'Zn'  'Zn'   0.2839   1.4301
 'International Tables Vol C Tables 4.2.6.8 and 6.1.1.4'

_symmetry_cell_setting            Monoclinic
_symmetry_space_group_name_H-M    P2(1)/n

loop_
 _symmetry_equiv_pos_as_xyz
 'x, y, z'
 '-x+1/2, y+1/2, -z+1/2'
 '-x, -y, -z'
 'x-1/2, -y-1/2, z-1/2'

_cell_length_a                    7.3936(11)
_cell_length_b                    17.204(3)
_cell_length_c                    8.4147(13)
_cell_angle_alpha                 90.00
_cell_angle_beta                  92.155(3)
_cell_angle_gamma                 90.00
_cell_volume                      1069.6(3)
_cell_formula_units_Z             4
```

```
_cell_measurement_temperature     173(2)
_cell_measurement_reflns_used     2043
_cell_measurement_theta_min       2.70
_cell_measurement_theta_max       28.79

_exptl_crystal_description        block
_exptl_crystal_colour             colourless
_exptl_crystal_size_max           0.22
_exptl_crystal_size_mid           0.17
_exptl_crystal_size_min           0.10
_exptl_crystal_density_meas       ?
_exptl_crystal_density_diffrn     1.688
_exptl_crystal_density_method     'not measured'
_exptl_crystal_F_000              548
_exptl_absorpt_coefficient_mu     2.991
_exptl_absorpt_correction_type    multi-scan
_exptl_absorpt_correction_T_min   0.5591
_exptl_absorpt_correction_T_max   0.7541
_exptl_absorpt_process_details    sadabs

_exptl_special_details
;
 ?
;

_diffrn_ambient_temperature       173(2)
_diffrn_radiation_wavelength      0.71073
_diffrn_radiation_type            MoK\a
_diffrn_radiation_source          'fine-focus sealed tube'
_diffrn_radiation_monochromator   graphite
_diffrn_measurement_device_type   'Bruker APEX-II CCD'
_diffrn_measurement_method        '\f and \w scans'
_diffrn_detector_area_resol_mean  ?
_diffrn_reflns_number             10387
_diffrn_reflns_av_R_equivalents   0.0463
_diffrn_reflns_av_sigmaI/netI     0.0525
_diffrn_reflns_limit_h_min        -10
_diffrn_reflns_limit_h_max        8
_diffrn_reflns_limit_k_min        -24
_diffrn_reflns_limit_k_max        24
_diffrn_reflns_limit_l_min        -12
_diffrn_reflns_limit_l_max        12
_diffrn_reflns_theta_min          2.37
_diffrn_reflns_theta_max          30.50
```

```
_reflns_number_total             3263
_reflns_number_gt                2408
_reflns_threshold_expression     >2sigma(I)

_computing_data_collection       'Bruker APEX2'
_computing_cell_refinement       'Bruker SAINT'
_computing_data_reduction        'Bruker SAINT'
_computing_structure_solution    'SHELXS-97 (Sheldrick, 2008)'
_computing_structure_refinement  'SHELXL-97 (Sheldrick, 2008)'
_computing_molecular_graphics    'Bruker SHELXTL'
_computing_publication_material  'Bruker SHELXTL'

_refine_special_details
;
 Refinement of F^2^ against ALL reflections.  The weighted R-factor wR and
 goodness of fit S are based on F^2^, conventional R-factors R are based
 on F, with F set to zero for negative F^2^. The threshold expression of
 F^2^ > 2sigma(F^2^) is used only for calculating R-factors(gt) etc. and is
 not relevant to the choice of reflections for refinement.  R-factors based
 on F^2^ are statistically about twice as large as those based on F, and R-
 factors based on ALL data will be even larger.
;

_refine_ls_structure_factor_coef  Fsqd
_refine_ls_matrix_type            full
_refine_ls_weighting_scheme       calc
_refine_ls_weighting_details
 'calc w=1/[\s^2^(Fo^2^)+(0.0234P)^2^+0.0000P] where P=(Fo^2^+2Fc^2^)/3'
_atom_sites_solution_primary      direct
_atom_sites_solution_secondary    difmap
_atom_sites_solution_hydrogens    geom
_refine_ls_hydrogen_treatment     mixed
_refine_ls_extinction_method      none
_refine_ls_extinction_coef        ?
_refine_ls_number_reflns          3263
_refine_ls_number_parameters      105
_refine_ls_number_restraints      0
_refine_ls_R_factor_all           0.0567
_refine_ls_R_factor_gt            0.0334
_refine_ls_wR_factor_ref          0.0694
_refine_ls_wR_factor_gt           0.0635
_refine_ls_goodness_of_fit_ref    1.019
_refine_ls_restrained_S_all       1.019
_refine_ls_shift/su_max           0.001
```

```
_refine_ls_shift/su_mean          0.000

loop_
 _atom_site_label
 _atom_site_type_symbol
 _atom_site_fract_x
 _atom_site_fract_y
 _atom_site_fract_z
 _atom_site_U_iso_or_equiv
 _atom_site_adp_type
 _atom_site_occupancy
 _atom_site_symmetry_multiplicity
 _atom_site_calc_flag
 _atom_site_refinement_flags
 _atom_site_disorder_assembly
 _atom_site_disorder_group
Zn1 Zn 0.53112(4) 0.122041(15) 0.35063(3) 0.01921(8) Uani 1 1 d . . .
Cl1 Cl 0.23183(9) 0.13191(4) 0.36822(8) 0.03379(15) Uani 1 1 d . . .
Cl2 Cl 0.61574(8) 0.01539(3) 0.21558(7) 0.02482(13) Uani 1 1 d . . .
Cl3 Cl 0.66408(8) 0.23285(3) 0.26807(7) 0.02649(13) Uani 1 1 d . . .
N1 N 0.6411(3) 0.10533(11) 0.5781(2) 0.0174(4) Uani 1 1 d . . .
H1N H 0.590(4) 0.0738(16) 0.611(3) 0.028(8) Uiso 1 1 d . . .
N2 N 0.8895(3) 0.13704(11) 0.8474(2) 0.0212(4) Uani 1 1 d . . .
C1 C 0.8345(3) 0.08650(16) 0.5745(3) 0.0284(6) Uani 1 1 d . . .
H1A H 0.8981 0.1297 0.5280 0.034 Uiso 1 1 calc R . .
H1B H 0.8502 0.0414 0.5072 0.034 Uiso 1 1 calc R . .
C2 C 0.9170(4) 0.06985(16) 0.7382(3) 0.0312(6) Uani 1 1 d . . .
H2A H 0.8617 0.0237 0.7813 0.037 Uiso 1 1 calc R . .
H2B H 1.0455 0.0600 0.7303 0.037 Uiso 1 1 calc R . .
C3 C 0.6923(3) 0.15492(14) 0.8542(3) 0.0242(5) Uani 1 1 d . . .
H3A H 0.6752 0.2000 0.9212 0.029 Uiso 1 1 calc R . .
H3B H 0.6298 0.1113 0.9004 0.029 Uiso 1 1 calc R . .
C4 C 0.6133(3) 0.17093(14) 0.6875(3) 0.0255(5) Uani 1 1 d . . .
H4A H 0.4846 0.1810 0.6933 0.031 Uiso 1 1 calc R . .
H4B H 0.6696 0.2172 0.6456 0.031 Uiso 1 1 calc R . .
C5 C 0.9722(4) 0.12183(15) 1.0090(3) 0.0289(5) Uani 1 1 d . . .
H5A H 0.9132 0.0782 1.0558 0.043 Uiso 1 1 calc R . .
H5B H 0.9580 0.1669 1.0747 0.043 Uiso 1 1 calc R . .
H5C H 1.0987 0.1106 1.0004 0.043 Uiso 1 1 calc R . .

loop_
 _atom_site_aniso_label
 _atom_site_aniso_U_11
 _atom_site_aniso_U_22
```

```
 _atom_site_aniso_U_33
 _atom_site_aniso_U_23
 _atom_site_aniso_U_13
 _atom_site_aniso_U_12
Zn1 0.01898(14) 0.02011(13) 0.01846(13) 0.00095(10) -0.00033(10) -0.00062(11)
Cl1 0.0184(3) 0.0481(4) 0.0348(3) -0.0014(3) 0.0004(2) 0.0013(3)
Cl2 0.0281(3) 0.0213(3) 0.0253(3) -0.0032(2) 0.0050(2) -0.0034(2)
Cl3 0.0291(3) 0.0190(3) 0.0320(3) 0.0023(2) 0.0101(2) 0.0006(2)
N1 0.0171(10) 0.0175(10) 0.0176(9) 0.0003(7) 0.0023(8) -0.0011(8)
N2 0.0212(10) 0.0244(10) 0.0176(9) 0.0001(7) -0.0029(8) -0.0033(8)
C1 0.0212(13) 0.0421(15) 0.0218(12) -0.0080(10) -0.0011(10) 0.0096(11)
C2 0.0259(14) 0.0386(15) 0.0286(13) -0.0087(11) -0.0055(11) 0.0150(12)
C3 0.0265(13) 0.0254(12) 0.0206(11) -0.0040(9) 0.0010(10) 0.0043(10)
C4 0.0264(13) 0.0274(13) 0.0226(12) -0.0032(9) -0.0006(10) 0.0109(11)
C5 0.0286(13) 0.0361(14) 0.0215(11) 0.0023(10) -0.0049(10) -0.0043(12)

_geom_special_details
;
 All esds (except the esd in the dihedral angle between two l.s. planes)
 are estimated using the full covariance matrix.  The cell esds are taken
 into account individually in the estimation of esds in distances, angles
 and torsion angles; correlations between esds in cell parameters are only
 used when they are defined by crystal symmetry.  An approximate (isotropic)
 treatment of cell esds is used for estimating esds involving l.s. planes.
;

loop_
 _geom_bond_atom_site_label_1
 _geom_bond_atom_site_label_2
 _geom_bond_distance
 _geom_bond_site_symmetry_2
 _geom_bond_publ_flag
Zn1 N1 2.0718(19) . ?
Zn1 Cl1 2.2298(8) . ?
Zn1 Cl2 2.2590(7) . ?
Zn1 Cl3 2.2661(7) . ?
N1 C1 1.467(3) . ?
N1 C4 1.476(3) . ?
N1 H1N 0.72(3) . ?
N2 C3 1.493(3) . ?
N2 C5 1.493(3) . ?
N2 C2 1.495(3) . ?
C1 C2 1.513(3) . ?
C1 H1A 0.9700 . ?
```

```
C1 H1B 0.9700 . ?
C2 H2A 0.9700 . ?
C2 H2B 0.9700 . ?
C3 C4 1.524(3) . ?
C3 H3A 0.9700 . ?
C3 H3B 0.9700 . ?
C4 H4A 0.9700 . ?
C4 H4B 0.9700 . ?
C5 H5A 0.9600 . ?
C5 H5B 0.9600 . ?
C5 H5C 0.9600 . ?

loop_
 _geom_angle_atom_site_label_1
 _geom_angle_atom_site_label_2
 _geom_angle_atom_site_label_3
 _geom_angle
 _geom_angle_site_symmetry_1
 _geom_angle_site_symmetry_3
 _geom_angle_publ_flag
N1 Zn1 Cl1 107.78(6) . . ?
N1 Zn1 Cl2 104.22(6) . . ?
Cl1 Zn1 Cl2 112.93(3) . . ?
N1 Zn1 Cl3 103.90(6) . . ?
Cl1 Zn1 Cl3 113.54(3) . . ?
Cl2 Zn1 Cl3 113.42(3) . . ?
C1 N1 C4 109.93(19) . . ?
C1 N1 Zn1 111.12(15) . . ?
C4 N1 Zn1 114.27(14) . . ?
C1 N1 H1N 111(2) . . ?
C4 N1 H1N 104(2) . . ?
Zn1 N1 H1N 105(2) . . ?
C3 N2 C5 111.58(19) . . ?
C3 N2 C2 109.81(18) . . ?
C5 N2 C2 111.31(18) . . ?
N1 C1 C2 112.5(2) . . ?
N1 C1 H1A 109.1 . . ?
C2 C1 H1A 109.1 . . ?
N1 C1 H1B 109.1 . . ?
C2 C1 H1B 109.1 . . ?
H1A C1 H1B 107.8 . . ?
N2 C2 C1 110.7(2) . . ?
N2 C2 H2A 109.5 . . ?
C1 C2 H2A 109.5 . . ?
```

```
N2 C2 H2B 109.5 . . ?
C1 C2 H2B 109.5 . . ?
H2A C2 H2B 108.1 . . ?
N2 C3 C4 110.0(2) . . ?
N2 C3 H3A 109.7 . . ?
C4 C3 H3A 109.7 . . ?
N2 C3 H3B 109.7 . . ?
C4 C3 H3B 109.7 . . ?
H3A C3 H3B 108.2 . . ?
N1 C4 C3 112.22(19) . . ?
N1 C4 H4A 109.2 . . ?
C3 C4 H4A 109.2 . . ?
N1 C4 H4B 109.2 . . ?
C3 C4 H4B 109.2 . . ?
H4A C4 H4B 107.9 . . ?
N2 C5 H5A 109.5 . . ?
N2 C5 H5B 109.5 . . ?
H5A C5 H5B 109.5 . . ?
N2 C5 H5C 109.5 . . ?
H5A C5 H5C 109.5 . . ?
H5B C5 H5C 109.5 . . ?

loop_
 _geom_torsion_atom_site_label_1
 _geom_torsion_atom_site_label_2
 _geom_torsion_atom_site_label_3
 _geom_torsion_atom_site_label_4
 _geom_torsion
 _geom_torsion_site_symmetry_1
 _geom_torsion_site_symmetry_2
 _geom_torsion_site_symmetry_3
 _geom_torsion_site_symmetry_4
 _geom_torsion_publ_flag
Cl1 Zn1 N1 C1 171.40(15) . . . . ?
Cl2 Zn1 N1 C1 51.18(17) . . . . ?
Cl3 Zn1 N1 C1 -67.84(16) . . . . ?
Cl1 Zn1 N1 C4 -63.51(17) . . . . ?
Cl2 Zn1 N1 C4 176.28(16) . . . . ?
Cl3 Zn1 N1 C4 57.25(18) . . . . ?
C4 N1 C1 C2 55.5(3) . . . . ?
Zn1 N1 C1 C2 -177.05(17) . . . . ?
C3 N2 C2 C1 56.6(3) . . . . ?
C5 N2 C2 C1 -179.3(2) . . . . ?
N1 C1 C2 N2 -56.7(3) . . . . ?
```

```
C5 N2 C3 C4 179.39(19) . . . . ?
C2 N2 C3 C4 -56.7(3) . . . . ?
C1 N1 C4 C3 -55.8(3) . . . . ?
Zn1 N1 C4 C3 178.50(17) . . . . ?
N2 C3 C4 N1 57.2(3) . . . . ?

_diffrn_measured_fraction_theta_max    0.998
_diffrn_reflns_theta_full              30.50
_diffrn_measured_fraction_theta_full   0.998
_refine_diff_density_max    0.909
_refine_diff_density_min   -0.380
_refine_diff_density_rms    0.094

data_compound_6

_audit_creation_method            SHELXL-97
_chemical_name_systematic
;
 ?
;
_chemical_name_common             ?
_chemical_melting_point           ?
_chemical_formula_moiety          ?
_chemical_formula_sum
 'C26 H36 Cl2 N4 O2 Zn'
_chemical_formula_weight          572.86
_chemical_absolute_configuration  'ad'

loop_
 _atom_type_symbol
 _atom_type_description
 _atom_type_scat_dispersion_real
 _atom_type_scat_dispersion_imag
 _atom_type_scat_source
 'C'  'C'   0.0181   0.0091
 'International Tables Vol C Tables 4.2.6.8 and 6.1.1.4'
 'H'  'H'   0.0000   0.0000
 'International Tables Vol C Tables 4.2.6.8 and 6.1.1.4'
 'N'  'N'   0.0311   0.0180
 'International Tables Vol C Tables 4.2.6.8 and 6.1.1.4'
 'O'  'O'   0.0492   0.0322
 'International Tables Vol C Tables 4.2.6.8 and 6.1.1.4'
 'Cl'  'Cl'   0.3639   0.7018
 'International Tables Vol C Tables 4.2.6.8 and 6.1.1.4'
```

```
 'Zn'  'Zn'  -1.5491   0.6778
 'International Tables Vol C Tables 4.2.6.8 and 6.1.1.4'

_symmetry_cell_setting            tetragonal
_symmetry_space_group_name_H-M    'P 41 21 2'

loop_
 _symmetry_equiv_pos_as_xyz
 'x, y, z'
 '-x, -y, z+1/2'
 'x+1/2, -y+1/2, -z+3/4'
 '-x+1/2, y+1/2, -z+1/4'
 '-y, -x, -z+1/2'
 'y, x, -z'
 'y+1/2, -x+1/2, z+3/4'
 '-y+1/2, x+1/2, z+1/4'

_cell_length_a                    11.30570(10)
_cell_length_b                    11.30570(10)
_cell_length_c                    23.1376(5)
_cell_angle_alpha                 90.00
_cell_angle_beta                  90.00
_cell_angle_gamma                 90.00
_cell_volume                      2957.42(7)
_cell_formula_units_Z             4
_cell_measurement_temperature     291(2)
_cell_measurement_reflns_used   12560
_cell_measurement_theta_min     3.8177
_cell_measurement_theta_max     69.7257

_exptl_crystal_description        ?
_exptl_crystal_colour             ?
_exptl_crystal_size_max           0.42
_exptl_crystal_size_mid           0.38
_exptl_crystal_size_min           0.35
_exptl_crystal_density_meas       ?
_exptl_crystal_density_diffrn     1.287
_exptl_crystal_density_method     'not measured'
_exptl_crystal_F_000              1200
_exptl_absorpt_coefficient_mu     3.034
_exptl_absorpt_correction_type    'multi-scan'
_exptl_absorpt_correction_T_min   0.3622
_exptl_absorpt_correction_T_max   0.4165
_exptl_absorpt_process_details
```

```
;
CrysAlisPro, Oxford Diffraction Ltd.,
Version 1.171.34.44 (release 25-10-2010 CrysAlis171 .NET)
(compiled Oct 25 2010,18:11:34)
Empirical absorption correction using spherical harmonics,
 implemented in SCALE3 ABSPACK scaling algorithm.
;

_exptl_special_details
;
 ?
;

_diffrn_ambient_temperature       291(2)
_diffrn_radiation_wavelength      1.54184
_diffrn_radiation_type            CuK\a
_diffrn_radiation_source          'Enhance Ultra (Cu) X-ray Source'
_diffrn_radiation_monochromator   'mirror'
_diffrn_measurement_device_type   'Gemini S Ultra, Oxford Diffraction'
_diffrn_measurement_method        '\w scans'
_diffrn_detector_area_resol_mean  15.9149
_diffrn_standards_number          ?
_diffrn_standards_interval_count  ?
_diffrn_standards_interval_time   ?
_diffrn_standards_decay_%         ?
_diffrn_reflns_number             20763
_diffrn_reflns_av_R_equivalents   0.0283
_diffrn_reflns_av_sigmaI/netI     0.0137
_diffrn_reflns_limit_h_min        -13
_diffrn_reflns_limit_h_max        12
_diffrn_reflns_limit_k_min        -13
_diffrn_reflns_limit_k_max        11
_diffrn_reflns_limit_l_min        -27
_diffrn_reflns_limit_l_max        28
_diffrn_reflns_theta_min          4.35
_diffrn_reflns_theta_max          69.85
_reflns_number_total              2786
_reflns_number_gt                 2569
_reflns_threshold_expression      >2sigma(I)

_computing_data_collection        'CrysAlisPro (Oxford Diffraction Ltd.)'
_computing_cell_refinement        'CrysAlisPro (Oxford Diffraction Ltd.)'
_computing_data_reduction         'CrysAlisPro (Oxford Diffraction Ltd.)'
_computing_structure_solution     'SHELXS-97 (Sheldrick, 1997)'
```

```
_computing_structure_refinement   'SHELXL-97 (Sheldrick, 1997)'
_computing_molecular_graphics     'Ortep-3 (L. J. Farrugia, 2001)'
_computing_publication_material   SHELXL-97

_refine_special_details
;
 Refinement of F^2^ against ALL reflections.  The weighted R-factor wR and
 goodness of fit S are based on F^2^, conventional R-factors R are based
 on F, with F set to zero for negative F^2^. The threshold expression of
 F^2^ > 2sigma(F^2^) is used only for calculating R-factors(gt) etc. and is
 not relevant to the choice of reflections for refinement.  R-factors based
 on F^2^ are statistically about twice as large as those based on F, and R-
 factors based on ALL data will be even larger.
;

_refine_ls_structure_factor_coef  Fsqd
_refine_ls_matrix_type            full
_refine_ls_weighting_scheme       calc
_refine_ls_weighting_details
 'calc w=1/[\s^2^(Fo^2^)+(0.0953P)^2^+0.9361P] where P=(Fo^2^+2Fc^2^)/3'
_atom_sites_solution_primary      direct
_atom_sites_solution_secondary    difmap
_atom_sites_solution_hydrogens    geom
_refine_ls_hydrogen_treatment     mixed
_refine_ls_extinction_method      SHELXL
_refine_ls_extinction_coef        0.0049(8)
_refine_ls_extinction_expression
 'Fc^*^=kFc[1+0.001xFc^2^\l^3^/sin(2\q)]^-1/4^'
_refine_ls_abs_structure_details
 'Flack H D (1983), Acta Cryst. A39, 876-881'
_refine_ls_abs_structure_Flack    0.00(5)
_refine_ls_number_reflns          2786
_refine_ls_number_parameters      168
_refine_ls_number_restraints      25
_refine_ls_R_factor_all           0.0558
_refine_ls_R_factor_gt            0.0526
_refine_ls_wR_factor_ref          0.1602
_refine_ls_wR_factor_gt           0.1555
_refine_ls_goodness_of_fit_ref    1.056
_refine_ls_restrained_S_all       1.149
_refine_ls_shift/su_max           0.000
_refine_ls_shift/su_mean          0.000

loop_
```

```
 _atom_site_label
 _atom_site_type_symbol
 _atom_site_fract_x
 _atom_site_fract_y
 _atom_site_fract_z
 _atom_site_U_iso_or_equiv
 _atom_site_adp_type
 _atom_site_occupancy
 _atom_site_symmetry_multiplicity
 _atom_site_calc_flag
 _atom_site_refinement_flags
 _atom_site_disorder_assembly
 _atom_site_disorder_group
Zn1 Zn 0.11935(4) 0.11935(4) 1.0000 0.0888(4) Uani 1 2 d S . .
Cl1 Cl 0.24051(15) 0.16220(18) 1.07411(12) 0.1626(10) Uani 1 1 d . . .
O1 O 0.0805(3) -0.2472(2) 0.97545(13) 0.0816(8) Uani 1 1 d . . .
N2 N 0.1372(3) -0.0595(3) 0.98919(14) 0.0689(7) Uani 1 1 d . . .
C9 C 0.2219(4) -0.1311(4) 1.0232(2) 0.0788(10) Uani 1 1 d . . .
H9 H 0.2267 -0.1012 1.0629 0.095 Uiso 1 1 calc R . .
C10 C 0.3428(4) -0.1276(5) 0.9949(2) 0.0968(12) Uani 1 1 d U . .
H10A H 0.3655 -0.0455 0.9896 0.116 Uiso 1 1 calc R . .
H10B H 0.3368 -0.1632 0.9569 0.116 Uiso 1 1 calc R . .
C2 C 0.0010(4) -0.0039(5) 0.88395(18) 0.0914(11) Uani 1 1 d . . .
H2 H 0.0739 0.0339 0.8863 0.110 Uiso 1 1 calc R . .
C3 C -0.0206(3) -0.1010(3) 0.91907(16) 0.0707(9) Uani 1 1 d . . .
C6 C -0.1864(6) -0.0227(6) 0.8414(2) 0.1091(18) Uani 1 1 d . . .
H6 H -0.2433 0.0029 0.8152 0.131 Uiso 1 1 calc R . .
C5 C -0.2088(5) -0.1225(5) 0.8754(2) 0.0999(15) Uani 1 1 d . . .
H5 H -0.2800 -0.1630 0.8712 0.120 Uiso 1 1 calc R . .
C4 C -0.1258(4) -0.1618(4) 0.9154(2) 0.0852(11) Uani 1 1 d . . .
H4 H -0.1409 -0.2270 0.9388 0.102 Uiso 1 1 calc R . .
C8 C 0.1635(4) -0.2532(4) 1.0225(2) 0.0855(11) Uani 1 1 d . . .
H8B H 0.2217 -0.3148 1.0159 0.103 Uiso 1 1 calc R . .
H8A H 0.1232 -0.2687 1.0588 0.103 Uiso 1 1 calc R . .
C7 C 0.0687(3) -0.1320(3) 0.96247(16) 0.0672(8) Uani 1 1 d . . .
N1 N -0.0591(8) 0.1450(8) 0.8151(3) 0.155(3) Uani 1 1 d D . .
C11 C 0.4409(6) -0.1907(7) 1.0286(3) 0.1181(16) Uani 1 1 d U . .
H11 H 0.4121 -0.2682 1.0415 0.142 Uiso 1 1 calc R . .
C1 C -0.0818(6) 0.0379(6) 0.8461(2) 0.1044(16) Uani 1 1 d D . .
C12 C 0.4777(8) -0.1181(8) 1.0810(4) 0.152(3) Uani 1 1 d U . .
H12A H 0.5032 -0.0412 1.0686 0.228 Uiso 1 1 calc R . .
H12B H 0.5414 -0.1572 1.1006 0.228 Uiso 1 1 calc R . .
H12C H 0.4116 -0.1100 1.1068 0.228 Uiso 1 1 calc R . .
C13 C 0.5471(6) -0.2080(9) 0.9880(4) 0.153(2) Uani 1 1 d U . .
```

```
H13A H 0.5215 -0.2468 0.9533 0.229 Uiso 1 1 calc R . .
H13B H 0.6059 -0.2557 1.0068 0.229 Uiso 1 1 calc R . .
H13C H 0.5804 -0.1324 0.9785 0.229 Uiso 1 1 calc R . .
H1A H -0.007(5) 0.106(6) 0.793(3) 0.184 Uiso 1 1 d D . .
H1B H -0.124(4) 0.137(7) 0.796(3) 0.184 Uiso 1 1 d D . .

loop_
 _atom_site_aniso_label
 _atom_site_aniso_U_11
 _atom_site_aniso_U_22
 _atom_site_aniso_U_33
 _atom_site_aniso_U_23
 _atom_site_aniso_U_13
 _atom_site_aniso_U_12
Zn1 0.0588(3) 0.0588(3) 0.1490(8) -0.0082(3) 0.0082(3) -0.0095(3)
Cl1 0.0948(10) 0.1297(13) 0.263(2) -0.0855(15) -0.0453(13) -0.0208(8)
O1 0.0808(17) 0.0579(14) 0.1062(18) -0.0043(13) -0.0096(15) -0.0005(12)
N2 0.0530(16) 0.0612(16) 0.0925(19) -0.0052(14) -0.0014(14) 0.0009(12)
C9 0.059(2) 0.080(3) 0.097(2) 0.000(2) -0.0080(18) 0.0048(19)
C10 0.076(2) 0.096(3) 0.118(3) -0.007(3) 0.001(2) -0.009(2)
C2 0.084(3) 0.106(4) 0.084(2) 0.015(2) 0.009(2) -0.004(2)
C3 0.064(2) 0.070(2) 0.0781(19) -0.0082(17) 0.0021(16) -0.0014(16)
C6 0.122(4) 0.123(4) 0.082(3) -0.006(3) -0.023(3) 0.015(4)
C5 0.092(3) 0.100(4) 0.108(3) -0.026(3) -0.024(3) -0.007(3)
C4 0.082(3) 0.078(2) 0.096(2) -0.011(2) -0.016(2) -0.010(2)
C8 0.076(2) 0.074(2) 0.106(3) 0.009(2) -0.006(2) 0.006(2)
C7 0.0583(18) 0.0590(19) 0.084(2) -0.0073(16) 0.0034(15) 0.0026(15)
N1 0.173(6) 0.173(6) 0.120(4) 0.058(4) 0.005(4) 0.009(5)
C11 0.108(2) 0.119(2) 0.127(2) 0.0031(19) -0.0089(16) 0.0060(18)
C1 0.109(4) 0.120(4) 0.084(3) 0.020(3) 0.003(3) 0.001(3)
C12 0.128(4) 0.176(5) 0.153(4) 0.002(4) -0.019(3) 0.005(4)
C13 0.101(3) 0.173(4) 0.185(4) -0.019(4) 0.000(3) 0.033(3)

_geom_special_details
;
 All esds (except the esd in the dihedral angle between two l.s. planes)
 are estimated using the full covariance matrix.  The cell esds are taken
 into account individually in the estimation of esds in distances, angles
 and torsion angles; correlations between esds in cell parameters are only
 used when they are defined by crystal symmetry.  An approximate (isotropic)
 treatment of cell esds is used for estimating esds involving l.s. planes.
;

loop_
```

```
 _geom_bond_atom_site_label_1
 _geom_bond_atom_site_label_2
 _geom_bond_distance
 _geom_bond_site_symmetry_2
 _geom_bond_publ_flag
Zn1 N2 2.047(3) . ?
Zn1 N2 2.047(3) 6_557 ?
Zn1 Cl1 2.2475(19) . ?
Zn1 Cl1 2.2475(19) 6_557 ?
O1 C7 1.344(5) . ?
O1 C8 1.440(5) . ?
N2 C7 1.286(5) . ?
N2 C9 1.481(5) . ?
C9 C10 1.516(6) . ?
C9 C8 1.530(7) . ?
C10 C11 1.532(8) . ?
C2 C1 1.365(7) . ?
C2 C3 1.388(6) . ?
C3 C4 1.376(6) . ?
C3 C7 1.467(5) . ?
C6 C1 1.372(9) . ?
C6 C5 1.398(9) . ?
C5 C4 1.391(7) . ?
N1 C1 1.430(9) . ?
C11 C12 1.521(10) . ?
C11 C13 1.538(10) . ?

loop_
 _geom_angle_atom_site_label_1
 _geom_angle_atom_site_label_2
 _geom_angle_atom_site_label_3
 _geom_angle
 _geom_angle_site_symmetry_1
 _geom_angle_site_symmetry_3
 _geom_angle_publ_flag
N2 Zn1 N2 102.10(17) . 6_557 ?
N2 Zn1 Cl1 104.24(10) . . ?
N2 Zn1 Cl1 119.17(11) 6_557 . ?
N2 Zn1 Cl1 119.17(11) . 6_557 ?
N2 Zn1 Cl1 104.24(10) 6_557 6_557 ?
Cl1 Zn1 Cl1 108.63(14) . 6_557 ?
C7 O1 C8 106.2(3) . . ?
C7 N2 C9 107.2(3) . . ?
C7 N2 Zn1 129.0(3) . . ?
```

C9 N2 Zn1 122.6(3) . . ?
N2 C9 C10 109.8(4) . . ?
N2 C9 C8 102.1(3) . . ?
C10 C9 C8 114.1(4) . . ?
C9 C10 C11 114.8(5) . . ?
C1 C2 C3 121.9(5) . . ?
C4 C3 C2 120.7(4) . . ?
C4 C3 C7 121.3(4) . . ?
C2 C3 C7 117.9(4) . . ?
C1 C6 C5 121.0(5) . . ?
C4 C5 C6 120.6(5) . . ?
C3 C4 C5 117.7(5) . . ?
O1 C8 C9 104.3(3) . . ?
N2 C7 O1 116.8(3) . . ?
N2 C7 C3 126.3(4) . . ?
O1 C7 C3 116.9(3) . . ?
C12 C11 C10 110.6(6) . . ?
C12 C11 C13 110.0(7) . . ?
C10 C11 C13 108.3(6) . . ?
C2 C1 C6 118.0(5) . . ?
C2 C1 N1 119.4(6) . . ?
C6 C1 N1 122.5(6) . . ?

_diffrn_measured_fraction_theta_max   0.995
_diffrn_reflns_theta_full   69.85
_diffrn_measured_fraction_theta_full   0.995
_refine_diff_density_max   0.291
_refine_diff_density_min   -0.328
_refine_diff_density_rms   0.044

data_compound_7

_audit_creation_method   SHELXL-2013
_chemical_name_systematic
;
 ?
;
_chemical_name_common   ?
_chemical_melting_point   ?
_chemical_formula_moiety   ?
_chemical_formula_sum
 'C30 H30 Cl2 N4 O3 Zn'
_chemical_formula_weight   630.85

```
loop_
 _atom_type_symbol
 _atom_type_description
 _atom_type_scat_dispersion_real
 _atom_type_scat_dispersion_imag
 _atom_type_scat_source
 'C'  'C'   0.0033   0.0016
 'International Tables Vol C Tables 4.2.6.8 and 6.1.1.4'
 'H'  'H'   0.0000   0.0000
 'International Tables Vol C Tables 4.2.6.8 and 6.1.1.4'
 'N'  'N'   0.0061   0.0033
 'International Tables Vol C Tables 4.2.6.8 and 6.1.1.4'
 'O'  'O'   0.0106   0.0060
 'International Tables Vol C Tables 4.2.6.8 and 6.1.1.4'
 'Cl'  'Cl'   0.1484   0.1585
 'International Tables Vol C Tables 4.2.6.8 and 6.1.1.4'
 'Zn'  'Zn'   0.2839   1.4301
 'International Tables Vol C Tables 4.2.6.8 and 6.1.1.4'

_space_group_crystal_system       monoclinic
_space_group_IT_number            5
_space_group_name_H-M_alt         'C 2'
_space_group_name_Hall            'C 2y'

_shelx_space_group_comment
;
The symmetry employed for this shelxl refinement is uniquely defined
by the following loop, which should always be used as a source of
symmetry information in preference to the above space-group names.
They are only intended as comments.
;

loop_
 _space_group_symop_operation_xyz
 'x, y, z'
 '-x, y, -z'
 'x+1/2, y+1/2, z'
 '-x+1/2, y+1/2, -z'

_cell_length_a                    14.361(2)
_cell_length_b                    10.2938(14)
_cell_length_c                    9.4877(14)
_cell_angle_alpha                 90
_cell_angle_beta                  93.739(4)
```

```
_cell_angle_gamma                 90
_cell_volume                      1399.6(4)
_cell_formula_units_Z             2
_cell_measurement_temperature     293(2)
_cell_measurement_reflns_used     807
_cell_measurement_theta_min       4.302
_cell_measurement_theta_max       39.404

_exptl_crystal_description        prismatic
_exptl_crystal_colour             colorless
_exptl_crystal_density_meas       ?
_exptl_crystal_density_method     ?
_exptl_crystal_density_diffrn     1.497
_exptl_crystal_F_000              652
_exptl_transmission_factor_min    ?
_exptl_transmission_factor_max    ?
_exptl_crystal_size_max           0.200
_exptl_crystal_size_mid           0.160
_exptl_crystal_size_min           0.120
_exptl_absorpt_coefficient_mu     1.108
_shelx_estimated_absorpt_T_min    0.809
_shelx_estimated_absorpt_T_max    0.878
_exptl_absorpt_correction_type    multi-scan
_exptl_absorpt_correction_T_min   0.6320
_exptl_absorpt_correction_T_max   0.7456
_exptl_absorpt_process_details    sadabs

_exptl_special_details
;
 ?
;

_diffrn_ambient_temperature       293(2)
_diffrn_radiation_wavelength      0.71073
_diffrn_radiation_type            MoK\a
_diffrn_source                    ?
_diffrn_measurement_device_type   'CCD area detector'
_diffrn_measurement_method        'phi and omega scans'
_diffrn_detector_area_resol_mean  ?
_diffrn_reflns_number             4216
_diffrn_reflns_av_unetI/netI      0.0780
_diffrn_reflns_av_R_equivalents   0.0393
_diffrn_reflns_limit_h_min        -17
_diffrn_reflns_limit_h_max        10
```

```
_diffrn_reflns_limit_k_min        -12
_diffrn_reflns_limit_k_max        10
_diffrn_reflns_limit_l_min        -10
_diffrn_reflns_limit_l_max        11
_diffrn_reflns_theta_min          2.151
_diffrn_reflns_theta_max          25.997
_diffrn_reflns_theta_full         25.242
_diffrn_measured_fraction_theta_max   1.000
_diffrn_measured_fraction_theta_full  1.000
_diffrn_reflns_Laue_measured_fraction_max    1.000
_diffrn_reflns_Laue_measured_fraction_full   1.000
_diffrn_reflns_point_group_measured_fraction_max   0.943
_diffrn_reflns_point_group_measured_fraction_full  0.949
_reflns_number_total              2600
_reflns_number_gt                 2078
_reflns_threshold_expression      'I > 2\s(I)'
_reflns_Friedel_coverage          0.782
_reflns_Friedel_fraction_max      0.880
_reflns_Friedel_fraction_full     0.891

_reflns_special_details
;
 Reflections were merged by SHELXL according to the crystal
 class for the calculation of statistics and refinement.

 _reflns_Friedel_fraction is defined as the number of unique
 Friedel pairs measured divided by the number that would be
 possible theoretically, ignoring centric projections and
 systematic absences.
;

_computing_data_collection        'Bruker SMART'
_computing_cell_refinement        'Bruker SMART'
_computing_data_reduction         'Bruker SHELXTL'
_computing_structure_solution     'Bruker SHELXTL'
_computing_structure_refinement   'SHELXL-2013 (Sheldrick, 2013)'
_computing_molecular_graphics     'Bruker SHELXTL'
_computing_publication_material   'Bruker SHELXTL'

_refine_special_details
;
 ?
;
_refine_ls_structure_factor_coef  Fsqd
```

```
_refine_ls_matrix_type           full
_refine_ls_weighting_scheme      calc
_refine_ls_weighting_details
'w=1/[\s^2^(Fo^2^)+(0.0598P)^2^] where P=(Fo^2^+2Fc^2^)/3'
_atom_sites_solution_primary     ?
_atom_sites_solution_secondary   ?
_atom_sites_solution_hydrogens   mixed
_refine_ls_hydrogen_treatment    mixed
_refine_ls_extinction_method     none
_refine_ls_extinction_coef       .
_refine_ls_abs_structure_details
;
 Flack x determined using 740 quotients [(I+)-(I-)]/[(I+)+(I-)]
 (Parsons and Flack (2004), Acta Cryst. A60, s61).
;
_refine_ls_abs_structure_Flack    0.022(14)
_chemical_absolute_configuration  ad
_refine_ls_number_reflns          2600
_refine_ls_number_parameters      186
_refine_ls_number_restraints      1
_refine_ls_R_factor_all           0.0601
_refine_ls_R_factor_gt            0.0468
_refine_ls_wR_factor_ref          0.1217
_refine_ls_wR_factor_gt           0.0974
_refine_ls_goodness_of_fit_ref    0.975
_refine_ls_restrained_S_all       0.975
_refine_ls_shift/su_max           0.000
_refine_ls_shift/su_mean          0.000

loop_
 _atom_site_label
 _atom_site_type_symbol
 _atom_site_fract_x
 _atom_site_fract_y
 _atom_site_fract_z
 _atom_site_U_iso_or_equiv
 _atom_site_adp_type
 _atom_site_occupancy
 _atom_site_site_symmetry_order
 _atom_site_calc_flag
 _atom_site_refinement_flags_posn
 _atom_site_refinement_flags_adp
 _atom_site_refinement_flags_occupancy
 _atom_site_disorder_assembly
```

_atom_site_disorder_group
Zn1 Zn 1.0000 1.03446(8) 0.5000 0.0370(4) Uani 1 2 d S T P . .
Cl1 Cl 1.00658(17) 1.18743(19) 0.6893(2) 0.0496(6) Uani 1 1 d . . . . .
N1 N 0.8484(4) 0.9883(5) 0.5237(6) 0.0372(15) Uani 1 1 d . . . . .
N2 N 0.9627(5) 0.8803(6) 0.3443(6) 0.0367(15) Uani 1 1 d . . . . .
O1 O 0.8453(4) 0.7784(5) 0.2250(5) 0.0432(13) Uani 1 1 d . . . . .
O2 O 1.0000 0.0457(12) 0.0000 0.088(3) Uani 1 2 d S T P . .
C1 C 0.7942(5) 1.0269(9) 0.6226(7) 0.0455(17) Uani 1 1 d . . . . .
H1 H 0.8199 1.0807 0.6936 0.055 Uiso 1 1 calc R U . . .
C2 C 0.7017(6) 0.9926(8) 0.6271(9) 0.053(2) Uani 1 1 d . . . . .
H2 H 0.6665 1.0240 0.6985 0.064 Uiso 1 1 calc R U . . .
C3 C 0.6625(6) 0.9122(8) 0.5259(10) 0.052(2) Uani 1 1 d . . . . .
H3 H 0.6003 0.8874 0.5269 0.063 Uiso 1 1 calc R U . . .
C4 C 0.7175(6) 0.8687(7) 0.4220(8) 0.0425(19) Uani 1 1 d . . . . .
H4 H 0.6929 0.8145 0.3505 0.051 Uiso 1 1 calc R U . . .
C5 C 0.8094(5) 0.9068(6) 0.4258(7) 0.0330(16) Uani 1 1 d . . . . .
C6 C 0.8764(6) 0.8577(7) 0.3291(7) 0.0369(17) Uani 1 1 d . . . . .
C7 C 0.9254(6) 0.7617(8) 0.1411(8) 0.044(2) Uani 1 1 d . . . . .
H7A H 0.9204 0.8165 0.0579 0.053 Uiso 1 1 calc R U . . .
H7B H 0.9314 0.6719 0.1119 0.053 Uiso 1 1 calc R U . . .
C8 C 1.0075(5) 0.8018(7) 0.2394(7) 0.0360(17) Uani 1 1 d . . . . .
H8 H 1.0511 0.8549 0.1888 0.043 Uiso 1 1 calc R U . . .
C9 C 1.0595(6) 0.6871(8) 0.3148(8) 0.045(2) Uani 1 1 d . . . . .
H9A H 1.0999 0.7202 0.3926 0.054 Uiso 1 1 calc R U . . .
H9B H 1.0145 0.6290 0.3534 0.054 Uiso 1 1 calc R U . . .
C10 C 1.1174(6) 0.6125(7) 0.2146(8) 0.0398(19) Uani 1 1 d . . . . .
C11 C 1.0850(6) 0.4992(7) 0.1510(9) 0.051(2) Uani 1 1 d . . . . .
H11 H 1.0270 0.4667 0.1717 0.061 Uiso 1 1 calc R U . . .
C12 C 1.1376(8) 0.4329(9) 0.0566(10) 0.069(3) Uani 1 1 d . . . . .
H12 H 1.1147 0.3576 0.0128 0.083 Uiso 1 1 calc R U . . .
C13 C 1.2229(8) 0.4797(11) 0.0291(10) 0.069(3) Uani 1 1 d . . . . .
H13 H 1.2580 0.4369 -0.0355 0.083 Uiso 1 1 calc R U . . .
C14 C 1.2583(8) 0.5901(11) 0.0959(11) 0.071(3) Uani 1 1 d . . . . .
H14 H 1.3178 0.6196 0.0791 0.086 Uiso 1 1 calc R U . . .
C15 C 1.2058(6) 0.6550(8) 0.1860(9) 0.051(2) Uani 1 1 d . . . . .
H15 H 1.2295 0.7299 0.2298 0.061 Uiso 1 1 calc R U . . .
H2A H 0.980(8) 0.090(10) 0.060(10) 0.09(4) Uiso 1 1 d . . . . .

loop_
_atom_site_aniso_label
_atom_site_aniso_U_11
_atom_site_aniso_U_22
_atom_site_aniso_U_33
_atom_site_aniso_U_23

```
 _atom_site_aniso_U_13
 _atom_site_aniso_U_12
Zn1 0.0369(7) 0.0367(7) 0.0378(7) 0.000 0.0044(5) 0.000
Cl1 0.0582(16) 0.0463(13) 0.0444(13) -0.0059(10) 0.0030(11) -0.0002(11)
N1 0.036(4) 0.037(4) 0.039(4) 0.003(3) 0.004(3) 0.004(3)
N2 0.040(4) 0.035(3) 0.035(4) -0.003(3) 0.005(3) -0.003(3)
O1 0.044(3) 0.047(3) 0.037(3) -0.008(2) -0.006(3) -0.001(3)
O2 0.139(10) 0.066(7) 0.063(6) 0.000 0.042(6) 0.000
C1 0.050(4) 0.040(4) 0.047(4) 0.000(5) 0.006(3) 0.002(5)
C2 0.050(5) 0.057(6) 0.055(5) 0.004(4) 0.022(4) 0.013(4)
C3 0.036(5) 0.056(6) 0.065(6) 0.007(5) 0.008(4) -0.001(4)
C4 0.042(5) 0.032(4) 0.052(5) 0.001(4) -0.010(4) -0.001(4)
C5 0.035(4) 0.030(4) 0.034(4) 0.007(3) 0.002(3) 0.004(3)
C6 0.047(5) 0.029(4) 0.034(4) 0.005(3) -0.004(3) 0.003(4)
C7 0.062(6) 0.040(4) 0.032(4) 0.002(3) 0.006(4) 0.004(4)
C8 0.044(5) 0.029(4) 0.035(4) -0.006(3) 0.003(3) 0.000(3)
C9 0.049(5) 0.051(5) 0.035(4) -0.004(4) 0.003(4) 0.004(4)
C10 0.052(5) 0.032(4) 0.035(4) 0.006(3) -0.006(4) 0.011(4)
C11 0.056(5) 0.039(6) 0.055(5) 0.002(4) -0.006(4) 0.001(4)
C12 0.088(8) 0.058(6) 0.058(6) -0.022(5) -0.021(6) 0.031(6)
C13 0.083(9) 0.081(7) 0.045(6) 0.005(5) 0.012(6) 0.050(7)
C14 0.054(6) 0.089(8) 0.072(7) 0.019(6) 0.012(6) 0.018(6)
C15 0.046(5) 0.045(5) 0.062(6) 0.000(4) 0.004(5) 0.000(4)

_geom_special_details
;
 All esds (except the esd in the dihedral angle between two l.s. planes)
 are estimated using the full covariance matrix.  The cell esds are taken
 into account individually in the estimation of esds in distances, angles
 and torsion angles; correlations between esds in cell parameters are only
 used when they are defined by crystal symmetry.  An approximate (isotropic)
 treatment of cell esds is used for estimating esds involving l.s. planes.
;

loop_
 _geom_bond_atom_site_label_1
 _geom_bond_atom_site_label_2
 _geom_bond_distance
 _geom_bond_site_symmetry_2
 _geom_bond_publ_flag
Zn1 N2 2.210(6) . ?
Zn1 N2 2.210(6) 2_756 ?
Zn1 N1 2.254(6) 2_756 ?
Zn1 N1 2.254(6) . ?
```

```
Zn1 Cl1 2.386(2) . ?
Zn1 Cl1 2.386(2) 2_756 ?
N1 C1 1.319(9) . ?
N1 C5 1.347(9) . ?
N2 C6 1.260(10) . ?
N2 C8 1.463(9) . ?
O1 C6 1.336(9) . ?
O1 C7 1.451(9) . ?
O2 H2A 0.80(9) . ?
C1 C2 1.377(10) . ?
C1 H1 0.9300 . ?
C2 C3 1.362(12) . ?
C2 H2 0.9300 . ?
C3 C4 1.378(11) . ?
C3 H3 0.9300 . ?
C4 C5 1.375(11) . ?
C4 H4 0.9300 . ?
C5 C6 1.463(10) . ?
C7 C8 1.511(11) . ?
C7 H7A 0.9700 . ?
C7 H7B 0.9700 . ?
C8 C9 1.547(11) . ?
C8 H8 0.9800 . ?
C9 C10 1.513(10) . ?
C9 H9A 0.9700 . ?
C9 H9B 0.9700 . ?
C10 C11 1.380(11) . ?
C10 C15 1.386(11) . ?
C11 C12 1.387(13) . ?
C11 H11 0.9300 . ?
C12 C13 1.358(14) . ?
C12 H12 0.9300 . ?
C13 C14 1.382(14) . ?
C13 H13 0.9300 . ?
C14 C15 1.353(12) . ?
C14 H14 0.9300 . ?
C15 H15 0.9300 . ?

loop_
 _geom_angle_atom_site_label_1
 _geom_angle_atom_site_label_2
 _geom_angle_atom_site_label_3
 _geom_angle
 _geom_angle_site_symmetry_1
```

_geom_angle_site_symmetry_3
_geom_angle_publ_flag
N2 Zn1 N2 88.2(3) . 2_756 ?
N2 Zn1 N1 88.6(2) . 2_756 ?
N2 Zn1 N1 73.9(2) 2_756 2_756 ?
N2 Zn1 N1 73.9(2) . . ?
N2 Zn1 N1 88.6(2) 2_756 . ?
N1 Zn1 N1 155.7(3) 2_756 . ?
N2 Zn1 Cl1 166.70(17) . . ?
N2 Zn1 Cl1 88.55(16) 2_756 . ?
N1 Zn1 Cl1 102.89(16) 2_756 . ?
N1 Zn1 Cl1 93.15(16) . . ?
N2 Zn1 Cl1 88.55(16) . 2_756 ?
N2 Zn1 Cl1 166.70(17) 2_756 2_756 ?
N1 Zn1 Cl1 93.15(16) 2_756 2_756 ?
N1 Zn1 Cl1 102.89(16) . 2_756 ?
Cl1 Zn1 Cl1 97.40(11) . 2_756 ?
C1 N1 C5 116.1(7) . . ?
C1 N1 Zn1 128.8(5) . . ?
C5 N1 Zn1 115.0(5) . . ?
C6 N2 C8 107.0(6) . . ?
C6 N2 Zn1 113.7(5) . . ?
C8 N2 Zn1 138.9(5) . . ?
C6 O1 C7 103.8(6) . . ?
N1 C1 C2 124.1(8) . . ?
N1 C1 H1 118.0 . . ?
C2 C1 H1 118.0 . . ?
C3 C2 C1 119.3(8) . . ?
C3 C2 H2 120.3 . . ?
C1 C2 H2 120.3 . . ?
C2 C3 C4 118.2(8) . . ?
C2 C3 H3 120.9 . . ?
C4 C3 H3 120.9 . . ?
C5 C4 C3 118.9(8) . . ?
C5 C4 H4 120.6 . . ?
C3 C4 H4 120.6 . . ?
N1 C5 C4 123.4(7) . . ?
N1 C5 C6 112.8(7) . . ?
C4 C5 C6 123.7(7) . . ?
N2 C6 O1 118.5(7) . . ?
N2 C6 C5 123.2(7) . . ?
O1 C6 C5 118.2(7) . . ?
O1 C7 C8 104.1(6) . . ?
O1 C7 H7A 110.9 . . ?

C8 C7 H7A 110.9 . . ?
O1 C7 H7B 110.9 . . ?
C8 C7 H7B 110.9 . . ?
H7A C7 H7B 109.0 . . ?
N2 C8 C7 102.2(6) . . ?
N2 C8 C9 109.1(6) . . ?
C7 C8 C9 114.2(6) . . ?
N2 C8 H8 110.3 . . ?
C7 C8 H8 110.3 . . ?
C9 C8 H8 110.3 . . ?
C10 C9 C8 111.4(6) . . ?
C10 C9 H9A 109.3 . . ?
C8 C9 H9A 109.3 . . ?
C10 C9 H9B 109.3 . . ?
C8 C9 H9B 109.3 . . ?
H9A C9 H9B 108.0 . . ?
C11 C10 C15 117.9(8) . . ?
C11 C10 C9 121.4(8) . . ?
C15 C10 C9 120.7(7) . . ?
C10 C11 C12 121.0(9) . . ?
C10 C11 H11 119.5 . . ?
C12 C11 H11 119.5 . . ?
C13 C12 C11 119.0(9) . . ?
C13 C12 H12 120.5 . . ?
C11 C12 H12 120.5 . . ?
C12 C13 C14 120.9(10) . . ?
C12 C13 H13 119.5 . . ?
C14 C13 H13 119.5 . . ?
C15 C14 C13 119.4(10) . . ?
C15 C14 H14 120.3 . . ?
C13 C14 H14 120.3 . . ?
C14 C15 C10 121.6(9) . . ?
C14 C15 H15 119.2 . . ?
C10 C15 H15 119.2 . . ?

loop_
_geom_torsion_atom_site_label_1
_geom_torsion_atom_site_label_2
_geom_torsion_atom_site_label_3
_geom_torsion_atom_site_label_4
_geom_torsion
_geom_torsion_site_symmetry_1
_geom_torsion_site_symmetry_2
_geom_torsion_site_symmetry_3

_geom_torsion_site_symmetry_4
_geom_torsion_publ_flag
C5 N1 C1 C2 -2.4(11) . . . . ?
Zn1 N1 C1 C2 -179.9(6) . . . . ?
N1 C1 C2 C3 1.1(13) . . . . ?
C1 C2 C3 C4 -0.3(12) . . . . ?
C2 C3 C4 C5 0.8(12) . . . . ?
C1 N1 C5 C4 2.9(10) . . . . ?
Zn1 N1 C5 C4 -179.2(5) . . . . ?
C1 N1 C5 C6 -173.9(6) . . . . ?
Zn1 N1 C5 C6 4.0(7) . . . . ?
C3 C4 C5 N1 -2.2(11) . . . . ?
C3 C4 C5 C6 174.2(7) . . . . ?
C8 N2 C6 O1 -2.9(9) . . . . ?
Zn1 N2 C6 O1 171.6(5) . . . . ?
C8 N2 C6 C5 172.7(6) . . . . ?
Zn1 N2 C6 C5 -12.8(9) . . . . ?
C7 O1 C6 N2 -10.9(8) . . . . ?
C7 O1 C6 C5 173.3(6) . . . . ?
N1 C5 C6 N2 6.0(10) . . . . ?
C4 C5 C6 N2 -170.8(7) . . . . ?
N1 C5 C6 O1 -178.3(5) . . . . ?
C4 C5 C6 O1 4.9(10) . . . . ?
C6 O1 C7 C8 18.9(7) . . . . ?
C6 N2 C8 C7 14.7(7) . . . . ?
Zn1 N2 C8 C7 -157.7(6) . . . . ?
C6 N2 C8 C9 -106.6(7) . . . . ?
Zn1 N2 C8 C9 81.1(8) . . . . ?
O1 C7 C8 N2 -20.3(7) . . . . ?
O1 C7 C8 C9 97.4(7) . . . . ?
N2 C8 C9 C10 -172.7(6) . . . . ?
C7 C8 C9 C10 73.7(9) . . . . ?
C8 C9 C10 C11 -98.2(8) . . . . ?
C8 C9 C10 C15 83.1(9) . . . . ?
C15 C10 C11 C12 -2.8(11) . . . . ?
C9 C10 C11 C12 178.5(7) . . . . ?
C10 C11 C12 C13 1.3(14) . . . . ?
C11 C12 C13 C14 1.3(14) . . . . ?
C12 C13 C14 C15 -2.4(14) . . . . ?
C13 C14 C15 C10 0.9(14) . . . . ?
C11 C10 C15 C14 1.7(12) . . . . ?
C9 C10 C15 C14 -179.6(7) . . . . ?

_refine_diff_density_max   0.554

```
_refine_diff_density_min   -0.296
_refine_diff_density_rms    0.077

_shelxl_version_number 2013-4

_shelx_res_file
;
TITL cd16458 in C2
CELL 0.71073  14.3610  10.2938   9.4877  90.000  93.739  90.000
ZERR    2.00   0.0022   0.0014   0.0014   0.000   0.004   0.000
LATT -7
SYMM -X, Y, -Z
SFAC C  H  N  O  Cl Zn
UNIT 60 60 8 6 4 2
OMIT  -3.00  52.00
L.S. 10
ACTA
BOND  $H
FMAP 2
PLAN 20
wpdb -1
size 0.20 0.16 0.12
mpla c10 > c15
mpla n1 c1 > c5
htab
conf
WGHT    0.059800
FVAR       0.15920
MOLE 1
ZN1   6    1.000000    1.034464    0.500000    10.50000    0.03686    0.03669 =
        0.03776    0.00000    0.00440    0.00000
CL1   5    1.006579    1.187431    0.689288    11.00000    0.05816    0.04629 =
        0.04438   -0.00594    0.00302   -0.00021
N1    3    0.848396    0.988328    0.523721    11.00000    0.03623    0.03673 =
        0.03877    0.00280    0.00413    0.00429
N2    3    0.962746    0.880268    0.344255    11.00000    0.04030    0.03496 =
        0.03533   -0.00320    0.00542   -0.00323
O1    4    0.845310    0.778404    0.225009    11.00000    0.04446    0.04746 =
        0.03665   -0.00796   -0.00553   -0.00103
MOLE 3
O2    4    1.000000    0.045668    0.000000    10.50000    0.13941    0.06632 =
        0.06310    0.00000    0.04227    0.00000
MOLE 1
C1    1    0.794163    1.026930    0.622567    11.00000    0.05000    0.03951 =
```

```
         0.04743   -0.00020    0.00600    0.00240
AFIX  43
H1    2    0.819949    1.080662    0.693616    11.00000   -1.20000
AFIX   0
C2    1    0.701741    0.992627    0.627111    11.00000    0.05033    0.05662 =
         0.05531    0.00416    0.02192    0.01312
AFIX  43
H2    2    0.666496    1.024047    0.698548    11.00000   -1.20000
AFIX   0
C3    1    0.662478    0.912219    0.525922    11.00000    0.03637    0.05640 =
         0.06499    0.00685    0.00771   -0.00129
AFIX  43
H3    2    0.600268    0.887352    0.526945    11.00000   -1.20000
AFIX   0
C4    1    0.717470    0.868719    0.421974    11.00000    0.04232    0.03181 =
         0.05155    0.00064   -0.00980   -0.00086
AFIX  43
H4    2    0.692854    0.814539    0.350476    11.00000   -1.20000
AFIX   0
C5    1    0.809396    0.906807    0.425775    11.00000    0.03536    0.03002 =
         0.03367    0.00739    0.00180    0.00381
C6    1    0.876447    0.857692    0.329088    11.00000    0.04733    0.02879 =
         0.03384    0.00479   -0.00365    0.00330
C7    1    0.925439    0.761669    0.141128    11.00000    0.06200    0.04013 =
         0.03155    0.00176    0.00623    0.00424
AFIX  23
H7A   2    0.920366    0.816533    0.057859    11.00000   -1.20000
H7B   2    0.931359    0.671918    0.111873    11.00000   -1.20000
AFIX   0
C8    1    1.007468    0.801779    0.239358    11.00000    0.04421    0.02851 =
         0.03529   -0.00619    0.00299   -0.00026
AFIX  13
H8    2    1.051050    0.854923    0.188785    11.00000   -1.20000
AFIX   0
C9    1    1.059510    0.687136    0.314801    11.00000    0.04896    0.05099 =
         0.03478   -0.00417    0.00255    0.00410
AFIX  23
H9A   2    1.099864    0.720181    0.392623    11.00000   -1.20000
H9B   2    1.014487    0.629026    0.353404    11.00000   -1.20000
AFIX   0
C10   1    1.117367    0.612518    0.214560    11.00000    0.05186    0.03180 =
         0.03461    0.00606   -0.00559    0.01079
C11   1    1.084994    0.499178    0.150957    11.00000    0.05644    0.03943 =
         0.05501    0.00173   -0.00599    0.00054
```

```
AFIX  43
H11   2    1.027024    0.466699    0.171688    11.00000   -1.20000
AFIX   0
C12   1    1.137555    0.432939    0.056597    11.00000    0.08759    0.05781 =
         0.05775   -0.02179   -0.02070    0.03108
AFIX  43
H12   2    1.114665    0.357581    0.012768    11.00000   -1.20000
AFIX   0
C13   1    1.222911    0.479718    0.029147    11.00000    0.08255    0.08108 =
         0.04509    0.00533    0.01228    0.04983
AFIX  43
H13   2    1.258008    0.436867   -0.035482    11.00000   -1.20000
AFIX   0
C14   1    1.258350    0.590129    0.095926    11.00000    0.05450    0.08928 =
         0.07168    0.01905    0.01170    0.01763
AFIX  43
H14   2    1.317834    0.619578    0.079106    11.00000   -1.20000
AFIX   0
C15   1    1.205750    0.655015    0.185993    11.00000    0.04561    0.04524 =
         0.06219    0.00047    0.00422    0.00013
AFIX  43
H15   2    1.229480    0.729928    0.229777    11.00000   -1.20000
AFIX   0
H2A   2    0.979590    0.090129    0.060110    11.00000    0.08679
HKLF 4

REM  cd16458 in C2
REM R1 =  0.0468 for    2078 Fo > 4sig(Fo)  and  0.0601 for all    2600 data
REM    186 parameters refined using      1 restraints

END

WGHT      0.0596      0.0000

REM Instructions for potential hydrogen bonds
HTAB C1 Cl1
EQIV $1 -x+2, y-1, -z+1
HTAB O2 Cl1_$1

REM Highest difference peak  0.554,  deepest hole -0.296,  1-sigma level  0.077
Q1    1   1.0440  1.0211  0.5974  11.00000  0.05    0.40
Q2    1   1.0021  0.9442  0.5584  11.00000  0.05    0.39
Q3    1   1.0200  0.0846  0.0581  11.00000  0.05    0.28
Q4    1   0.9892  0.1395  0.1040  11.00000  0.05    0.25
```

```
Q5    1    1.0207   1.1227   0.7556   11.00000   0.05     0.25
Q6    1    1.0240   1.0418   0.4001   11.00000   0.05     0.24
Q7    1    0.9174   1.0219   0.4965   11.00000   0.05     0.24
Q8    1    1.0130   1.1000   0.7220   11.00000   0.05     0.24
Q9    1    1.0616   0.7014   0.4259   11.00000   0.05     0.23
Q10   1    1.0531   1.2092   0.7868   11.00000   0.05     0.23
Q11   1    1.0512   0.5322   0.4471   11.00000   0.05     0.23
Q12   1    1.0639   0.7414   0.3012   11.00000   0.05     0.22
Q13   1    1.0177   0.2392   0.1377   11.00000   0.05     0.22
Q14   1    1.0591   1.2182   0.7250   11.00000   0.05     0.22
Q15   1    1.0037   0.8829   0.2489   11.00000   0.05     0.21
Q16   1    1.2024   0.4306   0.0978   11.00000   0.05     0.21
Q17   1    0.9809   1.1704   0.7727   11.00000   0.05     0.21
Q18   1    1.2463   0.5750   0.1853   11.00000   0.05     0.21
Q19   1    0.7606   0.9595   0.3625   11.00000   0.05     0.21
Q20   1    0.9419   0.8315   0.2836   11.00000   0.05     0.20
;
_shelx_res_checksum   62206

_shelx_hkl_file
;
  -2   0   0  114.89    3.28
  -4   0   0  281.49    7.70
  -6   0   0  294.70    8.50
  -8   0   0   80.09    3.61
 -10   0   0   57.98    3.34
 -12   0   0   20.39    2.41
 -14   0   0   28.05    3.02
 -16   0   0    5.61    1.92
 -16   0   0    8.92    2.05
 -18   0   0    1.48    1.52
 -18   0   0    0.99    1.50
 -19   1   0    1.26    1.65
 -19   1   0    2.04    1.60
 -17   1   0    5.17    1.95
 -17   1   0    6.84    2.01
 -15   1   0    3.98    1.65
 -13   1   0   14.25    2.36
 -11   1   0   32.79    2.72
  -9   1   0   54.89    3.16
  -7   1   0  195.81    6.52
  -5   1   0  316.35    8.94
  -3   1   0  456.96   11.78
   1  -1   0  256.50    6.76
```

1	−1	0	262.28	6.35
−1	1	0	244.31	6.51
−1	−1	0	255.17	6.43
−3	−1	0	443.49	11.39
−3	−1	0	462.19	11.37
−5	−1	0	305.71	8.35
−7	−1	0	202.88	6.41
−9	−1	0	46.62	2.71
−11	−1	0	29.25	2.57
−11	−1	0	28.39	2.32
−13	−1	0	16.28	2.21
−13	−1	0	12.30	2.14
−15	−1	0	6.20	1.73
−15	−1	0	6.26	1.81
−17	−1	0	4.39	1.81
−17	−1	0	4.98	1.81
−19	−1	0	2.02	1.37
−18	2	0	3.19	1.86
−16	2	0	5.40	2.01
−14	2	0	2.85	1.49
−12	2	0	12.96	2.23
−10	2	0	30.00	2.56
−8	2	0	35.16	2.46
−6	2	0	12.83	1.46
−4	2	0	162.29	5.14
2	−2	0	192.72	5.80
2	−2	0	199.95	5.42
−2	2	0	211.16	5.67
0	−2	0	155.38	4.17
0	−2	0	152.67	4.36
0	2	0	145.56	3.97
−2	−2	0	190.18	5.38
−4	−2	0	158.99	4.45
−4	−2	0	156.35	4.97
−6	−2	0	13.36	1.38
−6	−2	0	14.23	1.13
−8	−2	0	31.54	1.94
−8	−2	0	23.88	1.95
−10	−2	0	22.44	2.27
−10	−2	0	25.38	2.00
−12	−2	0	11.90	1.78
−12	−2	0	7.18	1.66
−14	−2	0	2.75	1.39
−14	−2	0	4.29	1.44

−16	−2	0	2.60	1.41
−16	−2	0	0.92	1.26
−18	−2	0	0.72	1.49
−17	3	0	−0.53	1.32
−15	3	0	2.47	1.53
−13	3	0	22.26	2.90
−11	3	0	0.73	1.07
−9	3	0	1.47	1.02
−7	3	0	5.74	1.23
5	−3	0	21.88	1.45
−5	3	0	22.63	1.67
3	−3	0	15.73	1.23
3	−3	0	17.01	1.15
−3	3	0	17.48	1.25
1	−3	0	84.03	2.86
1	−3	0	84.95	3.02
−1	−3	0	82.71	2.90
−3	−3	0	18.09	1.36
−5	−3	0	20.59	1.59
−5	−3	0	20.43	1.09
−7	−3	0	4.77	1.17
−7	−3	0	4.02	0.76
−9	−3	0	0.74	0.68
−9	−3	0	0.49	0.82
−11	−3	0	0.66	0.79
−11	−3	0	0.34	1.07
−13	−3	0	18.93	2.66
−13	−3	0	15.87	2.01
−15	−3	0	2.93	1.54
−17	−3	0	0.63	1.36
−18	4	0	0.45	1.59
−16	4	0	−0.43	1.39
−14	4	0	8.93	2.26
−12	4	0	17.16	2.61
−10	4	0	3.85	1.37
−8	4	0	16.58	2.04
6	−4	0	18.28	1.52
−6	4	0	20.10	1.91
4	−4	0	12.86	1.25
4	−4	0	13.50	1.42
−4	4	0	12.16	1.33
2	−4	0	18.46	1.42
2	−4	0	19.13	1.34
−2	4	0	18.07	1.24

0	−4	0	26.59	1.56
0	−4	0	25.97	1.53
0	4	0	21.02	1.09
−2	−4	0	18.23	1.39
−4	−4	0	13.26	1.50
−6	−4	0	18.56	1.87
−6	−4	0	17.04	0.99
−8	−4	0	14.02	1.16
−8	−4	0	16.17	2.00
−10	−4	0	5.31	1.06
−10	−4	0	4.38	1.42
−12	−4	0	20.67	2.71
−14	−4	0	14.77	2.63
−16	−4	0	1.05	1.30
−18	−4	0	0.64	1.53
−17	5	0	2.51	1.85
−15	5	0	4.24	1.88
−13	5	0	12.73	2.49
−11	5	0	8.04	1.92
−9	5	0	32.99	2.77
−7	5	0	147.28	5.86
5	−5	0	51.30	2.90
5	−5	0	51.73	2.61
−5	5	0	51.54	2.84
3	−5	0	29.63	1.79
3	−5	0	25.87	1.89
−3	5	0	25.87	1.76
1	−5	0	130.47	4.76
1	−5	0	134.93	4.58
−1	5	0	135.98	4.26
−1	−5	0	142.91	4.97
−1	−5	0	140.61	4.81
1	5	0	131.55	3.76
−3	−5	0	28.36	2.03
−5	−5	0	52.83	2.95
−7	−5	0	156.14	6.13
−7	−5	0	153.71	4.33
−9	−5	0	31.40	2.76
−11	−5	0	10.36	2.08
−13	−5	0	18.39	2.77
−15	−5	0	5.23	1.88
−17	−5	0	2.65	1.70
−16	6	0	4.04	1.82
−14	6	0	6.43	2.11

−12	6	0	3.10	1.52
−10	6	0	27.63	2.92
−8	6	0	16.93	2.23
6	−6	0	20.61	1.79
6	−6	0	25.11	2.26
−6	6	0	23.30	2.24
4	−6	0	127.09	4.76
4	−6	0	130.27	5.28
−4	6	0	126.93	4.87
2	−6	0	104.68	4.08
2	−6	0	100.05	4.23
0	−6	0	111.22	4.35
0	−6	0	116.02	4.53
0	6	0	107.89	3.54
−2	−6	0	107.81	4.36
−4	−6	0	126.90	5.10
−6	−6	0	23.49	2.40
−8	−6	0	19.04	2.45
−10	−6	0	26.94	3.08
−12	−6	0	2.98	1.62
−14	−6	0	11.10	2.61
−16	−6	0	2.87	1.71
−15	7	0	4.19	1.90
−13	7	0	2.46	1.45
−11	7	0	15.54	2.57
−9	7	0	28.08	2.89
7	−7	0	24.83	2.62
7	−7	0	26.12	2.13
−7	7	0	23.82	2.47
5	−7	0	63.01	3.64
5	−7	0	61.34	3.29
−5	7	0	68.79	3.47
3	−7	0	45.09	2.67
3	−7	0	48.20	2.98
−3	7	0	44.52	2.49
1	−7	0	91.90	4.06
1	−7	0	91.55	4.22
−1	7	0	95.29	3.47
−1	−7	0	89.67	4.20
−1	−7	0	94.38	4.28
1	7	0	98.46	2.98
−3	−7	0	42.00	2.78
−5	−7	0	57.73	3.53
−7	−7	0	27.57	2.77

−9	−7	0	29.54	3.11
−11	−7	0	16.51	2.76
−13	−7	0	1.17	1.71
−15	−7	0	3.84	2.18
−14	8	0	1.46	1.44
−12	8	0	10.41	2.35
−10	8	0	10.81	2.19
8	−8	0	6.74	1.58
8	−8	0	7.44	1.80
−8	8	0	7.42	1.75
6	−8	0	46.80	3.33
6	−8	0	42.10	2.89
4	−8	0	29.94	2.89
4	−8	0	31.24	2.38
−4	8	0	38.59	2.48
2	−8	0	35.51	2.52
2	−8	0	31.26	2.56
−2	8	0	38.03	2.17
0	−8	0	32.25	2.47
0	−8	0	38.84	2.81
−2	−8	0	39.02	2.87
−2	−8	0	31.38	2.59
−4	−8	0	33.92	2.77
−6	−8	0	48.19	3.45
−8	−8	0	5.32	1.77
−10	−8	0	11.46	2.48
−12	−8	0	6.54	2.26
−14	−8	0	3.32	2.01
−13	9	0	2.02	1.50
−11	9	0	3.65	1.64
9	−9	0	9.54	2.00
−9	9	0	13.25	2.39
7	−9	0	1.18	1.36
7	−9	0	3.48	1.35
−7	9	0	2.00	1.31
5	−9	0	14.62	2.32
5	−9	0	8.17	1.70
−5	9	0	10.60	1.76
3	−9	0	6.38	1.61
3	−9	0	9.20	1.74
−3	9	0	9.11	1.41
1	−9	0	0.64	0.94
1	−9	0	0.78	1.00
−1	−9	0	1.17	1.15

-1	-9	0	0.35	1.07
-3	-9	0	7.73	1.81
-3	-9	0	8.87	1.89
-5	-9	0	9.50	2.04
-7	-9	0	2.59	1.53
-9	-9	0	10.54	2.40
-11	-9	0	4.18	2.03
-13	-9	0	-0.10	1.71
-12	10	0	1.01	1.42
-10	10	0	4.57	1.72
8	-10	0	1.78	1.35
8	-10	0	2.60	1.30
-8	10	0	0.73	1.19
6	-10	0	1.96	1.21
6	-10	0	-0.16	1.22
-6	10	0	0.17	1.03
4	-10	0	-0.07	0.90
4	-10	0	-1.12	1.12
2	-10	0	0.69	1.15
2	-10	0	0.53	1.01
0	-10	0	5.58	1.63
0	-10	0	8.43	2.01
-2	-10	0	0.38	1.18
-2	-10	0	-0.54	1.05
-4	-10	0	0.13	1.22
-4	-10	0	0.34	1.07
-6	-10	0	0.84	1.35
-8	-10	0	2.61	1.60
-10	-10	0	2.59	1.81
-12	-10	0	-0.29	1.71
-11	11	0	2.27	1.57
7	-11	0	-0.11	1.47
5	-11	0	6.11	1.76
5	-11	0	5.71	1.93
3	-11	0	2.42	1.32
3	-11	0	1.05	1.33
1	-11	0	1.89	1.29
1	-11	0	2.19	1.54
-1	-11	0	1.53	1.36
-1	-11	0	2.72	1.48
-3	-11	0	1.27	1.39
-3	-11	0	1.95	1.55
-5	-11	0	7.13	2.01
-5	-11	0	8.06	2.17

-7	-11	0	0.81	1.37
-9	-11	0	2.68	1.85
-11	-11	0	2.18	1.91
4	-12	0	4.93	1.72
4	-12	0	4.14	2.07
2	-12	0	11.52	2.34
2	-12	0	9.67	2.31
0	-12	0	10.29	2.28
0	-12	0	9.61	2.17
-2	-12	0	9.52	2.40
-2	-12	0	9.58	2.31
-4	-12	0	5.57	2.00
-4	-12	0	3.28	1.84
-6	-12	0	3.96	1.77
-6	-12	0	2.02	1.91
-8	-12	0	7.15	2.28
-8	-12	0	8.85	2.59
1	-13	0	7.52	2.42
-1	-13	0	6.12	2.02
-1	-13	0	6.55	2.10
-3	-13	0	8.98	2.48
-3	-13	0	10.66	2.56
-5	-13	0	7.59	2.32
-5	-13	0	9.38	2.57
-5	-13	1	3.42	2.12
-5	-13	1	4.43	2.00
-3	-13	1	5.91	2.21
-1	-13	1	3.65	1.91
1	-13	1	1.36	1.66
3	-13	1	1.82	1.72
-8	-12	1	5.86	2.28
-8	-12	1	6.27	2.18
-6	-12	1	-0.23	1.50
-6	-12	1	0.44	1.49
-4	-12	1	9.40	2.45
-4	-12	1	6.53	2.03
-2	-12	1	5.07	1.98
-2	-12	1	5.65	1.99
0	-12	1	3.29	1.69
2	-12	1	3.82	1.71
4	-12	1	5.98	1.89
6	-12	1	0.69	1.38
-11	-11	1	1.27	1.85
-9	-11	1	3.65	2.03

-7	-11	1	1.76	1.61
-5	-11	1	1.63	1.53
-5	-11	1	0.21	1.28
-3	-11	1	7.24	2.02
-3	-11	1	7.50	1.92
-1	-11	1	2.31	1.48
-1	-11	1	3.67	1.55
1	-11	1	1.73	1.41
1	-11	1	2.00	1.44
3	-11	1	0.31	1.34
3	-11	1	1.82	1.29
5	-11	1	3.93	1.67
7	-11	1	5.54	1.77
9	-11	1	1.34	1.43
-11	11	-1	0.75	1.46
-12	-10	1	2.58	1.95
-10	-10	1	0.75	1.53
-8	-10	1	1.26	1.56
-6	-10	1	8.14	2.19
-4	-10	1	7.43	2.05
-4	-10	1	9.28	2.07
-2	-10	1	2.91	1.36
-2	-10	1	2.35	1.43
0	-10	1	1.46	1.29
0	-10	1	1.13	1.09
2	-10	1	8.54	1.88
2	-10	1	6.94	1.75
4	-10	1	4.94	1.68
4	-10	1	5.07	1.64
-6	10	-1	1.23	1.21
6	-10	1	2.19	1.25
-8	10	-1	5.73	1.70
8	-10	1	4.96	1.63
-10	10	-1	2.36	1.47
-12	10	-1	3.44	1.77
-13	-9	1	0.91	1.74
-11	-9	1	2.97	1.87
-9	-9	1	0.20	1.36
-7	-9	1	1.34	1.29
-5	-9	1	2.24	1.39
-3	-9	1	1.62	1.16
-3	-9	1	-0.36	1.04
-1	-9	1	1.04	1.15
-1	-9	1	0.12	0.93

1	−9	1	7.68	1.66
1	−9	1	6.69	1.55
−3	9	−1	12.82	1.64
3	−9	1	13.18	2.03
3	−9	1	11.82	1.98
−5	9	−1	15.28	2.01
5	−9	1	16.12	2.21
5	−9	1	14.22	2.14
−7	9	−1	0.00	0.96
7	−9	1	0.04	0.89
7	−9	1	0.52	1.15
−9	9	−1	10.77	2.14
9	−9	1	7.99	1.88
−11	9	−1	2.82	1.57
−13	9	−1	0.36	1.31
−14	−8	1	3.26	2.06
−12	−8	1	5.78	2.15
−10	−8	1	3.39	1.81
−8	−8	1	6.26	1.86
−6	−8	1	5.61	1.75
−4	−8	1	21.35	2.51
−2	−8	1	10.69	1.91
−2	−8	1	13.72	1.93
0	−8	1	18.72	2.18
0	−8	1	21.25	2.20
−2	8	−1	14.19	1.53
2	−8	1	10.79	1.81
2	−8	1	9.66	1.72
−4	8	−1	16.83	1.90
4	−8	1	21.04	2.34
4	−8	1	14.42	1.98
−6	8	−1	11.77	1.86
6	−8	1	15.01	2.10
6	−8	1	14.34	2.10
−8	8	−1	1.32	1.13
8	−8	1	1.07	1.05
−10	8	−1	7.25	1.92
−12	8	−1	4.37	1.76
−14	8	−1	−0.42	1.21
−15	−7	1	5.54	2.02
−13	−7	1	3.58	1.91
−11	−7	1	12.13	2.52
−9	−7	1	24.64	2.96
−7	−7	1	34.25	2.95

−5	−7	1	55.64	3.45
−3	−7	1	52.90	3.29
1	7	−1	27.00	1.39
−1	−7	1	29.72	2.18
−1	−7	1	29.63	2.36
−1	7	−1	17.74	1.39
1	−7	1	22.99	2.13
1	−7	1	20.94	1.87
−3	7	−1	18.98	1.63
3	−7	1	16.65	1.89
3	−7	1	15.58	1.85
−5	7	−1	22.26	2.13
5	−7	1	16.77	1.98
5	−7	1	17.49	1.99
−7	7	−1	1.95	1.10
7	−7	1	4.49	1.32
7	−7	1	3.42	1.21
−9	7	−1	12.61	2.22
−11	7	−1	10.98	2.29
−13	7	−1	0.89	1.23
−15	7	−1	1.32	1.47
−16	−6	1	2.08	1.78
−14	−6	1	5.35	1.93
−12	−6	1	12.24	2.45
−10	−6	1	38.78	3.29
−8	−6	1	34.46	2.94
−6	−6	1	10.73	1.81
−4	−6	1	64.96	3.43
2	6	−1	20.59	0.93
−2	−6	1	16.69	1.81
0	6	−1	162.63	4.84
0	−6	1	158.61	5.68
0	−6	1	152.48	5.48
2	−6	1	32.13	2.19
2	−6	1	31.99	2.11
−4	6	−1	14.84	1.66
4	−6	1	16.55	1.78
4	−6	1	17.59	1.80
−6	6	−1	31.99	2.38
6	−6	1	34.38	2.37
6	−6	1	30.72	2.32
−8	6	−1	7.27	1.62
8	−6	1	10.40	1.77
−10	6	−1	5.16	1.65

−12	6	−1	1.78	1.28
−14	6	−1	8.86	2.38
−16	6	−1	2.17	1.58
−17	−5	1	1.42	1.44
−15	−5	1	2.35	1.51
−13	−5	1	19.92	2.91
−11	−5	1	6.78	1.82
−9	−5	1	16.89	2.29
−7	−5	1	22.73	1.17
−7	−5	1	23.89	2.39
−5	−5	1	27.24	2.15
−3	−5	1	49.64	2.74
1	5	−1	8.51	0.72
−1	−5	1	9.50	1.30
−1	5	−1	78.33	2.97
1	−5	1	82.34	3.27
1	−5	1	84.01	3.46
−3	5	−1	38.81	2.09
3	−5	1	31.55	2.04
3	−5	1	30.53	2.00
−5	5	−1	16.22	1.80
5	−5	1	22.87	1.79
5	−5	1	21.06	1.72
−7	5	−1	27.48	2.27
7	−5	1	27.68	2.11
−9	5	−1	9.16	1.80
−11	5	−1	17.15	2.52
−13	5	−1	10.43	2.37
−15	5	−1	6.56	2.11
−17	5	−1	2.14	1.94
−18	−4	1	2.69	1.75
−16	−4	1	2.70	1.58
−14	−4	1	2.21	1.37
−12	−4	1	12.84	2.24
−10	−4	1	5.14	1.42
−10	−4	1	5.65	1.06
−8	−4	1	15.26	1.24
−8	−4	1	15.80	2.01
−6	−4	1	19.29	1.13
−6	−4	1	18.41	1.96
−4	−4	1	2.27	0.88
−4	−4	1	2.19	0.32
2	4	−1	21.83	0.90
−2	−4	1	21.55	1.59

0	4	−1	1.61	0.43
0	−4	1	2.44	0.73
0	−4	1	1.37	0.51
−2	4	−1	2.95	0.71
2	−4	1	5.74	0.96
−4	4	−1	30.95	1.97
4	−4	1	36.24	2.08
4	−4	1	34.10	1.95
−6	4	−1	38.19	2.49
6	−4	1	44.46	2.45
−8	4	−1	25.80	2.25
−10	4	−1	20.99	2.46
−12	4	−1	8.61	2.02
−14	4	−1	10.37	2.36
−16	4	−1	1.54	1.62
−18	4	−1	2.53	1.97
−17	−3	1	−0.93	1.19
−15	−3	1	2.22	1.44
−13	−3	1	27.82	2.97
−13	−3	1	24.07	2.35
−11	−3	1	1.89	0.87
−11	−3	1	1.89	1.18
−9	−3	1	4.90	1.00
−9	−3	1	4.50	1.29
−7	−3	1	4.11	0.76
−7	−3	1	4.27	1.12
−5	−3	1	13.88	0.93
−5	−3	1	15.06	1.54
−3	−3	1	6.99	1.00
−3	−3	1	7.16	0.49
−1	−3	1	26.07	1.02
−1	−3	1	24.64	1.50
−1	3	−1	319.35	8.63
1	−3	1	344.39	8.85
1	−3	1	329.03	8.91
−3	3	−1	5.25	0.85
3	−3	1	2.87	0.70
−5	3	−1	40.52	2.29
5	−3	1	36.38	2.03
−7	3	−1	43.34	2.72
−9	3	−1	11.76	1.86
−11	3	−1	27.50	2.83
−13	3	−1	8.17	2.12
−15	3	−1	0.85	1.45

−17	3	−1	1.34	1.55
−18	−2	1	2.17	1.58
−16	−2	1	3.97	1.79
−16	−2	1	3.74	1.42
−14	−2	1	17.04	2.21
−14	−2	1	14.63	2.44
−12	−2	1	11.81	1.77
−12	−2	1	11.91	2.05
−10	−2	1	16.08	1.72
−10	−2	1	14.39	1.95
−8	−2	1	10.25	1.54
−8	−2	1	11.31	1.32
−6	−2	1	145.34	4.99
−6	−2	1	140.76	4.54
−4	−2	1	3.28	0.76
−4	−2	1	3.40	0.54
−2	−2	1	27.00	1.04
−2	−2	1	27.10	1.48
0	−2	1	111.38	3.15
−2	2	−1	510.55	12.85
2	−2	1	499.65	13.05
2	−2	1	504.98	12.69
−4	2	−1	9.68	1.11
4	−2	1	7.33	0.88
−6	2	−1	31.82	2.09
−8	2	−1	20.39	2.09
−10	2	−1	11.64	1.91
−12	2	−1	9.80	2.01
−14	2	−1	12.20	2.49
−16	2	−1	5.78	1.96
−18	2	−1	−0.62	1.35
−19	−1	1	5.44	2.02
−17	−1	1	2.68	1.59
−17	−1	1	3.42	1.59
−15	−1	1	5.16	1.61
−15	−1	1	4.76	1.63
−13	−1	1	13.55	1.99
−13	−1	1	15.72	2.20
−11	−1	1	29.60	2.45
−11	−1	1	30.54	2.38
−9	−1	1	55.76	2.98
−9	−1	1	51.44	2.86
−7	−1	1	24.39	1.73
−7	−1	1	28.59	1.87

−5	−1	1	161.37	5.01
−5	−1	1	159.72	4.93
−3	−1	1	93.81	2.96
−3	−1	1	96.16	3.11
−1	−1	1	517.46	12.83
−1	−1	1	540.58	13.03
1	−1	1	118.60	3.38
1	−1	1	121.43	3.41
−3	1	−1	42.15	1.83
−5	1	−1	9.33	1.13
−7	1	−1	66.48	3.18
−9	1	−1	16.04	1.97
−11	1	−1	7.26	1.68
−13	1	−1	7.06	1.84
−15	1	−1	10.84	2.40
−17	1	−1	1.01	1.45
−18	0	1	4.84	1.93
−18	0	1	5.17	1.84
−16	0	1	1.54	1.35
−16	0	1	2.14	1.39
−14	0	1	16.71	2.46
−14	0	1	16.03	2.45
−12	0	1	8.46	1.73
−12	0	1	6.92	1.66
−10	0	1	28.20	2.30
−8	0	1	77.27	3.58
−6	0	1	251.95	7.57
−4	0	1	49.05	2.11
−2	0	1	56.69	1.96
0	0	−1	302.14	7.79
0	0	1	292.82	7.47
2	0	1	40.09	1.51
−4	0	−1	66.75	2.52
−6	0	−1	292.82	8.54
−8	0	−1	11.28	1.55
−10	0	−1	25.43	2.21
−12	0	−1	3.84	1.40
−14	0	−1	2.34	1.42
−16	0	−1	2.34	1.57
−18	0	−1	1.91	1.70
−18	0	−1	2.77	1.47
−19	1	1	2.90	1.83
−19	1	1	2.43	1.63
−17	1	1	3.13	1.58

−17	1	1	2.65	1.61
−15	1	1	6.26	1.86
−15	1	1	6.71	1.81
−13	1	1	18.45	2.49
−11	1	1	36.51	2.85
−9	1	1	55.84	3.18
−7	1	1	27.53	2.02
−5	1	1	172.52	5.56
−3	1	1	84.30	2.95
1	−1	−1	530.33	13.54
−1	1	1	521.09	13.07
1	1	1	−0.17	0.06
−3	−1	−1	36.85	1.51
−5	−1	−1	12.13	1.02
−7	−1	−1	68.05	3.00
−9	−1	−1	14.03	1.68
−11	−1	−1	6.59	1.46
−13	−1	−1	5.52	1.56
−15	−1	−1	9.28	2.29
−15	−1	−1	10.10	2.09
−17	−1	−1	0.99	1.46
−17	−1	−1	1.81	1.41
−18	2	1	2.28	1.70
−18	2	1	1.65	1.63
−16	2	1	1.66	1.53
−14	2	1	19.65	2.80
−12	2	1	12.32	2.18
−10	2	1	13.67	1.98
−8	2	1	19.87	2.02
−6	2	1	140.13	5.09
4	−2	−1	2.84	0.75
−4	2	1	7.33	0.98
2	−2	−1	26.91	1.61
2	−2	−1	28.65	1.15
−2	2	1	32.96	1.51
0	−2	−1	115.66	3.09
0	−2	−1	103.66	3.47
−2	−2	−1	498.74	12.74
2	2	1	523.70	12.57
−4	−2	−1	6.32	0.92
−4	−2	−1	7.76	0.73
−6	−2	−1	31.70	1.73
−6	−2	−1	33.22	2.13
−8	−2	−1	14.84	1.81

−8	−2	−1	15.57	1.56
−10	−2	−1	11.91	1.59
−10	−2	−1	7.98	1.67
−12	−2	−1	4.29	1.45
−12	−2	−1	6.41	1.47
−14	−2	−1	13.16	2.36
−14	−2	−1	13.53	2.13
−16	−2	−1	2.24	1.38
−16	−2	−1	2.35	1.36
−18	−2	−1	1.90	1.55
−17	3	1	0.81	1.31
−15	3	1	1.01	1.37
−13	3	1	29.26	3.22
−11	3	1	2.09	1.14
−9	3	1	5.10	1.34
−7	3	1	2.84	0.96
5	−3	−1	12.68	1.43
−5	3	1	10.55	1.34
3	−3	−1	8.18	0.77
3	−3	−1	7.74	1.14
−3	3	1	12.99	1.19
1	−3	−1	27.09	1.61
1	−3	−1	27.34	1.25
−1	−3	−1	364.92	9.41
1	3	1	362.16	9.11
−3	−3	−1	2.68	0.71
3	3	1	6.35	0.46
−5	−3	−1	38.91	2.24
−5	−3	−1	38.39	1.67
−7	−3	−1	36.91	2.43
−7	−3	−1	39.11	2.00
−9	−3	−1	13.96	1.50
−9	−3	−1	10.04	1.74
−11	−3	−1	25.28	2.62
−11	−3	−1	23.54	2.01
−13	−3	−1	7.45	1.85
−13	−3	−1	5.99	1.46
−15	−3	−1	0.43	1.20
−17	−3	−1	1.58	1.60
−18	4	1	−0.08	1.51
−16	4	1	1.94	1.69
−14	4	1	3.33	1.57
−12	4	1	8.40	1.94
−10	4	1	4.41	1.41

−8	4	1	14.07	1.94
6	−4	−1	18.97	1.91
−6	4	1	14.32	1.72
4	−4	−1	2.55	0.92
4	−4	−1	2.96	0.64
−4	4	1	5.52	1.02
2	−4	−1	21.52	1.27
2	−4	−1	19.21	1.53
−2	4	1	19.61	1.29
0	−4	−1	1.20	0.61
0	−4	−1	1.61	0.56
0	4	1	0.69	0.31
−2	−4	−1	4.32	0.86
2	4	1	3.04	0.33
−4	−4	−1	35.30	2.11
−4	−4	−1	35.64	1.27
−6	−4	−1	43.93	2.60
−6	−4	−1	41.71	1.76
−8	−4	−1	25.44	1.61
−8	−4	−1	29.12	2.38
−10	−4	−1	20.96	1.69
−10	−4	−1	20.75	2.54
−12	−4	−1	11.33	2.23
−14	−4	−1	11.00	2.45
−16	−4	−1	1.74	1.43
−18	−4	−1	1.85	1.50
−17	5	1	1.47	1.59
−15	5	1	−0.04	1.39
−13	5	1	18.91	2.87
−11	5	1	10.44	2.12
−9	5	1	12.93	2.02
7	−5	−1	21.92	2.31
−7	5	1	17.56	2.04
5	−5	−1	24.94	1.62
5	−5	−1	24.60	2.10
−5	5	1	20.79	1.83
3	−5	−1	47.83	2.23
3	−5	−1	48.12	2.75
−3	5	1	49.43	2.43
1	−5	−1	13.05	1.48
1	−5	−1	12.17	1.16
−1	5	1	9.05	0.94
−1	−5	−1	84.68	3.31
−1	−5	−1	79.24	3.62

1	5	1	87.25	2.66
−3	−5	−1	32.32	2.11
−5	−5	−1	18.95	1.95
−7	−5	−1	29.54	2.32
−7	−5	−1	26.56	1.33
−9	−5	−1	9.10	1.79
−11	−5	−1	19.59	2.66
−13	−5	−1	11.23	2.46
−15	−5	−1	4.26	1.98
−17	−5	−1	2.53	1.72
−16	6	1	0.53	1.15
−14	6	1	5.76	1.93
−12	6	1	10.93	2.29
−10	6	1	39.73	3.24
8	−6	−1	34.77	2.84
−8	6	1	31.92	2.70
6	−6	−1	13.66	1.51
6	−6	−1	12.67	1.98
−6	6	1	14.75	1.86
4	−6	−1	64.55	3.62
4	−6	−1	62.30	2.91
−4	6	1	67.70	3.20
2	−6	−1	18.66	1.51
2	−6	−1	21.29	2.03
0	−6	−1	153.01	5.82
0	−6	−1	154.65	5.33
0	6	1	157.10	4.68
−2	−6	−1	29.88	2.06
−2	−6	−1	27.68	2.22
−4	−6	−1	18.40	1.94
−6	−6	−1	32.24	2.57
−8	−6	−1	8.22	1.84
−10	−6	−1	3.85	1.59
−12	−6	−1	0.97	1.38
−14	−6	−1	11.02	2.58
−16	−6	−1	1.69	1.87
−15	7	1	7.15	2.11
−13	7	1	2.57	1.52
−11	7	1	10.86	2.27
−9	7	1	21.69	2.65
7	−7	−1	33.63	2.35
7	−7	−1	34.78	3.00
−7	7	1	37.57	2.82
5	−7	−1	65.59	3.18

5	−7	−1	56.78	3.68
−5	7	1	59.96	3.28
3	−7	−1	51.16	3.23
3	−7	−1	48.97	2.66
−3	7	1	49.47	2.59
1	−7	−1	28.39	1.98
1	−7	−1	26.22	2.26
−1	7	1	24.41	1.56
−1	−7	−1	21.55	1.99
−1	−7	−1	20.97	2.17
1	7	1	20.28	1.14
−3	−7	−1	14.97	1.84
−5	−7	−1	16.81	2.13
−7	−7	−1	7.75	1.80
−9	−7	−1	14.60	2.48
−11	−7	−1	11.75	2.55
−13	−7	−1	3.05	1.87
−15	−7	−1	2.26	1.82
−14	8	1	3.44	1.72
−12	8	1	7.37	2.01
−10	8	1	3.70	1.58
8	−8	−1	9.18	2.04
8	−8	−1	8.11	1.60
−8	8	1	5.76	1.60
6	−8	−1	8.80	1.94
6	−8	−1	6.49	1.34
−6	8	1	8.23	1.64
4	−8	−1	20.52	2.50
4	−8	−1	18.83	1.90
−4	8	1	21.61	1.82
2	−8	−1	11.54	1.62
2	−8	−1	13.38	2.00
−2	8	1	14.05	1.42
0	−8	−1	17.88	2.23
0	−8	−1	17.03	1.93
−2	−8	−1	6.39	1.58
−2	−8	−1	11.40	1.74
−4	−8	−1	19.32	2.27
−6	−8	−1	13.73	2.17
−8	−8	−1	−0.05	1.11
−10	−8	−1	8.68	2.15
−12	−8	−1	5.50	2.07
−14	−8	−1	0.32	1.89
−13	9	1	−0.19	1.17

-11	9	1	0.81	1.28
9	-9	-1	1.69	1.43
-9	9	1	1.93	1.30
7	-9	-1	1.00	1.21
7	-9	-1	0.92	1.01
-7	9	1	0.82	1.02
5	-9	-1	3.65	1.26
5	-9	-1	5.15	1.75
-5	9	1	5.17	1.32
3	-9	-1	1.28	1.19
3	-9	-1	1.08	0.95
-3	9	1	1.07	0.68
1	-9	-1	0.74	0.94
1	-9	-1	1.59	1.12
-1	-9	-1	12.71	2.17
-1	-9	-1	9.54	1.81
-3	-9	-1	10.28	2.03
-3	-9	-1	13.68	2.09
-5	-9	-1	13.47	2.23
-7	-9	-1	-0.99	1.13
-9	-9	-1	9.42	2.29
-11	-9	-1	5.49	2.07
-13	-9	-1	0.33	1.70
-12	10	1	0.39	1.23
-10	10	1	0.01	1.11
8	-10	-1	0.50	1.40
-8	10	1	2.24	1.34
6	-10	-1	9.34	2.09
-6	10	1	7.58	1.69
4	-10	-1	9.15	2.14
4	-10	-1	8.73	1.73
2	-10	-1	2.68	1.39
2	-10	-1	2.79	1.26
0	-10	-1	2.38	1.35
0	-10	-1	1.78	1.23
-2	-10	-1	9.67	2.15
-2	-10	-1	4.83	1.58
-4	-10	-1	4.80	1.65
-4	-10	-1	8.36	2.05
-6	-10	-1	2.14	1.44
-8	-10	-1	4.60	1.87
-10	-10	-1	5.88	2.11
-12	-10	-1	2.55	1.91
-11	11	1	0.88	1.21

9	-11	-1	4.31	1.88
7	-11	-1	1.70	1.67
5	-11	-1	0.12	1.28
3	-11	-1	4.99	1.57
3	-11	-1	8.99	2.19
1	-11	-1	2.66	1.39
1	-11	-1	2.56	1.51
-1	-11	-1	2.65	1.62
-1	-11	-1	2.09	1.38
-3	-11	-1	1.22	1.42
-3	-11	-1	0.47	1.35
-5	-11	-1	5.70	2.00
-5	-11	-1	4.79	1.90
-7	-11	-1	7.52	2.17
-7	-11	-1	5.09	2.02
-9	-11	-1	3.33	1.85
-11	-11	-1	0.26	1.69
8	-12	-1	5.24	2.32
6	-12	-1	-0.31	1.33
4	-12	-1	10.67	2.55
2	-12	-1	5.74	2.03
0	-12	-1	3.93	1.69
0	-12	-1	2.59	1.60
-2	-12	-1	4.06	1.73
-2	-12	-1	4.22	1.82
-4	-12	-1	5.06	1.99
-4	-12	-1	6.67	2.23
-6	-12	-1	1.18	1.71
-6	-12	-1	0.21	1.36
-8	-12	-1	1.00	1.64
-8	-12	-1	1.23	1.81
5	-13	-1	5.05	2.35
3	-13	-1	3.22	1.89
1	-13	-1	2.75	1.79
-1	-13	-1	2.11	1.55
-3	-13	-1	2.60	1.65
-5	-13	-1	2.31	1.91
-5	-13	-1	1.58	1.80
-5	-13	2	0.49	1.75
-3	-13	2	1.89	1.92
-1	-13	2	1.29	1.68
1	-13	2	-1.05	1.34
3	-13	2	-1.02	1.38
5	-13	2	2.49	1.90

-8	-12	2	0.81	1.93
-6	-12	2	1.28	1.64
-6	-12	2	2.18	1.95
-4	-12	2	7.77	2.40
-2	-12	2	0.66	1.40
0	-12	2	0.57	1.50
2	-12	2	3.00	1.69
4	-12	2	-0.06	1.33
6	-12	2	0.58	1.55
8	-12	2	0.97	1.50
-11	-11	2	2.09	2.00
-9	-11	2	9.10	2.67
-7	-11	2	1.96	1.78
-5	-11	2	6.51	1.93
-5	-11	2	4.94	2.09
-3	-11	2	13.04	2.37
-3	-11	2	14.00	2.72
-1	-11	2	5.75	1.90
1	-11	2	4.69	1.84
3	-11	2	16.99	2.66
5	-11	2	5.59	1.84
7	-11	2	9.56	2.27
9	-11	2	2.07	1.71
-12	-10	2	1.48	1.82
-10	-10	2	6.35	2.30
-8	-10	2	7.73	2.29
-6	-10	2	4.55	1.86
-4	-10	2	9.25	2.27
-4	-10	2	11.21	2.16
-2	-10	2	25.46	2.69
-2	-10	2	22.86	2.91
0	-10	2	21.28	2.70
0	-10	2	20.32	2.44
2	-10	2	25.13	2.93
4	-10	2	17.73	2.54
-6	10	-2	21.47	2.66
6	-10	2	19.81	2.76
-8	10	-2	6.86	2.00
8	-10	2	4.87	1.79
-10	10	-2	3.76	1.69
-12	10	-2	8.51	2.23
-13	-9	2	1.79	1.90
-11	-9	2	2.09	1.75
-9	-9	2	9.18	2.35

−7	−9	2	3.28	1.62
−5	−9	2	11.99	2.33
−3	−9	2	15.46	2.52
−3	−9	2	16.90	2.21
−1	−9	2	12.53	1.90
−1	−9	2	8.75	1.96
1	−9	2	22.89	2.11
1	−9	2	22.37	2.64
−3	9	−2	3.03	0.87
3	−9	2	0.15	0.58
3	−9	2	15.62	2.23
−5	9	−2	18.08	2.19
5	−9	2	20.31	2.60
−7	9	−2	5.18	1.67
7	−9	2	6.62	1.76
−9	9	−2	15.02	2.55
9	−9	2	18.86	2.67
−11	9	−2	8.19	2.23
−13	9	−2	1.37	1.45
−14	−8	2	−0.68	1.21
−12	−8	2	5.72	2.01
−10	−8	2	5.00	1.91
−8	−8	2	6.67	1.88
−6	−8	2	12.14	2.26
−4	−8	2	24.63	2.74
−2	−8	2	21.66	2.20
−2	−8	2	17.95	2.36
0	−8	2	10.69	1.94
0	−8	2	7.44	1.47
−2	8	−2	21.07	1.79
2	−8	2	18.12	2.28
−4	8	−2	10.85	1.68
4	−8	2	11.22	1.71
4	−8	2	12.76	2.02
−6	8	−2	4.02	1.33
6	−8	2	5.44	1.54
6	−8	2	5.24	1.38
−8	8	−2	5.16	1.59
8	−8	2	5.88	1.61
−10	8	−2	6.06	1.81
−12	8	−2	1.77	1.40
−14	8	−2	3.12	1.83
−15	−7	2	2.21	1.70
−13	−7	2	3.36	1.74

−11	−7	2	7.79	1.99
−9	−7	2	15.76	2.55
−7	−7	2	7.58	1.89
−5	−7	2	25.36	2.63
−3	−7	2	17.20	2.20
1	7	−2	16.23	1.21
−1	−7	2	18.07	1.77
−1	−7	2	16.78	2.06
−1	7	−2	7.92	1.17
1	−7	2	10.19	1.67
1	−7	2	5.40	1.02
−3	7	−2	15.00	1.65
3	−7	2	12.98	1.62
3	−7	2	10.68	1.74
−5	7	−2	6.01	1.40
5	−7	2	7.38	1.56
5	−7	2	9.60	1.55
−7	7	−2	13.82	2.05
7	−7	2	17.48	2.25
−9	7	−2	6.79	1.71
−11	7	−2	2.15	1.35
−13	7	−2	2.34	1.54
−15	7	−2	4.52	1.91
−16	−6	2	0.34	1.37
−14	−6	2	3.27	1.72
−12	−6	2	5.60	1.84
−10	−6	2	9.98	2.03
−8	−6	2	32.05	2.76
−6	−6	2	9.75	1.82
−4	−6	2	30.15	1.28
−4	−6	2	32.10	2.47
2	6	−2	15.19	1.03
−2	−6	2	14.30	1.83
0	6	−2	34.00	1.77
0	−6	2	28.53	2.21
−2	6	−2	12.54	1.36
2	−6	2	10.25	1.29
2	−6	2	11.09	1.58
−4	6	−2	10.57	1.51
4	−6	2	8.21	1.24
4	−6	2	9.28	1.53
−6	6	−2	23.36	2.05
6	−6	2	16.26	1.95
−8	6	−2	4.49	1.44

8	−6	2	4.40	1.32
−10	6	−2	9.02	1.99
−12	6	−2	0.21	1.20
−14	6	−2	−0.65	1.06
−16	6	−2	4.86	2.10
−17	−5	2	1.12	1.57
−15	−5	2	2.65	1.68
−13	−5	2	10.61	2.30
−11	−5	2	14.33	2.40
−9	−5	2	10.36	1.95
−7	−5	2	34.97	1.60
−7	−5	2	29.13	2.46
−5	−5	2	28.25	1.28
−5	−5	2	29.36	2.26
−3	−5	2	73.28	3.46
−3	−5	2	69.27	2.33
1	5	−2	131.68	4.04
−1	−5	2	126.19	4.89
1	−5	2	18.89	1.65
1	−5	2	23.90	1.50
−3	5	−2	22.02	1.64
3	−5	2	18.79	1.82
3	−5	2	18.18	1.40
−5	5	−2	46.72	2.73
5	−5	2	41.16	2.61
−7	5	−2	33.58	2.52
7	−5	2	31.78	2.40
−9	5	−2	24.58	2.69
−11	5	−2	11.21	2.21
−13	5	−2	19.80	3.02
−15	5	−2	1.03	1.58
−17	5	−2	2.67	2.03
−18	−4	2	0.89	1.49
−16	−4	2	3.42	1.73
−14	−4	2	9.87	2.26
−12	−4	2	0.31	1.07
−10	−4	2	17.15	1.52
−10	−4	2	15.69	2.24
−8	−4	2	22.84	1.60
−8	−4	2	22.31	2.35
−6	−4	2	15.59	1.12
−6	−4	2	19.25	2.01
−4	−4	2	282.11	8.59
−4	−4	2	284.02	7.50

−2	−4	2	56.75	2.77
−2	−4	2	62.82	2.14
0	−4	2	46.24	1.92
0	−4	2	45.52	1.95
0	−4	2	43.69	2.32
−2	4	−2	305.24	8.66
2	−4	2	320.40	8.62
2	−4	2	305.18	8.92
−4	4	−2	72.36	3.19
4	−4	2	69.69	3.24
−6	4	−2	29.01	2.19
6	−4	2	29.35	2.12
−8	4	−2	61.28	3.58
−10	4	−2	35.74	3.07
−12	4	−2	11.32	2.29
−14	4	−2	4.80	2.01
−16	4	−2	8.31	2.53
−17	−3	2	1.38	1.42
−15	−3	2	0.48	1.19
−13	−3	2	22.45	2.74
−13	−3	2	20.85	2.28
−11	−3	2	9.45	1.54
−11	−3	2	8.02	1.79
−9	−3	2	51.53	3.14
−9	−3	2	50.13	2.67
−7	−3	2	100.58	4.44
−7	−3	2	107.82	3.86
−5	−3	2	37.25	2.23
−5	−3	2	35.79	1.71
−3	−3	2	74.37	2.47
−3	−3	2	70.21	2.99
−1	−3	2	64.15	2.70
−1	−3	2	64.43	2.25
1	−3	2	860.75	21.72
1	−3	2	896.08	22.17
1	−3	2	871.81	21.78
−3	3	−2	186.93	5.88
3	−3	2	193.59	6.00
−5	3	−2	207.57	6.76
5	−3	2	212.45	6.66
−7	3	−2	182.00	6.62
−9	3	−2	44.79	3.05
−11	3	−2	34.38	3.04
−13	3	−2	14.62	2.59

−15	3	−2	6.09	2.04
−17	3	−2	6.44	2.40
−18	−2	2	3.24	1.71
−16	−2	2	5.73	1.89
−16	−2	2	4.99	1.62
−14	−2	2	15.70	2.50
−14	−2	2	17.96	2.34
−12	−2	2	18.71	2.14
−12	−2	2	17.82	2.32
−10	−2	2	1.78	0.90
−10	−2	2	1.34	0.90
−8	−2	2	38.14	2.25
−8	−2	2	38.58	2.49
−6	−2	2	223.63	6.76
−6	−2	2	227.84	7.04
−4	−2	2	188.69	5.47
−4	−2	2	183.76	5.74
−2	−2	2	255.86	7.18
−2	−2	2	268.68	7.04
0	−2	2	971.08	23.69
0	−2	2	984.01	23.92
2	−2	2	124.31	4.01
2	−2	2	120.34	3.95
−4	2	−2	67.21	2.83
4	−2	2	62.69	2.69
−6	2	−2	155.74	5.53
−8	2	−2	97.68	4.50
−10	2	−2	23.05	2.51
−12	2	−2	31.82	3.21
−14	2	−2	13.28	2.52
−16	2	−2	2.19	1.65
−18	2	−2	2.06	1.82
−19	−1	2	2.55	1.76
−17	−1	2	1.06	1.32
−17	−1	2	0.89	1.42
−15	−1	2	3.65	1.60
−15	−1	2	2.91	1.30
−13	−1	2	8.05	1.76
−13	−1	2	5.99	1.56
−11	−1	2	37.13	2.65
−11	−1	2	36.66	2.71
−9	−1	2	27.64	2.10
−9	−1	2	27.90	2.15
−7	−1	2	55.93	2.74

−7	−1	2	56.98	2.78
−5	−1	2	46.08	2.17
−5	−1	2	46.04	2.20
−3	−1	2	112.91	3.64
−3	−1	2	112.13	3.58
−1	−1	2	19.88	1.08
−1	−1	2	19.03	1.03
1	−1	2	509.67	12.55
1	−1	2	498.99	12.77
3	−1	2	243.21	6.88
3	−1	2	236.94	6.67
−5	1	−2	9.11	1.16
−7	1	−2	5.02	1.10
−9	1	−2	2.28	1.08
−11	1	−2	8.45	1.87
−13	1	−2	2.50	1.35
−15	1	−2	5.12	1.86
−17	1	−2	1.79	1.59
−18	0	2	1.48	1.55
−18	0	2	1.39	1.44
−16	0	2	0.02	1.08
−16	0	2	0.01	1.11
−14	0	2	0.86	1.03
−14	0	2	0.39	1.02
−12	0	2	37.13	2.73
−12	0	2	38.21	2.92
−10	0	2	17.02	2.01
−10	0	2	18.88	1.93
−8	0	2	22.42	1.84
−8	0	2	19.29	1.60
−6	0	2	13.75	1.20
−6	0	2	12.73	1.37
−4	0	2	6.89	0.73
−2	0	2	5.82	0.72
−2	0	2	5.86	0.54
0	0	−2	3.76	0.47
0	0	2	2.86	0.47
2	0	2	177.11	5.03
−4	0	−2	14.90	1.20
−6	0	−2	10.00	1.26
−8	0	−2	0.12	0.72
−10	0	−2	1.01	0.96
−12	0	−2	−1.22	0.83
−14	0	−2	5.36	1.78

−16	0	−2	−0.52	1.27
−18	0	−2	−0.08	1.43
−18	0	−2	1.38	1.32
−19	1	2	3.81	1.82
−19	1	2	5.26	2.09
−17	1	2	0.86	1.33
−17	1	2	0.09	1.31
−15	1	2	3.87	1.53
−15	1	2	5.15	1.69
−13	1	2	8.01	1.76
−13	1	2	8.52	1.87
−11	1	2	30.25	2.39
−11	1	2	34.09	2.70
−9	1	2	27.34	2.17
−7	1	2	53.21	2.83
−5	1	2	43.23	2.27
3	−1	−2	104.69	3.72
−3	1	2	109.44	2.95
−3	1	2	100.11	3.61
1	−1	−2	20.83	1.27
−1	1	2	25.27	1.26
1	1	2	511.29	12.84
3	1	2	221.12	6.16
−5	−1	−2	6.51	0.92
−7	−1	−2	5.27	1.06
−9	−1	−2	2.59	1.00
−11	−1	−2	6.65	1.61
−13	−1	−2	3.95	1.45
−15	−1	−2	9.41	2.19
−17	−1	−2	0.93	1.53
−17	−1	−2	3.97	1.70
−18	2	2	4.05	1.76
−18	2	2	3.51	1.95
−16	2	2	3.03	1.60
−16	2	2	6.14	1.87
−14	2	2	13.73	2.43
−12	2	2	16.45	2.34
−10	2	2	−0.24	0.78
−8	2	2	36.55	2.49
−6	2	2	201.42	6.70
4	−2	−2	171.79	5.76
−4	2	2	170.38	5.54
2	−2	−2	264.38	7.87
−2	2	2	267.62	7.48

0	−2	−2	984.71	25.45
0	−2	−2	1000.00	23.51
0	2	2	988.99	24.30
−2	−2	−2	124.36	3.78
−6	−2	−2	161.50	5.19
−8	−2	−2	99.40	4.06
−10	−2	−2	24.12	2.04
−12	−2	−2	28.55	2.48
−12	−2	−2	31.22	3.08
−14	−2	−2	15.97	2.64
−14	−2	−2	18.85	2.57
−16	−2	−2	1.84	1.40
−16	−2	−2	4.02	1.67
−18	−2	−2	−0.28	1.33
−17	3	2	3.31	1.76
−17	3	2	0.31	1.47
−15	3	2	1.83	1.44
−13	3	2	23.89	2.93
−11	3	2	5.63	1.50
−9	3	2	50.19	3.23
−7	3	2	100.06	4.37
5	−3	−2	32.64	2.22
−5	3	2	26.68	1.90
3	−3	−2	75.21	3.37
3	−3	−2	73.90	2.28
−3	3	2	76.23	3.06
1	−3	−2	60.47	2.72
1	−3	−2	63.11	1.99
−1	3	2	61.59	2.43
−1	−3	−2	909.52	21.63
−1	−3	−2	877.06	23.37
1	3	2	895.97	21.94
−3	−3	−2	188.58	5.64
3	3	2	184.39	5.16
−5	−3	−2	220.16	6.87
−5	−3	−2	223.64	6.40
−7	−3	−2	186.40	5.92
−7	−3	−2	191.40	6.57
−9	−3	−2	43.87	2.99
−9	−3	−2	40.00	2.42
−11	−3	−2	37.11	2.55
−11	−3	−2	39.73	3.13
−13	−3	−2	11.50	2.22
−13	−3	−2	17.62	2.22

−15	−3	−2	7.35	2.07
−17	−3	−2	3.96	1.69
−18	4	2	0.57	1.46
−16	4	2	0.72	1.45
−14	4	2	7.63	1.97
−12	4	2	2.90	1.38
−10	4	2	17.82	2.36
−8	4	2	25.54	2.27
6	−4	−2	19.28	2.10
−6	4	2	11.96	1.59
4	−4	−2	299.47	9.45
4	−4	−2	284.99	7.71
−4	4	2	296.43	8.80
2	−4	−2	66.98	2.34
2	−4	−2	65.86	3.19
−2	4	2	69.83	2.83
0	−4	−2	42.32	2.39
0	−4	−2	46.23	1.88
0	4	2	43.60	1.89
−2	−4	−2	323.33	8.91
2	4	2	325.44	8.44
−4	−4	−2	75.05	3.25
4	4	2	77.11	2.55
−6	−4	−2	26.11	2.06
−6	−4	−2	29.53	1.58
−8	−4	−2	59.81	2.76
−8	−4	−2	62.11	3.51
−10	−4	−2	29.03	2.08
−10	−4	−2	30.75	2.92
−12	−4	−2	10.40	2.13
−14	−4	−2	2.80	1.67
−16	−4	−2	6.47	2.38
−17	5	2	1.48	1.52
−15	5	2	4.65	1.91
−13	5	2	6.07	1.78
−11	5	2	14.34	2.34
−9	5	2	9.66	1.84
7	−5	−2	43.83	3.15
−7	5	2	44.68	2.90
5	−5	−2	29.29	1.65
5	−5	−2	29.15	2.41
−5	5	2	33.98	2.22
3	−5	−2	71.28	3.63
3	−5	−2	72.92	2.73

−3	5	2	76.01	3.22
1	−5	−2	107.38	3.63
1	−5	−2	111.44	4.51
−1	−5	−2	23.98	1.54
−1	−5	−2	22.51	1.85
1	5	2	19.13	1.15
−3	−5	−2	17.86	1.60
3	5	2	23.90	1.11
−5	−5	−2	43.55	2.60
−5	−5	−2	41.33	1.71
−7	−5	−2	33.73	1.74
−7	−5	−2	33.75	2.56
−9	−5	−2	17.44	2.31
−11	−5	−2	7.88	1.97
−13	−5	−2	14.58	2.66
−15	−5	−2	2.29	1.76
−17	−5	−2	1.15	1.88
−16	6	2	0.63	1.29
−14	6	2	2.74	1.58
−12	6	2	6.36	1.80
−10	6	2	11.75	2.10
8	−6	−2	31.79	2.89
−8	6	2	34.92	2.75
6	−6	−2	9.41	1.90
6	−6	−2	12.13	1.30
−6	6	2	11.99	1.72
4	−6	−2	29.36	2.53
4	−6	−2	32.35	1.83
−4	6	2	32.25	2.17
2	−6	−2	15.08	1.24
2	−6	−2	13.54	1.81
0	−6	−2	30.33	1.89
0	−6	−2	28.26	2.31
0	6	2	35.11	1.74
−2	−6	−2	10.76	1.70
−2	−6	−2	8.32	1.29
2	6	2	12.15	0.83
−4	−6	−2	7.96	1.44
4	6	2	11.82	0.74
−6	−6	−2	20.29	2.24
−8	−6	−2	4.78	1.51
−10	−6	−2	8.95	2.00
−12	−6	−2	1.68	1.44
−14	−6	−2	0.26	1.47

−16	−6	−2	1.38	1.75
−15	7	2	1.51	1.41
−13	7	2	2.29	1.37
−11	7	2	5.99	1.76
−9	7	2	9.37	1.89
7	−7	−2	11.17	2.24
−7	7	2	6.79	1.58
5	−7	−2	21.70	1.69
5	−7	−2	23.76	2.69
−5	7	2	22.80	2.00
3	−7	−2	16.46	1.43
3	−7	−2	15.06	2.12
−3	7	2	18.25	1.62
1	−7	−2	19.13	2.20
1	−7	−2	17.71	1.53
−1	7	2	16.24	1.33
−1	−7	−2	11.61	1.87
−1	−7	−2	11.93	1.49
1	7	2	10.50	0.97
−3	−7	−2	12.79	1.71
−3	−7	−2	12.19	1.96
−5	−7	−2	8.68	1.66
−7	−7	−2	23.48	2.62
−9	−7	−2	6.04	1.83
−11	−7	−2	2.85	1.70
−13	−7	−2	3.25	1.75
−15	−7	−2	6.23	2.46
−14	8	2	1.25	1.38
−12	8	2	2.22	1.47
−10	8	2	3.94	1.48
8	−8	−2	11.42	2.37
−8	8	2	6.52	1.67
6	−8	−2	13.28	1.62
6	−8	−2	13.42	2.32
−6	8	2	10.86	1.80
4	−8	−2	20.38	1.71
4	−8	−2	20.15	2.58
−4	8	2	17.83	1.92
2	−8	−2	18.34	1.78
2	−8	−2	18.69	2.40
−2	8	2	16.58	1.60
0	−8	−2	9.68	1.48
0	−8	−2	10.09	1.96
−2	−8	−2	21.09	2.14

−2	−8	−2	24.83	2.64
−4	−8	−2	13.95	1.97
−4	−8	−2	12.95	2.20
−6	−8	−2	3.22	1.38
−8	−8	−2	6.80	1.84
−10	−8	−2	13.36	2.63
−12	−8	−2	1.13	1.66
−14	−8	−2	0.84	1.85
−13	9	2	4.05	1.71
−11	9	2	0.66	1.20
9	−9	−2	4.54	1.84
−9	9	2	7.11	1.87
7	−9	−2	7.85	2.07
−7	9	2	2.56	1.30
5	−9	−2	15.03	2.61
−5	9	2	15.92	1.99
3	−9	−2	13.26	2.32
3	−9	−2	12.90	1.66
−3	9	2	17.75	1.83
1	−9	−2	8.40	1.84
1	−9	−2	8.46	1.49
−1	−9	−2	23.70	2.38
−1	−9	−2	22.65	2.79
−3	−9	−2	19.53	2.63
−3	−9	−2	15.41	2.17
−5	−9	−2	16.83	2.63
−5	−9	−2	21.66	2.54
−7	−9	−2	5.94	1.81
−9	−9	−2	17.83	2.82
−11	−9	−2	8.66	2.46
−13	−9	−2	0.53	1.72
−12	10	2	2.22	1.52
10	−10	−2	3.12	1.77
−10	10	2	5.73	1.82
8	−10	−2	5.21	2.00
−8	10	2	7.96	1.90
6	−10	−2	3.59	1.77
−6	10	2	4.80	1.51
4	−10	−2	9.44	2.21
2	−10	−2	20.79	2.90
0	−10	−2	22.70	2.91
0	−10	−2	19.60	2.24
−2	−10	−2	31.05	3.41
−2	−10	−2	24.52	2.71

-4	-10	-2	17.25	2.44
-4	-10	-2	14.81	2.63
-6	-10	-2	21.11	2.79
-6	-10	-2	22.93	3.13
-8	-10	-2	4.15	1.74
-10	-10	-2	7.75	2.29
-12	-10	-2	10.28	2.70
-11	11	2	3.34	1.67
9	-11	-2	7.84	2.39
7	-11	-2	1.74	1.44
5	-11	-2	3.94	1.91
3	-11	-2	13.57	2.66
1	-11	-2	6.35	1.94
-1	-11	-2	4.66	1.90
-3	-11	-2	17.66	2.97
-3	-11	-2	16.27	2.57
-5	-11	-2	6.75	2.09
-5	-11	-2	8.85	2.34
-7	-11	-2	10.37	2.52
-7	-11	-2	10.04	2.27
-9	-11	-2	4.80	1.99
8	-12	-2	0.89	1.65
6	-12	-2	2.38	1.75
4	-12	-2	7.42	2.25
2	-12	-2	1.44	1.47
0	-12	-2	1.06	1.37
-2	-12	-2	1.58	1.55
-4	-12	-2	1.33	1.60
-6	-12	-2	2.24	1.84
-6	-12	-2	4.34	1.90
-8	-12	-2	-0.33	1.63
-8	-12	-2	0.24	1.53
5	-13	-2	-0.11	1.57
3	-13	-2	0.51	1.60
1	-13	-2	2.73	1.87
-1	-13	-2	-0.13	1.45
-3	-13	-2	0.11	1.43
-5	-13	-2	2.16	1.88
-3	-13	3	-1.02	1.59
-1	-13	3	-0.71	1.59
1	-13	3	0.98	1.80
3	-13	3	0.25	1.67
-8	-12	3	1.62	1.83
-6	-12	3	2.05	1.95

-4	-12	3	6.47	2.28
-2	-12	3	0.22	1.44
0	-12	3	-0.78	1.44
2	-12	3	1.57	1.61
4	-12	3	0.19	1.45
6	-12	3	3.63	1.90
-9	-11	3	1.82	1.91
-7	-11	3	1.43	1.64
-5	-11	3	2.95	1.90
-3	-11	3	13.48	2.76
-1	-11	3	2.32	1.57
1	-11	3	5.80	1.97
3	-11	3	10.10	2.30
5	-11	3	2.33	1.51
7	-11	3	3.08	1.72
9	-11	3	0.18	1.48
-12	-10	3	-0.44	1.59
-10	-10	3	6.61	2.44
-8	-10	3	6.61	2.27
-6	-10	3	7.13	2.25
-4	-10	3	23.99	2.71
-4	-10	3	20.40	3.11
-2	-10	3	24.25	3.13
0	-10	3	15.54	2.59
2	-10	3	19.76	2.80
4	-10	3	20.97	2.84
-6	10	-3	7.47	1.83
6	-10	3	8.67	2.17
-8	10	-3	3.32	1.62
8	-10	3	4.24	1.75
-10	10	-3	5.76	2.01
10	-10	3	4.07	1.95
-12	10	-3	2.47	1.58
-13	-9	3	6.14	2.14
-11	-9	3	8.34	2.31
-9	-9	3	13.05	2.68
-7	-9	3	21.99	3.12
-5	-9	3	13.39	2.50
-3	-9	3	47.15	3.69
-3	-9	3	37.35	2.83
-1	-9	3	15.09	1.95
-1	-9	3	16.08	2.49
1	-9	3	23.16	2.79
-3	9	-3	15.03	1.92

3	−9	3	13.50	2.26
−5	9	−3	15.87	2.24
5	−9	3	14.97	2.41
−7	9	−3	12.29	2.16
7	−9	3	14.60	2.42
−9	9	−3	10.61	2.26
9	−9	3	14.33	2.50
−11	9	−3	4.66	1.71
−13	9	−3	1.29	1.50
−14	−8	3	2.84	1.73
−12	−8	3	8.47	2.23
−10	−8	3	4.77	1.78
−8	−8	3	4.71	1.65
−6	−8	3	13.62	2.42
−4	−8	3	4.72	1.69
−2	−8	3	13.54	2.26
0	8	−3	28.14	1.97
0	−8	3	32.51	2.27
0	−8	3	33.80	2.81
2	−8	3	11.44	2.04
−4	8	−3	7.97	1.62
4	−8	3	4.80	1.48
−6	8	−3	4.82	1.45
6	−8	3	6.73	1.68
−8	8	−3	4.11	1.52
8	−8	3	4.11	1.57
−10	8	−3	4.53	1.73
−12	8	−3	0.81	1.39
−14	8	−3	6.53	2.23
−15	−7	3	0.26	1.27
−13	−7	3	3.66	1.75
−11	−7	3	2.19	1.50
−9	−7	3	0.40	1.05
−7	−7	3	4.47	1.57
−5	−7	3	12.75	2.15
−3	−7	3	10.37	1.94
1	7	−3	2.49	0.78
−1	−7	3	1.51	0.83
−1	−7	3	1.46	1.05
−1	7	−3	0.18	0.47
1	−7	3	0.23	0.67
1	−7	3	−0.04	0.80
−3	7	−3	2.97	1.02
3	−7	3	4.70	1.11

3	−7	3	2.44	1.14
−5	7	−3	3.69	1.21
5	−7	3	6.71	1.59
−7	7	−3	2.40	1.14
7	−7	3	3.62	1.36
−9	7	−3	2.38	1.28
9	−7	3	1.66	1.31
−11	7	−3	2.98	1.49
−13	7	−3	1.19	1.43
−15	7	−3	4.73	2.12
−16	−6	3	0.32	1.49
−14	−6	3	−0.04	1.22
−12	−6	3	0.65	1.37
−10	−6	3	0.13	1.10
−8	−6	3	8.36	1.85
−6	−6	3	1.07	1.03
−4	−6	3	4.24	0.68
−4	−6	3	1.61	1.00
−2	−6	3	0.98	0.96
−2	−6	3	1.41	0.57
0	6	−3	23.11	1.57
0	−6	3	20.20	1.49
0	−6	3	18.52	2.02
−2	6	−3	0.40	0.61
2	−6	3	1.21	0.85
2	−6	3	1.97	0.74
−4	6	−3	10.58	1.56
4	−6	3	9.36	1.70
4	−6	3	10.33	1.20
−6	6	−3	5.32	1.35
6	−6	3	4.76	1.37
−8	6	−3	0.92	0.97
8	−6	3	2.59	1.21
−10	6	−3	9.36	2.14
−12	6	−3	5.30	1.71
−14	6	−3	−0.59	1.00
−16	6	−3	2.69	1.72
−17	−5	3	−1.05	1.39
−15	−5	3	1.93	1.61
−13	−5	3	7.02	1.98
−11	−5	3	15.40	2.50
−9	−5	3	24.66	2.68
−7	−5	3	21.29	1.41
−7	−5	3	19.01	2.25

−5	−5	3	26.72	1.49
−5	−5	3	24.26	2.11
−3	−5	3	34.80	2.45
−3	−5	3	35.58	1.72
−1	−5	3	13.49	1.12
−1	−5	3	11.40	1.55
−1	5	−3	53.62	2.63
1	−5	3	56.44	3.03
1	−5	3	61.36	2.30
−3	5	−3	77.56	3.37
3	−5	3	71.07	2.72
3	−5	3	71.10	3.54
−5	5	−3	27.77	2.12
5	−5	3	26.78	2.23
−7	5	−3	14.89	2.04
7	−5	3	17.88	2.09
−9	5	−3	13.16	2.19
−11	5	−3	15.98	2.70
−13	5	−3	3.56	1.68
−15	5	−3	1.82	1.61
−18	−4	3	5.69	2.22
−16	−4	3	8.97	2.41
−14	−4	3	9.87	2.28
−12	−4	3	17.49	2.58
−10	−4	3	20.19	2.52
−10	−4	3	26.14	1.98
−8	−4	3	26.86	2.37
−8	−4	3	34.46	2.00
−6	−4	3	29.33	2.29
−6	−4	3	26.47	1.60
−4	−4	3	119.02	3.98
−4	−4	3	123.30	4.75
−2	−4	3	130.52	4.86
−2	−4	3	127.49	4.17
0	−4	3	116.04	4.43
0	−4	3	117.69	4.03
0	−4	3	120.12	3.62
2	−4	3	357.08	9.45
2	−4	3	356.43	10.14
2	−4	3	364.92	10.33
−4	4	−3	34.36	2.21
4	−4	3	25.98	2.06
−6	4	−3	41.75	2.66
6	−4	3	40.81	2.61

−8	4	−3	43.29	3.03
−10	4	−3	2.86	1.30
−12	4	−3	14.75	2.64
−14	4	−3	3.58	1.79
−16	4	−3	3.95	1.99
−17	−3	3	2.29	1.60
−15	−3	3	3.89	1.71
−13	−3	3	22.98	2.44
−13	−3	3	23.96	2.92
−11	−3	3	1.73	1.22
−11	−3	3	1.61	0.90
−9	−3	3	71.77	3.88
−9	−3	3	70.77	3.43
−7	−3	3	72.43	3.16
−7	−3	3	72.58	3.62
−5	−3	3	151.47	5.44
−5	−3	3	156.59	4.97
−3	−3	3	76.53	2.86
−3	−3	3	74.99	3.19
−1	−3	3	108.74	3.62
−1	−3	3	97.89	3.78
1	−3	3	294.13	8.50
1	−3	3	295.55	8.32
3	−3	3	41.79	2.26
3	−3	3	43.77	2.41
−5	3	−3	111.35	4.47
5	−3	3	116.48	4.50
−7	3	−3	101.69	4.53
−9	3	−3	23.66	2.58
−11	3	−3	19.30	2.68
−13	3	−3	4.48	1.69
−15	3	−3	4.70	1.99
−17	3	−3	0.14	1.78
−18	−2	3	3.63	1.81
−16	−2	3	2.40	1.52
−16	−2	3	3.39	1.65
−14	−2	3	7.45	1.90
−14	−2	3	9.44	1.94
−12	−2	3	52.38	3.38
−12	−2	3	52.21	3.43
−10	−2	3	13.07	1.88
−10	−2	3	14.80	1.83
−8	−2	3	73.92	3.50
−8	−2	3	83.72	3.84

−6	−2	3	80.25	3.44
−6	−2	3	72.81	3.20
−4	−2	3	16.20	1.35
−4	−2	3	18.37	1.32
−2	−2	3	207.68	5.99
−2	−2	3	199.57	6.00
0	−2	3	56.12	2.36
0	−2	3	57.89	2.40
2	−2	3	255.18	7.38
2	−2	3	251.94	7.33
−4	2	−3	46.95	2.56
4	−2	3	53.03	2.54
4	−2	3	53.77	2.58
−6	2	−3	55.23	2.95
−8	2	−3	36.95	2.65
−10	2	−3	12.66	2.15
−12	2	−3	14.07	2.41
−14	2	−3	3.31	1.74
−16	2	−3	6.25	2.13
−18	2	−3	4.20	2.25
−17	−1	3	1.58	1.45
−17	−1	3	4.61	1.82
−15	−1	3	5.48	1.88
−15	−1	3	4.87	1.64
−13	−1	3	16.11	2.29
−13	−1	3	13.41	2.12
−11	−1	3	30.72	2.53
−11	−1	3	32.39	2.61
−9	−1	3	10.25	1.59
−9	−1	3	9.67	1.51
−7	−1	3	18.28	1.68
−7	−1	3	17.61	1.74
−5	−1	3	185.33	6.01
−5	−1	3	184.78	5.72
−3	−1	3	9.22	0.89
−3	−1	3	8.68	1.02
−1	−1	3	52.54	2.08
−1	−1	3	56.59	2.30
−1	1	−3	6.94	0.50
1	−1	3	11.73	0.94
1	−1	3	10.75	1.02
3	−1	3	12.54	1.10
3	−1	3	11.83	1.03
−5	1	−3	6.05	1.12

-7	1	-3	28.38	2.10
-9	1	-3	13.15	1.95
-11	1	-3	4.48	1.43
-13	1	-3	9.89	2.20
-15	1	-3	5.00	1.91
-17	1	-3	1.62	1.73
-18	0	3	4.27	1.91
-18	0	3	4.55	1.87
-16	0	3	0.17	1.17
-16	0	3	-0.74	1.06
-14	0	3	0.24	1.15
-14	0	3	0.01	0.96
-12	0	3	50.07	3.20
-12	0	3	42.26	3.19
-10	0	3	0.50	0.82
-10	0	3	-0.05	0.75
-8	0	3	1.07	0.81
-8	0	3	-0.17	0.58
-6	0	3	22.51	1.76
-6	0	3	19.63	1.39
-4	0	3	11.52	1.25
-4	0	3	9.36	0.87
2	0	-3	6.65	0.80
-2	0	3	6.74	0.63
-2	0	3	7.14	0.92
0	0	-3	0.75	0.44
0	0	3	-0.04	0.21
0	0	3	0.39	0.36
2	0	3	0.47	0.35
4	0	3	77.53	3.08
-6	0	-3	0.54	0.66
-8	0	-3	5.14	1.22
-10	0	-3	5.20	1.51
-12	0	-3	5.56	1.63
-14	0	-3	12.08	2.47
-16	0	-3	0.42	1.42
-18	0	-3	-2.10	1.32
-17	1	3	2.73	1.63
-17	1	3	2.98	1.65
-15	1	3	3.47	1.59
-15	1	3	5.92	1.67
-13	1	3	12.63	1.94
-13	1	3	15.04	2.40
-11	1	3	22.70	2.51

−11	1	3	24.57	2.07
−9	1	3	7.70	1.22
−9	1	3	7.42	1.55
−7	1	3	6.34	0.89
−7	1	3	14.03	1.69
−5	1	3	160.22	4.70
−5	1	3	162.85	5.62
3	−1	−3	10.41	1.15
−3	1	3	7.27	0.53
−3	1	3	5.50	0.94
1	−1	−3	52.85	2.18
−1	1	3	43.56	2.03
−1	1	3	42.47	1.37
−1	−1	−3	10.97	0.91
1	1	3	8.97	0.92
3	1	3	12.40	1.04
5	1	3	5.93	0.98
−7	−1	−3	29.21	2.05
−9	−1	−3	20.62	2.11
−11	−1	−3	3.58	1.38
−13	−1	−3	14.57	2.42
−15	−1	−3	5.90	1.88
−17	−1	−3	1.80	1.37
−17	−1	−3	−0.19	1.39
−18	2	3	3.98	1.98
−16	2	3	1.56	1.41
−16	2	3	2.76	1.66
−14	2	3	5.80	1.59
−14	2	3	5.82	1.73
−12	2	3	43.89	3.40
−12	2	3	44.87	2.92
−10	2	3	14.66	1.68
−10	2	3	12.25	1.99
−8	2	3	68.73	3.66
−6	2	3	63.79	2.45
−6	2	3	63.33	3.29
−4	2	3	10.37	0.66
−4	2	3	7.71	1.15
2	−2	−3	203.05	6.47
−2	2	3	196.14	6.13
0	−2	−3	59.10	2.56
0	2	3	55.80	2.37
2	2	3	230.11	6.55
4	2	3	50.00	2.22

−6	−2	−3	64.35	3.00
−8	−2	−3	33.75	2.27
−10	−2	−3	14.10	1.92
−12	−2	−3	14.29	2.12
−14	−2	−3	4.96	1.67
−14	−2	−3	6.47	1.92
−16	−2	−3	5.62	2.18
−18	−2	−3	3.29	1.77
−17	3	3	4.15	2.04
−15	3	3	3.12	1.54
−13	3	3	21.95	2.93
−11	3	3	2.21	1.20
−9	3	3	63.93	3.74
−7	3	3	61.67	3.38
5	−3	−3	160.61	6.12
−5	3	3	157.40	5.77
3	−3	−3	86.96	3.89
−3	3	3	87.00	3.54
1	−3	−3	105.03	2.79
1	−3	−3	102.07	3.99
−1	3	3	102.40	3.68
−1	−3	−3	279.23	8.74
−1	−3	−3	299.81	7.71
1	3	3	302.81	8.17
−3	−3	−3	42.75	2.07
−7	−3	−3	113.04	4.72
−7	−3	−3	109.75	4.22
−9	−3	−3	27.90	2.12
−9	−3	−3	29.65	2.52
−11	−3	−3	24.26	2.37
−11	−3	−3	21.20	2.72
−13	−3	−3	5.28	1.56
−13	−3	−3	3.14	1.48
−15	−3	−3	4.88	1.93
−17	−3	−3	1.94	1.81
−18	4	3	3.67	1.95
−16	4	3	4.86	2.06
−14	4	3	9.49	2.21
−12	4	3	18.30	2.69
−10	4	3	22.83	2.66
−8	4	3	36.74	2.78
6	−4	−3	29.88	2.55
−6	4	3	29.98	2.30
4	−4	−3	129.31	5.47

−4	4	3	138.21	5.11
2	−4	−3	135.10	5.23
2	−4	−3	128.70	3.61
−2	4	3	135.64	4.72
0	−4	−3	124.42	3.68
0	−4	−3	119.26	4.73
0	4	3	120.70	4.13
−2	−4	−3	365.16	11.04
−2	−4	−3	365.58	9.77
2	4	3	368.12	9.80
−4	−4	−3	28.04	1.96
4	4	3	33.21	1.63
−6	−4	−3	43.00	2.24
−6	−4	−3	41.31	2.57
−8	−4	−3	41.83	2.85
−8	−4	−3	39.52	2.28
−10	−4	−3	2.46	1.19
−10	−4	−3	2.44	0.99
−12	−4	−3	12.87	2.34
−14	−4	−3	3.74	1.67
−16	−4	−3	2.31	1.77
−17	5	3	0.81	1.55
−15	5	3	2.86	1.67
−13	5	3	8.88	2.13
−11	5	3	15.08	2.38
−9	5	3	25.17	2.72
7	−5	−3	18.76	2.41
−7	5	3	21.81	2.28
5	−5	−3	24.52	2.27
−5	5	3	25.00	2.06
3	−5	−3	35.88	1.56
3	−5	−3	30.33	2.39
−3	5	3	37.02	2.22
1	−5	−3	12.48	1.00
1	−5	−3	12.51	1.70
−1	5	3	16.96	1.34
−1	−5	−3	62.81	3.28
−1	−5	−3	58.59	2.51
1	5	3	53.75	2.39
−3	−5	−3	79.31	3.44
3	5	3	84.27	2.93
−5	−5	−3	26.67	2.09
5	5	3	29.42	1.55
−7	−5	−3	19.93	2.17

-7	-5	-3	17.79	1.41
-9	-5	-3	8.40	1.86
-11	-5	-3	13.79	2.49
-13	-5	-3	2.82	1.69
-15	-5	-3	0.78	1.47
-16	6	3	2.16	1.52
-14	6	3	3.01	1.51
-12	6	3	1.04	1.21
-10	6	3	0.29	1.03
8	-6	-3	11.03	2.19
-8	6	3	9.29	1.78
6	-6	-3	1.87	1.20
-6	6	3	1.37	0.89
4	-6	-3	2.83	1.31
4	-6	-3	3.72	0.75
-4	6	3	3.98	1.10
2	-6	-3	1.74	0.62
2	-6	-3	1.14	1.01
-2	6	3	2.33	0.77
0	-6	-3	16.79	1.35
0	-6	-3	19.83	2.20
0	6	3	22.73	1.51
-2	-6	-3	1.52	0.99
-2	-6	-3	2.31	0.87
2	6	3	0.97	0.46
-4	-6	-3	9.28	1.51
4	6	3	11.09	0.93
-6	-6	-3	6.28	1.42
-8	-6	-3	1.73	1.14
-10	-6	-3	8.44	1.94
-12	-6	-3	6.27	2.00
-14	-6	-3	0.98	1.55
-16	-6	-3	0.75	1.74
-15	7	3	1.07	1.48
-13	7	3	1.06	1.32
-11	7	3	4.03	1.58
9	-7	-3	0.66	1.19
-9	7	3	0.58	1.07
7	-7	-3	4.62	1.71
-7	7	3	1.84	1.04
5	-7	-3	13.60	2.34
-5	7	3	13.96	1.83
3	-7	-3	8.62	1.77
3	-7	-3	8.69	1.07

-3	7	3	7.15	1.26
1	-7	-3	0.81	0.97
1	-7	-3	0.55	0.61
-1	7	3	2.13	0.70
-1	-7	-3	0.01	0.84
-1	-7	-3	0.67	0.73
1	7	3	0.52	0.49
-3	-7	-3	5.38	1.49
-3	-7	-3	4.72	1.21
-5	-7	-3	8.13	1.62
-7	-7	-3	4.87	1.58
-9	-7	-3	2.35	1.46
-11	-7	-3	2.76	1.61
-13	-7	-3	0.30	1.54
-15	-7	-3	4.36	2.16
-14	8	3	2.97	1.65
-12	8	3	5.68	1.85
-10	8	3	3.92	1.51
8	-8	-3	5.16	1.88
-8	8	3	4.33	1.54
6	-8	-3	15.23	2.51
-6	8	3	14.67	2.05
4	-8	-3	4.05	1.47
-4	8	3	5.83	1.30
2	-8	-3	11.22	2.17
2	-8	-3	12.77	1.45
-2	8	3	11.38	1.45
0	-8	-3	29.53	3.03
0	-8	-3	34.77	2.24
-2	-8	-3	9.63	1.53
-2	-8	-3	8.98	1.91
-4	-8	-3	3.33	1.30
-4	-8	-3	4.60	1.62
-6	-8	-3	4.24	1.50
-8	-8	-3	4.68	1.69
-10	-8	-3	5.91	1.99
-12	-8	-3	4.49	2.13
-14	-8	-3	6.24	2.44
-13	9	3	1.93	1.60
-11	9	3	6.30	1.81
9	-9	-3	17.86	3.06
-9	9	3	14.27	2.44
7	-9	-3	27.26	3.39
-7	9	3	22.97	2.67

5	-9	-3	16.42	2.72
-5	9	3	10.92	1.78
3	-9	-3	42.59	3.53
1	-9	-3	11.70	2.18
-1	-9	-3	22.46	2.90
-1	-9	-3	22.17	2.22
-3	-9	-3	19.60	2.76
-3	-9	-3	14.66	2.01
-5	-9	-3	15.05	2.19
-5	-9	-3	12.18	2.41
-7	-9	-3	12.19	2.27
-9	-9	-3	11.64	2.36
-11	-9	-3	2.97	1.92
-13	-9	-3	1.23	1.88
-12	10	3	3.09	1.60
10	-10	-3	4.96	1.95
-10	10	3	5.44	1.85
8	-10	-3	7.49	2.33
-8	10	3	8.32	1.96
6	-10	-3	4.51	1.83
4	-10	-3	24.09	3.25
2	-10	-3	23.09	3.11
0	-10	-3	17.32	2.79
-2	-10	-3	18.04	2.93
-4	-10	-3	21.54	3.12
-4	-10	-3	20.58	2.55
-6	-10	-3	7.22	2.11
-6	-10	-3	8.65	2.01
-8	-10	-3	1.35	1.56
-10	-10	-3	6.08	2.15
9	-11	-3	1.71	1.65
7	-11	-3	3.24	1.82
5	-11	-3	4.47	1.93
3	-11	-3	7.63	2.21
1	-11	-3	4.35	1.82
-1	-11	-3	6.35	2.04
-3	-11	-3	7.41	2.24
-5	-11	-3	4.22	1.83
-7	-11	-3	4.20	1.76
-7	-11	-3	4.19	1.91
-9	-11	-3	0.80	1.49
-9	-11	-3	0.37	1.63
8	-12	-3	2.04	1.87
6	-12	-3	2.49	1.83

4	-12	-3	6.49	2.31
2	-12	-3	-0.62	1.33
0	-12	-3	0.56	1.39
-2	-12	-3	2.10	1.70
-4	-12	-3	0.11	1.39
-6	-12	-3	2.00	1.74
3	-13	-3	0.29	1.52
1	-13	-3	-0.04	1.44
-1	-13	-3	0.04	1.35
-3	-13	-3	0.55	1.60
-1	-13	4	1.32	1.84
-6	-12	4	2.40	2.04
-4	-12	4	2.71	1.90
-2	-12	4	2.13	1.86
0	-12	4	2.17	1.81
2	-12	4	3.82	1.94
4	-12	4	0.85	1.65
6	-12	4	1.64	1.70
-9	-11	4	2.38	2.07
-7	-11	4	3.03	2.14
-5	-11	4	3.18	2.00
-3	-11	4	9.19	2.53
-1	-11	4	1.26	1.50
1	-11	4	0.16	1.24
3	-11	4	4.45	1.82
5	-11	4	0.48	1.48
7	-11	4	2.23	1.63
9	-11	4	-0.54	1.45
-12	-10	4	4.68	1.95
-10	-10	4	5.92	2.03
-8	-10	4	1.44	1.65
-6	-10	4	2.97	2.02
-4	-10	4	5.22	2.00
-2	-10	4	9.27	2.30
0	-10	4	4.71	1.88
2	-10	4	5.01	1.86
4	-10	4	6.97	2.02
-6	10	-4	0.56	1.21
6	-10	4	0.37	1.28
-8	10	-4	0.69	1.31
8	-10	4	1.43	1.47
-10	10	-4	1.40	1.38
10	-10	4	0.66	1.53
-13	-9	4	2.62	1.89

-11	-9	4	5.35	1.97
-9	-9	4	11.03	2.48
-7	-9	4	11.61	2.45
-5	-9	4	7.86	2.18
-3	-9	4	22.39	2.96
-1	-9	4	19.72	2.75
1	-9	4	6.64	1.90
-3	9	-4	19.67	2.31
3	-9	4	24.93	2.99
-5	9	-4	2.11	1.28
5	-9	4	1.06	1.24
-7	9	-4	1.44	1.28
7	-9	4	1.71	1.40
-9	9	-4	4.21	1.65
9	-9	4	2.17	1.53
-11	9	-4	2.20	1.59
-14	-8	4	3.51	1.77
-12	-8	4	6.13	1.99
-10	-8	4	4.14	1.74
-8	-8	4	14.14	2.59
-6	-8	4	9.19	2.15
-4	-8	4	6.35	1.74
-2	-8	4	51.38	3.70
0	8	-4	19.09	1.86
0	-8	4	19.85	2.55
-2	8	-4	1.80	0.96
2	-8	4	3.60	1.41
-4	8	-4	16.53	2.14
4	-8	4	16.56	2.41
-6	8	-4	18.94	2.48
6	-8	4	17.99	2.63
-8	8	-4	6.59	1.79
8	-8	4	4.98	1.62
-10	8	-4	3.28	1.65
-12	8	-4	5.87	2.07
-15	-7	4	-1.26	1.21
-13	-7	4	1.50	1.60
-11	-7	4	3.08	1.49
-9	-7	4	3.15	1.56
-7	-7	4	10.27	2.05
-5	-7	4	6.14	1.68
-3	-7	4	4.05	0.87
-3	-7	4	1.53	1.11
1	7	-4	15.00	1.59

−1	−7	4	14.64	1.47
−1	−7	4	11.56	1.94
−1	7	−4	2.36	0.97
1	−7	4	2.41	1.16
−3	7	−4	33.23	2.44
3	−7	4	37.98	2.97
−5	7	−4	14.58	2.01
5	−7	4	10.48	2.07
−7	7	−4	14.47	2.29
7	−7	4	8.99	1.94
−9	7	−4	21.48	2.90
9	−7	4	19.85	2.85
−11	7	−4	3.51	1.69
−13	7	−4	4.27	1.86
−16	−6	4	1.47	1.51
−14	−6	4	−0.29	1.27
−12	−6	4	10.50	2.31
−10	−6	4	0.89	1.22
−8	−6	4	1.40	1.10
−6	−6	4	1.54	1.19
−4	−6	4	35.77	1.94
−4	−6	4	35.68	2.84
−2	−6	4	9.42	1.18
−2	−6	4	9.89	1.72
0	−6	4	0.30	0.57
0	−6	4	2.07	0.67
0	−6	4	0.24	0.75
−2	6	−4	56.96	3.05
2	−6	4	59.89	3.43
−4	6	−4	14.15	1.87
4	−6	4	12.63	1.97
−6	6	−4	4.84	1.41
6	−6	4	5.39	1.47
−8	6	−4	12.58	2.16
8	−6	4	14.05	2.25
−10	6	−4	8.07	2.05
−12	6	−4	5.43	2.00
−14	6	−4	2.19	1.64
−17	−5	4	1.62	1.48
−15	−5	4	3.65	1.81
−13	−5	4	8.50	2.15
−11	−5	4	6.47	1.85
−9	−5	4	12.25	2.11
−7	−5	4	17.02	1.45

−7	−5	4	19.02	2.24
−5	−5	4	13.74	1.91
−5	−5	4	13.43	1.32
−3	−5	4	19.63	1.46
−3	−5	4	23.07	2.26
−1	−5	4	50.71	2.49
−1	−5	4	52.32	2.98
1	−5	4	89.33	2.79
1	−5	4	82.74	4.01
1	−5	4	84.29	3.63
−3	5	−4	35.70	2.38
3	−5	4	32.67	2.41
−5	5	−4	16.05	1.98
5	−5	4	17.66	2.10
−7	5	−4	21.17	2.36
7	−5	4	24.93	2.56
−9	5	−4	6.33	1.73
−11	5	−4	13.81	2.58
−13	5	−4	6.32	2.09
−15	5	−4	−0.72	1.52
−16	−4	4	7.00	2.19
−14	−4	4	3.50	1.69
−12	−4	4	15.05	2.49
−10	−4	4	17.60	1.87
−10	−4	4	15.91	2.31
−8	−4	4	33.84	2.71
−8	−4	4	35.53	2.23
−6	−4	4	76.00	3.79
−6	−4	4	76.14	3.25
−4	−4	4	45.95	2.33
−4	−4	4	49.31	2.80
−2	−4	4	55.58	2.95
−2	−4	4	56.44	2.61
0	−4	4	45.54	1.62
0	−4	4	44.92	2.56
0	−4	4	49.25	2.41
2	−4	4	85.91	3.62
2	−4	4	83.38	3.80
−4	4	−4	26.79	2.13
4	−4	4	31.12	2.28
−6	4	−4	29.64	2.33
6	−4	4	29.01	2.33
−8	4	−4	25.23	2.63
−10	4	−4	15.57	2.45

−12	4	−4	4.24	1.78
−14	4	−4	1.18	1.59
−16	4	−4	1.23	1.83
−17	−3	4	1.68	1.63
−15	−3	4	0.34	1.38
−13	−3	4	7.47	1.97
−13	−3	4	9.88	1.93
−11	−3	4	13.42	1.96
−11	−3	4	11.93	2.11
−9	−3	4	21.78	2.04
−9	−3	4	18.34	2.22
−7	−3	4	77.30	3.52
−7	−3	4	76.43	3.78
−5	−3	4	35.09	2.07
−5	−3	4	34.25	2.24
−3	−3	4	184.37	6.07
−3	−3	4	179.59	5.82
−1	−3	4	81.46	3.43
−1	−3	4	85.07	3.36
1	−3	4	105.49	3.97
1	−3	4	107.89	4.12
3	−3	4	10.20	1.37
3	−3	4	10.13	1.43
−5	3	−4	63.55	3.32
5	−3	4	57.11	3.07
−7	3	−4	28.10	2.38
−9	3	−4	1.56	1.15
−11	3	−4	5.34	1.81
−13	3	−4	1.65	1.42
−15	3	−4	1.62	1.78
−17	3	−4	0.96	1.85
−18	−2	4	3.06	1.77
−16	−2	4	0.37	1.47
−16	−2	4	2.56	1.44
−14	−2	4	2.91	1.49
−14	−2	4	3.29	1.47
−12	−2	4	29.82	2.96
−12	−2	4	25.87	2.66
−10	−2	4	20.18	2.29
−10	−2	4	17.61	2.12
−8	−2	4	22.42	1.93
−8	−2	4	23.24	2.04
−6	−2	4	31.13	2.08
−6	−2	4	28.53	1.99

−4	−2	4	44.42	2.36
−4	−2	4	46.37	2.35
−2	−2	4	41.08	2.15
−2	−2	4	39.63	2.07
0	−2	4	24.15	1.60
0	−2	4	25.99	1.66
2	−2	4	128.92	4.67
2	−2	4	140.19	4.77
4	−2	4	29.04	1.94
4	−2	4	34.00	2.15
−6	2	−4	5.40	1.26
−8	2	−4	16.67	2.08
−10	2	−4	2.30	1.33
−12	2	−4	1.05	1.31
−14	2	−4	3.18	1.73
−16	2	−4	4.40	2.05
−17	−1	4	0.69	1.47
−17	−1	4	0.30	1.44
−15	−1	4	8.49	2.00
−15	−1	4	5.40	1.74
−13	−1	4	12.85	2.18
−13	−1	4	10.15	2.00
−11	−1	4	29.76	2.53
−11	−1	4	34.29	2.74
−9	−1	4	0.94	0.82
−9	−1	4	0.57	0.83
−7	−1	4	49.90	2.93
−7	−1	4	55.67	2.80
−5	−1	4	50.38	2.64
−5	−1	4	49.48	2.37
−3	−1	4	26.98	1.52
−1	−1	4	50.58	2.43
−1	−1	4	49.00	2.13
−1	1	−4	89.43	2.74
1	−1	4	83.34	3.18
1	−1	4	90.03	3.51
3	−1	4	96.42	3.66
3	−1	4	102.40	3.87
5	−1	4	26.89	1.98
−7	1	−4	34.77	2.47
−9	1	−4	44.64	3.11
−11	1	−4	6.21	1.70
−13	1	−4	11.08	2.40
−15	1	−4	8.95	2.45

−17	1	−4	2.42	1.83
−18	0	4	6.33	2.13
−16	0	4	−0.19	1.14
−16	0	4	−0.30	1.24
−14	0	4	4.51	1.70
−14	0	4	4.59	1.58
−12	0	4	14.48	2.00
−12	0	4	16.30	2.40
−10	0	4	8.57	1.48
−10	0	4	8.52	1.71
−8	0	4	68.84	3.32
−8	0	4	96.68	4.27
−6	0	4	135.83	4.50
−6	0	4	134.03	5.25
−4	0	4	0.44	0.43
−4	0	4	1.01	0.63
2	0	−4	40.86	2.03
−2	0	4	42.42	1.69
−2	0	4	45.20	2.31
0	0	−4	44.99	1.93
0	0	4	42.60	2.20
0	0	4	44.01	1.82
−2	0	−4	77.47	2.72
2	0	4	73.55	3.11
4	0	4	26.19	1.77
6	0	4	164.15	5.89
−8	0	−4	44.79	2.89
−10	0	−4	28.42	2.78
−12	0	−4	37.96	3.25
−14	0	−4	7.29	2.03
−16	0	−4	2.22	1.71
−17	1	4	1.05	1.46
−15	1	4	4.79	1.68
−15	1	4	5.47	1.70
−13	1	4	10.36	2.18
−13	1	4	7.78	1.71
−11	1	4	33.09	2.40
−11	1	4	37.20	3.07
−9	1	4	−0.19	0.69
−9	1	4	0.17	0.60
−7	1	4	40.57	2.73
−7	1	4	43.98	2.14
−5	1	4	45.38	2.68
−5	1	4	46.64	1.89

3	−1	−4	50.21	2.66
−3	1	4	58.91	1.82
−3	1	4	56.08	2.77
1	−1	−4	47.31	2.33
−1	1	4	47.64	2.38
−1	1	4	50.82	1.66
−1	−1	−4	80.87	3.14
1	1	4	85.76	3.29
3	1	4	98.00	3.71
5	1	4	27.70	1.87
−7	−1	−4	34.60	2.37
−9	−1	−4	41.16	2.82
−11	−1	−4	8.23	1.81
−13	−1	−4	16.57	2.64
−15	−1	−4	6.74	2.08
−17	−1	−4	1.53	1.84
−18	2	4	6.24	2.25
−16	2	4	1.91	1.67
−14	2	4	3.02	1.54
−12	2	4	25.73	3.04
−10	2	4	18.63	1.79
−10	2	4	17.50	2.39
−8	2	4	20.56	1.55
−8	2	4	17.29	2.21
−6	2	4	25.19	2.12
−6	2	4	33.29	1.53
4	−2	−4	50.83	2.95
−4	2	4	55.30	2.90
2	−2	−4	40.01	2.39
−2	2	4	42.24	2.31
0	−2	−4	21.56	1.58
0	2	4	22.64	1.56
−2	−2	−4	131.01	4.66
2	2	4	128.98	4.45
4	2	4	26.84	1.69
6	2	4	3.66	0.93
−8	−2	−4	21.87	2.10
−10	−2	−4	1.72	1.05
−12	−2	−4	3.60	1.51
−14	−2	−4	3.16	1.52
−16	−2	−4	2.94	2.04
−17	3	4	1.91	1.74
−15	3	4	0.94	1.40
−13	3	4	8.36	2.06

−11	3	4	11.40	2.22
−9	3	4	18.93	1.54
−9	3	4	16.41	2.27
−7	3	4	82.86	4.18
−7	3	4	79.69	2.85
5	−3	−4	36.64	2.71
−5	3	4	38.47	1.34
−5	3	4	39.40	2.55
3	−3	−4	184.46	6.76
−3	3	4	189.11	6.36
1	−3	−4	84.79	3.83
−1	3	4	92.42	3.61
−1	−3	−4	111.41	3.42
−1	−3	−4	110.56	4.42
1	3	4	108.68	4.00
3	3	4	11.06	1.21
5	3	4	68.22	2.98
−7	−3	−4	27.37	2.05
−9	−3	−4	2.75	1.04
−11	−3	−4	4.67	1.47
−13	−3	−4	1.14	1.33
−13	−3	−4	2.72	1.38
−15	−3	−4	1.72	1.49
−17	−3	−4	2.32	1.78
−16	4	4	3.22	1.90
−14	4	4	6.88	2.07
−12	4	4	9.66	2.12
−10	4	4	15.80	2.45
−8	4	4	40.35	3.03
6	−4	−4	83.85	4.52
−6	4	4	78.98	4.05
4	−4	−4	50.77	3.25
−4	4	4	48.62	2.85
2	−4	−4	50.06	3.04
−2	4	4	54.64	2.73
0	−4	−4	46.26	1.77
0	−4	−4	41.69	2.67
0	4	4	42.80	2.27
−2	−4	−4	83.71	3.19
−2	−4	−4	88.07	3.99
2	4	4	90.23	3.40
6	4	4	14.50	1.27
−8	−4	−4	28.48	2.10
−8	−4	−4	28.09	2.44

−10	−4	−4	9.16	1.89
−10	−4	−4	11.08	1.69
−12	−4	−4	5.06	1.77
−14	−4	−4	1.35	1.55
−16	−4	−4	0.91	1.66
−17	5	4	−0.16	1.44
−15	5	4	4.55	1.96
−13	5	4	11.40	2.52
−11	5	4	4.33	1.60
−9	5	4	12.08	2.16
7	−5	−4	18.56	2.52
−7	5	4	17.72	2.25
5	−5	−4	12.12	2.01
−5	5	4	12.81	1.77
3	−5	−4	23.30	2.37
−3	5	4	19.41	1.73
1	−5	−4	54.23	1.94
1	−5	−4	51.76	3.11
−1	5	4	50.38	2.66
−1	−5	−4	90.50	4.34
−1	−5	−4	87.01	3.20
−3	−5	−4	30.98	1.97
−3	−5	−4	30.52	2.53
3	5	4	37.26	1.91
−5	−5	−4	19.63	1.93
5	5	4	16.93	1.35
−7	−5	−4	24.00	2.45
−7	−5	−4	22.90	1.84
−9	−5	−4	5.57	1.59
−11	−5	−4	17.69	2.67
−13	−5	−4	4.75	1.92
−15	−5	−4	−1.46	1.35
−16	6	4	2.33	1.60
−14	6	4	−0.14	1.25
−12	6	4	6.06	1.92
−10	6	4	1.24	1.17
8	−6	−4	2.55	1.40
−8	6	4	2.79	1.21
6	−6	−4	1.57	1.23
−6	6	4	1.34	0.95
4	−6	−4	36.31	3.06
−4	6	4	37.54	2.57
2	−6	−4	12.93	2.08
−2	6	4	15.84	1.63

0	−6	−4	1.54	1.15
0	−6	−4	0.87	0.59
0	6	4	−0.01	0.29
−2	−6	−4	61.10	2.86
−2	−6	−4	62.33	3.75
2	6	4	63.48	2.78
−4	−6	−4	12.22	1.63
−4	−6	−4	10.39	1.94
4	6	4	12.57	1.12
−6	−6	−4	5.50	1.35
−8	−6	−4	11.11	2.00
−10	−6	−4	9.45	2.16
−12	−6	−4	9.62	2.37
−14	−6	−4	2.39	1.80
−15	7	4	3.54	1.84
−13	7	4	3.02	1.60
−11	7	4	1.39	1.36
9	−7	−4	6.10	1.93
−9	7	4	6.06	1.75
7	−7	−4	7.88	2.05
−7	7	4	11.59	1.99
5	−7	−4	4.29	1.60
−5	7	4	2.84	1.08
3	−7	−4	2.93	1.40
−3	7	4	5.21	1.21
1	−7	−4	15.46	2.23
−1	−7	−4	3.18	1.39
−1	−7	−4	1.45	0.82
1	7	4	3.02	0.82
−3	−7	−4	32.92	2.96
−3	−7	−4	29.84	2.20
−5	−7	−4	8.50	1.95
−5	−7	−4	12.74	1.83
−7	−7	−4	12.00	2.05
−9	−7	−4	20.06	2.68
−11	−7	−4	1.55	1.51
−13	−7	−4	6.39	2.21
−14	8	4	0.85	1.40
−12	8	4	4.68	1.71
10	−8	−4	1.69	1.48
−10	8	4	5.81	1.80
8	−8	−4	15.23	2.84
−8	8	4	15.05	2.38
6	−8	−4	6.12	1.85

−6	8	4	6.12	1.47
4	−8	−4	6.66	1.85
−4	8	4	8.85	1.56
0	−8	−4	15.83	2.45
−2	−8	−4	1.75	0.94
−2	−8	−4	4.01	1.62
−4	−8	−4	11.41	1.78
−4	−8	−4	14.46	2.46
−6	−8	−4	17.38	2.31
−6	−8	−4	21.21	2.98
−8	−8	−4	7.44	1.93
−10	−8	−4	3.08	1.72
−12	−8	−4	7.13	2.29
−13	9	4	1.23	1.44
−11	9	4	4.27	1.85
9	−9	−4	6.52	2.00
−9	9	4	6.46	1.86
7	−9	−4	12.30	2.55
−7	9	4	8.40	1.86
5	−9	−4	5.89	2.05
−5	9	4	7.32	1.65
3	−9	−4	22.87	3.02
1	−9	−4	13.79	2.44
−1	−9	−4	7.81	2.03
−3	−9	−4	22.70	3.07
−5	−9	−4	0.61	1.14
−5	−9	−4	1.44	1.32
−7	−9	−4	2.76	1.53
−7	−9	−4	2.00	1.66
−9	−9	−4	2.34	1.62
−11	−9	−4	1.27	1.70
−12	10	4	1.56	1.60
10	−10	−4	5.01	2.05
−10	10	4	4.53	1.70
8	−10	−4	4.33	1.98
−8	10	4	2.11	1.37
6	−10	−4	0.26	1.31
4	−10	−4	8.32	2.31
2	−10	−4	7.57	2.15
0	−10	−4	4.65	1.77
−2	−10	−4	4.20	1.84
−4	−10	−4	5.91	2.01
−6	−10	−4	−0.67	1.11
−8	−10	−4	0.95	1.50

-8	-10	-4	1.14	1.65
-10	-10	-4	0.46	1.66
9	-11	-4	1.87	1.82
7	-11	-4	2.98	1.81
5	-11	-4	4.41	1.96
3	-11	-4	4.94	1.92
1	-11	-4	2.34	1.56
-1	-11	-4	0.68	1.50
-3	-11	-4	3.72	1.72
-5	-11	-4	-0.05	1.24
-7	-11	-4	-0.72	1.29
-9	-11	-4	-0.86	1.44
6	-12	-4	2.37	1.84
4	-12	-4	0.85	1.54
2	-12	-4	1.66	1.64
0	-12	-4	2.37	1.77
-2	-12	-4	3.82	1.84
-4	-12	-4	0.68	1.49
-6	-12	-4	1.67	1.87
1	-13	-4	-0.02	1.79
-4	-12	5	6.39	2.43
-2	-12	5	3.87	2.18
0	-12	5	4.59	2.18
2	-12	5	4.39	2.15
4	-12	5	6.24	2.39
-9	-11	5	3.77	2.01
-7	-11	5	3.25	1.88
-5	-11	5	1.18	1.88
-3	-11	5	3.94	2.03
-1	-11	5	4.92	2.14
1	-11	5	0.15	1.52
3	-11	5	2.99	1.75
5	-11	5	1.75	1.70
7	-11	5	1.17	1.66
-10	-10	5	2.46	1.73
-8	-10	5	0.51	1.47
-6	-10	5	4.47	1.78
-4	-10	5	1.64	1.53
-2	-10	5	-1.14	1.31
0	-10	5	0.82	1.44
2	-10	5	1.24	1.31
4	-10	5	0.34	1.42
-6	10	-5	0.82	1.43
6	-10	5	1.46	1.52

−8	10	−5	0.21	1.45
8	−10	5	0.55	1.44
−10	10	−5	0.91	1.37
10	−10	5	−0.26	1.45
−11	−9	5	1.24	1.56
−9	−9	5	0.65	1.40
−7	−9	5	3.69	1.75
−5	−9	5	4.45	1.73
−3	−9	5	12.73	2.41
−1	−9	5	0.24	1.25
1	−9	5	1.77	1.39
−3	9	−5	0.89	1.09
3	−9	5	−0.27	1.19
−5	9	−5	3.61	1.48
5	−9	5	1.87	1.51
−7	9	−5	−0.04	1.11
7	−9	5	1.05	1.41
−9	9	−5	0.38	1.22
9	−9	5	−0.96	1.10
−11	9	−5	3.95	1.86
−14	−8	5	1.30	1.75
−12	−8	5	2.36	1.58
−10	−8	5	4.71	1.82
−8	−8	5	6.40	1.96
−6	−8	5	4.00	1.66
−4	−8	5	5.83	1.75
−2	−8	5	39.45	3.34
0	−8	5	9.79	2.06
−2	8	−5	3.57	1.27
2	−8	5	4.46	1.55
−4	8	−5	26.21	2.64
4	−8	5	18.69	2.72
−6	8	−5	1.84	1.22
6	−8	5	2.55	1.41
−8	8	−5	5.25	1.69
8	−8	5	5.32	1.81
−10	8	−5	4.85	1.80
−12	8	−5	4.30	1.87
−15	−7	5	2.33	1.73
−13	−7	5	2.96	1.63
−11	−7	5	8.90	2.23
−9	−7	5	10.38	2.22
−7	−7	5	16.52	2.56
−5	−7	5	16.51	2.55

−3	−7	5	48.40	3.42
−3	−7	5	43.98	2.52
−1	−7	5	15.54	1.76
−1	−7	5	13.64	2.07
−1	7	−5	19.88	2.09
1	−7	5	19.80	2.45
−3	7	−5	69.29	3.76
3	−7	5	64.78	4.05
−5	7	−5	30.09	2.63
5	−7	5	32.33	3.27
−7	7	−5	15.18	2.39
7	−7	5	12.11	2.28
−9	7	−5	10.95	2.42
9	−7	5	13.94	2.53
−11	7	−5	11.37	2.57
−13	7	−5	4.85	1.97
−14	−6	5	4.62	1.88
−12	−6	5	7.11	1.99
−10	−6	5	4.66	1.72
−8	−6	5	11.49	2.10
−6	−6	5	26.00	2.83
−6	−6	5	28.39	1.91
−4	−6	5	21.49	2.54
−4	−6	5	21.67	1.70
−2	−6	5	57.94	3.54
−2	−6	5	53.22	2.78
0	−6	5	49.27	2.76
0	−6	5	51.19	3.32
−2	6	−5	57.98	3.33
2	−6	5	55.69	3.45
−4	6	−5	17.95	2.12
4	−6	5	16.65	2.29
−6	6	−5	29.56	2.55
6	−6	5	20.81	2.60
−8	6	−5	38.61	3.22
8	−6	5	39.40	3.31
−10	6	−5	11.27	2.36
−12	6	−5	5.64	1.98
−14	6	−5	4.63	1.97
−15	−5	5	1.90	1.53
−13	−5	5	1.65	1.43
−11	−5	5	10.14	2.08
−9	−5	5	8.54	1.89
−7	−5	5	24.64	1.90

−7	−5	5	28.02	2.69
−5	−5	5	34.17	2.14
−5	−5	5	32.18	2.68
−3	−5	5	2.39	1.16
−3	−5	5	4.15	0.97
−1	−5	5	14.05	1.54
−1	−5	5	18.20	1.98
1	−5	5	20.18	1.77
1	−5	5	19.20	2.12
3	−5	5	35.34	2.62
3	−5	5	31.43	2.39
−5	5	−5	20.52	2.27
5	−5	5	24.42	2.49
−7	5	−5	11.03	2.02
7	−5	5	10.09	1.89
−9	5	−5	9.51	2.10
−11	5	−5	7.18	2.16
−13	5	−5	4.73	1.85
−15	5	−5	2.67	1.93
−16	−4	5	0.92	1.44
−14	−4	5	2.06	1.51
−12	−4	5	1.77	1.35
−10	−4	5	18.01	2.07
−10	−4	5	18.02	2.42
−8	−4	5	5.61	1.22
−8	−4	5	5.87	1.51
−6	−4	5	4.62	1.31
−6	−4	5	5.24	1.11
−4	−4	5	12.63	1.78
−4	−4	5	16.64	1.64
−2	−4	5	29.83	2.18
−2	−4	5	28.07	1.96
0	−4	5	11.54	1.61
0	−4	5	10.09	1.37
2	−4	5	12.90	1.68
2	−4	5	15.30	1.74
4	−4	5	2.34	0.98
4	−4	5	2.52	1.04
−6	4	−5	5.75	1.42
6	−4	5	7.51	1.54
−8	4	−5	0.40	0.97
−10	4	−5	1.56	1.18
−12	4	−5	0.24	1.35
−14	4	−5	−0.47	1.48

−16	4	−5	1.77	1.88
−17	−3	5	−0.34	1.38
−15	−3	5	−1.13	1.16
−13	−3	5	2.76	1.26
−13	−3	5	2.53	1.44
−11	−3	5	8.64	1.66
−11	−3	5	10.48	2.06
−9	−3	5	7.07	1.61
−9	−3	5	10.43	1.67
−7	−3	5	8.89	1.41
−7	−3	5	7.92	1.49
−5	−3	5	8.01	1.37
−5	−3	5	8.17	1.28
−3	−3	5	2.11	0.82
−3	−3	5	4.23	0.98
−1	−3	5	7.66	1.20
−1	−3	5	7.93	1.22
1	−3	5	1.04	0.68
1	−3	5	0.21	0.61
3	−3	5	2.62	0.93
3	−3	5	2.48	0.94
5	−3	5	3.63	1.23
5	−3	5	4.08	1.08
−7	3	−5	0.48	0.85
−9	3	−5	6.06	1.71
−11	3	−5	4.86	1.75
−13	3	−5	2.77	1.77
−15	3	−5	−0.41	1.35
−16	−2	5	2.37	1.62
−16	−2	5	1.52	1.58
−14	−2	5	3.28	1.40
−14	−2	5	2.80	1.55
−12	−2	5	19.20	2.50
−12	−2	5	21.91	2.59
−10	−2	5	12.06	1.90
−10	−2	5	15.29	2.07
−8	−2	5	7.38	1.46
−8	−2	5	7.95	1.49
−6	−2	5	46.59	2.61
−6	−2	5	49.76	2.81
−4	−2	5	89.48	3.89
−4	−2	5	90.52	3.69
−2	−2	5	30.44	1.85
−2	−2	5	28.20	1.98

0	−2	5	27.81	1.96
0	−2	5	32.07	1.93
2	−2	5	7.50	1.21
2	−2	5	7.84	1.25
4	−2	5	51.86	2.79
4	−2	5	47.83	2.79
6	−2	5	7.41	1.47
−8	2	−5	21.55	2.48
−10	2	−5	0.72	1.03
−12	2	−5	10.30	2.34
−14	2	−5	3.57	1.81
−16	2	−5	2.10	1.68
−17	−1	5	4.75	1.85
−15	−1	5	2.14	1.62
−15	−1	5	2.15	1.42
−13	−1	5	8.89	2.02
−13	−1	5	8.12	1.93
−11	−1	5	23.37	2.69
−11	−1	5	25.81	2.54
−9	−1	5	27.52	2.40
−9	−1	5	30.79	2.27
−7	−1	5	33.40	2.42
−7	−1	5	34.48	2.18
−1	−1	5	113.46	3.85
−1	−1	5	108.87	4.32
−1	1	−5	75.85	2.52
1	−1	5	68.86	2.94
1	−1	5	65.41	3.21
−3	1	−5	81.83	2.81
3	−1	5	87.15	3.48
3	−1	5	79.85	3.55
5	−1	5	41.48	2.58
−7	1	−5	60.78	3.59
−9	1	−5	46.98	3.24
−11	1	−5	6.85	1.82
−13	1	−5	11.50	2.56
−15	1	−5	4.90	2.03
−17	1	−5	3.17	1.95
−18	0	5	2.35	1.76
−16	0	5	6.06	2.06
−14	0	5	6.05	1.85
−12	0	5	39.18	2.97
−12	0	5	43.67	3.41
−10	0	5	3.83	1.36

−8	0	5	44.77	2.47
−8	0	5	41.37	2.91
−6	0	5	33.79	1.83
−6	0	5	29.74	2.28
−4	0	5	13.31	1.05
−4	0	5	16.68	1.74
2	0	−5	132.34	4.69
−2	0	5	137.03	5.07
−2	0	5	137.60	4.13
0	0	−5	45.85	2.16
0	0	5	43.08	1.94
0	0	5	43.63	2.41
−2	0	−5	247.81	7.17
2	0	5	242.97	7.77
4	0	5	69.75	3.28
6	0	5	35.75	2.41
−8	0	−5	47.21	3.09
−10	0	−5	24.20	2.68
−12	0	−5	21.25	2.86
−14	0	−5	4.34	1.82
−16	0	−5	6.04	2.30
−17	1	5	5.16	2.04
−15	1	5	2.73	1.53
−13	1	5	6.95	1.90
−11	1	5	21.93	2.75
−11	1	5	24.99	2.29
−9	1	5	32.10	2.15
−9	1	5	31.20	2.75
−7	1	5	45.88	2.18
−7	1	5	45.26	3.01
−5	1	5	89.18	4.10
−5	1	5	89.57	2.90
3	−1	−5	17.16	1.76
−3	1	5	24.86	1.11
−3	1	5	22.82	1.82
1	−1	−5	118.64	4.45
−1	1	5	121.81	3.48
−1	1	5	109.62	4.45
−1	−1	−5	69.33	2.95
1	1	5	72.19	3.22
−3	−1	−5	87.38	3.46
3	1	5	80.18	3.57
5	1	5	38.38	2.38
7	1	5	69.85	3.60

−9	−1	−5	46.98	3.15
−11	−1	−5	5.55	1.63
−13	−1	−5	13.90	2.58
−15	−1	−5	2.34	1.62
−17	−1	−5	−0.37	1.66
−16	2	5	2.27	1.67
−14	2	5	2.77	1.58
−12	2	5	20.17	2.88
−10	2	5	19.76	2.61
−8	2	5	9.31	1.14
−8	2	5	5.81	1.51
−6	2	5	47.93	3.06
−6	2	5	49.15	1.83
4	−2	−5	88.82	4.22
−4	2	5	90.27	4.07
2	−2	−5	26.05	2.08
−2	2	5	33.78	2.15
0	−2	−5	31.00	2.08
0	2	5	35.29	2.13
−2	−2	−5	9.17	1.29
2	2	5	9.43	1.27
4	2	5	55.41	2.77
6	2	5	10.66	1.47
−8	−2	−5	25.31	2.43
−10	−2	−5	−0.27	0.76
−12	−2	−5	11.41	2.16
−14	−2	−5	5.35	1.88
−16	−2	−5	1.70	1.76
−17	3	5	1.81	1.68
−15	3	5	0.16	1.25
−13	3	5	2.63	1.51
−11	3	5	5.64	1.74
−9	3	5	12.88	2.16
−7	3	5	8.29	1.68
5	−3	−5	8.62	1.77
−5	3	5	6.69	1.39
3	−3	−5	4.19	1.29
−3	3	5	6.15	1.24
1	−3	−5	7.76	1.42
−1	3	5	9.58	1.36
−1	−3	−5	1.60	0.91
1	3	5	2.95	0.82
−3	−3	−5	2.25	0.93
3	3	5	1.04	0.58

5	3	5	5.70	1.11
7	3	5	0.14	0.61
−9	−3	−5	6.68	1.54
−11	−3	−5	3.29	1.32
−13	−3	−5	6.68	1.86
−13	−3	−5	2.51	1.62
−15	−3	−5	1.55	1.60
−16	4	5	2.45	1.68
−14	4	5	1.98	1.50
−12	4	5	1.04	1.16
−10	4	5	13.05	2.33
−8	4	5	3.98	1.40
6	−4	−5	5.36	1.61
−6	4	5	2.70	1.10
4	−4	−5	14.44	2.09
−4	4	5	11.68	1.66
2	−4	−5	22.04	2.25
−2	4	5	18.26	1.86
0	−4	−5	10.61	1.64
0	−4	−5	11.29	0.89
0	4	5	9.50	1.35
−2	−4	−5	14.94	1.27
−2	−4	−5	13.75	1.83
2	4	5	11.91	1.41
−4	−4	−5	2.48	0.88
−4	−4	−5	2.20	1.11
4	4	5	3.72	0.87
−6	−4	−5	9.95	1.60
6	4	5	5.87	1.15
−8	−4	−5	3.19	1.28
−8	−4	−5	2.70	1.05
−10	−4	−5	0.51	0.91
−10	−4	−5	2.15	1.33
−12	−4	−5	1.32	1.38
−14	−4	−5	1.17	1.51
−16	−4	−5	1.49	1.78
−15	5	5	1.74	1.58
−13	5	5	2.93	1.55
−11	5	5	6.40	1.84
−9	5	5	6.27	1.74
7	−5	−5	22.33	2.88
−7	5	5	21.73	2.50
5	−5	−5	34.83	3.02
−5	5	5	32.56	2.59

3	−5	−5	3.80	1.38
−3	5	5	3.70	1.05
1	−5	−5	20.37	2.35
−1	5	5	13.89	1.61
−1	−5	−5	22.47	1.44
−1	−5	−5	23.15	2.46
1	5	5	19.83	1.66
−3	−5	−5	31.97	2.00
−3	−5	−5	31.70	2.64
3	5	5	30.88	1.96
−5	−5	−5	28.28	2.61
−5	−5	−5	25.78	2.13
−7	−5	−5	14.77	2.05
7	5	5	14.43	1.67
−9	−5	−5	7.96	1.79
−11	−5	−5	10.26	2.22
−13	−5	−5	2.80	1.75
−15	−5	−5	2.27	1.85
−14	6	5	1.25	1.51
−12	6	5	8.99	2.20
−10	6	5	3.06	1.47
8	−6	−5	10.77	2.35
−8	6	5	11.41	2.08
6	−6	−5	31.57	3.29
−6	6	5	25.57	2.65
4	−6	−5	23.38	2.85
−4	6	5	16.55	1.99
2	−6	−5	57.99	3.84
−2	6	5	55.90	3.14
0	−6	−5	51.30	3.47
0	6	5	55.58	2.91
−2	−6	−5	55.99	2.71
−2	−6	−5	66.26	4.04
−4	−6	−5	14.81	2.27
−4	−6	−5	15.51	1.76
4	6	5	19.79	1.57
−6	−6	−5	23.69	2.33
−8	−6	−5	35.80	2.96
−10	−6	−5	10.43	2.19
−12	−6	−5	5.46	1.98
−14	−6	−5	4.27	2.04
−15	7	5	3.74	1.93
−13	7	5	4.53	1.84
−11	7	5	7.69	2.16

9	−7	−5	8.69	2.24
−9	7	5	12.61	2.29
7	−7	−5	16.43	2.70
−7	7	5	15.35	2.34
5	−7	−5	12.88	2.48
−5	7	5	11.81	1.90
3	−7	−5	39.29	3.36
1	−7	−5	17.55	2.54
−1	7	5	12.46	1.56
−1	−7	−5	21.10	2.71
1	7	5	18.63	1.63
−3	−7	−5	60.70	4.15
−5	−7	−5	27.64	2.32
−5	−7	−5	26.02	3.07
−7	−7	−5	14.97	2.20
−9	−7	−5	11.07	2.13
−11	−7	−5	7.65	2.11
−13	−7	−5	2.19	1.81
−14	8	5	0.88	1.41
−12	8	5	2.75	1.52
10	−8	−5	3.24	1.74
−10	8	5	7.30	1.93
8	−8	−5	4.87	1.90
−8	8	5	7.30	1.87
6	−8	−5	5.99	2.00
−6	8	5	8.74	1.90
4	−8	−5	7.29	2.07
−4	8	5	8.85	1.63
2	−8	−5	36.47	3.39
−2	8	5	31.99	2.43
0	−8	−5	13.70	2.44
−2	−8	−5	3.27	1.58
−4	−8	−5	26.99	3.28
−6	−8	−5	1.85	1.54
−8	−8	−5	7.34	1.89
−10	−8	−5	2.32	1.68
−12	−8	−5	2.43	1.88
−11	9	5	0.15	1.07
9	−9	−5	0.23	1.34
−9	9	5	0.97	1.21
7	−9	−5	0.76	1.39
−7	9	5	2.89	1.33
5	−9	−5	5.45	2.02
−5	9	5	5.00	1.44

3	-9	-5	12.73	2.57
1	-9	-5	-0.97	1.03
-1	-9	-5	1.73	1.51
-3	-9	-5	0.24	1.37
-5	-9	-5	1.38	1.53
-7	-9	-5	0.67	1.49
-9	-9	-5	0.22	1.54
-11	-9	-5	1.47	1.81
10	-10	-5	3.05	1.75
-10	10	5	2.09	1.39
8	-10	-5	0.82	1.58
-8	10	5	0.99	1.25
6	-10	-5	3.53	1.73
4	-10	-5	0.17	1.29
2	-10	-5	-0.19	1.31
0	-10	-5	-0.53	1.16
-2	-10	-5	0.58	1.42
-4	-10	-5	0.36	1.54
-6	-10	-5	0.11	1.58
-8	-10	-5	-0.17	1.51
-10	-10	-5	0.84	1.93
9	-11	-5	5.44	2.30
7	-11	-5	4.79	2.06
5	-11	-5	0.73	1.59
3	-11	-5	4.32	1.81
1	-11	-5	5.68	2.15
-1	-11	-5	1.48	1.56
-3	-11	-5	3.16	1.91
-5	-11	-5	-0.79	1.31
-7	-11	-5	2.83	2.02
4	-12	-5	8.01	2.52
2	-12	-5	2.13	1.72
0	-12	-5	3.96	2.05
-2	-12	-5	5.12	2.16
-4	-12	-5	5.40	2.35
-2	-12	6	11.61	2.98
0	-12	6	1.65	1.91
-7	-11	6	3.50	2.05
-5	-11	6	6.18	2.19
-3	-11	6	0.26	1.20
-1	-11	6	6.65	2.36
1	-11	6	5.44	2.13
3	-11	6	1.66	1.74
5	-11	6	1.84	1.85

-10	-10	6	-0.21	1.62
-8	-10	6	4.06	1.96
-6	-10	6	1.37	1.45
-4	-10	6	-0.22	1.26
-2	-10	6	3.37	1.65
0	-10	6	-0.63	1.14
2	-10	6	1.85	1.57
4	-10	6	5.97	2.14
-6	10	-6	1.44	1.47
6	-10	6	0.29	1.54
-8	10	-6	3.30	1.62
8	-10	6	0.14	1.43
-11	-9	6	3.16	1.92
-9	-9	6	2.54	1.69
-7	-9	6	0.75	1.39
-5	-9	6	0.15	1.23
-3	-9	6	2.01	1.38
-1	-9	6	0.21	1.22
1	-9	6	0.74	1.16
-3	9	-6	3.87	1.50
3	-9	6	3.84	1.66
-5	9	-6	4.45	1.65
5	-9	6	1.43	1.52
-7	9	-6	3.04	1.66
7	-9	6	-0.09	1.36
-9	9	-6	-0.45	1.28
9	-9	6	0.06	1.47
-12	-8	6	2.04	1.71
-10	-8	6	0.92	1.34
-8	-8	6	4.73	1.87
-6	-8	6	11.00	2.37
-4	-8	6	6.45	1.79
-2	-8	6	8.14	1.91
0	8	-6	2.34	1.24
0	-8	6	2.14	1.30
-2	8	-6	4.58	1.43
2	-8	6	4.43	1.61
-4	8	-6	18.38	2.53
4	-8	6	15.79	2.66
-6	8	-6	0.55	1.06
6	-8	6	1.84	1.41
-8	8	-6	0.44	1.22
8	-8	6	0.92	1.43
-10	8	-6	2.69	1.69

−12	8	−6	1.04	1.60
−13	−7	6	1.93	1.64
−11	−7	6	6.26	2.00
−9	−7	6	11.99	2.45
−7	−7	6	9.32	2.20
−5	−7	6	16.20	2.51
−3	−7	6	44.45	3.44
−3	−7	6	46.05	2.75
−1	−7	6	36.03	2.56
−1	−7	6	39.38	3.20
1	−7	6	14.11	2.01
1	−7	6	10.44	2.07
−3	7	−6	24.69	2.57
3	−7	6	25.01	2.90
−5	7	−6	12.31	2.18
5	−7	6	12.99	2.38
−7	7	−6	6.53	1.86
7	−7	6	7.22	1.90
−9	7	−6	12.97	2.61
−11	7	−6	3.38	1.65
−13	7	−6	5.53	2.06
−14	−6	6	3.62	1.84
−12	−6	6	5.49	1.91
−10	−6	6	10.10	2.27
−8	−6	6	23.60	2.89
−6	−6	6	24.77	2.88
−6	−6	6	26.83	2.03
−4	−6	6	8.70	1.41
−4	−6	6	6.18	1.57
−2	−6	6	48.23	2.80
−2	−6	6	44.97	3.17
0	−6	6	61.94	3.40
0	−6	6	61.04	3.78
2	−6	6	53.11	3.52
2	−6	6	48.50	3.20
−4	6	−6	20.26	2.38
4	−6	6	22.94	2.72
−6	6	−6	15.14	2.28
6	−6	6	18.39	2.56
−8	6	−6	10.39	2.19
8	−6	6	11.28	2.25
−10	6	−6	4.42	1.73
−12	6	−6	5.21	1.89
−14	6	−6	0.60	1.46

−15	−5	6	3.59	1.78
−13	−5	6	3.32	1.72
−11	−5	6	19.48	2.81
−9	−5	6	7.62	1.83
−9	−5	6	8.12	1.53
−7	−5	6	15.73	2.28
−7	−5	6	17.34	1.96
−5	−5	6	28.92	2.16
−5	−5	6	23.18	2.54
−3	−5	6	16.32	2.05
−3	−5	6	16.26	1.82
−1	−5	6	39.37	2.52
−1	−5	6	38.00	2.79
1	−5	6	5.19	1.34
1	−5	6	5.99	1.37
3	−5	6	5.46	1.49
3	−5	6	8.11	1.66
−5	5	−6	29.98	2.89
5	−5	6	34.25	2.80
−7	5	−6	7.96	1.85
7	−5	6	7.12	1.74
−9	5	−6	3.95	1.48
−11	5	−6	−0.42	1.13
−13	5	−6	4.19	2.05
−16	−4	6	0.10	1.63
−14	−4	6	3.26	1.68
−12	−4	6	5.23	1.77
−10	−4	6	8.77	1.92
−10	−4	6	6.52	1.55
−8	−4	6	1.36	0.94
−8	−4	6	2.53	1.24
−6	−4	6	7.91	1.66
−6	−4	6	7.56	1.42
−4	−4	6	22.92	1.96
−4	−4	6	24.88	2.17
−2	−4	6	1.93	0.94
−2	−4	6	1.28	0.77
0	−4	6	16.60	1.90
0	−4	6	11.32	1.56
2	−4	6	15.32	1.92
2	−4	6	15.68	1.97
4	−4	6	7.24	1.47
4	−4	6	6.39	1.49
−6	4	−6	4.18	1.43

6	−4	6	0.09	0.85
−8	4	−6	6.68	1.70
−10	4	−6	5.50	1.75
−12	4	−6	2.02	1.61
−14	4	−6	1.70	1.77
−15	−3	6	3.34	1.81
−13	−3	6	1.48	1.43
−13	−3	6	2.26	1.23
−11	−3	6	11.30	2.06
−11	−3	6	10.24	1.95
−9	−3	6	2.13	1.07
−9	−3	6	2.71	1.25
−7	−3	6	9.33	1.61
−7	−3	6	6.24	1.39
−5	−3	6	11.83	1.63
−5	−3	6	10.82	1.61
−3	−3	6	3.48	0.99
−3	−3	6	3.23	1.00
−1	−3	6	6.80	1.31
−1	−3	6	7.37	1.31
1	−3	6	0.41	0.64
1	−3	6	0.31	0.65
3	−3	6	4.78	1.18
3	−3	6	5.42	1.27
5	−3	6	11.78	1.78
5	−3	6	9.14	1.68
−7	3	−6	5.25	1.47
−9	3	−6	2.69	1.37
−11	3	−6	6.99	2.00
−13	3	−6	4.08	1.84
−15	3	−6	1.70	1.85
−16	−2	6	0.89	1.47
−14	−2	6	8.87	2.15
−14	−2	6	7.67	1.96
−12	−2	6	8.83	1.95
−12	−2	6	6.85	1.70
−10	−2	6	29.77	2.55
−10	−2	6	30.97	2.71
−8	−2	6	14.98	1.83
−8	−2	6	12.37	1.86
−6	−2	6	34.19	2.54
−6	−2	6	38.87	2.42
−4	−2	6	22.46	1.72
−4	−2	6	22.69	1.93

−2	−2	6	23.04	1.96
−2	−2	6	21.19	1.66
0	−2	6	37.58	2.49
0	−2	6	35.11	2.19
2	−2	6	9.25	1.36
2	−2	6	7.23	1.31
4	−2	6	34.75	2.53
4	−2	6	35.12	2.37
6	−2	6	20.80	2.35
−8	2	−6	7.47	1.72
−10	2	−6	5.45	1.73
−12	2	−6	9.00	2.27
−14	2	−6	4.82	2.10
−16	2	−6	0.58	1.62
−17	−1	6	7.12	2.25
−15	−1	6	4.27	1.76
−13	−1	6	9.60	2.12
−11	−1	6	17.13	2.19
−11	−1	6	17.12	2.45
−9	−1	6	13.43	2.04
−7	−1	6	94.17	3.86
−7	−1	6	95.15	4.66
−5	−1	6	60.22	2.79
−5	−1	6	58.48	3.32
−3	−1	6	31.75	1.83
−3	−1	6	31.25	2.29
−1	1	−6	56.29	2.26
1	−1	6	53.20	3.06
1	−1	6	59.85	2.73
−3	1	−6	51.78	2.07
3	−1	6	48.68	2.65
3	−1	6	47.96	2.85
−5	1	−6	22.34	1.46
5	−1	6	17.53	2.01
7	−1	6	30.70	2.58
−9	1	−6	28.78	2.93
−11	1	−6	15.88	2.61
−13	1	−6	2.66	1.65
−15	1	−6	2.36	1.79
−16	0	6	3.01	1.80
−14	0	6	5.72	1.92
−12	0	6	17.55	2.72
−10	0	6	26.30	2.88
−8	0	6	4.99	1.08

−8	0	6	5.20	1.42
−6	0	6	36.72	2.00
−6	0	6	39.21	2.83
−4	0	6	29.60	2.28
−4	0	6	23.21	1.41
2	0	−6	146.27	5.23
−2	0	6	153.70	5.76
−2	0	6	148.19	4.61
0	0	−6	102.45	3.83
0	0	6	102.15	3.61
0	0	6	98.89	4.33
−2	0	−6	84.84	3.23
2	0	6	79.22	3.85
−4	0	−6	26.53	1.72
4	0	6	25.49	2.03
6	0	6	12.03	1.74
−8	0	−6	20.19	2.49
−10	0	−6	1.86	1.25
−12	0	−6	1.51	1.33
−14	0	−6	1.75	1.69
−16	0	−6	0.26	1.56
−17	1	6	4.46	2.09
−15	1	6	5.14	1.96
−13	1	6	9.00	2.20
−11	1	6	18.93	2.74
−9	1	6	19.60	2.54
−7	1	6	97.90	4.78
−5	1	6	62.00	2.25
−5	1	6	58.64	3.50
3	−1	−6	36.55	2.44
−3	1	6	35.61	1.54
−3	1	6	36.90	2.54
1	−1	−6	37.07	2.38
−1	1	6	39.39	2.56
−1	1	6	41.90	1.77
−1	−1	−6	58.90	2.89
1	1	6	57.88	3.11
−3	−1	−6	58.39	2.88
3	1	6	57.44	3.13
−5	−1	−6	17.68	1.64
5	1	6	21.11	2.10
7	1	6	34.33	2.59
−9	−1	−6	27.67	2.76
−11	−1	−6	12.24	2.23

−13	−1	−6	1.20	1.40
−15	−1	−6	4.28	1.91
−16	2	6	3.85	1.86
−14	2	6	5.43	1.94
−12	2	6	12.01	2.49
−10	2	6	28.99	3.23
−8	2	6	8.81	1.86
−6	2	6	37.39	2.78
4	−2	−6	25.30	2.28
−4	2	6	25.47	2.17
2	−2	−6	22.49	2.05
−2	2	6	21.36	2.08
0	−2	−6	38.14	2.48
0	2	6	37.08	2.44
−2	−2	−6	9.02	1.43
2	2	6	9.10	1.41
−4	−2	−6	34.41	2.35
4	2	6	37.01	2.41
6	2	6	23.41	2.05
8	2	6	4.72	1.30
−10	−2	−6	4.67	1.48
−12	−2	−6	6.30	1.79
−14	−2	−6	4.93	1.97
−15	3	6	4.10	1.89
−13	3	6	1.34	1.43
−11	3	6	10.52	2.32
−9	3	6	3.76	1.37
−7	3	6	6.89	1.63
5	−3	−6	13.22	2.15
−5	3	6	10.73	1.79
3	−3	−6	3.95	1.33
−3	3	6	1.78	0.84
1	−3	−6	8.49	1.57
−1	3	6	8.15	1.42
−1	−3	−6	0.00	0.74
1	3	6	0.52	0.66
−3	−3	−6	8.70	1.58
3	3	6	5.56	1.22
−5	−3	−6	14.74	1.92
5	3	6	8.62	1.45
7	3	6	6.69	1.41
−9	−3	−6	3.04	1.16
−11	−3	−6	5.20	1.61
−15	−3	−6	−0.75	1.47

−16	4	6	1.79	1.66
−14	4	6	3.67	1.92
−12	4	6	3.52	1.69
−10	4	6	5.97	1.74
−8	4	6	0.66	0.99
6	−4	−6	8.37	1.99
−6	4	6	6.67	1.59
4	−4	−6	24.61	2.70
−4	4	6	21.51	2.30
2	−4	−6	1.77	1.18
−2	4	6	3.59	1.04
0	−4	−6	16.54	2.05
0	4	6	12.63	1.66
−2	−4	−6	15.50	1.29
−2	−4	−6	15.54	2.07
2	4	6	12.00	1.59
−4	−4	−6	8.16	1.34
−4	−4	−6	7.10	1.64
4	4	6	4.00	1.07
−6	−4	−6	1.24	0.98
6	4	6	2.54	0.92
−8	−4	−6	3.87	1.47
8	4	6	7.28	1.48
−10	−4	−6	10.00	1.87
−10	−4	−6	6.12	1.84
−12	−4	−6	2.79	1.60
−14	−4	−6	1.40	1.62
−15	5	6	3.83	2.04
−13	5	6	2.57	1.52
−11	5	6	12.71	2.60
−9	5	6	10.08	2.17
7	−5	−6	18.84	2.85
−7	5	6	13.20	2.16
5	−5	−6	26.21	3.01
−5	5	6	26.17	2.34
3	−5	−6	20.28	2.65
−3	5	6	13.58	1.86
1	−5	−6	39.50	3.15
−1	5	6	43.70	2.75
−1	−5	−6	6.06	1.65
1	5	6	7.40	1.38
−3	−5	−6	5.35	1.62
−3	−5	−6	6.56	1.16
3	5	6	8.84	1.39

−5	−5	−6	27.74	2.22
−5	−5	−6	32.87	2.88
5	5	6	33.39	2.20
−7	−5	−6	10.17	1.84
7	5	6	8.50	1.43
−9	−5	−6	7.90	1.90
−11	−5	−6	2.19	1.54
−13	−5	−6	3.15	1.81
−14	6	6	4.80	1.90
−12	6	6	7.06	2.14
−10	6	6	7.89	2.14
8	−6	−6	29.38	3.65
−8	6	6	26.27	3.03
6	−6	−6	25.49	3.19
−6	6	6	22.60	2.59
4	−6	−6	10.36	2.28
−4	6	6	6.77	1.51
2	−6	−6	49.79	3.69
−2	6	6	48.46	3.00
0	−6	−6	58.96	3.89
0	6	6	59.20	3.24
−2	−6	−6	50.96	3.71
2	6	6	52.16	2.93
−4	−6	−6	27.01	3.01
−6	−6	−6	18.38	2.18
−6	−6	−6	21.00	2.81
−8	−6	−6	11.37	2.02
−10	−6	−6	3.18	1.56
−12	−6	−6	2.07	1.82
−14	−6	−6	0.05	1.60
−13	7	6	3.04	1.72
−11	7	6	9.30	2.35
9	−7	−6	9.08	2.40
−9	7	6	13.80	2.51
7	−7	−6	11.60	2.67
−7	7	6	9.59	2.01
5	−7	−6	19.03	2.94
−5	7	6	13.98	2.06
3	−7	−6	42.04	3.63
−3	7	6	47.43	3.12
1	−7	−6	36.87	3.41
−1	7	6	36.63	2.62
−1	−7	−6	11.16	2.28
1	7	6	12.84	1.71

-3	-7	-6	23.41	3.08
-5	-7	-6	15.41	2.63
-7	-7	-6	9.76	2.33
-9	-7	-6	9.26	2.05
-11	-7	-6	0.31	1.49
-13	-7	-6	0.74	1.81
-12	8	6	4.04	1.91
10	-8	-6	0.99	1.35
-10	8	6	2.97	1.54
8	-8	-6	5.27	2.02
-8	8	6	5.01	1.62
6	-8	-6	5.50	2.11
-6	8	6	7.85	1.79
4	-8	-6	9.58	2.39
-4	8	6	5.98	1.53
2	-8	-6	8.36	2.18
-2	8	6	8.64	1.62
0	-8	-6	1.59	1.46
-2	-8	-6	4.07	1.82
-4	-8	-6	15.00	2.80
-6	-8	-6	-0.52	1.27
-8	-8	-6	3.52	1.74
-11	9	6	1.25	1.35
9	-9	-6	5.97	2.20
-9	9	6	4.53	1.75
7	-9	-6	2.76	1.72
-7	9	6	1.59	1.19
5	-9	-6	-0.33	1.25
-5	9	6	0.39	0.89
3	-9	-6	1.37	1.60
1	-9	-6	1.58	1.58
-1	-9	-6	0.40	1.37
-3	-9	-6	5.24	2.08
-5	-9	-6	1.39	1.61
-7	-9	-6	1.43	1.71
-9	-9	-6	0.54	1.65
10	-10	-6	2.49	1.98
-10	10	6	0.58	1.37
8	-10	-6	3.55	2.01
-8	10	6	-0.04	1.16
6	-10	-6	0.12	1.36
4	-10	-6	0.07	1.40
2	-10	-6	2.32	1.67
0	-10	-6	0.68	1.64

-2	-10	-6	0.79	1.67
-4	-10	-6	3.43	2.08
-6	-10	-6	0.93	1.83
-8	-10	-6	-0.41	1.68
7	-11	-6	8.04	2.62
5	-11	-6	2.74	1.87
3	-11	-6	0.50	1.53
1	-11	-6	5.38	2.19
-1	-11	-6	4.17	2.17
-3	-11	-6	1.51	1.88
-5	-11	-6	0.45	1.71
2	-12	-6	8.39	2.58
0	-12	-6	2.34	2.04
-5	-11	7	0.38	1.61
-3	-11	7	1.51	1.60
-1	-11	7	2.47	1.64
1	-11	7	0.07	1.41
3	-11	7	0.61	1.67
-8	-10	7	1.96	1.78
-6	-10	7	1.73	1.44
-4	-10	7	1.88	1.49
-2	-10	7	3.65	1.74
0	-10	7	2.76	1.65
2	-10	7	0.84	1.38
4	-10	7	5.83	1.97
6	-10	7	-0.46	1.39
-9	-9	7	2.31	1.74
-7	-9	7	1.84	1.57
-5	-9	7	4.10	1.83
-3	-9	7	2.98	1.58
-1	-9	7	3.43	1.61
1	-9	7	5.65	1.83
-3	9	-7	13.46	2.41
3	-9	7	11.91	2.51
-5	9	-7	4.13	1.70
5	-9	7	3.37	1.76
-7	9	-7	2.04	1.53
7	-9	7	1.21	1.50
-9	9	-7	1.49	1.56
9	-9	7	0.00	1.51
-12	-8	7	3.78	1.83
-10	-8	7	-0.83	1.25
-8	-8	7	3.10	1.77
-6	-8	7	6.97	2.07

−4	−8	7	3.79	1.72
−2	−8	7	3.92	1.61
0	−8	7	2.86	1.48
0	−8	7	4.80	1.47
−2	8	−7	1.68	1.21
2	−8	7	2.83	1.45
−4	8	−7	4.82	1.68
4	−8	7	4.36	1.67
−6	8	−7	2.90	1.52
6	−8	7	3.30	1.54
−8	8	−7	1.48	1.28
8	−8	7	2.42	1.46
−10	8	−7	−0.04	1.32
−13	−7	7	0.35	1.41
−11	−7	7	1.68	1.58
−9	−7	7	1.40	1.51
−7	−7	7	3.97	1.64
−5	−7	7	5.69	1.78
−3	−7	7	2.89	1.44
−3	−7	7	2.91	1.11
−1	−7	7	7.14	1.60
−1	−7	7	7.03	1.80
1	−7	7	2.09	1.21
1	−7	7	3.86	1.50
−3	7	−7	0.60	1.15
3	−7	7	1.52	1.20
−5	7	−7	0.27	1.18
5	−7	7	−0.68	0.82
−7	7	−7	2.13	1.41
7	−7	7	−0.09	1.06
−9	7	−7	0.70	1.17
−11	7	−7	0.45	1.40
−14	−6	7	2.49	1.64
−12	−6	7	1.10	1.44
−10	−6	7	8.89	2.21
−8	−6	7	0.95	1.33
−6	−6	7	1.67	1.26
−6	−6	7	1.19	0.96
−4	−6	7	3.24	1.11
−4	−6	7	1.72	1.21
−2	−6	7	14.87	1.93
−2	−6	7	19.45	2.49
0	−6	7	0.19	0.96
0	−6	7	0.89	0.99

2	−6	7	0.45	1.01
2	−6	7	0.72	1.00
−4	6	−7	12.48	2.22
4	−6	7	10.62	2.13
−6	6	−7	0.64	1.12
6	−6	7	2.42	1.32
−8	6	−7	1.34	1.33
8	−6	7	2.13	1.39
−10	6	−7	0.48	1.14
−12	6	−7	3.60	1.90
−15	−5	7	1.53	1.71
−13	−5	7	3.29	1.76
−11	−5	7	5.20	1.86
−9	−5	7	9.20	1.78
−9	−5	7	9.52	2.04
−7	−5	7	5.43	1.38
−7	−5	7	6.90	1.79
−5	−5	7	6.25	1.39
−5	−5	7	5.79	1.55
−3	−5	7	14.57	2.00
−3	−5	7	15.87	2.01
−1	−5	7	29.13	2.39
−1	−5	7	29.35	2.49
1	−5	7	21.20	2.34
1	−5	7	16.62	2.20
3	−5	7	2.29	1.20
3	−5	7	2.74	1.24
−5	5	−7	22.41	2.76
5	−5	7	16.20	2.35
−7	5	−7	2.96	1.42
7	−5	7	2.03	1.21
−9	5	−7	4.33	1.68
−11	5	−7	3.29	1.54
−13	5	−7	0.54	1.28
−12	−4	7	4.82	1.74
−10	−4	7	9.60	1.89
−10	−4	7	9.52	2.01
−8	−4	7	1.34	0.95
−8	−4	7	0.89	1.12
−6	−4	7	33.66	2.63
−6	−4	7	36.30	2.80
−4	−4	7	21.87	2.27
−4	−4	7	20.62	2.22
−2	−4	7	21.35	2.22

−2	−4	7	22.44	2.29
0	−4	7	25.14	2.21
0	−4	7	26.19	2.24
2	−4	7	5.61	1.40
2	−4	7	6.96	1.55
4	−4	7	23.07	2.56
4	−4	7	20.83	2.37
−6	4	−7	15.79	2.50
6	−4	7	12.55	2.08
−8	4	−7	10.56	2.13
−10	4	−7	7.03	1.97
−12	4	−7	4.87	2.04
−14	4	−7	6.44	2.46
−13	−3	7	5.60	1.65
−11	−3	7	6.24	1.64
−11	−3	7	8.53	1.92
−9	−3	7	0.91	1.00
−9	−3	7	1.41	1.17
−7	−3	7	12.00	1.87
−7	−3	7	11.06	1.86
−5	−3	7	14.93	1.97
−5	−3	7	11.95	1.69
−3	−3	7	0.70	0.73
−3	−3	7	1.14	0.81
−1	−3	7	13.27	1.90
−1	−3	7	15.41	1.74
1	−3	7	18.54	1.99
1	−3	7	16.05	1.99
3	−3	7	27.61	2.45
3	−3	7	26.70	2.23
5	−3	7	17.94	2.17
5	−3	7	15.60	2.24
−7	3	−7	18.85	2.64
−9	3	−7	12.83	2.49
−11	3	−7	10.19	2.40
−13	3	−7	3.40	1.83
−16	−2	7	1.23	1.52
−14	−2	7	1.37	1.45
−12	−2	7	0.75	1.17
−10	−2	7	4.32	1.41
−10	−2	7	2.22	1.18
−8	−2	7	5.37	1.49
−8	−2	7	2.91	1.12
−6	−2	7	8.89	1.42

−6	−2	7	9.52	1.75
−4	−2	7	17.34	1.70
−4	−2	7	20.11	2.23
−2	−2	7	23.23	1.78
−2	−2	7	25.41	2.18
0	−2	7	17.73	2.09
0	−2	7	20.28	1.86
2	−2	7	15.33	1.94
2	−2	7	21.05	1.81
4	−2	7	13.91	1.81
4	−2	7	12.58	2.00
6	−2	7	4.64	1.45
−8	2	−7	7.10	1.82
−10	2	−7	18.73	2.81
−12	2	−7	2.63	1.68
−14	2	−7	5.34	2.21
−15	−1	7	1.49	1.50
−13	−1	7	4.38	1.64
−11	−1	7	6.82	1.84
−9	−1	7	3.94	1.44
−7	−1	7	5.83	1.19
−7	−1	7	4.94	1.41
−5	−1	7	21.94	2.38
−5	−1	7	20.96	1.65
−3	−1	7	14.35	1.92
−3	−1	7	17.06	1.39
−1	−1	7	20.76	2.19
1	−1	7	14.39	1.52
1	−1	7	14.53	1.91
3	−1	7	6.76	1.46
5	−1	7	5.40	1.42
7	−1	7	15.70	2.33
−9	1	−7	3.44	1.41
−11	1	−7	1.35	1.30
−13	1	−7	4.56	1.95
−15	1	−7	−0.71	1.42
−16	0	7	2.62	1.66
−14	0	7	1.04	1.29
−12	0	7	6.42	1.87
−10	0	7	5.99	1.70
−8	0	7	19.29	2.54
−6	0	7	15.43	2.12
−4	0	7	5.83	0.89
−4	0	7	5.19	1.35

2	0	−7	0.13	0.67
−2	0	7	0.02	0.65
−2	0	7	0.40	0.51
0	0	−7	22.40	1.72
0	0	7	18.68	2.10
0	0	7	24.70	1.60
−2	0	−7	9.61	1.22
2	0	7	5.77	1.38
4	0	7	0.83	0.80
6	0	7	19.64	2.28
8	0	7	10.55	2.01
−10	0	−7	0.39	1.06
−12	0	−7	0.93	1.25
−14	0	−7	0.08	1.48
−15	1	7	1.56	1.58
−13	1	7	2.42	1.52
−11	1	7	5.83	1.77
−9	1	7	2.70	1.24
−7	1	7	4.53	1.45
−5	1	7	16.42	2.12
3	−1	−7	13.60	1.79
−3	1	7	15.96	2.04
1	−1	−7	23.15	1.96
−1	1	7	20.33	2.19
−1	−1	−7	15.10	1.63
1	1	7	10.16	1.68
−3	−1	−7	13.30	1.57
3	1	7	9.86	1.66
−5	−1	−7	4.59	1.17
5	1	7	1.69	0.88
7	1	7	13.07	1.99
−9	−1	−7	5.03	1.64
−11	−1	−7	1.58	1.20
−13	−1	−7	8.45	2.13
−15	−1	−7	0.22	1.61
−16	2	7	4.14	2.10
−14	2	7	1.25	1.38
−12	2	7	1.85	1.36
−10	2	7	5.15	1.73
−8	2	7	3.06	1.33
−6	2	7	5.02	1.45
4	−2	−7	17.71	2.21
−4	2	7	14.08	2.02
2	−2	−7	23.80	2.18

-2	2	7	20.15	2.22
0	-2	-7	17.16	1.92
0	2	7	20.26	2.19
-2	-2	-7	20.61	1.99
2	2	7	17.47	2.02
-4	-2	-7	15.88	1.91
4	2	7	16.11	1.96
-6	-2	-7	4.66	1.27
6	2	7	3.26	1.19
8	2	7	6.53	1.61
-10	-2	-7	15.06	2.36
-12	-2	-7	2.04	1.38
-14	-2	-7	6.32	2.28
-15	3	7	2.40	1.85
-13	3	7	2.92	1.62
-11	3	7	11.90	2.50
-9	3	7	1.05	1.08
-7	3	7	9.32	1.94
5	-3	-7	11.26	2.07
-5	3	7	14.75	2.10
3	-3	-7	1.53	1.18
-3	3	7	0.06	0.75
1	-3	-7	15.63	2.09
-1	3	7	13.10	1.89
-1	-3	-7	18.75	2.15
1	3	7	14.59	1.90
-3	-3	-7	29.53	2.48
3	3	7	28.22	2.34
-5	-3	-7	18.14	2.20
5	3	7	23.78	2.11
-7	-3	-7	16.46	2.22
7	3	7	18.94	2.20
-9	-3	-7	14.07	2.29
-11	-3	-7	8.84	2.00
-14	4	7	0.59	1.60
-12	4	7	4.09	1.87
-10	4	7	8.48	2.13
-8	4	7	1.93	1.24
6	-4	-7	36.54	3.24
-6	4	7	27.07	2.88
4	-4	-7	18.52	2.55
-4	4	7	18.89	2.24
2	-4	-7	22.11	2.66
-2	4	7	26.45	2.34

0	−4	−7	24.48	2.66
0	4	7	26.29	2.27
−2	−4	−7	5.24	1.54
2	4	7	6.40	1.38
−4	−4	−7	20.87	2.51
4	4	7	21.59	2.19
−6	−4	−7	15.34	2.25
−6	−4	−7	13.40	1.94
6	4	7	16.06	2.01
−8	−4	−7	10.71	2.05
8	4	7	13.53	2.04
−10	−4	−7	8.48	1.86
−10	−4	−7	5.22	1.82
−12	−4	−7	5.86	1.95
−14	−4	−7	2.70	1.90
−15	5	7	2.95	1.91
−13	5	7	3.97	1.78
−11	5	7	3.30	1.51
−9	5	7	9.98	2.15
7	−5	−7	4.68	1.74
−7	5	7	1.73	1.13
5	−5	−7	5.73	1.86
−5	5	7	4.94	1.53
3	−5	−7	16.84	2.63
−3	5	7	20.32	2.32
1	−5	−7	29.07	3.06
−1	5	7	29.33	2.45
−1	−5	−7	19.80	2.48
1	5	7	20.44	2.22
−3	−5	−7	2.41	1.38
3	5	7	5.46	1.33
−5	−5	−7	13.40	2.28
5	5	7	15.55	1.92
−7	−5	−7	2.53	1.42
−7	−5	−7	3.16	1.33
7	5	7	1.34	1.03
−9	−5	−7	6.93	1.84
−11	−5	−7	1.92	1.62
−13	−5	−7	1.14	1.61
−14	6	7	1.22	1.47
−12	6	7	0.14	1.20
−10	6	7	8.98	2.23
8	−6	−7	2.63	1.68
−8	6	7	4.03	1.57

6	−6	−7	0.71	1.28
−6	6	7	1.05	1.10
4	−6	−7	2.86	1.61
−4	6	7	2.59	1.18
2	−6	−7	16.54	2.63
−2	6	7	21.20	2.35
0	−6	−7	0.70	1.25
0	6	7	0.46	0.87
−2	−6	−7	1.65	1.36
2	6	7	0.15	0.85
−4	−6	−7	12.23	2.44
4	6	7	13.91	1.87
−6	−6	−7	2.69	1.62
−8	−6	−7	1.36	1.40
−12	−6	−7	0.02	1.76
−13	7	7	0.39	1.41
−11	7	7	0.80	1.21
9	−7	−7	2.93	1.93
−9	7	7	2.44	1.42
7	−7	−7	2.40	1.75
−7	7	7	5.28	1.61
5	−7	−7	3.71	1.82
−5	7	7	7.82	1.83
3	−7	−7	1.45	1.37
−3	7	7	4.11	1.30
1	−7	−7	9.76	2.37
−1	7	7	7.73	1.64
−1	−7	−7	2.31	1.52
1	7	7	2.10	1.07
−3	−7	−7	1.45	1.39
−5	−7	−7	0.79	1.40
−7	−7	−7	−0.38	1.27
−9	−7	−7	1.73	1.60
−11	−7	−7	1.50	1.61
−12	8	7	2.64	1.60
10	−8	−7	0.84	1.63
−10	8	7	2.20	1.43
8	−8	−7	2.62	1.88
−8	8	7	3.00	1.40
6	−8	−7	8.26	2.41
−6	8	7	5.41	1.68
4	−8	−7	3.56	2.02
−4	8	7	3.09	1.25
2	−8	−7	4.06	1.87

−2	8	7	3.04	1.26
0	−8	−7	2.76	1.80
−2	−8	−7	2.31	1.68
−4	−8	−7	2.35	1.71
−6	−8	−7	3.56	1.82
−8	−8	−7	1.24	1.67
−10	−8	−7	0.81	1.76
9	−9	−7	3.62	2.12
−9	9	7	2.78	1.55
7	−9	−7	3.19	2.11
−7	9	7	0.42	1.04
5	−9	−7	1.77	1.77
−5	9	7	2.33	1.29
3	−9	−7	4.71	2.14
1	−9	−7	0.90	1.76
−1	−9	−7	5.36	2.15
−3	−9	−7	11.78	2.82
−5	−9	−7	3.87	1.99
−7	−9	−7	0.31	1.71
−9	−9	−7	1.82	1.76
8	−10	−7	4.27	2.32
−8	10	7	1.55	1.45
6	−10	−7	0.47	1.68
4	−10	−7	0.74	1.83
2	−10	−7	5.86	2.40
0	−10	−7	3.75	2.18
−2	−10	−7	1.02	1.55
−4	−10	−7	6.50	2.52
−6	−10	−7	−2.42	1.49
5	−11	−7	2.64	1.89
3	−11	−7	1.21	1.84
1	−11	−7	1.09	1.78
−1	−11	−7	2.38	2.09
−3	−11	−7	−1.03	1.62
−4	−10	8	6.18	2.16
−2	−10	8	4.33	1.88
0	−10	8	4.47	1.79
2	−10	8	8.00	2.37
4	−10	8	8.99	2.44
−7	−9	8	8.28	2.34
−5	−9	8	8.16	2.17
−3	−9	8	6.38	2.01
−1	−9	8	6.16	2.04
1	−9	8	7.52	2.13

−3	9	−8	8.59	2.19
3	−9	8	7.53	2.13
−5	9	−8	4.62	1.83
5	−9	8	6.94	2.08
−7	9	−8	4.17	1.83
7	−9	8	4.72	1.81
−10	−8	8	2.75	1.76
−8	−8	8	5.31	1.99
−6	−8	8	4.33	1.77
−4	−8	8	2.97	1.51
−2	−8	8	9.15	2.17
0	−8	8	8.43	2.12
0	−8	8	12.74	2.36
−2	8	−8	4.38	1.66
2	−8	8	5.92	1.79
−4	8	−8	0.14	1.20
4	−8	8	0.93	1.21
−6	8	−8	2.87	1.63
6	−8	8	0.55	1.12
−8	8	−8	−0.14	1.12
8	−8	8	1.13	1.40
−11	−7	8	1.56	1.83
−9	−7	8	2.86	1.53
−7	−7	8	−0.92	1.11
−5	−7	8	1.23	1.25
−3	−7	8	1.41	1.12
−3	−7	8	0.74	1.22
−1	−7	8	1.09	1.10
−1	−7	8	1.64	1.27
1	−7	8	1.68	1.23
1	−7	8	1.32	1.26
−3	7	−8	−0.38	1.08
3	−7	8	−0.40	0.95
−5	7	−8	0.99	1.17
5	−7	8	3.01	1.49
−7	7	−8	1.24	1.38
7	−7	8	1.31	1.31
−9	7	−8	0.35	1.26
−12	−6	8	1.85	1.63
−10	−6	8	3.17	1.78
−8	−6	8	4.46	1.71
−6	−6	8	−0.12	1.11
−6	−6	8	1.52	1.10
−4	−6	8	2.34	1.12

−4	−6	8	3.36	1.45
−2	−6	8	0.88	1.05
−2	−6	8	3.40	1.37
0	−6	8	−0.13	0.91
0	−6	8	0.79	0.97
2	−6	8	2.38	1.31
2	−6	8	0.27	1.00
4	−6	8	0.11	1.15
4	−6	8	1.04	1.09
−6	6	−8	2.80	1.42
6	−6	8	−0.08	1.04
−8	6	−8	2.23	1.49
−10	6	−8	−0.42	1.16
−11	−5	8	5.88	1.97
−9	−5	8	10.97	2.32
−7	−5	8	5.94	1.72
−7	−5	8	5.92	1.62
−5	−5	8	11.61	1.86
−5	−5	8	18.97	2.41
−3	−5	8	15.43	2.19
−3	−5	8	14.57	2.12
−1	−5	8	6.21	1.52
−1	−5	8	6.90	1.66
1	−5	8	7.03	1.71
1	−5	8	4.98	1.49
3	−5	8	9.38	1.99
3	−5	8	6.05	1.65
5	−5	8	13.36	2.28
5	−5	8	11.05	2.28
−7	5	−8	4.50	1.65
−9	5	−8	5.01	1.79
−11	5	−8	0.79	1.35
−10	−4	8	15.31	2.45
−10	−4	8	11.67	2.11
−8	−4	8	8.04	1.85
−8	−4	8	5.75	1.55
−6	−4	8	20.25	2.32
−6	−4	8	17.03	2.33
−4	−4	8	18.03	2.27
−4	−4	8	18.18	2.14
−2	−4	8	26.67	2.34
−2	−4	8	23.09	2.58
0	−4	8	67.12	3.73
0	−4	8	59.54	3.70

2	−4	8	18.24	2.44
2	−4	8	20.27	2.37
4	−4	8	28.27	3.00
4	−4	8	22.15	2.52
6	−4	8	19.99	2.67
6	−4	8	24.43	3.08
−8	4	−8	4.49	1.74
−10	4	−8	10.27	2.35
−12	4	−8	4.29	1.98
−13	−3	8	6.23	1.95
−11	−3	8	10.09	2.18
−9	−3	8	6.98	1.69
−7	−3	8	29.02	2.92
−7	−3	8	30.47	2.52
−5	−3	8	16.41	1.92
−5	−3	8	17.84	2.31
−3	−3	8	9.04	1.56
−3	−3	8	10.11	1.87
−1	−3	8	31.83	2.44
−1	−3	8	35.04	2.81
1	−3	8	35.23	2.62
1	−3	8	35.39	2.84
3	−3	8	30.98	2.51
3	−3	8	34.31	2.90
5	−3	8	22.56	2.76
7	−3	8	19.54	2.82
−9	3	−8	11.95	2.45
−11	3	−8	7.63	2.23
−13	3	−8	2.02	1.76
−14	−2	8	5.78	1.94
−12	−2	8	3.45	1.54
−10	−2	8	11.32	2.21
−8	−2	8	1.51	1.13
−6	−2	8	23.81	2.61
−6	−2	8	10.01	1.44
−2	−2	8	21.35	2.42
−2	−2	8	21.84	1.84
0	−2	8	29.75	2.62
0	−2	8	28.48	2.18
2	−2	8	13.88	2.06
2	−2	8	14.11	1.77
4	−2	8	10.15	1.94
6	−2	8	5.02	1.56
−8	2	−8	15.90	2.68

−10	2	−8	4.93	1.89
−12	2	−8	0.07	1.32
−15	−1	8	1.37	1.60
−13	−1	8	6.25	2.08
−11	−1	8	3.92	1.51
−9	−1	8	13.31	2.30
−7	−1	8	9.86	1.98
−5	−1	8	10.73	1.97
−3	−1	8	17.91	2.33
−3	−1	8	20.20	1.64
−1	−1	8	6.25	1.51
−1	−1	8	6.04	1.12
1	−1	8	21.09	2.49
1	−1	8	21.52	1.78
3	−1	8	5.71	1.53
5	−1	8	8.47	1.77
7	−1	8	5.31	1.59
−9	1	−8	0.09	1.04
−11	1	−8	0.98	1.38
−13	1	−8	2.73	1.82
−14	0	8	1.55	1.60
−12	0	8	1.80	1.37
−10	0	8	6.20	1.84
−8	0	8	1.40	1.11
−6	0	8	7.57	1.75
−4	0	8	2.10	1.10
−2	0	8	5.45	1.47
0	0	8	1.00	0.64
0	0	8	−0.36	0.72
2	0	8	0.44	0.83
4	0	8	4.32	1.32
6	0	8	−0.86	0.71
8	0	8	0.26	1.02
−10	0	−8	−0.37	0.99
−12	0	−8	4.87	1.92
−14	0	−8	−0.75	1.36
−15	1	8	0.37	1.38
−13	1	8	6.35	2.09
−11	1	8	5.90	1.90
−9	1	8	12.20	2.35
−7	1	8	9.74	1.98
−5	1	8	9.24	1.89
3	−1	−8	17.81	2.08
−3	1	8	13.68	2.08

1	−1	−8	5.28	1.32
−1	1	8	4.80	1.36
−1	−1	−8	24.27	2.06
1	1	8	15.23	2.10
3	1	8	5.88	1.44
5	1	8	6.19	1.48
7	1	8	6.42	1.64
−9	−1	−8	0.14	1.09
−11	−1	−8	3.61	1.60
−13	−1	−8	6.69	2.16
−14	2	8	7.22	2.23
−12	2	8	1.69	1.33
−10	2	8	9.90	2.32
−8	2	8	0.24	1.07
−6	2	8	24.14	2.78
4	−2	−8	41.97	3.13
−4	2	8	35.66	2.95
2	−2	−8	25.13	2.48
−2	2	8	21.50	2.47
0	−2	−8	27.97	2.45
0	2	8	28.96	2.55
−2	−2	−8	18.07	2.10
2	2	8	16.77	2.26
−4	−2	−8	11.43	1.75
4	2	8	8.70	1.72
6	2	8	3.34	1.29
8	2	8	13.34	2.26
−10	−2	−8	5.08	1.73
−12	−2	−8	1.43	1.45
−15	3	8	3.56	1.98
−13	3	8	4.08	1.91
−11	3	8	7.88	2.16
−9	3	8	8.78	2.10
−7	3	8	28.99	3.17
5	−3	−8	23.97	2.81
−5	3	8	20.17	2.58
3	−3	−8	10.32	1.99
−3	3	8	9.01	1.82
1	−3	−8	36.31	2.95
−1	3	8	41.04	3.06
−1	−3	−8	37.27	2.91
1	3	8	38.21	2.91
−3	−3	−8	30.53	2.59
3	3	8	34.83	2.78

−5	−3	−8	25.86	2.73
5	3	8	27.73	2.71
−7	−3	−8	18.40	2.39
7	3	8	17.98	2.39
9	3	8	12.98	2.28
−11	−3	−8	6.16	1.78
−14	4	8	5.01	2.12
−12	4	8	3.69	1.81
−10	4	8	14.19	2.61
−8	4	8	10.23	2.20
6	−4	−8	17.36	2.80
−6	4	8	18.41	2.53
4	−4	−8	19.42	2.72
−4	4	8	17.19	2.41
2	−4	−8	28.55	3.09
−2	4	8	25.91	2.71
0	−4	−8	64.93	4.14
0	4	8	74.26	4.12
−2	−4	−8	23.54	2.65
2	4	8	23.97	2.47
−4	−4	−8	28.31	2.98
4	4	8	28.35	2.49
−6	−4	−8	19.83	2.66
6	4	8	19.82	2.39
−8	−4	−8	5.62	1.75
8	4	8	6.59	1.67
−10	−4	−8	12.06	2.48
−12	−4	−8	2.68	1.90
−13	5	8	0.14	1.29
−11	5	8	5.32	1.78
−9	5	8	8.82	2.17
7	−5	−8	6.36	2.10
−7	5	8	9.14	2.06
5	−5	−8	18.35	2.90
−5	5	8	22.16	2.65
3	−5	−8	18.58	2.80
−3	5	8	19.18	2.43
1	−5	−8	6.88	1.91
−1	5	8	10.44	1.93
−1	−5	−8	2.71	1.50
1	5	8	1.80	1.04
−3	−5	−8	6.80	1.91
3	5	8	8.91	1.78
−5	−5	−8	13.79	2.48

5	5	8	15.43	2.18
−7	−5	−8	2.73	1.58
7	5	8	4.39	1.43
−9	−5	−8	3.14	1.74
−11	−5	−8	2.06	1.77
−12	6	8	0.24	1.27
−10	6	8	4.84	1.84
8	−6	−8	5.26	2.11
−8	6	8	4.37	1.71
6	−6	−8	0.45	1.40
−6	6	8	1.01	0.98
4	−6	−8	2.21	1.67
−4	6	8	3.87	1.43
2	−6	−8	1.51	1.58
−2	6	8	3.46	1.31
0	−6	−8	0.97	1.30
0	6	8	0.44	0.95
−2	−6	−8	4.02	1.60
2	6	8	3.29	1.35
−4	−6	−8	1.15	1.43
4	6	8	2.78	1.20
−6	−6	−8	1.44	1.53
−8	−6	−8	2.35	1.64
−10	−6	−8	−0.57	1.42
−11	7	8	2.14	1.36
9	−7	−8	−0.12	1.36
−9	7	8	0.44	1.07
7	−7	−8	−0.17	1.48
−7	7	8	0.19	0.98
5	−7	−8	1.43	1.56
−5	7	8	0.90	0.98
3	−7	−8	2.75	1.67
−3	7	8	1.99	1.16
1	−7	−8	2.20	1.62
−1	7	8	3.06	1.33
−1	−7	−8	2.31	1.66
1	7	8	2.03	1.17
−3	−7	−8	−0.62	1.30
−5	−7	−8	1.31	1.44
−7	−7	−8	1.85	1.74
−9	−7	−8	2.21	1.78
−10	8	8	2.08	1.33
8	−8	−8	7.28	2.58
−8	8	8	7.59	2.09

6	−8	−8	6.38	2.29
4	−8	−8	6.13	2.33
−4	8	8	3.97	1.51
2	−8	−8	6.47	2.25
−2	8	8	6.84	1.78
0	−8	−8	8.70	2.34
−2	−8	−8	4.76	2.04
−4	−8	−8	5.19	2.09
−6	−8	−8	5.12	2.15
−8	−8	−8	1.32	1.75
7	−9	−8	6.70	2.58
−7	9	8	3.82	1.56
5	−9	−8	9.22	2.78
−5	9	8	8.64	2.02
3	−9	−8	5.90	2.38
1	−9	−8	6.17	2.45
−1	−9	−8	9.14	2.72
−3	−9	−8	7.68	2.50
−5	−9	−8	7.48	2.56
−7	−9	−8	7.28	2.64
4	−10	−8	5.62	2.59
2	−10	−8	4.95	2.33
0	−10	−8	5.93	2.57
−2	−10	−8	7.09	2.58
−4	−10	−8	7.30	2.60
−5	−9	9	3.95	1.82
−3	−9	9	2.15	1.46
−1	−9	9	5.38	1.96
1	−9	9	2.01	1.52
3	−9	9	4.10	1.87
−8	−8	9	6.10	2.05
−6	−8	9	1.92	1.51
−4	−8	9	4.16	1.81
−2	−8	9	4.92	1.80
0	−8	9	2.05	1.61
0	−8	9	0.55	1.29
−2	8	−9	0.35	1.36
2	−8	9	2.38	1.54
−4	8	−9	0.53	1.21
4	−8	9	2.51	1.44
−6	8	−9	3.02	1.60
6	−8	9	0.71	1.27
−9	−7	9	1.69	1.52
−7	−7	9	1.00	1.40

−5	−7	9	6.13	1.92
−3	−7	9	3.31	1.51
−3	−7	9	4.49	1.68
−1	−7	9	−0.42	0.99
−1	−7	9	0.72	1.18
1	−7	9	3.48	1.57
1	−7	9	1.89	1.34
3	−7	9	0.95	1.34
3	−7	9	1.24	1.46
−5	7	−9	3.86	1.65
5	−7	9	1.77	1.36
−7	7	−9	1.80	1.47
7	−7	9	3.19	1.53
−10	−6	9	3.33	1.94
−8	−6	9	2.61	1.56
−6	−6	9	3.36	1.41
−6	−6	9	4.74	1.65
−4	−6	9	1.63	1.28
−4	−6	9	2.00	1.10
−2	−6	9	1.76	1.20
−2	−6	9	3.59	1.45
0	−6	9	1.56	1.24
0	−6	9	2.31	1.34
2	−6	9	1.24	1.22
2	−6	9	1.14	1.26
4	−6	9	2.05	1.48
4	−6	9	3.85	1.55
−6	6	−9	1.32	1.30
6	−6	9	2.62	1.35
−8	6	−9	0.52	1.44
−7	−5	9	4.93	1.72
−7	−5	9	3.43	1.39
−5	−5	9	3.58	1.36
−5	−5	9	3.42	1.41
−3	−5	9	0.76	1.09
−3	−5	9	1.36	1.10
−1	−5	9	1.81	1.16
−1	−5	9	0.73	1.03
1	−5	9	4.00	1.52
1	−5	9	2.51	1.33
3	−5	9	3.11	1.30
3	−5	9	3.45	1.55
5	−5	9	5.61	1.70
5	−5	9	5.36	1.86

−7	5	−9	2.25	1.35
−9	5	−9	−0.09	1.11
−10	−4	9	3.63	1.60
−8	−4	9	2.31	1.36
−6	−4	9	1.84	1.22
−6	−4	9	0.72	1.06
−4	−4	9	8.73	1.85
−4	−4	9	10.00	1.87
−2	−4	9	3.55	1.43
−2	−4	9	3.29	1.24
0	−4	9	9.15	1.82
0	−4	9	12.54	2.22
2	−4	9	8.65	1.77
2	−4	9	9.33	2.00
4	−4	9	7.53	1.96
4	−4	9	8.07	1.81
6	−4	9	1.84	1.48
−8	4	−9	0.12	1.04
−10	4	−9	5.77	2.03
−13	−3	9	2.00	1.57
−11	−3	9	1.64	1.33
−9	−3	9	5.46	1.68
−7	−3	9	12.63	2.33
−5	−3	9	15.73	2.38
−3	−3	9	9.62	1.90
−3	−3	9	5.21	1.36
−1	−3	9	17.34	2.06
−1	−3	9	22.87	2.80
1	−3	9	6.53	1.78
1	−3	9	6.51	1.51
3	−3	9	3.51	1.27
3	−3	9	2.43	1.35
5	−3	9	1.78	1.19
7	−3	9	7.53	2.18
−9	3	−9	6.89	2.12
−11	3	−9	−0.15	1.44
−14	−2	9	7.68	2.33
−12	−2	9	7.57	2.15
−10	−2	9	8.37	2.09
−8	−2	9	9.63	2.04
−6	−2	9	7.59	1.82
−4	−2	9	3.98	1.47
−2	−2	9	0.76	0.93
−2	−2	9	0.91	0.80

0	−2	9	20.57	2.04
0	−2	9	20.28	2.65
2	−2	9	15.71	2.42
2	−2	9	12.93	1.81
4	−2	9	4.61	1.62
6	−2	9	12.64	2.34
−8	2	−9	1.88	1.56
−10	2	−9	1.51	1.37
−12	2	−9	0.23	1.48
−13	−1	9	4.00	1.72
−11	−1	9	0.70	1.32
−9	−1	9	1.98	1.31
−7	−1	9	1.02	1.15
−5	−1	9	7.75	1.83
−3	−1	9	11.62	2.13
−1	−1	9	2.54	1.14
1	−1	9	16.19	2.45
1	−1	9	12.79	1.66
3	−1	9	2.59	1.28
5	−1	9	1.02	1.13
7	−1	9	6.35	1.87
−9	1	−9	2.62	1.63
−11	1	−9	2.66	1.62
−14	0	9	1.21	1.50
−12	0	9	−0.20	1.28
−10	0	9	0.87	1.23
−8	0	9	−0.13	1.06
−6	0	9	0.29	1.08
−4	0	9	8.83	2.00
−2	0	9	1.39	1.11
0	0	9	0.99	0.98
2	0	9	−0.45	0.83
4	0	9	0.98	1.09
6	0	9	−0.05	0.92
8	0	9	1.33	1.19
−10	0	−9	2.44	1.52
−12	0	−9	2.79	1.73
−13	1	9	2.23	1.77
−11	1	9	−0.04	1.14
−9	1	9	2.15	1.36
−7	1	9	1.95	1.28
−5	1	9	9.53	2.10
3	−1	−9	14.30	2.00
−3	1	9	12.79	2.30

−1	1	9	3.98	1.41
1	1	9	15.45	2.35
3	1	9	2.98	1.27
5	1	9	2.96	1.33
7	1	9	5.64	1.65
9	1	9	3.44	1.57
−11	−1	−9	2.26	1.58
−14	2	9	4.55	2.09
−12	2	9	5.31	2.04
−10	2	9	6.01	1.95
−8	2	9	7.00	1.96
−6	2	9	9.22	2.10
4	−2	−9	5.32	1.65
−4	2	9	4.45	1.50
2	−2	−9	2.58	1.30
−2	2	9	1.26	1.09
0	−2	−9	23.82	2.57
0	2	9	27.66	2.96
2	2	9	16.17	2.45
4	2	9	7.88	1.74
6	2	9	10.90	2.09
8	2	9	1.08	1.11
−10	−2	−9	0.37	1.30
−12	−2	−9	0.07	1.39
−13	3	9	1.26	1.64
−11	3	9	3.51	1.79
−9	3	9	4.01	1.60
−7	3	9	8.38	2.03
5	−3	−9	18.44	2.69
−5	3	9	16.46	2.59
3	−3	−9	7.38	1.88
−3	3	9	7.76	1.86
1	−3	−9	18.77	2.55
−1	3	9	24.73	2.85
−1	−3	−9	7.26	1.75
1	3	9	8.29	1.77
3	3	9	4.17	1.41
5	3	9	4.89	1.55
7	3	9	4.97	1.59
9	3	9	6.37	1.85
−11	−3	−9	1.14	1.49
−12	4	9	2.83	1.76
−10	4	9	4.70	1.73
−8	4	9	0.70	0.99

6	−4	−9	2.57	1.59
−6	4	9	1.51	1.11
4	−4	−9	10.80	2.32
−4	4	9	9.90	2.06
2	−4	−9	2.99	1.53
−2	4	9	5.37	1.51
0	−4	−9	10.78	2.18
0	4	9	10.91	2.03
−2	−4	−9	9.68	2.11
2	4	9	7.72	1.80
−4	−4	−9	5.94	1.78
4	4	9	8.32	1.84
6	4	9	1.53	1.19
8	4	9	1.32	1.21
−11	5	9	5.09	1.87
−9	5	9	3.05	1.59
7	−5	−9	4.51	1.97
−7	5	9	5.20	1.73
5	−5	−9	4.79	1.94
−5	5	9	4.38	1.58
3	−5	−9	1.94	1.57
−3	5	9	2.33	1.26
1	−5	−9	0.02	1.22
−1	5	9	1.32	1.10
−1	−5	−9	1.65	1.45
1	5	9	3.61	1.38
−3	−5	−9	2.57	1.46
3	5	9	2.88	1.30
−5	−5	−9	6.60	2.03
5	5	9	7.94	1.85
−7	−5	−9	0.30	1.49
−10	6	9	4.04	1.76
8	−6	−9	3.33	1.91
−8	6	9	3.78	1.61
6	−6	−9	4.66	1.98
−6	6	9	3.60	1.46
4	−6	−9	2.14	1.58
−4	6	9	2.85	1.35
2	−6	−9	4.44	1.95
−2	6	9	2.70	1.32
0	−6	−9	0.88	1.55
0	6	9	3.42	1.31
−2	−6	−9	2.15	1.57
2	6	9	0.71	1.11

−4	−6	−9	3.06	1.64
4	6	9	2.49	1.36
−6	−6	−9	0.90	1.58
−8	−6	−9	1.01	1.76
9	−7	−9	0.26	1.71
−9	7	9	2.03	1.40
7	−7	−9	0.71	1.81
−7	7	9	0.60	1.08
5	−7	−9	2.59	1.81
−5	7	9	3.10	1.47
3	−7	−9	6.14	2.28
−3	7	9	5.25	1.62
1	−7	−9	−1.08	1.20
−1	7	9	−0.85	0.90
−1	−7	−9	4.03	1.97
1	7	9	2.44	1.36
−3	−7	−9	0.33	1.41
−5	−7	−9	1.99	1.76
−7	−7	−9	3.53	1.97
8	−8	−9	3.56	2.30
−8	8	9	1.98	1.46
6	−8	−9	0.80	1.79
−6	8	9	1.70	1.23
4	−8	−9	2.54	1.91
−4	8	9	1.94	1.39
2	−8	−9	2.51	2.08
−2	8	9	3.01	1.50
0	−8	−9	2.67	1.97
−2	−8	−9	1.55	1.82
−4	−8	−9	0.17	1.46
−6	−8	−9	−1.26	1.69
5	−9	−9	1.89	2.06
3	−9	−9	−0.79	1.77
1	−9	−9	3.86	2.24
−1	−9	−9	0.50	1.75
−3	−9	−9	1.91	1.99
−4	−8	10	6.12	1.89
−2	−8	10	3.08	1.51
0	−8	10	4.08	1.80
−2	8	−10	3.36	1.83
2	−8	10	1.51	1.50
−7	−7	10	3.15	1.71
−5	−7	10	2.65	1.57
−3	−7	10	4.30	1.64

−1	−7	10	2.60	1.38
−1	−7	10	4.26	1.80
1	−7	10	6.67	2.09
1	−7	10	6.38	1.94
3	−7	10	4.11	1.60
3	−7	10	5.44	2.08
−5	7	−10	7.31	2.08
5	−7	10	3.67	1.64
−6	−6	10	8.66	2.22
−4	−6	10	8.55	2.01
−4	−6	10	6.71	1.84
−2	−6	10	7.01	1.87
−2	−6	10	5.69	1.87
0	−6	10	6.83	1.81
0	−6	10	5.60	1.89
2	−6	10	4.83	1.62
2	−6	10	8.15	2.19
4	−6	10	1.79	1.23
4	−6	10	6.95	2.34
−6	6	−10	4.14	1.83
6	−6	10	3.56	1.70
−7	−5	10	2.95	1.43
−5	−5	10	1.92	1.42
−3	−5	10	4.28	1.59
−3	−5	10	4.50	1.54
−1	−5	10	2.90	1.43
−1	−5	10	1.99	1.35
1	−5	10	5.29	1.87
1	−5	10	5.67	1.72
3	−5	10	0.90	1.17
3	−5	10	2.47	1.51
5	−5	10	4.10	2.00
5	−5	10	2.38	1.38
−7	5	−10	0.82	1.26
−10	−4	10	0.19	1.40
−8	−4	10	1.43	1.42
−6	−4	10	3.42	1.57
−4	−4	10	3.48	1.51
−2	−4	10	3.50	1.37
−2	−4	10	0.83	1.17
0	−4	10	−0.83	0.99
0	−4	10	0.52	1.08
2	−4	10	3.61	1.44
2	−4	10	3.65	1.71

4	−4	10	1.50	1.35
4	−4	10	0.99	1.12
6	−4	10	0.39	1.50
−8	4	−10	1.12	1.42
−11	−3	10	0.94	1.50
−9	−3	10	1.94	1.46
−7	−3	10	2.14	1.44
−5	−3	10	5.62	1.86
−3	−3	10	5.62	1.77
−1	−3	10	3.64	1.50
1	−3	10	3.62	1.35
1	−3	10	2.48	1.38
3	−3	10	2.27	1.21
3	−3	10	4.43	1.76
5	−3	10	−0.70	0.98
7	−3	10	0.53	1.50
−9	3	−10	1.00	1.33
−12	−2	10	0.77	1.47
−10	−2	10	1.91	1.45
−8	−2	10	4.13	1.66
−6	−2	10	2.28	1.39
−4	−2	10	7.41	1.94
−2	−2	10	15.03	2.56
0	−2	10	9.14	2.11
2	−2	10	2.12	1.42
4	−2	10	3.45	1.65
6	−2	10	8.56	2.16
8	−2	10	2.44	1.45
−10	2	−10	0.77	1.44
−11	−1	10	0.96	1.39
−9	−1	10	6.78	2.08
−7	−1	10	4.98	1.76
−5	−1	10	10.38	2.26
−3	−1	10	12.16	2.42
−1	−1	10	14.07	2.50
1	−1	10	25.14	3.11
3	−1	10	9.21	2.10
5	−1	10	8.65	2.15
7	−1	10	5.63	1.80
−9	1	−10	1.70	1.61
−12	0	10	2.67	1.75
−10	0	10	3.79	1.75
−8	0	10	3.01	1.63
−6	0	10	10.07	2.23

−4	0	10	14.70	2.63
−2	0	10	1.14	1.25
0	0	10	7.51	1.97
2	0	10	11.53	2.25
4	0	10	5.48	1.85
6	0	10	3.36	1.49
8	0	10	9.54	2.32
−10	0	−10	0.08	1.49
−11	1	10	1.73	1.54
−9	1	10	6.02	1.96
−7	1	10	4.06	1.75
−5	1	10	7.84	1.99
−3	1	10	7.78	2.00
−1	1	10	15.69	2.53
1	1	10	23.50	3.04
3	1	10	11.99	2.29
5	1	10	6.63	1.83
7	1	10	6.54	1.85
9	1	10	1.84	1.44
−12	2	10	2.95	1.71
−10	2	10	3.50	1.89
−8	2	10	3.51	1.69
−6	2	10	1.95	1.30
−4	2	10	9.08	2.13
−2	2	10	15.62	2.52
0	2	10	19.08	2.74
2	2	10	2.13	1.23
4	2	10	4.49	1.68
6	2	10	9.44	2.16
8	2	10	3.59	1.56
−10	−2	−10	0.50	1.49
−11	3	10	0.85	1.33
−9	3	10	1.13	1.20
−7	3	10	0.70	1.17
5	−3	−10	4.53	1.71
−5	3	10	5.81	1.79
3	−3	−10	4.10	1.71
−3	3	10	4.09	1.60
−1	3	10	4.52	1.51
1	3	10	4.97	1.67
3	3	10	3.77	1.46
5	3	10	0.17	1.05
7	3	10	0.12	1.08
9	3	10	0.52	1.13

−10	4	10	0.50	1.25
−8	4	10	1.06	1.20
6	−4	−10	6.59	2.05
−6	4	10	5.85	1.85
4	−4	−10	3.80	1.72
−4	4	10	2.51	1.40
2	−4	−10	1.45	1.34
−2	4	10	0.61	1.13
0	−4	−10	0.43	1.31
0	4	10	−0.69	0.82
2	4	10	5.12	1.74
4	4	10	1.70	1.12
6	4	10	1.37	1.25
8	4	10	0.85	1.24
−9	5	10	0.89	1.17
7	−5	−10	1.40	1.50
−7	5	10	0.59	1.13
5	−5	−10	2.13	1.75
−5	5	10	1.64	1.20
3	−5	−10	4.13	1.89
−3	5	10	2.99	1.47
1	−5	−10	3.27	1.86
−1	5	10	4.77	1.63
−1	−5	−10	8.10	2.18
1	5	10	4.18	1.51
3	5	10	2.42	1.35
5	5	10	1.10	1.27
−8	6	10	4.52	1.68
6	−6	−10	8.33	2.59
−6	6	10	6.24	1.98
4	−6	−10	7.07	2.36
−4	6	10	7.30	2.00
2	−6	−10	8.98	2.58
−2	6	10	5.46	1.68
0	−6	−10	7.35	2.35
0	6	10	5.84	1.81
−2	−6	−10	5.81	2.14
2	6	10	6.15	1.87
−4	−6	−10	4.76	2.08
7	−7	−10	3.95	2.39
−7	7	10	2.50	1.35
5	−7	−10	0.56	1.87
−5	7	10	3.13	1.49
3	−7	−10	5.62	2.23

-3	7	10	2.96	1.50
1	-7	-10	3.34	2.14
-1	7	10	1.74	1.34
-1	-7	-10	5.73	2.23
-3	-7	-10	3.71	2.21
-5	-7	-10	4.48	2.12
4	-8	-10	7.45	2.57
-4	8	10	4.40	1.69
2	-8	-10	3.57	2.18
0	-8	-10	3.25	2.13
-2	-8	-10	6.24	2.40
-4	-6	11	3.28	1.68
-2	-6	11	3.81	1.88
0	-6	11	4.10	1.95
0	-6	11	2.18	1.46
2	-6	11	3.57	1.58
2	-6	11	5.32	2.04
-7	-5	11	6.06	2.00
-5	-5	11	5.24	1.80
-3	-5	11	2.93	1.63
-1	-5	11	6.10	2.04
1	-5	11	3.39	1.79
1	-5	11	4.12	1.56
3	-5	11	2.89	1.81
3	-5	11	2.98	1.52
5	-5	11	1.97	1.77
-8	-4	11	1.73	1.56
-6	-4	11	1.77	1.37
-4	-4	11	6.43	1.98
-2	-4	11	2.22	1.36
0	-4	11	0.18	1.24
2	-4	11	2.47	1.60
4	-4	11	1.14	1.43
6	-4	11	0.84	1.67
-9	-3	11	-0.76	1.25
-7	-3	11	1.62	1.47
-5	-3	11	-0.81	1.01
-3	-3	11	-0.42	1.29
-1	-3	11	2.85	1.54
1	-3	11	-0.25	1.17
3	-3	11	1.15	1.48
5	-3	11	1.04	1.41
7	-3	11	0.46	1.46
-10	-2	11	1.36	1.59

−8	−2	11	0.39	1.23
−6	−2	11	3.14	1.70
−4	−2	11	2.96	1.61
−2	−2	11	5.31	1.90
0	−2	11	1.08	1.36
2	−2	11	3.19	1.71
4	−2	11	1.27	1.65
6	−2	11	0.55	1.30
−9	−1	11	2.12	1.62
−7	−1	11	1.93	1.55
−5	−1	11	10.17	2.39
−3	−1	11	8.45	2.29
−1	−1	11	1.29	1.41
1	−1	11	9.65	2.37
3	−1	11	4.97	1.84
5	−1	11	5.04	1.71
7	−1	11	6.48	2.01
−10	0	11	0.74	1.39
−8	0	11	8.27	2.35
−6	0	11	3.07	1.69
−4	0	11	2.45	1.60
−2	0	11	7.71	2.15
0	0	11	18.76	3.02
2	0	11	17.71	2.85
4	0	11	2.20	1.36
6	0	11	4.95	1.85
8	0	11	2.49	1.60
−9	1	11	4.12	1.93
−7	1	11	2.90	1.68
−5	1	11	9.76	2.45
−3	1	11	9.02	2.13
−1	1	11	1.99	1.21
1	1	11	7.08	1.97
3	1	11	5.87	1.89
5	1	11	5.18	1.80
7	1	11	3.72	1.64
−10	2	11	0.74	1.53
−8	2	11	0.79	1.47
−6	2	11	2.14	1.42
−4	2	11	3.61	1.54
−2	2	11	6.00	1.96
0	2	11	1.83	1.25
2	2	11	5.17	1.81
4	2	11	2.42	1.28

6	2	11	2.48	1.48
−9	3	11	0.53	1.50
−7	3	11	1.30	1.38
−5	3	11	−0.59	0.90
−3	3	11	2.05	1.27
−1	3	11	4.19	1.64
1	3	11	0.54	1.10
3	3	11	0.55	1.21
5	3	11	1.01	1.20
7	3	11	−0.84	1.05
−8	4	11	0.47	1.39
−6	4	11	0.63	1.18
−4	4	11	3.06	1.56
−2	4	11	4.28	1.61
0	4	11	1.55	1.20
2	4	11	1.57	1.26
4	4	11	0.09	1.15
6	4	11	−0.35	1.11
−7	5	11	10.15	2.42
5	−5	−11	7.74	2.50
−5	5	11	2.95	1.60
−3	5	11	2.70	1.55
−1	5	11	3.95	1.61
1	5	11	3.34	1.59
3	5	11	0.42	1.20
5	5	11	−0.11	1.32
4	−6	−11	5.66	2.43
−4	6	11	4.67	1.72
2	−6	−11	1.56	1.84
−2	6	11	3.10	1.51
0	6	11	3.57	1.54
2	6	11	5.30	1.79
−2	−4	12	4.43	1.91
0	−4	12	0.15	1.45
−5	−3	12	−0.03	1.36
−3	−3	12	1.46	1.60
−1	−3	12	1.10	1.49
1	−3	12	2.24	1.78
3	−3	12	0.31	1.55
−6	−2	12	1.99	1.61
−4	−2	12	1.31	1.65
−2	−2	12	0.93	1.47
0	−2	12	1.49	1.45
2	−2	12	7.51	2.34

```
   4  -2  12     0.10     1.27
  -7  -1  12     1.47     1.62
  -5  -1  12     0.99     1.54
  -3  -1  12     3.99     1.99
  -1  -1  12     0.04     1.33
   1  -1  12     4.04     1.83
   3  -1  12     1.29     1.42
  -6   0  12     2.00     1.59
  -4   0  12     2.94     1.79
  -2   0  12     2.82     1.78
   0   0  12     2.18     1.45
   2   0  12     2.60     1.57
   4   0  12     2.78     1.52
  -7   1  12     2.54     1.63
  -5   1  12     1.67     1.54
  -3   1  12     5.05     1.77
  -1   1  12     2.48     1.48
   1   1  12     4.03     1.65
   3   1  12     0.88     1.15
  -6   2  12     5.34     1.96
  -4   2  12     0.49     1.12
  -2   2  12    -0.56     1.02
   0   2  12    -0.28     1.08
   2   2  12     3.20     1.53
   4   2  12     1.43     1.53
  -5   3  12     4.29     1.97
  -3   3  12     3.71     1.74
  -1   3  12     0.95     1.19
   1   3  12     1.97     1.30
   3   3  12     3.31     1.59
  -2   4  12     5.06     1.70
   0   4  12     2.96     1.56
   0   0   0     0.00     0.00
;
_shelx_hkl_checksum   63976


data_compound_8

_audit_creation_method            SHELXL-97
_chemical_name_systematic
;
 ?
;
```

```
_chemical_name_common             ?
_chemical_melting_point           ?
_chemical_formula_moiety          ?
_chemical_formula_sum
 'C11 H14 Cl2 N2 O Zn'
_chemical_formula_weight          326.51

loop_
 _atom_type_symbol
 _atom_type_description
 _atom_type_scat_dispersion_real
 _atom_type_scat_dispersion_imag
 _atom_type_scat_source
 'C'  'C'   0.0033   0.0016
 'International Tables Vol C Tables 4.2.6.8 and 6.1.1.4'
 'H'  'H'   0.0000   0.0000
 'International Tables Vol C Tables 4.2.6.8 and 6.1.1.4'
 'N'  'N'   0.0061   0.0033
 'International Tables Vol C Tables 4.2.6.8 and 6.1.1.4'
 'O'  'O'   0.0106   0.0060
 'International Tables Vol C Tables 4.2.6.8 and 6.1.1.4'
 'Cl'  'Cl'   0.1484   0.1585
 'International Tables Vol C Tables 4.2.6.8 and 6.1.1.4'
 'Zn'  'Zn'   0.2839   1.4301
 'International Tables Vol C Tables 4.2.6.8 and 6.1.1.4'

_symmetry_cell_setting            Monoclinic
_symmetry_space_group_name_H-M    P2(1)

loop_
 _symmetry_equiv_pos_as_xyz
 'x, y, z'
 '-x, y+1/2, -z'

_cell_length_a                    9.3099(9)
_cell_length_b                    9.4799(9)
_cell_length_c                    15.8234(14)
_cell_angle_alpha                 90.00
_cell_angle_beta                  92.554(2)
_cell_angle_gamma                 90.00
_cell_volume                      1395.1(2)
_cell_formula_units_Z             4
_cell_measurement_temperature     293(2)
_cell_measurement_reflns_used     3140
```

```
_cell_measurement_theta_min       4.982
_cell_measurement_theta_max       51.002

_exptl_crystal_description        prismatic
_exptl_crystal_colour             colorless
_exptl_crystal_size_max           0.212
_exptl_crystal_size_mid           0.167
_exptl_crystal_size_min           0.132
_exptl_crystal_density_meas       ?
_exptl_crystal_density_diffrn     1.555
_exptl_crystal_density_method     'not measured'
_exptl_crystal_F_000              664
_exptl_absorpt_coefficient_mu     2.128
_exptl_absorpt_correction_type    empirical
_exptl_absorpt_correction_T_min   0.63880
_exptl_absorpt_correction_T_max   1.00000
_exptl_absorpt_process_details    sadabs

_exptl_special_details
;
 ?
;

_diffrn_ambient_temperature       293(2)
_diffrn_radiation_wavelength      0.71073
_diffrn_radiation_type            MoK\a
_diffrn_radiation_source          'fine-focus sealed tube'
_diffrn_radiation_monochromator   graphite
_diffrn_measurement_device_type   'CCD area detector'
_diffrn_measurement_method        'phi and omega scans'
_diffrn_detector_area_resol_mean  ?
_diffrn_standards_number          ?
_diffrn_standards_interval_count  ?
_diffrn_standards_interval_time   ?
_diffrn_standards_decay_%         ?
_diffrn_reflns_number             8481
_diffrn_reflns_av_R_equivalents   0.0272
_diffrn_reflns_av_sigmaI/netI     0.0440
_diffrn_reflns_limit_h_min        -11
_diffrn_reflns_limit_h_max        11
_diffrn_reflns_limit_k_min        -11
_diffrn_reflns_limit_k_max        8
_diffrn_reflns_limit_l_min        -17
_diffrn_reflns_limit_l_max        19
```

```
_diffrn_reflns_theta_min          2.19
_diffrn_reflns_theta_max          25.97
_reflns_number_total              4331
_reflns_number_gt                 3722
_reflns_threshold_expression      >2sigma(I)

_computing_data_collection        'Bruker SMART'
_computing_cell_refinement        'Bruker SMART'
_computing_data_reduction         'Bruker SHELXTL'
_computing_structure_solution     'SHELXS-97 (Sheldrick, 1990)'
_computing_structure_refinement   'SHELXL-97 (Sheldrick, 1997)'
_computing_molecular_graphics     'Bruker SHELXTL'
_computing_publication_material   'Bruker SHELXTL'

_refine_special_details
;
 Refinement of F^2^ against ALL reflections.  The weighted R-factor wR and
 goodness of fit S are based on F^2^, conventional R-factors R are based
 on F, with F set to zero for negative F^2^. The threshold expression of
 F^2^ > 2sigma(F^2^) is used only for calculating R-factors(gt) etc. and is
 not relevant to the choice of reflections for refinement.  R-factors based
 on F^2^ are statistically about twice as large as those based on F, and R-
 factors based on ALL data will be even larger.
;

_refine_ls_structure_factor_coef  Fsqd
_refine_ls_matrix_type            full
_refine_ls_weighting_scheme       calc
_refine_ls_weighting_details
 'calc w=1/[\s^2^(Fo^2^)+(0.0513P)^2^+0.0000P] where P=(Fo^2^+2Fc^2^)/3'
_atom_sites_solution_primary      direct
_atom_sites_solution_secondary    difmap
_atom_sites_solution_hydrogens    geom
_refine_ls_hydrogen_treatment     constr
_refine_ls_extinction_method      none
_refine_ls_extinction_coef        ?
_refine_ls_abs_structure_details
 'Flack H D (1983), Acta Cryst. A39, 876-881'
_refine_ls_abs_structure_Flack    0.005(13)
_chemical_absolute_configuration  ad
_refine_ls_number_reflns          4331
_refine_ls_number_parameters      311
_refine_ls_number_restraints      1
_refine_ls_R_factor_all           0.0435
```

_refine_ls_R_factor_gt 0.0366
_refine_ls_wR_factor_ref 0.0930
_refine_ls_wR_factor_gt 0.0882
_refine_ls_goodness_of_fit_ref 1.004
_refine_ls_restrained_S_all 1.004
_refine_ls_shift/su_max 0.000
_refine_ls_shift/su_mean 0.000

loop_
_atom_site_label
_atom_site_type_symbol
_atom_site_fract_x
_atom_site_fract_y
_atom_site_fract_z
_atom_site_U_iso_or_equiv
_atom_site_adp_type
_atom_site_occupancy
_atom_site_symmetry_multiplicity
_atom_site_calc_flag
_atom_site_refinement_flags
_atom_site_disorder_assembly
_atom_site_disorder_group
Zn1 Zn 0.42487(6) 0.28500(6) 0.34068(3) 0.05325(16) Uani 1 1 d . . .
Zn2 Zn 0.03526(6) 0.92037(5) 0.15525(3) 0.05708(16) Uani 1 1 d . . .
Cl1 Cl 0.22155(14) 0.2646(2) 0.40422(8) 0.0780(4) Uani 1 1 d . . .
Cl2 Cl 0.57729(15) 0.45167(15) 0.38326(8) 0.0709(4) Uani 1 1 d . . .
Cl3 Cl -0.11624(14) 0.75682(14) 0.10694(8) 0.0679(3) Uani 1 1 d . . .
Cl4 Cl 0.24456(14) 0.9388(2) 0.09708(9) 0.0800(4) Uani 1 1 d . . .
N1 N 0.5284(4) 0.0868(4) 0.3346(2) 0.0492(9) Uani 1 1 d . . .
N2 N 0.4190(4) 0.2566(4) 0.21280(19) 0.0488(9) Uani 1 1 d . . .
N3 N -0.0466(4) 1.1272(5) 0.1639(2) 0.0549(10) Uani 1 1 d . . .
N4 N 0.0240(4) 0.9374(5) 0.2840(2) 0.0555(9) Uani 1 1 d . . .
O1 O 0.5247(4) 0.1357(4) 0.11136(17) 0.0596(8) Uani 1 1 d . . .
O2 O -0.0689(4) 1.0675(4) 0.38504(19) 0.0667(10) Uani 1 1 d . . .
C1 C 0.5630(6) -0.0059(7) 0.3947(3) 0.0646(14) Uani 1 1 d . . .
H1 H 0.5450 0.0173 0.4504 0.078 Uiso 1 1 calc R . .
C2 C 0.6239(6) -0.1339(6) 0.3791(3) 0.0720(15) Uani 1 1 d . . .
H2 H 0.6449 -0.1968 0.4230 0.086 Uiso 1 1 calc R . .
C3 C 0.6535(6) -0.1678(7) 0.2970(3) 0.0734(16) Uani 1 1 d . . .
H3 H 0.6960 -0.2537 0.2846 0.088 Uiso 1 1 calc R . .
C4 C 0.6192(5) -0.0727(6) 0.2335(3) 0.0590(11) Uani 1 1 d . . .
H4 H 0.6380 -0.0930 0.1775 0.071 Uiso 1 1 calc R . .
C5 C 0.5569(4) 0.0521(5) 0.2545(2) 0.0465(10) Uani 1 1 d . . .
C6 C 0.4986(5) 0.1552(5) 0.1926(2) 0.0461(10) Uani 1 1 d . . .

```
C7 C 0.4449(6) 0.2478(6) 0.0649(3) 0.0675(14) Uani 1 1 d . . .
H7A H 0.3817 0.2082 0.0206 0.081 Uiso 1 1 calc R . .
H7B H 0.5103 0.3137 0.0398 0.081 Uiso 1 1 calc R . .
C8 C 0.3582(5) 0.3203(5) 0.1326(2) 0.0527(11) Uani 1 1 d . . .
H8 H 0.2579 0.2897 0.1249 0.063 Uiso 1 1 calc R . .
C9 C 0.3605(5) 0.4798(5) 0.1323(3) 0.0549(12) Uani 1 1 d . . .
H9 H 0.3066 0.5100 0.1809 0.066 Uiso 1 1 calc R . .
C10 C 0.5059(7) 0.5466(7) 0.1428(4) 0.0850(18) Uani 1 1 d . . .
H10A H 0.5527 0.5145 0.1945 0.128 Uiso 1 1 calc R . .
H10B H 0.4956 0.6473 0.1445 0.128 Uiso 1 1 calc R . .
H10C H 0.5626 0.5209 0.0960 0.128 Uiso 1 1 calc R . .
C11 C 0.2774(6) 0.5348(7) 0.0535(3) 0.0827(17) Uani 1 1 d . . .
H11A H 0.2740 0.6359 0.0553 0.124 Uiso 1 1 calc R . .
H11B H 0.1813 0.4979 0.0520 0.124 Uiso 1 1 calc R . .
H11C H 0.3247 0.5051 0.0039 0.124 Uiso 1 1 calc R . .
C12 C -0.0673(5) 1.2241(6) 0.1034(3) 0.0627(14) Uani 1 1 d . . .
H12 H -0.0514 1.2006 0.0475 0.075 Uiso 1 1 calc R . .
C13 C -0.1124(6) 1.3602(7) 0.1229(4) 0.0734(16) Uani 1 1 d . . .
H13 H -0.1265 1.4267 0.0801 0.088 Uiso 1 1 calc R . .
C14 C -0.1358(6) 1.3954(7) 0.2042(3) 0.0770(16) Uani 1 1 d . . .
H14 H -0.1652 1.4863 0.2174 0.092 Uiso 1 1 calc R . .
C15 C -0.1157(5) 1.2952(7) 0.2680(3) 0.0664(13) Uani 1 1 d . . .
H15 H -0.1323 1.3165 0.3241 0.080 Uiso 1 1 calc R . .
C16 C -0.0705(5) 1.1640(5) 0.2446(3) 0.0516(11) Uani 1 1 d . . .
C17 C -0.0353(5) 1.0510(6) 0.3047(3) 0.0532(12) Uani 1 1 d . . .
C18 C -0.0026(6) 0.9482(7) 0.4299(3) 0.0733(15) Uani 1 1 d . . .
H18A H -0.0707 0.9029 0.4658 0.088 Uiso 1 1 calc R . .
H18B H 0.0803 0.9783 0.4645 0.088 Uiso 1 1 calc R . .
C19 C 0.0424(6) 0.8476(6) 0.3594(3) 0.0666(13) Uani 1 1 d . . .
H19 H -0.0236 0.7671 0.3550 0.080 Uiso 1 1 calc R . .
C20 C 0.1982(7) 0.7953(8) 0.3728(3) 0.0853(18) Uani 1 1 d . . .
H20 H 0.2637 0.8762 0.3769 0.102 Uiso 1 1 calc R . .
C21 C 0.2117(9) 0.7109(10) 0.4517(3) 0.126(3) Uani 1 1 d . . .
H21A H 0.1488 0.6309 0.4471 0.190 Uiso 1 1 calc R . .
H21B H 0.1860 0.7682 0.4987 0.190 Uiso 1 1 calc R . .
H21C H 0.3092 0.6792 0.4604 0.190 Uiso 1 1 calc R . .
C22 C 0.2382(8) 0.7010(9) 0.2996(4) 0.123(3) Uani 1 1 d . . .
H22A H 0.3370 0.6728 0.3072 0.185 Uiso 1 1 calc R . .
H22B H 0.2250 0.7520 0.2474 0.185 Uiso 1 1 calc R . .
H22C H 0.1779 0.6189 0.2980 0.185 Uiso 1 1 calc R . .

loop_
 _atom_site_aniso_label
 _atom_site_aniso_U_11
```

```
 _atom_site_aniso_U_22
 _atom_site_aniso_U_33
 _atom_site_aniso_U_23
 _atom_site_aniso_U_13
 _atom_site_aniso_U_12
Zn1 0.0672(3) 0.0516(3) 0.0416(2) -0.0053(2) 0.00928(19) 0.0017(3)
Zn2 0.0675(3) 0.0481(3) 0.0555(3) -0.0088(3) 0.0014(2) 0.0034(3)
Cl1 0.0727(7) 0.0990(12) 0.0639(7) -0.0154(8) 0.0212(5) -0.0081(8)
Cl2 0.0837(8) 0.0620(9) 0.0669(7) -0.0127(7) 0.0033(6) -0.0097(7)
Cl3 0.0798(8) 0.0504(8) 0.0735(7) -0.0122(6) 0.0037(6) -0.0070(6)
Cl4 0.0716(7) 0.0768(11) 0.0926(9) -0.0122(8) 0.0154(6) -0.0078(8)
N1 0.063(2) 0.039(2) 0.0446(19) -0.0002(17) 0.0007(16) -0.0070(18)
N2 0.064(2) 0.042(2) 0.0412(16) -0.0012(17) 0.0062(14) 0.0043(19)
N3 0.051(2) 0.050(2) 0.064(2) -0.001(2) -0.0011(17) -0.0036(19)
N4 0.069(2) 0.049(3) 0.0486(19) -0.0021(19) -0.0019(16) 0.008(2)
O1 0.081(2) 0.054(2) 0.0451(16) -0.0028(16) 0.0183(14) 0.0056(18)
O2 0.078(2) 0.057(2) 0.067(2) -0.0025(19) 0.0228(16) 0.0005(19)
C1 0.080(4) 0.065(4) 0.048(3) 0.008(3) -0.003(2) -0.005(3)
C2 0.086(4) 0.052(3) 0.076(3) 0.014(3) -0.016(3) -0.002(3)
C3 0.076(3) 0.052(4) 0.090(4) 0.000(3) -0.014(3) 0.010(3)
C4 0.061(2) 0.052(3) 0.064(3) -0.009(3) 0.003(2) 0.003(3)
C5 0.048(2) 0.042(3) 0.050(2) -0.002(2) 0.0037(17) -0.007(2)
C6 0.059(3) 0.041(3) 0.038(2) -0.0015(19) 0.0077(18) -0.008(2)
C7 0.108(4) 0.051(3) 0.044(2) 0.006(2) 0.007(2) 0.004(3)
C8 0.065(3) 0.047(3) 0.045(2) 0.003(2) -0.0025(19) -0.004(2)
C9 0.069(3) 0.044(3) 0.052(2) -0.003(2) 0.001(2) 0.005(2)
C10 0.102(5) 0.069(4) 0.082(4) 0.011(3) -0.014(3) -0.018(4)
C11 0.097(4) 0.074(4) 0.077(3) 0.019(3) -0.003(3) 0.009(3)
C12 0.064(3) 0.062(4) 0.061(3) 0.003(3) -0.008(2) -0.001(3)
C13 0.076(4) 0.061(4) 0.082(4) 0.011(3) -0.013(3) 0.005(3)
C14 0.085(4) 0.052(4) 0.093(4) -0.007(3) -0.001(3) 0.011(3)
C15 0.077(3) 0.053(3) 0.070(3) -0.008(3) 0.010(2) 0.007(3)
C16 0.047(2) 0.046(3) 0.062(3) -0.006(2) 0.0050(19) 0.001(2)
C17 0.050(2) 0.053(3) 0.057(3) -0.009(2) 0.0086(19) -0.003(2)
C18 0.093(4) 0.073(4) 0.055(3) 0.008(3) 0.016(2) 0.003(3)
C19 0.084(4) 0.056(3) 0.059(3) 0.001(2) -0.002(2) 0.001(3)
C20 0.132(5) 0.072(4) 0.050(2) 0.005(3) -0.007(3) 0.028(4)
C21 0.189(9) 0.117(7) 0.074(4) 0.025(4) 0.009(5) 0.039(6)
C22 0.142(7) 0.133(7) 0.093(4) 0.006(5) -0.005(4) 0.073(6)

_geom_special_details
;
 All esds (except the esd in the dihedral angle between two l.s. planes)
 are estimated using the full covariance matrix.  The cell esds are taken
```

```
 into account individually in the estimation of esds in distances, angles
 and torsion angles; correlations between esds in cell parameters are only
 used when they are defined by crystal symmetry.  An approximate (isotropic)
 treatment of cell esds is used for estimating esds involving l.s. planes.
;

loop_
 _geom_bond_atom_site_label_1
 _geom_bond_atom_site_label_2
 _geom_bond_distance
 _geom_bond_site_symmetry_2
 _geom_bond_publ_flag
Zn1 N2 2.039(3) . ?
Zn1 N1 2.116(4) . ?
Zn1 Cl1 2.1911(12) . ?
Zn1 Cl2 2.2089(15) . ?
Zn2 N4 2.051(3) . ?
Zn2 N3 2.110(4) . ?
Zn2 Cl4 2.1988(14) . ?
Zn2 Cl3 2.2094(14) . ?
N1 C1 1.325(6) . ?
N1 C5 1.347(5) . ?
N2 C6 1.264(6) . ?
N2 C8 1.493(5) . ?
N3 C12 1.335(7) . ?
N3 C16 1.352(6) . ?
N4 C17 1.260(7) . ?
N4 C19 1.469(6) . ?
O1 C6 1.331(5) . ?
O1 C7 1.474(6) . ?
O2 C17 1.332(5) . ?
O2 C18 1.457(7) . ?
C1 C2 1.366(8) . ?
C1 H1 0.9300 . ?
C2 C3 1.379(7) . ?
C2 H2 0.9300 . ?
C3 C4 1.377(7) . ?
C3 H3 0.9300 . ?
C4 C5 1.365(7) . ?
C4 H4 0.9300 . ?
C5 C6 1.470(6) . ?
C7 C8 1.533(7) . ?
C7 H7A 0.9700 . ?
C7 H7B 0.9700 . ?
```

C8 C9 1.512(6) . ?
C8 H8 0.9800 . ?
C9 C10 1.497(8) . ?
C9 C11 1.529(6) . ?
C9 H9 0.9800 . ?
C10 H10A 0.9600 . ?
C10 H10B 0.9600 . ?
C10 H10C 0.9600 . ?
C11 H11A 0.9600 . ?
C11 H11B 0.9600 . ?
C11 H11C 0.9600 . ?
C12 C13 1.396(8) . ?
C12 H12 0.9300 . ?
C13 C14 1.357(7) . ?
C13 H13 0.9300 . ?
C14 C15 1.392(8) . ?
C14 H14 0.9300 . ?
C15 C16 1.369(8) . ?
C15 H15 0.9300 . ?
C16 C17 1.460(7) . ?
C18 C19 1.540(7) . ?
C18 H18A 0.9700 . ?
C18 H18B 0.9700 . ?
C19 C20 1.538(8) . ?
C19 H19 0.9800 . ?
C20 C21 1.484(8) . ?
C20 C22 1.522(8) . ?
C20 H20 0.9800 . ?
C21 H21A 0.9600 . ?
C21 H21B 0.9600 . ?
C21 H21C 0.9600 . ?
C22 H22A 0.9600 . ?
C22 H22B 0.9600 . ?
C22 H22C 0.9600 . ?

loop_
 _geom_angle_atom_site_label_1
 _geom_angle_atom_site_label_2
 _geom_angle_atom_site_label_3
 _geom_angle
 _geom_angle_site_symmetry_1
 _geom_angle_site_symmetry_3
 _geom_angle_publ_flag
N2 Zn1 N1 80.20(14) . . ?

N2 Zn1 Cl1 117.25(11) . . ?
N1 Zn1 Cl1 110.31(11) . . ?
N2 Zn1 Cl2 112.68(11) . . ?
N1 Zn1 Cl2 111.18(11) . . ?
Cl1 Zn1 Cl2 118.66(6) . . ?
N4 Zn2 N3 80.09(16) . . ?
N4 Zn2 Cl4 119.68(11) . . ?
N3 Zn2 Cl4 106.42(12) . . ?
N4 Zn2 Cl3 109.81(13) . . ?
N3 Zn2 Cl3 116.59(11) . . ?
Cl4 Zn2 Cl3 118.35(6) . . ?
C1 N1 C5 117.5(4) . . ?
C1 N1 Zn1 130.8(3) . . ?
C5 N1 Zn1 111.7(3) . . ?
C6 N2 C8 107.3(3) . . ?
C6 N2 Zn1 111.2(3) . . ?
C8 N2 Zn1 141.4(3) . . ?
C12 N3 C16 118.4(5) . . ?
C12 N3 Zn2 129.3(3) . . ?
C16 N3 Zn2 112.1(3) . . ?
C17 N4 C19 108.8(4) . . ?
C17 N4 Zn2 111.5(3) . . ?
C19 N4 Zn2 138.7(3) . . ?
C6 O1 C7 105.8(4) . . ?
C17 O2 C18 105.1(4) . . ?
N1 C1 C2 123.3(5) . . ?
N1 C1 H1 118.4 . . ?
C2 C1 H1 118.4 . . ?
C1 C2 C3 118.7(5) . . ?
C1 C2 H2 120.7 . . ?
C3 C2 H2 120.7 . . ?
C4 C3 C2 119.1(5) . . ?
C4 C3 H3 120.5 . . ?
C2 C3 H3 120.5 . . ?
C5 C4 C3 118.5(4) . . ?
C5 C4 H4 120.8 . . ?
C3 C4 H4 120.8 . . ?
N1 C5 C4 123.0(4) . . ?
N1 C5 C6 112.5(4) . . ?
C4 C5 C6 124.2(4) . . ?
N2 C6 O1 119.0(4) . . ?
N2 C6 C5 122.7(3) . . ?
O1 C6 C5 118.2(4) . . ?
O1 C7 C8 104.1(3) . . ?

O1 C7 H7A 110.9 . . ?
C8 C7 H7A 110.9 . . ?
O1 C7 H7B 110.9 . . ?
C8 C7 H7B 110.9 . . ?
H7A C7 H7B 109.0 . . ?
N2 C8 C9 113.7(4) . . ?
N2 C8 C7 102.8(4) . . ?
C9 C8 C7 116.0(4) . . ?
N2 C8 H8 108.0 . . ?
C9 C8 H8 108.0 . . ?
C7 C8 H8 108.0 . . ?
C10 C9 C8 115.8(5) . . ?
C10 C9 C11 111.6(4) . . ?
C8 C9 C11 109.7(4) . . ?
C10 C9 H9 106.4 . . ?
C8 C9 H9 106.4 . . ?
C11 C9 H9 106.4 . . ?
C9 C10 H10A 109.5 . . ?
C9 C10 H10B 109.5 . . ?
H10A C10 H10B 109.5 . . ?
C9 C10 H10C 109.5 . . ?
H10A C10 H10C 109.5 . . ?
H10B C10 H10C 109.5 . . ?
C9 C11 H11A 109.5 . . ?
C9 C11 H11B 109.5 . . ?
H11A C11 H11B 109.5 . . ?
C9 C11 H11C 109.5 . . ?
H11A C11 H11C 109.5 . . ?
H11B C11 H11C 109.5 . . ?
N3 C12 C13 120.8(5) . . ?
N3 C12 H12 119.6 . . ?
C13 C12 H12 119.6 . . ?
C14 C13 C12 120.0(6) . . ?
C14 C13 H13 120.0 . . ?
C12 C13 H13 120.0 . . ?
C13 C14 C15 119.8(6) . . ?
C13 C14 H14 120.1 . . ?
C15 C14 H14 120.1 . . ?
C16 C15 C14 117.2(5) . . ?
C16 C15 H15 121.4 . . ?
C14 C15 H15 121.4 . . ?
N3 C16 C15 123.8(5) . . ?
N3 C16 C17 112.6(4) . . ?
C15 C16 C17 123.6(4) . . ?

N4 C17 O2 118.2(5) . . ?
N4 C17 C16 123.1(4) . . ?
O2 C17 C16 118.7(4) . . ?
O2 C18 C19 104.6(3) . . ?
O2 C18 H18A 110.8 . . ?
C19 C18 H18A 110.8 . . ?
O2 C18 H18B 110.8 . . ?
C19 C18 H18B 110.8 . . ?
H18A C18 H18B 108.9 . . ?
N4 C19 C20 112.0(4) . . ?
N4 C19 C18 101.7(4) . . ?
C20 C19 C18 112.7(4) . . ?
N4 C19 H19 110.1 . . ?
C20 C19 H19 110.1 . . ?
C18 C19 H19 110.1 . . ?
C21 C20 C22 108.0(6) . . ?
C21 C20 C19 109.5(5) . . ?
C22 C20 C19 110.2(5) . . ?
C21 C20 H20 109.7 . . ?
C22 C20 H20 109.7 . . ?
C19 C20 H20 109.7 . . ?
C20 C21 H21A 109.5 . . ?
C20 C21 H21B 109.5 . . ?
H21A C21 H21B 109.5 . . ?
C20 C21 H21C 109.5 . . ?
H21A C21 H21C 109.5 . . ?
H21B C21 H21C 109.5 . . ?
C20 C22 H22A 109.5 . . ?
C20 C22 H22B 109.5 . . ?
H22A C22 H22B 109.5 . . ?
C20 C22 H22C 109.5 . . ?
H22A C22 H22C 109.5 . . ?
H22B C22 H22C 109.5 . . ?

loop_
 _geom_torsion_atom_site_label_1
 _geom_torsion_atom_site_label_2
 _geom_torsion_atom_site_label_3
 _geom_torsion_atom_site_label_4
 _geom_torsion
 _geom_torsion_site_symmetry_1
 _geom_torsion_site_symmetry_2
 _geom_torsion_site_symmetry_3
 _geom_torsion_site_symmetry_4

_geom_torsion_publ_flag
N2 Zn1 N1 C1 -168.5(4) . . . . ?
Cl1 Zn1 N1 C1 -53.0(4) . . . . ?
Cl2 Zn1 N1 C1 80.8(4) . . . . ?
N2 Zn1 N1 C5 8.8(3) . . . . ?
Cl1 Zn1 N1 C5 124.4(3) . . . . ?
Cl2 Zn1 N1 C5 -101.8(3) . . . . ?
N1 Zn1 N2 C6 -11.9(3) . . . . ?
Cl1 Zn1 N2 C6 -119.8(3) . . . . ?
Cl2 Zn1 N2 C6 97.1(3) . . . . ?
N1 Zn1 N2 C8 172.8(5) . . . . ?
Cl1 Zn1 N2 C8 64.9(5) . . . . ?
Cl2 Zn1 N2 C8 -78.2(5) . . . . ?
N4 Zn2 N3 C12 172.6(4) . . . . ?
Cl4 Zn2 N3 C12 54.4(4) . . . . ?
Cl3 Zn2 N3 C12 -80.1(4) . . . . ?
N4 Zn2 N3 C16 -2.6(3) . . . . ?
Cl4 Zn2 N3 C16 -120.8(3) . . . . ?
Cl3 Zn2 N3 C16 104.7(3) . . . . ?
N3 Zn2 N4 C17 5.8(3) . . . . ?
Cl4 Zn2 N4 C17 109.2(3) . . . . ?
Cl3 Zn2 N4 C17 -109.0(3) . . . . ?
N3 Zn2 N4 C19 172.7(5) . . . . ?
Cl4 Zn2 N4 C19 -84.0(5) . . . . ?
Cl3 Zn2 N4 C19 57.8(5) . . . . ?
C5 N1 C1 C2 -0.8(7) . . . . ?
Zn1 N1 C1 C2 176.5(4) . . . . ?
N1 C1 C2 C3 1.2(8) . . . . ?
C1 C2 C3 C4 -0.8(8) . . . . ?
C2 C3 C4 C5 0.0(8) . . . . ?
C1 N1 C5 C4 -0.1(6) . . . . ?
Zn1 N1 C5 C4 -177.9(3) . . . . ?
C1 N1 C5 C6 173.2(4) . . . . ?
Zn1 N1 C5 C6 -4.5(4) . . . . ?
C3 C4 C5 N1 0.5(7) . . . . ?
C3 C4 C5 C6 -172.1(4) . . . . ?
C8 N2 C6 O1 7.1(5) . . . . ?
Zn1 N2 C6 O1 -169.9(3) . . . . ?
C8 N2 C6 C5 -169.0(4) . . . . ?
Zn1 N2 C6 C5 14.1(5) . . . . ?
C7 O1 C6 N2 -0.4(6) . . . . ?
C7 O1 C6 C5 175.9(4) . . . . ?
N1 C5 C6 N2 -6.5(6) . . . . ?
C4 C5 C6 N2 166.7(4) . . . . ?

N1 C5 C6 O1 177.4(4) . . . . ?
C4 C5 C6 O1 -9.4(7) . . . . ?
C6 O1 C7 C8 -6.2(5) . . . . ?
C6 N2 C8 C9 -136.3(5) . . . . ?
Zn1 N2 C8 C9 39.1(7) . . . . ?
C6 N2 C8 C7 -10.1(5) . . . . ?
Zn1 N2 C8 C7 165.3(4) . . . . ?
O1 C7 C8 N2 9.6(5) . . . . ?
O1 C7 C8 C9 134.3(5) . . . . ?
N2 C8 C9 C10 60.6(6) . . . . ?
C7 C8 C9 C10 -58.2(6) . . . . ?
N2 C8 C9 C11 -172.0(4) . . . . ?
C7 C8 C9 C11 69.1(6) . . . . ?
C16 N3 C12 C13 0.0(7) . . . . ?
Zn2 N3 C12 C13 -174.9(4) . . . . ?
N3 C12 C13 C14 0.0(8) . . . . ?
C12 C13 C14 C15 -0.5(9) . . . . ?
C13 C14 C15 C16 0.9(8) . . . . ?
C12 N3 C16 C15 0.4(7) . . . . ?
Zn2 N3 C16 C15 176.2(4) . . . . ?
C12 N3 C16 C17 -176.4(4) . . . . ?
Zn2 N3 C16 C17 -0.7(5) . . . . ?
C14 C15 C16 N3 -0.9(8) . . . . ?
C14 C15 C16 C17 175.7(5) . . . . ?
C19 N4 C17 O2 -0.4(6) . . . . ?
Zn2 N4 C17 O2 170.4(3) . . . . ?
C19 N4 C17 C16 -179.5(4) . . . . ?
Zn2 N4 C17 C16 -8.6(6) . . . . ?
C18 O2 C17 N4 8.6(6) . . . . ?
C18 O2 C17 C16 -172.2(4) . . . . ?
N3 C16 C17 N4 6.5(7) . . . . ?
C15 C16 C17 N4 -170.4(5) . . . . ?
N3 C16 C17 O2 -172.6(4) . . . . ?
C15 C16 C17 O2 10.6(7) . . . . ?
C17 O2 C18 C19 -12.3(5) . . . . ?
C17 N4 C19 C20 -128.0(5) . . . . ?
Zn2 N4 C19 C20 65.0(7) . . . . ?
C17 N4 C19 C18 -7.4(6) . . . . ?
Zn2 N4 C19 C18 -174.5(4) . . . . ?
O2 C18 C19 N4 11.8(5) . . . . ?
O2 C18 C19 C20 131.9(5) . . . . ?
N4 C19 C20 C21 177.4(6) . . . . ?
C18 C19 C20 C21 63.4(7) . . . . ?
N4 C19 C20 C22 -63.9(7) . . . . ?

```
C18 C19 C20 C22 -177.8(6) . . . . ?

_diffrn_measured_fraction_theta_max    0.999
_diffrn_reflns_theta_full              25.97
_diffrn_measured_fraction_theta_full   0.999
_refine_diff_density_max    0.621
_refine_diff_density_min   -0.340
_refine_diff_density_rms    0.065

data_compound_9

_audit_creation_method            SHELXL-97
_chemical_name_systematic
;
 ?
;
_chemical_name_common             ?
_chemical_melting_point           ?
_chemical_formula_moiety          ?
_chemical_formula_sum
 'C24 H20 Cl2 N2 O2 Zn'
_chemical_formula_weight          504.69

loop_
 _atom_type_symbol
 _atom_type_description
 _atom_type_scat_dispersion_real
 _atom_type_scat_dispersion_imag
 _atom_type_scat_source
 'C'  'C'   0.0033   0.0016
 'International Tables Vol C Tables 4.2.6.8 and 6.1.1.4'
 'H'  'H'   0.0000   0.0000
 'International Tables Vol C Tables 4.2.6.8 and 6.1.1.4'
 'N'  'N'   0.0061   0.0033
 'International Tables Vol C Tables 4.2.6.8 and 6.1.1.4'
 'O'  'O'   0.0106   0.0060
 'International Tables Vol C Tables 4.2.6.8 and 6.1.1.4'
 'Cl'  'Cl'   0.1484   0.1585
 'International Tables Vol C Tables 4.2.6.8 and 6.1.1.4'
 'Zn'  'Zn'   0.2839   1.4301
 'International Tables Vol C Tables 4.2.6.8 and 6.1.1.4'

_symmetry_cell_setting            Monoclinic
_symmetry_space_group_name_H-M    P2(1)
```

```
loop_
 _symmetry_equiv_pos_as_xyz
 'x, y, z'
 '-x, y+1/2, -z'

_cell_length_a                    10.4326(8)
_cell_length_b                    7.5507(6)
_cell_length_c                    14.5952(13)
_cell_angle_alpha                 90.00
_cell_angle_beta                  100.491(2)
_cell_angle_gamma                 90.00
_cell_volume                      1130.49(16)
_cell_formula_units_Z             2
_cell_measurement_temperature     293(2)
_cell_measurement_reflns_used     1411
_cell_measurement_theta_min       5.285
_cell_measurement_theta_max       50.258

_exptl_crystal_description        prismatic
_exptl_crystal_colour             colorless
_exptl_crystal_size_max           0.112
_exptl_crystal_size_mid           0.087
_exptl_crystal_size_min           0.069
_exptl_crystal_density_meas       ?
_exptl_crystal_density_diffrn     1.483
_exptl_crystal_density_method     'not measured'
_exptl_crystal_F_000              516
_exptl_absorpt_coefficient_mu     1.346
_exptl_absorpt_correction_type    empirical
_exptl_absorpt_correction_T_min   0.43135
_exptl_absorpt_correction_T_max   1.00000
_exptl_absorpt_process_details    sadabs

_exptl_special_details
;
 ?
;

_diffrn_ambient_temperature       293(2)
_diffrn_radiation_wavelength      0.71073
_diffrn_radiation_type            MoK\a
_diffrn_radiation_source          'fine-focus sealed tube'
_diffrn_radiation_monochromator   graphite
```

```
_diffrn_measurement_device_type   'CCD area detector'
_diffrn_measurement_method        'phi and omega scans'
_diffrn_detector_area_resol_mean  ?
_diffrn_standards_number          ?
_diffrn_standards_interval_count  ?
_diffrn_standards_interval_time   ?
_diffrn_standards_decay_%         ?
_diffrn_reflns_number             6889
_diffrn_reflns_av_R_equivalents   0.0364
_diffrn_reflns_av_sigmaI/netI     0.0722
_diffrn_reflns_limit_h_min        -12
_diffrn_reflns_limit_h_max        9
_diffrn_reflns_limit_k_min        -9
_diffrn_reflns_limit_k_max        9
_diffrn_reflns_limit_l_min        -17
_diffrn_reflns_limit_l_max        16
_diffrn_reflns_theta_min          1.99
_diffrn_reflns_theta_max          26.00
_reflns_number_total              4365
_reflns_number_gt                 3535
_reflns_threshold_expression      >2sigma(I)

_computing_data_collection        'Bruker SMART'
_computing_cell_refinement        'Bruker SMART'
_computing_data_reduction         'Bruker SHELXTL'
_computing_structure_solution     'SHELXS-97 (Sheldrick, 1990)'
_computing_structure_refinement   'SHELXL-97 (Sheldrick, 1997)'
_computing_molecular_graphics     'Bruker SHELXTL'
_computing_publication_material   'Bruker SHELXTL'

_refine_special_details
;
 Refinement of F^2^ against ALL reflections.  The weighted R-factor wR and
 goodness of fit S are based on F^2^, conventional R-factors R are based
 on F, with F set to zero for negative F^2^. The threshold expression of
 F^2^ > 2sigma(F^2^) is used only for calculating R-factors(gt) etc. and is
 not relevant to the choice of reflections for refinement.  R-factors based
 on F^2^ are statistically about twice as large as those based on F, and R-
 factors based on ALL data will be even larger.
;

_refine_ls_structure_factor_coef  Fsqd
_refine_ls_matrix_type            full
_refine_ls_weighting_scheme       calc
```

```
_refine_ls_weighting_details
 'calc w=1/[\s^2^(Fo^2^)+(0.0391P)^2^+0.0000P] where P=(Fo^2^+2Fc^2^)/3'
_atom_sites_solution_primary      direct
_atom_sites_solution_secondary    difmap
_atom_sites_solution_hydrogens    geom
_refine_ls_hydrogen_treatment     constr
_refine_ls_extinction_method      none
_refine_ls_extinction_coef        ?
_refine_ls_abs_structure_details
 'Flack H D (1983), Acta Cryst. A39, 876-881'
_refine_ls_abs_structure_Flack    0.048(17)
_chemical_absolute_configuration  ad
_refine_ls_number_reflns          4365
_refine_ls_number_parameters      280
_refine_ls_number_restraints      1
_refine_ls_R_factor_all           0.0731
_refine_ls_R_factor_gt            0.0556
_refine_ls_wR_factor_ref          0.1070
_refine_ls_wR_factor_gt           0.1005
_refine_ls_goodness_of_fit_ref    1.067
_refine_ls_restrained_S_all       1.067
_refine_ls_shift/su_max           0.000
_refine_ls_shift/su_mean          0.000

loop_
 _atom_site_label
 _atom_site_type_symbol
 _atom_site_fract_x
 _atom_site_fract_y
 _atom_site_fract_z
 _atom_site_U_iso_or_equiv
 _atom_site_adp_type
 _atom_site_occupancy
 _atom_site_symmetry_multiplicity
 _atom_site_calc_flag
 _atom_site_refinement_flags
 _atom_site_disorder_assembly
 _atom_site_disorder_group
Zn1 Zn 0.23132(5) 0.27482(8) 0.23615(4) 0.04248(16) Uani 1 1 d . . .
Cl1 Cl 0.19130(14) 0.2846(3) 0.37862(8) 0.0633(4) Uani 1 1 d . . .
Cl2 Cl 0.27362(15) 0.5230(2) 0.16634(11) 0.0651(4) Uani 1 1 d . . .
N1 N 0.0903(4) 0.1377(5) 0.1493(3) 0.0417(10) Uani 1 1 d . . .
N2 N 0.3637(4) 0.0792(6) 0.2332(3) 0.0438(11) Uani 1 1 d . . .
O1 O -0.0129(4) -0.0164(6) 0.0279(2) 0.0585(11) Uani 1 1 d . . .
```

O2 O 0.4801(4) -0.1335(5) 0.1825(3) 0.0656(12) Uani 1 1 d . . .
C1 C 0.0963(5) 0.0604(7) 0.0740(4) 0.0481(14) Uani 1 1 d . . .
C2 C -0.1160(5) 0.0443(9) 0.0744(4) 0.0574(16) Uani 1 1 d . . .
H2A H -0.1752 -0.0516 0.0817 0.069 Uiso 1 1 calc R . .
H2B H -0.1651 0.1397 0.0399 0.069 Uiso 1 1 calc R . .
C3 C -0.0434(5) 0.1095(7) 0.1696(4) 0.0467(13) Uani 1 1 d . . .
H3 H -0.0798 0.2234 0.1843 0.056 Uiso 1 1 calc R . .
C4 C -0.0446(5) -0.0166(7) 0.2485(4) 0.0421(12) Uani 1 1 d . . .
C5 C -0.1123(5) 0.0226(10) 0.3186(5) 0.0663(17) Uani 1 1 d . . .
H5 H -0.1557 0.1303 0.3178 0.080 Uiso 1 1 calc R . .
C6 C -0.1166(7) -0.0943(13) 0.3892(5) 0.087(2) Uani 1 1 d . . .
H6 H -0.1629 -0.0654 0.4360 0.104 Uiso 1 1 calc R . .
C7 C -0.0539(6) -0.2528(13) 0.3919(5) 0.080(2) Uani 1 1 d . . .
H7 H -0.0572 -0.3322 0.4401 0.096 Uiso 1 1 calc R . .
C8 C 0.0145(6) -0.2943(9) 0.3226(5) 0.0677(18) Uani 1 1 d . . .
H8 H 0.0570 -0.4027 0.3237 0.081 Uiso 1 1 calc R . .
C9 C 0.0201(5) -0.1771(7) 0.2522(4) 0.0565(17) Uani 1 1 d . . .
H9 H 0.0679 -0.2054 0.2063 0.068 Uiso 1 1 calc R . .
C10 C 0.3904(5) -0.0070(7) 0.1646(4) 0.0456(13) Uani 1 1 d . . .
C11 C 0.5113(7) -0.1493(9) 0.2829(6) 0.080(2) Uani 1 1 d . . .
H11A H 0.6048 -0.1439 0.3046 0.097 Uiso 1 1 calc R . .
H11B H 0.4788 -0.2602 0.3033 0.097 Uiso 1 1 calc R . .
C12 C 0.4437(5) 0.0078(7) 0.3195(4) 0.0512(14) Uani 1 1 d . . .
H12 H 0.3853 -0.0369 0.3597 0.061 Uiso 1 1 calc R . .
C13 C 0.5367(5) 0.1399(7) 0.3741(4) 0.0437(13) Uani 1 1 d . . .
C14 C 0.5744(5) 0.2926(10) 0.3340(4) 0.0555(14) Uani 1 1 d . . .
H14 H 0.5386 0.3209 0.2727 0.067 Uiso 1 1 calc R . .
C15 C 0.6659(6) 0.4031(8) 0.3859(5) 0.0643(18) Uani 1 1 d . . .
H15 H 0.6914 0.5053 0.3586 0.077 Uiso 1 1 calc R . .
C16 C 0.7194(6) 0.3653(9) 0.4763(5) 0.0651(19) Uani 1 1 d . . .
H16 H 0.7802 0.4408 0.5109 0.078 Uiso 1 1 calc R . .
C17 C 0.6812(7) 0.2132(10) 0.5144(5) 0.069(2) Uani 1 1 d . . .
H17 H 0.7180 0.1845 0.5755 0.083 Uiso 1 1 calc R . .
C18 C 0.5890(5) 0.1006(8) 0.4647(4) 0.0527(14) Uani 1 1 d . . .
H18 H 0.5629 -0.0006 0.4927 0.063 Uiso 1 1 calc R . .
C19 C 0.2086(6) 0.0483(7) 0.0244(3) 0.0475(14) Uani 1 1 d . . .
C20 C 0.1747(7) 0.0632(8) -0.0731(4) 0.0660(19) Uani 1 1 d . . .
H20 H 0.0876 0.0780 -0.1004 0.079 Uiso 1 1 calc R . .
C21 C 0.2668(9) 0.0565(10) -0.1283(5) 0.089(2) Uani 1 1 d . . .
H21 H 0.2427 0.0699 -0.1925 0.107 Uiso 1 1 calc R . .
C22 C 0.3951(8) 0.0299(10) -0.0891(6) 0.087(2) Uani 1 1 d . . .
H22 H 0.4580 0.0255 -0.1268 0.105 Uiso 1 1 calc R . .
C23 C 0.4309(7) 0.0099(9) 0.0057(5) 0.0727(19) Uani 1 1 d . . .
H23 H 0.5178 -0.0118 0.0312 0.087 Uiso 1 1 calc R . .

```
C24 C 0.3389(6) 0.0215(7) 0.0649(4) 0.0514(14) Uani 1 1 d . . .

loop_
 _atom_site_aniso_label
 _atom_site_aniso_U_11
 _atom_site_aniso_U_22
 _atom_site_aniso_U_33
 _atom_site_aniso_U_23
 _atom_site_aniso_U_13
 _atom_site_aniso_U_12
Zn1 0.0470(3) 0.0391(3) 0.0397(3) -0.0071(4) 0.0037(2) -0.0025(4)
Cl1 0.0914(10) 0.0579(8) 0.0422(7) -0.0079(10) 0.0164(6) 0.0064(11)
Cl2 0.0726(11) 0.0519(8) 0.0683(10) 0.0096(8) 0.0066(8) -0.0141(8)
N1 0.043(3) 0.040(2) 0.040(3) -0.003(2) 0.002(2) -0.0082(19)
N2 0.042(3) 0.041(2) 0.047(3) -0.004(2) 0.006(2) 0.0023(19)
O1 0.055(3) 0.071(3) 0.043(2) -0.011(2) -0.0083(19) -0.014(2)
O2 0.063(3) 0.042(2) 0.092(4) -0.010(2) 0.015(2) 0.004(2)
C1 0.058(4) 0.042(3) 0.040(3) -0.001(2) -0.004(3) -0.008(3)
C2 0.050(4) 0.057(4) 0.058(4) 0.008(3) -0.010(3) -0.003(3)
C3 0.038(3) 0.039(3) 0.060(4) 0.000(3) 0.001(3) 0.006(2)
C4 0.027(3) 0.047(3) 0.050(3) -0.001(3) 0.003(2) 0.003(2)
C5 0.047(4) 0.082(5) 0.075(4) -0.005(4) 0.024(3) 0.012(3)
C6 0.062(5) 0.139(8) 0.068(5) 0.008(5) 0.032(4) -0.008(5)
C7 0.063(5) 0.107(8) 0.067(4) 0.035(5) 0.003(3) -0.026(5)
C8 0.065(4) 0.061(4) 0.072(5) 0.015(3) -0.002(4) 0.004(3)
C9 0.048(4) 0.062(5) 0.060(4) 0.005(3) 0.011(3) 0.004(3)
C10 0.037(3) 0.037(3) 0.065(4) -0.008(3) 0.015(3) -0.006(2)
C11 0.071(5) 0.048(4) 0.107(6) -0.010(4) -0.024(4) 0.002(3)
C12 0.048(4) 0.042(3) 0.060(4) 0.009(3) 0.000(3) -0.004(3)
C13 0.035(3) 0.045(3) 0.050(3) 0.001(3) 0.004(2) 0.011(2)
C14 0.047(3) 0.059(4) 0.057(3) 0.006(4) 0.000(2) -0.003(4)
C15 0.066(5) 0.041(3) 0.083(5) -0.003(3) 0.008(4) -0.006(3)
C16 0.056(4) 0.056(4) 0.077(5) -0.017(4) -0.005(4) -0.005(3)
C17 0.070(5) 0.073(5) 0.055(4) -0.010(3) -0.012(4) 0.019(4)
C18 0.052(4) 0.043(3) 0.060(4) 0.008(3) 0.001(3) 0.005(3)
C19 0.071(4) 0.037(3) 0.036(3) -0.006(2) 0.013(3) -0.010(3)
C20 0.099(6) 0.055(4) 0.045(4) 0.001(3) 0.015(4) -0.009(4)
C21 0.146(8) 0.076(5) 0.053(4) -0.005(4) 0.039(5) -0.014(5)
C22 0.125(7) 0.068(5) 0.086(6) -0.013(5) 0.065(5) -0.014(5)
C23 0.086(5) 0.061(4) 0.080(5) -0.018(4) 0.038(4) -0.014(4)
C24 0.065(4) 0.039(3) 0.056(4) -0.013(3) 0.025(3) -0.007(3)

_geom_special_details
;
```

```
 All esds (except the esd in the dihedral angle between two l.s. planes)
 are estimated using the full covariance matrix.  The cell esds are taken
 into account individually in the estimation of esds in distances, angles
 and torsion angles; correlations between esds in cell parameters are only
 used when they are defined by crystal symmetry.  An approximate (isotropic)
 treatment of cell esds is used for estimating esds involving l.s. planes.
;

loop_
 _geom_bond_atom_site_label_1
 _geom_bond_atom_site_label_2
 _geom_bond_distance
 _geom_bond_site_symmetry_2
 _geom_bond_publ_flag
Zn1 N2 2.028(4) . ?
Zn1 N1 2.041(4) . ?
Zn1 Cl1 2.1954(13) . ?
Zn1 Cl2 2.2154(16) . ?
N1 C1 1.256(6) . ?
N1 C3 1.492(6) . ?
N2 C10 1.267(6) . ?
N2 C12 1.480(7) . ?
O1 C1 1.345(6) . ?
O1 C2 1.447(7) . ?
O2 C10 1.330(6) . ?
O2 C11 1.448(8) . ?
C1 C19 1.489(7) . ?
C2 C3 1.537(7) . ?
C2 H2A 0.9700 . ?
C2 H2B 0.9700 . ?
C3 C4 1.497(7) . ?
C3 H3 0.9800 . ?
C4 C5 1.378(7) . ?
C4 C9 1.383(7) . ?
C5 C6 1.364(10) . ?
C5 H5 0.9300 . ?
C6 C7 1.361(11) . ?
C6 H6 0.9300 . ?
C7 C8 1.376(9) . ?
C7 H7 0.9300 . ?
C8 C9 1.365(8) . ?
C8 H8 0.9300 . ?
C9 H9 0.9300 . ?
C10 C24 1.473(8) . ?
```

```
C11 C12 1.525(9) . ?
C11 H11A 0.9700 . ?
C11 H11B 0.9700 . ?
C12 C13 1.514(7) . ?
C12 H12 0.9800 . ?
C13 C18 1.368(7) . ?
C13 C14 1.382(8) . ?
C14 C15 1.384(8) . ?
C14 H14 0.9300 . ?
C15 C16 1.366(9) . ?
C15 H15 0.9300 . ?
C16 C17 1.367(9) . ?
C16 H16 0.9300 . ?
C17 C18 1.385(8) . ?
C17 H17 0.9300 . ?
C18 H18 0.9300 . ?
C19 C24 1.395(7) . ?
C19 C20 1.406(7) . ?
C20 C21 1.363(9) . ?
C20 H20 0.9300 . ?
C21 C22 1.371(9) . ?
C21 H21 0.9300 . ?
C22 C23 1.374(10) . ?
C22 H22 0.9300 . ?
C23 C24 1.407(7) . ?
C23 H23 0.9300 . ?

loop_
 _geom_angle_atom_site_label_1
 _geom_angle_atom_site_label_2
 _geom_angle_atom_site_label_3
 _geom_angle
 _geom_angle_site_symmetry_1
 _geom_angle_site_symmetry_3
 _geom_angle_publ_flag
N2 Zn1 N1 91.96(17) . . ?
N2 Zn1 Cl1 106.98(13) . . ?
N1 Zn1 Cl1 111.44(12) . . ?
N2 Zn1 Cl2 114.40(13) . . ?
N1 Zn1 Cl2 108.96(12) . . ?
Cl1 Zn1 Cl2 119.69(7) . . ?
C1 N1 C3 107.7(4) . . ?
C1 N1 Zn1 129.4(4) . . ?
C3 N1 Zn1 122.9(3) . . ?
```

C10 N2 C12 108.3(5) . . ?
C10 N2 Zn1 129.7(4) . . ?
C12 N2 Zn1 121.9(3) . . ?
C1 O1 C2 105.2(4) . . ?
C10 O2 C11 106.2(5) . . ?
N1 C1 O1 117.8(5) . . ?
N1 C1 C19 128.6(5) . . ?
O1 C1 C19 113.6(5) . . ?
O1 C2 C3 103.9(4) . . ?
O1 C2 H2A 111.0 . . ?
C3 C2 H2A 111.0 . . ?
O1 C2 H2B 111.0 . . ?
C3 C2 H2B 111.0 . . ?
H2A C2 H2B 109.0 . . ?
N1 C3 C4 112.5(4) . . ?
N1 C3 C2 101.0(4) . . ?
C4 C3 C2 114.8(5) . . ?
N1 C3 H3 109.4 . . ?
C4 C3 H3 109.4 . . ?
C2 C3 H3 109.4 . . ?
C5 C4 C9 118.2(5) . . ?
C5 C4 C3 120.8(5) . . ?
C9 C4 C3 121.0(5) . . ?
C6 C5 C4 121.0(7) . . ?
C6 C5 H5 119.5 . . ?
C4 C5 H5 119.5 . . ?
C7 C6 C5 120.5(7) . . ?
C7 C6 H6 119.7 . . ?
C5 C6 H6 119.7 . . ?
C6 C7 C8 119.4(7) . . ?
C6 C7 H7 120.3 . . ?
C8 C7 H7 120.3 . . ?
C9 C8 C7 120.3(6) . . ?
C9 C8 H8 119.8 . . ?
C7 C8 H8 119.8 . . ?
C8 C9 C4 120.6(5) . . ?
C8 C9 H9 119.7 . . ?
C4 C9 H9 119.7 . . ?
N2 C10 O2 117.5(5) . . ?
N2 C10 C24 127.8(5) . . ?
O2 C10 C24 114.6(5) . . ?
O2 C11 C12 104.9(5) . . ?
O2 C11 H11A 110.8 . . ?
C12 C11 H11A 110.8 . . ?

O2 C11 H11B 110.8 . . ?
C12 C11 H11B 110.8 . . ?
H11A C11 H11B 108.8 . . ?
N2 C12 C13 114.6(4) . . ?
N2 C12 C11 102.2(5) . . ?
C13 C12 C11 113.8(5) . . ?
N2 C12 H12 108.6 . . ?
C13 C12 H12 108.6 . . ?
C11 C12 H12 108.6 . . ?
C18 C13 C14 119.8(5) . . ?
C18 C13 C12 118.1(5) . . ?
C14 C13 C12 122.1(5) . . ?
C13 C14 C15 119.5(5) . . ?
C13 C14 H14 120.3 . . ?
C15 C14 H14 120.3 . . ?
C16 C15 C14 121.5(6) . . ?
C16 C15 H15 119.2 . . ?
C14 C15 H15 119.2 . . ?
C15 C16 C17 118.0(7) . . ?
C15 C16 H16 121.0 . . ?
C17 C16 H16 121.0 . . ?
C16 C17 C18 121.9(7) . . ?
C16 C17 H17 119.0 . . ?
C18 C17 H17 119.0 . . ?
C13 C18 C17 119.3(6) . . ?
C13 C18 H18 120.4 . . ?
C17 C18 H18 120.4 . . ?
C24 C19 C20 119.1(5) . . ?
C24 C19 C1 126.5(5) . . ?
C20 C19 C1 114.3(5) . . ?
C21 C20 C19 121.4(7) . . ?
C21 C20 H20 119.3 . . ?
C19 C20 H20 119.3 . . ?
C20 C21 C22 119.9(7) . . ?
C20 C21 H21 120.1 . . ?
C22 C21 H21 120.1 . . ?
C23 C22 C21 120.1(7) . . ?
C23 C22 H22 119.9 . . ?
C21 C22 H22 119.9 . . ?
C22 C23 C24 121.4(7) . . ?
C22 C23 H23 119.3 . . ?
C24 C23 H23 119.3 . . ?
C19 C24 C23 118.0(6) . . ?
C19 C24 C10 126.3(5) . . ?

```
C23 C24 C10 115.6(6) . . ?

loop_
 _geom_torsion_atom_site_label_1
 _geom_torsion_atom_site_label_2
 _geom_torsion_atom_site_label_3
 _geom_torsion_atom_site_label_4
 _geom_torsion
 _geom_torsion_site_symmetry_1
 _geom_torsion_site_symmetry_2
 _geom_torsion_site_symmetry_3
 _geom_torsion_site_symmetry_4
 _geom_torsion_publ_flag
N2 Zn1 N1 C1 -47.5(5) . . . . ?
Cl1 Zn1 N1 C1 -156.7(4) . . . . ?
Cl2 Zn1 N1 C1 69.1(5) . . . . ?
N2 Zn1 N1 C3 130.6(4) . . . . ?
Cl1 Zn1 N1 C3 21.5(4) . . . . ?
Cl2 Zn1 N1 C3 -112.7(4) . . . . ?
N1 Zn1 N2 C10 45.8(5) . . . . ?
Cl1 Zn1 N2 C10 158.9(4) . . . . ?
Cl2 Zn1 N2 C10 -66.1(5) . . . . ?
N1 Zn1 N2 C12 -131.0(4) . . . . ?
Cl1 Zn1 N2 C12 -17.9(4) . . . . ?
Cl2 Zn1 N2 C12 117.1(4) . . . . ?
C3 N1 C1 O1 3.8(6) . . . . ?
Zn1 N1 C1 O1 -177.8(3) . . . . ?
C3 N1 C1 C19 -179.6(5) . . . . ?
Zn1 N1 C1 C19 -1.2(9) . . . . ?
C2 O1 C1 N1 10.2(7) . . . . ?
C2 O1 C1 C19 -166.8(4) . . . . ?
C1 O1 C2 C3 -18.8(6) . . . . ?
C1 N1 C3 C4 107.8(5) . . . . ?
Zn1 N1 C3 C4 -70.6(5) . . . . ?
C1 N1 C3 C2 -15.1(5) . . . . ?
Zn1 N1 C3 C2 166.4(3) . . . . ?
O1 C2 C3 N1 20.1(5) . . . . ?
O1 C2 C3 C4 -101.2(5) . . . . ?
N1 C3 C4 C5 133.4(5) . . . . ?
C2 C3 C4 C5 -111.8(6) . . . . ?
N1 C3 C4 C9 -47.9(7) . . . . ?
C2 C3 C4 C9 66.9(6) . . . . ?
C9 C4 C5 C6 -0.7(9) . . . . ?
C3 C4 C5 C6 178.1(6) . . . . ?
```

C4 C5 C6 C7 0.0(11) . . . . ?
C5 C6 C7 C8 0.1(12) . . . . ?
C6 C7 C8 C9 0.5(11) . . . . ?
C7 C8 C9 C4 -1.2(9) . . . . ?
C5 C4 C9 C8 1.3(8) . . . . ?
C3 C4 C9 C8 -177.5(5) . . . . ?
C12 N2 C10 O2 -1.0(6) . . . . ?
Zn1 N2 C10 O2 -178.1(3) . . . . ?
C12 N2 C10 C24 -177.3(5) . . . . ?
Zn1 N2 C10 C24 5.5(8) . . . . ?
C11 O2 C10 N2 7.0(6) . . . . ?
C11 O2 C10 C24 -176.2(5) . . . . ?
C10 O2 C11 C12 -9.4(6) . . . . ?
C10 N2 C12 C13 118.5(5) . . . . ?
Zn1 N2 C12 C13 -64.1(6) . . . . ?
C10 N2 C12 C11 -5.1(6) . . . . ?
Zn1 N2 C12 C11 172.3(4) . . . . ?
O2 C11 C12 N2 8.7(6) . . . . ?
O2 C11 C12 C13 -115.5(5) . . . . ?
N2 C12 C13 C18 163.5(5) . . . . ?
C11 C12 C13 C18 -79.4(7) . . . . ?
N2 C12 C13 C14 -19.6(7) . . . . ?
C11 C12 C13 C14 97.5(6) . . . . ?
C18 C13 C14 C15 0.7(9) . . . . ?
C12 C13 C14 C15 -176.1(5) . . . . ?
C13 C14 C15 C16 -0.4(10) . . . . ?
C14 C15 C16 C17 0.6(10) . . . . ?
C15 C16 C17 C18 -1.2(10) . . . . ?
C14 C13 C18 C17 -1.3(9) . . . . ?
C12 C13 C18 C17 175.6(5) . . . . ?
C16 C17 C18 C13 1.6(9) . . . . ?
N1 C1 C19 C24 40.9(9) . . . . ?
O1 C1 C19 C24 -142.4(5) . . . . ?
N1 C1 C19 C20 -140.2(6) . . . . ?
O1 C1 C19 C20 36.5(7) . . . . ?
C24 C19 C20 C21 -1.7(9) . . . . ?
C1 C19 C20 C21 179.4(6) . . . . ?
C19 C20 C21 C22 1.8(11) . . . . ?
C20 C21 C22 C23 0.1(12) . . . . ?
C21 C22 C23 C24 -2.0(11) . . . . ?
C20 C19 C24 C23 -0.3(8) . . . . ?
C1 C19 C24 C23 178.6(5) . . . . ?
C20 C19 C24 C10 -176.3(5) . . . . ?
C1 C19 C24 C10 2.5(9) . . . . ?

```
C22 C23 C24 C19 2.1(9) . . . . ?
C22 C23 C24 C10 178.6(6) . . . . ?
N2 C10 C24 C19 -46.3(8) . . . . ?
O2 C10 C24 C19 137.2(5) . . . . ?
N2 C10 C24 C23 137.6(6) . . . . ?
O2 C10 C24 C23 -38.9(7) . . . . ?

_diffrn_measured_fraction_theta_max    0.999
_diffrn_reflns_theta_full              26.00
_diffrn_measured_fraction_theta_full   0.999
_refine_diff_density_max    0.569
_refine_diff_density_min   -0.315
_refine_diff_density_rms    0.072

data_compound_10

_audit_creation_method            SHELXL-97
_chemical_name_systematic
;
 ?
;
_chemical_name_common             ?
_chemical_melting_point           ?
_chemical_formula_moiety          ?
_chemical_formula_sum
 'C23 H35 Cl2 N3 O2 Zn'
_chemical_formula_weight          521.81
_chemical_absolute_configuration  'ad'

loop_
 _atom_type_symbol
 _atom_type_description
 _atom_type_scat_dispersion_real
 _atom_type_scat_dispersion_imag
 _atom_type_scat_source
 'C'  'C'   0.0181   0.0091
 'International Tables Vol C Tables 4.2.6.8 and 6.1.1.4'
 'H'  'H'   0.0000   0.0000
 'International Tables Vol C Tables 4.2.6.8 and 6.1.1.4'
 'N'  'N'   0.0311   0.0180
 'International Tables Vol C Tables 4.2.6.8 and 6.1.1.4'
 'O'  'O'   0.0492   0.0322
 'International Tables Vol C Tables 4.2.6.8 and 6.1.1.4'
 'Cl'  'Cl'   0.3639   0.7018
```

```
 'International Tables Vol C Tables 4.2.6.8 and 6.1.1.4'
 'Zn'  'Zn'  -1.5491   0.6778
 'International Tables Vol C Tables 4.2.6.8 and 6.1.1.4'

_symmetry_cell_setting           monoclinic
_symmetry_space_group_name_H-M    'P 21'

loop_
 _symmetry_equiv_pos_as_xyz
 'x, y, z'
 '-x, y+1/2, -z'

_cell_length_a                    9.78320(10)
_cell_length_b                    11.33510(10)
_cell_length_c                    11.81900(10)
_cell_angle_alpha                 90.00
_cell_angle_beta                  96.3280(10)
_cell_angle_gamma                 90.00
_cell_volume                      1302.67(2)
_cell_formula_units_Z             2
_cell_measurement_temperature     291(2)
_cell_measurement_reflns_used     11393
_cell_measurement_theta_min       3.7597
_cell_measurement_theta_max       62.6022

_exptl_crystal_description        block
_exptl_crystal_colour             colorless
_exptl_crystal_size_max           0.40
_exptl_crystal_size_mid           0.38
_exptl_crystal_size_min           0.32
_exptl_crystal_density_meas       ?
_exptl_crystal_density_diffrn     1.330
_exptl_crystal_density_method     'not measured'
_exptl_crystal_F_000              548
_exptl_absorpt_coefficient_mu     3.376
_exptl_absorpt_correction_type    'multi-scan'
_exptl_absorpt_correction_T_min   0.3454
_exptl_absorpt_correction_T_max   0.4113
_exptl_absorpt_process_details
;
CrysAlisPro, Oxford Diffraction Ltd.,
Version 1.171.34.44 (release 25-10-2010 CrysAlis171 .NET)
(compiled Oct 25 2010,18:11:34)
Empirical absorption correction using spherical harmonics,
```

```
 implemented in SCALE3 ABSPACK scaling algorithm.
;

_exptl_special_details
;
 ?
;

_diffrn_ambient_temperature       291(2)
_diffrn_radiation_wavelength      1.54184
_diffrn_radiation_type            CuK\a
_diffrn_radiation_source          'Enhance Ultra (Cu) X-ray Source'
_diffrn_radiation_monochromator   'mirror'
_diffrn_measurement_device_type   'Gemini S Ultra, Oxford Diffraction'
_diffrn_measurement_method        '\w scans'
_diffrn_detector_area_resol_mean  15.9149
_diffrn_standards_number          ?
_diffrn_standards_interval_count  ?
_diffrn_standards_interval_time   ?
_diffrn_standards_decay_%         ?
_diffrn_reflns_number             13197
_diffrn_reflns_av_R_equivalents   0.0207
_diffrn_reflns_av_sigmaI/netI     0.0201
_diffrn_reflns_limit_h_min        -11
_diffrn_reflns_limit_h_max        11
_diffrn_reflns_limit_k_min        -13
_diffrn_reflns_limit_k_max        13
_diffrn_reflns_limit_l_min        -12
_diffrn_reflns_limit_l_max        13
_diffrn_reflns_theta_min          3.76
_diffrn_reflns_theta_max          62.69
_reflns_number_total              4158
_reflns_number_gt                 4098
_reflns_threshold_expression      >2sigma(I)

_computing_data_collection        'CrysAlisPro (Oxford Diffraction Ltd.)'
_computing_cell_refinement        'CrysAlisPro (Oxford Diffraction Ltd.)'
_computing_data_reduction         'CrysAlisPro (Oxford Diffraction Ltd.)'
_computing_structure_solution     'SHELXS-97 (Sheldrick, 1997)'
_computing_structure_refinement   'SHELXL-97 (Sheldrick, 1997)'
_computing_molecular_graphics     'Ortep-3 (L. J. Farrugia, 2001)'
_computing_publication_material   SHELXL-97

_refine_special_details
```

```
;
 Refinement of F^2^ against ALL reflections.  The weighted R-factor wR and
 goodness of fit S are based on F^2^, conventional R-factors R are based
 on F, with F set to zero for negative F^2^. The threshold expression of
 F^2^ > 2sigma(F^2^) is used only for calculating R-factors(gt) etc. and is
 not relevant to the choice of reflections for refinement.  R-factors based
 on F^2^ are statistically about twice as large as those based on F, and R-
 factors based on ALL data will be even larger.
;

_refine_ls_structure_factor_coef  Fsqd
_refine_ls_matrix_type            full
_refine_ls_weighting_scheme       calc
_refine_ls_weighting_details
 'calc w=1/[\s^2^(Fo^2^)+(0.0473P)^2^+0.0709P] where P=(Fo^2^+2Fc^2^)/3'
_atom_sites_solution_primary      direct
_atom_sites_solution_secondary    difmap
_atom_sites_solution_hydrogens    geom
_refine_ls_hydrogen_treatment     mixed
_refine_ls_extinction_method      none
_refine_ls_extinction_coef        ?
_refine_ls_abs_structure_details
 'Flack H D (1983), Acta Cryst. A39, 876-881'
_refine_ls_abs_structure_Flack    -0.001(10)
_refine_ls_number_reflns          4158
_refine_ls_number_parameters      290
_refine_ls_number_restraints      1
_refine_ls_R_factor_all           0.0246
_refine_ls_R_factor_gt            0.0243
_refine_ls_wR_factor_ref          0.0645
_refine_ls_wR_factor_gt           0.0643
_refine_ls_goodness_of_fit_ref    1.041
_refine_ls_restrained_S_all       1.041
_refine_ls_shift/su_max           0.000
_refine_ls_shift/su_mean          0.000

loop_
 _atom_site_label
 _atom_site_type_symbol
 _atom_site_fract_x
 _atom_site_fract_y
 _atom_site_fract_z
 _atom_site_U_iso_or_equiv
 _atom_site_adp_type
```

_atom_site_occupancy
_atom_site_symmetry_multiplicity
_atom_site_calc_flag
_atom_site_refinement_flags
_atom_site_disorder_assembly
_atom_site_disorder_group
Zn1 Zn 0.26463(2) 0.617529(17) 0.178916(18) 0.03991(9) Uani 1 1 d . . .
Cl1 Cl 0.35481(6) 0.73462(5) 0.05532(5) 0.05706(14) Uani 1 1 d . . .
Cl2 Cl 0.15601(6) 0.70975(6) 0.31006(5) 0.06113(15) Uani 1 1 d . . .
N1 N 0.12737(17) 0.49443(14) 0.10271(14) 0.0426(4) Uani 1 1 d . . .
N3 N 0.47257(16) 0.31361(15) 0.28065(14) 0.0404(4) Uani 1 1 d . . .
O1 O -0.00194(17) 0.33182(16) 0.09224(17) 0.0659(4) Uani 1 1 d . . .
O2 O 0.5991(2) 0.27265(18) 0.49539(16) 0.0816(6) Uani 1 1 d . . .
N2 N 0.40504(16) 0.49712(15) 0.23453(15) 0.0441(4) Uani 1 1 d . . .
C19 C 0.4733(2) 0.19430(18) 0.32895(17) 0.0441(4) Uani 1 1 d . . .
H19 H 0.3914 0.1869 0.3692 0.053 Uiso 1 1 calc R . .
C14 C 0.5437(2) 0.48723(18) 0.19498(18) 0.0444(4) Uani 1 1 d . . .
H14 H 0.5358 0.4986 0.1123 0.053 Uiso 1 1 calc R . .
C1 C 0.1186(2) 0.37245(19) 0.2741(2) 0.0488(5) Uani 1 1 d . . .
C10 C 0.1616(3) 0.4983(2) -0.1041(2) 0.0649(7) Uani 1 1 d . . .
H10 H 0.2154 0.5709 -0.0908 0.078 Uiso 1 1 calc R . .
C18 C 0.5868(2) 0.35932(19) 0.22294(19) 0.0477(5) Uani 1 1 d . . .
H18A H 0.5970 0.3150 0.1542 0.057 Uiso 1 1 calc R . .
H18B H 0.6726 0.3564 0.2725 0.057 Uiso 1 1 calc R . .
C3 C 0.2618(3) 0.3587(2) 0.45321(19) 0.0564(6) Uani 1 1 d . . .
H3 H 0.3480 0.3626 0.4949 0.068 Uiso 1 1 calc R . .
C15 C 0.6408(2) 0.5807(2) 0.2525(3) 0.0646(6) Uani 1 1 d . . .
H15 H 0.5982 0.6581 0.2376 0.078 Uiso 1 1 calc R . .
C20 C 0.4639(2) 0.0984(2) 0.23611(19) 0.0552(5) Uani 1 1 d . . .
H20 H 0.5500 0.0995 0.2009 0.066 Uiso 1 1 calc R . .
C9 C 0.0585(2) 0.4954(2) -0.01649(18) 0.0522(5) Uani 1 1 d . . .
H9 H -0.0012 0.5649 -0.0270 0.063 Uiso 1 1 calc R . .
C2 C 0.24857(19) 0.37779(19) 0.33569(17) 0.0428(4) Uani 1 1 d . . .
C13 C 0.37719(18) 0.39754(17) 0.28161(15) 0.0378(4) Uani 1 1 d . . .
C7 C 0.08718(19) 0.40318(18) 0.15241(18) 0.0461(5) Uani 1 1 d . . .
C4 C 0.1473(3) 0.3340(3) 0.5077(2) 0.0756(8) Uani 1 1 d . . .
H4 H 0.1564 0.3251 0.5864 0.091 Uiso 1 1 calc R . .
C16 C 0.7765(2) 0.5787(3) 0.1984(3) 0.0784(9) Uani 1 1 d . . .
H16A H 0.8169 0.5016 0.2073 0.118 Uiso 1 1 calc R . .
H16C H 0.8385 0.6359 0.2353 0.118 Uiso 1 1 calc R . .
H16B H 0.7588 0.5972 0.1189 0.118 Uiso 1 1 calc R . .
C6 C 0.0060(3) 0.3403(3) 0.3315(3) 0.0731(8) Uani 1 1 d . . .
H6 H -0.0799 0.3308 0.2903 0.088 Uiso 1 1 calc R . .
C23 C 0.5980(3) 0.1796(2) 0.4164(2) 0.0593(6) Uani 1 1 d . . .

H23A H 0.5934 0.1045 0.4552 0.071 Uiso 1 1 calc R . .
H23B H 0.6815 0.1811 0.3792 0.071 Uiso 1 1 calc R . .
C21 C 0.3467(3) 0.1219(3) 0.1433(2) 0.0876(8) Uani 1 1 d . . .
H21A H 0.3386 0.0569 0.0909 0.131 Uiso 1 1 calc R . .
H21C H 0.3651 0.1929 0.1035 0.131 Uiso 1 1 calc R . .
H21B H 0.2623 0.1306 0.1770 0.131 Uiso 1 1 calc R . .
C17 C 0.6642(4) 0.5631(4) 0.3814(3) 0.1005(11) Uani 1 1 d . . .
H17A H 0.5777 0.5673 0.4124 0.151 Uiso 1 1 calc R . .
H17C H 0.7243 0.6238 0.4147 0.151 Uiso 1 1 calc R . .
H17B H 0.7053 0.4873 0.3980 0.151 Uiso 1 1 calc R . .
C8 C -0.0314(3) 0.3842(3) -0.0193(2) 0.0736(8) Uani 1 1 d . . .
H8B H -0.1281 0.4045 -0.0340 0.088 Uiso 1 1 calc R . .
H8A H -0.0081 0.3302 -0.0779 0.088 Uiso 1 1 calc R . .
C12 C 0.0830(5) 0.5078(4) -0.2235(2) 0.1028(12) Uani 1 1 d . . .
H12B H 0.1474 0.5132 -0.2790 0.154 Uiso 1 1 calc R . .
H12C H 0.0260 0.5770 -0.2277 0.154 Uiso 1 1 calc R . .
H12A H 0.0265 0.4392 -0.2385 0.154 Uiso 1 1 calc R . .
C5 C 0.0200(3) 0.3225(3) 0.4468(3) 0.0868(10) Uani 1 1 d . . .
H5 H -0.0561 0.3028 0.4836 0.104 Uiso 1 1 calc R . .
C22 C 0.4498(5) -0.0224(3) 0.2885(3) 0.0906(10) Uani 1 1 d . . .
H22B H 0.3685 -0.0244 0.3271 0.136 Uiso 1 1 calc R . .
H22C H 0.5290 -0.0382 0.3418 0.136 Uiso 1 1 calc R . .
H22A H 0.4430 -0.0812 0.2296 0.136 Uiso 1 1 calc R . .
C11 C 0.2612(4) 0.3974(4) -0.0920(4) 0.1008(11) Uani 1 1 d . . .
H11B H 0.3141 0.4009 -0.0187 0.151 Uiso 1 1 calc R . .
H11C H 0.3218 0.4023 -0.1505 0.151 Uiso 1 1 calc R . .
H11A H 0.2116 0.3242 -0.0992 0.151 Uiso 1 1 calc R . .
H2 H 0.680(4) 0.256(4) 0.554(4) 0.116(13) Uiso 1 1 d . . .

loop_
 _atom_site_aniso_label
 _atom_site_aniso_U_11
 _atom_site_aniso_U_22
 _atom_site_aniso_U_33
 _atom_site_aniso_U_23
 _atom_site_aniso_U_13
 _atom_site_aniso_U_12
Zn1 0.03742(13) 0.03598(13) 0.04652(14) 0.00116(11) 0.00557(9) 0.00244(10)
Cl1 0.0618(3) 0.0502(3) 0.0607(3) 0.0098(2) 0.0141(2) -0.0068(2)
Cl2 0.0569(3) 0.0723(4) 0.0546(3) -0.0170(3) 0.0085(2) 0.0108(3)
N1 0.0385(8) 0.0436(9) 0.0458(8) 0.0010(7) 0.0052(7) -0.0012(7)
N3 0.0379(8) 0.0383(8) 0.0460(9) 0.0026(7) 0.0085(7) 0.0048(7)
O1 0.0541(9) 0.0586(10) 0.0827(11) 0.0065(9) -0.0031(8) -0.0197(8)
O2 0.1002(15) 0.0720(13) 0.0650(11) -0.0150(9) -0.0259(11) 0.0198(11)

N2 0.0328(8) 0.0422(9) 0.0581(10) 0.0070(8) 0.0091(7) 0.0029(7)
C19 0.0466(10) 0.0399(10) 0.0464(10) 0.0023(9) 0.0070(8) 0.0057(8)
C14 0.0329(9) 0.0477(11) 0.0531(11) 0.0024(9) 0.0078(8) 0.0026(8)
C1 0.0394(10) 0.0475(11) 0.0616(12) 0.0140(10) 0.0148(9) 0.0066(8)
C10 0.0878(18) 0.0579(14) 0.0504(12) -0.0073(11) 0.0136(12) -0.0219(12)
C18 0.0408(10) 0.0460(11) 0.0585(12) -0.0009(9) 0.0148(9) 0.0045(8)
C3 0.0693(14) 0.0527(13) 0.0495(12) 0.0056(10) 0.0161(11) 0.0166(11)
C15 0.0428(11) 0.0445(12) 0.1066(19) -0.0007(12) 0.0093(12) -0.0038(9)
C20 0.0654(12) 0.0456(13) 0.0551(11) -0.0069(10) 0.0084(10) 0.0012(9)
C9 0.0524(12) 0.0517(12) 0.0506(11) -0.0025(9) -0.0033(9) -0.0037(9)
C2 0.0421(10) 0.0380(10) 0.0501(10) 0.0042(8) 0.0129(8) 0.0080(7)
C13 0.0346(9) 0.0411(10) 0.0372(9) -0.0018(8) 0.0022(7) 0.0030(8)
C7 0.0313(9) 0.0460(11) 0.0615(12) 0.0034(10) 0.0071(8) -0.0003(8)
C4 0.096(2) 0.0743(18) 0.0633(14) 0.0210(13) 0.0409(16) 0.0302(15)
C16 0.0442(13) 0.0718(18) 0.119(2) 0.0263(16) 0.0101(14) -0.0081(10)
C6 0.0429(12) 0.0827(18) 0.098(2) 0.0354(16) 0.0248(13) 0.0086(12)
C23 0.0689(15) 0.0485(13) 0.0578(12) 0.0018(11) -0.0054(11) 0.0131(11)
C21 0.102(2) 0.0834(19) 0.0713(15) -0.0198(17) -0.0172(14) -0.011(2)
C17 0.095(2) 0.113(3) 0.093(2) -0.045(2) 0.0084(18) -0.035(2)
C8 0.0690(18) 0.085(2) 0.0640(15) 0.0011(14) -0.0044(12) -0.0311(15)
C12 0.155(4) 0.102(3) 0.0502(14) -0.0066(16) 0.0052(18) -0.035(2)
C5 0.0688(17) 0.104(2) 0.097(2) 0.0450(19) 0.0489(17) 0.0271(16)
C22 0.145(3) 0.0468(15) 0.0806(19) -0.0074(14) 0.014(2) -0.0097(17)
C11 0.101(2) 0.106(3) 0.103(2) -0.018(2) 0.047(2) 0.006(2)

_geom_special_details
;
 All esds (except the esd in the dihedral angle between two l.s. planes)
 are estimated using the full covariance matrix. The cell esds are taken
 into account individually in the estimation of esds in distances, angles
 and torsion angles; correlations between esds in cell parameters are only
 used when they are defined by crystal symmetry. An approximate (isotropic)
 treatment of cell esds is used for estimating esds involving l.s. planes.
;

loop_
 _geom_bond_atom_site_label_1
 _geom_bond_atom_site_label_2
 _geom_bond_distance
 _geom_bond_site_symmetry_2
 _geom_bond_publ_flag
Zn1 N2 1.9957(16) . ?
Zn1 N1 2.0724(17) . ?
Zn1 Cl1 2.2267(5) . ?

Zn1 Cl2 2.2333(5) . ?
N1 C7 1.273(3) . ?
N1 C9 1.493(3) . ?
N3 C13 1.334(3) . ?
N3 C18 1.467(3) . ?
N3 C19 1.468(3) . ?
O1 C7 1.336(3) . ?
O1 C8 1.445(3) . ?
O2 C23 1.408(3) . ?
O2 H2 1.01(4) . ?
N2 C13 1.301(3) . ?
N2 C14 1.487(2) . ?
C19 C23 1.518(3) . ?
C19 C20 1.540(3) . ?
C19 H19 0.9800 . ?
C14 C15 1.531(3) . ?
C14 C18 1.536(3) . ?
C14 H14 0.9800 . ?
C1 C2 1.395(3) . ?
C1 C6 1.403(3) . ?
C1 C7 1.479(3) . ?
C10 C11 1.500(5) . ?
C10 C9 1.523(3) . ?
C10 C12 1.535(4) . ?
C10 H10 0.9800 . ?
C18 H18A 0.9700 . ?
C18 H18B 0.9700 . ?
C3 C4 1.381(4) . ?
C3 C2 1.398(3) . ?
C3 H3 0.9300 . ?
C15 C17 1.528(5) . ?
C15 C16 1.535(3) . ?
C15 H15 0.9800 . ?
C20 C22 1.515(4) . ?
C20 C21 1.521(4) . ?
C20 H20 0.9800 . ?
C9 C8 1.535(3) . ?
C9 H9 0.9800 . ?
C2 C13 1.490(3) . ?
C4 C5 1.374(5) . ?
C4 H4 0.9300 . ?
C16 H16A 0.9600 . ?
C16 H16C 0.9600 . ?
C16 H16B 0.9600 . ?

C6 C5 1.369(5) . ?
C6 H6 0.9300 . ?
C23 H23A 0.9700 . ?
C23 H23B 0.9700 . ?
C21 H21A 0.9600 . ?
C21 H21C 0.9600 . ?
C21 H21B 0.9600 . ?
C17 H17A 0.9600 . ?
C17 H17C 0.9600 . ?
C17 H17B 0.9600 . ?
C8 H8B 0.9700 . ?
C8 H8A 0.9700 . ?
C12 H12B 0.9600 . ?
C12 H12C 0.9600 . ?
C12 H12A 0.9600 . ?
C5 H5 0.9300 . ?
C22 H22B 0.9600 . ?
C22 H22C 0.9600 . ?
C22 H22A 0.9600 . ?
C11 H11B 0.9600 . ?
C11 H11C 0.9600 . ?
C11 H11A 0.9600 . ?

loop_
 _geom_angle_atom_site_label_1
 _geom_angle_atom_site_label_2
 _geom_angle_atom_site_label_3
 _geom_angle
 _geom_angle_site_symmetry_1
 _geom_angle_site_symmetry_3
 _geom_angle_publ_flag
N2 Zn1 N1 94.14(7) . . ?
N2 Zn1 Cl1 108.40(5) . . ?
N1 Zn1 Cl1 113.63(5) . . ?
N2 Zn1 Cl2 117.03(5) . . ?
N1 Zn1 Cl2 106.36(5) . . ?
Cl1 Zn1 Cl2 115.44(3) . . ?
C7 N1 C9 108.11(17) . . ?
C7 N1 Zn1 124.40(14) . . ?
C9 N1 Zn1 127.49(13) . . ?
C13 N3 C18 108.77(16) . . ?
C13 N3 C19 128.87(16) . . ?
C18 N3 C19 122.35(16) . . ?
C7 O1 C8 106.85(18) . . ?

C23 O2 H2 105(2) . . ?
C13 N2 C14 108.09(15) . . ?
C13 N2 Zn1 124.43(13) . . ?
C14 N2 Zn1 124.52(13) . . ?
N3 C19 C23 109.60(18) . . ?
N3 C19 C20 112.09(17) . . ?
C23 C19 C20 112.82(18) . . ?
N3 C19 H19 107.3 . . ?
C23 C19 H19 107.3 . . ?
C20 C19 H19 107.3 . . ?
N2 C14 C15 110.57(17) . . ?
N2 C14 C18 103.98(15) . . ?
C15 C14 C18 114.56(18) . . ?
N2 C14 H14 109.2 . . ?
C15 C14 H14 109.2 . . ?
C18 C14 H14 109.2 . . ?
C2 C1 C6 118.6(2) . . ?
C2 C1 C7 125.22(17) . . ?
C6 C1 C7 116.2(2) . . ?
C11 C10 C9 113.0(2) . . ?
C11 C10 C12 113.3(3) . . ?
C9 C10 C12 108.9(3) . . ?
C11 C10 H10 107.1 . . ?
C9 C10 H10 107.1 . . ?
C12 C10 H10 107.1 . . ?
N3 C18 C14 103.21(15) . . ?
N3 C18 H18A 111.1 . . ?
C14 C18 H18A 111.1 . . ?
N3 C18 H18B 111.1 . . ?
C14 C18 H18B 111.1 . . ?
H18A C18 H18B 109.1 . . ?
C4 C3 C2 120.3(3) . . ?
C4 C3 H3 119.9 . . ?
C2 C3 H3 119.9 . . ?
C17 C15 C14 111.7(2) . . ?
C17 C15 C16 111.8(3) . . ?
C14 C15 C16 109.1(2) . . ?
C17 C15 H15 108.0 . . ?
C14 C15 H15 108.0 . . ?
C16 C15 H15 108.0 . . ?
C22 C20 C21 110.9(3) . . ?
C22 C20 C19 110.4(2) . . ?
C21 C20 C19 111.8(2) . . ?
C22 C20 H20 107.9 . . ?

C21 C20 H20 107.9 . . ?
C19 C20 H20 107.9 . . ?
N1 C9 C10 112.16(19) . . ?
N1 C9 C8 102.26(18) . . ?
C10 C9 C8 115.1(2) . . ?
N1 C9 H9 109.0 . . ?
C10 C9 H9 109.0 . . ?
C8 C9 H9 109.0 . . ?
C1 C2 C3 119.38(19) . . ?
C1 C2 C13 123.18(18) . . ?
C3 C2 C13 117.37(19) . . ?
N2 C13 N3 115.83(16) . . ?
N2 C13 C2 122.73(17) . . ?
N3 C13 C2 121.42(17) . . ?
N1 C7 O1 117.59(19) . . ?
N1 C7 C1 126.8(2) . . ?
O1 C7 C1 115.39(18) . . ?
C5 C4 C3 120.6(2) . . ?
C5 C4 H4 119.7 . . ?
C3 C4 H4 119.7 . . ?
C15 C16 H16A 109.5 . . ?
C15 C16 H16C 109.5 . . ?
H16A C16 H16C 109.5 . . ?
C15 C16 H16B 109.5 . . ?
H16A C16 H16B 109.5 . . ?
H16C C16 H16B 109.5 . . ?
C5 C6 C1 121.4(3) . . ?
C5 C6 H6 119.3 . . ?
C1 C6 H6 119.3 . . ?
O2 C23 C19 108.42(19) . . ?
O2 C23 H23A 110.0 . . ?
C19 C23 H23A 110.0 . . ?
O2 C23 H23B 110.0 . . ?
C19 C23 H23B 110.0 . . ?
H23A C23 H23B 108.4 . . ?
C20 C21 H21A 109.5 . . ?
C20 C21 H21C 109.5 . . ?
H21A C21 H21C 109.5 . . ?
C20 C21 H21B 109.5 . . ?
H21A C21 H21B 109.5 . . ?
H21C C21 H21B 109.5 . . ?
C15 C17 H17A 109.5 . . ?
C15 C17 H17C 109.5 . . ?
H17A C17 H17C 109.5 . . ?

C15 C17 H17B 109.5 . . ?
H17A C17 H17B 109.5 . . ?
H17C C17 H17B 109.5 . . ?
O1 C8 C9 105.15(18) . . ?
O1 C8 H8B 110.7 . . ?
C9 C8 H8B 110.7 . . ?
O1 C8 H8A 110.7 . . ?
C9 C8 H8A 110.7 . . ?
H8B C8 H8A 108.8 . . ?
C10 C12 H12B 109.5 . . ?
C10 C12 H12C 109.5 . . ?
H12B C12 H12C 109.5 . . ?
C10 C12 H12A 109.5 . . ?
H12B C12 H12A 109.5 . . ?
H12C C12 H12A 109.5 . . ?
C6 C5 C4 119.5(2) . . ?
C6 C5 H5 120.2 . . ?
C4 C5 H5 120.2 . . ?
C20 C22 H22B 109.5 . . ?
C20 C22 H22C 109.5 . . ?
H22B C22 H22C 109.5 . . ?
C20 C22 H22A 109.5 . . ?
H22B C22 H22A 109.5 . . ?
H22C C22 H22A 109.5 . . ?
C10 C11 H11B 109.5 . . ?
C10 C11 H11C 109.5 . . ?
H11B C11 H11C 109.5 . . ?
C10 C11 H11A 109.5 . . ?
H11B C11 H11A 109.5 . . ?
H11C C11 H11A 109.5 . . ?

loop_
 _geom_torsion_atom_site_label_1
 _geom_torsion_atom_site_label_2
 _geom_torsion_atom_site_label_3
 _geom_torsion_atom_site_label_4
 _geom_torsion
 _geom_torsion_site_symmetry_1
 _geom_torsion_site_symmetry_2
 _geom_torsion_site_symmetry_3
 _geom_torsion_site_symmetry_4
 _geom_torsion_publ_flag
N2 Zn1 N1 C7 51.53(17) . . . . ?
Cl1 Zn1 N1 C7 163.68(15) . . . . ?

Cl2 Zn1 N1 C7 -68.21(16) . . . . ?
N2 Zn1 N1 C9 -128.68(17) . . . . ?
Cl1 Zn1 N1 C9 -16.53(18) . . . . ?
Cl2 Zn1 N1 C9 111.58(16) . . . . ?
N1 Zn1 N2 C13 -43.62(17) . . . . ?
Cl1 Zn1 N2 C13 -160.21(15) . . . . ?
Cl2 Zn1 N2 C13 67.11(18) . . . . ?
N1 Zn1 N2 C14 114.57(16) . . . . ?
Cl1 Zn1 N2 C14 -2.02(17) . . . . ?
Cl2 Zn1 N2 C14 -134.70(14) . . . . ?
C13 N3 C19 C23 -120.5(2) . . . . ?
C18 N3 C19 C23 58.2(2) . . . . ?
C13 N3 C19 C20 113.4(2) . . . . ?
C18 N3 C19 C20 -67.9(2) . . . . ?
C13 N2 C14 C15 -120.4(2) . . . . ?
Zn1 N2 C14 C15 78.4(2) . . . . ?
C13 N2 C14 C18 3.0(2) . . . . ?
Zn1 N2 C14 C18 -158.17(14) . . . . ?
C13 N3 C18 C14 2.4(2) . . . . ?
C19 N3 C18 C14 -176.48(17) . . . . ?
N2 C14 C18 N3 -3.2(2) . . . . ?
C15 C14 C18 N3 117.6(2) . . . . ?
N2 C14 C15 C17 63.2(3) . . . . ?
C18 C14 C15 C17 -53.9(3) . . . . ?
N2 C14 C15 C16 -172.6(2) . . . . ?
C18 C14 C15 C16 70.3(3) . . . . ?
N3 C19 C20 C22 -174.0(2) . . . . ?
C23 C19 C20 C22 61.7(3) . . . . ?
N3 C19 C20 C21 -50.0(3) . . . . ?
C23 C19 C20 C21 -174.3(2) . . . . ?
C7 N1 C9 C10 -122.7(2) . . . . ?
Zn1 N1 C9 C10 57.5(2) . . . . ?
C7 N1 C9 C8 1.1(2) . . . . ?
Zn1 N1 C9 C8 -178.67(17) . . . . ?
C11 C10 C9 N1 57.1(3) . . . . ?
C12 C10 C9 N1 -176.1(2) . . . . ?
C11 C10 C9 C8 -59.3(3) . . . . ?
C12 C10 C9 C8 67.5(3) . . . . ?
C6 C1 C2 C3 -4.2(3) . . . . ?
C7 C1 C2 C3 173.1(2) . . . . ?
C6 C1 C2 C13 172.9(2) . . . . ?
C7 C1 C2 C13 -9.9(3) . . . . ?
C4 C3 C2 C1 0.4(3) . . . . ?
C4 C3 C2 C13 -176.8(2) . . . . ?

```
C14 N2 C13 N3 -1.7(2) . . . . ?
Zn1 N2 C13 N3 159.55(14) . . . . ?
C14 N2 C13 C2 177.14(18) . . . . ?
Zn1 N2 C13 C2 -21.6(3) . . . . ?
C18 N3 C13 N2 -0.6(2) . . . . ?
C19 N3 C13 N2 178.23(18) . . . . ?
C18 N3 C13 C2 -179.39(18) . . . . ?
C19 N3 C13 C2 -0.6(3) . . . . ?
C1 C2 C13 N2 68.4(3) . . . . ?
C3 C2 C13 N2 -114.5(2) . . . . ?
C1 C2 C13 N3 -112.9(2) . . . . ?
C3 C2 C13 N3 64.2(3) . . . . ?
C9 N1 C7 O1 0.1(3) . . . . ?
Zn1 N1 C7 O1 179.90(14) . . . . ?
C9 N1 C7 C1 -173.8(2) . . . . ?
Zn1 N1 C7 C1 6.0(3) . . . . ?
C8 O1 C7 N1 -1.4(3) . . . . ?
C8 O1 C7 C1 173.2(2) . . . . ?
C2 C1 C7 N1 -46.6(3) . . . . ?
C6 C1 C7 N1 130.7(2) . . . . ?
C2 C1 C7 O1 139.4(2) . . . . ?
C6 C1 C7 O1 -43.3(3) . . . . ?
C2 C3 C4 C5 3.0(4) . . . . ?
C2 C1 C6 C5 4.7(4) . . . . ?
C7 C1 C6 C5 -172.8(3) . . . . ?
N3 C19 C23 O2 52.9(2) . . . . ?
C20 C19 C23 O2 178.5(2) . . . . ?
C7 O1 C8 C9 1.9(3) . . . . ?
N1 C9 C8 O1 -1.8(3) . . . . ?
C10 C9 C8 O1 120.0(3) . . . . ?
C1 C6 C5 C4 -1.3(5) . . . . ?
C3 C4 C5 C6 -2.6(5) . . . . ?

loop_
 _geom_hbond_atom_site_label_D
 _geom_hbond_atom_site_label_H
 _geom_hbond_atom_site_label_A
 _geom_hbond_distance_DH
 _geom_hbond_distance_HA
 _geom_hbond_distance_DA
 _geom_hbond_angle_DHA
 _geom_hbond_site_symmetry_A
O2 H2 Cl2  1.01(4) 2.20(4) 3.213(2) 175(4) 2_646
```

```
_diffrn_measured_fraction_theta_max    0.996
_diffrn_reflns_theta_full              62.69
_diffrn_measured_fraction_theta_full   0.996
_refine_diff_density_max    0.122
_refine_diff_density_min   -0.278
_refine_diff_density_rms    0.040

data_compound_11

_audit_creation_method            SHELXL-97
_chemical_name_systematic
;
 ?
;
_chemical_name_common             ?
_chemical_melting_point           ?
_chemical_formula_moiety          ?
_chemical_formula_sum
 'C32 H48 N6 O4 Zn'
_chemical_formula_weight          646.13

loop_
 _atom_type_symbol
 _atom_type_description
 _atom_type_scat_dispersion_real
 _atom_type_scat_dispersion_imag
 _atom_type_scat_source
 'C'  'C'   0.0033   0.0016
 'International Tables Vol C Tables 4.2.6.8 and 6.1.1.4'
 'H'  'H'   0.0000   0.0000
 'International Tables Vol C Tables 4.2.6.8 and 6.1.1.4'
 'N'  'N'   0.0061   0.0033
 'International Tables Vol C Tables 4.2.6.8 and 6.1.1.4'
 'O'  'O'   0.0106   0.0060
 'International Tables Vol C Tables 4.2.6.8 and 6.1.1.4'
 'Zn'  'Zn'   0.2839   1.4301
 'International Tables Vol C Tables 4.2.6.8 and 6.1.1.4'

_symmetry_cell_setting            Trigonal
_symmetry_space_group_name_H-M    P3(2)21

loop_
 _symmetry_equiv_pos_as_xyz
 'x, y, z'
```

```
 '-y, x-y, z+2/3'
 '-x+y, -x, z+1/3'
 'y, x, -z'
 'x-y, -y, -z+1/3'
 '-x, -x+y, -z+2/3'

_cell_length_a                    12.0352(4)
_cell_length_b                    12.0352(4)
_cell_length_c                    20.9216(10)
_cell_angle_alpha                 90.00
_cell_angle_beta                  90.00
_cell_angle_gamma                 120.00
_cell_volume                      2624.41(18)
_cell_formula_units_Z             3
_cell_measurement_temperature     293(2)
_cell_measurement_reflns_used     4351
_cell_measurement_theta_min       4.366
_cell_measurement_theta_max       44.928

_exptl_crystal_description        prismatic
_exptl_crystal_colour             colorless
_exptl_crystal_size_max           0.321
_exptl_crystal_size_mid           0.225
_exptl_crystal_size_min           0.168
_exptl_crystal_density_meas       ?
_exptl_crystal_density_diffrn     1.226
_exptl_crystal_density_method     'not measured'
_exptl_crystal_F_000              1032
_exptl_absorpt_coefficient_mu     0.744
_exptl_absorpt_correction_type    empirical
_exptl_absorpt_correction_T_min   0.71902
_exptl_absorpt_correction_T_max   1.00000
_exptl_absorpt_process_details    sadabs

_exptl_special_details
;
 ?
;

_diffrn_ambient_temperature       293(2)
_diffrn_radiation_wavelength      0.71073
_diffrn_radiation_type            MoK\a
_diffrn_radiation_source          'fine-focus sealed tube'
_diffrn_radiation_monochromator   graphite
```

```
_diffrn_measurement_device_type   'CCD area detector'
_diffrn_measurement_method        'phi and omega scans'
_diffrn_detector_area_resol_mean  ?
_diffrn_standards_number          ?
_diffrn_standards_interval_count  ?
_diffrn_standards_interval_time   ?
_diffrn_standards_decay_%         ?
_diffrn_reflns_number             16046
_diffrn_reflns_av_R_equivalents   0.0323
_diffrn_reflns_av_sigmaI/netI     0.0357
_diffrn_reflns_limit_h_min        -10
_diffrn_reflns_limit_h_max        14
_diffrn_reflns_limit_k_min        -14
_diffrn_reflns_limit_k_max        13
_diffrn_reflns_limit_l_min        -25
_diffrn_reflns_limit_l_max        25
_diffrn_reflns_theta_min          1.95
_diffrn_reflns_theta_max          25.99
_reflns_number_total              3450
_reflns_number_gt                 3068
_reflns_threshold_expression      >2sigma(I)

_computing_data_collection        'Bruker SMART'
_computing_cell_refinement        'Bruker SMART'
_computing_data_reduction         'Bruker SHELXTL'
_computing_structure_solution     'SHELXS-97 (Sheldrick, 1990)'
_computing_structure_refinement   'SHELXL-97 (Sheldrick, 1997)'
_computing_molecular_graphics     'Bruker SHELXTL'
_computing_publication_material   'Bruker SHELXTL'

_refine_special_details
;
 Refinement of F^2^ against ALL reflections.  The weighted R-factor wR and
 goodness of fit S are based on F^2^, conventional R-factors R are based
 on F, with F set to zero for negative F^2^. The threshold expression of
 F^2^ > 2sigma(F^2^) is used only for calculating R-factors(gt) etc. and is
 not relevant to the choice of reflections for refinement.  R-factors based
 on F^2^ are statistically about twice as large as those based on F, and R-
 factors based on ALL data will be even larger.
;

_refine_ls_structure_factor_coef  Fsqd
_refine_ls_matrix_type            full
_refine_ls_weighting_scheme       calc
```

```
_refine_ls_weighting_details
 'calc w=1/[\s^2^(Fo^2^)+(0.0518P)^2^+0.0895P] where P=(Fo^2^+2Fc^2^)/3'
_atom_sites_solution_primary      direct
_atom_sites_solution_secondary    difmap
_atom_sites_solution_hydrogens    geom
_refine_ls_hydrogen_treatment     constr
_refine_ls_extinction_method      none
_refine_ls_extinction_coef        ?
_refine_ls_abs_structure_details
 'Flack H D (1983), Acta Cryst. A39, 876-881'
_refine_ls_abs_structure_Flack    -0.001(13)
_chemical_absolute_configuration  ad
_refine_ls_number_reflns          3450
_refine_ls_number_parameters      199
_refine_ls_number_restraints      0
_refine_ls_R_factor_all           0.0434
_refine_ls_R_factor_gt            0.0371
_refine_ls_wR_factor_ref          0.0935
_refine_ls_wR_factor_gt           0.0892
_refine_ls_goodness_of_fit_ref    1.062
_refine_ls_restrained_S_all       1.062
_refine_ls_shift/su_max           0.000
_refine_ls_shift/su_mean          0.000

loop_
 _atom_site_label
 _atom_site_type_symbol
 _atom_site_fract_x
 _atom_site_fract_y
 _atom_site_fract_z
 _atom_site_U_iso_or_equiv
 _atom_site_adp_type
 _atom_site_occupancy
 _atom_site_symmetry_multiplicity
 _atom_site_calc_flag
 _atom_site_refinement_flags
 _atom_site_disorder_assembly
 _atom_site_disorder_group
Zn1 Zn 0.60961(3) 0.60961(3) 0.0000 0.05223(13) Uani 1 2 d S . .
N1 N 0.4599(2) 0.6008(2) 0.04368(9) 0.0560(5) Uani 1 1 d . . .
N2 N 0.71644(19) 0.6473(2) 0.07793(9) 0.0533(5) Uani 1 1 d . . .
N3 N 0.5050(3) 0.6579(3) 0.26804(11) 0.0864(8) Uani 1 1 d . . .
O1 O 0.35095(18) 0.6146(2) 0.12621(8) 0.0687(5) Uani 1 1 d . . .
O2 O 0.75123(18) 0.6674(2) 0.18333(8) 0.0677(5) Uani 1 1 d . . .
```

```
C1 C 0.5517(3) 0.6367(2) 0.15035(11) 0.0532(6) Uani 1 1 d . . .
C2 C 0.5269(3) 0.6488(3) 0.21571(12) 0.0640(7) Uani 1 1 d . . .
C3 C 0.4574(2) 0.6171(2) 0.10428(11) 0.0537(5) Uani 1 1 d . . .
C4 C 0.2780(3) 0.6122(3) 0.07023(13) 0.0739(8) Uani 1 1 d . . .
H4A H 0.2841 0.6949 0.0638 0.089 Uiso 1 1 calc R . .
H4B H 0.1883 0.5473 0.0747 0.089 Uiso 1 1 calc R . .
C5 C 0.3392(3) 0.5803(3) 0.01471(12) 0.0595(6) Uani 1 1 d . . .
H5 H 0.3588 0.6423 -0.0199 0.071 Uiso 1 1 calc R . .
C6 C 0.2625(3) 0.4444(3) -0.01180(14) 0.0722(7) Uani 1 1 d . . .
H6A H 0.3201 0.4264 -0.0363 0.087 Uiso 1 1 calc R . .
H6B H 0.2312 0.3849 0.0239 0.087 Uiso 1 1 calc R . .
C7 C 0.1505(3) 0.4186(3) -0.05335(16) 0.0845(9) Uani 1 1 d . . .
H7 H 0.0982 0.4481 -0.0312 0.101 Uiso 1 1 calc R . .
C8 C 0.0685(5) 0.2734(4) -0.0653(2) 0.1376(18) Uani 1 1 d . . .
H8A H -0.0068 0.2558 -0.0891 0.206 Uiso 1 1 calc R . .
H8B H 0.0436 0.2291 -0.0251 0.206 Uiso 1 1 calc R . .
H8C H 0.1175 0.2446 -0.0892 0.206 Uiso 1 1 calc R . .
C9 C 0.1928(5) 0.4861(5) -0.11613(19) 0.140(2) Uani 1 1 d . . .
H9A H 0.2375 0.5772 -0.1093 0.210 Uiso 1 1 calc R . .
H9B H 0.1192 0.4623 -0.1427 0.210 Uiso 1 1 calc R . .
H9C H 0.2490 0.4623 -0.1368 0.210 Uiso 1 1 calc R . .
C10 C 0.6715(2) 0.6494(2) 0.13416(11) 0.0523(5) Uani 1 1 d . . .
C11 C 0.8729(3) 0.6948(3) 0.15609(14) 0.0753(8) Uani 1 1 d . . .
H11A H 0.9049 0.6443 0.1768 0.090 Uiso 1 1 calc R . .
H11B H 0.9361 0.7850 0.1606 0.090 Uiso 1 1 calc R . .
C12 C 0.8443(3) 0.6583(3) 0.08562(13) 0.0622(7) Uani 1 1 d . . .
H12 H 0.8365 0.5741 0.0786 0.075 Uiso 1 1 calc R . .
C13 C 0.9469(3) 0.7558(3) 0.04230(14) 0.0728(8) Uani 1 1 d . . .
H13A H 1.0295 0.7722 0.0582 0.087 Uiso 1 1 calc R . .
H13B H 0.9453 0.8353 0.0459 0.087 Uiso 1 1 calc R . .
C14 C 0.9390(3) 0.7222(4) -0.02803(15) 0.0822(10) Uani 1 1 d . . .
H14 H 0.8515 0.6950 -0.0422 0.099 Uiso 1 1 calc R . .
C15 C 1.0302(4) 0.8420(5) -0.06697(19) 0.1211(16) Uani 1 1 d . . .
H15A H 1.0134 0.9102 -0.0572 0.182 Uiso 1 1 calc R . .
H15B H 1.0166 0.8224 -0.1118 0.182 Uiso 1 1 calc R . .
H15C H 1.1174 0.8680 -0.0562 0.182 Uiso 1 1 calc R . .
C16 C 0.9639(5) 0.6159(5) -0.0424(2) 0.1264(16) Uani 1 1 d . . .
H16A H 1.0480 0.6381 -0.0275 0.190 Uiso 1 1 calc R . .
H16B H 0.9587 0.6015 -0.0877 0.190 Uiso 1 1 calc R . .
H16C H 0.9010 0.5393 -0.0212 0.190 Uiso 1 1 calc R . .

loop_
 _atom_site_aniso_label
 _atom_site_aniso_U_11
```

```
 _atom_site_aniso_U_22
 _atom_site_aniso_U_33
 _atom_site_aniso_U_23
 _atom_site_aniso_U_13
 _atom_site_aniso_U_12
Zn1 0.05722(19) 0.05722(19) 0.04368(19) -0.00122(9) 0.00122(9) 0.0297(2)
N1 0.0546(12) 0.0653(13) 0.0486(11) -0.0033(10) -0.0022(9) 0.0304(11)
N2 0.0518(11) 0.0592(12) 0.0500(10) -0.0001(9) -0.0027(9) 0.0285(10)
N3 0.0790(17) 0.109(2) 0.0547(14) -0.0072(14) 0.0068(12) 0.0344(17)
O1 0.0601(11) 0.0891(14) 0.0630(11) -0.0091(9) 0.0028(9) 0.0417(11)
O2 0.0651(11) 0.0874(15) 0.0516(9) 0.0025(9) -0.0074(8) 0.0388(11)
C1 0.0570(15) 0.0558(15) 0.0463(11) -0.0024(11) 0.0015(11) 0.0279(13)
C2 0.0580(15) 0.0656(17) 0.0565(14) -0.0041(13) 0.0036(12) 0.0221(14)
C3 0.0539(14) 0.0490(14) 0.0555(13) 0.0018(11) 0.0062(10) 0.0236(12)
C4 0.0597(17) 0.090(2) 0.0783(18) -0.0004(16) -0.0013(14) 0.0419(17)
C5 0.0572(15) 0.0637(16) 0.0605(14) 0.0046(12) -0.0015(11) 0.0323(13)
C6 0.0718(17) 0.0661(18) 0.0748(17) 0.0074(15) -0.0049(14) 0.0316(16)
C7 0.0715(19) 0.090(2) 0.088(2) -0.0102(17) -0.0119(17) 0.0374(17)
C8 0.126(4) 0.100(3) 0.122(3) -0.009(3) -0.031(3) 0.008(3)
C9 0.151(4) 0.116(3) 0.090(3) 0.020(3) -0.042(3) 0.019(3)
C10 0.0579(13) 0.0502(13) 0.0478(12) 0.0025(10) -0.0034(9) 0.0264(12)
C11 0.0634(18) 0.095(2) 0.0656(15) 0.0089(16) -0.0077(14) 0.0381(16)
C12 0.0572(15) 0.0726(18) 0.0639(14) 0.0065(13) 0.0000(12) 0.0378(14)
C13 0.0566(16) 0.085(2) 0.0778(18) 0.0052(15) 0.0018(14) 0.0360(15)
C14 0.0582(17) 0.117(3) 0.0705(19) 0.0121(18) 0.0108(14) 0.0434(18)
C15 0.109(3) 0.176(5) 0.093(2) 0.038(3) 0.035(2) 0.083(3)
C16 0.138(4) 0.142(4) 0.114(3) -0.003(3) 0.031(3) 0.081(4)

_geom_special_details
;
 All esds (except the esd in the dihedral angle between two l.s. planes)
 are estimated using the full covariance matrix.  The cell esds are taken
 into account individually in the estimation of esds in distances, angles
 and torsion angles; correlations between esds in cell parameters are only
 used when they are defined by crystal symmetry.  An approximate (isotropic)
 treatment of cell esds is used for estimating esds involving l.s. planes.
;

loop_
 _geom_bond_atom_site_label_1
 _geom_bond_atom_site_label_2
 _geom_bond_distance
 _geom_bond_site_symmetry_2
 _geom_bond_publ_flag
```

Zn1 N1 1.975(2) . ?
Zn1 N1 1.975(2) 4 ?
Zn1 N2 1.9833(18) . ?
Zn1 N2 1.9834(18) 4 ?
N1 C3 1.285(3) . ?
N1 C5 1.477(3) . ?
N2 C10 1.300(3) . ?
N2 C12 1.486(3) . ?
N3 C2 1.144(3) . ?
O1 C3 1.347(3) . ?
O1 C4 1.456(3) . ?
O2 C10 1.349(3) . ?
O2 C11 1.447(4) . ?
C1 C10 1.413(4) . ?
C1 C3 1.416(4) . ?
C1 C2 1.423(3) . ?
C4 C5 1.524(4) . ?
C4 H4A 0.9700 . ?
C4 H4B 0.9700 . ?
C5 C6 1.525(4) . ?
C5 H5 0.9800 . ?
C6 C7 1.500(4) . ?
C6 H6A 0.9700 . ?
C6 H6B 0.9700 . ?
C7 C9 1.494(5) . ?
C7 C8 1.539(6) . ?
C7 H7 0.9800 . ?
C8 H8A 0.9600 . ?
C8 H8B 0.9600 . ?
C8 H8C 0.9600 . ?
C9 H9A 0.9600 . ?
C9 H9B 0.9600 . ?
C9 H9C 0.9600 . ?
C11 C12 1.528(4) . ?
C11 H11A 0.9700 . ?
C11 H11B 0.9700 . ?
C12 C13 1.509(4) . ?
C12 H12 0.9800 . ?
C13 C14 1.516(4) . ?
C13 H13A 0.9700 . ?
C13 H13B 0.9700 . ?
C14 C16 1.483(6) . ?
C14 C15 1.537(5) . ?
C14 H14 0.9800 . ?

```
C15 H15A 0.9600 . ?
C15 H15B 0.9600 . ?
C15 H15C 0.9600 . ?
C16 H16A 0.9600 . ?
C16 H16B 0.9600 . ?
C16 H16C 0.9600 . ?

loop_
 _geom_angle_atom_site_label_1
 _geom_angle_atom_site_label_2
 _geom_angle_atom_site_label_3
 _geom_angle
 _geom_angle_site_symmetry_1
 _geom_angle_site_symmetry_3
 _geom_angle_publ_flag
N1 Zn1 N1 122.26(13) . 4 ?
N1 Zn1 N2 95.81(7) . . ?
N1 Zn1 N2 108.79(8) 4 . ?
N1 Zn1 N2 108.80(8) . 4 ?
N1 Zn1 N2 95.82(7) 4 4 ?
N2 Zn1 N2 127.97(12) . 4 ?
C3 N1 C5 109.5(2) . . ?
C3 N1 Zn1 122.61(17) . . ?
C5 N1 Zn1 127.86(15) . . ?
C10 N2 C12 108.8(2) . . ?
C10 N2 Zn1 121.31(16) . . ?
C12 N2 Zn1 129.29(16) . . ?
C3 O1 C4 106.49(19) . . ?
C10 O2 C11 107.09(19) . . ?
C10 C1 C3 123.0(2) . . ?
C10 C1 C2 118.3(2) . . ?
C3 C1 C2 118.7(2) . . ?
N3 C2 C1 178.9(3) . . ?
N1 C3 O1 115.3(2) . . ?
N1 C3 C1 128.2(2) . . ?
O1 C3 C1 116.5(2) . . ?
O1 C4 C5 104.8(2) . . ?
O1 C4 H4A 110.8 . . ?
C5 C4 H4A 110.8 . . ?
O1 C4 H4B 110.8 . . ?
C5 C4 H4B 110.8 . . ?
H4A C4 H4B 108.9 . . ?
N1 C5 C4 101.81(19) . . ?
N1 C5 C6 110.3(2) . . ?
```

C4 C5 C6 115.9(2) . . ?
N1 C5 H5 109.5 . . ?
C4 C5 H5 109.5 . . ?
C6 C5 H5 109.5 . . ?
C7 C6 C5 115.6(3) . . ?
C7 C6 H6A 108.4 . . ?
C5 C6 H6A 108.4 . . ?
C7 C6 H6B 108.4 . . ?
C5 C6 H6B 108.4 . . ?
H6A C6 H6B 107.4 . . ?
C9 C7 C6 111.7(3) . . ?
C9 C7 C8 109.0(3) . . ?
C6 C7 C8 109.2(3) . . ?
C9 C7 H7 108.9 . . ?
C6 C7 H7 108.9 . . ?
C8 C7 H7 108.9 . . ?
C7 C8 H8A 109.5 . . ?
C7 C8 H8B 109.5 . . ?
H8A C8 H8B 109.5 . . ?
C7 C8 H8C 109.5 . . ?
H8A C8 H8C 109.5 . . ?
H8B C8 H8C 109.5 . . ?
C7 C9 H9A 109.5 . . ?
C7 C9 H9B 109.5 . . ?
H9A C9 H9B 109.5 . . ?
C7 C9 H9C 109.5 . . ?
H9A C9 H9C 109.5 . . ?
H9B C9 H9C 109.5 . . ?
N2 C10 O2 115.1(2) . . ?
N2 C10 C1 128.8(2) . . ?
O2 C10 C1 116.1(2) . . ?
O2 C11 C12 104.9(2) . . ?
O2 C11 H11A 110.8 . . ?
C12 C11 H11A 110.8 . . ?
O2 C11 H11B 110.8 . . ?
C12 C11 H11B 110.8 . . ?
H11A C11 H11B 108.8 . . ?
N2 C12 C13 114.4(2) . . ?
N2 C12 C11 101.8(2) . . ?
C13 C12 C11 112.1(2) . . ?
N2 C12 H12 109.4 . . ?
C13 C12 H12 109.4 . . ?
C11 C12 H12 109.4 . . ?
C12 C13 C14 117.2(3) . . ?

```
C12 C13 H13A 108.0 . . ?
C14 C13 H13A 108.0 . . ?
C12 C13 H13B 108.0 . . ?
C14 C13 H13B 108.0 . . ?
H13A C13 H13B 107.2 . . ?
C16 C14 C13 114.4(3) . . ?
C16 C14 C15 110.2(3) . . ?
C13 C14 C15 110.1(3) . . ?
C16 C14 H14 107.3 . . ?
C13 C14 H14 107.3 . . ?
C15 C14 H14 107.3 . . ?
C14 C15 H15A 109.5 . . ?
C14 C15 H15B 109.5 . . ?
H15A C15 H15B 109.5 . . ?
C14 C15 H15C 109.5 . . ?
H15A C15 H15C 109.5 . . ?
H15B C15 H15C 109.5 . . ?
C14 C16 H16A 109.5 . . ?
C14 C16 H16B 109.5 . . ?
H16A C16 H16B 109.5 . . ?
C14 C16 H16C 109.5 . . ?
H16A C16 H16C 109.5 . . ?
H16B C16 H16C 109.5 . . ?

loop_
 _geom_torsion_atom_site_label_1
 _geom_torsion_atom_site_label_2
 _geom_torsion_atom_site_label_3
 _geom_torsion_atom_site_label_4
 _geom_torsion
 _geom_torsion_site_symmetry_1
 _geom_torsion_site_symmetry_2
 _geom_torsion_site_symmetry_3
 _geom_torsion_site_symmetry_4
 _geom_torsion_publ_flag
N1 Zn1 N1 C3 116.1(2) 4 . . . ?
N2 Zn1 N1 C3 -0.5(2) . . . . ?
N2 Zn1 N1 C3 -134.0(2) 4 . . . ?
N1 Zn1 N1 C5 -66.2(2) 4 . . . ?
N2 Zn1 N1 C5 177.2(2) . . . . ?
N2 Zn1 N1 C5 43.8(2) 4 . . . ?
N1 Zn1 N2 C10 4.4(2) . . . . ?
N1 Zn1 N2 C10 -122.6(2) 4 . . . ?
N2 Zn1 N2 C10 123.7(2) 4 . . . ?
```

N1 Zn1 N2 C12 174.8(2) . . . . ?

N1 Zn1 N2 C12 47.8(2) 4 . . . ?

N2 Zn1 N2 C12 -65.9(2) 4 . . . ?

C10 C1 C2 N3 -172(100) . . . . ?

C3 C1 C2 N3 10(19) . . . . ?

C5 N1 C3 O1 -1.4(3) . . . . ?

Zn1 N1 C3 O1 176.73(17) . . . . ?

C5 N1 C3 C1 178.4(3) . . . . ?

Zn1 N1 C3 C1 -3.5(4) . . . . ?

C4 O1 C3 N1 -8.2(3) . . . . ?

C4 O1 C3 C1 172.0(2) . . . . ?

C10 C1 C3 N1 4.2(5) . . . . ?

C2 C1 C3 N1 -178.2(3) . . . . ?

C10 C1 C3 O1 -176.0(2) . . . . ?

C2 C1 C3 O1 1.7(4) . . . . ?

C3 O1 C4 C5 13.6(3) . . . . ?

C3 N1 C5 C4 9.7(3) . . . . ?

Zn1 N1 C5 C4 -168.3(2) . . . . ?

C3 N1 C5 C6 -113.9(3) . . . . ?

Zn1 N1 C5 C6 68.1(3) . . . . ?

O1 C4 C5 N1 -13.8(3) . . . . ?

O1 C4 C5 C6 105.9(3) . . . . ?

N1 C5 C6 C7 -168.8(2) . . . . ?

C4 C5 C6 C7 76.2(3) . . . . ?

C5 C6 C7 C9 70.3(4) . . . . ?

C5 C6 C7 C8 -169.1(3) . . . . ?

C12 N2 C10 O2 3.4(3) . . . . ?

Zn1 N2 C10 O2 175.52(16) . . . . ?

C12 N2 C10 C1 -177.3(3) . . . . ?

Zn1 N2 C10 C1 -5.1(4) . . . . ?

C11 O2 C10 N2 6.9(3) . . . . ?

C11 O2 C10 C1 -172.6(2) . . . . ?

C3 C1 C10 N2 0.8(5) . . . . ?

C2 C1 C10 N2 -176.8(3) . . . . ?

C3 C1 C10 O2 -179.8(2) . . . . ?

C2 C1 C10 O2 2.5(4) . . . . ?

C10 O2 C11 C12 -13.6(3) . . . . ?

C10 N2 C12 C13 -132.5(2) . . . . ?

Zn1 N2 C12 C13 56.2(3) . . . . ?

C10 N2 C12 C11 -11.3(3) . . . . ?

Zn1 N2 C12 C11 177.34(18) . . . . ?

O2 C11 C12 N2 14.7(3) . . . . ?

O2 C11 C12 C13 137.4(3) . . . . ?

N2 C12 C13 C14 -72.7(4) . . . . ?

```
C11 C12 C13 C14 172.1(3) . . . . ?
C12 C13 C14 C16 -68.3(4) . . . . ?
C12 C13 C14 C15 167.0(3) . . . . ?

_diffrn_measured_fraction_theta_max    1.000
_diffrn_reflns_theta_full              25.99
_diffrn_measured_fraction_theta_full   1.000
_refine_diff_density_max    0.402
_refine_diff_density_min   -0.170
_refine_diff_density_rms    0.044

data_compound_12

_audit_creation_method            SHELXL-97
_chemical_name_systematic
;
 ?
;
_chemical_name_common             ?
_chemical_melting_point           ?
_chemical_formula_moiety          ?
_chemical_formula_sum
 'C40 H32 N6 O4 Zn'
_chemical_formula_weight          726.09

loop_
 _atom_type_symbol
 _atom_type_description
 _atom_type_scat_dispersion_real
 _atom_type_scat_dispersion_imag
 _atom_type_scat_source
 'C'  'C'   0.0033   0.0016
 'International Tables Vol C Tables 4.2.6.8 and 6.1.1.4'
 'H'  'H'   0.0000   0.0000
 'International Tables Vol C Tables 4.2.6.8 and 6.1.1.4'
 'N'  'N'   0.0061   0.0033
 'International Tables Vol C Tables 4.2.6.8 and 6.1.1.4'
 'O'  'O'   0.0106   0.0060
 'International Tables Vol C Tables 4.2.6.8 and 6.1.1.4'
 'Zn'  'Zn'   0.2839   1.4301
 'International Tables Vol C Tables 4.2.6.8 and 6.1.1.4'

_symmetry_cell_setting            Orthorhombic
_symmetry_space_group_name_H-M    P2(1)2(1)2(1)
```

```
loop_
 _symmetry_equiv_pos_as_xyz
 'x, y, z'
 'x+1/2, -y+1/2, -z'
 '-x, y+1/2, -z+1/2'
 '-x+1/2, -y, z+1/2'

_cell_length_a                    10.5470(15)
_cell_length_b                    15.264(2)
_cell_length_c                    21.146(3)
_cell_angle_alpha                 90.00
_cell_angle_beta                  90.00
_cell_angle_gamma                 90.00
_cell_volume                      3404.3(8)
_cell_formula_units_Z             4
_cell_measurement_temperature     133(2)
_cell_measurement_reflns_used     3988
_cell_measurement_theta_min       2.34
_cell_measurement_theta_max       24.20

_exptl_crystal_description        block
_exptl_crystal_colour             brown
_exptl_crystal_size_max           0.25
_exptl_crystal_size_mid           0.08
_exptl_crystal_size_min           0.04
_exptl_crystal_density_meas       ?
_exptl_crystal_density_diffrn     1.417
_exptl_crystal_density_method     'not measured'
_exptl_crystal_F_000              1504
_exptl_absorpt_coefficient_mu     0.774
_exptl_absorpt_correction_type    multi-scan
_exptl_absorpt_correction_T_min   0.8300
_exptl_absorpt_correction_T_max   0.9697
_exptl_absorpt_process_details    sadabs

_exptl_special_details
;
 ?
;

_diffrn_ambient_temperature       133(2)
_diffrn_radiation_wavelength      0.71073
_diffrn_radiation_type            MoK\a
```

```
_diffrn_radiation_source          'fine-focus sealed tube'
_diffrn_radiation_monochromator   graphite
_diffrn_measurement_device_type   'Bruker APEX-II CCD'
_diffrn_measurement_method        '\f and \w scans'
_diffrn_detector_area_resol_mean  ?
_diffrn_reflns_number             27153
_diffrn_reflns_av_R_equivalents   0.0734
_diffrn_reflns_av_sigmaI/netI     0.0751
_diffrn_reflns_limit_h_min        -13
_diffrn_reflns_limit_h_max        13
_diffrn_reflns_limit_k_min        -19
_diffrn_reflns_limit_k_max        17
_diffrn_reflns_limit_l_min        -27
_diffrn_reflns_limit_l_max        27
_diffrn_reflns_theta_min          1.65
_diffrn_reflns_theta_max          27.51
_reflns_number_total              7824
_reflns_number_gt                 5963
_reflns_threshold_expression      >2sigma(I)

_computing_data_collection        'Bruker APEX2'
_computing_cell_refinement        'Bruker SAINT'
_computing_data_reduction         'Bruker SAINT'
_computing_structure_solution     'SHELXS-97 (Sheldrick, 2008)'
_computing_structure_refinement   'SHELXL-97 (Sheldrick, 2008)'
_computing_molecular_graphics     'Bruker SHELXTL'
_computing_publication_material   'Bruker SHELXTL'

_refine_special_details
;
 Refinement of F^2^ against ALL reflections.  The weighted R-factor wR and
 goodness of fit S are based on F^2^, conventional R-factors R are based
 on F, with F set to zero for negative F^2^. The threshold expression of
 F^2^ > 2sigma(F^2^) is used only for calculating R-factors(gt) etc. and is
 not relevant to the choice of reflections for refinement.  R-factors based
 on F^2^ are statistically about twice as large as those based on F, and R-
 factors based on ALL data will be even larger.
;

_refine_ls_structure_factor_coef  Fsqd
_refine_ls_matrix_type            full
_refine_ls_weighting_scheme       calc
_refine_ls_weighting_details
 'calc w=1/[\s^2^(Fo^2^)+(0.0372P)^2^+0.0000P] where P=(Fo^2^+2Fc^2^)/3'
```

```
_atom_sites_solution_primary      direct
_atom_sites_solution_secondary    difmap
_atom_sites_solution_hydrogens    geom
_refine_ls_hydrogen_treatment     constr
_refine_ls_extinction_method      none
_refine_ls_extinction_coef        ?
_refine_ls_abs_structure_details
 'Flack H D (1983), Acta Cryst. A39, 876-881'
_refine_ls_abs_structure_Flack    0.001(13)
_chemical_absolute_configuration  ad
_refine_ls_number_reflns          7824
_refine_ls_number_parameters      460
_refine_ls_number_restraints      1
_refine_ls_R_factor_all           0.0689
_refine_ls_R_factor_gt            0.0418
_refine_ls_wR_factor_ref          0.0953
_refine_ls_wR_factor_gt           0.0817
_refine_ls_goodness_of_fit_ref    1.053
_refine_ls_restrained_S_all       1.054
_refine_ls_shift/su_max           0.001
_refine_ls_shift/su_mean          0.000

loop_
 _atom_site_label
 _atom_site_type_symbol
 _atom_site_fract_x
 _atom_site_fract_y
 _atom_site_fract_z
 _atom_site_U_iso_or_equiv
 _atom_site_adp_type
 _atom_site_occupancy
 _atom_site_symmetry_multiplicity
 _atom_site_calc_flag
 _atom_site_refinement_flags
 _atom_site_disorder_assembly
 _atom_site_disorder_group
Zn1 Zn 0.49733(4) 0.009807(18) 0.004895(14) 0.01653(8) Uani 1 1 d . . .
N1 N 0.4443(3) 0.08753(16) 0.07627(12) 0.0180(6) Uani 1 1 d . . .
N2 N 0.3311(2) -0.05386(16) 0.00397(13) 0.0171(5) Uani 1 1 d . . .
N3 N 0.1263(3) -0.0490(2) 0.20384(16) 0.0413(9) Uani 1 1 d . . .
N4 N 0.6607(2) -0.04014(18) 0.03723(12) 0.0183(6) Uani 1 1 d . . .
N5 N 0.5588(2) 0.01974(17) -0.08327(11) 0.0177(6) Uani 1 1 d . . .
N6 N 0.9385(3) -0.1381(2) -0.12471(17) 0.0466(9) Uani 1 1 d . . .
O1 O 0.3587(2) 0.10404(15) 0.17297(10) 0.0246(5) Uani 1 1 d . . .
```

O2 O 0.1565(2) -0.11074(16) 0.05102(11) 0.0247(6) Uani 1 1 d . . .
O3 O 0.8560(2) -0.10024(17) 0.02780(11) 0.0236(6) Uani 1 1 d . . .
O4 O 0.6734(2) -0.02586(17) -0.16605(10) 0.0269(6) Uani 1 1 d . . .
C1 C 0.2741(3) -0.0084(2) 0.11113(14) 0.0204(7) Uani 1 1 d . . .
C2 C 0.1909(3) -0.0302(2) 0.16281(17) 0.0266(8) Uani 1 1 d . . .
C3 C 0.3620(3) 0.0602(2) 0.11751(15) 0.0193(7) Uani 1 1 d . . .
C4 C 0.4529(4) 0.1711(2) 0.17075(17) 0.0322(9) Uani 1 1 d . . .
H4A H 0.5154 0.1635 0.2053 0.039 Uiso 1 1 calc R . .
H4B H 0.4136 0.2298 0.1747 0.039 Uiso 1 1 calc R . .
C5 C 0.5179(4) 0.1603(2) 0.10478(15) 0.0239(8) Uani 1 1 d . . .
H5 H 0.6080 0.1417 0.1107 0.029 Uiso 1 1 calc R . .
C6 C 0.5134(3) 0.24357(19) 0.06644(14) 0.0203(7) Uani 1 1 d . . .
C7 C 0.6244(3) 0.2906(3) 0.05553(17) 0.0254(8) Uani 1 1 d . . .
H7 H 0.7040 0.2671 0.0680 0.030 Uiso 1 1 calc R . .
C8 C 0.6184(4) 0.3720(3) 0.02632(19) 0.0324(10) Uani 1 1 d . . .
H8 H 0.6944 0.4041 0.0195 0.039 Uiso 1 1 calc R . .
C9 C 0.5059(4) 0.40659(19) 0.00725(17) 0.0348(8) Uani 1 1 d . . .
H9 H 0.5032 0.4629 -0.0118 0.042 Uiso 1 1 calc R . .
C10 C 0.3937(4) 0.3585(3) 0.0159(2) 0.0347(10) Uani 1 1 d . . .
H10 H 0.3149 0.3814 0.0017 0.042 Uiso 1 1 calc R . .
C11 C 0.3988(3) 0.2776(3) 0.04532(18) 0.0279(9) Uani 1 1 d . . .
H11 H 0.3230 0.2449 0.0511 0.033 Uiso 1 1 calc R . .
C12 C 0.2610(3) -0.0562(2) 0.05464(16) 0.0202(7) Uani 1 1 d . . .
C13 C 0.1709(3) -0.1594(2) -0.00731(17) 0.0255(7) Uani 1 1 d . . .
H13A H 0.0884 -0.1675 -0.0288 0.031 Uiso 1 1 calc R . .
H13B H 0.2097 -0.2175 0.0004 0.031 Uiso 1 1 calc R . .
C14 C 0.2600(3) -0.1000(2) -0.04635(15) 0.0203(7) Uani 1 1 d . . .
H14 H 0.2064 -0.0558 -0.0688 0.024 Uiso 1 1 calc R . .
C15 C 0.3374(3) -0.1474(2) -0.09513(16) 0.0198(7) Uani 1 1 d . . .
C16 C 0.4530(3) -0.1833(2) -0.08155(16) 0.0248(8) Uani 1 1 d . . .
H16 H 0.4864 -0.1781 -0.0400 0.030 Uiso 1 1 calc R . .
C17 C 0.5236(4) -0.2278(2) -0.12807(17) 0.0304(9) Uani 1 1 d . . .
H17 H 0.6060 -0.2497 -0.1192 0.036 Uiso 1 1 calc R . .
C18 C 0.4686(4) -0.2388(2) -0.18767(18) 0.0400(11) Uani 1 1 d U . .
H18 H 0.5125 -0.2711 -0.2192 0.048 Uiso 1 1 calc R . .
C19 C 0.3527(4) -0.2039(3) -0.20109(18) 0.0371(9) Uani 1 1 d U . .
H19 H 0.3171 -0.2112 -0.2420 0.044 Uiso 1 1 calc R . .
C20 C 0.2868(4) -0.1580(2) -0.15563(17) 0.0301(9) Uani 1 1 d . . .
H20 H 0.2064 -0.1335 -0.1655 0.036 Uiso 1 1 calc R . .
C21 C 0.7530(3) -0.0662(2) -0.06745(16) 0.0215(8) Uani 1 1 d . . .
C22 C 0.8564(3) -0.1058(3) -0.09921(17) 0.0284(8) Uani 1 1 d . . .
C23 C 0.7515(3) -0.06796(18) -0.00031(17) 0.0206(7) Uani 1 1 d . . .
C24 C 0.8280(3) -0.1043(2) 0.09473(16) 0.0241(8) Uani 1 1 d . . .
H24A H 0.9016 -0.0849 0.1202 0.029 Uiso 1 1 calc R . .

H24B H 0.8042 -0.1644 0.1076 0.029 Uiso 1 1 calc R . .
C25 C 0.7150(3) -0.0404(2) 0.10230(15) 0.0215(8) Uani 1 1 d . . .
H25 H 0.7487 0.0194 0.1119 0.026 Uiso 1 1 calc R . .
C26 C 0.6246(3) -0.0667(2) 0.15402(16) 0.0209(7) Uani 1 1 d . . .
C27 C 0.6276(4) -0.0235(3) 0.21163(16) 0.0301(8) Uani 1 1 d . . .
H27 H 0.6815 0.0260 0.2163 0.036 Uiso 1 1 calc R . .
C28 C 0.5561(4) -0.0494(3) 0.26162(18) 0.0415(10) Uani 1 1 d . . .
H28 H 0.5642 -0.0208 0.3013 0.050 Uiso 1 1 calc R . .
C29 C 0.4706(4) -0.1185(3) 0.25421(18) 0.0423(11) Uani 1 1 d . . .
H29 H 0.4163 -0.1348 0.2881 0.051 Uiso 1 1 calc R . .
C30 C 0.4655(4) -0.1633(3) 0.19702(19) 0.0369(10) Uani 1 1 d . . .
H30 H 0.4083 -0.2109 0.1917 0.044 Uiso 1 1 calc R . .
C31 C 0.5454(3) -0.1377(2) 0.14721(18) 0.0304(9) Uani 1 1 d . . .
H31 H 0.5449 -0.1696 0.1086 0.036 Uiso 1 1 calc R . .
C32 C 0.6563(3) -0.0223(2) -0.10315(14) 0.0196(7) Uani 1 1 d . . .
C33 C 0.5654(3) 0.0129(2) -0.19505(15) 0.0279(8) Uani 1 1 d . . .
H33A H 0.5160 -0.0315 -0.2186 0.033 Uiso 1 1 calc R . .
H33B H 0.5912 0.0599 -0.2246 0.033 Uiso 1 1 calc R . .
C34 C 0.4860(4) 0.0510(2) -0.13949(14) 0.0229(7) Uani 1 1 d . . .
H34 H 0.3998 0.0237 -0.1395 0.028 Uiso 1 1 calc R . .
C35 C 0.4730(3) 0.1503(2) -0.14179(14) 0.0212(8) Uani 1 1 d . . .
C36 C 0.3656(3) 0.1865(2) -0.16832(16) 0.0274(8) Uani 1 1 d . . .
H36 H 0.2976 0.1499 -0.1815 0.033 Uiso 1 1 calc R . .
C37 C 0.3576(4) 0.2766(3) -0.17549(18) 0.0382(10) Uani 1 1 d . . .
H37 H 0.2842 0.3010 -0.1947 0.046 Uiso 1 1 calc R . .
C38 C 0.4503(4) 0.3302(3) -0.15613(18) 0.0399(11) Uani 1 1 d . . .
H38 H 0.4410 0.3918 -0.1605 0.048 Uiso 1 1 calc R . .
C39 C 0.5620(4) 0.2953(3) -0.12920(19) 0.0413(10) Uani 1 1 d . . .
H39 H 0.6286 0.3329 -0.1159 0.050 Uiso 1 1 calc R . .
C40 C 0.5730(4) 0.2041(3) -0.12230(18) 0.0319(9) Uani 1 1 d . . .
H40 H 0.6476 0.1792 -0.1046 0.038 Uiso 1 1 calc R . .

loop_
_atom_site_aniso_label
_atom_site_aniso_U_11
_atom_site_aniso_U_22
_atom_site_aniso_U_33
_atom_site_aniso_U_23
_atom_site_aniso_U_13
_atom_site_aniso_U_12
Zn1 0.01454(14) 0.01748(16) 0.01757(15) 0.00127(15) 0.00108(18) 0.00078(18)
N1 0.0189(14) 0.0135(14) 0.0216(14) -0.0013(11) 0.0031(11) -0.0015(11)
N2 0.0134(13) 0.0159(13) 0.0221(14) -0.0029(13) 0.0010(13) -0.0006(9)
N3 0.038(2) 0.044(2) 0.0412(19) -0.0045(17) 0.0237(17) -0.0083(17)

N4 0.0147(14) 0.0209(15) 0.0193(13) 0.0009(12) -0.0037(12) 0.0033(11)
N5 0.0166(13) 0.0209(14) 0.0156(12) 0.0037(11) 0.0002(10) 0.0012(11)
N6 0.039(2) 0.058(2) 0.043(2) -0.0071(18) 0.0067(17) 0.0195(19)
O1 0.0257(14) 0.0254(13) 0.0229(12) -0.0056(11) 0.0035(10) -0.0010(11)
O2 0.0191(13) 0.0268(14) 0.0281(14) -0.0037(12) 0.0065(11) -0.0051(10)
O3 0.0113(12) 0.0302(14) 0.0292(13) 0.0061(11) -0.0042(10) 0.0054(10)
O4 0.0227(13) 0.0404(15) 0.0174(11) -0.0001(11) 0.0016(9) 0.0064(11)
C1 0.0224(17) 0.0217(19) 0.0172(15) -0.0002(15) 0.0046(12) 0.0002(15)
C2 0.029(2) 0.0196(19) 0.0314(19) -0.0027(15) 0.0039(16) 0.0002(14)
C3 0.0164(17) 0.0187(17) 0.0228(17) -0.0015(14) -0.0031(14) 0.0104(13)
C4 0.045(2) 0.025(2) 0.0265(19) -0.0046(16) 0.0006(17) -0.0099(17)
C5 0.028(2) 0.0182(16) 0.0255(16) -0.0036(13) -0.0040(16) -0.0010(16)
C6 0.0222(18) 0.0173(15) 0.0214(14) -0.0062(12) 0.0012(16) 0.0001(15)
C7 0.0152(18) 0.026(2) 0.035(2) -0.0020(17) -0.0011(16) -0.0016(15)
C8 0.030(2) 0.025(2) 0.042(2) 0.0032(18) 0.0031(18) -0.0101(17)
C9 0.045(2) 0.0163(14) 0.0428(19) 0.0062(15) 0.005(3) -0.004(2)
C10 0.027(2) 0.026(2) 0.050(3) 0.0028(19) -0.0044(18) 0.0066(16)
C11 0.0203(19) 0.023(2) 0.040(2) 0.0011(18) 0.0038(17) -0.0003(15)
C12 0.0186(18) 0.0150(18) 0.0270(19) 0.0028(15) -0.0009(14) 0.0027(14)
C13 0.0209(17) 0.0255(18) 0.0299(19) -0.0047(17) 0.0030(16) -0.0054(13)
C14 0.0219(18) 0.0179(18) 0.0211(18) 0.0001(15) -0.0023(14) -0.0011(15)
C15 0.0149(16) 0.0182(17) 0.0262(17) -0.0011(14) 0.0004(14) -0.0026(13)
C16 0.0281(19) 0.0202(18) 0.0261(18) -0.0004(15) 0.0021(15) -0.0011(14)
C17 0.030(2) 0.0184(17) 0.042(2) 0.0033(15) 0.0113(17) 0.0001(15)
C18 0.063(3) 0.027(2) 0.0298(19) 0.0001(16) 0.0215(19) 0.0004(18)
C19 0.046(3) 0.041(2) 0.025(2) -0.0023(18) 0.0016(19) 0.0011(19)
C20 0.037(2) 0.027(2) 0.0265(19) 0.0012(16) -0.0038(17) 0.0023(16)
C21 0.0194(18) 0.0203(19) 0.0247(18) -0.0006(15) -0.0017(14) 0.0030(14)
C22 0.0190(19) 0.035(2) 0.031(2) -0.0019(17) -0.0031(16) 0.0070(17)
C23 0.0212(17) 0.0142(15) 0.0263(18) 0.0050(17) -0.0041(17) -0.0020(12)
C24 0.0180(18) 0.029(2) 0.0256(18) 0.0045(16) -0.0043(15) 0.0024(15)
C25 0.027(2) 0.0181(19) 0.0198(18) 0.0007(15) -0.0052(14) 0.0027(15)
C26 0.0152(17) 0.0235(19) 0.0242(18) 0.0052(14) -0.0049(14) 0.0017(14)
C27 0.0260(19) 0.036(2) 0.0286(18) 0.0006(17) -0.0040(15) 0.0022(16)
C28 0.049(3) 0.051(3) 0.025(2) -0.0001(19) 0.0003(18) 0.003(2)
C29 0.045(3) 0.053(3) 0.029(2) 0.0139(19) 0.0073(18) 0.012(2)
C30 0.033(2) 0.032(2) 0.046(2) 0.0147(19) 0.0008(18) -0.0040(17)
C31 0.034(2) 0.028(2) 0.0299(19) 0.0030(16) -0.0025(16) -0.0004(16)
C32 0.0160(16) 0.0230(18) 0.0200(15) 0.0028(14) 0.0014(13) -0.0049(14)
C33 0.0309(19) 0.033(2) 0.0200(15) 0.0016(16) -0.0023(14) 0.0069(17)
C34 0.0233(19) 0.0272(16) 0.0184(14) 0.0054(12) 0.0000(15) 0.0014(16)
C35 0.024(2) 0.0236(17) 0.0161(15) 0.0041(13) 0.0030(13) 0.0025(14)
C36 0.0179(19) 0.038(2) 0.0265(19) 0.0041(16) 0.0021(16) 0.0072(16)
C37 0.034(2) 0.044(3) 0.036(2) 0.0131(19) 0.0085(19) 0.021(2)

```
C38 0.065(3) 0.025(2) 0.030(2) 0.0047(17) 0.017(2) 0.011(2)
C39 0.059(3) 0.028(2) 0.037(2) 0.0018(19) 0.007(2) -0.013(2)
C40 0.030(2) 0.033(2) 0.033(2) 0.0052(18) -0.0018(17) -0.0051(17)

_geom_special_details
;
 All esds (except the esd in the dihedral angle between two l.s. planes)
 are estimated using the full covariance matrix.  The cell esds are taken
 into account individually in the estimation of esds in distances, angles
 and torsion angles; correlations between esds in cell parameters are only
 used when they are defined by crystal symmetry.  An approximate (isotropic)
 treatment of cell esds is used for estimating esds involving l.s. planes.
;

loop_
 _geom_bond_atom_site_label_1
 _geom_bond_atom_site_label_2
 _geom_bond_distance
 _geom_bond_site_symmetry_2
 _geom_bond_publ_flag
Zn1 N5 1.980(2) . ?
Zn1 N1 1.999(3) . ?
Zn1 N4 2.005(3) . ?
Zn1 N2 2.005(2) . ?
N1 C3 1.299(4) . ?
N1 C5 1.483(4) . ?
N2 C12 1.302(4) . ?
N2 C14 1.480(4) . ?
N3 C2 1.140(4) . ?
N4 C23 1.314(4) . ?
N4 C25 1.490(4) . ?
N5 C32 1.283(4) . ?
N5 C34 1.494(4) . ?
N6 C22 1.133(5) . ?
O1 C3 1.351(4) . ?
O1 C4 1.428(4) . ?
O2 C12 1.383(4) . ?
O2 C13 1.448(4) . ?
O3 C23 1.345(4) . ?
O3 C24 1.447(4) . ?
O4 C32 1.343(3) . ?
O4 C33 1.423(4) . ?
C1 C3 1.404(5) . ?
C1 C12 1.406(4) . ?
```

C1 C2 1.441(5) . ?
C4 C5 1.563(5) . ?
C4 H4A 0.9900 . ?
C4 H4B 0.9900 . ?
C5 C6 1.508(4) . ?
C5 H5 1.0000 . ?
C6 C11 1.389(5) . ?
C6 C7 1.393(5) . ?
C7 C8 1.389(5) . ?
C7 H7 0.9500 . ?
C8 C9 1.360(6) . ?
C8 H8 0.9500 . ?
C9 C10 1.404(5) . ?
C9 H9 0.9500 . ?
C10 C11 1.384(5) . ?
C10 H10 0.9500 . ?
C11 H11 0.9500 . ?
C13 C14 1.545(4) . ?
C13 H13A 0.9900 . ?
C13 H13B 0.9900 . ?
C14 C15 1.500(5) . ?
C14 H14 1.0000 . ?
C15 C16 1.368(5) . ?
C15 C20 1.396(5) . ?
C16 C17 1.409(5) . ?
C16 H16 0.9500 . ?
C17 C18 1.398(5) . ?
C17 H17 0.9500 . ?
C18 C19 1.364(6) . ?
C18 H18 0.9500 . ?
C19 C20 1.377(5) . ?
C19 H19 0.9500 . ?
C20 H20 0.9500 . ?
C21 C22 1.416(5) . ?
C21 C23 1.420(5) . ?
C21 C32 1.435(4) . ?
C24 C25 1.548(5) . ?
C24 H24A 0.9900 . ?
C24 H24B 0.9900 . ?
C25 C26 1.505(5) . ?
C25 H25 1.0000 . ?
C26 C31 1.376(5) . ?
C26 C27 1.386(5) . ?
C27 C28 1.358(5) . ?

```
C27 H27 0.9500 . ?
C28 C29 1.396(6) . ?
C28 H28 0.9500 . ?
C29 C30 1.391(6) . ?
C29 H29 0.9500 . ?
C30 C31 1.405(5) . ?
C30 H30 0.9500 . ?
C31 H31 0.9500 . ?
C33 C34 1.556(4) . ?
C33 H33A 0.9900 . ?
C33 H33B 0.9900 . ?
C34 C35 1.522(4) . ?
C34 H34 1.0000 . ?
C35 C36 1.380(5) . ?
C35 C40 1.399(5) . ?
C36 C37 1.386(5) . ?
C36 H36 0.9500 . ?
C37 C38 1.340(6) . ?
C37 H37 0.9500 . ?
C38 C39 1.413(6) . ?
C38 H38 0.9500 . ?
C39 C40 1.405(6) . ?
C39 H39 0.9500 . ?
C40 H40 0.9500 . ?

loop_
 _geom_angle_atom_site_label_1
 _geom_angle_atom_site_label_2
 _geom_angle_atom_site_label_3
 _geom_angle
 _geom_angle_site_symmetry_1
 _geom_angle_site_symmetry_3
 _geom_angle_publ_flag
N5 Zn1 N1 139.20(10) . . ?
N5 Zn1 N4 93.94(11) . . ?
N1 Zn1 N4 102.05(11) . . ?
N5 Zn1 N2 108.32(11) . . ?
N1 Zn1 N2 92.88(11) . . ?
N4 Zn1 N2 124.81(10) . . ?
C3 N1 C5 108.5(3) . . ?
C3 N1 Zn1 120.2(2) . . ?
C5 N1 Zn1 127.2(2) . . ?
C12 N2 C14 106.9(3) . . ?
C12 N2 Zn1 120.1(2) . . ?
```

C14 N2 Zn1 132.9(2) . . ?
C23 N4 C25 106.1(3) . . ?
C23 N4 Zn1 122.9(2) . . ?
C25 N4 Zn1 130.2(2) . . ?
C32 N5 C34 108.1(2) . . ?
C32 N5 Zn1 122.2(2) . . ?
C34 N5 Zn1 127.3(2) . . ?
C3 O1 C4 108.0(3) . . ?
C12 O2 C13 105.8(2) . . ?
C23 O3 C24 106.3(3) . . ?
C32 O4 C33 107.6(2) . . ?
C3 C1 C12 122.2(3) . . ?
C3 C1 C2 120.1(3) . . ?
C12 C1 C2 117.7(3) . . ?
N3 C2 C1 178.6(4) . . ?
N1 C3 O1 116.1(3) . . ?
N1 C3 C1 128.0(3) . . ?
O1 C3 C1 115.9(3) . . ?
O1 C4 C5 105.0(3) . . ?
O1 C4 H4A 110.8 . . ?
C5 C4 H4A 110.8 . . ?
O1 C4 H4B 110.8 . . ?
C5 C4 H4B 110.8 . . ?
H4A C4 H4B 108.8 . . ?
N1 C5 C6 113.3(3) . . ?
N1 C5 C4 102.3(3) . . ?
C6 C5 C4 112.1(3) . . ?
N1 C5 H5 109.6 . . ?
C6 C5 H5 109.6 . . ?
C4 C5 H5 109.6 . . ?
C11 C6 C7 119.0(3) . . ?
C11 C6 C5 121.0(3) . . ?
C7 C6 C5 119.8(3) . . ?
C8 C7 C6 119.8(4) . . ?
C8 C7 H7 120.1 . . ?
C6 C7 H7 120.1 . . ?
C9 C8 C7 121.2(4) . . ?
C9 C8 H8 119.4 . . ?
C7 C8 H8 119.4 . . ?
C8 C9 C10 119.6(3) . . ?
C8 C9 H9 120.2 . . ?
C10 C9 H9 120.2 . . ?
C11 C10 C9 119.5(4) . . ?
C11 C10 H10 120.3 . . ?

C9 C10 H10 120.3 . . ?
C10 C11 C6 120.8(3) . . ?
C10 C11 H11 119.6 . . ?
C6 C11 H11 119.6 . . ?
N2 C12 O2 115.0(3) . . ?
N2 C12 C1 128.9(3) . . ?
O2 C12 C1 116.0(3) . . ?
O2 C13 C14 102.6(2) . . ?
O2 C13 H13A 111.3 . . ?
C14 C13 H13A 111.3 . . ?
O2 C13 H13B 111.3 . . ?
C14 C13 H13B 111.3 . . ?
H13A C13 H13B 109.2 . . ?
N2 C14 C15 116.6(3) . . ?
N2 C14 C13 101.7(2) . . ?
C15 C14 C13 114.5(3) . . ?
N2 C14 H14 107.8 . . ?
C15 C14 H14 107.8 . . ?
C13 C14 H14 107.8 . . ?
C16 C15 C20 119.1(3) . . ?
C16 C15 C14 122.2(3) . . ?
C20 C15 C14 118.6(3) . . ?
C15 C16 C17 121.2(3) . . ?
C15 C16 H16 119.4 . . ?
C17 C16 H16 119.4 . . ?
C18 C17 C16 117.9(3) . . ?
C18 C17 H17 121.0 . . ?
C16 C17 H17 121.0 . . ?
C19 C18 C17 120.9(4) . . ?
C19 C18 H18 119.6 . . ?
C17 C18 H18 119.6 . . ?
C18 C19 C20 120.4(4) . . ?
C18 C19 H19 119.8 . . ?
C20 C19 H19 119.8 . . ?
C19 C20 C15 120.4(4) . . ?
C19 C20 H20 119.8 . . ?
C15 C20 H20 119.8 . . ?
C22 C21 C23 118.3(3) . . ?
C22 C21 C32 119.8(3) . . ?
C23 C21 C32 121.8(3) . . ?
N6 C22 C21 179.5(5) . . ?
N4 C23 O3 116.6(3) . . ?
N4 C23 C21 127.3(3) . . ?
O3 C23 C21 116.1(3) . . ?

O3 C24 C25 103.4(3) . . ?
O3 C24 H24A 111.1 . . ?
C25 C24 H24A 111.1 . . ?
O3 C24 H24B 111.1 . . ?
C25 C24 H24B 111.1 . . ?
H24A C24 H24B 109.1 . . ?
N4 C25 C26 115.4(3) . . ?
N4 C25 C24 101.7(3) . . ?
C26 C25 C24 113.3(3) . . ?
N4 C25 H25 108.7 . . ?
C26 C25 H25 108.7 . . ?
C24 C25 H25 108.7 . . ?
C31 C26 C27 118.8(3) . . ?
C31 C26 C25 121.2(3) . . ?
C27 C26 C25 119.8(3) . . ?
C28 C27 C26 122.2(4) . . ?
C28 C27 H27 118.9 . . ?
C26 C27 H27 118.9 . . ?
C27 C28 C29 119.5(4) . . ?
C27 C28 H28 120.3 . . ?
C29 C28 H28 120.3 . . ?
C30 C29 C28 119.6(4) . . ?
C30 C29 H29 120.2 . . ?
C28 C29 H29 120.2 . . ?
C29 C30 C31 119.4(4) . . ?
C29 C30 H30 120.3 . . ?
C31 C30 H30 120.3 . . ?
C26 C31 C30 120.3(4) . . ?
C26 C31 H31 119.8 . . ?
C30 C31 H31 119.8 . . ?
N5 C32 O4 116.9(3) . . ?
N5 C32 C21 129.1(3) . . ?
O4 C32 C21 114.0(3) . . ?
O4 C33 C34 105.1(2) . . ?
O4 C33 H33A 110.7 . . ?
C34 C33 H33A 110.7 . . ?
O4 C33 H33B 110.7 . . ?
C34 C33 H33B 110.7 . . ?
H33A C33 H33B 108.8 . . ?
N5 C34 C35 113.0(3) . . ?
N5 C34 C33 101.8(3) . . ?
C35 C34 C33 113.4(3) . . ?
N5 C34 H34 109.5 . . ?
C35 C34 H34 109.5 . . ?

```
C33 C34 H34 109.5 . . ?
C36 C35 C40 120.3(3) . . ?
C36 C35 C34 119.0(3) . . ?
C40 C35 C34 120.5(3) . . ?
C35 C36 C37 119.4(4) . . ?
C35 C36 H36 120.3 . . ?
C37 C36 H36 120.3 . . ?
C38 C37 C36 121.9(4) . . ?
C38 C37 H37 119.0 . . ?
C36 C37 H37 119.0 . . ?
C37 C38 C39 120.0(4) . . ?
C37 C38 H38 120.0 . . ?
C39 C38 H38 120.0 . . ?
C40 C39 C38 119.0(4) . . ?
C40 C39 H39 120.5 . . ?
C38 C39 H39 120.5 . . ?
C35 C40 C39 119.2(4) . . ?
C35 C40 H40 120.4 . . ?
C39 C40 H40 120.4 . . ?

loop_
 _geom_torsion_atom_site_label_1
 _geom_torsion_atom_site_label_2
 _geom_torsion_atom_site_label_3
 _geom_torsion_atom_site_label_4
 _geom_torsion
 _geom_torsion_site_symmetry_1
 _geom_torsion_site_symmetry_2
 _geom_torsion_site_symmetry_3
 _geom_torsion_site_symmetry_4
 _geom_torsion_publ_flag
N5 Zn1 N1 C3 151.2(2) . . . . ?
N4 Zn1 N1 C3 -98.0(3) . . . . ?
N2 Zn1 N1 C3 28.5(3) . . . . ?
N5 Zn1 N1 C5 -54.3(3) . . . . ?
N4 Zn1 N1 C5 56.5(3) . . . . ?
N2 Zn1 N1 C5 -177.0(2) . . . . ?
N5 Zn1 N2 C12 -168.7(2) . . . . ?
N1 Zn1 N2 C12 -24.1(2) . . . . ?
N4 Zn1 N2 C12 82.7(3) . . . . ?
N5 Zn1 N2 C14 8.0(3) . . . . ?
N1 Zn1 N2 C14 152.6(3) . . . . ?
N4 Zn1 N2 C14 -100.6(3) . . . . ?
N5 Zn1 N4 C23 -11.8(3) . . . . ?
```

N1 Zn1 N4 C23 -154.0(2) . . . . ?
N2 Zn1 N4 C23 103.8(3) . . . . ?
N5 Zn1 N4 C25 156.7(3) . . . . ?
N1 Zn1 N4 C25 14.5(3) . . . . ?
N2 Zn1 N4 C25 -87.7(3) . . . . ?
N1 Zn1 N5 C32 131.3(3) . . . . ?
N4 Zn1 N5 C32 17.7(3) . . . . ?
N2 Zn1 N5 C32 -111.0(3) . . . . ?
N1 Zn1 N5 C34 -68.4(3) . . . . ?
N4 Zn1 N5 C34 178.0(2) . . . . ?
N2 Zn1 N5 C34 49.3(3) . . . . ?
C3 C1 C2 N3 -113(17) . . . . ?
C12 C1 C2 N3 69(17) . . . . ?
C5 N1 C3 O1 2.5(4) . . . . ?
Zn1 N1 C3 O1 161.3(2) . . . . ?
C5 N1 C3 C1 -178.9(3) . . . . ?
Zn1 N1 C3 C1 -20.1(5) . . . . ?
C4 O1 C3 N1 -0.7(4) . . . . ?
C4 O1 C3 C1 -179.5(3) . . . . ?
C12 C1 C3 N1 -2.8(5) . . . . ?
C2 C1 C3 N1 179.2(3) . . . . ?
C12 C1 C3 O1 175.9(3) . . . . ?
C2 C1 C3 O1 -2.2(5) . . . . ?
C3 O1 C4 C5 -1.2(4) . . . . ?
C3 N1 C5 C6 -123.8(3) . . . . ?
Zn1 N1 C5 C6 79.3(3) . . . . ?
C3 N1 C5 C4 -2.9(3) . . . . ?
Zn1 N1 C5 C4 -159.8(2) . . . . ?
O1 C4 C5 N1 2.5(3) . . . . ?
O1 C4 C5 C6 124.2(3) . . . . ?
N1 C5 C6 C11 49.6(4) . . . . ?
C4 C5 C6 C11 -65.6(4) . . . . ?
N1 C5 C6 C7 -134.8(3) . . . . ?
C4 C5 C6 C7 110.0(4) . . . . ?
C11 C6 C7 C8 2.7(5) . . . . ?
C5 C6 C7 C8 -172.9(3) . . . . ?
C6 C7 C8 C9 -0.9(6) . . . . ?
C7 C8 C9 C10 -1.4(6) . . . . ?
C8 C9 C10 C11 1.7(6) . . . . ?
C9 C10 C11 C6 0.2(6) . . . . ?
C7 C6 C11 C10 -2.4(5) . . . . ?
C5 C6 C11 C10 173.2(3) . . . . ?
C14 N2 C12 O2 10.2(4) . . . . ?
Zn1 N2 C12 O2 -172.3(2) . . . . ?

C14 N2 C12 C1 -166.8(3) . . . . ?
Zn1 N2 C12 C1 10.6(5) . . . . ?
C13 O2 C12 N2 8.8(4) . . . . ?
C13 O2 C12 C1 -173.8(3) . . . . ?
C3 C1 C12 N2 8.3(6) . . . . ?
C2 C1 C12 N2 -173.6(3) . . . . ?
C3 C1 C12 O2 -168.8(3) . . . . ?
C2 C1 C12 O2 9.3(5) . . . . ?
C12 O2 C13 C14 -22.5(3) . . . . ?
C12 N2 C14 C15 -148.7(3) . . . . ?
Zn1 N2 C14 C15 34.4(4) . . . . ?
C12 N2 C14 C13 -23.3(3) . . . . ?
Zn1 N2 C14 C13 159.7(2) . . . . ?
O2 C13 C14 N2 27.5(3) . . . . ?
O2 C13 C14 C15 154.2(3) . . . . ?
N2 C14 C15 C16 30.6(5) . . . . ?
C13 C14 C15 C16 -88.0(4) . . . . ?
N2 C14 C15 C20 -152.1(3) . . . . ?
C13 C14 C15 C20 89.3(4) . . . . ?
C20 C15 C16 C17 2.4(5) . . . . ?
C14 C15 C16 C17 179.7(3) . . . . ?
C15 C16 C17 C18 -3.7(5) . . . . ?
C16 C17 C18 C19 3.0(5) . . . . ?
C17 C18 C19 C20 -0.9(6) . . . . ?
C18 C19 C20 C15 -0.5(6) . . . . ?
C16 C15 C20 C19 -0.2(5) . . . . ?
C14 C15 C20 C19 -177.6(3) . . . . ?
C23 C21 C22 N6 104(52) . . . . ?
C32 C21 C22 N6 -79(52) . . . . ?
C25 N4 C23 O3 9.4(4) . . . . ?
Zn1 N4 C23 O3 -179.8(2) . . . . ?
C25 N4 C23 C21 -169.3(3) . . . . ?
Zn1 N4 C23 C21 1.6(4) . . . . ?
C24 O3 C23 N4 7.1(4) . . . . ?
C24 O3 C23 C21 -174.1(3) . . . . ?
C22 C21 C23 N4 -175.2(3) . . . . ?
C32 C21 C23 N4 7.7(5) . . . . ?
C22 C21 C23 O3 6.2(4) . . . . ?
C32 C21 C23 O3 -171.0(3) . . . . ?
C23 O3 C24 C25 -19.4(3) . . . . ?
C23 N4 C25 C26 -143.2(3) . . . . ?
Zn1 N4 C25 C26 46.8(4) . . . . ?
C23 N4 C25 C24 -20.3(3) . . . . ?
Zn1 N4 C25 C24 169.8(2) . . . . ?

O3 C24 C25 N4 23.8(3) . . . . ?
O3 C24 C25 C26 148.2(3) . . . . ?
N4 C25 C26 C31 44.6(4) . . . . ?
C24 C25 C26 C31 -71.9(4) . . . . ?
N4 C25 C26 C27 -140.6(3) . . . . ?
C24 C25 C26 C27 102.8(4) . . . . ?
C31 C26 C27 C28 0.7(5) . . . . ?
C25 C26 C27 C28 -174.2(4) . . . . ?
C26 C27 C28 C29 -4.0(6) . . . . ?
C27 C28 C29 C30 4.0(6) . . . . ?
C28 C29 C30 C31 -0.7(6) . . . . ?
C27 C26 C31 C30 2.6(5) . . . . ?
C25 C26 C31 C30 177.4(3) . . . . ?
C29 C30 C31 C26 -2.6(5) . . . . ?
C34 N5 C32 O4 2.4(4) . . . . ?
Zn1 N5 C32 O4 166.0(2) . . . . ?
C34 N5 C32 C21 -178.5(3) . . . . ?
Zn1 N5 C32 C21 -14.9(5) . . . . ?
C33 O4 C32 N5 -6.0(4) . . . . ?
C33 O4 C32 C21 174.7(3) . . . . ?
C22 C21 C32 N5 -177.3(3) . . . . ?
C23 C21 C32 N5 -0.2(6) . . . . ?
C22 C21 C32 O4 1.8(5) . . . . ?
C23 C21 C32 O4 179.0(3) . . . . ?
C32 O4 C33 C34 6.6(4) . . . . ?
C32 N5 C34 C35 -120.0(3) . . . . ?
Zn1 N5 C34 C35 77.4(3) . . . . ?
C32 N5 C34 C33 1.9(3) . . . . ?
Zn1 N5 C34 C33 -160.6(2) . . . . ?
O4 C33 C34 N5 -5.1(3) . . . . ?
O4 C33 C34 C35 116.5(3) . . . . ?
N5 C34 C35 C36 -148.2(3) . . . . ?
C33 C34 C35 C36 96.6(4) . . . . ?
N5 C34 C35 C40 37.2(4) . . . . ?
C33 C34 C35 C40 -78.0(4) . . . . ?
C40 C35 C36 C37 0.0(5) . . . . ?
C34 C35 C36 C37 -174.6(3) . . . . ?
C35 C36 C37 C38 -1.5(6) . . . . ?
C36 C37 C38 C39 1.9(6) . . . . ?
C37 C38 C39 C40 -0.9(6) . . . . ?
C36 C35 C40 C39 0.9(5) . . . . ?
C34 C35 C40 C39 175.4(3) . . . . ?
C38 C39 C40 C35 -0.5(6) . . . . ?

```
_diffrn_measured_fraction_theta_max    1.000
_diffrn_reflns_theta_full              27.51
_diffrn_measured_fraction_theta_full   1.000
_refine_diff_density_max    0.389
_refine_diff_density_min   -0.538
_refine_diff_density_rms    0.066

data_compound_13

_audit_creation_method            SHELXL-97
_chemical_name_systematic
;
 ?
;
_chemical_name_common             ?
_chemical_melting_point           ?
_chemical_formula_moiety          ?
_chemical_formula_sum
 'C25 H31 Cl6 N4 O4 Zn2'
_chemical_formula_weight          794.98

loop_
 _atom_type_symbol
 _atom_type_description
 _atom_type_scat_dispersion_real
 _atom_type_scat_dispersion_imag
 _atom_type_scat_source
 'C'  'C'   0.0033   0.0016
 'International Tables Vol C Tables 4.2.6.8 and 6.1.1.4'
 'H'  'H'   0.0000   0.0000
 'International Tables Vol C Tables 4.2.6.8 and 6.1.1.4'
 'N'  'N'   0.0061   0.0033
 'International Tables Vol C Tables 4.2.6.8 and 6.1.1.4'
 'O'  'O'   0.0106   0.0060
 'International Tables Vol C Tables 4.2.6.8 and 6.1.1.4'
 'Cl'  'Cl'   0.1484   0.1585
 'International Tables Vol C Tables 4.2.6.8 and 6.1.1.4'
 'Zn'  'Zn'   0.2839   1.4301
 'International Tables Vol C Tables 4.2.6.8 and 6.1.1.4'

_symmetry_cell_setting            Orthorhombic
_symmetry_space_group_name_H-M    P2(1)2(1)2

loop_
```

```
 _symmetry_equiv_pos_as_xyz
 'x, y, z'
 '-x, -y, z'
 '-x+1/2, y+1/2, -z'
 'x+1/2, -y+1/2, -z'

_cell_length_a                    18.253(3)
_cell_length_b                    22.150(4)
_cell_length_c                    9.1847(17)
_cell_angle_alpha                 90.00
_cell_angle_beta                  90.00
_cell_angle_gamma                 90.00
_cell_volume                      3713.3(12)
_cell_formula_units_Z             4
_cell_measurement_temperature     293(2)
_cell_measurement_reflns_used     1240
_cell_measurement_theta_min       4.805
_cell_measurement_theta_max       32.805

_exptl_crystal_description        prismatic
_exptl_crystal_colour             colorless
_exptl_crystal_size_max           0.165
_exptl_crystal_size_mid           0.134
_exptl_crystal_size_min           0.058
_exptl_crystal_density_meas       ?
_exptl_crystal_density_diffrn     1.422
_exptl_crystal_density_method     'not measured'
_exptl_crystal_F_000              1612
_exptl_absorpt_coefficient_mu     1.756
_exptl_absorpt_correction_type    empirical
_exptl_absorpt_correction_T_min   0.09689
_exptl_absorpt_correction_T_max   1.00000
_exptl_absorpt_process_details    sadabs

_exptl_special_details
;
 ?
;

_diffrn_ambient_temperature       293(2)
_diffrn_radiation_wavelength      0.71073
_diffrn_radiation_type            MoK\a
_diffrn_radiation_source          'fine-focus sealed tube'
_diffrn_radiation_monochromator   graphite
```

```
_diffrn_measurement_device_type   'CCD area detector'
_diffrn_measurement_method        'phi and omega scans'
_diffrn_detector_area_resol_mean  ?
_diffrn_standards_number          ?
_diffrn_standards_interval_count  ?
_diffrn_standards_interval_time   ?
_diffrn_standards_decay_%         ?
_diffrn_reflns_number             6915
_diffrn_reflns_av_R_equivalents   0.0000
_diffrn_reflns_av_sigmaI/netI     0.1596
_diffrn_reflns_limit_h_min        -22
_diffrn_reflns_limit_h_max        22
_diffrn_reflns_limit_k_min        0
_diffrn_reflns_limit_k_max        26
_diffrn_reflns_limit_l_min        0
_diffrn_reflns_limit_l_max        11
_diffrn_reflns_theta_min          2.15
_diffrn_reflns_theta_max          25.50
_reflns_number_total              6915
_reflns_number_gt                 3276
_reflns_threshold_expression      >2sigma(I)

_computing_data_collection        'Bruker SMART'
_computing_cell_refinement        'Bruker SMART'
_computing_data_reduction         'Bruker SHELXTL'
_computing_structure_solution     'SHELXS-97 (Sheldrick, 1990)'
_computing_structure_refinement   'SHELXL-97 (Sheldrick, 1997)'
_computing_molecular_graphics     'Bruker SHELXTL'
_computing_publication_material   'Bruker SHELXTL'

_refine_special_details
;
 Refinement of F^2^ against ALL reflections.  The weighted R-factor wR and
 goodness of fit S are based on F^2^, conventional R-factors R are based
 on F, with F set to zero for negative F^2^. The threshold expression of
 F^2^ > 2sigma(F^2^) is used only for calculating R-factors(gt) etc. and is
 not relevant to the choice of reflections for refinement.  R-factors based
 on F^2^ are statistically about twice as large as those based on F, and R-
 factors based on ALL data will be even larger.
;

_refine_ls_structure_factor_coef  Fsqd
_refine_ls_matrix_type            full
_refine_ls_weighting_scheme       calc
```

```
_refine_ls_weighting_details
 'calc w=1/[\s^2^(Fo^2^)+(0.0690P)^2^+0.0000P] where P=(Fo^2^+2Fc^2^)/3'
_atom_sites_solution_primary      direct
_atom_sites_solution_secondary    difmap
_atom_sites_solution_hydrogens    geom
_refine_ls_hydrogen_treatment     constr
_refine_ls_extinction_method      none
_refine_ls_extinction_coef        ?
_refine_ls_abs_structure_details
 'Flack H D (1983), Acta Cryst. A39, 876-881'
_refine_ls_abs_structure_Flack    0.01(3)
_chemical_absolute_configuration  ad
_refine_ls_number_reflns          6915
_refine_ls_number_parameters      376
_refine_ls_number_restraints      20
_refine_ls_R_factor_all           0.1582
_refine_ls_R_factor_gt            0.0725
_refine_ls_wR_factor_ref          0.1649
_refine_ls_wR_factor_gt           0.1409
_refine_ls_goodness_of_fit_ref    0.888
_refine_ls_restrained_S_all       0.891
_refine_ls_shift/su_max           0.000
_refine_ls_shift/su_mean          0.000

# SQUEEZE RESULTS (APPEND TO CIF)
# Note: Data are Listed for all Voids in the P1 Unit Cell
# i.e. Centre of Gravity, Solvent Accessible Volume,
# Recovered number of Electrons in the Void and
# Details about the Squeezed Material
loop_
  _platon_squeeze_void_nr
  _platon_squeeze_void_average_x
  _platon_squeeze_void_average_y
  _platon_squeeze_void_average_z
  _platon_squeeze_void_volume
  _platon_squeeze_void_count_electrons
  _platon_squeeze_void_content
   1  0.000  0.000  0.486      273        48 ' '
   2  0.500  0.500  0.514      273        47 ' '
_platon_squeeze_details
;
;
```

```
loop_
 _atom_site_label
 _atom_site_type_symbol
 _atom_site_fract_x
 _atom_site_fract_y
 _atom_site_fract_z
 _atom_site_U_iso_or_equiv
 _atom_site_adp_type
 _atom_site_occupancy
 _atom_site_symmetry_multiplicity
 _atom_site_calc_flag
 _atom_site_refinement_flags
 _atom_site_disorder_assembly
 _atom_site_disorder_group
Zn1 Zn 0.88440(6) 0.30422(5) 1.02584(10) 0.0556(3) Uani 1 1 d . . .
Zn2 Zn 0.69271(6) 0.24476(5) 0.75403(12) 0.0632(3) Uani 1 1 d . . .
Cl1 Cl 0.88028(16) 0.38238(12) 1.1767(3) 0.0745(8) Uani 1 1 d . . .
Cl2 Cl 0.75395(18) 0.23320(17) 0.5508(3) 0.1069(12) Uani 1 1 d . . .
Cl3 Cl 0.61486(15) 0.32319(12) 0.7529(3) 0.0786(7) Uani 1 1 d . . .
Cl4 Cl 0.8798(3) 0.1358(2) 0.7716(7) 0.1748(19) Uani 1 1 d . . .
Cl5 Cl 0.9707(3) 0.0938(3) 0.9978(7) 0.209(2) Uani 1 1 d . . .
Cl6 Cl 0.8481(3) 0.0271(2) 0.9186(9) 0.231(3) Uani 1 1 d . . .
N1 N 0.8908(5) 0.3278(3) 0.8144(8) 0.056(2) Uani 1 1 d . . .
N2 N 1.0767(4) 0.2145(4) 0.9122(8) 0.060(2) Uani 1 1 d . . .
N3 N 0.6462(4) 0.1712(4) 0.8420(9) 0.059(2) Uani 1 1 d . . .
N4 N 0.8307(4) 0.2340(3) 1.1071(8) 0.052(2) Uani 1 1 d . . .
O1 O 0.9296(5) 0.3256(4) 0.5850(7) 0.083(2) Uani 1 1 d . . .
O2 O 0.9857(3) 0.2700(3) 1.0123(6) 0.0603(18) Uani 1 1 d . . .
O3 O 0.6311(4) 0.1004(3) 1.0125(9) 0.087(2) Uani 1 1 d . . .
O4 O 0.7612(3) 0.2566(3) 0.9207(7) 0.0635(18) Uani 1 1 d . . .
C1 C 0.9387(6) 0.3093(5) 0.7241(10) 0.061(3) Uani 1 1 d . . .
C2 C 0.8662(7) 0.3599(6) 0.5770(11) 0.091(4) Uani 1 1 d . . .
H2A H 0.8305 0.3401 0.5150 0.110 Uiso 1 1 calc R . .
H2B H 0.8769 0.3994 0.5366 0.110 Uiso 1 1 calc R . .
C3 C 0.8358(5) 0.3662(4) 0.7335(10) 0.060(3) Uani 1 1 d . . .
H3 H 0.7882 0.3459 0.7376 0.072 Uiso 1 1 calc R . .
C4 C 0.8267(7) 0.4284(5) 0.7929(14) 0.098(4) Uani 1 1 d . . .
H4 H 0.8112 0.4234 0.8943 0.117 Uiso 1 1 calc R . .
C5 C 0.8987(7) 0.4642(5) 0.7983(16) 0.117(5) Uani 1 1 d . . .
H5A H 0.9347 0.4416 0.8517 0.175 Uiso 1 1 calc R . .
H5B H 0.8904 0.5022 0.8457 0.175 Uiso 1 1 calc R . .
H5C H 0.9159 0.4711 0.7010 0.175 Uiso 1 1 calc R . .
C6 C 0.7631(7) 0.4607(6) 0.7151(17) 0.124(5) Uani 1 1 d . . .
```

H6A H 0.7785 0.4729 0.6196 0.186 Uiso 1 1 calc R . .
H6B H 0.7490 0.4957 0.7701 0.186 Uiso 1 1 calc R . .
H6C H 0.7221 0.4338 0.7071 0.186 Uiso 1 1 calc R . .
C7 C 0.9998(5) 0.2706(4) 0.7498(11) 0.057(2) Uani 1 1 d . . .
C8 C 1.0395(6) 0.2461(6) 0.6388(10) 0.076(3) Uani 1 1 d . . .
H8 H 1.0257 0.2551 0.5437 0.091 Uiso 1 1 calc R . .
C9 C 1.0979(7) 0.2092(6) 0.6596(13) 0.090(4) Uani 1 1 d . . .
H9 H 1.1256 0.1962 0.5806 0.108 Uiso 1 1 calc R . .
C10 C 1.1162(7) 0.1907(5) 0.8015(13) 0.077(3) Uani 1 1 d D . .
C11 C 1.0181(5) 0.2534(5) 0.8977(10) 0.060(3) Uani 1 1 d . . .
C12 C 1.1729(6) 0.1474(5) 0.8402(14) 0.088(4) Uani 1 1 d D . .
H12A H 1.1945 0.1587 0.9315 0.132 Uiso 1 1 calc R . .
H12B H 1.2100 0.1471 0.7660 0.132 Uiso 1 1 calc R . .
H12C H 1.1518 0.1079 0.8485 0.132 Uiso 1 1 calc R . .
C13 C 0.6667(6) 0.1499(5) 0.9626(14) 0.068(3) Uani 1 1 d . . .
C14 C 0.5753(8) 0.0886(6) 0.8987(15) 0.111(3) Uani 1 1 d U . .
H14A H 0.5798 0.0476 0.8627 0.133 Uiso 1 1 calc R . .
H14B H 0.5264 0.0939 0.9380 0.133 Uiso 1 1 calc R . .
C15 C 0.5886(7) 0.1322(6) 0.7797(16) 0.105(3) Uani 1 1 d U . .
H15 H 0.5442 0.1563 0.7641 0.126 Uiso 1 1 calc R . .
C16 C 0.6144(8) 0.1066(6) 0.6334(16) 0.112(3) Uani 1 1 d U . .
H16 H 0.6270 0.1401 0.5683 0.134 Uiso 1 1 calc R . .
C17 C 0.5545(7) 0.0723(6) 0.5694(17) 0.133(4) Uani 1 1 d U . .
H17A H 0.5431 0.0384 0.6307 0.200 Uiso 1 1 calc R . .
H17B H 0.5687 0.0581 0.4748 0.200 Uiso 1 1 calc R . .
H17C H 0.5121 0.0977 0.5606 0.200 Uiso 1 1 calc R . .
C18 C 0.6821(8) 0.0667(7) 0.6554(17) 0.148(7) Uani 1 1 d . . .
H18A H 0.6673 0.0283 0.6939 0.221 Uiso 1 1 calc R . .
H18B H 0.7150 0.0860 0.7225 0.221 Uiso 1 1 calc R . .
H18C H 0.7063 0.0609 0.5638 0.221 Uiso 1 1 calc R . .
C19 C 0.7273(6) 0.1686(5) 1.0604(11) 0.063(3) Uani 1 1 d . . .
C20 C 0.7465(7) 0.1361(5) 1.1846(14) 0.084(4) Uani 1 1 d . . .
H20 H 0.7182 0.1030 1.2116 0.100 Uiso 1 1 calc R . .
C21 C 0.8044(7) 0.1513(5) 1.2661(11) 0.075(3) Uani 1 1 d . . .
H21 H 0.8156 0.1289 1.3488 0.090 Uiso 1 1 calc R . .
C22 C 0.8471(5) 0.1994(5) 1.2287(11) 0.059(3) Uani 1 1 d D . .
C23 C 0.7714(5) 0.2183(4) 1.0258(11) 0.051(2) Uani 1 1 d . . .
C24 C 0.9128(5) 0.2182(5) 1.3120(10) 0.079(3) Uani 1 1 d D . .
H24A H 0.9121 0.1995 1.4063 0.119 Uiso 1 1 calc R . .
H24B H 0.9127 0.2613 1.3230 0.119 Uiso 1 1 calc R . .
H24C H 0.9561 0.2060 1.2606 0.119 Uiso 1 1 calc R . .
C25 C 0.9138(10) 0.0739(7) 0.862(2) 0.162(8) Uani 1 1 d . . .
H25 H 0.9433 0.0514 0.7913 0.194 Uiso 1 1 calc R . .

```
loop_
 _atom_site_aniso_label
 _atom_site_aniso_U_11
 _atom_site_aniso_U_22
 _atom_site_aniso_U_33
 _atom_site_aniso_U_23
 _atom_site_aniso_U_13
 _atom_site_aniso_U_12
Zn1 0.0628(6) 0.0713(7) 0.0329(5) -0.0058(6) 0.0024(6) -0.0013(6)
Zn2 0.0621(6) 0.0822(8) 0.0455(6) -0.0066(7) 0.0006(6) -0.0070(7)
Cl1 0.0748(17) 0.092(2) 0.0572(14) -0.0248(14) 0.0011(16) 0.0004(17)
Cl2 0.105(2) 0.147(3) 0.0686(19) -0.035(2) 0.0295(18) -0.016(2)
Cl3 0.0750(16) 0.0945(19) 0.0665(14) 0.0161(16) -0.001(2) 0.0094(16)
Cl4 0.175(4) 0.153(4) 0.196(5) -0.002(4) 0.039(5) 0.000(4)
Cl5 0.236(6) 0.194(5) 0.198(6) 0.007(5) -0.029(6) -0.055(4)
Cl6 0.201(5) 0.157(5) 0.335(9) -0.007(5) 0.044(6) -0.039(4)
N1 0.073(6) 0.054(5) 0.040(4) 0.001(4) -0.006(5) -0.021(5)
N2 0.050(5) 0.083(7) 0.047(4) -0.010(5) -0.006(4) -0.002(5)
N3 0.046(5) 0.081(7) 0.050(5) -0.003(5) -0.010(4) 0.001(4)
N4 0.056(5) 0.059(5) 0.041(4) -0.010(4) -0.001(4) -0.007(4)
O1 0.106(6) 0.107(7) 0.036(4) 0.011(4) 0.003(4) 0.027(5)
O2 0.066(4) 0.091(5) 0.024(3) -0.001(3) -0.001(3) 0.011(4)
O3 0.090(5) 0.064(4) 0.106(6) 0.009(4) -0.021(5) -0.036(4)
O4 0.072(4) 0.066(5) 0.053(4) 0.005(4) -0.017(3) -0.016(4)
C1 0.078(8) 0.069(7) 0.035(5) 0.003(6) -0.003(5) -0.029(6)
C2 0.116(11) 0.117(10) 0.041(6) 0.013(7) -0.003(7) -0.008(9)
C3 0.063(6) 0.070(7) 0.048(6) 0.004(6) -0.008(6) -0.002(5)
C4 0.146(12) 0.065(8) 0.083(9) 0.010(7) -0.044(9) -0.010(8)
C5 0.147(13) 0.067(8) 0.137(12) 0.004(8) -0.026(11) -0.015(8)
C6 0.135(11) 0.090(9) 0.148(14) 0.032(10) 0.005(12) 0.034(8)
C7 0.068(6) 0.052(6) 0.050(5) 0.000(6) 0.011(6) -0.002(5)
C8 0.079(8) 0.116(10) 0.032(5) -0.005(7) 0.019(6) 0.003(8)
C9 0.092(10) 0.103(11) 0.074(8) -0.023(7) 0.031(8) 0.003(8)
C10 0.064(7) 0.084(8) 0.085(8) -0.038(7) -0.002(7) -0.002(7)
C11 0.051(6) 0.078(8) 0.051(6) -0.008(6) -0.009(5) -0.008(6)
C12 0.058(7) 0.102(10) 0.103(9) -0.044(8) 0.000(7) -0.011(7)
C13 0.061(7) 0.049(7) 0.093(9) -0.018(7) 0.027(7) -0.004(5)
C14 0.108(7) 0.093(7) 0.130(8) -0.027(6) -0.027(7) -0.017(6)
C15 0.101(6) 0.089(7) 0.123(7) -0.031(5) -0.028(6) -0.011(5)
C16 0.107(6) 0.100(7) 0.129(7) -0.038(6) -0.032(7) -0.003(6)
C17 0.130(9) 0.115(9) 0.155(10) -0.048(8) -0.035(8) 0.012(7)
C18 0.121(12) 0.170(15) 0.152(14) -0.105(13) -0.032(11) 0.031(11)
C19 0.078(7) 0.060(7) 0.049(6) -0.001(6) -0.004(6) -0.007(6)
C20 0.110(10) 0.055(7) 0.086(9) -0.001(7) 0.037(8) 0.002(7)
```

C21 0.109(9) 0.079(8) 0.037(6) -0.002(6) -0.025(7) -0.016(7)
C22 0.066(6) 0.058(6) 0.053(6) -0.021(6) 0.001(6) 0.001(6)
C23 0.041(5) 0.061(7) 0.051(6) -0.003(6) 0.001(6) -0.005(5)
C24 0.074(7) 0.118(10) 0.045(5) -0.014(6) -0.001(6) 0.000(7)
C25 0.193(18) 0.084(12) 0.21(2) -0.004(12) 0.003(16) -0.059(12)

_geom_special_details
;
 All esds (except the esd in the dihedral angle between two l.s. planes)
 are estimated using the full covariance matrix. The cell esds are taken
 into account individually in the estimation of esds in distances, angles
 and torsion angles; correlations between esds in cell parameters are only
 used when they are defined by crystal symmetry. An approximate (isotropic)
 treatment of cell esds is used for estimating esds involving l.s. planes.
;

loop_
 _geom_bond_atom_site_label_1
 _geom_bond_atom_site_label_2
 _geom_bond_distance
 _geom_bond_site_symmetry_2
 _geom_bond_publ_flag
Zn1 N4 1.984(7) . ?
Zn1 O2 2.003(6) . ?
Zn1 N1 2.015(7) . ?
Zn1 Cl1 2.219(3) . ?
Zn2 O4 1.994(6) . ?
Zn2 N3 2.006(9) . ?
Zn2 Cl2 2.191(3) . ?
Zn2 Cl3 2.244(3) . ?
Cl4 C25 1.717(18) . ?
Cl5 C25 1.683(18) . ?
Cl6 C25 1.668(15) . ?
N1 C1 1.273(11) . ?
N1 C3 1.511(11) . ?
N2 C10 1.353(12) . ?
N2 C11 1.380(12) . ?
N3 C13 1.262(14) . ?
N3 C15 1.477(13) . ?
N4 C23 1.360(11) . ?
N4 C22 1.388(11) . ?
O1 C1 1.338(10) . ?
O1 C2 1.387(12) . ?
O2 C11 1.262(10) . ?

O3 C13 1.355(11) . ?
O3 C14 1.483(14) . ?
O4 C23 1.299(10) . ?
C1 C7 1.425(13) . ?
C2 C3 1.547(14) . ?
C2 H2A 0.9700 . ?
C2 H2B 0.9700 . ?
C3 C4 1.490(14) . ?
C3 H3 0.9800 . ?
C4 C5 1.535(15) . ?
C4 C6 1.540(15) . ?
C4 H4 0.9800 . ?
C5 H5A 0.9600 . ?
C5 H5B 0.9600 . ?
C5 H5C 0.9600 . ?
C6 H6A 0.9600 . ?
C6 H6B 0.9600 . ?
C6 H6C 0.9600 . ?
C7 C8 1.364(12) . ?
C7 C11 1.450(13) . ?
C8 C9 1.357(15) . ?
C8 H8 0.9300 . ?
C9 C10 1.406(15) . ?
C9 H9 0.9300 . ?
C10 C12 1.456(12) . ?
C12 H12A 0.9600 . ?
C12 H12B 0.9600 . ?
C12 H12C 0.9600 . ?
C13 C19 1.484(14) . ?
C14 C15 1.479(17) . ?
C14 H14A 0.9700 . ?
C14 H14B 0.9700 . ?
C15 C16 1.533(18) . ?
C15 H15 0.9800 . ?
C16 C17 1.455(16) . ?
C16 C18 1.533(17) . ?
C16 H16 0.9800 . ?
C17 H17A 0.9600 . ?
C17 H17B 0.9600 . ?
C17 H17C 0.9600 . ?
C18 H18A 0.9600 . ?
C18 H18B 0.9600 . ?
C18 H18C 0.9600 . ?
C19 C20 1.394(14) . ?

```
C19 C23 1.400(13) . ?
C20 C21 1.338(14) . ?
C20 H20 0.9300 . ?
C21 C22 1.365(13) . ?
C21 H21 0.9300 . ?
C22 C24 1.482(10) . ?
C24 H24A 0.9600 . ?
C24 H24B 0.9600 . ?
C24 H24C 0.9600 . ?
C25 H25 0.9800 . ?

loop_
 _geom_angle_atom_site_label_1
 _geom_angle_atom_site_label_2
 _geom_angle_atom_site_label_3
 _geom_angle
 _geom_angle_site_symmetry_1
 _geom_angle_site_symmetry_3
 _geom_angle_publ_flag
N4 Zn1 O2 100.5(3) . . ?
N4 Zn1 N1 126.5(3) . . ?
O2 Zn1 N1 89.1(3) . . ?
N4 Zn1 Cl1 111.1(2) . . ?
O2 Zn1 Cl1 111.45(19) . . ?
N1 Zn1 Cl1 113.7(2) . . ?
O4 Zn2 N3 93.7(3) . . ?
O4 Zn2 Cl2 110.4(2) . . ?
N3 Zn2 Cl2 117.7(2) . . ?
O4 Zn2 Cl3 107.4(2) . . ?
N3 Zn2 Cl3 111.2(2) . . ?
Cl2 Zn2 Cl3 114.19(13) . . ?
C1 N1 C3 108.5(8) . . ?
C1 N1 Zn1 125.8(7) . . ?
C3 N1 Zn1 125.5(6) . . ?
C10 N2 C11 125.7(9) . . ?
C13 N3 C15 109.4(10) . . ?
C13 N3 Zn2 122.1(7) . . ?
C15 N3 Zn2 128.4(8) . . ?
C23 N4 C22 118.2(8) . . ?
C23 N4 Zn1 112.7(6) . . ?
C22 N4 Zn1 129.0(6) . . ?
C1 O1 C2 107.6(9) . . ?
C11 O2 Zn1 126.5(6) . . ?
C13 O3 C14 103.5(9) . . ?
```

C23 O4 Zn2 125.0(6) . . ?
N1 C1 O1 116.7(10) . . ?
N1 C1 C7 128.5(9) . . ?
O1 C1 C7 114.7(10) . . ?
O1 C2 C3 107.5(8) . . ?
O1 C2 H2A 110.2 . . ?
C3 C2 H2A 110.2 . . ?
O1 C2 H2B 110.2 . . ?
C3 C2 H2B 110.2 . . ?
H2A C2 H2B 108.5 . . ?
C4 C3 N1 114.6(8) . . ?
C4 C3 C2 117.6(9) . . ?
N1 C3 C2 99.6(8) . . ?
C4 C3 H3 108.1 . . ?
N1 C3 H3 108.1 . . ?
C2 C3 H3 108.1 . . ?
C3 C4 C5 113.2(11) . . ?
C3 C4 C6 110.1(10) . . ?
C5 C4 C6 114.8(10) . . ?
C3 C4 H4 106.0 . . ?
C5 C4 H4 106.0 . . ?
C6 C4 H4 106.0 . . ?
C4 C5 H5A 109.5 . . ?
C4 C5 H5B 109.5 . . ?
H5A C5 H5B 109.5 . . ?
C4 C5 H5C 109.5 . . ?
H5A C5 H5C 109.5 . . ?
H5B C5 H5C 109.5 . . ?
C4 C6 H6A 109.5 . . ?
C4 C6 H6B 109.5 . . ?
H6A C6 H6B 109.5 . . ?
C4 C6 H6C 109.5 . . ?
H6A C6 H6C 109.5 . . ?
H6B C6 H6C 109.5 . . ?
C8 C7 C1 122.1(10) . . ?
C8 C7 C11 118.2(9) . . ?
C1 C7 C11 119.6(9) . . ?
C9 C8 C7 123.5(10) . . ?
C9 C8 H8 118.2 . . ?
C7 C8 H8 118.2 . . ?
C8 C9 C10 119.5(11) . . ?
C8 C9 H9 120.2 . . ?
C10 C9 H9 120.2 . . ?
N2 C10 C9 117.2(10) . . ?

N2 C10 C12 116.9(11) . . ?
C9 C10 C12 126.0(11) . . ?
O2 C11 N2 117.7(9) . . ?
O2 C11 C7 126.7(10) . . ?
N2 C11 C7 115.7(9) . . ?
C10 C12 H12A 109.5 . . ?
C10 C12 H12B 109.5 . . ?
H12A C12 H12B 109.5 . . ?
C10 C12 H12C 109.5 . . ?
H12A C12 H12C 109.5 . . ?
H12B C12 H12C 109.5 . . ?
N3 C13 O3 117.2(11) . . ?
N3 C13 C19 130.4(10) . . ?
O3 C13 C19 112.3(11) . . ?
C15 C14 O3 107.1(10) . . ?
C15 C14 H14A 110.3 . . ?
O3 C14 H14A 110.3 . . ?
C15 C14 H14B 110.3 . . ?
O3 C14 H14B 110.3 . . ?
H14A C14 H14B 108.6 . . ?
N3 C15 C14 102.3(10) . . ?
N3 C15 C16 109.7(11) . . ?
C14 C15 C16 117.1(12) . . ?
N3 C15 H15 109.1 . . ?
C14 C15 H15 109.1 . . ?
C16 C15 H15 109.1 . . ?
C17 C16 C18 111.0(11) . . ?
C17 C16 C15 108.4(12) . . ?
C18 C16 C15 110.2(12) . . ?
C17 C16 H16 109.1 . . ?
C18 C16 H16 109.1 . . ?
C15 C16 H16 109.1 . . ?
C16 C17 H17A 109.5 . . ?
C16 C17 H17B 109.5 . . ?
H17A C17 H17B 109.5 . . ?
C16 C17 H17C 109.5 . . ?
H17A C17 H17C 109.5 . . ?
H17B C17 H17C 109.5 . . ?
C16 C18 H18A 109.5 . . ?
C16 C18 H18B 109.5 . . ?
H18A C18 H18B 109.5 . . ?
C16 C18 H18C 109.5 . . ?
H18A C18 H18C 109.5 . . ?
H18B C18 H18C 109.5 . . ?

C20 C19 C23 116.6(11) . . ?
C20 C19 C13 122.5(11) . . ?
C23 C19 C13 120.7(10) . . ?
C21 C20 C19 121.7(11) . . ?
C21 C20 H20 119.2 . . ?
C19 C20 H20 119.2 . . ?
C20 C21 C22 120.5(10) . . ?
C20 C21 H21 119.7 . . ?
C22 C21 H21 119.7 . . ?
C21 C22 N4 120.7(9) . . ?
C21 C22 C24 123.6(10) . . ?
N4 C22 C24 115.7(9) . . ?
O4 C23 N4 110.8(8) . . ?
O4 C23 C19 126.9(9) . . ?
N4 C23 C19 122.2(10) . . ?
C22 C24 H24A 109.5 . . ?
C22 C24 H24B 109.5 . . ?
H24A C24 H24B 109.5 . . ?
C22 C24 H24C 109.5 . . ?
H24A C24 H24C 109.5 . . ?
H24B C24 H24C 109.5 . . ?
Cl6 C25 Cl5 112.0(12) . . ?
Cl6 C25 Cl4 112.8(12) . . ?
Cl5 C25 Cl4 111.9(9) . . ?
Cl6 C25 H25 106.6 . . ?
Cl5 C25 H25 106.6 . . ?
Cl4 C25 H25 106.6 . . ?

```
loop_
 _geom_torsion_atom_site_label_1
 _geom_torsion_atom_site_label_2
 _geom_torsion_atom_site_label_3
 _geom_torsion_atom_site_label_4
 _geom_torsion
 _geom_torsion_site_symmetry_1
 _geom_torsion_site_symmetry_2
 _geom_torsion_site_symmetry_3
 _geom_torsion_site_symmetry_4
 _geom_torsion_publ_flag
N4 Zn1 N1 C1 -88.7(9) . . . . ?
O2 Zn1 N1 C1 13.8(8) . . . . ?
Cl1 Zn1 N1 C1 126.9(7) . . . . ?
N4 Zn1 N1 C3 85.9(7) . . . . ?
O2 Zn1 N1 C3 -171.6(7) . . . . ?
```

Cl1 Zn1 N1 C3 -58.5(7) . . . . ?

O4 Zn2 N3 C13 -1.3(8) . . . . ?

Cl2 Zn2 N3 C13 114.1(8) . . . . ?

Cl3 Zn2 N3 C13 -111.5(8) . . . . ?

O4 Zn2 N3 C15 -177.7(9) . . . . ?

Cl2 Zn2 N3 C15 -62.3(9) . . . . ?

Cl3 Zn2 N3 C15 72.1(9) . . . . ?

O2 Zn1 N4 C23 -126.0(6) . . . . ?

N1 Zn1 N4 C23 -29.3(8) . . . . ?

Cl1 Zn1 N4 C23 115.9(6) . . . . ?

O2 Zn1 N4 C22 51.7(7) . . . . ?

N1 Zn1 N4 C22 148.4(7) . . . . ?

Cl1 Zn1 N4 C22 -66.4(7) . . . . ?

N4 Zn1 O2 C11 105.4(9) . . . . ?

N1 Zn1 O2 C11 -21.6(9) . . . . ?

Cl1 Zn1 O2 C11 -136.8(8) . . . . ?

N3 Zn2 O4 C23 9.7(7) . . . . ?

Cl2 Zn2 O4 C23 -111.7(7) . . . . ?

Cl3 Zn2 O4 C23 123.2(7) . . . . ?

C3 N1 C1 O1 -1.6(12) . . . . ?

Zn1 N1 C1 O1 173.8(6) . . . . ?

C3 N1 C1 C7 -178.3(9) . . . . ?

Zn1 N1 C1 C7 -2.9(14) . . . . ?

C2 O1 C1 N1 -0.5(13) . . . . ?

C2 O1 C1 C7 176.6(9) . . . . ?

C1 O1 C2 C3 2.3(12) . . . . ?

C1 N1 C3 C4 -123.8(10) . . . . ?

Zn1 N1 C3 C4 60.8(11) . . . . ?

C1 N1 C3 C2 2.7(10) . . . . ?

Zn1 N1 C3 C2 -172.7(7) . . . . ?

O1 C2 C3 C4 121.4(11) . . . . ?

O1 C2 C3 N1 -2.9(11) . . . . ?

N1 C3 C4 C5 56.9(13) . . . . ?

C2 C3 C4 C5 -59.6(13) . . . . ?

N1 C3 C4 C6 -173.0(9) . . . . ?

C2 C3 C4 C6 70.5(13) . . . . ?

N1 C1 C7 C8 168.4(10) . . . . ?

O1 C1 C7 C8 -8.3(14) . . . . ?

N1 C1 C7 C11 -7.6(15) . . . . ?

O1 C1 C7 C11 175.7(9) . . . . ?

C1 C7 C8 C9 179.9(11) . . . . ?

C11 C7 C8 C9 -4.0(16) . . . . ?

C7 C8 C9 C10 5.3(19) . . . . ?

C11 N2 C10 C9 2.8(17) . . . . ?

C11 N2 C10 C12 -176.2(10) . . . . ?
C8 C9 C10 N2 -4.5(18) . . . . ?
C8 C9 C10 C12 174.4(11) . . . . ?
Zn1 O2 C11 N2 -160.2(6) . . . . ?
Zn1 O2 C11 C7 19.1(15) . . . . ?
C10 N2 C11 O2 177.8(10) . . . . ?
C10 N2 C11 C7 -1.6(15) . . . . ?
C8 C7 C11 O2 -177.3(11) . . . . ?
C1 C7 C11 O2 -1.2(16) . . . . ?
C8 C7 C11 N2 2.0(13) . . . . ?
C1 C7 C11 N2 178.1(8) . . . . ?
C15 N3 C13 O3 -3.6(12) . . . . ?
Zn2 N3 C13 O3 179.4(6) . . . . ?
C15 N3 C13 C19 173.4(10) . . . . ?
Zn2 N3 C13 C19 -3.6(15) . . . . ?
C14 O3 C13 N3 -0.8(12) . . . . ?
C14 O3 C13 C19 -178.3(9) . . . . ?
C13 O3 C14 C15 4.8(13) . . . . ?
C13 N3 C15 C14 6.2(13) . . . . ?
Zn2 N3 C15 C14 -177.0(8) . . . . ?
C13 N3 C15 C16 -118.8(11) . . . . ?
Zn2 N3 C15 C16 57.9(13) . . . . ?
O3 C14 C15 N3 -6.5(14) . . . . ?
O3 C14 C15 C16 113.5(12) . . . . ?
N3 C15 C16 C17 -175.6(11) . . . . ?
C14 C15 C16 C17 68.4(16) . . . . ?
N3 C15 C16 C18 62.7(15) . . . . ?
C14 C15 C16 C18 -53.2(17) . . . . ?
N3 C13 C19 C20 -174.0(10) . . . . ?
O3 C13 C19 C20 3.1(14) . . . . ?
N3 C13 C19 C23 1.8(17) . . . . ?
O3 C13 C19 C23 178.9(8) . . . . ?
C23 C19 C20 C21 -0.5(15) . . . . ?
C13 C19 C20 C21 175.4(10) . . . . ?
C19 C20 C21 C22 -0.5(17) . . . . ?
C20 C21 C22 N4 1.1(16) . . . . ?
C20 C21 C22 C24 -178.9(9) . . . . ?
C23 N4 C22 C21 -0.6(13) . . . . ?
Zn1 N4 C22 C21 -178.2(7) . . . . ?
C23 N4 C22 C24 179.3(8) . . . . ?
Zn1 N4 C22 C24 1.7(11) . . . . ?
Zn2 O4 C23 N4 168.6(5) . . . . ?
Zn2 O4 C23 C19 -14.3(13) . . . . ?
C22 N4 C23 O4 176.8(7) . . . . ?

```
Zn1 N4 C23 O4 -5.2(9) . . . . ?
C22 N4 C23 C19 -0.4(13) . . . . ?
Zn1 N4 C23 C19 177.5(7) . . . . ?
C20 C19 C23 O4 -175.8(9) . . . . ?
C13 C19 C23 O4 8.2(15) . . . . ?
C20 C19 C23 N4 1.0(14) . . . . ?
C13 C19 C23 N4 -175.0(8) . . . . ?

_diffrn_measured_fraction_theta_max    0.999
_diffrn_reflns_theta_full              25.50
_diffrn_measured_fraction_theta_full   0.999
_refine_diff_density_max    0.405
_refine_diff_density_min   -0.436
_refine_diff_density_rms    0.080

data_compound_14

_audit_creation_method            SHELXL-97
_chemical_name_systematic
;
 ?
;
_chemical_name_common             ?
_chemical_melting_point           ?
_chemical_formula_moiety          ?
_chemical_formula_sum
 'C26 H34 Cl2 N4 O4 Zn2'
_chemical_formula_weight          668.21

loop_
 _atom_type_symbol
 _atom_type_description
 _atom_type_scat_dispersion_real
 _atom_type_scat_dispersion_imag
 _atom_type_scat_source
 'C'  'C'   0.0033   0.0016
 'International Tables Vol C Tables 4.2.6.8 and 6.1.1.4'
 'H'  'H'   0.0000   0.0000
 'International Tables Vol C Tables 4.2.6.8 and 6.1.1.4'
 'N'  'N'   0.0061   0.0033
 'International Tables Vol C Tables 4.2.6.8 and 6.1.1.4'
 'O'  'O'   0.0106   0.0060
 'International Tables Vol C Tables 4.2.6.8 and 6.1.1.4'
 'Cl'  'Cl'   0.1484   0.1585
```

```
 'International Tables Vol C Tables 4.2.6.8 and 6.1.1.4'
 'Zn'  'Zn'   0.2839   1.4301
 'International Tables Vol C Tables 4.2.6.8 and 6.1.1.4'

_symmetry_cell_setting            Monoclinic
_symmetry_space_group_name_H-M    C2

loop_
 _symmetry_equiv_pos_as_xyz
 'x, y, z'
 '-x, y, -z'
 'x+1/2, y+1/2, z'
 '-x+1/2, y+1/2, -z'

_cell_length_a                    18.656(13)
_cell_length_b                    16.055(11)
_cell_length_c                    23.275(16)
_cell_angle_alpha                 90.00
_cell_angle_beta                  105.774(16)
_cell_angle_gamma                 90.00
_cell_volume                      6709(8)
_cell_formula_units_Z             8
_cell_measurement_temperature     293(2)
_cell_measurement_reflns_used     3354
_cell_measurement_theta_min       4.508
_cell_measurement_theta_max       51.392

_exptl_crystal_description        prismatic
_exptl_crystal_colour             colorless
_exptl_crystal_size_max           0.212
_exptl_crystal_size_mid           0.145
_exptl_crystal_size_min           0.113
_exptl_crystal_density_meas       ?
_exptl_crystal_density_diffrn     1.323
_exptl_crystal_density_method     'not measured'
_exptl_crystal_F_000              2752
_exptl_absorpt_coefficient_mu     1.622
_exptl_absorpt_correction_type    empirical
_exptl_absorpt_correction_T_min   0.42919
_exptl_absorpt_correction_T_max   1.00000
_exptl_absorpt_process_details    sadabs

_exptl_special_details
;
```

```
 ?
;

_diffrn_ambient_temperature       293(2)
_diffrn_radiation_wavelength      0.71073
_diffrn_radiation_type            MoK\a
_diffrn_radiation_source          'fine-focus sealed tube'
_diffrn_radiation_monochromator   graphite
_diffrn_measurement_device_type   'CCD area detector'
_diffrn_measurement_method        'phi and omega scans'
_diffrn_detector_area_resol_mean  ?
_diffrn_standards_number          ?
_diffrn_standards_interval_count  ?
_diffrn_standards_interval_time   ?
_diffrn_standards_decay_%         ?
_diffrn_reflns_number             9654
_diffrn_reflns_av_R_equivalents   0.0000
_diffrn_reflns_av_sigmaI/netI     0.0595
_diffrn_reflns_limit_h_min        -22
_diffrn_reflns_limit_h_max        22
_diffrn_reflns_limit_k_min        -11
_diffrn_reflns_limit_k_max        19
_diffrn_reflns_limit_l_min        0
_diffrn_reflns_limit_l_max        28
_diffrn_reflns_theta_min          0.91
_diffrn_reflns_theta_max          26.00
_reflns_number_total              9654
_reflns_number_gt                 5951
_reflns_threshold_expression      >2sigma(I)

_computing_data_collection        'Bruker SMART'
_computing_cell_refinement        'Bruker SMART'
_computing_data_reduction         'Bruker SHELXTL'
_computing_structure_solution     'SHELXS-97 (Sheldrick, 1990)'
_computing_structure_refinement   'SHELXL-97 (Sheldrick, 1997)'
_computing_molecular_graphics     'Bruker SHELXTL'
_computing_publication_material   'Bruker SHELXTL'

_refine_special_details
;
 Refinement of F^2^ against ALL reflections.  The weighted R-factor wR and
 goodness of fit S are based on F^2^, conventional R-factors R are based
 on F, with F set to zero for negative F^2^. The threshold expression of
 F^2^ > 2sigma(F^2^) is used only for calculating R-factors(gt) etc. and is
```

```
 not relevant to the choice of reflections for refinement.  R-factors based
 on F^2^ are statistically about twice as large as those based on F, and R-
 factors based on ALL data will be even larger.
;

_refine_ls_structure_factor_coef  Fsqd
_refine_ls_matrix_type            full
_refine_ls_weighting_scheme       calc
_refine_ls_weighting_details
 'calc w=1/[\s^2^(Fo^2^)+(0.0924P)^2^+0.0000P] where P=(Fo^2^+2Fc^2^)/3'
_atom_sites_solution_primary      direct
_atom_sites_solution_secondary    difmap
_atom_sites_solution_hydrogens    geom
_refine_ls_hydrogen_treatment     constr
_refine_ls_extinction_method      none
_refine_ls_extinction_coef        ?
_refine_ls_abs_structure_details
 'Flack H D (1983), Acta Cryst. A39, 876-881'
_refine_ls_abs_structure_Flack    0.078(18)
_chemical_absolute_configuration  ad
_refine_ls_number_reflns          9654
_refine_ls_number_parameters      836
_refine_ls_number_restraints      853
_refine_ls_R_factor_all           0.0833
_refine_ls_R_factor_gt            0.0521
_refine_ls_wR_factor_ref          0.1561
_refine_ls_wR_factor_gt           0.1432
_refine_ls_goodness_of_fit_ref    0.952
_refine_ls_restrained_S_all       0.957
_refine_ls_shift/su_max           0.039
_refine_ls_shift/su_mean          0.005

# SQUEEZE RESULTS (APPEND TO CIF)
# Note: Data are Listed for all Voids in the P1 Unit Cell
# i.e. Centre of Gravity, Solvent Accessible Volume,
# Recovered number of Electrons in the Void and
# Details about the Squeezed Material
loop_
  _platon_squeeze_void_nr
  _platon_squeeze_void_average_x
  _platon_squeeze_void_average_y
  _platon_squeeze_void_average_z
  _platon_squeeze_void_volume
  _platon_squeeze_void_count_electrons
```

```
  _platon_squeeze_void_content
   1  0.235 -0.118  0.701      2124       373 ' '
_platon_squeeze_details
;
;



loop_
 _atom_site_label
 _atom_site_type_symbol
 _atom_site_fract_x
 _atom_site_fract_y
 _atom_site_fract_z
 _atom_site_U_iso_or_equiv
 _atom_site_adp_type
 _atom_site_occupancy
 _atom_site_symmetry_multiplicity
 _atom_site_calc_flag
 _atom_site_refinement_flags
 _atom_site_disorder_assembly
 _atom_site_disorder_group
Zn1 Zn 0.33487(6) 0.72237(9) 0.47345(5) 0.0789(4) Uani 1 1 d . . .
Zn2 Zn 0.48864(5) 0.80524(7) 0.36966(4) 0.0654(3) Uani 1 1 d . . .
Zn3 Zn 1.01548(6) -0.02351(7) 0.12644(4) 0.0641(3) Uani 1 1 d . . .
Zn4 Zn 1.16874(6) 0.06101(9) 0.02386(5) 0.0783(4) Uani 1 1 d . . .
Cl1 Cl 0.2408(3) 0.6915(4) 0.5117(3) 0.1204(18) Uani 0.80 1 d PU A 1
Cl1' Cl 0.2664(11) 0.6649(13) 0.5333(11) 0.104(6) Uani 0.20 1 d PU A 2
Cl2 Cl 0.41189(17) 0.8437(3) 0.28203(12) 0.1212(12) Uani 1 1 d . . .
Cl3 Cl 1.09573(15) -0.0617(3) 0.21244(11) 0.1065(11) Uani 1 1 d . . .
Cl4 Cl 1.25527(17) 0.0958(3) -0.02183(15) 0.1462(18) Uani 1 1 d . . .
N1 N 0.2927(4) 0.8201(8) 0.4179(3) 0.086(3) Uani 1 1 d DU A .
N2 N 0.4629(3) 0.9060(6) 0.5785(3) 0.0531(19) Uani 1 1 d U . .
N3 N 0.3895(4) 0.6217(7) 0.4474(3) 0.075(2) Uani 1 1 d U A .
N4 N 0.5434(4) 0.7046(7) 0.3475(3) 0.073(2) Uani 1 1 d U A .
N5 N 0.9650(4) -0.1168(5) 0.0751(3) 0.056(2) Uani 1 1 d U . .
N6 N 0.7877(4) -0.0346(7) -0.0755(3) 0.089(3) Uani 1 1 d DU . .
N7 N 1.1201(4) 0.1536(6) 0.0526(3) 0.070(2) Uani 1 1 d U . .
N8 N 0.9572(4) 0.0707(6) 0.1453(3) 0.069(2) Uani 1 1 d U . .
O2 O 0.4059(3) 0.7895(5) 0.5325(3) 0.072(2) Uani 1 1 d U A .
O3 O 0.5749(4) 0.5750(6) 0.3467(4) 0.110(3) Uani 1 1 d U . .
O4 O 0.4313(3) 0.7372(5) 0.4098(3) 0.077(2) Uani 1 1 d U A .
O5 O 0.9038(3) -0.0100(5) 0.0322(2) 0.0631(16) Uani 1 1 d U . .
O7 O 1.0713(3) 0.0466(5) 0.0852(3) 0.0717(19) Uani 1 1 d U . .
```

O8 O 0.9273(4) 0.2080(7) 0.1495(4) 0.126(3) Uani 1 1 d U . .
C1 C 0.4101(5) 0.8701(7) 0.5313(3) 0.053(2) Uani 1 1 d U . .
C2 C 0.4746(6) 0.9891(9) 0.5862(5) 0.086(3) Uani 1 1 d U . .
C3 C 0.4287(6) 1.0381(9) 0.5475(5) 0.111(4) Uani 1 1 d U A .
H3 H 0.4297 1.0954 0.5538 0.133 Uiso 1 1 calc R . .
C4 C 0.3766(7) 1.0024(9) 0.4953(5) 0.101(4) Uani 1 1 d U . .
H4 H 0.3495 1.0382 0.4660 0.122 Uiso 1 1 calc R A .
C5 C 0.3663(6) 0.9233(8) 0.4877(4) 0.066(3) Uani 1 1 d U A .
C6 C 0.3139(6) 0.8924(10) 0.4331(5) 0.079(3) Uani 1 1 d U . .
O1 O 0.2913(10) 0.9481(13) 0.3895(8) 0.083(5) Uani 0.55 1 d PU A 1
C7 C 0.2387(12) 0.9146(16) 0.3373(9) 0.095(6) Uani 0.55 1 d PDU A 1
H7A H 0.2493 0.9333 0.3009 0.115 Uiso 0.55 1 calc PR A 1
H7B H 0.1879 0.9291 0.3362 0.115 Uiso 0.55 1 calc PR A 1
C8 C 0.2530(16) 0.8210(16) 0.3473(15) 0.140(9) Uani 0.55 1 d PDU A 1
H8 H 0.2883 0.8022 0.3256 0.168 Uiso 0.55 1 calc PR A 1
C9 C 0.1899(6) 0.7564(11) 0.3401(6) 0.129(5) Uani 0.80 1 d PDU A 1
H9A H 0.1776 0.7517 0.3779 0.155 Uiso 0.80 1 calc PR A 1
H9B H 0.1461 0.7773 0.3109 0.155 Uiso 0.80 1 calc PR A 1
C10 C 0.2065(10) 0.6725(14) 0.3215(11) 0.153(10) Uani 0.50 1 d PDU A 1
H10 H 0.2367 0.6497 0.3594 0.184 Uiso 0.50 1 calc PR A 1
C11 C 0.2532(16) 0.650(3) 0.2802(12) 0.172(14) Uani 0.50 1 d PDU A 1
C12 C 0.1300(16) 0.6360(18) 0.3189(13) 0.136(12) Uani 0.50 1 d PDU A 1
O1’ O 0.2723(16) 0.9525(14) 0.3999(14) 0.109(9) Uani 0.45 1 d PDU A 2
C7’ C 0.2197(17) 0.915(2) 0.3607(13) 0.133(10) Uani 0.45 1 d PDU A 2
H7’1 H 0.2095 0.9424 0.3222 0.160 Uiso 0.45 1 calc PR A 2
H7’2 H 0.1743 0.9126 0.3733 0.160 Uiso 0.45 1 calc PR A 2
C8’ C 0.252(2) 0.8284(19) 0.3585(14) 0.103(7) Uani 0.45 1 d PDU A 2
H8’ H 0.2958 0.8526 0.3488 0.124 Uiso 0.45 1 calc PR A 2
C9’ C 0.241(2) 0.768(3) 0.305(2) 0.156(12) Uani 0.20 1 d PDU A 2
H9’1 H 0.2812 0.7276 0.3149 0.188 Uiso 0.20 1 calc PR A 2
H9’2 H 0.2467 0.7996 0.2712 0.188 Uiso 0.20 1 calc PR A 2
C10’ C 0.1661(12) 0.7162(18) 0.2824(10) 0.145(9) Uani 0.50 1 d PDU A 2
H10’ H 0.1402 0.7513 0.2487 0.174 Uiso 0.50 1 calc PR A 2
C11’ C 0.2072(13) 0.6574(15) 0.2500(11) 0.136(11) Uani 0.50 1 d PDU A 2
C12’ C 0.099(2) 0.662(3) 0.2886(14) 0.188(17) Uani 0.50 1 d PDU A 2
C13 C 0.5301(6) 1.0161(8) 0.6395(4) 0.105(4) Uani 1 1 d U A .
H13A H 0.5382 1.0749 0.6372 0.158 Uiso 1 1 calc R . .
H13B H 0.5759 0.9869 0.6427 0.158 Uiso 1 1 calc R . .
H13C H 0.5129 1.0044 0.6740 0.158 Uiso 1 1 calc R . .
C14 C 0.4360(5) 0.6574(9) 0.4164(4) 0.071(3) Uani 1 1 d U . .
C15 C 0.3856(7) 0.5406(9) 0.4563(5) 0.093(3) Uani 1 1 d U . .
C16 C 0.4297(8) 0.4952(7) 0.4342(6) 0.117(4) Uani 1 1 d U A .
H16 H 0.4308 0.4381 0.4407 0.141 Uiso 1 1 calc R . .
C17 C 0.4753(8) 0.5284(14) 0.4013(7) 0.143(6) Uani 1 1 d U . .

H17 H 0.5018 0.4919 0.3838 0.171 Uiso 1 1 calc R A .
C18 C 0.4819(7) 0.6035(7) 0.3945(5) 0.080(3) Uani 1 1 d U A .
C19 C 0.5346(5) 0.6321(9) 0.3625(4) 0.072(3) Uani 1 1 d U A .
C21 C 0.5979(5) 0.7054(8) 0.3107(5) 0.105(4) Uani 1 1 d DU . .
H21 H 0.5774 0.7125 0.2676 0.158 Uiso 0.50 1 calc PR B 1
H21' H 0.5824 0.7260 0.2705 0.158 Uiso 0.50 1 d PR B 2
C20 C 0.6391(15) 0.6231(12) 0.3320(15) 0.123(9) Uani 0.50 1 d PDU C 1
H20A H 0.6808 0.6309 0.3670 0.148 Uiso 0.50 1 calc PR C 1
H20B H 0.6560 0.5962 0.3007 0.148 Uiso 0.50 1 calc PR C 1
C22 C 0.6653(13) 0.7597(13) 0.3413(12) 0.116(8) Uani 0.50 1 d PDU C 1
H22A H 0.7100 0.7406 0.3317 0.139 Uiso 0.50 1 calc PR C 1
H22B H 0.6739 0.7596 0.3843 0.139 Uiso 0.50 1 calc PR C 1
C23 C 0.6421(11) 0.8481(15) 0.3148(8) 0.120(7) Uani 0.60 1 d PDU C 1
H23 H 0.5997 0.8680 0.3282 0.144 Uiso 0.60 1 calc PR C 1
C24 C 0.640(2) 0.889(3) 0.2541(10) 0.263(18) Uiso 0.50 1 d PDU C 1
C25 C 0.7115(16) 0.899(2) 0.3452(11) 0.226(16) Uani 0.60 1 d PDU C 1
C20' C 0.6162(14) 0.6106(13) 0.3102(14) 0.102(8) Uani 0.50 1 d PDU C 2
H20C H 0.6004 0.5883 0.2700 0.122 Uiso 0.50 1 calc PR C 2
H20D H 0.6691 0.6006 0.3265 0.122 Uiso 0.50 1 calc PR C 2
C22' C 0.6618(14) 0.7693(18) 0.3221(10) 0.097(8) Uani 0.50 1 d PDU C 2
H22C H 0.6580 0.8126 0.3502 0.117 Uiso 0.50 1 calc PR C 2
H22D H 0.7109 0.7441 0.3332 0.117 Uiso 0.50 1 calc PR C 2
C23' C 0.637(3) 0.797(3) 0.2568(14) 0.261(15) Uani 0.40 1 d PDU C 2
H23' H 0.5902 0.8271 0.2386 0.314 Uiso 0.40 1 calc PR C 2
C24' C 0.659(5) 0.711(4) 0.235(2) 0.51(4) Uiso 0.50 1 d PDU C 2
C25' C 0.716(4) 0.831(5) 0.259(2) 0.43(4) Uiso 0.40 1 d PDU C 2
C26 C 0.3307(7) 0.5183(11) 0.4899(6) 0.134(5) Uani 1 1 d U A .
H26A H 0.2812 0.5296 0.4655 0.201 Uiso 1 1 calc R . .
H26B H 0.3403 0.5509 0.5258 0.201 Uiso 1 1 calc R . .
H26C H 0.3352 0.4602 0.4999 0.201 Uiso 1 1 calc R . .
C27 C 0.9093(5) -0.0847(7) 0.0295(4) 0.057(2) Uani 1 1 d U . .
C28 C 0.9742(5) -0.2013(7) 0.0797(3) 0.063(2) Uani 1 1 d U . .
C29 C 0.9287(6) -0.2550(8) 0.0381(4) 0.097(4) Uani 1 1 d U . .
H29 H 0.9368 -0.3121 0.0425 0.117 Uiso 1 1 calc R . .
C30 C 0.8737(6) -0.2272(7) -0.0079(5) 0.087(3) Uani 1 1 d U . .
H30 H 0.8428 -0.2639 -0.0344 0.104 Uiso 1 1 calc R . .
C31 C 0.8646(5) -0.1404(8) -0.0146(4) 0.068(3) Uani 1 1 d U . .
C32 C 0.8075(6) -0.1082(8) -0.0639(4) 0.074(3) Uani 1 1 d DU D .
O6 O 0.7636(6) -0.1625(9) -0.0998(5) 0.091(4) Uani 0.70 1 d PU D 1
C33 C 0.7099(12) -0.0973(14) -0.1470(8) 0.087(5) Uani 0.50 1 d PDU D 1
H33A H 0.7231 -0.0980 -0.1845 0.104 Uiso 0.50 1 calc PR D 1
H33B H 0.6583 -0.1146 -0.1548 0.104 Uiso 0.50 1 calc PR D 1
C34 C 0.7184(13) -0.0126(13) -0.1224(9) 0.102(7) Uani 0.50 1 d PDU D 1
H34 H 0.6777 0.0023 -0.1052 0.123 Uiso 0.50 1 calc PR D 1

C35 C 0.7310(14) 0.0530(14) -0.1646(9) 0.104(7) Uani 0.50 1 d PDU D 1
H35A H 0.7712 0.0340 -0.1804 0.125 Uiso 0.50 1 calc PR D 1
H35B H 0.7484 0.1028 -0.1414 0.125 Uiso 0.50 1 calc PR D 1
C36 C 0.6649(13) 0.0788(17) -0.2186(11) 0.139(8) Uani 0.50 1 d PDU D 1
H36 H 0.6748 0.0390 -0.2474 0.166 Uiso 0.50 1 calc PR D 1
C37 C 0.5968(17) 0.122(4) -0.262(3) 0.43(3) Uiso 0.50 1 d PDU D 1
C38 C 0.7158(16) 0.1491(19) -0.2296(13) 0.134(10) Uani 0.40 1 d PDU D 1
O6’ O 0.7934(17) -0.189(2) -0.1076(15) 0.099(9) Uani 0.30 1 d PU D 2
C33’ C 0.7343(13) -0.1362(16) -0.1522(9) 0.108(7) Uani 0.50 1 d PDU D 2
H33C H 0.6871 -0.1656 -0.1613 0.130 Uiso 0.50 1 calc PR D 2
H33D H 0.7488 -0.1313 -0.1890 0.130 Uiso 0.50 1 calc PR D 2
C34’ C 0.7225(13) -0.0476(14) -0.1301(8) 0.091(6) Uani 0.50 1 d PDU D 2
H34’ H 0.6748 -0.0432 -0.1202 0.109 Uiso 0.50 1 calc PR D 2
C35’ C 0.7249(13) 0.0094(12) -0.1809(8) 0.103(6) Uani 0.50 1 d PDU D 2
H35C H 0.7714 -0.0003 -0.1909 0.123 Uiso 0.50 1 calc PR D 2
H35D H 0.6848 -0.0063 -0.2154 0.123 Uiso 0.50 1 calc PR D 2
C36’ C 0.7186(11) 0.0996(10) -0.1706(9) 0.078(6) Uani 0.50 1 d PDU D 2
H36’ H 0.7371 0.1016 -0.1269 0.093 Uiso 0.50 1 calc PR D 2
C37’ C 0.732(2) 0.1902(19) -0.186(3) 0.31(2) Uiso 0.50 1 d PDU D 2
C38’ C 0.6390(12) 0.1063(15) -0.1778(12) 0.159(9) Uani 0.60 1 d PDU D 2
C39 C 1.0390(6) -0.2342(9) 0.1284(4) 0.111(5) Uani 1 1 d U . .
H39A H 1.0801 -0.1961 0.1345 0.167 Uiso 1 1 calc R . .
H39B H 1.0537 -0.2876 0.1169 0.167 Uiso 1 1 calc R . .
H39C H 1.0245 -0.2396 0.1648 0.167 Uiso 1 1 calc R . .
C40 C 1.0711(5) 0.1264(7) 0.0830(4) 0.061(3) Uani 1 1 d U . .
C41 C 1.1263(7) 0.2365(11) 0.0468(5) 0.103(4) Uani 1 1 d U . .
C42 C 1.0836(8) 0.3010(11) 0.0725(6) 0.144(5) Uani 1 1 d U . .
H42 H 1.0915 0.3581 0.0717 0.173 Uiso 1 1 calc R . .
C43 C 1.0305(7) 0.2639(8) 0.0976(5) 0.100(4) Uani 1 1 d U . .
H43 H 0.9974 0.2989 0.1094 0.120 Uiso 1 1 calc R . .
C44 C 1.0241(5) 0.1766(8) 0.1062(5) 0.072(3) Uani 1 1 d U . .
C45 C 0.9727(6) 0.1447(9) 0.1333(4) 0.079(3) Uani 1 1 d U . .
C46 C 0.8799(6) 0.1622(9) 0.1815(5) 0.103(4) Uani 1 1 d U . .
H46A H 0.8982 0.1698 0.2244 0.124 Uiso 1 1 calc R . .
H46B H 0.8285 0.1806 0.1684 0.124 Uiso 1 1 calc R . .
C47 C 0.8870(6) 0.0757(8) 0.1650(5) 0.102(3) Uani 1 1 d DU . .
H47 H 0.8446 0.0615 0.1311 0.123 Uiso 1 1 calc R . .
C48 C 0.8891(7) 0.0158(7) 0.2155(5) 0.124(4) Uani 1 1 d DU . .
H48A H 0.8495 0.0311 0.2332 0.149 Uiso 1 1 calc R . .
H48B H 0.9358 0.0241 0.2459 0.149 Uiso 1 1 calc R . .
C49 C 0.8814(7) -0.0799(7) 0.2007(5) 0.109(4) Uani 1 1 d DU . .
H49 H 0.9176 -0.0969 0.1794 0.131 Uiso 1 1 calc R . .
C50 C 0.9040(10) -0.1149(14) 0.2653(6) 0.245(9) Uiso 1 1 d DU . .
H50A H 0.8685 -0.1561 0.2695 0.368 Uiso 1 1 calc R . .

H50B H 0.9051 -0.0704 0.2930 0.368 Uiso 1 1 calc R . .
H50C H 0.9525 -0.1398 0.2733 0.368 Uiso 1 1 calc R . .
C51 C 0.8076(9) -0.1047(15) 0.1680(8) 0.215(8) Uiso 1 1 d DU . .
H51A H 0.7949 -0.0786 0.1294 0.322 Uiso 1 1 calc R . .
H51B H 0.7728 -0.0880 0.1894 0.322 Uiso 1 1 calc R . .
H51C H 0.8060 -0.1641 0.1632 0.322 Uiso 1 1 calc R . .
C52 C 1.1762(6) 0.2769(10) 0.0124(6) 0.126(5) Uani 1 1 d U . .
H52A H 1.2270 0.2610 0.0302 0.189 Uiso 1 1 calc R . .
H52B H 1.1718 0.3364 0.0139 0.189 Uiso 1 1 calc R . .
H52C H 1.1611 0.2586 -0.0284 0.189 Uiso 1 1 calc R . .

loop_
 _atom_site_aniso_label
 _atom_site_aniso_U_11
 _atom_site_aniso_U_22
 _atom_site_aniso_U_33
 _atom_site_aniso_U_23
 _atom_site_aniso_U_13
 _atom_site_aniso_U_12
Zn1 0.0628(7) 0.0816(11) 0.0848(7) -0.0211(7) 0.0072(6) -0.0141(7)
Zn2 0.0607(6) 0.0625(9) 0.0628(6) -0.0075(6) -0.0006(5) -0.0008(7)
Zn3 0.0620(6) 0.0620(9) 0.0585(6) -0.0040(6) -0.0003(5) -0.0009(6)
Zn4 0.0648(7) 0.0845(11) 0.0776(7) -0.0221(7) 0.0056(6) -0.0117(7)
Cl1 0.100(4) 0.120(5) 0.152(5) 0.003(3) 0.052(3) -0.021(3)
Cl1’ 0.088(12) 0.066(10) 0.155(17) 0.019(9) 0.030(10) 0.001(8)
Cl2 0.135(2) 0.118(3) 0.0734(15) -0.0063(17) -0.0354(16) 0.014(2)
Cl3 0.1042(17) 0.110(3) 0.0730(15) 0.0014(16) -0.0317(14) 0.010(2)
Cl4 0.1079(19) 0.183(5) 0.168(3) -0.068(3) 0.071(2) -0.071(3)
N1 0.058(4) 0.122(9) 0.065(5) 0.000(5) -0.005(4) -0.009(6)
N2 0.045(3) 0.058(6) 0.050(4) 0.000(4) 0.001(3) 0.002(4)
N3 0.061(4) 0.073(7) 0.075(5) -0.016(4) -0.010(4) 0.002(4)
N4 0.068(4) 0.082(7) 0.065(4) -0.017(4) 0.009(4) 0.008(5)
N5 0.072(4) 0.052(5) 0.044(3) 0.006(4) 0.013(3) 0.001(4)
N6 0.073(5) 0.101(8) 0.067(4) 0.035(5) -0.025(4) -0.038(5)
N7 0.067(4) 0.065(6) 0.074(5) 0.018(4) 0.011(4) -0.014(4)
N8 0.079(5) 0.063(6) 0.065(4) -0.014(4) 0.019(4) -0.010(5)
O2 0.060(3) 0.079(6) 0.064(4) -0.012(4) -0.008(3) -0.010(4)
O3 0.115(5) 0.075(6) 0.147(6) -0.043(5) 0.047(5) 0.003(5)
O4 0.083(4) 0.054(5) 0.089(4) -0.017(4) 0.016(3) 0.003(4)
O5 0.070(4) 0.040(4) 0.066(3) -0.003(3) -0.005(3) -0.002(3)
O7 0.062(3) 0.069(6) 0.087(4) 0.003(4) 0.026(3) -0.002(4)
O8 0.118(6) 0.118(9) 0.151(6) 0.010(6) 0.054(5) 0.039(6)
C1 0.051(5) 0.059(7) 0.049(4) -0.013(5) 0.011(4) 0.007(5)
C2 0.093(7) 0.074(9) 0.082(6) 0.001(6) 0.011(6) -0.014(7)

C3	0.136(9)	0.066(8)	0.095(7)	-0.015(6)	-0.029(6)	-0.005(8)
C4	0.121(9)	0.101(11)	0.074(6)	0.032(7)	0.012(6)	0.042(8)
C5	0.079(6)	0.061(7)	0.054(5)	-0.003(5)	0.011(5)	0.034(6)
C6	0.057(5)	0.093(9)	0.080(7)	-0.001(7)	0.010(5)	0.019(6)
O1	0.069(8)	0.123(11)	0.050(7)	0.014(7)	0.000(6)	0.022(7)
C7	0.086(8)	0.112(9)	0.071(8)	-0.003(7)	-0.010(6)	-0.002(7)
C8	0.102(12)	0.192(16)	0.081(15)	-0.031(13)	-0.050(11)	-0.022(13)
C9	0.068(6)	0.217(15)	0.087(8)	-0.038(10)	-0.004(6)	-0.035(9)
C10	0.122(14)	0.157(19)	0.118(15)	0.033(15)	-0.074(12)	-0.100(15)
C11	0.17(3)	0.26(4)	0.103(17)	-0.09(2)	0.063(18)	-0.04(2)
C12	0.19(3)	0.13(2)	0.11(2)	-0.040(18)	0.077(18)	-0.09(2)
O1’	0.105(11)	0.116(11)	0.088(11)	0.006(8)	-0.004(8)	0.016(8)
C7’	0.109(16)	0.159(18)	0.113(17)	0.006(17)	-0.002(14)	-0.017(16)
C8’	0.094(12)	0.170(16)	0.036(10)	-0.037(11)	0.001(9)	-0.001(13)
C9’	0.13(2)	0.19(2)	0.095(19)	-0.03(2)	-0.051(18)	-0.01(2)
C10’	0.144(16)	0.156(19)	0.087(12)	0.006(14)	-0.050(12)	-0.054(15)
C11’	0.125(18)	0.14(2)	0.19(2)	0.07(2)	0.112(18)	0.025(16)
C12’	0.28(4)	0.17(3)	0.11(2)	-0.05(2)	0.05(2)	-0.04(3)
C13	0.129(8)	0.059(8)	0.100(7)	-0.017(6)	-0.014(7)	-0.020(7)
C14	0.067(6)	0.077(9)	0.059(5)	-0.023(6)	0.001(5)	-0.018(6)
C15	0.124(8)	0.051(7)	0.100(7)	0.006(6)	0.025(6)	0.001(7)
C16	0.177(10)	0.029(5)	0.159(9)	0.010(6)	0.067(9)	0.013(7)
C17	0.152(11)	0.141(15)	0.147(11)	0.008(11)	0.060(9)	0.057(11)
C18	0.099(7)	0.042(6)	0.080(6)	-0.010(6)	-0.007(6)	0.014(6)
C19	0.060(5)	0.074(8)	0.073(6)	-0.012(6)	0.003(5)	0.019(6)
C21	0.083(6)	0.131(10)	0.106(7)	-0.046(7)	0.033(5)	-0.017(6)
C20	0.090(17)	0.114(17)	0.16(2)	-0.057(15)	0.024(13)	0.015(14)
C22	0.118(15)	0.144(17)	0.105(17)	-0.033(15)	0.061(12)	-0.018(14)
C23	0.112(11)	0.175(18)	0.103(11)	0.014(12)	0.081(10)	0.039(13)
C25	0.19(3)	0.28(4)	0.22(3)	0.10(3)	0.09(2)	0.04(3)
C20’	0.064(12)	0.099(15)	0.143(17)	-0.064(13)	0.028(12)	-0.030(11)
C22’	0.088(12)	0.134(16)	0.057(12)	-0.008(11)	0.000(10)	-0.034(12)
C23’	0.24(2)	0.27(3)	0.26(2)	0.00(2)	0.04(2)	0.03(2)
C26	0.157(11)	0.098(11)	0.164(11)	0.033(10)	0.072(9)	-0.007(10)
C27	0.059(5)	0.054(7)	0.051(5)	-0.010(5)	0.005(4)	-0.002(5)
C28	0.081(6)	0.042(6)	0.056(4)	0.007(4)	0.001(4)	0.004(5)
C29	0.138(9)	0.060(7)	0.090(7)	-0.014(6)	0.023(7)	-0.024(7)
C30	0.107(8)	0.042(6)	0.083(6)	-0.003(5)	-0.021(5)	-0.016(6)
C31	0.046(5)	0.091(9)	0.058(5)	-0.007(6)	-0.003(4)	-0.013(6)
C32	0.081(6)	0.079(8)	0.049(5)	0.006(5)	-0.005(5)	-0.030(6)
O6	0.080(6)	0.105(8)	0.056(5)	-0.005(5)	-0.038(4)	-0.026(5)
C33	0.087(8)	0.092(9)	0.051(7)	0.000(7)	-0.033(6)	0.019(8)
C34	0.114(12)	0.080(14)	0.087(11)	-0.013(11)	-0.016(10)	0.001(12)
C35	0.137(14)	0.086(15)	0.069(12)	-0.016(12)	-0.006(10)	-0.080(13)

C36 0.145(16) 0.116(17) 0.134(16) -0.018(15) 0.002(15) 0.005(16)
C38 0.157(13) 0.135(14) 0.129(12) 0.024(9) 0.072(9) -0.009(9)
O6' 0.116(17) 0.069(14) 0.083(14) -0.018(11) -0.022(14) 0.000(13)
C33' 0.113(10) 0.107(11) 0.092(10) -0.013(8) 0.007(8) -0.004(8)
C34' 0.116(12) 0.070(14) 0.065(10) -0.027(11) -0.011(9) -0.008(12)
C35' 0.122(13) 0.095(14) 0.069(11) -0.026(10) -0.012(10) -0.011(12)
C36' 0.125(13) 0.036(10) 0.065(9) 0.012(9) 0.013(9) -0.025(11)
C38' 0.22(2) 0.13(2) 0.151(19) 0.001(17) 0.088(18) 0.03(2)
C39 0.141(9) 0.082(10) 0.084(6) 0.005(6) -0.015(6) 0.053(8)
C40 0.058(5) 0.047(6) 0.065(5) 0.001(5) -0.009(4) 0.006(5)
C41 0.100(7) 0.090(10) 0.115(8) 0.002(8) 0.023(6) -0.023(8)
C42 0.174(11) 0.082(10) 0.192(12) 0.008(9) 0.078(10) -0.029(9)
C43 0.120(8) 0.052(7) 0.131(9) -0.003(6) 0.039(7) -0.006(7)
C44 0.061(5) 0.081(8) 0.078(6) -0.012(6) 0.024(5) -0.001(6)
C45 0.084(7) 0.079(8) 0.070(6) -0.018(6) 0.013(5) 0.015(7)
C46 0.113(8) 0.103(10) 0.117(8) -0.015(7) 0.070(7) -0.003(8)
C47 0.095(7) 0.103(9) 0.113(7) -0.008(7) 0.037(6) 0.020(7)
C48 0.157(9) 0.129(10) 0.110(7) -0.010(7) 0.077(7) -0.045(8)
C49 0.138(9) 0.068(6) 0.129(8) 0.011(6) 0.049(7) -0.009(7)
C52 0.140(10) 0.095(11) 0.160(10) 0.009(9) 0.070(9) -0.028(9)

_geom_special_details
;
 All esds (except the esd in the dihedral angle between two l.s. planes)
 are estimated using the full covariance matrix. The cell esds are taken
 into account individually in the estimation of esds in distances, angles
 and torsion angles; correlations between esds in cell parameters are only
 used when they are defined by crystal symmetry. An approximate (isotropic)
 treatment of cell esds is used for estimating esds involving l.s. planes.
;

loop_
 _geom_bond_atom_site_label_1
 _geom_bond_atom_site_label_2
 _geom_bond_distance
 _geom_bond_site_symmetry_2
 _geom_bond_publ_flag
Zn1 O2 1.953(6) . ?
Zn1 N1 2.050(10) . ?
Zn1 N3 2.087(10) . ?
Zn1 Cl1 2.228(6) . ?
Zn1 Cl1' 2.32(3) . ?
Zn2 O4 1.938(8) . ?
Zn2 N4 2.051(10) . ?

Zn2 N2 2.073(8) 2_656 ?
Zn2 Cl2 2.237(3) . ?
Zn3 O7 1.954(7) . ?
Zn3 N8 1.979(9) . ?
Zn3 N5 1.987(8) . ?
Zn3 Cl3 2.235(3) . ?
Zn4 N7 1.953(10) . ?
Zn4 O5 1.967(6) 2_755 ?
Zn4 N6 1.982(10) 2_755 ?
Zn4 Cl4 2.232(3) . ?
N1 C6 1.246(16) . ?
N1 C8' 1.39(3) . ?
N1 C8 1.61(3) . ?
N2 C2 1.355(16) . ?
N2 C1 1.386(11) . ?
N2 Zn2 2.073(8) 2_656 ?
N3 C15 1.324(16) . ?
N3 C14 1.392(13) . ?
N4 C19 1.239(15) . ?
N4 C21 1.496(12) . ?
N5 C27 1.368(11) . ?
N5 C28 1.369(14) . ?
N6 C32 1.245(15) . ?
N6 C34 1.490(17) . ?
N6 C34' 1.517(16) . ?
N6 Zn4 1.982(10) 2_755 ?
N7 C41 1.347(18) . ?
N7 C40 1.369(11) . ?
N8 C45 1.272(15) . ?
N8 C47 1.504(12) . ?
O2 C1 1.299(12) . ?
O3 C19 1.300(13) . ?
O3 C20' 1.41(3) . ?
O3 C20 1.54(3) . ?
O4 C14 1.291(14) . ?
O5 C27 1.208(12) . ?
O5 Zn4 1.967(6) 2_755 ?
O7 C40 1.283(12) . ?
O8 C45 1.438(14) . ?
O8 C46 1.495(13) . ?
C1 C5 1.405(13) . ?
C2 C3 1.321(16) . ?
C2 C13 1.450(13) . ?
C3 C4 1.452(14) . ?

C3 H3 0.9300 . ?
C4 C5 1.289(18) . ?
C4 H4 0.9300 . ?
C5 C6 1.464(15) . ?
C6 O1 1.33(2) . ?
C6 O1’ 1.34(3) . ?
O1 C7 1.44(3) . ?
C7 C8 1.533(18) . ?
C7 H7A 0.9700 . ?
C7 H7B 0.9700 . ?
C8 C9 1.543(19) . ?
C8 H8 0.9800 . ?
C9 C10 1.473(18) . ?
C9 H9A 0.9700 . ?
C9 H9B 0.9700 . ?
C10 C11 1.509(18) . ?
C10 C12 1.529(18) . ?
C10 H10 0.9800 . ?
O1’ C7’ 1.29(4) . ?
C7’ C8’ 1.524(19) . ?
C7’ H7’1 0.9700 . ?
C7’ H7’2 0.9700 . ?
C8’ C9’ 1.552(19) . ?
C8’ H8’ 0.9800 . ?
C9’ C10’ 1.58(2) . ?
C9’ H9’1 0.9700 . ?
C9’ H9’2 0.9700 . ?
C10’ C11’ 1.537(18) . ?
C10’ C12’ 1.562(19) . ?
C10’ H10’ 0.9800 . ?
C13 H13A 0.9600 . ?
C13 H13B 0.9600 . ?
C13 H13C 0.9600 . ?
C14 C18 1.409(16) . ?
C15 C16 1.303(16) . ?
C15 C26 1.491(16) . ?
C16 C17 1.396(19) . ?
C16 H16 0.9300 . ?
C17 C18 1.23(2) . ?
C17 H17 0.9300 . ?
C18 C19 1.459(17) . ?
C21 C22 1.537(14) . ?
C21 C22’ 1.539(17) . ?
C21 C20 1.541(17) . ?

C21 C20' 1.561(18) . ?
C21 H21 0.9800 . ?
C21 H21' 0.9605 . ?
C20 H20A 0.9700 . ?
C20 H20B 0.9700 . ?
C22 C23 1.561(14) . ?
C22 H22A 0.9700 . ?
C22 H22B 0.9700 . ?
C23 C25 1.532(14) . ?
C23 C24 1.546(14) . ?
C23 H23 0.9800 . ?
C20' H20C 0.9700 . ?
C20' H20D 0.9700 . ?
C22' C23' 1.53(2) . ?
C22' H22C 0.9700 . ?
C22' H22D 0.9700 . ?
C23' C25' 1.56(2) . ?
C23' C24' 1.57(2) . ?
C23' H23' 0.9800 . ?
C26 H26A 0.9600 . ?
C26 H26B 0.9600 . ?
C26 H26C 0.9600 . ?
C27 C31 1.443(13) . ?
C28 C29 1.396(13) . ?
C28 C39 1.510(12) . ?
C29 C30 1.343(13) . ?
C29 H29 0.9300 . ?
C30 C31 1.408(16) . ?
C30 H30 0.9300 . ?
C31 C32 1.433(14) . ?
C32 O6 1.325(15) . ?
C32 O6' 1.62(3) . ?
O6 C33 1.65(2) . ?
C33 C34 1.466(17) . ?
C33 H33A 0.9700 . ?
C33 H33B 0.9700 . ?
C34 C35 1.501(18) . ?
C34 H34 0.9800 . ?
C35 C36 1.559(18) . ?
C35 H35A 0.9700 . ?
C35 H35B 0.9700 . ?
C36 C38 1.541(18) . ?
C36 C37 1.55(2) . ?
C36 H36 0.9800 . ?

O6’ C33’ 1.55(4) . ?
C33’ C34’ 1.549(18) . ?
C33’ H33C 0.9700 . ?
C33’ H33D 0.9700 . ?
C34’ C35’ 1.504(18) . ?
C34’ H34’ 0.9800 . ?
C35’ C36’ 1.478(16) . ?
C35’ H35C 0.9700 . ?
C35’ H35D 0.9700 . ?
C36’ C38’ 1.451(18) . ?
C36’ C37’ 1.54(2) . ?
C36’ H36’ 0.9800 . ?
C39 H39A 0.9600 . ?
C39 H39B 0.9600 . ?
C39 H39C 0.9600 . ?
C40 C44 1.402(14) . ?
C41 C42 1.52(2) . ?
C41 C52 1.527(16) . ?
C42 C43 1.411(17) . ?
C42 H42 0.9300 . ?
C43 C44 1.425(17) . ?
C43 H43 0.9300 . ?
C44 C45 1.381(15) . ?
C46 C47 1.457(16) . ?
C46 H46A 0.9700 . ?
C46 H46B 0.9700 . ?
C47 C48 1.512(12) . ?
C47 H47 0.9800 . ?
C48 C49 1.573(13) . ?
C48 H48A 0.9700 . ?
C48 H48B 0.9700 . ?
C49 C51 1.436(14) . ?
C49 C50 1.551(14) . ?
C49 H49 0.9800 . ?
C50 H50A 0.9600 . ?
C50 H50B 0.9600 . ?
C50 H50C 0.9600 . ?
C51 H51A 0.9600 . ?
C51 H51B 0.9600 . ?
C51 H51C 0.9600 . ?
C52 H52A 0.9600 . ?
C52 H52B 0.9600 . ?
C52 H52C 0.9600 . ?

```
loop_
 _geom_angle_atom_site_label_1
 _geom_angle_atom_site_label_2
 _geom_angle_atom_site_label_3
 _geom_angle
 _geom_angle_site_symmetry_1
 _geom_angle_site_symmetry_3
 _geom_angle_publ_flag
O2 Zn1 N1 95.3(4) . . ?
O2 Zn1 N3 109.9(3) . . ?
N1 Zn1 N3 122.9(4) . . ?
O2 Zn1 Cl1 107.4(3) . . ?
N1 Zn1 Cl1 102.3(3) . . ?
N3 Zn1 Cl1 116.4(3) . . ?
O2 Zn1 Cl1' 100.1(7) . . ?
N1 Zn1 Cl1' 120.2(5) . . ?
N3 Zn1 Cl1' 105.0(6) . . ?
Cl1 Zn1 Cl1' 18.3(4) . . ?
O4 Zn2 N4 93.2(4) . . ?
O4 Zn2 N2 111.7(3) . 2_656 ?
N4 Zn2 N2 126.4(3) . 2_656 ?
O4 Zn2 Cl2 107.1(2) . . ?
N4 Zn2 Cl2 103.1(2) . . ?
N2 Zn2 Cl2 112.7(3) 2_656 . ?
O7 Zn3 N8 93.8(3) . . ?
O7 Zn3 N5 111.6(3) . . ?
N8 Zn3 N5 120.7(3) . . ?
O7 Zn3 Cl3 106.4(2) . . ?
N8 Zn3 Cl3 106.6(2) . . ?
N5 Zn3 Cl3 115.1(3) . . ?
N7 Zn4 O5 111.6(3) . 2_755 ?
N7 Zn4 N6 122.3(3) . 2_755 ?
O5 Zn4 N6 92.7(3) 2_755 2_755 ?
N7 Zn4 Cl4 115.9(3) . . ?
O5 Zn4 Cl4 106.6(2) 2_755 . ?
N6 Zn4 Cl4 104.6(3) 2_755 . ?
C6 N1 C8' 103.8(15) . . ?
C6 N1 C8 107.7(13) . . ?
C8' N1 C8 7(2) . . ?
C6 N1 Zn1 119.7(8) . . ?
C8' N1 Zn1 135.4(14) . . ?
C8 N1 Zn1 130.0(13) . . ?
C2 N2 C1 124.6(9) . . ?
C2 N2 Zn2 131.4(7) . 2_656 ?
```

C1 N2 Zn2 103.9(7) . 2_656 ?
C15 N3 C14 123.7(11) . . ?
C15 N3 Zn1 131.6(9) . . ?
C14 N3 Zn1 104.7(8) . . ?
C19 N4 C21 109.3(10) . . ?
C19 N4 Zn2 123.8(8) . . ?
C21 N4 Zn2 126.9(9) . . ?
C27 N5 C28 119.1(8) . . ?
C27 N5 Zn3 108.6(7) . . ?
C28 N5 Zn3 132.2(6) . . ?
C32 N6 C34 122.0(11) . . ?
C32 N6 C34' 100.0(11) . . ?
C34 N6 C34' 23.0(10) . . ?
C32 N6 Zn4 123.8(6) . 2_755 ?
C34 N6 Zn4 113.1(10) . 2_755 ?
C34' N6 Zn4 135.9(11) . 2_755 ?
C41 N7 C40 117.0(11) . . ?
C41 N7 Zn4 131.1(8) . . ?
C40 N7 Zn4 111.9(7) . . ?
C45 N8 C47 106.6(10) . . ?
C45 N8 Zn3 119.7(7) . . ?
C47 N8 Zn3 133.2(8) . . ?
C1 O2 Zn1 124.6(6) . . ?
C19 O3 C20' 109.6(12) . . ?
C19 O3 C20 104.6(12) . . ?
C20' O3 C20 23.1(16) . . ?
C14 O4 Zn2 126.0(7) . . ?
C27 O5 Zn4 126.5(6) . 2_755 ?
C40 O7 Zn3 126.8(7) . . ?
C45 O8 C46 104.7(10) . . ?
O2 C1 N2 115.5(8) . . ?
O2 C1 C5 126.5(9) . . ?
N2 C1 C5 117.9(10) . . ?
C3 C2 N2 116.4(10) . . ?
C3 C2 C13 125.8(13) . . ?
N2 C2 C13 117.4(11) . . ?
C2 C3 C4 119.8(12) . . ?
C2 C3 H3 120.1 . . ?
C4 C3 H3 120.1 . . ?
C5 C4 C3 123.0(11) . . ?
C5 C4 H4 118.5 . . ?
C3 C4 H4 118.5 . . ?
C4 C5 C1 117.6(11) . . ?
C4 C5 C6 119.5(12) . . ?

C1 C5 C6 122.7(12) . . ?
N1 C6 O1 113.4(14) . . ?
N1 C6 O1' 114.6(15) . . ?
O1 C6 O1' 20.9(16) . . ?
N1 C6 C5 130.5(12) . . ?
O1 C6 C5 115.6(16) . . ?
O1' C6 C5 113.6(18) . . ?
C6 O1 C7 113.2(19) . . ?
O1 C7 C8 100.8(17) . . ?
O1 C7 H7A 111.8 . . ?
C8 C7 H7A 111.7 . . ?
O1 C7 H7B 111.5 . . ?
C8 C7 H7B 111.5 . . ?
H7A C7 H7B 109.4 . . ?
C7 C8 C9 123(3) . . ?
C7 C8 N1 100.2(19) . . ?
C9 C8 N1 103.3(19) . . ?
C7 C8 H8 109.6 . . ?
C9 C8 H8 109.7 . . ?
N1 C8 H8 109.8 . . ?
C10 C9 C8 115.5(18) . . ?
C10 C9 H9A 108.3 . . ?
C8 C9 H9A 108.3 . . ?
C10 C9 H9B 108.5 . . ?
C8 C9 H9B 108.5 . . ?
H9A C9 H9B 107.5 . . ?
C9 C10 C11 128(2) . . ?
C9 C10 C12 95.4(14) . . ?
C11 C10 C12 125(2) . . ?
C9 C10 H10 101.2 . . ?
C11 C10 H10 101.1 . . ?
C12 C10 H10 101.1 . . ?
C7' O1' C6 106.3(19) . . ?
O1' C7' C8' 102(2) . . ?
O1' C7' H7'1 111.4 . . ?
C8' C7' H7'1 111.3 . . ?
O1' C7' H7'2 110.8 . . ?
C8' C7' H7'2 111.6 . . ?
H7'1 C7' H7'2 109.1 . . ?
N1 C8' C7' 99.6(17) . . ?
N1 C8' C9' 131(3) . . ?
C7' C8' C9' 129(4) . . ?
N1 C8' H8' 89.4 . . ?
C7' C8' H8' 89.7 . . ?

C9’ C8’ H8’ 89.6 . . ?
C8’ C9’ C10’ 121(3) . . ?
C8’ C9’ H9’1 107.0 . . ?
C10’ C9’ H9’1 107.4 . . ?
C8’ C9’ H9’2 106.8 . . ?
C10’ C9’ H9’2 107.2 . . ?
H9’1 C9’ H9’2 106.9 . . ?
C11’ C10’ C12’ 103(2) . . ?
C11’ C10’ C9’ 88.4(13) . . ?
C12’ C10’ C9’ 156(3) . . ?
C11’ C10’ H10’ 99.4 . . ?
C12’ C10’ H10’ 99.4 . . ?
C9’ C10’ H10’ 99.5 . . ?
C2 C13 H13A 109.5 . . ?
C2 C13 H13B 109.5 . . ?
H13A C13 H13B 109.5 . . ?
C2 C13 H13C 109.5 . . ?
H13A C13 H13C 109.5 . . ?
H13B C13 H13C 109.5 . . ?
O4 C14 N3 116.0(10) . . ?
O4 C14 C18 126.5(11) . . ?
N3 C14 C18 117.4(12) . . ?
C16 C15 N3 114.8(12) . . ?
C16 C15 C26 132.1(14) . . ?
N3 C15 C26 113.1(13) . . ?
C15 C16 C17 123.2(13) . . ?
C15 C16 H16 118.4 . . ?
C17 C16 H16 118.4 . . ?
C18 C17 C16 123.0(16) . . ?
C18 C17 H17 118.5 . . ?
C16 C17 H17 118.5 . . ?
C17 C18 C14 117.6(15) . . ?
C17 C18 C19 118.8(14) . . ?
C14 C18 C19 123.5(11) . . ?
N4 C19 O3 116.9(11) . . ?
N4 C19 C18 126.8(11) . . ?
O3 C19 C18 116.2(12) . . ?
N4 C21 C22 109.8(15) . . ?
N4 C21 C22’ 121.5(14) . . ?
C22 C21 C22’ 17.2(15) . . ?
N4 C21 C20 99.5(16) . . ?
C22 C21 C20 93.7(11) . . ?
C22’ C21 C20 102.8(18) . . ?
N4 C21 C20’ 100.4(16) . . ?

C22 C21 C20’ 113.8(14) . . ?
C22’ C21 C20’ 119.4(18) . . ?
C20 C21 C20’ 22.4(14) . . ?
N4 C21 H21 116.8 . . ?
C22 C21 H21 116.8 . . ?
C22’ C21 H21 99.6 . . ?
C20 C21 H21 116.8 . . ?
C20’ C21 H21 97.5 . . ?
N4 C21 H21’ 119.2 . . ?
C22 C21 H21’ 104.3 . . ?
C22’ C21 H21’ 87.2 . . ?
C20 C21 H21’ 126.9 . . ?
C20’ C21 H21’ 109.7 . . ?
H21 C21 H21’ 14.1 . . ?
C21 C20 O3 98.8(17) . . ?
C21 C20 H20A 112.0 . . ?
O3 C20 H20A 112.0 . . ?
C21 C20 H20B 112.0 . . ?
O3 C20 H20B 112.0 . . ?
H20A C20 H20B 109.7 . . ?
C21 C22 C23 103.2(18) . . ?
C21 C22 H22A 111.1 . . ?
C23 C22 H22A 111.2 . . ?
C21 C22 H22B 111.1 . . ?
C23 C22 H22B 111.1 . . ?
H22A C22 H22B 109.1 . . ?
C25 C23 C24 90.2(12) . . ?
C25 C23 C22 101.2(19) . . ?
C24 C23 C22 133(3) . . ?
C25 C23 H23 109.5 . . ?
C24 C23 H23 109.3 . . ?
C22 C23 H23 109.3 . . ?
O3 C20’ C21 103.5(19) . . ?
O3 C20’ H20C 111.1 . . ?
C21 C20’ H20C 111.1 . . ?
O3 C20’ H20D 111.1 . . ?
C21 C20’ H20D 111.0 . . ?
H20C C20’ H20D 109.0 . . ?
C23’ C22’ C21 91(2) . . ?
C23’ C22’ H22C 113.7 . . ?
C21 C22’ H22C 113.5 . . ?
C23’ C22’ H22D 113.5 . . ?
C21 C22’ H22D 113.4 . . ?
H22C C22’ H22D 110.8 . . ?

C22’ C23’ C25’ 92.1(15) . . ?
C22’ C23’ C24’ 91.7(15) . . ?
C25’ C23’ C24’ 88.9(15) . . ?
C22’ C23’ H23’ 124.7 . . ?
C25’ C23’ H23’ 124.2 . . ?
C24’ C23’ H23’ 125.0 . . ?
C15 C26 H26A 109.4 . . ?
C15 C26 H26B 109.5 . . ?
H26A C26 H26B 109.5 . . ?
C15 C26 H26C 109.5 . . ?
H26A C26 H26C 109.5 . . ?
H26B C26 H26C 109.5 . . ?
O5 C27 N5 113.0(8) . . ?
O5 C27 C31 127.7(10) . . ?
N5 C27 C31 119.3(10) . . ?
N5 C28 C29 121.2(9) . . ?
N5 C28 C39 117.5(10) . . ?
C29 C28 C39 121.1(12) . . ?
C30 C29 C28 122.4(12) . . ?
C30 C29 H29 118.8 . . ?
C28 C29 H29 118.8 . . ?
C29 C30 C31 117.4(10) . . ?
C29 C30 H30 121.3 . . ?
C31 C30 H30 121.3 . . ?
C30 C31 C32 119.2(10) . . ?
C30 C31 C27 120.3(9) . . ?
C32 C31 C27 120.4(11) . . ?
N6 C32 O6 113.2(10) . . ?
N6 C32 C31 128.8(11) . . ?
O6 C32 C31 117.6(13) . . ?
N6 C32 O6’ 129.5(14) . . ?
O6 C32 O6’ 27.5(12) . . ?
C31 C32 O6’ 99.8(15) . . ?
C32 O6 C33 99.3(13) . . ?
C34 C33 O6 110.3(13) . . ?
C34 C33 H33A 109.4 . . ?
O6 C33 H33A 109.6 . . ?
C34 C33 H33B 109.7 . . ?
O6 C33 H33B 109.6 . . ?
H33A C33 H33B 108.1 . . ?
C33 C34 N6 92.7(13) . . ?
C33 C34 C35 114(2) . . ?
N6 C34 C35 112.8(17) . . ?
C33 C34 H34 111.9 . . ?

N6 C34 H34 111.9 . . ?
C35 C34 H34 111.8 . . ?
C34 C35 C36 118.9(17) . . ?
C34 C35 H35A 107.2 . . ?
C36 C35 H35A 107.5 . . ?
C34 C35 H35B 107.7 . . ?
C36 C35 H35B 107.8 . . ?
H35A C35 H35B 107.0 . . ?
C38 C36 C37 89.8(14) . . ?
C38 C36 C35 86.6(12) . . ?
C37 C36 C35 166(4) . . ?
C38 C36 H36 97.0 . . ?
C37 C36 H36 96.9 . . ?
C35 C36 H36 97.3 . . ?
C33' O6' C32 87.0(18) . . ?
O6' C33' C34' 114.8(17) . . ?
O6' C33' H33C 108.6 . . ?
C34' C33' H33C 108.7 . . ?
O6' C33' H33D 108.6 . . ?
C34' C33' H33D 108.4 . . ?
H33C C33' H33D 107.5 . . ?
C35' C34' N6 112.7(17) . . ?
C35' C34' C33' 105.1(18) . . ?
N6 C34' C33' 104.5(14) . . ?
C35' C34' H34' 111.5 . . ?
N6 C34' H34' 111.4 . . ?
C33' C34' H34' 111.3 . . ?
C36' C35' C34' 116.6(17) . . ?
C36' C35' H35C 108.2 . . ?
C34' C35' H35C 108.2 . . ?
C36' C35' H35D 108.1 . . ?
C34' C35' H35D 108.1 . . ?
H35C C35' H35D 107.3 . . ?
C38' C36' C35' 100.2(13) . . ?
C38' C36' C37' 97.5(15) . . ?
C35' C36' C37' 150(3) . . ?
C38' C36' H36' 100.1 . . ?
C35' C36' H36' 100.1 . . ?
C37' C36' H36' 100.6 . . ?
C28 C39 H39A 109.5 . . ?
C28 C39 H39B 109.5 . . ?
H39A C39 H39B 109.5 . . ?
C28 C39 H39C 109.4 . . ?
H39A C39 H39C 109.5 . . ?

H39B C39 H39C 109.5 . . ?
O7 C40 N7 110.1(9) . . ?
O7 C40 C44 123.6(10) . . ?
N7 C40 C44 126.2(10) . . ?
N7 C41 C42 124.3(11) . . ?
N7 C41 C52 123.6(14) . . ?
C42 C41 C52 112.1(13) . . ?
C43 C42 C41 112.1(13) . . ?
C43 C42 H42 123.9 . . ?
C41 C42 H42 123.9 . . ?
C42 C43 C44 124.6(13) . . ?
C42 C43 H43 117.7 . . ?
C44 C43 H43 117.7 . . ?
C45 C44 C40 123.2(12) . . ?
C45 C44 C43 121.5(11) . . ?
C40 C44 C43 115.2(10) . . ?
N8 C45 C44 132.3(11) . . ?
N8 C45 O8 114.7(10) . . ?
C44 C45 O8 113.0(12) . . ?
C47 C46 O8 103.3(9) . . ?
C47 C46 H46A 111.1 . . ?
O8 C46 H46A 111.1 . . ?
C47 C46 H46B 111.1 . . ?
O8 C46 H46B 111.1 . . ?
H46A C46 H46B 109.1 . . ?
C46 C47 N8 106.3(10) . . ?
C46 C47 C48 112.7(10) . . ?
N8 C47 C48 111.3(9) . . ?
C46 C47 H47 108.8 . . ?
N8 C47 H47 108.8 . . ?
C48 C47 H47 108.8 . . ?
C47 C48 C49 118.0(10) . . ?
C47 C48 H48A 107.8 . . ?
C49 C48 H48A 107.8 . . ?
C47 C48 H48B 107.8 . . ?
C49 C48 H48B 107.8 . . ?
H48A C48 H48B 107.1 . . ?
C51 C49 C50 112.6(13) . . ?
C51 C49 C48 113.8(14) . . ?
C50 C49 C48 99.0(12) . . ?
C51 C49 H49 110.3 . . ?
C50 C49 H49 110.4 . . ?
C48 C49 H49 110.3 . . ?
C49 C50 H50A 109.5 . . ?

```
C49 C50 H50B 109.5 . . ?
H50A C50 H50B 109.5 . . ?
C49 C50 H50C 109.4 . . ?
H50A C50 H50C 109.5 . . ?
H50B C50 H50C 109.5 . . ?
C49 C51 H51A 109.4 . . ?
C49 C51 H51B 109.4 . . ?
H51A C51 H51B 109.5 . . ?
C49 C51 H51C 109.5 . . ?
H51A C51 H51C 109.5 . . ?
H51B C51 H51C 109.5 . . ?
C41 C52 H52A 109.5 . . ?
C41 C52 H52B 109.4 . . ?
H52A C52 H52B 109.5 . . ?
C41 C52 H52C 109.5 . . ?
H52A C52 H52C 109.5 . . ?
H52B C52 H52C 109.5 . . ?

loop_
 _geom_torsion_atom_site_label_1
 _geom_torsion_atom_site_label_2
 _geom_torsion_atom_site_label_3
 _geom_torsion_atom_site_label_4
 _geom_torsion
 _geom_torsion_site_symmetry_1
 _geom_torsion_site_symmetry_2
 _geom_torsion_site_symmetry_3
 _geom_torsion_site_symmetry_4
 _geom_torsion_publ_flag
O2 Zn1 N1 C6 -4.7(9) . . . . ?
N3 Zn1 N1 C6 -122.6(9) . . . . ?
Cl1 Zn1 N1 C6 104.4(9) . . . . ?
Cl1' Zn1 N1 C6 100.2(11) . . . . ?
O2 Zn1 N1 C8' 161(2) . . . . ?
N3 Zn1 N1 C8' 44(2) . . . . ?
Cl1 Zn1 N1 C8' -89(2) . . . . ?
Cl1' Zn1 N1 C8' -94(2) . . . . ?
O2 Zn1 N1 C8 154.8(16) . . . . ?
N3 Zn1 N1 C8 36.9(17) . . . . ?
Cl1 Zn1 N1 C8 -96.1(16) . . . . ?
Cl1' Zn1 N1 C8 -100.2(18) . . . . ?
O2 Zn1 N3 C15 111.9(10) . . . . ?
N1 Zn1 N3 C15 -137.5(10) . . . . ?
Cl1 Zn1 N3 C15 -10.4(11) . . . . ?
```

Cl1’ Zn1 N3 C15 5.1(12) . . . . ?
O2 Zn1 N3 C14 -67.5(6) . . . . ?
N1 Zn1 N3 C14 43.1(6) . . . . ?
Cl1 Zn1 N3 C14 170.2(5) . . . . ?
Cl1’ Zn1 N3 C14 -174.3(7) . . . . ?
O4 Zn2 N4 C19 -2.5(9) . . . . ?
N2 Zn2 N4 C19 117.6(8) 2_656 . . . ?
Cl2 Zn2 N4 C19 -110.9(8) . . . . ?
O4 Zn2 N4 C21 176.3(7) . . . . ?
N2 Zn2 N4 C21 -63.6(8) 2_656 . . . ?
Cl2 Zn2 N4 C21 67.9(7) . . . . ?
O7 Zn3 N5 C27 -68.6(6) . . . . ?
N8 Zn3 N5 C27 40.0(6) . . . . ?
Cl3 Zn3 N5 C27 170.1(4) . . . . ?
O7 Zn3 N5 C28 114.9(8) . . . . ?
N8 Zn3 N5 C28 -136.5(8) . . . . ?
Cl3 Zn3 N5 C28 -6.4(9) . . . . ?
O5 Zn4 N7 C41 -117.8(10) 2_755 . . . ?
N6 Zn4 N7 C41 134.1(10) 2_755 . . . ?
Cl4 Zn4 N7 C41 4.4(11) . . . . ?
O5 Zn4 N7 C40 61.3(6) 2_755 . . . ?
N6 Zn4 N7 C40 -46.8(7) 2_755 . . . ?
Cl4 Zn4 N7 C40 -176.5(5) . . . . ?
O7 Zn3 N8 C45 -6.3(8) . . . . ?
N5 Zn3 N8 C45 -124.1(8) . . . . ?
Cl3 Zn3 N8 C45 102.1(8) . . . . ?
O7 Zn3 N8 C47 163.9(8) . . . . ?
N5 Zn3 N8 C47 46.0(9) . . . . ?
Cl3 Zn3 N8 C47 -87.8(8) . . . . ?
N1 Zn1 O2 C1 6.0(7) . . . . ?
N3 Zn1 O2 C1 133.9(7) . . . . ?
Cl1 Zn1 O2 C1 -98.7(7) . . . . ?
Cl1’ Zn1 O2 C1 -116.0(8) . . . . ?
N4 Zn2 O4 C14 1.4(8) . . . . ?
N2 Zn2 O4 C14 -130.0(8) 2_656 . . . ?
Cl2 Zn2 O4 C14 106.2(8) . . . . ?
N8 Zn3 O7 C40 8.4(8) . . . . ?
N5 Zn3 O7 C40 133.6(7) . . . . ?
Cl3 Zn3 O7 C40 -100.1(7) . . . . ?
Zn1 O2 C1 N2 178.1(4) . . . . ?
Zn1 O2 C1 C5 -1.3(13) . . . . ?
C2 N2 C1 O2 -178.3(9) . . . . ?
Zn2 N2 C1 O2 -0.1(8) 2_656 . . . ?
C2 N2 C1 C5 1.2(13) . . . . ?

Zn2 N2 C1 C5 179.3(6) 2_656 . . . ?
C1 N2 C2 C3 4.4(16) . . . . ?
Zn2 N2 C2 C3 -173.2(8) 2_656 . . . ?
C1 N2 C2 C13 177.4(8) . . . . ?
Zn2 N2 C2 C13 -0.2(15) 2_656 . . . ?
N2 C2 C3 C4 -8.8(17) . . . . ?
C13 C2 C3 C4 178.9(10) . . . . ?
C2 C3 C4 C5 8(2) . . . . ?
C3 C4 C5 C1 -2.6(19) . . . . ?
C3 C4 C5 C6 -178.7(9) . . . . ?
O2 C1 C5 C4 177.4(11) . . . . ?
N2 C1 C5 C4 -1.9(15) . . . . ?
O2 C1 C5 C6 -6.7(15) . . . . ?
N2 C1 C5 C6 174.0(8) . . . . ?
C8’ N1 C6 O1 0(2) . . . . ?
C8 N1 C6 O1 6.5(19) . . . . ?
Zn1 N1 C6 O1 170.2(10) . . . . ?
C8’ N1 C6 O1’ 23(2) . . . . ?
C8 N1 C6 O1’ 29(2) . . . . ?
Zn1 N1 C6 O1’ -167.0(14) . . . . ?
C8’ N1 C6 C5 -171.2(17) . . . . ?
C8 N1 C6 C5 -164.8(16) . . . . ?
Zn1 N1 C6 C5 -1.2(17) . . . . ?
C4 C5 C6 N1 -176.0(13) . . . . ?
C1 C5 C6 N1 8.1(18) . . . . ?
C4 C5 C6 O1 12.9(17) . . . . ?
C1 C5 C6 O1 -163.0(12) . . . . ?
C4 C5 C6 O1’ -10(2) . . . . ?
C1 C5 C6 O1’ 174.1(15) . . . . ?
N1 C6 O1 C7 8(2) . . . . ?
O1’ C6 O1 C7 -90(6) . . . . ?
C5 C6 O1 C7 -178.9(13) . . . . ?
C6 O1 C7 C8 -19(3) . . . . ?
O1 C7 C8 C9 133(2) . . . . ?
O1 C7 C8 N1 20(2) . . . . ?
C6 N1 C8 C7 -17(2) . . . . ?
C8’ N1 C8 C7 41(16) . . . . ?
Zn1 N1 C8 C7 -178.7(11) . . . . ?
C6 N1 C8 C9 -145.1(16) . . . . ?
C8’ N1 C8 C9 -87(17) . . . . ?
Zn1 N1 C8 C9 54(3) . . . . ?
C7 C8 C9 C10 151(3) . . . . ?
N1 C8 C9 C10 -98(2) . . . . ?
C8 C9 C10 C11 -34(3) . . . . ?

C8 C9 C10 C12 -178(2) . . . . ?
N1 C6 O1' C7' 1(3) . . . . ?
O1 C6 O1' C7' 92(6) . . . . ?
C5 C6 O1' C7' -167(2) . . . . ?
C6 O1' C7' C8' -22(3) . . . . ?
C6 N1 C8' C7' -33(3) . . . . ?
C8 N1 C8' C7' -157(18) . . . . ?
Zn1 N1 C8' C7' 159.2(15) . . . . ?
C6 N1 C8' C9' 148(4) . . . . ?
C8 N1 C8' C9' 25(13) . . . . ?
Zn1 N1 C8' C9' -19(5) . . . . ?
O1' C7' C8' N1 34(3) . . . . ?
O1' C7' C8' C9' -147(4) . . . . ?
N1 C8' C9' C10' 102(5) . . . . ?
C7' C8' C9' C10' -76(6) . . . . ?
C8' C9' C10' C11' -162(5) . . . . ?
C8' C9' C10' C12' -43(10) . . . . ?
Zn2 O4 C14 N3 179.5(5) . . . . ?
Zn2 O4 C14 C18 -1.9(14) . . . . ?
C15 N3 C14 O4 177.6(10) . . . . ?
Zn1 N3 C14 O4 -2.9(9) . . . . ?
C15 N3 C14 C18 -1.0(14) . . . . ?
Zn1 N3 C14 C18 178.4(7) . . . . ?
C14 N3 C15 C16 0.7(17) . . . . ?
Zn1 N3 C15 C16 -178.6(8) . . . . ?
C14 N3 C15 C26 -179.1(8) . . . . ?
Zn1 N3 C15 C26 1.6(17) . . . . ?
N3 C15 C16 C17 -3(2) . . . . ?
C26 C15 C16 C17 176.9(14) . . . . ?
C15 C16 C17 C18 6(3) . . . . ?
C16 C17 C18 C14 -6(2) . . . . ?
C16 C17 C18 C19 176.2(11) . . . . ?
O4 C14 C18 C17 -174.8(13) . . . . ?
N3 C14 C18 C17 3.7(17) . . . . ?
O4 C14 C18 C19 2.9(16) . . . . ?
N3 C14 C18 C19 -178.6(9) . . . . ?
C21 N4 C19 O3 3.1(12) . . . . ?
Zn2 N4 C19 O3 -177.9(6) . . . . ?
C21 N4 C19 C18 -174.8(9) . . . . ?
Zn2 N4 C19 C18 4.1(15) . . . . ?
C20' O3 C19 N4 -5.3(16) . . . . ?
C20 O3 C19 N4 18.3(16) . . . . ?
C20' O3 C19 C18 172.9(13) . . . . ?
C20 O3 C19 C18 -163.5(14) . . . . ?

C17 C18 C19 N4 173.5(14) . . . . ?
C14 C18 C19 N4 -4.3(17) . . . . ?
C17 C18 C19 O3 -4.5(17) . . . . ?
C14 C18 C19 O3 177.8(9) . . . . ?
C19 N4 C21 C22 -120.1(14) . . . . ?
Zn2 N4 C21 C22 61.0(14) . . . . ?
C19 N4 C21 C22' -134.1(16) . . . . ?
Zn2 N4 C21 C22' 46.9(17) . . . . ?
C19 N4 C21 C20 -22.6(13) . . . . ?
Zn2 N4 C21 C20 158.4(11) . . . . ?
C19 N4 C21 C20' 0.1(14) . . . . ?
Zn2 N4 C21 C20' -178.8(10) . . . . ?
N4 C21 C20 O3 29.7(17) . . . . ?
C22 C21 C20 O3 140.4(19) . . . . ?
C22' C21 C20 O3 155.2(16) . . . . ?
C20' C21 C20 O3 -65(5) . . . . ?
C19 O3 C20 C21 -29.9(19) . . . . ?
C20' O3 C20 C21 76(3) . . . . ?
N4 C21 C22 C23 -90(2) . . . . ?
C22' C21 C22 C23 46(6) . . . . ?
C20 C21 C22 C23 169(2) . . . . ?
C20' C21 C22 C23 158.5(19) . . . . ?
C21 C22 C23 C25 -178.6(19) . . . . ?
C21 C22 C23 C24 -78(3) . . . . ?
C19 O3 C20' C21 4.7(17) . . . . ?
C20 O3 C20' C21 -77(3) . . . . ?
N4 C21 C20' O3 -2.8(16) . . . . ?
C22 C21 C20' O3 114.4(18) . . . . ?
C22' C21 C20' O3 132.7(15) . . . . ?
C20 C21 C20' O3 87(7) . . . . ?
N4 C21 C22' C23' -125(2) . . . . ?
C22 C21 C22' C23' -176(8) . . . . ?
C20 C21 C22' C23' 125(3) . . . . ?
C20' C21 C22' C23' 109(3) . . . . ?
C21 C22' C23' C25' -156(4) . . . . ?
C21 C22' C23' C24' -67(4) . . . . ?
Zn4 O5 C27 N5 -179.7(4) 2_755 . . . ?
Zn4 O5 C27 C31 -0.5(15) 2_755 . . . ?
C28 N5 C27 O5 175.7(8) . . . . ?
Zn3 N5 C27 O5 -1.3(9) . . . . ?
C28 N5 C27 C31 -3.6(12) . . . . ?
Zn3 N5 C27 C31 179.4(6) . . . . ?
C27 N5 C28 C29 1.1(13) . . . . ?
Zn3 N5 C28 C29 177.3(7) . . . . ?

C27 N5 C28 C39 176.4(8) . . . . ?
Zn3 N5 C28 C39 -7.4(13) . . . . ?
N5 C28 C29 C30 -0.4(16) . . . . ?
C39 C28 C29 C30 -175.6(10) . . . . ?
C28 C29 C30 C31 2.3(18) . . . . ?
C29 C30 C31 C32 178.7(9) . . . . ?
C29 C30 C31 C27 -4.8(17) . . . . ?
O5 C27 C31 C30 -173.6(11) . . . . ?
N5 C27 C31 C30 5.5(14) . . . . ?
O5 C27 C31 C32 2.8(16) . . . . ?
N5 C27 C31 C32 -178.0(8) . . . . ?
C34 N6 C32 O6 4(2) . . . . ?
C34' N6 C32 O6 -3.3(16) . . . . ?
Zn4 N6 C32 O6 170.7(8) 2_755 . . . ?
C34 N6 C32 C31 -168.7(15) . . . . ?
C34' N6 C32 C31 -175.9(14) . . . . ?
Zn4 N6 C32 C31 -2.0(17) 2_755 . . . ?
C34 N6 C32 O6' 30(3) . . . . ?
C34' N6 C32 O6' 23(2) . . . . ?
Zn4 N6 C32 O6' -163.1(17) 2_755 . . . ?
C30 C31 C32 N6 175.1(12) . . . . ?
C27 C31 C32 N6 -1.4(17) . . . . ?
C30 C31 C32 O6 2.7(16) . . . . ?
C27 C31 C32 O6 -173.8(10) . . . . ?
C30 C31 C32 O6' -19.6(18) . . . . ?
C27 C31 C32 O6' 163.9(15) . . . . ?
N6 C32 O6 C33 6.4(15) . . . . ?
C31 C32 O6 C33 179.9(12) . . . . ?
O6' C32 O6 C33 -126(4) . . . . ?
C32 O6 C33 C34 -15(2) . . . . ?
O6 C33 C34 N6 15(2) . . . . ?
O6 C33 C34 C35 131.3(18) . . . . ?
C32 N6 C34 C33 -13(2) . . . . ?
C34' N6 C34 C33 6(4) . . . . ?
Zn4 N6 C34 C33 179.1(13) 2_755 . . . ?
C32 N6 C34 C35 -130.9(18) . . . . ?
C34' N6 C34 C35 -112(5) . . . . ?
Zn4 N6 C34 C35 61(2) 2_755 . . . ?
C33 C34 C35 C36 72(3) . . . . ?
N6 C34 C35 C36 176(2) . . . . ?
C34 C35 C36 C38 172(3) . . . . ?
C34 C35 C36 C37 96(9) . . . . ?
N6 C32 O6' C33' -15(3) . . . . ?
O6 C32 O6' C33' 47(3) . . . . ?

C31 C32 O6’ C33’ -179.7(16) . . . . ?
C32 O6’ C33’ C34’ -2(3) . . . . ?
C32 N6 C34’ C35’ -132.0(16) . . . . ?
C34 N6 C34’ C35’ 64(4) . . . . ?
Zn4 N6 C34’ C35’ 55(2) 2_755 . . . ?
C32 N6 C34’ C33’ -19(2) . . . . ?
C34 N6 C34’ C33’ 177(6) . . . . ?
Zn4 N6 C34’ C33’ 168.7(12) 2_755 . . . ?
O6’ C33’ C34’ C35’ 132(2) . . . . ?
O6’ C33’ C34’ N6 13(3) . . . . ?
N6 C34’ C35’ C36’ -64(3) . . . . ?
C33’ C34’ C35’ C36’ -177.1(19) . . . . ?
C34’ C35’ C36’ C38’ -77(2) . . . . ?
C34’ C35’ C36’ C37’ 158(4) . . . . ?
Zn3 O7 C40 N7 176.8(5) . . . . ?
Zn3 O7 C40 C44 -6.1(13) . . . . ?
C41 N7 C40 O7 -179.7(9) . . . . ?
Zn4 N7 C40 O7 1.0(9) . . . . ?
C41 N7 C40 C44 3.3(14) . . . . ?
Zn4 N7 C40 C44 -176.0(7) . . . . ?
C40 N7 C41 C42 1.0(18) . . . . ?
Zn4 N7 C41 C42 -180.0(9) . . . . ?
C40 N7 C41 C52 -177.7(9) . . . . ?
Zn4 N7 C41 C52 1.3(19) . . . . ?
N7 C41 C42 C43 -6(2) . . . . ?
C52 C41 C42 C43 172.4(11) . . . . ?
C41 C42 C43 C44 9(2) . . . . ?
O7 C40 C44 C45 -0.6(15) . . . . ?
N7 C40 C44 C45 176.0(9) . . . . ?
O7 C40 C44 C43 -178.0(10) . . . . ?
N7 C40 C44 C43 -1.4(15) . . . . ?
C42 C43 C44 C45 177.3(11) . . . . ?
C42 C43 C44 C40 -5.3(19) . . . . ?
C47 N8 C45 C44 -170.1(11) . . . . ?
Zn3 N8 C45 C44 2.5(17) . . . . ?
C47 N8 C45 O8 8.1(11) . . . . ?
Zn3 N8 C45 O8 -179.4(6) . . . . ?
C40 C44 C45 N8 2.5(19) . . . . ?
C43 C44 C45 N8 179.7(12) . . . . ?
C40 C44 C45 O8 -175.7(9) . . . . ?
C43 C44 C45 O8 1.5(15) . . . . ?
C46 O8 C45 N8 5.3(12) . . . . ?
C46 O8 C45 C44 -176.2(9) . . . . ?
C45 O8 C46 C47 -16.3(11) . . . . ?

```
O8 C46 C47 N8 21.0(11) . . . . ?
O8 C46 C47 C48 143.1(9) . . . . ?
C45 N8 C47 C46 -18.7(11) . . . . ?
Zn3 N8 C47 C46 170.2(7) . . . . ?
C45 N8 C47 C48 -141.8(10) . . . . ?
Zn3 N8 C47 C48 47.1(13) . . . . ?
C46 C47 C48 C49 168.7(10) . . . . ?
N8 C47 C48 C49 -72.0(14) . . . . ?
C47 C48 C49 C51 -72.3(16) . . . . ?
C47 C48 C49 C50 168.0(11) . . . . ?

_diffrn_measured_fraction_theta_max    1.000
_diffrn_reflns_theta_full              26.00
_diffrn_measured_fraction_theta_full   1.000
_refine_diff_density_max    0.357
_refine_diff_density_min   -0.380
_refine_diff_density_rms    0.070

data_mo_compound_15

_audit_creation_method            SHELXL-97
_chemical_name_systematic
;
 ?
;
_chemical_name_common             ?
_chemical_melting_point           ?
_chemical_formula_moiety          ?
_chemical_formula_sum
 'C64 H60 Cl4 N8 O8 Zn4'
_chemical_formula_weight          1472.48

loop_
 _atom_type_symbol
 _atom_type_description
 _atom_type_scat_dispersion_real
 _atom_type_scat_dispersion_imag
 _atom_type_scat_source
 'C'  'C'   0.0033   0.0016
 'International Tables Vol C Tables 4.2.6.8 and 6.1.1.4'
 'H'  'H'   0.0000   0.0000
 'International Tables Vol C Tables 4.2.6.8 and 6.1.1.4'
 'N'  'N'   0.0061   0.0033
 'International Tables Vol C Tables 4.2.6.8 and 6.1.1.4'
```

```
 'O'  'O'   0.0106   0.0060
 'International Tables Vol C Tables 4.2.6.8 and 6.1.1.4'
 'Zn'  'Zn'   0.2839   1.4301
 'International Tables Vol C Tables 4.2.6.8 and 6.1.1.4'
 'Cl'  'Cl'   0.1484   0.1585
 'International Tables Vol C Tables 4.2.6.8 and 6.1.1.4'

_symmetry_cell_setting            Tetragonal
_symmetry_space_group_name_H-M    P4(1)

loop_
 _symmetry_equiv_pos_as_xyz
 'x, y, z'
 '-y, x, z+1/4'
 'y, -x, z+3/4'
 '-x, -y, z+1/2'

_cell_length_a                    16.1089(14)
_cell_length_b                    16.1089(14)
_cell_length_c                    25.737(2)
_cell_angle_alpha                 90.00
_cell_angle_beta                  90.00
_cell_angle_gamma                 90.00
_cell_volume                      6678.7(10)
_cell_formula_units_Z             4
_cell_measurement_temperature     143(2)
_cell_measurement_reflns_used     7089
_cell_measurement_theta_min       2.39
_cell_measurement_theta_max       26.18

_exptl_crystal_description        PRISM
_exptl_crystal_colour             colourless
_exptl_crystal_size_max           0.28
_exptl_crystal_size_mid           0.15
_exptl_crystal_size_min           0.10
_exptl_crystal_density_meas       ?
_exptl_crystal_density_diffrn     1.464
_exptl_crystal_density_method     'not measured'
_exptl_crystal_F_000              3008
_exptl_absorpt_coefficient_mu     1.638
_exptl_absorpt_correction_type    multi-scan
_exptl_absorpt_correction_T_min   0.6570
_exptl_absorpt_correction_T_max   0.8534
_exptl_absorpt_process_details    sadabs
```

```
_exptl_special_details
;
 ?
;

_diffrn_ambient_temperature       143(2)
_diffrn_radiation_wavelength      0.71073
_diffrn_radiation_type            MoK\a
_diffrn_radiation_source          'fine-focus sealed tube'
_diffrn_radiation_monochromator   graphite
_diffrn_measurement_device_type   'Bruker APEX-II CCD'
_diffrn_measurement_method        '\f and \w scans'
_diffrn_detector_area_resol_mean  ?
_diffrn_reflns_number             45019
_diffrn_reflns_av_R_equivalents   0.0452
_diffrn_reflns_av_sigmaI/netI     0.0720
_diffrn_reflns_limit_h_min        -21
_diffrn_reflns_limit_h_max        13
_diffrn_reflns_limit_k_min        -21
_diffrn_reflns_limit_k_max        21
_diffrn_reflns_limit_l_min        -34
_diffrn_reflns_limit_l_max        34
_diffrn_reflns_theta_min          1.79
_diffrn_reflns_theta_max          28.71
_reflns_number_total              17062
_reflns_number_gt                 13065
_reflns_threshold_expression      >2sigma(I)

_computing_data_collection        'Bruker APEX2'
_computing_cell_refinement        'Bruker SAINT'
_computing_data_reduction         'Bruker SAINT'
_computing_structure_solution     'SHELXS-97 (Sheldrick, 2008)'
_computing_structure_refinement   'SHELXL-97 (Sheldrick, 2008)'
_computing_molecular_graphics     'Bruker SHELXTL'
_computing_publication_material   'Bruker SHELXTL'

_refine_special_details
;
 Refinement of F^2^ against ALL reflections.  The weighted R-factor wR and
 goodness of fit S are based on F^2^, conventional R-factors R are based
 on F, with F set to zero for negative F^2^. The threshold expression of
 F^2^ > 2sigma(F^2^) is used only for calculating R-factors(gt) etc. and is
 not relevant to the choice of reflections for refinement.  R-factors based
```

```
 on F^2^ are statistically about twice as large as those based on F, and R-
 factors based on ALL data will be even larger.
;

_refine_ls_structure_factor_coef  Fsqd
_refine_ls_matrix_type            full
_refine_ls_weighting_scheme       calc
_refine_ls_weighting_details
 'calc w=1/[\s^2^(Fo^2^)+(0.0236P)^2^+0.0000P] where P=(Fo^2^+2Fc^2^)/3'
_atom_sites_solution_primary      direct
_atom_sites_solution_secondary    difmap
_atom_sites_solution_hydrogens    geom
_refine_ls_hydrogen_treatment     constr
_refine_ls_extinction_method      none
_refine_ls_extinction_coef        ?
_refine_ls_abs_structure_details
 'Flack H D (1983), Acta Cryst. A39, 876-881'
_refine_ls_abs_structure_Flack    0.010(6)
_chemical_absolute_configuration  ad
_refine_ls_number_reflns          17062
_refine_ls_number_parameters      797
_refine_ls_number_restraints      1
_refine_ls_R_factor_all           0.0613
_refine_ls_R_factor_gt            0.0394
_refine_ls_wR_factor_ref          0.0765
_refine_ls_wR_factor_gt           0.0710
_refine_ls_goodness_of_fit_ref    0.971
_refine_ls_restrained_S_all       0.971
_refine_ls_shift/su_max           0.007
_refine_ls_shift/su_mean          0.000

# SQUEEZE RESULTS (APPEND TO CIF)
# Note: Data are Listed for all Voids in the P1 Unit Cell
# i.e. Centre of Gravity, Solvent Accessible Volume,
# Recovered number of Electrons in the Void and
# Details about the Squeezed Material
loop_
  _platon_squeeze_void_nr
  _platon_squeeze_void_average_x
  _platon_squeeze_void_average_y
  _platon_squeeze_void_average_z
  _platon_squeeze_void_volume
  _platon_squeeze_void_count_electrons
  _platon_squeeze_void_content
```

```
   1  0.137  0.232  0.962          8          1 ' '
   2  0.291  0.640  0.338        117         27 ' '
   3  0.360  0.291  0.588        117         27 ' '
   4  0.232  0.863  0.712          8          1 ' '
   5  0.266  0.388  0.737          9          0 ' '
   6  0.388  0.734  0.487          9          0 ' '
   7  0.640  0.709  0.088        117         27 ' '
   8  0.709  0.360  0.838        118         27 ' '
   9  0.612  0.266  0.987          9          0 ' '
  10  0.734  0.612  0.237          9          0 ' '
  11  0.768  0.137  0.212          8          1 ' '
  12  0.863  0.768  0.462          8          1 ' '
_platon_squeeze_details
;
;


loop_
 _atom_site_label
 _atom_site_type_symbol
 _atom_site_fract_x
 _atom_site_fract_y
 _atom_site_fract_z
 _atom_site_U_iso_or_equiv
 _atom_site_adp_type
 _atom_site_occupancy
 _atom_site_symmetry_multiplicity
 _atom_site_calc_flag
 _atom_site_refinement_flags
 _atom_site_disorder_assembly
 _atom_site_disorder_group
Zn1 Zn 0.31511(2) -0.03619(2) 0.230413(14) 0.02540(8) Uani 1 1 d . . .
Zn2 Zn 0.07319(2) 0.07968(2) 0.186196(14) 0.02415(8) Uani 1 1 d . . .
Zn3 Zn 0.17314(2) 0.33160(2) 0.213360(13) 0.02597(9) Uani 1 1 d . . .
Zn4 Zn 0.41678(2) 0.21661(2) 0.180224(14) 0.02851(9) Uani 1 1 d . . .
Cl1 Cl 0.28346(6) -0.16790(5) 0.25125(3) 0.0364(2) Uani 1 1 d . . .
Cl2 Cl -0.05997(5) 0.08499(6) 0.16184(4) 0.0376(2) Uani 1 1 d . . .
Cl3 Cl 0.18444(7) 0.47005(5) 0.22805(4) 0.0490(2) Uani 1 1 d . . .
Cl4 Cl 0.55029(5) 0.17876(6) 0.16805(4) 0.0446(2) Uani 1 1 d . . .
N1 N 0.39145(15) -0.04276(16) 0.16924(10) 0.0254(6) Uani 1 1 d . . .
N2 N 0.15499(15) 0.07180(15) 0.12528(10) 0.0227(5) Uani 1 1 d . . .
N3 N 0.07780(15) 0.01156(15) 0.25208(10) 0.0247(6) Uani 1 1 d . . .
N4 N 0.16796(15) 0.26452(16) 0.27980(10) 0.0225(5) Uani 1 1 d . . .
N5 N 0.10897(16) 0.34178(16) 0.14615(10) 0.0268(6) Uani 1 1 d . . .
```

N6 N 0.36074(16) 0.25570(16) 0.11405(10) 0.0276(6) Uani 1 1 d . . .
N7 N 0.42436(16) 0.29126(16) 0.24280(11) 0.0291(6) Uani 1 1 d . . .
N8 N 0.35467(15) 0.03849(16) 0.28926(10) 0.0256(6) Uani 1 1 d . . .
O1 O 0.43393(14) -0.01151(15) 0.08892(9) 0.0344(5) Uani 1 1 d . . .
O2 O 0.22221(13) 0.00824(13) 0.18930(8) 0.0276(5) Uani 1 1 d . . .
O3 O 0.12183(15) -0.01452(14) 0.33340(9) 0.0358(6) Uani 1 1 d . . .
O4 O 0.10839(13) 0.18308(12) 0.22091(8) 0.0265(5) Uani 1 1 d . . .
O5 O 0.09922(13) 0.36869(16) 0.06099(9) 0.0370(6) Uani 1 1 d . . .
O6 O 0.27007(13) 0.28436(14) 0.17672(8) 0.0301(5) Uani 1 1 d . . .
O7 O 0.44283(17) 0.31471(14) 0.32789(10) 0.0406(6) Uani 1 1 d . . .
O8 O 0.36618(13) 0.12535(13) 0.22108(8) 0.0283(5) Uani 1 1 d . . .
C1 C 0.22601(18) 0.03525(18) 0.14305(12) 0.0223(6) Uani 1 1 d . . .
C2 C 0.1495(2) 0.1016(2) 0.07621(12) 0.0288(7) Uani 1 1 d . . .
C3 C 0.2163(2) 0.0962(2) 0.04260(13) 0.0378(8) Uani 1 1 d . . .
H3 H 0.2119 0.1170 0.0082 0.045 Uiso 1 1 calc R . .
C4 C 0.2890(2) 0.0608(2) 0.05914(13) 0.0343(8) Uani 1 1 d . . .
H4 H 0.3349 0.0577 0.0360 0.041 Uiso 1 1 calc R . .
C5 C 0.2967(2) 0.02920(19) 0.10949(12) 0.0256(7) Uani 1 1 d . . .
C6 C 0.37370(19) -0.0091(2) 0.12556(12) 0.0263(7) Uani 1 1 d . . .
C7 C 0.5067(2) -0.0468(3) 0.11434(14) 0.0399(9) Uani 1 1 d . . .
H7A H 0.5507 -0.0043 0.1184 0.048 Uiso 1 1 calc R . .
H7B H 0.5292 -0.0937 0.0939 0.048 Uiso 1 1 calc R . .
C8 C 0.47600(19) -0.0764(2) 0.16720(12) 0.0280(7) Uani 1 1 d . . .
H8 H 0.4738 -0.1385 0.1677 0.034 Uiso 1 1 calc R . .
C9 C 0.52787(19) -0.0450(2) 0.21352(14) 0.0350(8) Uani 1 1 d . . .
H9A H 0.5251 0.0163 0.2151 0.042 Uiso 1 1 calc R . .
H9B H 0.5047 -0.0674 0.2463 0.042 Uiso 1 1 calc R . .
C10 C 0.6172(2) -0.0722(2) 0.20784(14) 0.0357(8) Uani 1 1 d . . .
C11 C 0.6775(2) -0.0188(2) 0.18952(16) 0.0441(9) Uani 1 1 d . . .
H11 H 0.6639 0.0375 0.1826 0.053 Uiso 1 1 calc R . .
C12 C 0.7575(2) -0.0468(3) 0.18115(18) 0.0517(11) Uani 1 1 d . . .
H12 H 0.7983 -0.0092 0.1686 0.062 Uiso 1 1 calc R . .
C13 C 0.7789(3) -0.1259(3) 0.1904(2) 0.0708(15) Uani 1 1 d . . .
H13 H 0.8333 -0.1450 0.1828 0.085 Uiso 1 1 calc R . .
C14 C 0.7200(3) -0.1794(3) 0.2115(3) 0.095(2) Uani 1 1 d . . .
H14 H 0.7348 -0.2347 0.2203 0.114 Uiso 1 1 calc R . .
C15 C 0.6409(2) -0.1519(3) 0.2193(2) 0.0726(17) Uani 1 1 d . . .
H15 H 0.6009 -0.1892 0.2330 0.087 Uiso 1 1 calc R . .
C16 C 0.0696(2) 0.1412(2) 0.05903(14) 0.0376(8) Uani 1 1 d . . .
H16A H 0.0644 0.1962 0.0749 0.056 Uiso 1 1 calc R . .
H16B H 0.0227 0.1064 0.0699 0.056 Uiso 1 1 calc R . .
H16C H 0.0694 0.1467 0.0211 0.056 Uiso 1 1 calc R . .
C17 C 0.13589(18) 0.18850(18) 0.26779(12) 0.0210(6) Uani 1 1 d . . .
C18 C 0.19693(19) 0.2808(2) 0.32857(13) 0.0278(7) Uani 1 1 d . . .

C19 C 0.1941(2) 0.2220(2) 0.36667(13) 0.0333(8) Uani 1 1 d . . .
H19 H 0.2123 0.2349 0.4008 0.040 Uiso 1 1 calc R . .
C20 C 0.1650(2) 0.1443(2) 0.35527(13) 0.0330(8) Uani 1 1 d . . .
H20 H 0.1650 0.1028 0.3815 0.040 Uiso 1 1 calc R . .
C21 C 0.13523(19) 0.1248(2) 0.30574(12) 0.0265(7) Uani 1 1 d . . .
C22 C 0.10851(19) 0.04066(19) 0.29437(12) 0.0245(7) Uani 1 1 d . . .
C23 C 0.0866(2) -0.0929(2) 0.31589(13) 0.0357(8) Uani 1 1 d . . .
H23A H 0.0338 -0.1049 0.3341 0.043 Uiso 1 1 calc R . .
H23B H 0.1257 -0.1393 0.3219 0.043 Uiso 1 1 calc R . .
C24 C 0.0718(2) -0.07952(18) 0.25730(13) 0.0272(7) Uani 1 1 d . . .
H24 H 0.1177 -0.1062 0.2372 0.033 Uiso 1 1 calc R . .
C25 C -0.01065(19) -0.11370(19) 0.23894(14) 0.0326(8) Uani 1 1 d . . .
H25A H -0.0148 -0.1069 0.2008 0.039 Uiso 1 1 calc R . .
H25B H -0.0562 -0.0815 0.2551 0.039 Uiso 1 1 calc R . .
C26 C -0.0209(2) -0.2049(2) 0.25256(14) 0.0351(8) Uani 1 1 d . . .
C27 C -0.0930(3) -0.2327(3) 0.2757(2) 0.0650(14) Uani 1 1 d . . .
H27 H -0.1347 -0.1938 0.2850 0.078 Uiso 1 1 calc R . .
C28 C -0.1055(3) -0.3163(4) 0.2856(3) 0.097(2) Uani 1 1 d . . .
H28 H -0.1567 -0.3347 0.2998 0.117 Uiso 1 1 calc R . .
C29 C -0.0447(4) -0.3718(3) 0.2748(2) 0.0784(18) Uani 1 1 d . . .
H29 H -0.0540 -0.4291 0.2812 0.094 Uiso 1 1 calc R . .
C30 C 0.0302(3) -0.3466(2) 0.25466(17) 0.0605(13) Uani 1 1 d . . .
H30 H 0.0735 -0.3856 0.2488 0.073 Uiso 1 1 calc R . .
C31 C 0.0415(3) -0.2630(2) 0.24301(15) 0.0466(10) Uani 1 1 d . . .
H31 H 0.0925 -0.2452 0.2283 0.056 Uiso 1 1 calc R . .
C32 C 0.2307(2) 0.3651(2) 0.33880(14) 0.0386(8) Uani 1 1 d . . .
H32A H 0.2460 0.3698 0.3755 0.058 Uiso 1 1 calc R . .
H32B H 0.1885 0.4068 0.3304 0.058 Uiso 1 1 calc R . .
H32C H 0.2799 0.3742 0.3172 0.058 Uiso 1 1 calc R . .
C33 C 0.2840(2) 0.28551(19) 0.12824(12) 0.0270(7) Uani 1 1 d . . .
C34 C 0.3854(2) 0.2571(2) 0.06370(14) 0.0346(8) Uani 1 1 d . . .
C35 C 0.3352(2) 0.2885(2) 0.02524(14) 0.0409(9) Uani 1 1 d . . .
H35 H 0.3541 0.2903 -0.0097 0.049 Uiso 1 1 calc R . .
C36 C 0.2565(2) 0.3176(2) 0.03796(13) 0.0369(8) Uani 1 1 d . . .
H36 H 0.2215 0.3392 0.0115 0.044 Uiso 1 1 calc R . .
C37 C 0.2286(2) 0.31543(19) 0.08884(12) 0.0273(7) Uani 1 1 d . . .
C38 C 0.1449(2) 0.34211(19) 0.10144(12) 0.0279(7) Uani 1 1 d . . .
C39 C 0.0170(2) 0.3865(3) 0.08090(15) 0.0458(10) Uani 1 1 d . . .
H39A H -0.0242 0.3469 0.0668 0.055 Uiso 1 1 calc R . .
H39B H -0.0002 0.4436 0.0717 0.055 Uiso 1 1 calc R . .
C40 C 0.02503(19) 0.3768(2) 0.13970(13) 0.0291(7) Uani 1 1 d . . .
H40 H 0.0228 0.4328 0.1564 0.035 Uiso 1 1 calc R . .
C41 C -0.0408(2) 0.3212(2) 0.16394(15) 0.0395(9) Uani 1 1 d . . .
H41A H -0.0316 0.3185 0.2019 0.047 Uiso 1 1 calc R . .

H41B H -0.0350 0.2644 0.1498 0.047 Uiso 1 1 calc R . .
C42 C -0.1290(2) 0.3524(3) 0.15348(15) 0.0422(9) Uani 1 1 d . . .
C43 C -0.1489(3) 0.4363(3) 0.1566(2) 0.0614(13) Uani 1 1 d . . .
H43 H -0.1073 0.4754 0.1657 0.074 Uiso 1 1 calc R . .
C44 C -0.2289(3) 0.4632(4) 0.1463(2) 0.0768(17) Uani 1 1 d . . .
H44 H -0.2419 0.5207 0.1484 0.092 Uiso 1 1 calc R . .
C45 C -0.2885(3) 0.4083(5) 0.13350(19) 0.0789(18) Uani 1 1 d . . .
H45 H -0.3432 0.4275 0.1267 0.095 Uiso 1 1 calc R . .
C46 C -0.2712(3) 0.3245(4) 0.13012(18) 0.0695(15) Uani 1 1 d . . .
H46 H -0.3136 0.2863 0.1210 0.083 Uiso 1 1 calc R . .
C47 C -0.1901(2) 0.2963(3) 0.14033(16) 0.0555(11) Uani 1 1 d . . .
H47 H -0.1775 0.2387 0.1382 0.067 Uiso 1 1 calc R . .
C48 C 0.4688(2) 0.2218(3) 0.05102(16) 0.0534(11) Uani 1 1 d . . .
H48A H 0.5117 0.2527 0.0699 0.080 Uiso 1 1 calc R . .
H48B H 0.4787 0.2264 0.0136 0.080 Uiso 1 1 calc R . .
H48C H 0.4706 0.1633 0.0613 0.080 Uiso 1 1 calc R . .
C49 C 0.37735(18) 0.11454(19) 0.27002(12) 0.0233(7) Uani 1 1 d . . .
C50 C 0.36551(19) 0.0203(2) 0.34025(13) 0.0287(7) Uani 1 1 d . . .
C51 C 0.4001(2) 0.0762(2) 0.37389(14) 0.0362(8) Uani 1 1 d . . .
H51 H 0.4090 0.0612 0.4092 0.043 Uiso 1 1 calc R . .
C52 C 0.4222(2) 0.1541(2) 0.35671(14) 0.0366(8) Uani 1 1 d . . .
H52 H 0.4456 0.1933 0.3801 0.044 Uiso 1 1 calc R . .
C53 C 0.4101(2) 0.17489(19) 0.30482(13) 0.0280(7) Uani 1 1 d . . .
C54 C 0.42688(19) 0.2605(2) 0.28883(13) 0.0297(7) Uani 1 1 d . . .
C55 C 0.4651(3) 0.3919(2) 0.30231(16) 0.0470(10) Uani 1 1 d . . .
H55A H 0.5261 0.3997 0.3020 0.056 Uiso 1 1 calc R . .
H55B H 0.4390 0.4399 0.3199 0.056 Uiso 1 1 calc R . .
C56 C 0.4316(2) 0.3820(2) 0.24703(14) 0.0338(8) Uani 1 1 d . . .
H56 H 0.3748 0.4070 0.2452 0.041 Uiso 1 1 calc R . .
C57 C 0.4854(2) 0.4204(2) 0.20529(16) 0.0415(9) Uani 1 1 d . . .
H57A H 0.4633 0.4064 0.1705 0.050 Uiso 1 1 calc R . .
H57B H 0.5427 0.3986 0.2078 0.050 Uiso 1 1 calc R . .
C58 C 0.4854(2) 0.5147(2) 0.21303(16) 0.0422(9) Uani 1 1 d . . .
C59 C 0.5528(3) 0.5543(2) 0.23610(18) 0.0538(11) Uani 1 1 d . . .
H59 H 0.6021 0.5241 0.2434 0.065 Uiso 1 1 calc R . .
C60 C 0.5479(3) 0.6375(3) 0.2483(2) 0.0669(14) Uani 1 1 d . . .
H60 H 0.5941 0.6647 0.2636 0.080 Uiso 1 1 calc R . .
C61 C 0.4755(3) 0.6816(3) 0.2381(2) 0.0676(14) Uani 1 1 d . . .
H61 H 0.4722 0.7387 0.2472 0.081 Uiso 1 1 calc R . .
C62 C 0.4090(3) 0.6434(3) 0.21532(18) 0.0597(12) Uani 1 1 d . . .
H62 H 0.3600 0.6740 0.2080 0.072 Uiso 1 1 calc R . .
C63 C 0.4135(3) 0.5606(2) 0.20305(16) 0.0497(10) Uani 1 1 d . . .
H63 H 0.3670 0.5340 0.1875 0.060 Uiso 1 1 calc R . .
C64 C 0.3387(2) -0.0635(2) 0.35913(14) 0.0426(9) Uani 1 1 d . . .

```
H64A H 0.2799 -0.0719 0.3508 0.064 Uiso 1 1 calc R . .
H64B H 0.3466 -0.0668 0.3968 0.064 Uiso 1 1 calc R . .
H64C H 0.3720 -0.1065 0.3421 0.064 Uiso 1 1 calc R . .

loop_
 _atom_site_aniso_label
 _atom_site_aniso_U_11
 _atom_site_aniso_U_22
 _atom_site_aniso_U_33
 _atom_site_aniso_U_23
 _atom_site_aniso_U_13
 _atom_site_aniso_U_12
Zn1 0.02298(18) 0.0330(2) 0.02020(17) 0.00039(15) 0.00113(15) -0.00153(16)
Zn2 0.02717(18) 0.02463(18) 0.02065(18) 0.00045(15) -0.00107(15) -0.00456(15)
Zn3 0.02799(19) 0.03000(19) 0.01992(18) 0.00048(15) -0.00064(16) 0.00299(16)
Zn4 0.0324(2) 0.02673(19) 0.0264(2) -0.00040(16) 0.00099(17) -0.00169(17)
Cl1 0.0460(5) 0.0334(4) 0.0299(4) 0.0030(4) -0.0022(4) -0.0046(4)
Cl2 0.0293(4) 0.0434(5) 0.0402(5) 0.0041(4) -0.0077(4) -0.0105(4)
Cl3 0.0833(7) 0.0294(4) 0.0344(5) 0.0003(4) -0.0139(5) 0.0021(5)
Cl4 0.0331(5) 0.0545(6) 0.0463(6) -0.0011(5) 0.0045(4) 0.0030(4)
N1 0.0205(13) 0.0324(14) 0.0233(14) -0.0003(11) 0.0023(11) -0.0005(11)
N2 0.0298(14) 0.0196(12) 0.0186(13) -0.0032(10) -0.0001(11) -0.0025(11)
N3 0.0260(13) 0.0250(13) 0.0230(14) 0.0023(11) 0.0061(11) -0.0034(11)
N4 0.0202(12) 0.0292(14) 0.0181(13) -0.0023(11) 0.0007(10) 0.0009(11)
N5 0.0281(14) 0.0278(14) 0.0245(15) 0.0040(11) -0.0017(11) 0.0014(12)
N6 0.0289(15) 0.0298(15) 0.0241(14) -0.0012(12) 0.0065(11) -0.0015(12)
N7 0.0314(14) 0.0243(13) 0.0315(16) -0.0012(12) -0.0030(12) 0.0010(12)
N8 0.0258(14) 0.0294(14) 0.0215(14) -0.0001(11) 0.0004(11) 0.0014(12)
O1 0.0326(12) 0.0490(14) 0.0216(12) 0.0015(11) 0.0072(10) 0.0033(11)
O2 0.0252(11) 0.0359(12) 0.0217(11) 0.0023(10) -0.0005(9) 0.0004(9)
O3 0.0470(14) 0.0334(13) 0.0271(13) 0.0102(10) -0.0039(11) -0.0059(11)
O4 0.0324(12) 0.0258(11) 0.0215(12) 0.0008(9) -0.0011(9) -0.0042(9)
O5 0.0308(12) 0.0573(16) 0.0227(12) 0.0111(11) -0.0007(10) 0.0050(11)
O6 0.0296(11) 0.0413(13) 0.0194(12) 0.0028(10) 0.0031(9) 0.0059(10)
O7 0.0589(16) 0.0318(13) 0.0312(14) -0.0119(11) -0.0022(12) -0.0071(12)
O8 0.0323(12) 0.0294(11) 0.0232(13) -0.0007(9) -0.0028(9) -0.0037(10)
C1 0.0246(16) 0.0190(15) 0.0233(16) -0.0022(12) -0.0017(13) -0.0039(12)
C2 0.0356(18) 0.0279(17) 0.0230(17) -0.0036(14) -0.0025(14) 0.0026(14)
C3 0.055(2) 0.040(2) 0.0182(17) 0.0042(15) 0.0025(16) 0.0062(18)
C4 0.0375(19) 0.042(2) 0.0234(18) 0.0012(15) 0.0050(15) 0.0048(16)
C5 0.0314(17) 0.0264(16) 0.0190(16) -0.0007(13) 0.0013(13) 0.0019(14)
C6 0.0269(16) 0.0312(17) 0.0207(16) -0.0050(14) 0.0062(13) -0.0027(14)
C7 0.0300(18) 0.059(2) 0.031(2) 0.0025(18) 0.0050(16) 0.0043(18)
C8 0.0276(16) 0.0310(17) 0.0255(17) 0.0002(14) 0.0034(13) -0.0044(14)
```

C9	0.0284(17)	0.044(2)	0.0320(19)	-0.0025(16)	-0.0018(15)	-0.0102(16)
C10	0.0258(17)	0.047(2)	0.0341(19)	0.0001(17)	-0.0018(15)	-0.0081(16)
C11	0.038(2)	0.045(2)	0.049(2)	-0.0008(19)	0.0061(18)	-0.0106(17)
C12	0.0254(19)	0.070(3)	0.060(3)	0.003(2)	0.0017(19)	-0.019(2)
C13	0.028(2)	0.094(4)	0.091(4)	0.007(3)	-0.013(2)	0.008(2)
C14	0.038(3)	0.066(3)	0.182(7)	0.027(4)	-0.026(3)	0.000(2)
C15	0.032(2)	0.060(3)	0.126(5)	0.042(3)	-0.012(3)	-0.014(2)
C16	0.042(2)	0.040(2)	0.031(2)	0.0035(16)	-0.0056(16)	0.0053(17)
C17	0.0187(14)	0.0245(15)	0.0199(15)	-0.0046(12)	0.0028(12)	0.0018(12)
C18	0.0260(16)	0.0331(17)	0.0242(17)	-0.0069(14)	-0.0016(13)	0.0025(14)
C19	0.040(2)	0.042(2)	0.0180(16)	-0.0034(15)	-0.0048(14)	-0.0021(17)
C20	0.0350(19)	0.041(2)	0.0225(17)	0.0076(14)	-0.0009(14)	-0.0030(16)
C21	0.0258(16)	0.0334(17)	0.0202(16)	0.0012(14)	0.0028(13)	0.0012(14)
C22	0.0233(16)	0.0303(16)	0.0199(16)	0.0056(13)	0.0064(13)	-0.0011(13)
C23	0.046(2)	0.0285(17)	0.033(2)	0.0067(15)	0.0028(16)	-0.0069(16)
C24	0.0301(17)	0.0211(15)	0.0304(18)	0.0052(14)	0.0052(14)	-0.0008(13)
C25	0.0283(17)	0.0262(16)	0.043(2)	0.0049(15)	0.0000(16)	-0.0005(14)
C26	0.041(2)	0.0346(18)	0.0295(19)	0.0056(15)	-0.0051(16)	-0.0107(16)
C27	0.040(2)	0.056(3)	0.099(4)	0.039(3)	0.000(2)	-0.006(2)
C28	0.056(3)	0.091(4)	0.145(6)	0.077(4)	-0.029(3)	-0.032(3)
C29	0.094(4)	0.054(3)	0.087(4)	0.039(3)	-0.055(3)	-0.038(3)
C30	0.112(4)	0.029(2)	0.040(2)	-0.0019(18)	-0.025(3)	0.004(2)
C31	0.072(3)	0.0297(19)	0.038(2)	0.0019(16)	0.002(2)	0.0036(19)
C32	0.052(2)	0.0357(19)	0.0280(19)	-0.0083(16)	-0.0067(17)	-0.0049(18)
C33	0.0381(18)	0.0211(16)	0.0216(16)	0.0017(13)	0.0009(14)	-0.0054(14)
C34	0.044(2)	0.0356(19)	0.0241(18)	-0.0003(15)	0.0078(16)	-0.0021(17)
C35	0.044(2)	0.051(2)	0.0269(19)	-0.0010(17)	0.0098(16)	-0.0019(19)
C36	0.047(2)	0.039(2)	0.0245(18)	0.0014(15)	-0.0017(16)	0.0007(17)
C37	0.0325(18)	0.0263(16)	0.0230(17)	0.0016(13)	0.0003(14)	-0.0048(14)
C38	0.0354(18)	0.0279(17)	0.0205(16)	0.0044(13)	-0.0028(14)	-0.0032(15)
C39	0.036(2)	0.070(3)	0.031(2)	0.0147(19)	-0.0028(16)	0.012(2)
C40	0.0274(17)	0.0312(17)	0.0288(18)	0.0042(14)	-0.0039(14)	0.0048(14)
C41	0.0345(19)	0.044(2)	0.040(2)	0.0132(17)	-0.0018(16)	0.0036(17)
C42	0.0285(18)	0.064(3)	0.034(2)	0.0155(19)	0.0026(16)	0.0030(18)
C43	0.044(2)	0.066(3)	0.074(3)	0.028(3)	0.014(2)	0.016(2)
C44	0.060(3)	0.100(4)	0.070(4)	0.038(3)	0.020(3)	0.036(3)
C45	0.037(3)	0.152(6)	0.048(3)	0.028(3)	0.007(2)	0.025(3)
C46	0.037(2)	0.128(5)	0.043(3)	-0.006(3)	0.000(2)	-0.007(3)
C47	0.043(2)	0.085(3)	0.038(2)	0.004(2)	-0.0013(19)	-0.004(2)
C48	0.050(2)	0.075(3)	0.035(2)	0.006(2)	0.0148(19)	0.018(2)
C49	0.0171(14)	0.0297(17)	0.0233(16)	-0.0045(13)	0.0011(12)	0.0039(13)
C50	0.0273(16)	0.0361(18)	0.0226(17)	-0.0002(14)	0.0003(14)	0.0047(14)
C51	0.047(2)	0.041(2)	0.0216(17)	-0.0010(15)	-0.0035(15)	0.0063(17)
C52	0.040(2)	0.040(2)	0.0299(19)	-0.0091(16)	-0.0041(16)	-0.0009(17)

C53 0.0283(17) 0.0276(16) 0.0280(17) -0.0034(14) -0.0022(14) 0.0000(14)
C54 0.0245(16) 0.0316(18) 0.033(2) -0.0104(15) 0.0006(14) 0.0062(14)
C55 0.062(3) 0.035(2) 0.044(2) -0.0090(18) -0.001(2) -0.0113(19)
C56 0.0366(18) 0.0256(16) 0.039(2) -0.0036(15) 0.0000(16) -0.0014(15)
C57 0.041(2) 0.038(2) 0.045(2) 0.0010(17) 0.0017(18) -0.0041(17)
C58 0.050(2) 0.0354(19) 0.041(2) 0.0058(17) 0.0006(18) -0.0088(18)
C59 0.061(3) 0.043(2) 0.057(3) 0.004(2) -0.002(2) -0.015(2)
C60 0.084(4) 0.049(3) 0.068(3) 0.005(2) -0.002(3) -0.035(3)
C61 0.100(4) 0.033(2) 0.070(3) 0.003(2) 0.022(3) -0.012(3)
C62 0.075(3) 0.042(2) 0.062(3) 0.018(2) 0.010(3) -0.001(2)
C63 0.065(3) 0.043(2) 0.041(2) 0.0094(19) -0.005(2) -0.009(2)
C64 0.054(2) 0.048(2) 0.0257(19) 0.0059(17) -0.0058(17) -0.0087(19)

_geom_special_details
;
 All esds (except the esd in the dihedral angle between two l.s. planes)
 are estimated using the full covariance matrix. The cell esds are taken
 into account individually in the estimation of esds in distances, angles
 and torsion angles; correlations between esds in cell parameters are only
 used when they are defined by crystal symmetry. An approximate (isotropic)
 treatment of cell esds is used for estimating esds involving l.s. planes.
;

loop_
 _geom_bond_atom_site_label_1
 _geom_bond_atom_site_label_2
 _geom_bond_distance
 _geom_bond_site_symmetry_2
 _geom_bond_publ_flag
Zn1 O2 1.968(2) . ?
Zn1 N1 2.001(3) . ?
Zn1 N8 2.036(3) . ?
Zn1 Cl1 2.2471(9) . ?
Zn2 O4 1.973(2) . ?
Zn2 N3 2.021(3) . ?
Zn2 N2 2.052(2) . ?
Zn2 Cl2 2.2362(9) . ?
Zn3 O6 1.977(2) . ?
Zn3 N5 2.022(3) . ?
Zn3 N4 2.024(3) . ?
Zn3 Cl3 2.2694(10) . ?
Zn4 O8 1.983(2) . ?
Zn4 N7 2.014(3) . ?
Zn4 N6 2.028(3) . ?

Zn4 Cl4 2.2573(10) . ?
N1 C6 1.280(4) . ?
N1 C8 1.467(4) . ?
N2 C2 1.354(4) . ?
N2 C1 1.366(4) . ?
N3 C22 1.284(4) . ?
N3 C24 1.477(4) . ?
N4 C18 1.365(4) . ?
N4 C17 1.365(4) . ?
N5 C38 1.288(4) . ?
N5 C40 1.474(4) . ?
N6 C34 1.356(4) . ?
N6 C33 1.375(4) . ?
N7 C54 1.285(4) . ?
N7 C56 1.470(4) . ?
N8 C50 1.356(4) . ?
N8 C49 1.371(4) . ?
O1 C6 1.354(4) . ?
O1 C7 1.458(4) . ?
O2 C1 1.269(4) . ?
O3 C22 1.358(4) . ?
O3 C23 1.457(4) . ?
O4 C17 1.288(4) . ?
O5 C38 1.345(4) . ?
O5 C39 1.449(4) . ?
O6 C33 1.268(4) . ?
O7 C54 1.356(4) . ?
O7 C55 1.452(4) . ?
O8 C49 1.284(4) . ?
C1 C5 1.432(4) . ?
C2 C3 1.384(5) . ?
C2 C16 1.503(4) . ?
C3 C4 1.370(5) . ?
C3 H3 0.9500 . ?
C4 C5 1.398(4) . ?
C4 H4 0.9500 . ?
C5 C6 1.446(4) . ?
C7 C8 1.524(5) . ?
C7 H7A 0.9900 . ?
C7 H7B 0.9900 . ?
C8 C9 1.541(4) . ?
C8 H8 1.0000 . ?
C9 C10 1.511(5) . ?
C9 H9A 0.9900 . ?

C9 H9B 0.9900 . ?
C10 C15 1.372(5) . ?
C10 C11 1.379(5) . ?
C11 C12 1.384(5) . ?
C11 H11 0.9500 . ?
C12 C13 1.341(6) . ?
C12 H12 0.9500 . ?
C13 C14 1.392(7) . ?
C13 H13 0.9500 . ?
C14 C15 1.363(7) . ?
C14 H14 0.9500 . ?
C15 H15 0.9500 . ?
C16 H16A 0.9800 . ?
C16 H16B 0.9800 . ?
C16 H16C 0.9800 . ?
C17 C21 1.416(4) . ?
C18 C19 1.363(5) . ?
C18 C32 1.486(4) . ?
C19 C20 1.369(5) . ?
C19 H19 0.9500 . ?
C20 C21 1.397(5) . ?
C20 H20 0.9500 . ?
C21 C22 1.452(4) . ?
C23 C24 1.542(5) . ?
C23 H23A 0.9900 . ?
C23 H23B 0.9900 . ?
C24 C25 1.513(4) . ?
C24 H24 1.0000 . ?
C25 C26 1.520(4) . ?
C25 H25A 0.9900 . ?
C25 H25B 0.9900 . ?
C26 C27 1.379(5) . ?
C26 C31 1.396(5) . ?
C27 C28 1.386(6) . ?
C27 H27 0.9500 . ?
C28 C29 1.355(8) . ?
C28 H28 0.9500 . ?
C29 C30 1.375(7) . ?
C29 H29 0.9500 . ?
C30 C31 1.391(5) . ?
C30 H30 0.9500 . ?
C31 H31 0.9500 . ?
C32 H32A 0.9800 . ?
C32 H32B 0.9800 . ?

C32 H32C 0.9800 . ?
C33 C37 1.434(4) . ?
C34 C35 1.375(5) . ?
C34 C48 1.493(5) . ?
C35 C36 1.392(5) . ?
C35 H35 0.9500 . ?
C36 C37 1.385(4) . ?
C36 H36 0.9500 . ?
C37 C38 1.451(5) . ?
C39 C40 1.527(5) . ?
C39 H39A 0.9900 . ?
C39 H39B 0.9900 . ?
C40 C41 1.522(5) . ?
C40 H40 1.0000 . ?
C41 C42 1.530(5) . ?
C41 H41A 0.9900 . ?
C41 H41B 0.9900 . ?
C42 C47 1.379(6) . ?
C42 C43 1.391(6) . ?
C43 C44 1.386(6) . ?
C43 H43 0.9500 . ?
C44 C45 1.346(8) . ?
C44 H44 0.9500 . ?
C45 C46 1.381(8) . ?
C45 H45 0.9500 . ?
C46 C47 1.408(6) . ?
C46 H46 0.9500 . ?
C47 H47 0.9500 . ?
C48 H48A 0.9800 . ?
C48 H48B 0.9800 . ?
C48 H48C 0.9800 . ?
C49 C53 1.423(4) . ?
C50 C51 1.368(5) . ?
C50 C64 1.497(5) . ?
C51 C52 1.378(5) . ?
C51 H51 0.9500 . ?
C52 C53 1.391(5) . ?
C52 H52 0.9500 . ?
C53 C54 1.464(5) . ?
C55 C56 1.530(5) . ?
C55 H55A 0.9900 . ?
C55 H55B 0.9900 . ?
C56 C57 1.513(5) . ?
C56 H56 1.0000 . ?

C57 C58 1.532(5) . ?
C57 H57A 0.9900 . ?
C57 H57B 0.9900 . ?
C58 C59 1.393(5) . ?
C58 C63 1.397(5) . ?
C59 C60 1.377(6) . ?
C59 H59 0.9500 . ?
C60 C61 1.391(7) . ?
C60 H60 0.9500 . ?
C61 C62 1.368(6) . ?
C61 H61 0.9500 . ?
C62 C63 1.373(6) . ?
C62 H62 0.9500 . ?
C63 H63 0.9500 . ?
C64 H64A 0.9800 . ?
C64 H64B 0.9800 . ?
C64 H64C 0.9800 . ?

```
loop_
 _geom_angle_atom_site_label_1
 _geom_angle_atom_site_label_2
 _geom_angle_atom_site_label_3
 _geom_angle
 _geom_angle_site_symmetry_1
 _geom_angle_site_symmetry_3
 _geom_angle_publ_flag
O2 Zn1 N1 93.64(9) . . ?
O2 Zn1 N8 115.03(10) . . ?
N1 Zn1 N8 115.10(10) . . ?
O2 Zn1 Cl1 107.41(7) . . ?
N1 Zn1 Cl1 106.10(8) . . ?
N8 Zn1 Cl1 116.83(8) . . ?
O4 Zn2 N3 93.89(9) . . ?
O4 Zn2 N2 102.33(9) . . ?
N3 Zn2 N2 125.72(10) . . ?
O4 Zn2 Cl2 111.73(7) . . ?
N3 Zn2 Cl2 106.93(7) . . ?
N2 Zn2 Cl2 113.84(8) . . ?
O6 Zn3 N5 91.54(10) . . ?
O6 Zn3 N4 103.31(9) . . ?
N5 Zn3 N4 138.15(10) . . ?
O6 Zn3 Cl3 113.23(7) . . ?
N5 Zn3 Cl3 95.96(8) . . ?
N4 Zn3 Cl3 112.78(8) . . ?
```

O8 Zn4 N7 92.50(10) . . ?

O8 Zn4 N6 119.51(10) . . ?

N7 Zn4 N6 120.89(11) . . ?

O8 Zn4 Cl4 105.37(7) . . ?

N7 Zn4 Cl4 102.39(8) . . ?

N6 Zn4 Cl4 113.04(8) . . ?

C6 N1 C8 109.4(3) . . ?

C6 N1 Zn1 122.1(2) . . ?

C8 N1 Zn1 128.2(2) . . ?

C2 N2 C1 121.3(3) . . ?

C2 N2 Zn2 130.4(2) . . ?

C1 N2 Zn2 107.99(19) . . ?

C22 N3 C24 108.1(3) . . ?

C22 N3 Zn2 121.8(2) . . ?

C24 N3 Zn2 127.8(2) . . ?

C18 N4 C17 120.7(3) . . ?

C18 N4 Zn3 131.3(2) . . ?

C17 N4 Zn3 107.65(19) . . ?

C38 N5 C40 108.1(3) . . ?

C38 N5 Zn3 122.3(2) . . ?

C40 N5 Zn3 126.6(2) . . ?

C34 N6 C33 120.8(3) . . ?

C34 N6 Zn4 132.6(2) . . ?

C33 N6 Zn4 106.6(2) . . ?

C54 N7 C56 108.2(3) . . ?

C54 N7 Zn4 120.6(2) . . ?

C56 N7 Zn4 131.1(2) . . ?

C50 N8 C49 120.6(3) . . ?

C50 N8 Zn1 129.2(2) . . ?

C49 N8 Zn1 110.0(2) . . ?

C6 O1 C7 106.0(3) . . ?

C1 O2 Zn1 126.3(2) . . ?

C22 O3 C23 106.1(2) . . ?

C17 O4 Zn2 125.45(19) . . ?

C38 O5 C39 106.8(3) . . ?

C33 O6 Zn3 127.2(2) . . ?

C54 O7 C55 105.2(3) . . ?

C49 O8 Zn4 124.3(2) . . ?

O2 C1 N2 114.9(3) . . ?

O2 C1 C5 125.5(3) . . ?

N2 C1 C5 119.6(3) . . ?

N2 C2 C3 120.6(3) . . ?

N2 C2 C16 118.8(3) . . ?

C3 C2 C16 120.6(3) . . ?

C4 C3 C2 119.8(3) . . ?

C4 C3 H3 120.1 . . ?

C2 C3 H3 120.1 . . ?

C3 C4 C5 121.0(3) . . ?

C3 C4 H4 119.5 . . ?

C5 C4 H4 119.5 . . ?

C4 C5 C1 117.7(3) . . ?

C4 C5 C6 119.7(3) . . ?

C1 C5 C6 122.6(3) . . ?

N1 C6 O1 116.1(3) . . ?

N1 C6 C5 128.6(3) . . ?

O1 C6 C5 115.3(3) . . ?

O1 C7 C8 105.2(3) . . ?

O1 C7 H7A 110.7 . . ?

C8 C7 H7A 110.7 . . ?

O1 C7 H7B 110.7 . . ?

C8 C7 H7B 110.7 . . ?

H7A C7 H7B 108.8 . . ?

N1 C8 C7 102.6(3) . . ?

N1 C8 C9 110.8(3) . . ?

C7 C8 C9 114.3(3) . . ?

N1 C8 H8 109.7 . . ?

C7 C8 H8 109.7 . . ?

C9 C8 H8 109.7 . . ?

C10 C9 C8 110.3(3) . . ?

C10 C9 H9A 109.6 . . ?

C8 C9 H9A 109.6 . . ?

C10 C9 H9B 109.6 . . ?

C8 C9 H9B 109.6 . . ?

H9A C9 H9B 108.1 . . ?

C15 C10 C11 117.4(3) . . ?

C15 C10 C9 121.0(3) . . ?

C11 C10 C9 121.5(3) . . ?

C10 C11 C12 120.4(4) . . ?

C10 C11 H11 119.8 . . ?

C12 C11 H11 119.8 . . ?

C13 C12 C11 121.4(4) . . ?

C13 C12 H12 119.3 . . ?

C11 C12 H12 119.3 . . ?

C12 C13 C14 118.8(4) . . ?

C12 C13 H13 120.6 . . ?

C14 C13 H13 120.6 . . ?

C15 C14 C13 119.5(5) . . ?

C15 C14 H14 120.2 . . ?

C13 C14 H14 120.2 . . ?
C14 C15 C10 122.2(4) . . ?
C14 C15 H15 118.9 . . ?
C10 C15 H15 118.9 . . ?
C2 C16 H16A 109.5 . . ?
C2 C16 H16B 109.5 . . ?
H16A C16 H16B 109.5 . . ?
C2 C16 H16C 109.5 . . ?
H16A C16 H16C 109.5 . . ?
H16B C16 H16C 109.5 . . ?
O4 C17 N4 113.8(3) . . ?
O4 C17 C21 126.5(3) . . ?
N4 C17 C21 119.8(3) . . ?
C19 C18 N4 121.1(3) . . ?
C19 C18 C32 121.2(3) . . ?
N4 C18 C32 117.6(3) . . ?
C18 C19 C20 119.5(3) . . ?
C18 C19 H19 120.3 . . ?
C20 C19 H19 120.3 . . ?
C19 C20 C21 121.2(3) . . ?
C19 C20 H20 119.4 . . ?
C21 C20 H20 119.4 . . ?
C20 C21 C17 117.6(3) . . ?
C20 C21 C22 119.7(3) . . ?
C17 C21 C22 122.6(3) . . ?
N3 C22 O3 116.7(3) . . ?
N3 C22 C21 128.7(3) . . ?
O3 C22 C21 114.5(3) . . ?
O3 C23 C24 103.9(2) . . ?
O3 C23 H23A 111.0 . . ?
C24 C23 H23A 111.0 . . ?
O3 C23 H23B 111.0 . . ?
C24 C23 H23B 111.0 . . ?
H23A C23 H23B 109.0 . . ?
N3 C24 C25 113.0(3) . . ?
N3 C24 C23 102.6(3) . . ?
C25 C24 C23 112.9(3) . . ?
N3 C24 H24 109.4 . . ?
C25 C24 H24 109.4 . . ?
C23 C24 H24 109.4 . . ?
C24 C25 C26 112.0(3) . . ?
C24 C25 H25A 109.2 . . ?
C26 C25 H25A 109.2 . . ?
C24 C25 H25B 109.2 . . ?

C26 C25 H25B 109.2 . . ?
H25A C25 H25B 107.9 . . ?
C27 C26 C31 117.7(3) . . ?
C27 C26 C25 120.3(3) . . ?
C31 C26 C25 122.0(3) . . ?
C26 C27 C28 121.1(5) . . ?
C26 C27 H27 119.4 . . ?
C28 C27 H27 119.4 . . ?
C29 C28 C27 119.9(5) . . ?
C29 C28 H28 120.0 . . ?
C27 C28 H28 120.0 . . ?
C28 C29 C30 121.1(4) . . ?
C28 C29 H29 119.4 . . ?
C30 C29 H29 119.4 . . ?
C29 C30 C31 118.8(5) . . ?
C29 C30 H30 120.6 . . ?
C31 C30 H30 120.6 . . ?
C30 C31 C26 121.1(4) . . ?
C30 C31 H31 119.4 . . ?
C26 C31 H31 119.4 . . ?
C18 C32 H32A 109.5 . . ?
C18 C32 H32B 109.5 . . ?
H32A C32 H32B 109.5 . . ?
C18 C32 H32C 109.5 . . ?
H32A C32 H32C 109.5 . . ?
H32B C32 H32C 109.5 . . ?
O6 C33 N6 114.6(3) . . ?
O6 C33 C37 126.2(3) . . ?
N6 C33 C37 119.3(3) . . ?
N6 C34 C35 121.4(3) . . ?
N6 C34 C48 117.8(3) . . ?
C35 C34 C48 120.7(3) . . ?
C34 C35 C36 119.4(3) . . ?
C34 C35 H35 120.3 . . ?
C36 C35 H35 120.3 . . ?
C37 C36 C35 120.7(3) . . ?
C37 C36 H36 119.7 . . ?
C35 C36 H36 119.7 . . ?
C36 C37 C33 118.3(3) . . ?
C36 C37 C38 120.3(3) . . ?
C33 C37 C38 121.3(3) . . ?
N5 C38 O5 116.5(3) . . ?
N5 C38 C37 128.0(3) . . ?
O5 C38 C37 115.5(3) . . ?

O5 C39 C40 104.7(3) . . ?
O5 C39 H39A 110.8 . . ?
C40 C39 H39A 110.8 . . ?
O5 C39 H39B 110.8 . . ?
C40 C39 H39B 110.8 . . ?
H39A C39 H39B 108.9 . . ?
N5 C40 C41 111.6(3) . . ?
N5 C40 C39 103.2(3) . . ?
C41 C40 C39 114.0(3) . . ?
N5 C40 H40 109.3 . . ?
C41 C40 H40 109.3 . . ?
C39 C40 H40 109.3 . . ?
C40 C41 C42 112.4(3) . . ?
C40 C41 H41A 109.1 . . ?
C42 C41 H41A 109.1 . . ?
C40 C41 H41B 109.1 . . ?
C42 C41 H41B 109.1 . . ?
H41A C41 H41B 107.8 . . ?
C47 C42 C43 119.1(4) . . ?
C47 C42 C41 119.4(4) . . ?
C43 C42 C41 121.5(4) . . ?
C44 C43 C42 120.5(5) . . ?
C44 C43 H43 119.7 . . ?
C42 C43 H43 119.7 . . ?
C45 C44 C43 120.3(5) . . ?
C45 C44 H44 119.9 . . ?
C43 C44 H44 119.9 . . ?
C44 C45 C46 120.9(5) . . ?
C44 C45 H45 119.6 . . ?
C46 C45 H45 119.6 . . ?
C45 C46 C47 119.5(5) . . ?
C45 C46 H46 120.3 . . ?
C47 C46 H46 120.3 . . ?
C42 C47 C46 119.7(5) . . ?
C42 C47 H47 120.1 . . ?
C46 C47 H47 120.1 . . ?
C34 C48 H48A 109.5 . . ?
C34 C48 H48B 109.5 . . ?
H48A C48 H48B 109.5 . . ?
C34 C48 H48C 109.5 . . ?
H48A C48 H48C 109.5 . . ?
H48B C48 H48C 109.5 . . ?
O8 C49 N8 116.0(3) . . ?
O8 C49 C53 125.2(3) . . ?

N8 C49 C53 118.8(3) . . ?
N8 C50 C51 121.5(3) . . ?
N8 C50 C64 118.2(3) . . ?
C51 C50 C64 120.3(3) . . ?
C50 C51 C52 120.1(3) . . ?
C50 C51 H51 119.9 . . ?
C52 C51 H51 119.9 . . ?
C51 C52 C53 119.4(3) . . ?
C51 C52 H52 120.3 . . ?
C53 C52 H52 120.3 . . ?
C52 C53 C49 119.5(3) . . ?
C52 C53 C54 118.1(3) . . ?
C49 C53 C54 122.3(3) . . ?
N7 C54 O7 116.2(3) . . ?
N7 C54 C53 128.1(3) . . ?
O7 C54 C53 115.7(3) . . ?
O7 C55 C56 104.2(3) . . ?
O7 C55 H55A 110.9 . . ?
C56 C55 H55A 110.9 . . ?
O7 C55 H55B 110.9 . . ?
C56 C55 H55B 110.9 . . ?
H55A C55 H55B 108.9 . . ?
N7 C56 C57 113.5(3) . . ?
N7 C56 C55 101.6(3) . . ?
C57 C56 C55 114.5(3) . . ?
N7 C56 H56 109.0 . . ?
C57 C56 H56 109.0 . . ?
C55 C56 H56 109.0 . . ?
C56 C57 C58 108.2(3) . . ?
C56 C57 H57A 110.1 . . ?
C58 C57 H57A 110.1 . . ?
C56 C57 H57B 110.1 . . ?
C58 C57 H57B 110.1 . . ?
H57A C57 H57B 108.4 . . ?
C59 C58 C63 118.8(4) . . ?
C59 C58 C57 120.6(4) . . ?
C63 C58 C57 120.1(3) . . ?
C60 C59 C58 119.9(4) . . ?
C60 C59 H59 120.1 . . ?
C58 C59 H59 120.1 . . ?
C59 C60 C61 120.2(4) . . ?
C59 C60 H60 119.9 . . ?
C61 C60 H60 119.9 . . ?
C62 C61 C60 120.4(4) . . ?

C62 C61 H61 119.8 . . ?
C60 C61 H61 119.8 . . ?
C61 C62 C63 119.7(5) . . ?
C61 C62 H62 120.1 . . ?
C63 C62 H62 120.1 . . ?
C62 C63 C58 121.0(4) . . ?
C62 C63 H63 119.5 . . ?
C58 C63 H63 119.5 . . ?
C50 C64 H64A 109.5 . . ?
C50 C64 H64B 109.5 . . ?
H64A C64 H64B 109.5 . . ?
C50 C64 H64C 109.5 . . ?
H64A C64 H64C 109.5 . . ?
H64B C64 H64C 109.5 . . ?

loop_
 _geom_torsion_atom_site_label_1
 _geom_torsion_atom_site_label_2
 _geom_torsion_atom_site_label_3
 _geom_torsion_atom_site_label_4
 _geom_torsion
 _geom_torsion_site_symmetry_1
 _geom_torsion_site_symmetry_2
 _geom_torsion_site_symmetry_3
 _geom_torsion_site_symmetry_4
 _geom_torsion_publ_flag
O2 Zn1 N1 C6 11.1(3) . . . . ?
N8 Zn1 N1 C6 -108.8(3) . . . . ?
Cl1 Zn1 N1 C6 120.4(2) . . . . ?
O2 Zn1 N1 C8 -175.8(2) . . . . ?
N8 Zn1 N1 C8 64.4(3) . . . . ?
Cl1 Zn1 N1 C8 -66.5(3) . . . . ?
O4 Zn2 N2 C2 -90.3(3) . . . . ?
N3 Zn2 N2 C2 165.7(3) . . . . ?
Cl2 Zn2 N2 C2 30.4(3) . . . . ?
O4 Zn2 N2 C1 84.17(19) . . . . ?
N3 Zn2 N2 C1 -19.9(2) . . . . ?
Cl2 Zn2 N2 C1 -155.11(16) . . . . ?
O4 Zn2 N3 C22 -8.6(2) . . . . ?
N2 Zn2 N3 C22 99.6(3) . . . . ?
Cl2 Zn2 N3 C22 -122.7(2) . . . . ?
O4 Zn2 N3 C24 -169.5(2) . . . . ?
N2 Zn2 N3 C24 -61.3(3) . . . . ?
Cl2 Zn2 N3 C24 76.4(2) . . . . ?

O6 Zn3 N4 C18 97.1(3) . . . . ?
N5 Zn3 N4 C18 -155.1(3) . . . . ?
Cl3 Zn3 N4 C18 -25.5(3) . . . . ?
O6 Zn3 N4 C17 -76.00(19) . . . . ?
N5 Zn3 N4 C17 31.8(3) . . . . ?
Cl3 Zn3 N4 C17 161.39(16) . . . . ?
O6 Zn3 N5 C38 -19.9(3) . . . . ?
N4 Zn3 N5 C38 -132.0(2) . . . . ?
Cl3 Zn3 N5 C38 93.6(3) . . . . ?
O6 Zn3 N5 C40 -177.9(3) . . . . ?
N4 Zn3 N5 C40 70.0(3) . . . . ?
Cl3 Zn3 N5 C40 -64.4(2) . . . . ?
O8 Zn4 N6 C34 -118.5(3) . . . . ?
N7 Zn4 N6 C34 128.1(3) . . . . ?
Cl4 Zn4 N6 C34 6.3(3) . . . . ?
O8 Zn4 N6 C33 62.0(2) . . . . ?
N7 Zn4 N6 C33 -51.4(2) . . . . ?
Cl4 Zn4 N6 C33 -173.16(17) . . . . ?
O8 Zn4 N7 C54 24.2(3) . . . . ?
N6 Zn4 N7 C54 151.1(2) . . . . ?
Cl4 Zn4 N7 C54 -82.1(3) . . . . ?
O8 Zn4 N7 C56 -158.8(3) . . . . ?
N6 Zn4 N7 C56 -31.9(3) . . . . ?
Cl4 Zn4 N7 C56 94.9(3) . . . . ?
O2 Zn1 N8 C50 127.7(3) . . . . ?
N1 Zn1 N8 C50 -125.0(3) . . . . ?
Cl1 Zn1 N8 C50 0.4(3) . . . . ?
O2 Zn1 N8 C49 -57.2(2) . . . . ?
N1 Zn1 N8 C49 50.1(2) . . . . ?
Cl1 Zn1 N8 C49 175.52(17) . . . . ?
N1 Zn1 O2 C1 -11.6(3) . . . . ?
N8 Zn1 O2 C1 108.3(2) . . . . ?
Cl1 Zn1 O2 C1 -119.8(2) . . . . ?
N3 Zn2 O4 C17 9.8(2) . . . . ?
N2 Zn2 O4 C17 -118.1(2) . . . . ?
Cl2 Zn2 O4 C17 119.7(2) . . . . ?
N5 Zn3 O6 C33 15.2(3) . . . . ?
N4 Zn3 O6 C33 155.8(3) . . . . ?
Cl3 Zn3 O6 C33 -81.9(3) . . . . ?
N7 Zn4 O8 C49 -26.9(2) . . . . ?
N6 Zn4 O8 C49 -154.8(2) . . . . ?
Cl4 Zn4 O8 C49 76.7(2) . . . . ?
Zn1 O2 C1 N2 -172.59(18) . . . . ?
Zn1 O2 C1 C5 8.2(4) . . . . ?

C2 N2 C1 O2 -178.3(3) . . . . ?
Zn2 N2 C1 O2 6.6(3) . . . . ?
C2 N2 C1 C5 0.9(4) . . . . ?
Zn2 N2 C1 C5 -174.2(2) . . . . ?
C1 N2 C2 C3 -0.4(5) . . . . ?
Zn2 N2 C2 C3 173.4(2) . . . . ?
C1 N2 C2 C16 -180.0(3) . . . . ?
Zn2 N2 C2 C16 -6.1(4) . . . . ?
N2 C2 C3 C4 -0.3(5) . . . . ?
C16 C2 C3 C4 179.2(3) . . . . ?
C2 C3 C4 C5 0.5(5) . . . . ?
C3 C4 C5 C1 -0.1(5) . . . . ?
C3 C4 C5 C6 178.9(3) . . . . ?
O2 C1 C5 C4 178.5(3) . . . . ?
N2 C1 C5 C4 -0.6(4) . . . . ?
O2 C1 C5 C6 -0.5(5) . . . . ?
N2 C1 C5 C6 -179.6(3) . . . . ?
C8 N1 C6 O1 -0.2(4) . . . . ?
Zn1 N1 C6 O1 174.1(2) . . . . ?
C8 N1 C6 C5 177.7(3) . . . . ?
Zn1 N1 C6 C5 -7.9(5) . . . . ?
C7 O1 C6 N1 -5.7(4) . . . . ?
C7 O1 C6 C5 176.1(3) . . . . ?
C4 C5 C6 N1 -178.3(3) . . . . ?
C1 C5 C6 N1 0.6(5) . . . . ?
C4 C5 C6 O1 -0.3(4) . . . . ?
C1 C5 C6 O1 178.6(3) . . . . ?
C6 O1 C7 C8 8.7(4) . . . . ?
C6 N1 C8 C7 5.7(3) . . . . ?
Zn1 N1 C8 C7 -168.2(2) . . . . ?
C6 N1 C8 C9 128.0(3) . . . . ?
Zn1 N1 C8 C9 -45.8(4) . . . . ?
O1 C7 C8 N1 -8.6(3) . . . . ?
O1 C7 C8 C9 -128.5(3) . . . . ?
N1 C8 C9 C10 -173.7(3) . . . . ?
C7 C8 C9 C10 -58.5(4) . . . . ?
C8 C9 C10 C15 -77.8(5) . . . . ?
C8 C9 C10 C11 100.1(4) . . . . ?
C15 C10 C11 C12 2.6(6) . . . . ?
C9 C10 C11 C12 -175.3(4) . . . . ?
C10 C11 C12 C13 0.1(7) . . . . ?
C11 C12 C13 C14 -3.4(8) . . . . ?
C12 C13 C14 C15 3.9(9) . . . . ?
C13 C14 C15 C10 -1.1(10) . . . . ?

C11 C10 C15 C14 -2.1(8) . . . . ?
C9 C10 C15 C14 175.9(5) . . . . ?
Zn2 O4 C17 N4 170.39(18) . . . . ?
Zn2 O4 C17 C21 -9.7(4) . . . . ?
C18 N4 C17 O4 177.7(3) . . . . ?
Zn3 N4 C17 O4 -8.3(3) . . . . ?
C18 N4 C17 C21 -2.2(4) . . . . ?
Zn3 N4 C17 C21 171.8(2) . . . . ?
C17 N4 C18 C19 -0.5(5) . . . . ?
Zn3 N4 C18 C19 -172.9(2) . . . . ?
C17 N4 C18 C32 -179.8(3) . . . . ?
Zn3 N4 C18 C32 7.8(4) . . . . ?
N4 C18 C19 C20 2.8(5) . . . . ?
C32 C18 C19 C20 -177.9(3) . . . . ?
C18 C19 C20 C21 -2.3(5) . . . . ?
C19 C20 C21 C17 -0.3(5) . . . . ?
C19 C20 C21 C22 177.1(3) . . . . ?
O4 C17 C21 C20 -177.4(3) . . . . ?
N4 C17 C21 C20 2.5(4) . . . . ?
O4 C17 C21 C22 5.4(5) . . . . ?
N4 C17 C21 C22 -174.7(3) . . . . ?
C24 N3 C22 O3 -4.6(4) . . . . ?
Zn2 N3 C22 O3 -168.8(2) . . . . ?
C24 N3 C22 C21 172.1(3) . . . . ?
Zn2 N3 C22 C21 7.8(4) . . . . ?
C23 O3 C22 N3 -6.3(4) . . . . ?
C23 O3 C22 C21 176.5(3) . . . . ?
C20 C21 C22 N3 178.1(3) . . . . ?
C17 C21 C22 N3 -4.7(5) . . . . ?
C20 C21 C22 O3 -5.2(4) . . . . ?
C17 C21 C22 O3 172.0(3) . . . . ?
C22 O3 C23 C24 13.6(3) . . . . ?
C22 N3 C24 C25 134.6(3) . . . . ?
Zn2 N3 C24 C25 -62.4(3) . . . . ?
C22 N3 C24 C23 12.7(3) . . . . ?
Zn2 N3 C24 C23 175.6(2) . . . . ?
O3 C23 C24 N3 -15.7(3) . . . . ?
O3 C23 C24 C25 -137.6(3) . . . . ?
N3 C24 C25 C26 -170.8(3) . . . . ?
C23 C24 C25 C26 -54.9(4) . . . . ?
C24 C25 C26 C27 131.7(4) . . . . ?
C24 C25 C26 C31 -47.4(5) . . . . ?
C31 C26 C27 C28 -4.8(7) . . . . ?
C25 C26 C27 C28 176.1(5) . . . . ?

C26 C27 C28 C29 3.3(9) . . . . ?
C27 C28 C29 C30 0.8(9) . . . . ?
C28 C29 C30 C31 -3.1(7) . . . . ?
C29 C30 C31 C26 1.6(6) . . . . ?
C27 C26 C31 C30 2.3(6) . . . . ?
C25 C26 C31 C30 -178.5(3) . . . . ?
Zn3 O6 C33 N6 173.38(19) . . . . ?
Zn3 O6 C33 C37 -5.7(5) . . . . ?
C34 N6 C33 O6 -176.8(3) . . . . ?
Zn4 N6 C33 O6 2.8(3) . . . . ?
C34 N6 C33 C37 2.4(4) . . . . ?
Zn4 N6 C33 C37 -178.1(2) . . . . ?
C33 N6 C34 C35 0.3(5) . . . . ?
Zn4 N6 C34 C35 -179.1(3) . . . . ?
C33 N6 C34 C48 -178.6(3) . . . . ?
Zn4 N6 C34 C48 1.9(5) . . . . ?
N6 C34 C35 C36 -1.7(5) . . . . ?
C48 C34 C35 C36 177.3(4) . . . . ?
C34 C35 C36 C37 0.2(5) . . . . ?
C35 C36 C37 C33 2.4(5) . . . . ?
C35 C36 C37 C38 -177.0(3) . . . . ?
O6 C33 C37 C36 175.4(3) . . . . ?
N6 C33 C37 C36 -3.7(4) . . . . ?
O6 C33 C37 C38 -5.3(5) . . . . ?
N6 C33 C37 C38 175.7(3) . . . . ?
C40 N5 C38 O5 -3.1(4) . . . . ?
Zn3 N5 C38 O5 -164.7(2) . . . . ?
C40 N5 C38 C37 178.5(3) . . . . ?
Zn3 N5 C38 C37 16.9(5) . . . . ?
C39 O5 C38 N5 -2.7(4) . . . . ?
C39 O5 C38 C37 175.9(3) . . . . ?
C36 C37 C38 N5 177.9(3) . . . . ?
C33 C37 C38 N5 -1.5(5) . . . . ?
C36 C37 C38 O5 -0.6(4) . . . . ?
C33 C37 C38 O5 -179.9(3) . . . . ?
C38 O5 C39 C40 6.9(4) . . . . ?
C38 N5 C40 C41 130.0(3) . . . . ?
Zn3 N5 C40 C41 -69.5(4) . . . . ?
C38 N5 C40 C39 7.1(4) . . . . ?
Zn3 N5 C40 C39 167.6(2) . . . . ?
O5 C39 C40 N5 -8.3(4) . . . . ?
O5 C39 C40 C41 -129.6(3) . . . . ?
N5 C40 C41 C42 -174.9(3) . . . . ?
C39 C40 C41 C42 -58.4(4) . . . . ?

C40 C41 C42 C47 137.7(4) . . . . ?
C40 C41 C42 C43 -41.9(5) . . . . ?
C47 C42 C43 C44 -0.3(7) . . . . ?
C41 C42 C43 C44 179.3(4) . . . . ?
C42 C43 C44 C45 0.2(7) . . . . ?
C43 C44 C45 C46 -0.2(8) . . . . ?
C44 C45 C46 C47 0.1(7) . . . . ?
C43 C42 C47 C46 0.2(6) . . . . ?
C41 C42 C47 C46 -179.4(4) . . . . ?
C45 C46 C47 C42 -0.1(6) . . . . ?
Zn4 O8 C49 N8 -164.82(19) . . . . ?
Zn4 O8 C49 C53 15.8(4) . . . . ?
C50 N8 C49 O8 178.7(3) . . . . ?
Zn1 N8 C49 O8 3.1(3) . . . . ?
C50 N8 C49 C53 -1.9(4) . . . . ?
Zn1 N8 C49 C53 -177.5(2) . . . . ?
C49 N8 C50 C51 -0.9(5) . . . . ?
Zn1 N8 C50 C51 173.8(2) . . . . ?
C49 N8 C50 C64 179.3(3) . . . . ?
Zn1 N8 C50 C64 -6.0(4) . . . . ?
N8 C50 C51 C52 2.3(5) . . . . ?
C64 C50 C51 C52 -177.9(3) . . . . ?
C50 C51 C52 C53 -0.9(5) . . . . ?
C51 C52 C53 C49 -1.9(5) . . . . ?
C51 C52 C53 C54 174.4(3) . . . . ?
O8 C49 C53 C52 -177.4(3) . . . . ?
N8 C49 C53 C52 3.3(5) . . . . ?
O8 C49 C53 C54 6.5(5) . . . . ?
N8 C49 C53 C54 -172.8(3) . . . . ?
C56 N7 C54 O7 -5.9(4) . . . . ?
Zn4 N7 C54 O7 171.7(2) . . . . ?
C56 N7 C54 C53 170.3(3) . . . . ?
Zn4 N7 C54 C53 -12.1(5) . . . . ?
C55 O7 C54 N7 -8.7(4) . . . . ?
C55 O7 C54 C53 174.6(3) . . . . ?
C52 C53 C54 N7 175.6(3) . . . . ?
C49 C53 C54 N7 -8.2(5) . . . . ?
C52 C53 C54 O7 -8.2(5) . . . . ?
C49 C53 C54 O7 168.0(3) . . . . ?
C54 O7 C55 C56 18.5(4) . . . . ?
C54 N7 C56 C57 140.2(3) . . . . ?
Zn4 N7 C56 C57 -37.0(4) . . . . ?
C54 N7 C56 C55 16.8(4) . . . . ?
Zn4 N7 C56 C55 -160.5(2) . . . . ?

O7 C55 C56 N7 -21.1(4) . . . . ?
O7 C55 C56 C57 -143.8(3) . . . . ?
N7 C56 C57 C58 178.1(3) . . . . ?
C55 C56 C57 C58 -65.8(4) . . . . ?
C56 C57 C58 C59 102.2(4) . . . . ?
C56 C57 C58 C63 -69.7(5) . . . . ?
C63 C58 C59 C60 -0.4(6) . . . . ?
C57 C58 C59 C60 -172.5(4) . . . . ?
C58 C59 C60 C61 0.8(7) . . . . ?
C59 C60 C61 C62 -1.0(8) . . . . ?
C60 C61 C62 C63 1.0(7) . . . . ?
C61 C62 C63 C58 -0.7(7) . . . . ?
C59 C58 C63 C62 0.4(6) . . . . ?
C57 C58 C63 C62 172.5(4) . . . . ?

_diffrn_measured_fraction_theta_max   0.999
_diffrn_reflns_theta_full   28.71
_diffrn_measured_fraction_theta_full   0.999
_refine_diff_density_max   0.358
_refine_diff_density_min   -0.289
_refine_diff_density_rms   0.060
