# Supplementary material for: One-step multicomponent synthesis of chiral oxazolinyl-zinc complexes
Source: Chem Cent J. 2017 Aug 9;11:81. doi: 10.1186/s13065-017-0305-1 (PMC5549684; doi:10.1186/s13065-017-0305-1)
Supplement: Supplementary file 2 — Additional file 2. Copies of NMR spectra. [file 13065_2017_305_MOESM2_ESM.pdf]

Supporting Information

One-step multicomponent synthesis of chiral oxazolinyl-zinc complexes

Mei Luo,[a*] Jing Cheng Zhang,[a] Wen Min Pang, [b] King Kuok (Mimi) Hii[c*]

[a] College of Chemistry and Chemical Engineering, Hefei University of Technology, 230009, People's Republic of China
[b] Department of Chemistry, University of Science and Technoogy of China, 230009, People's Republic of China

[c] Department of Chemistry, Imperial College London, Exhibition Road, South Kensington, London SW7 2AZ, U.K.

luomei@pku.edu.cn, luomeihuahua@sohu.com
mimi.hii@imperial.ac.uk

Contents of the supporting information:

Copies of NMR S7

S7

Compound 1

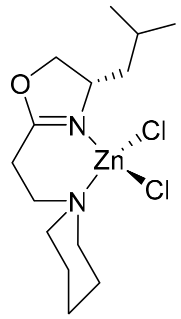

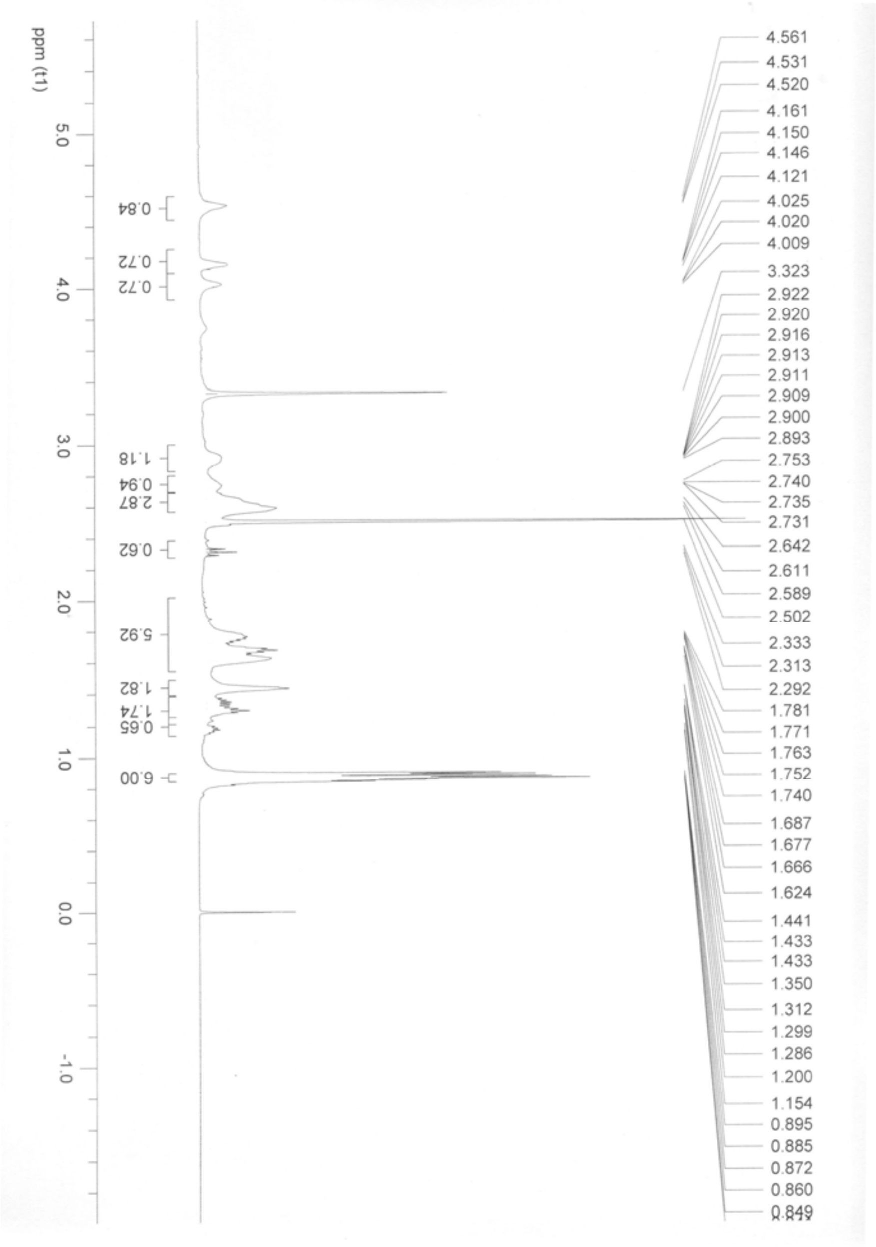

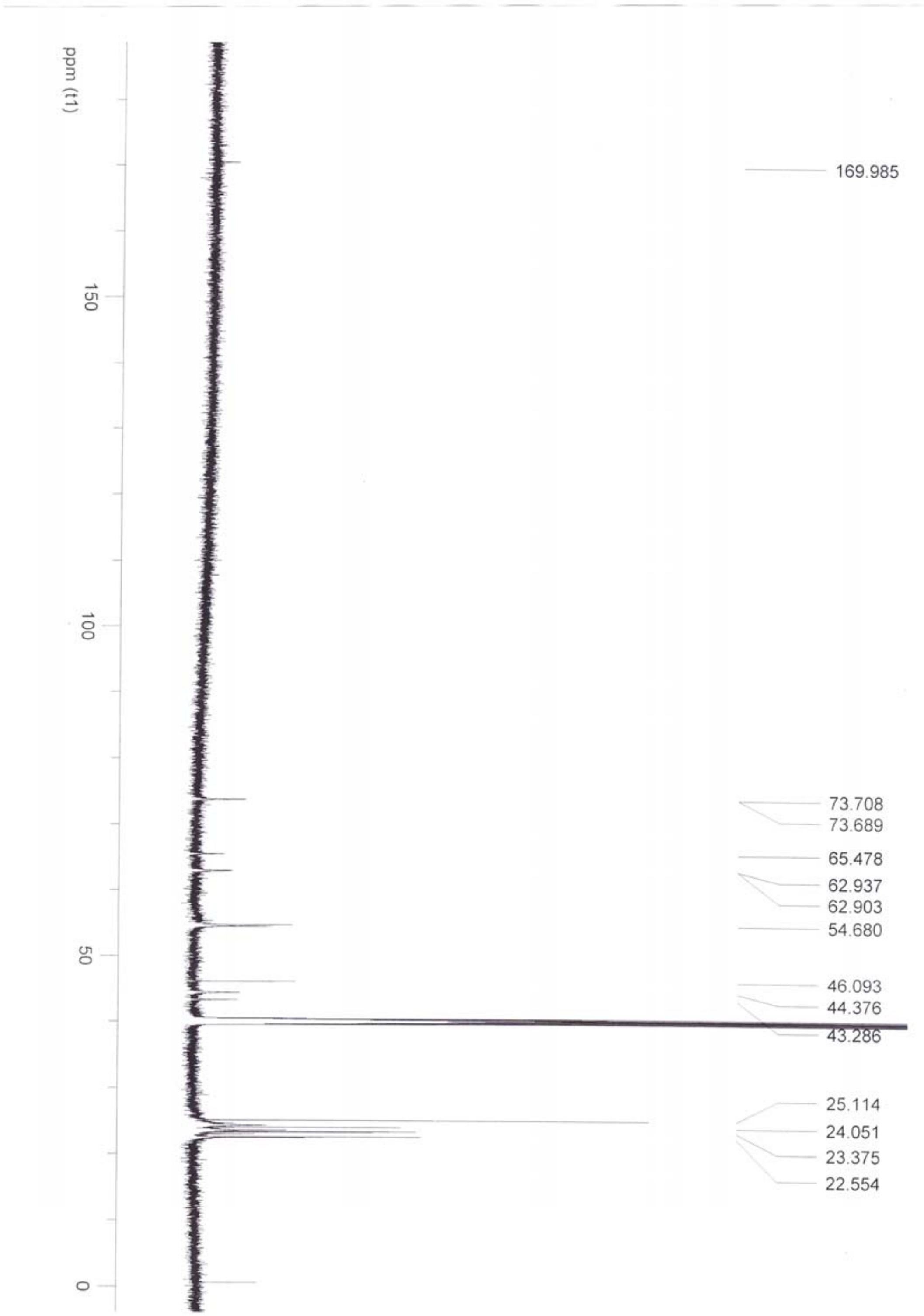
ppm (t1)
150
100
50
0
169.985
73.708
73.689
65.478
62.937
62.903
54.680
46.093
44.376
43.286
25.114
24.051
23.375
22.554

Compound 2

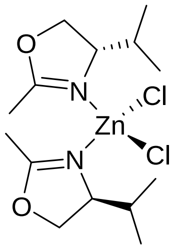
O
N
Zn
Cl
Cl
N
O

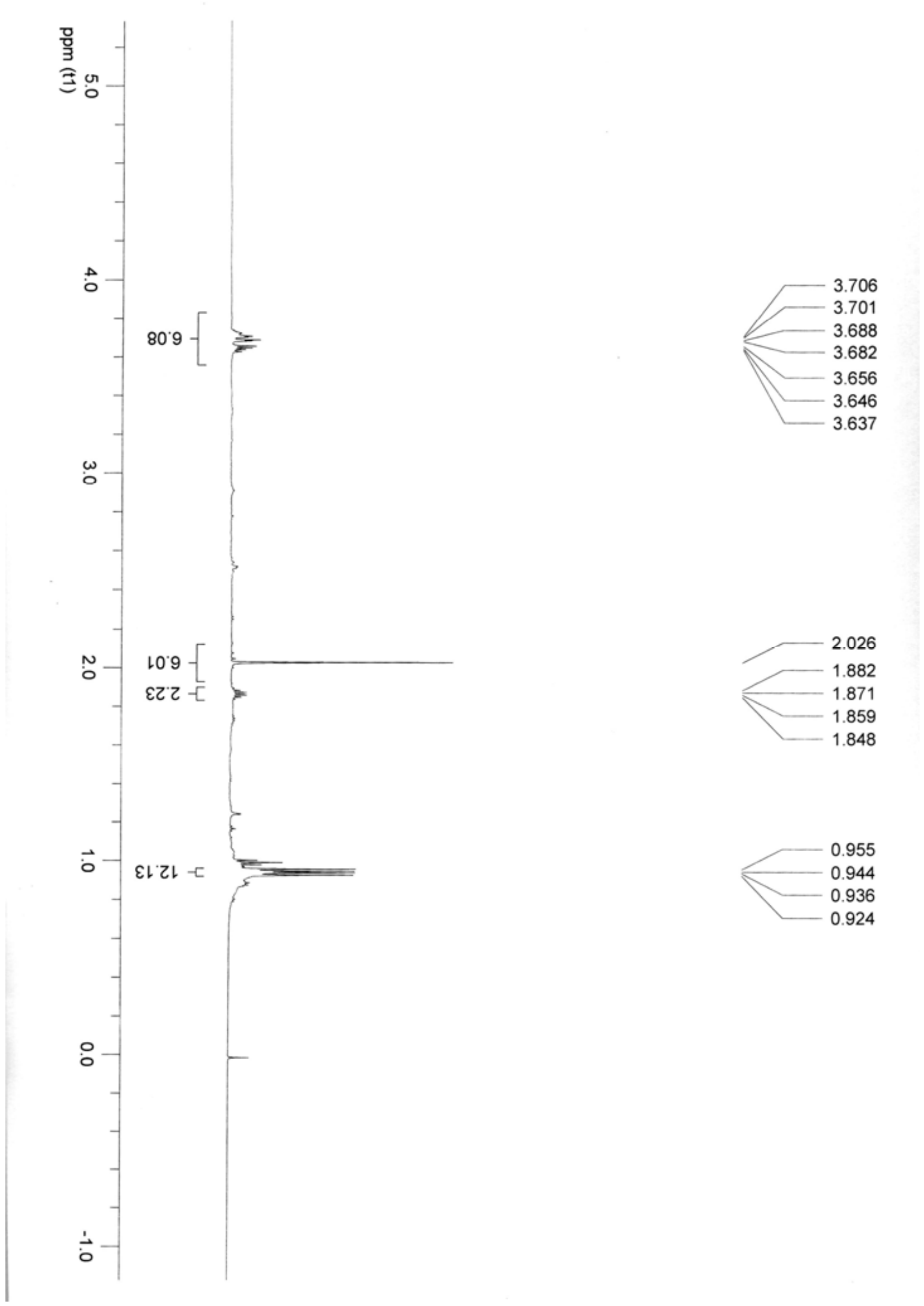
ppm (t1)
5.0
4.0
3.0
2.0
1.0
0.0
-1.0
6.08
6.01
2.23
12.13
3.706
3.701
3.688
3.682
3.656
3.646
3.637
2.026
1.882
1.871
1.859
1.848
0.955
0.944
0.936
0.924

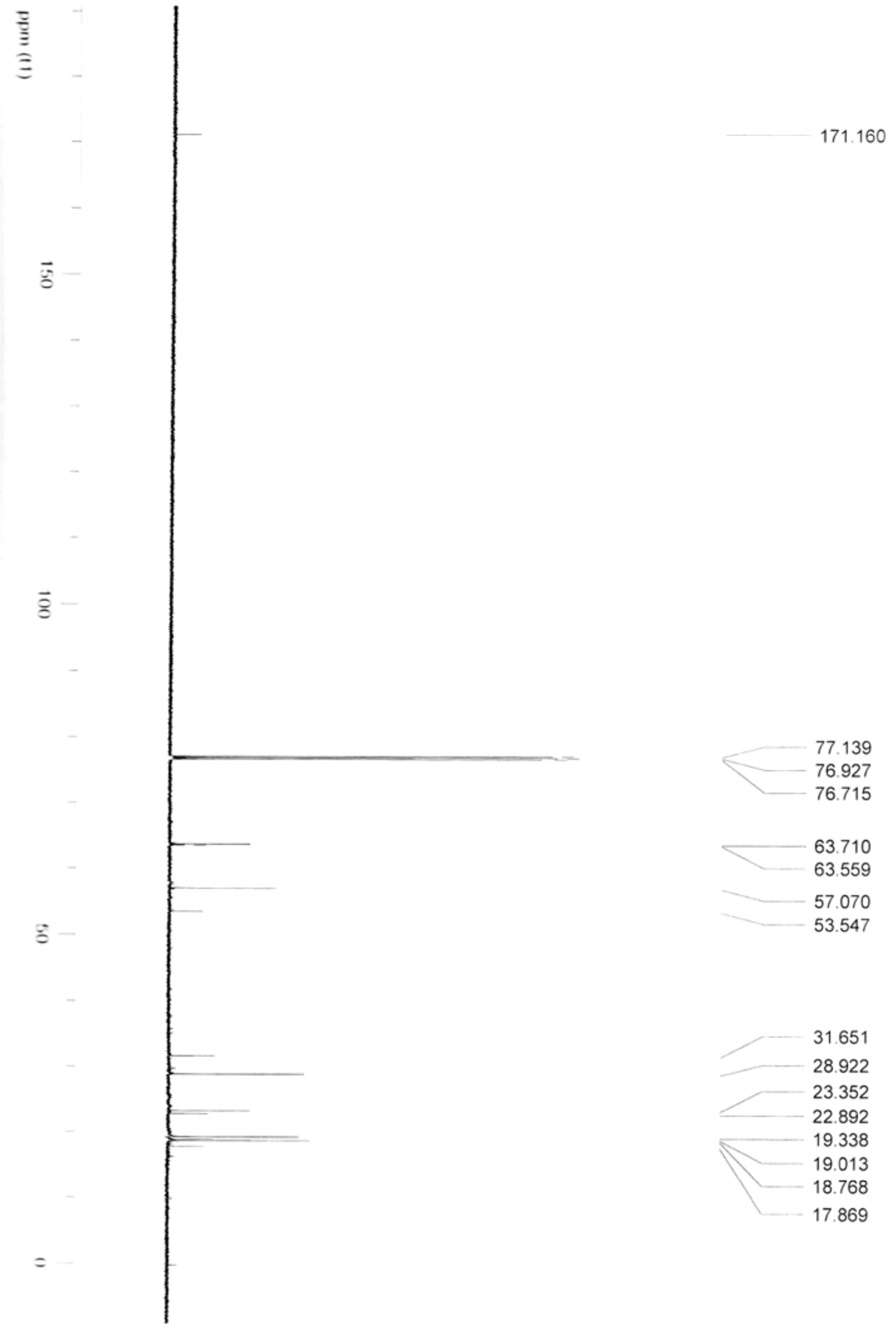
171.160
77.139
76.927
76.715
63.710
63.559
57.070
53.547
31.651
28.922
23.352
22.892
19.338
19.013
18.768
17.869
ppm (f1)
150
100
50
0

Compound 3

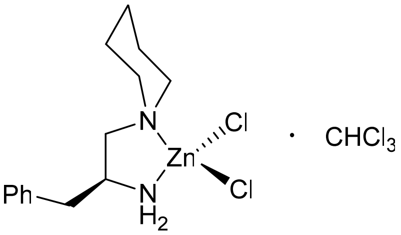
N
Cl
Zn
Cl
Ph
N
H2
· CHCl3

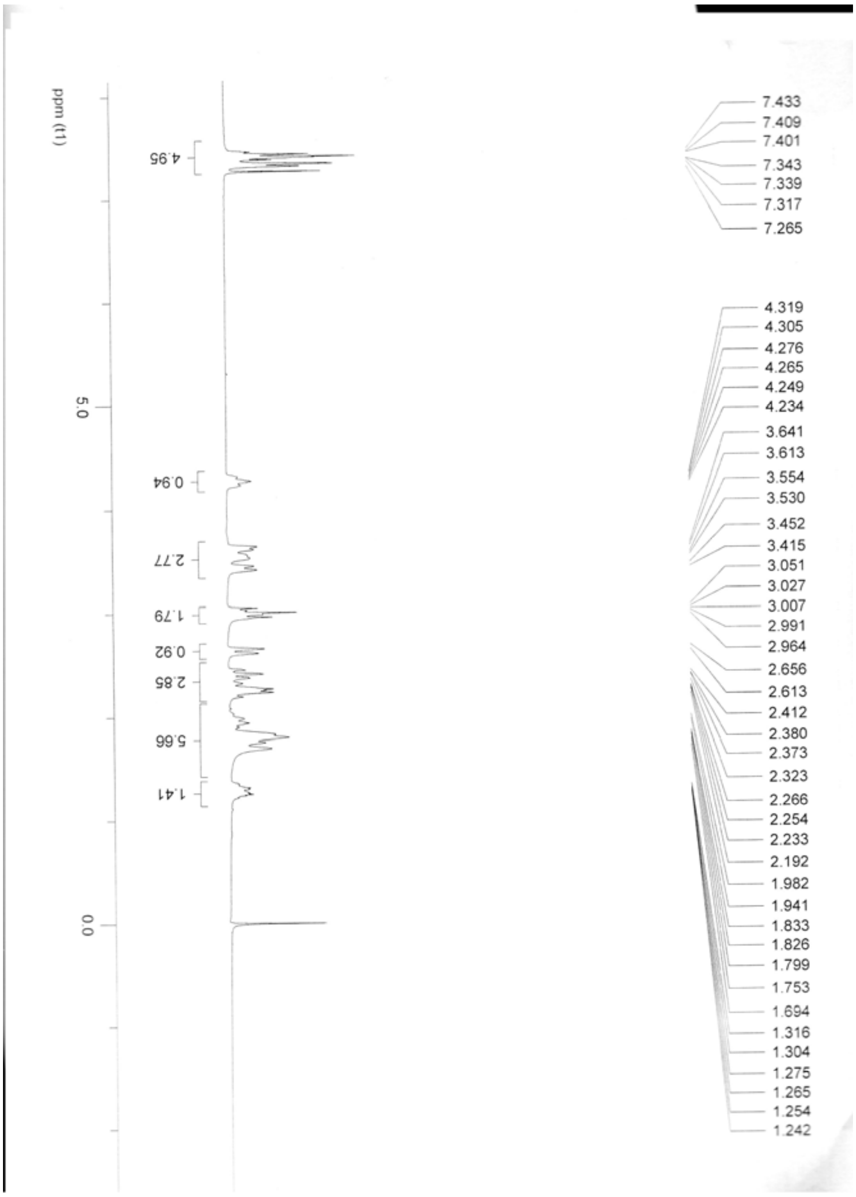
ppm (t1)
5.0
0.0
4.95
0.94
2.77
1.79
0.92
2.85
5.66
1.41
7.433
7.409
7.401
7.343
7.339
7.317
7.265
4.319
4.305
4.276
4.265
4.249
4.234
3.641
3.613
3.554
3.530
3.452
3.415
3.051
3.027
3.007
2.991
2.964
2.656
2.613
2.412
2.380
2.373
2.323
2.266
2.254
2.233
2.192
1.982
1.941
1.833
1.826
1.799
1.753
1.694
1.316
1.304
1.275
1.265
1.254
1.242

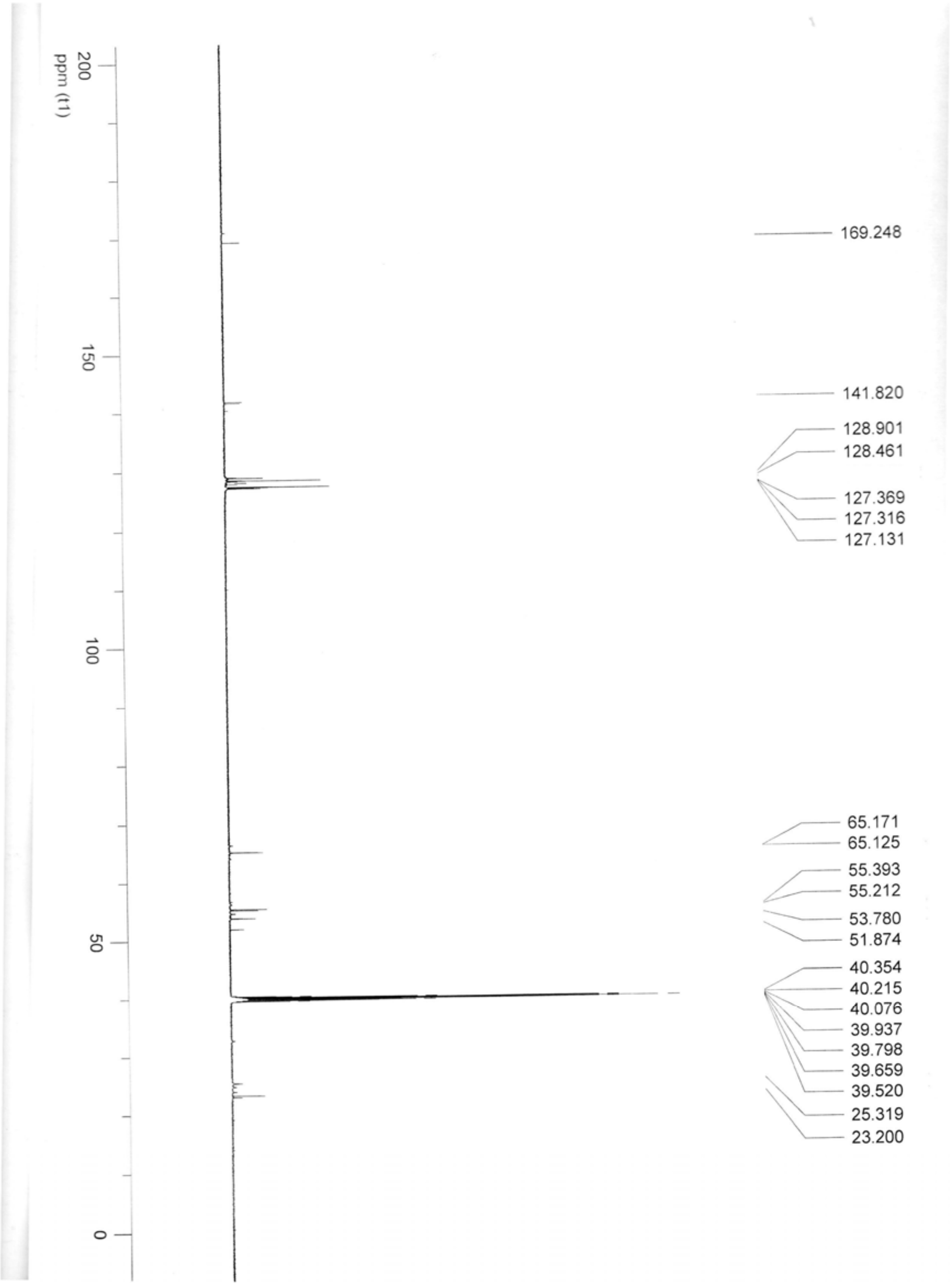
200
ppm (t1)
150
100
50
0
169.248
141.820
128.901
128.461
127.369
127.316
127.131
65.171
65.125
55.393
55.212
53.780
51.874
40.354
40.215
40.076
39.937
39.798
39.659
39.520
25.319
23.200

Compound 4

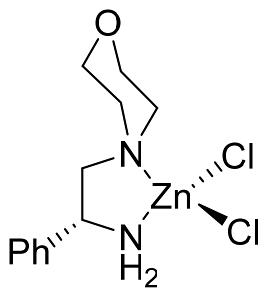

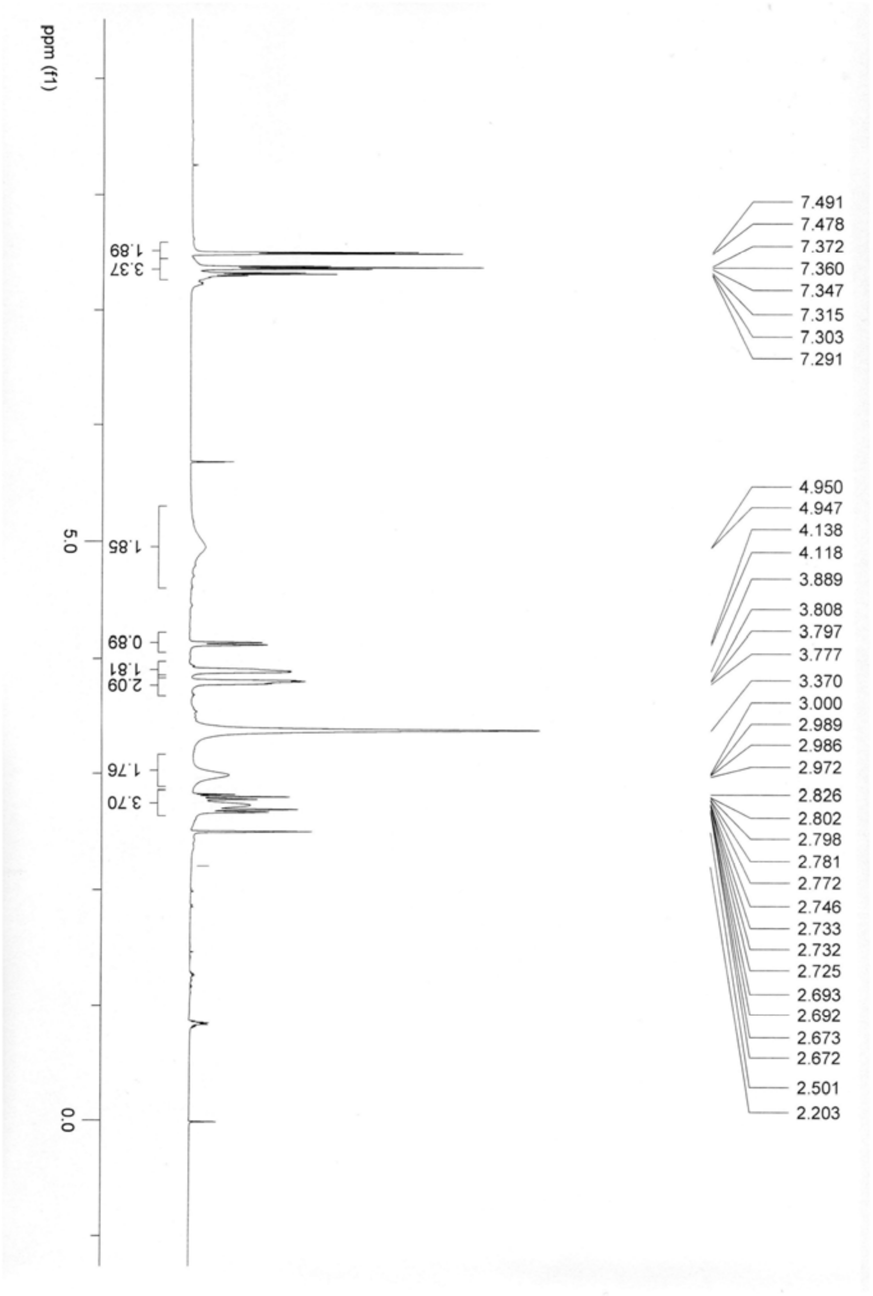
7.491
7.478
7.372
7.360
7.347
7.315
7.303
7.291
4.950
4.947
4.138
4.118
3.889
3.808
3.797
3.777
3.370
3.000
2.989
2.986
2.972
2.826
2.802
2.798
2.781
2.772
2.746
2.733
2.732
2.725
2.693
2.692
2.673
2.672
2.501
2.203
1.89
3.37
1.85
0.89
1.81
2.09
1.76
3.70
5.0
0.0
ppm (f1)

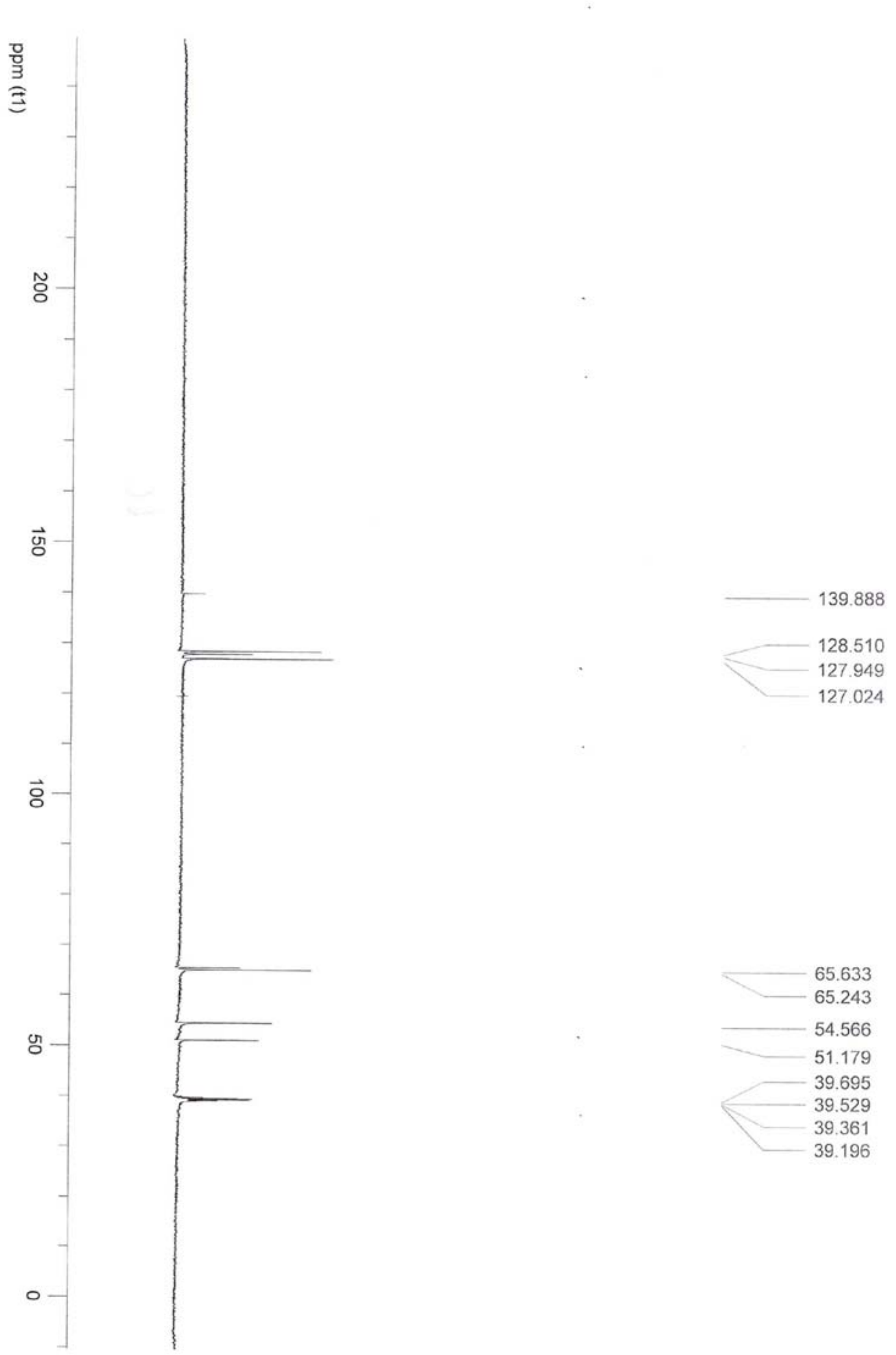
ppm (t1)
200
150
100
50
0
139.888
128.510
127.949
127.024
65.633
65.243
54.566
51.179
39.695
39.529
39.361
39.196

Compound 5

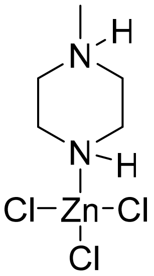

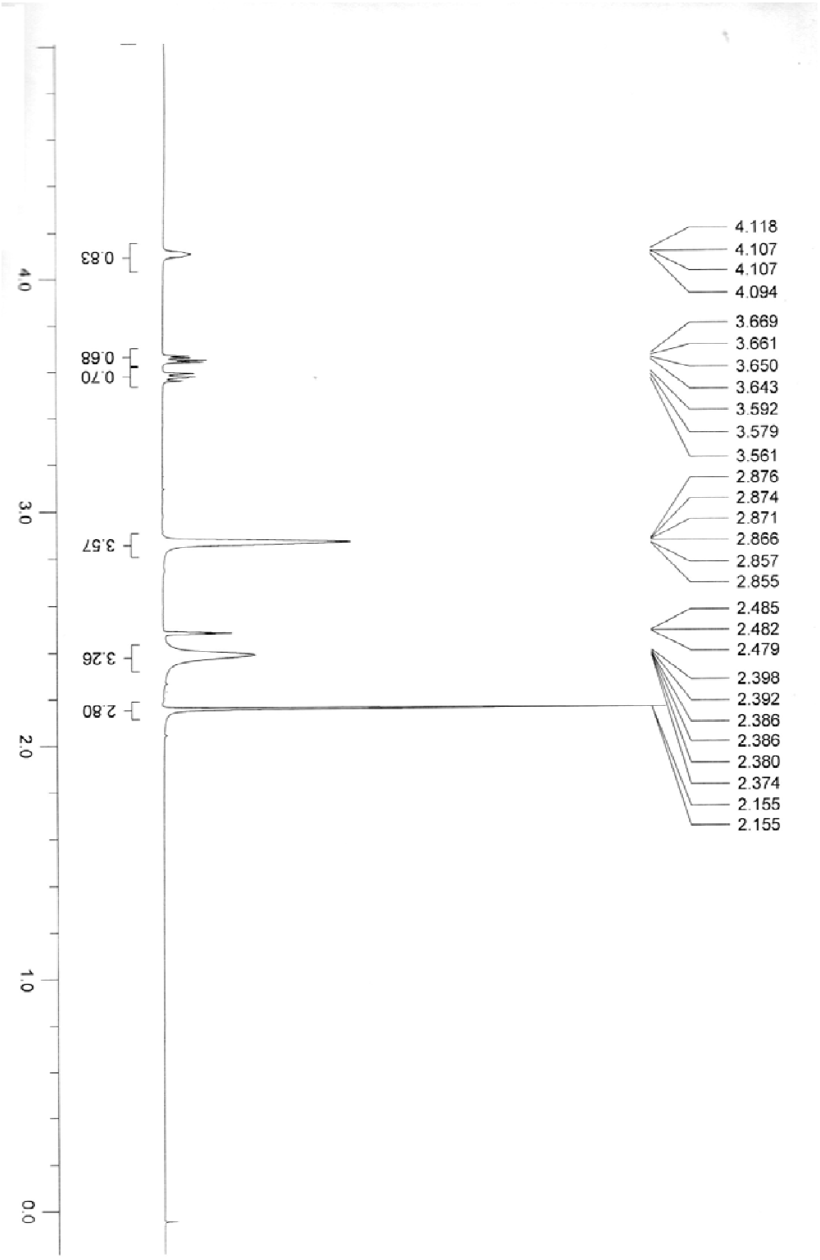
4.118
4.107
4.107
4.094
3.669
3.661
3.650
3.643
3.592
3.579
3.561
2.876
2.874
2.871
2.866
2.857
2.855
2.485
2.482
2.479
2.398
2.392
2.386
2.386
2.380
2.374
2.155
2.155

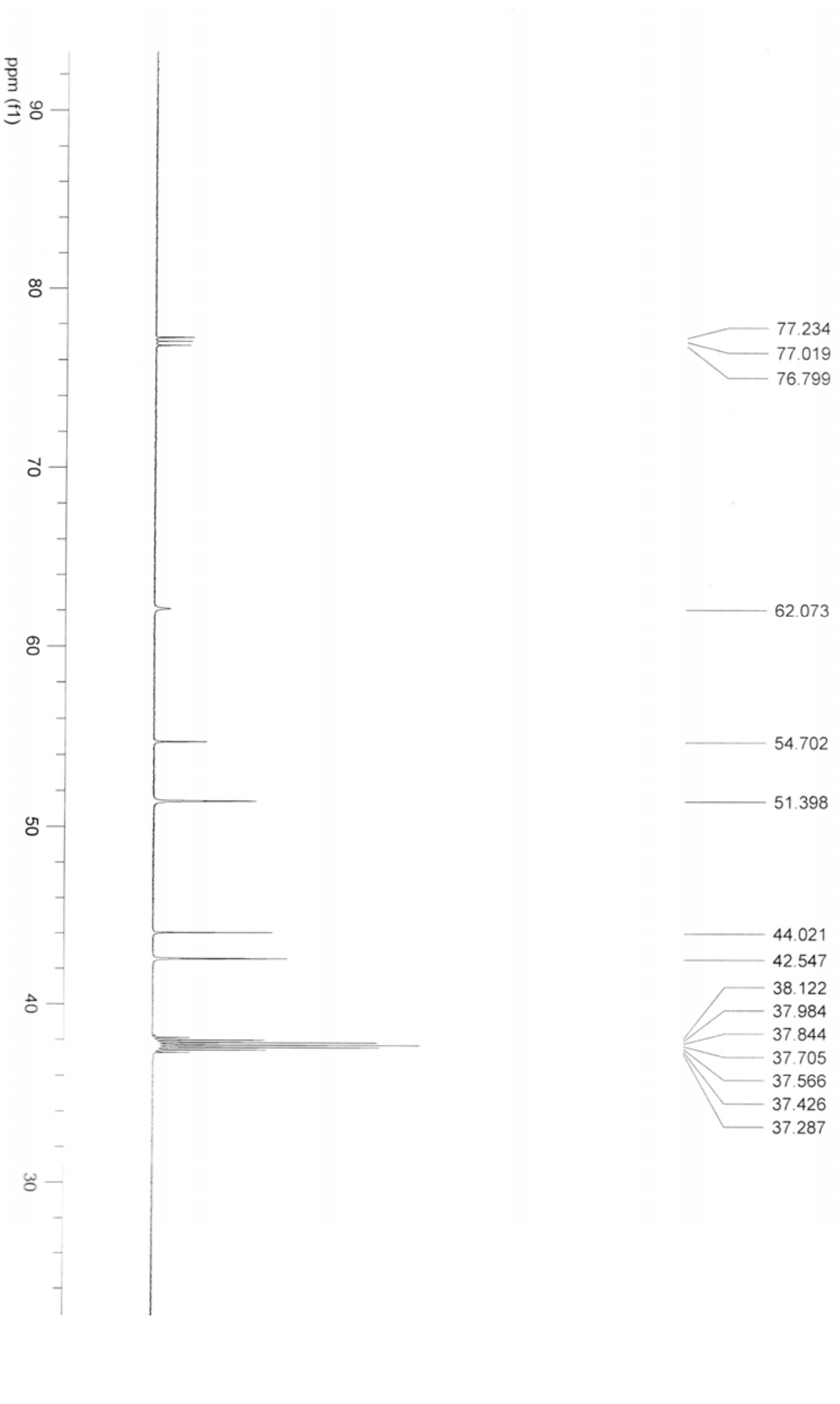
ppm (f1)
90
80
70
60
50
40
30
77.234
77.019
76.799
62.073
54.702
51.398
44.021
42.547
38.122
37.984
37.844
37.705
37.566
37.426
37.287

Compound 6

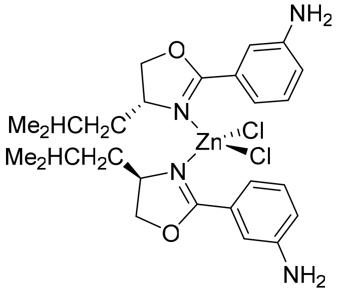
NH2
O
N
Me2HCH2C
Zn
Cl
Cl
Me2HCH2C
N
O
NH2

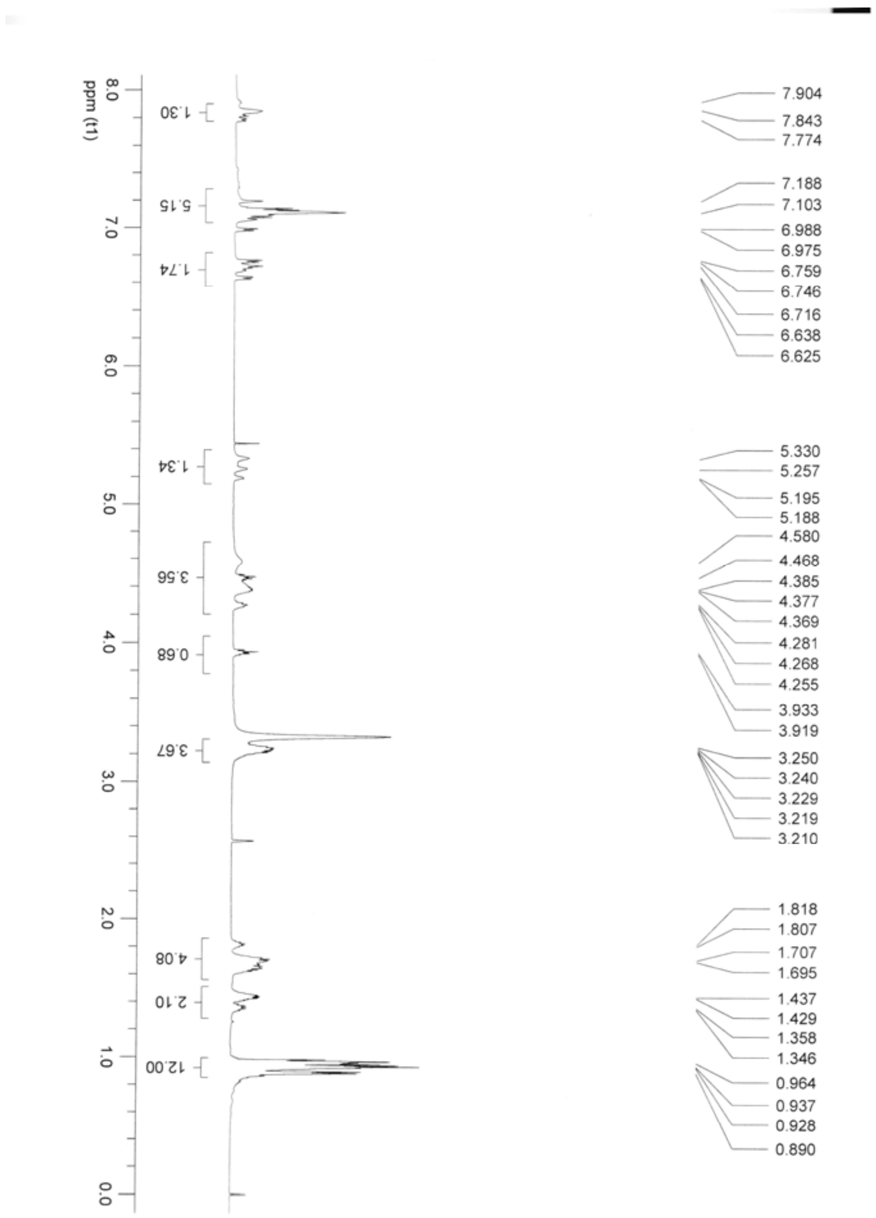
ppm (t1)
8.0
7.0
6.0
5.0
4.0
3.0
2.0
1.0
0.0
1.30
5.15
1.74
1.34
3.56
0.68
3.67
4.08
2.10
12.00
7.904
7.843
7.774
7.188
7.103
6.988
6.975
6.759
6.746
6.716
6.638
6.625
5.330
5.257
5.195
5.188
4.580
4.468
4.385
4.377
4.369
4.281
4.268
4.255
3.933
3.919
3.250
3.240
3.229
3.219
3.210
1.818
1.807
1.707
1.695
1.437
1.429
1.358
1.346
0.964
0.937
0.928
0.890

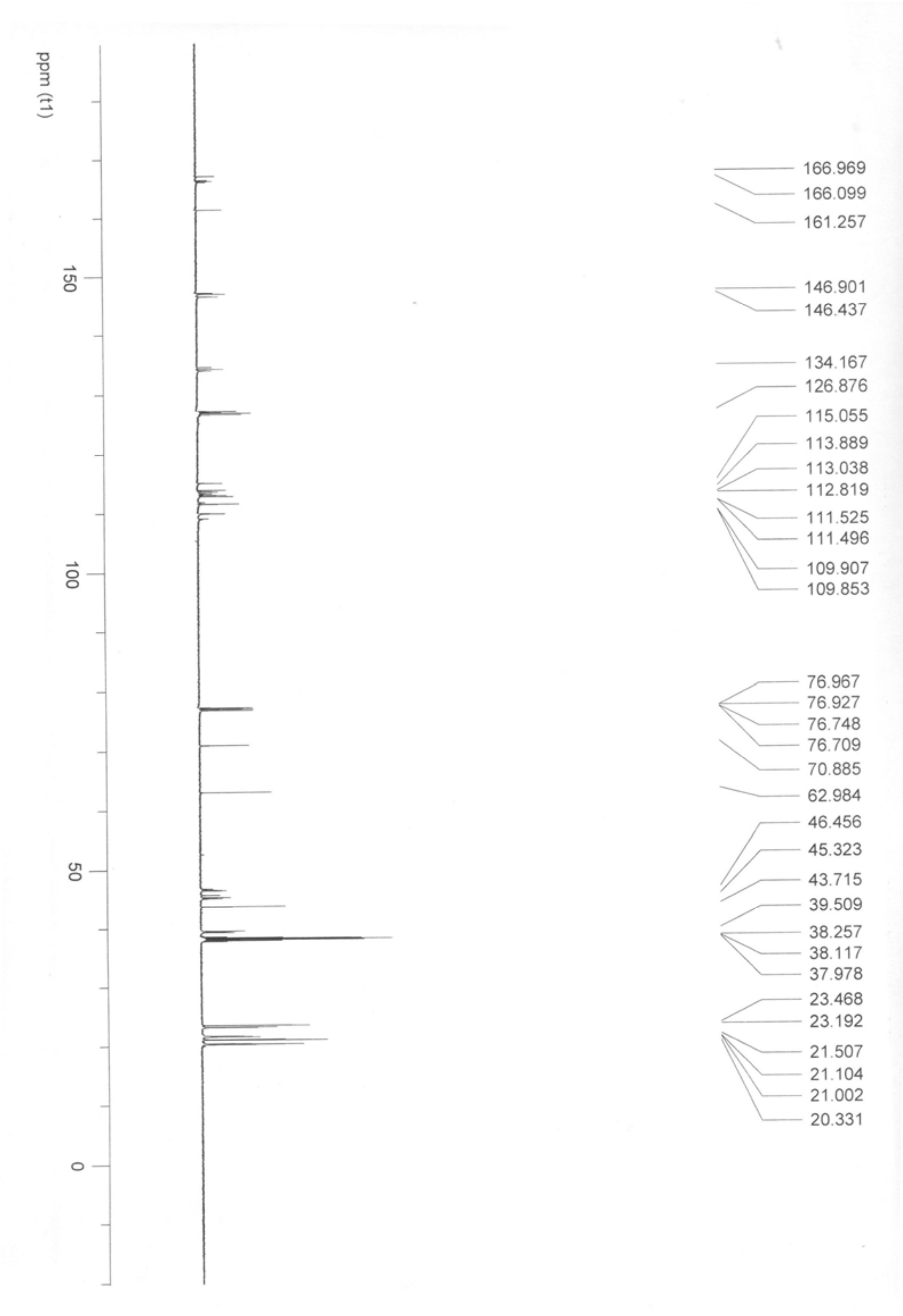
ppm (t1)
150
100
50
0
166.969
166.099
161.257
146.901
146.437
134.167
126.876
115.055
113.889
113.038
112.819
111.525
111.496
109.907
109.853
76.967
76.927
76.748
76.709
70.885
62.984
46.456
45.323
43.715
39.509
38.257
38.117
37.978
23.468
23.192
21.507
21.104
21.002
20.331

Compound 7

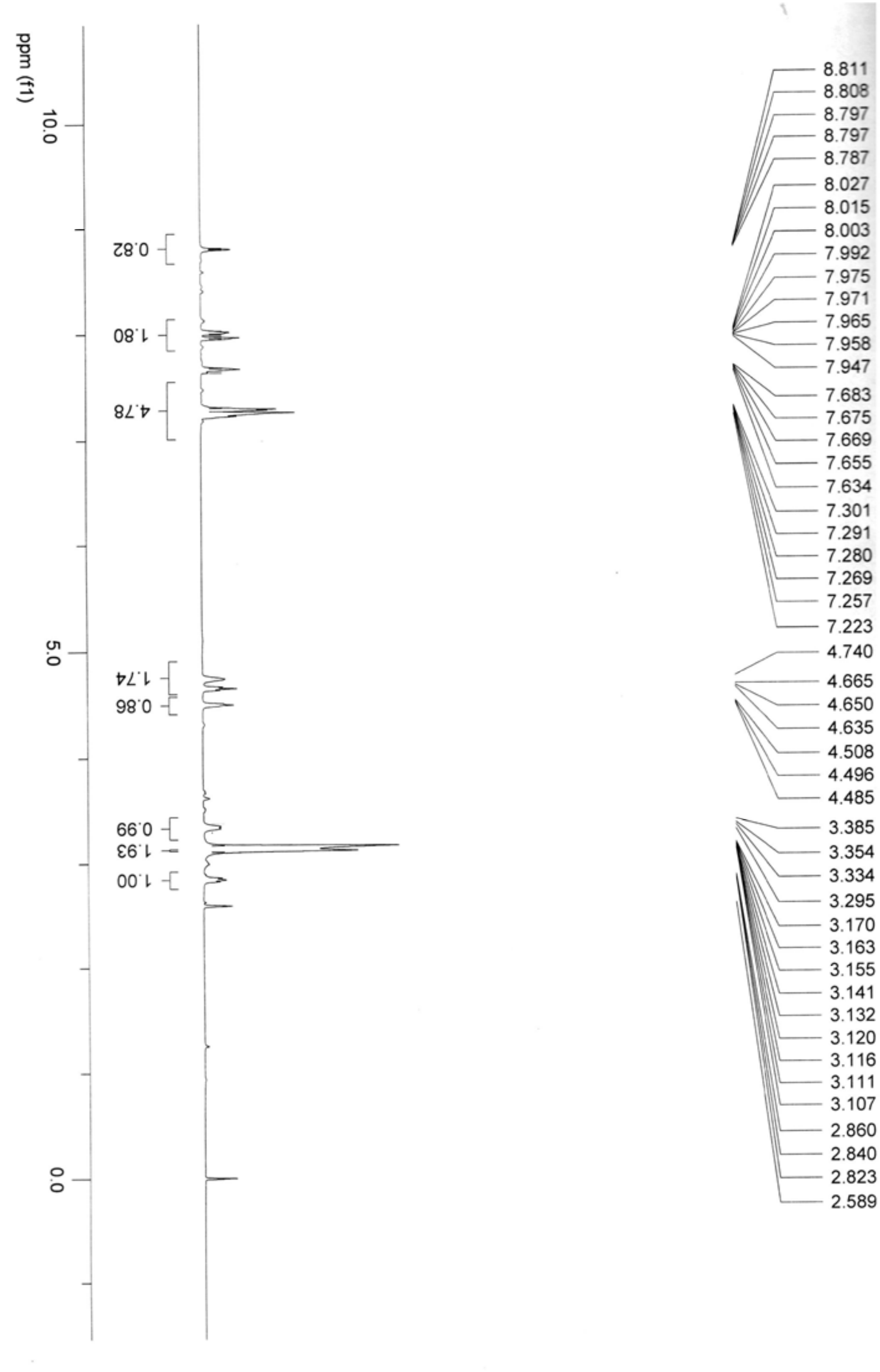
ppm (f1)
10.0
5.0
0.0
0.82
1.80
4.78
1.74
0.86
0.99
1.93
1.00
8.811
8.808
8.797
8.797
8.787
8.027
8.015
8.003
7.992
7.975
7.971
7.965
7.958
7.947
7.683
7.675
7.669
7.655
7.634
7.301
7.291
7.280
7.269
7.257
7.223
4.740
4.665
4.650
4.635
4.508
4.496
4.485
3.385
3.354
3.334
3.295
3.170
3.163
3.155
3.141
3.132
3.120
3.116
3.111
3.107
2.860
2.840
2.823
2.589

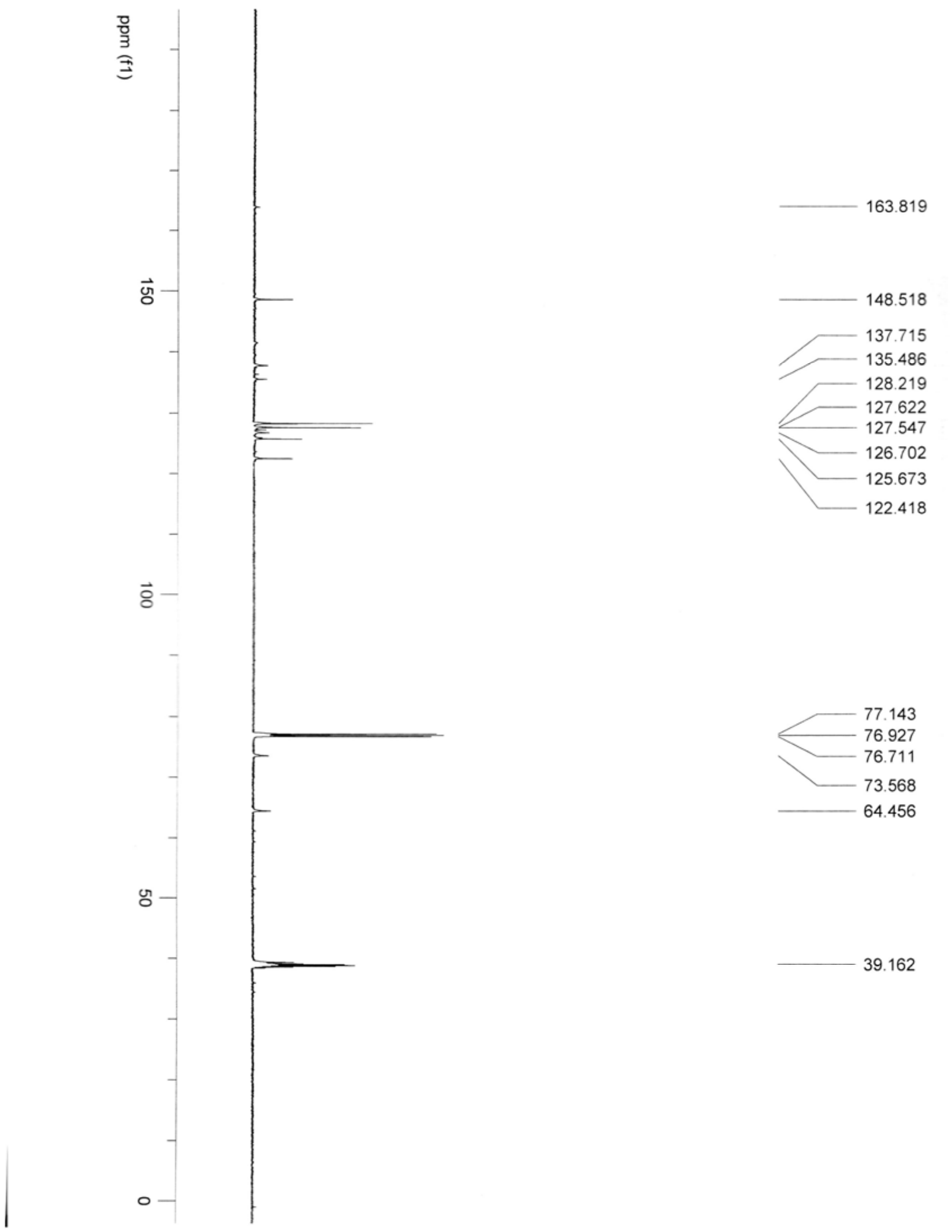
ppm (f1)
150
100
50
0
163.819
148.518
137.715
135.486
128.219
127.622
127.547
126.702
125.673
122.418
77.143
76.927
76.711
73.568
64.456
39.162

Compound 8

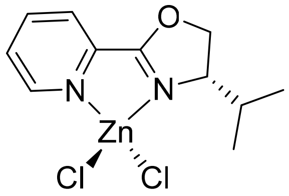
O
N
N
Zn
Cl
Cl

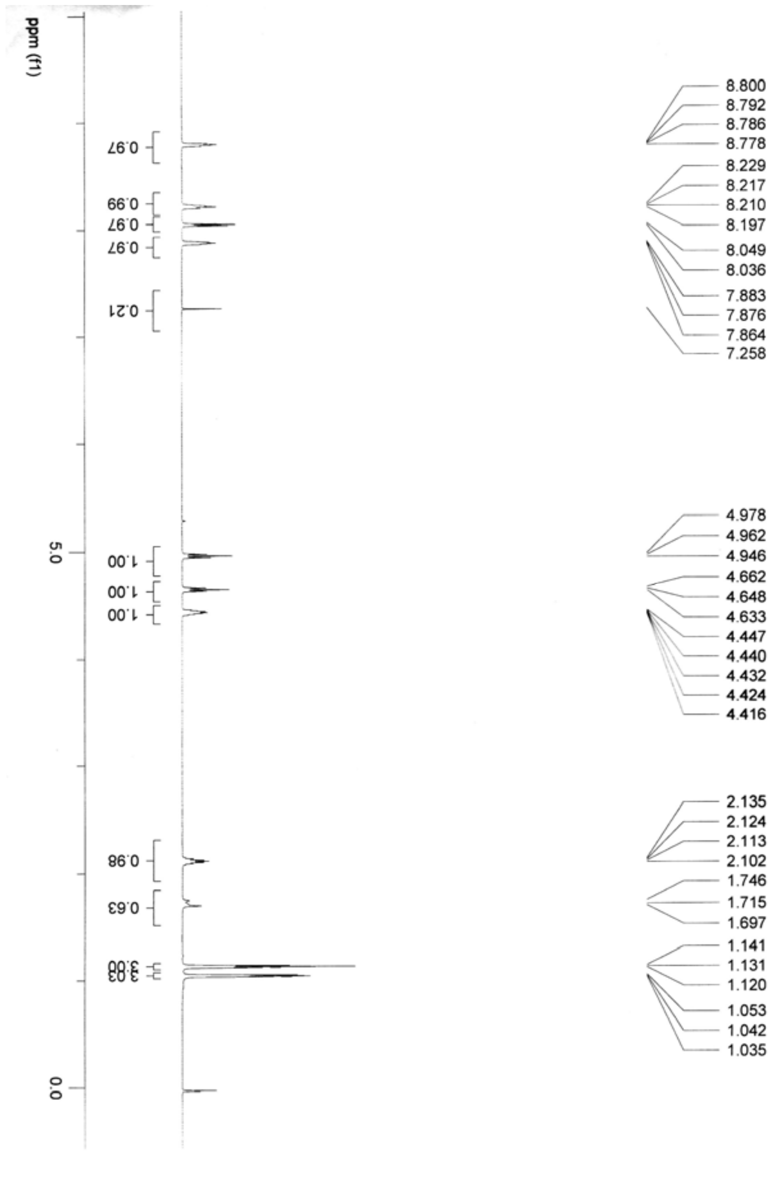
ppm (f1)
5.0
0.0
0.97
0.99
0.97
0.97
0.21
1.00
1.00
1.00
0.98
0.63
3.00
3.03
8.800
8.792
8.786
8.778
8.229
8.217
8.210
8.197
8.049
8.036
7.883
7.876
7.864
7.258
4.978
4.962
4.946
4.662
4.648
4.633
4.447
4.440
4.432
4.424
4.416
2.135
2.124
2.113
2.102
1.746
1.715
1.697
1.141
1.131
1.120
1.053
1.042
1.035

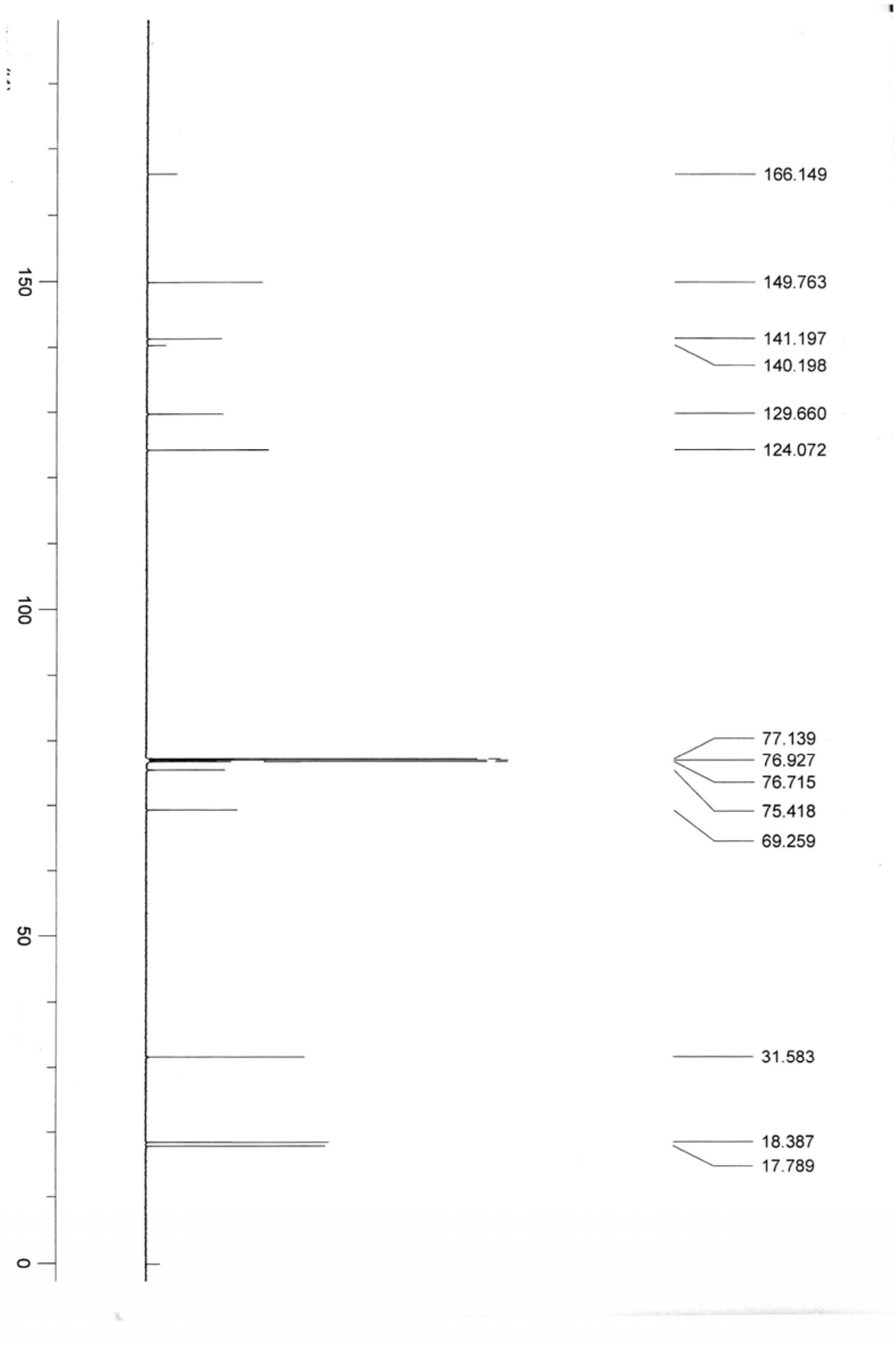
166.149
149.763
141.197
140.198
129.660
124.072
77.139
76.927
76.715
75.418
69.259
31.583
18.387
17.789
150
100
50
0

Compound 9

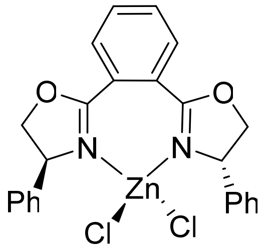
O
O
N
N
Zn
Ph
Ph
Cl
Cl

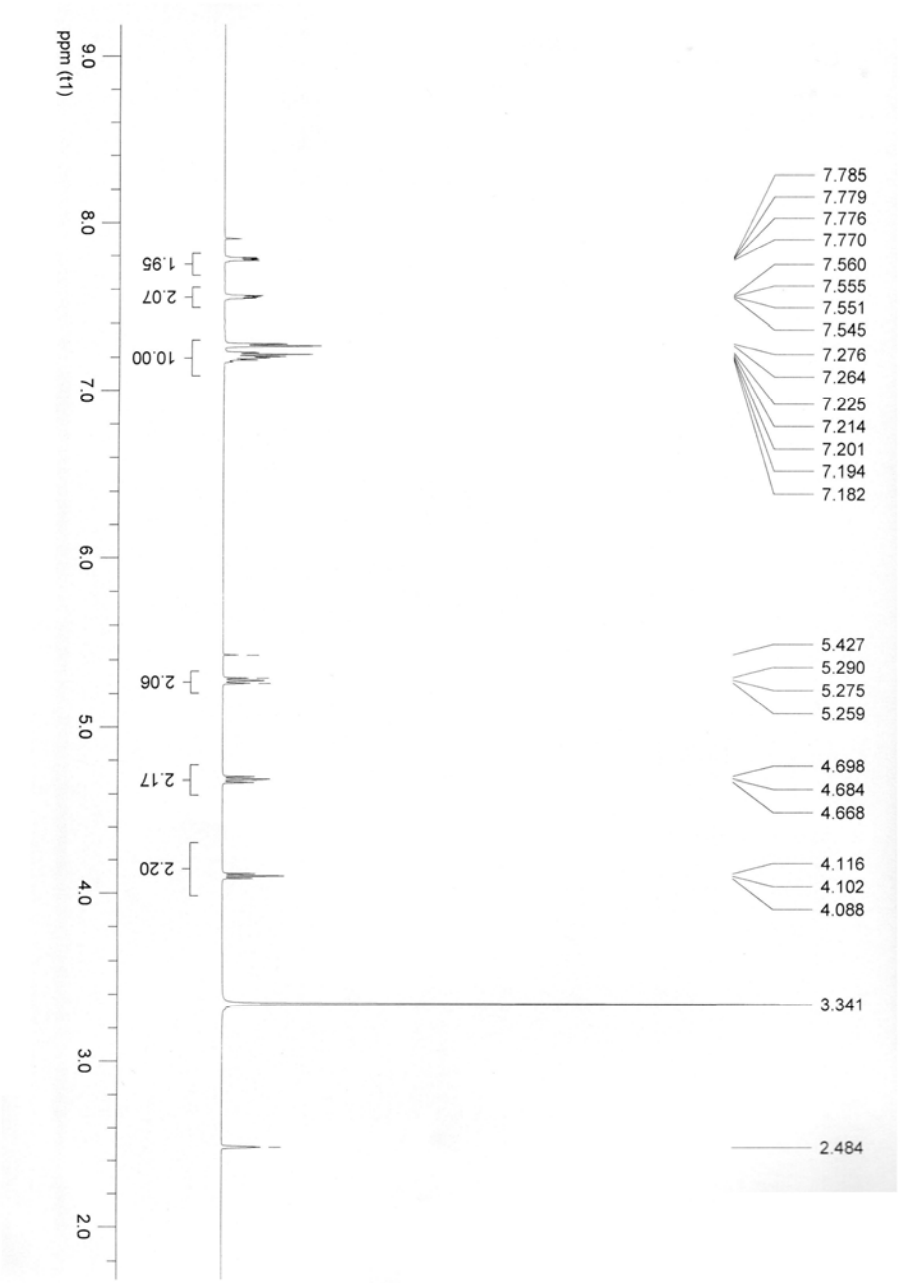
ppm (t1)
9.0
8.0
7.0
6.0
5.0
4.0
3.0
2.0
1.95
2.07
10.00
2.06
2.17
2.20
7.785
7.779
7.776
7.770
7.560
7.555
7.551
7.545
7.276
7.264
7.225
7.214
7.201
7.194
7.182
5.427
5.290
5.275
5.259
4.698
4.684
4.668
4.116
4.102
4.088
3.341
2.484

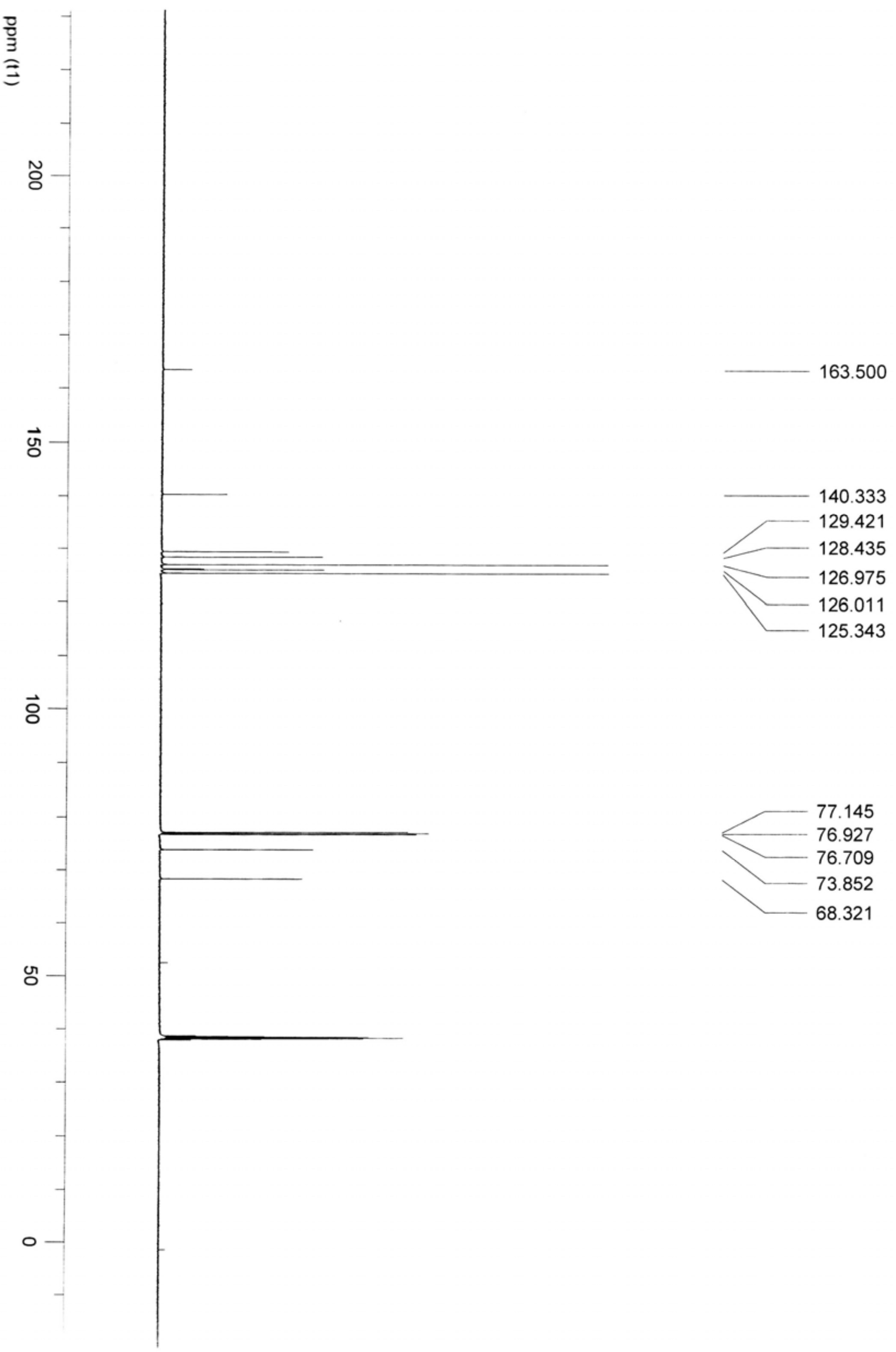
ppm (t1)
200
150
100
50
0
163.500
140.333
129.421
128.435
126.975
126.011
125.343
77.145
76.927
76.709
73.852
68.321

Compound 10

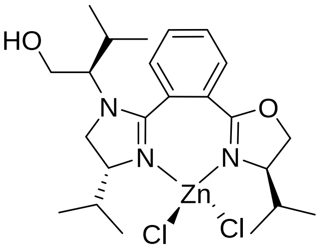
HO
N
O
N
N
Zn
Cl
Cl

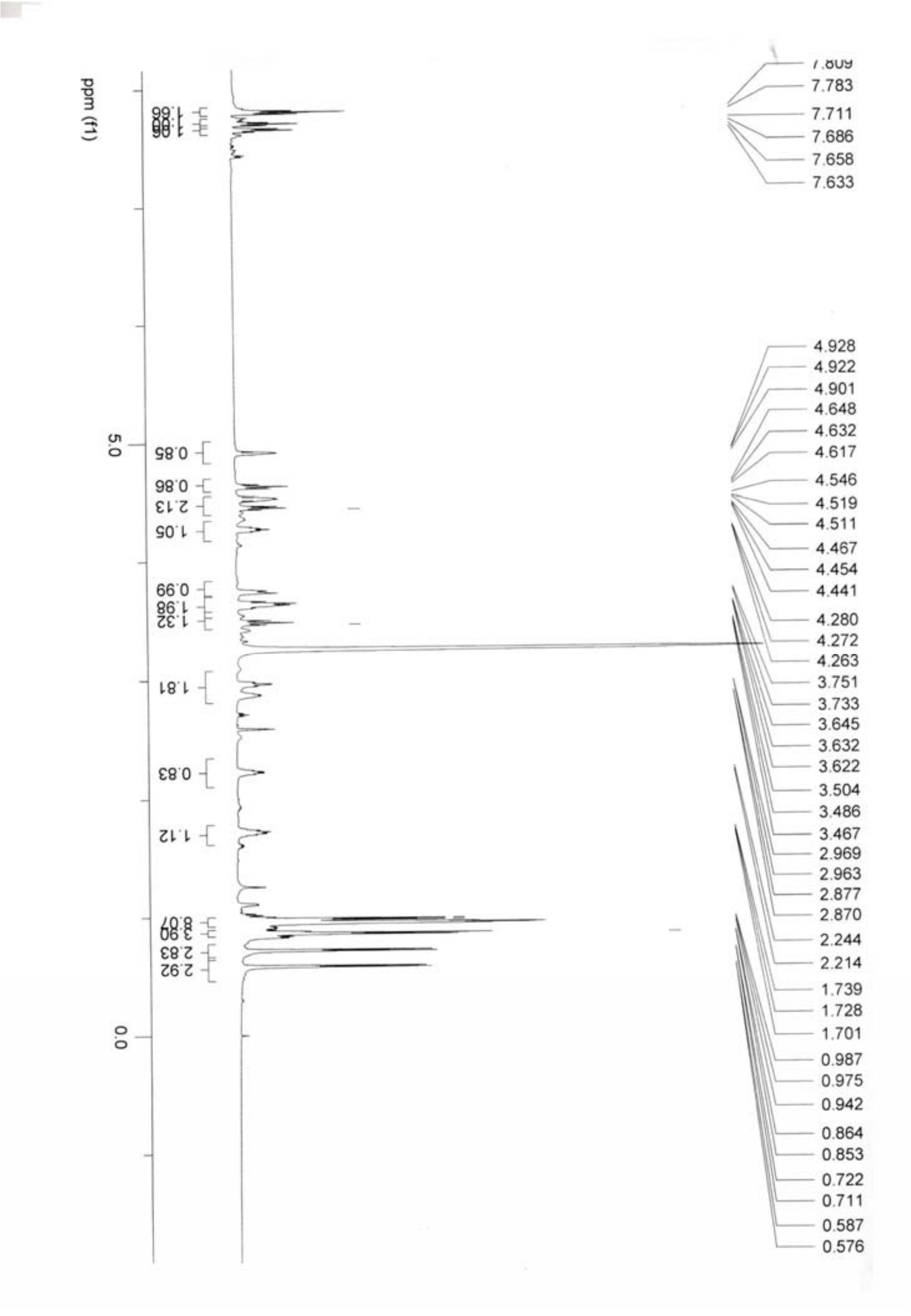
ppm (f1)
5.0
0.0
7.783
7.711
7.686
7.658
7.633
4.928
4.922
4.901
4.648
4.632
4.617
4.546
4.519
4.511
4.467
4.454
4.441
4.280
4.272
4.263
3.751
3.733
3.645
3.632
3.622
3.504
3.486
3.467
2.969
2.963
2.877
2.870
2.244
2.214
1.739
1.728
1.701
0.987
0.975
0.942
0.864
0.853
0.722
0.711
0.587
0.576

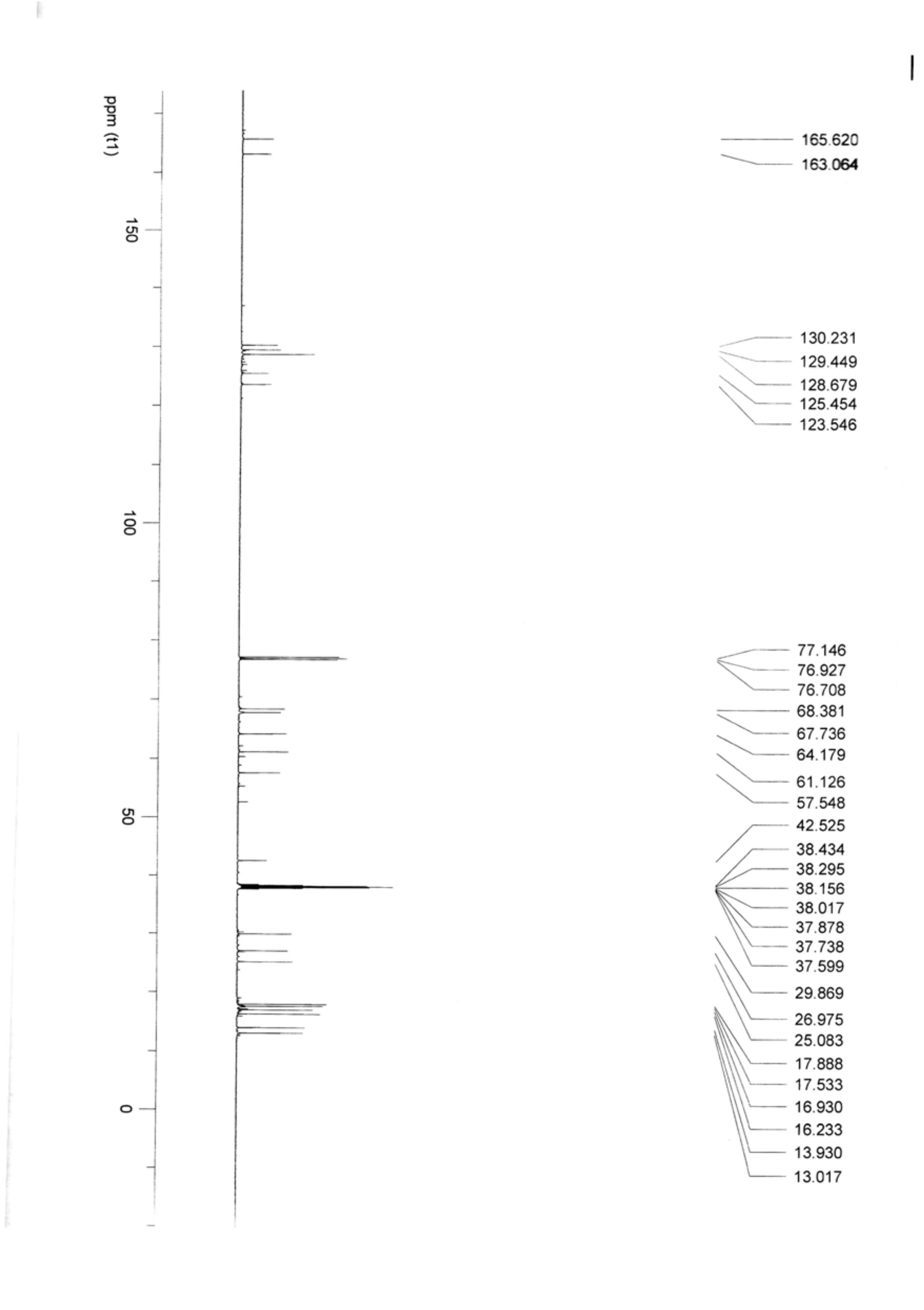
ppm (t1)
150
100
50
0
165.620
163.064
130.231
129.449
128.679
125.454
123.546
77.146
76.927
76.708
68.381
67.736
64.179
61.126
57.548
42.525
38.434
38.295
38.156
38.017
37.878
37.738
37.599
29.869
26.975
25.083
17.888
17.533
16.930
16.233
13.930
13.017

Compound 11

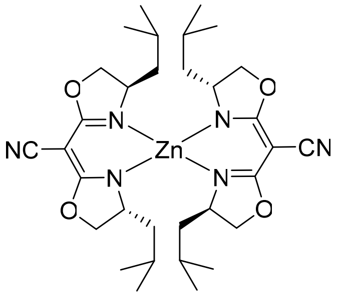
NC
CN
Zn
N
O

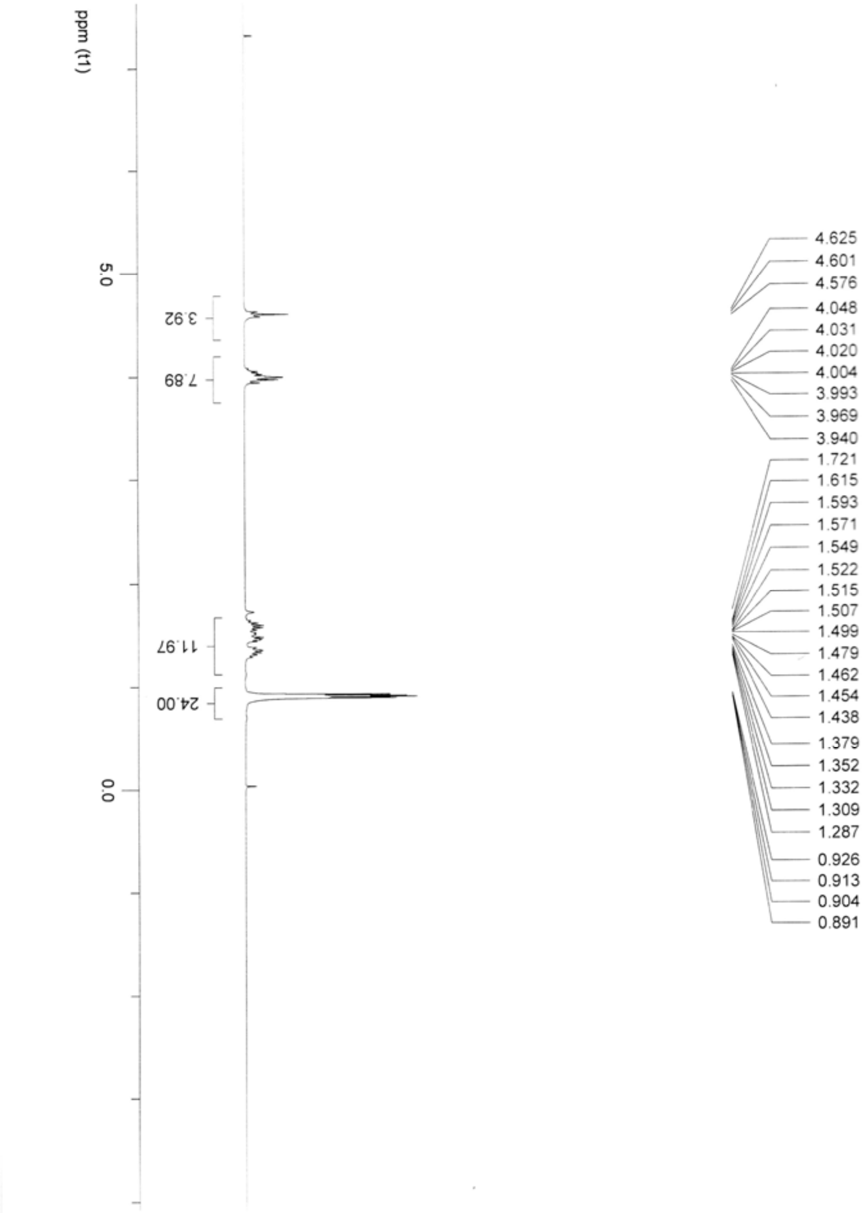
ppm (t1)
5.0
0.0
3.92
7.89
11.97
24.00
4.625
4.601
4.576
4.048
4.031
4.020
4.004
3.993
3.969
3.940
1.721
1.615
1.593
1.571
1.549
1.522
1.515
1.507
1.499
1.479
1.462
1.454
1.438
1.379
1.352
1.332
1.309
1.287
0.926
0.913
0.904
0.891

Compound 12

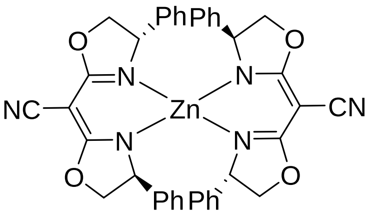
Ph
Ph
O
N
Zn
NC
CN

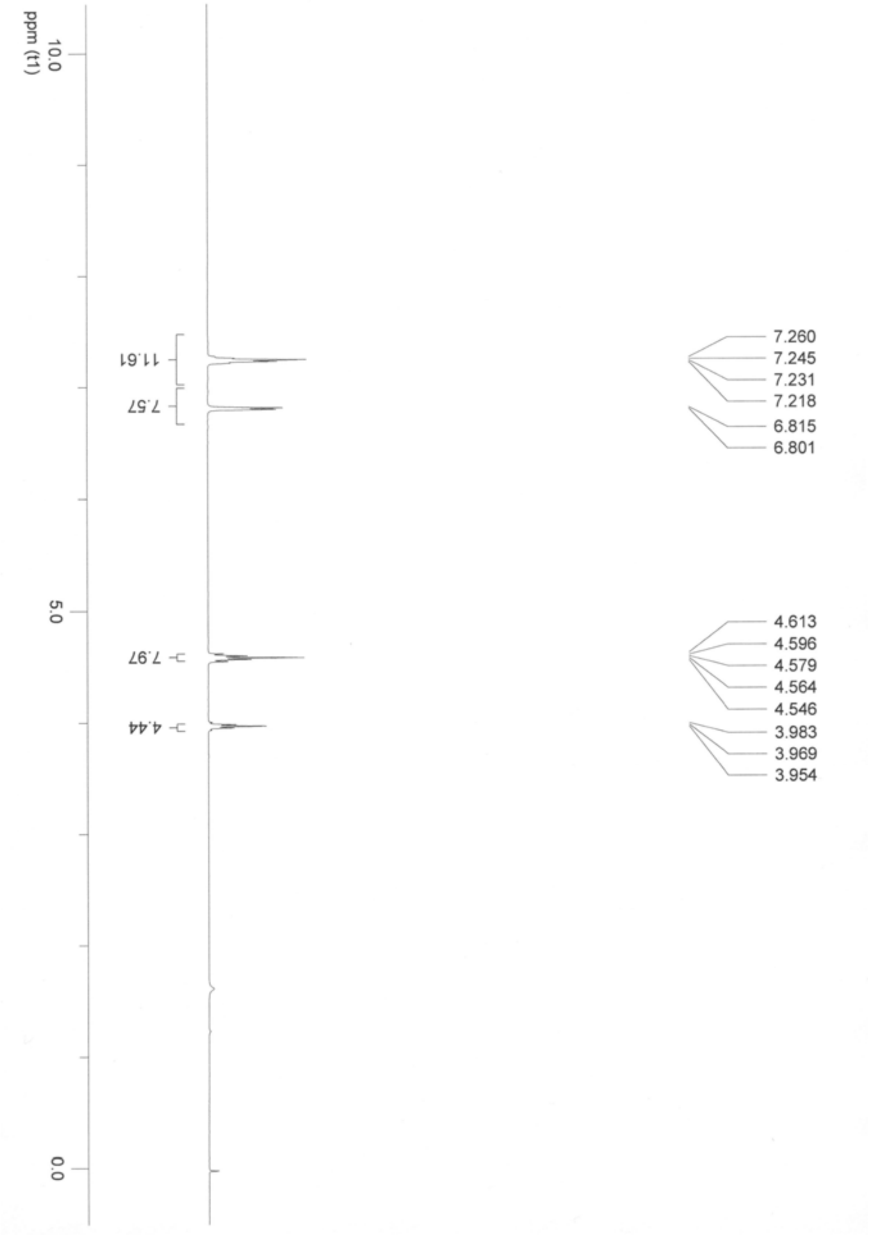
ppm (t1)
10.0
5.0
0.0
11.61
7.57
7.97
4.44
7.260
7.245
7.231
7.218
6.815
6.801
4.613
4.596
4.579
4.564
4.546
3.983
3.969
3.954

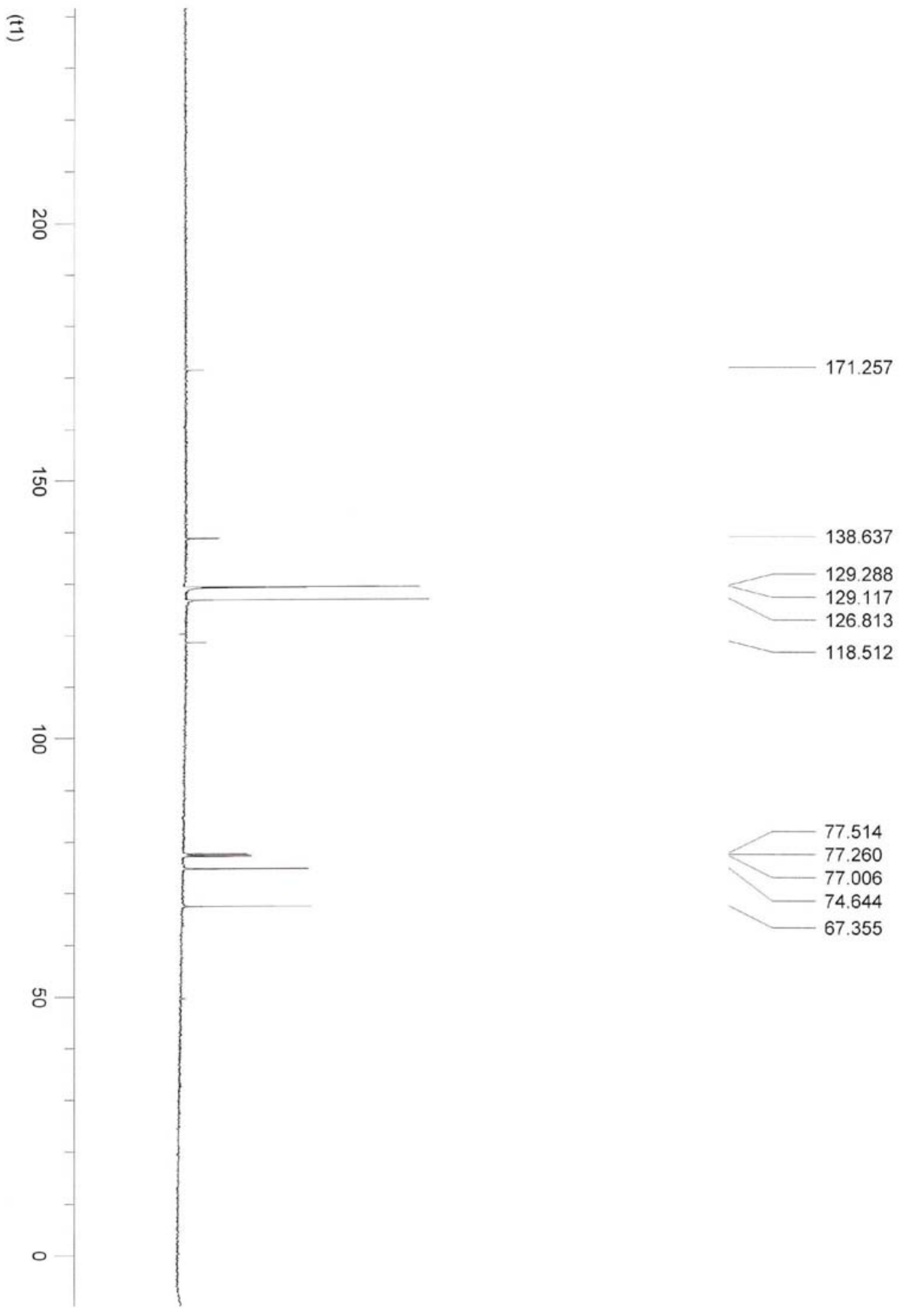
(t1)
200
150
100
50
0
171.257
138.637
129.288
129.117
126.813
118.512
77.514
77.260
77.006
74.644
67.355

Compound 13

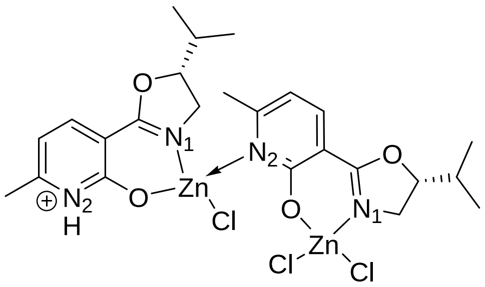

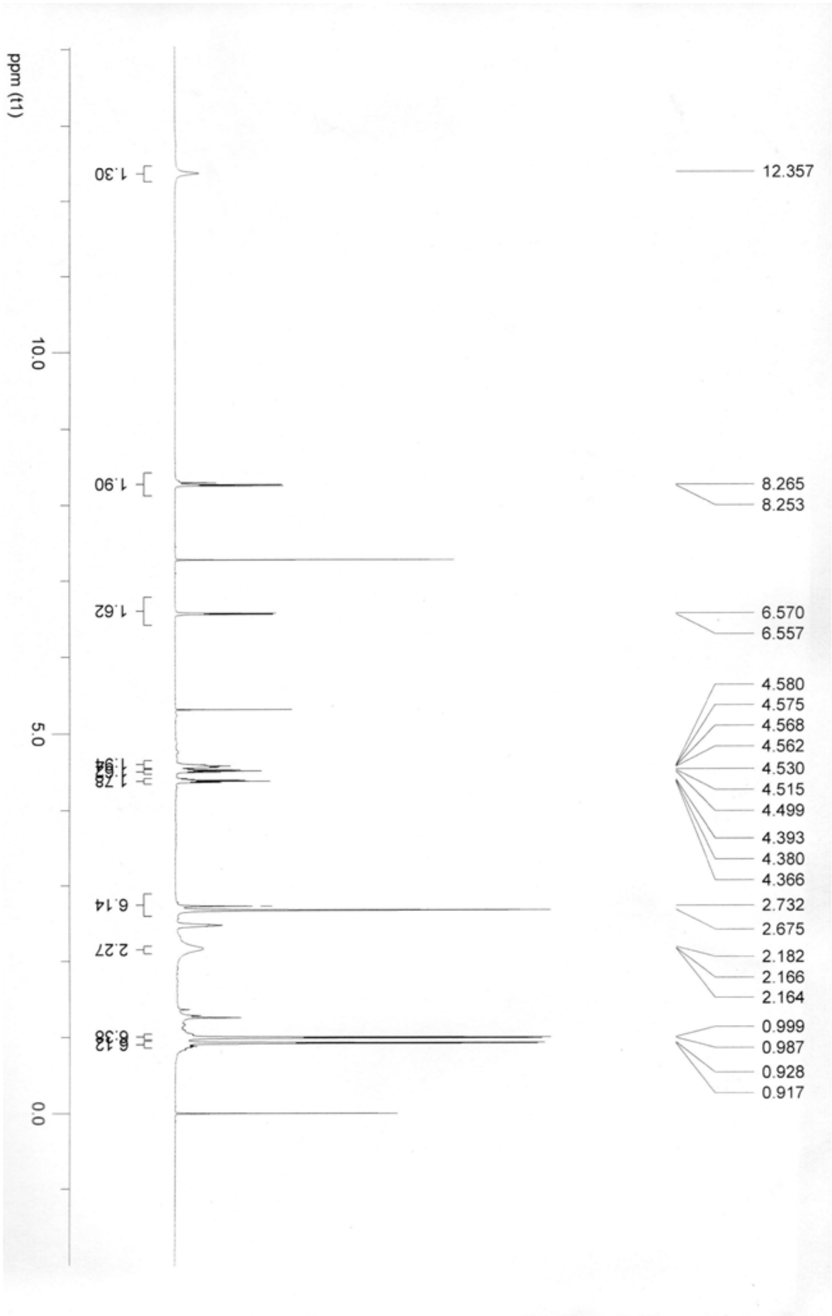
ppm (t1)
10.0
5.0
0.0
12.357
8.265
8.253
6.570
6.557
4.580
4.575
4.568
4.562
4.530
4.515
4.499
4.393
4.380
4.366
2.732
2.675
2.182
2.166
2.164
0.999
0.987
0.928
0.917

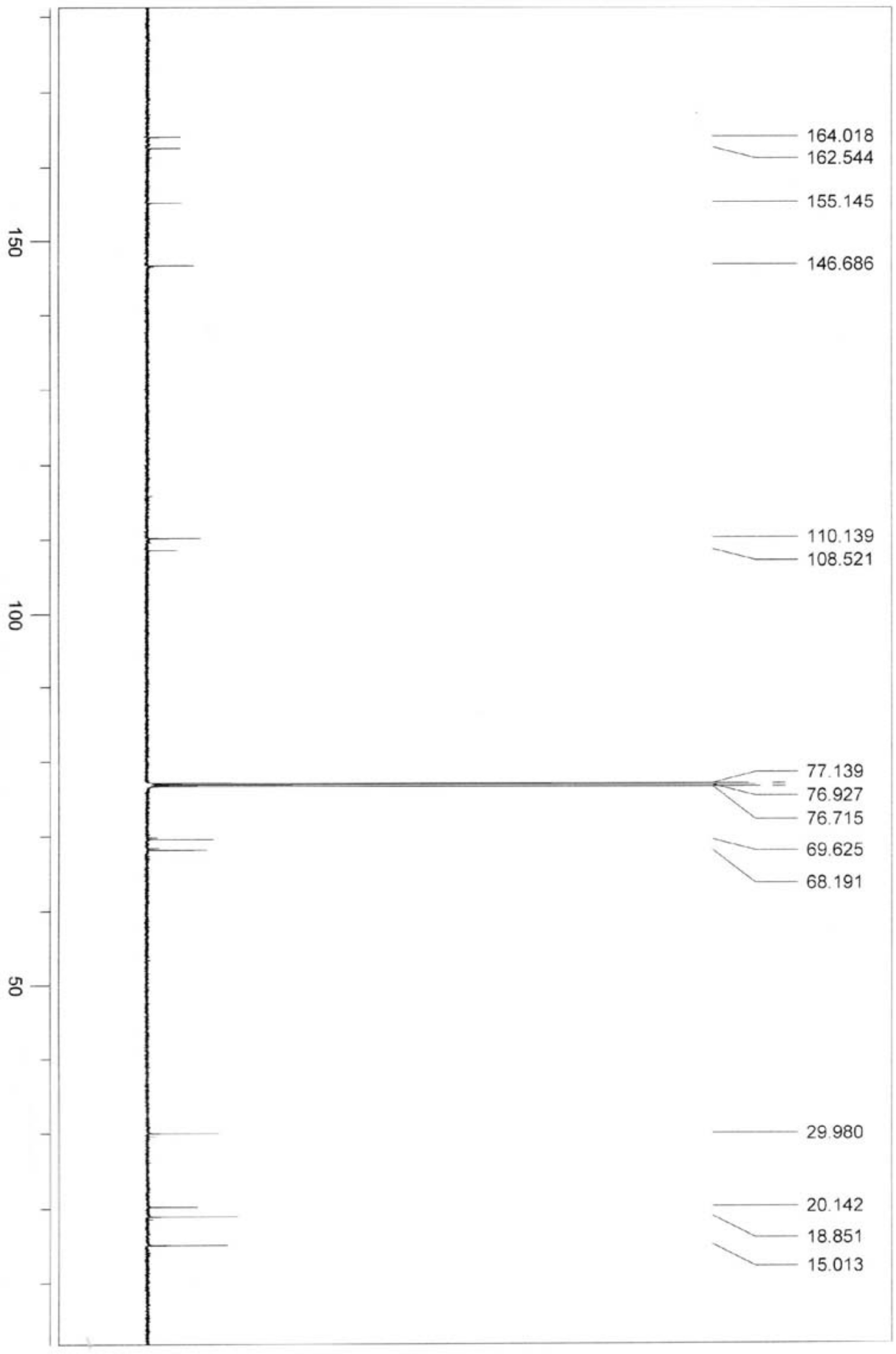
164.018
162.544
155.145
146.686
110.139
108.521
77.139
76.927
76.715
69.625
68.191
29.980
20.142
18.851
15.013
150
100
50

Compound 14

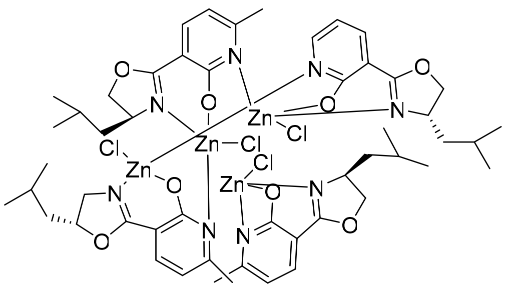

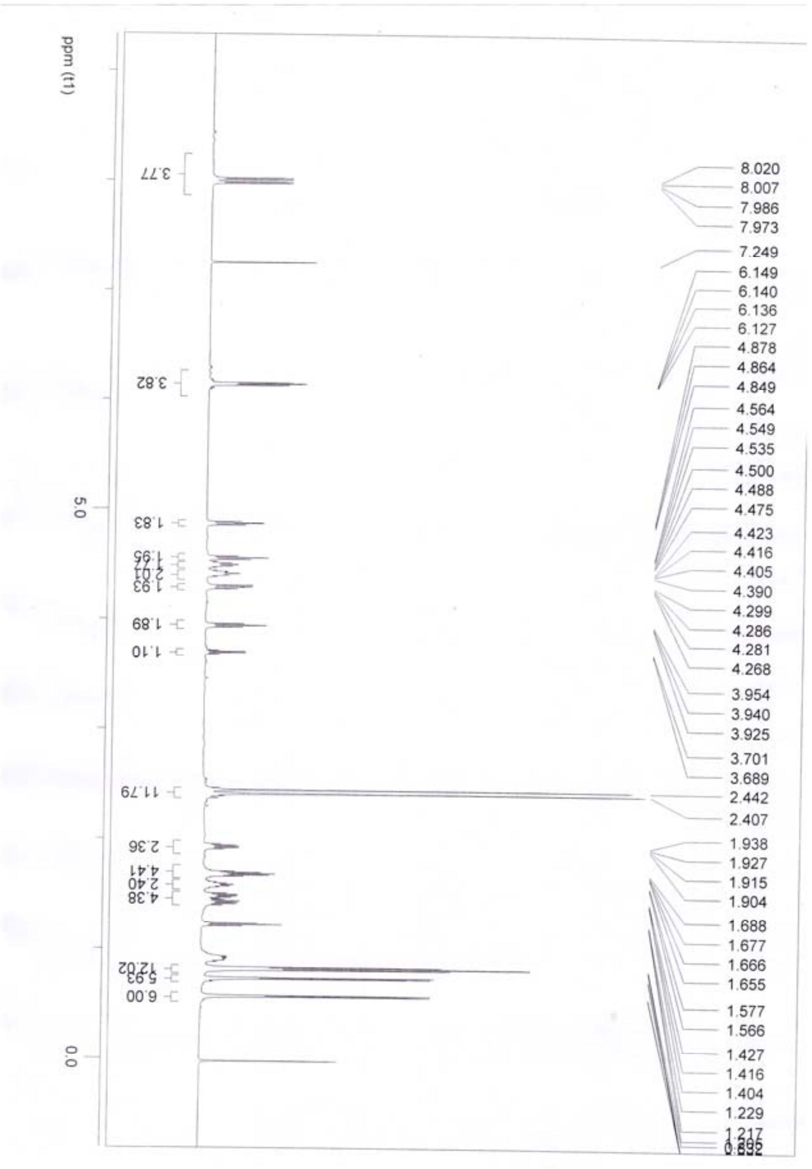

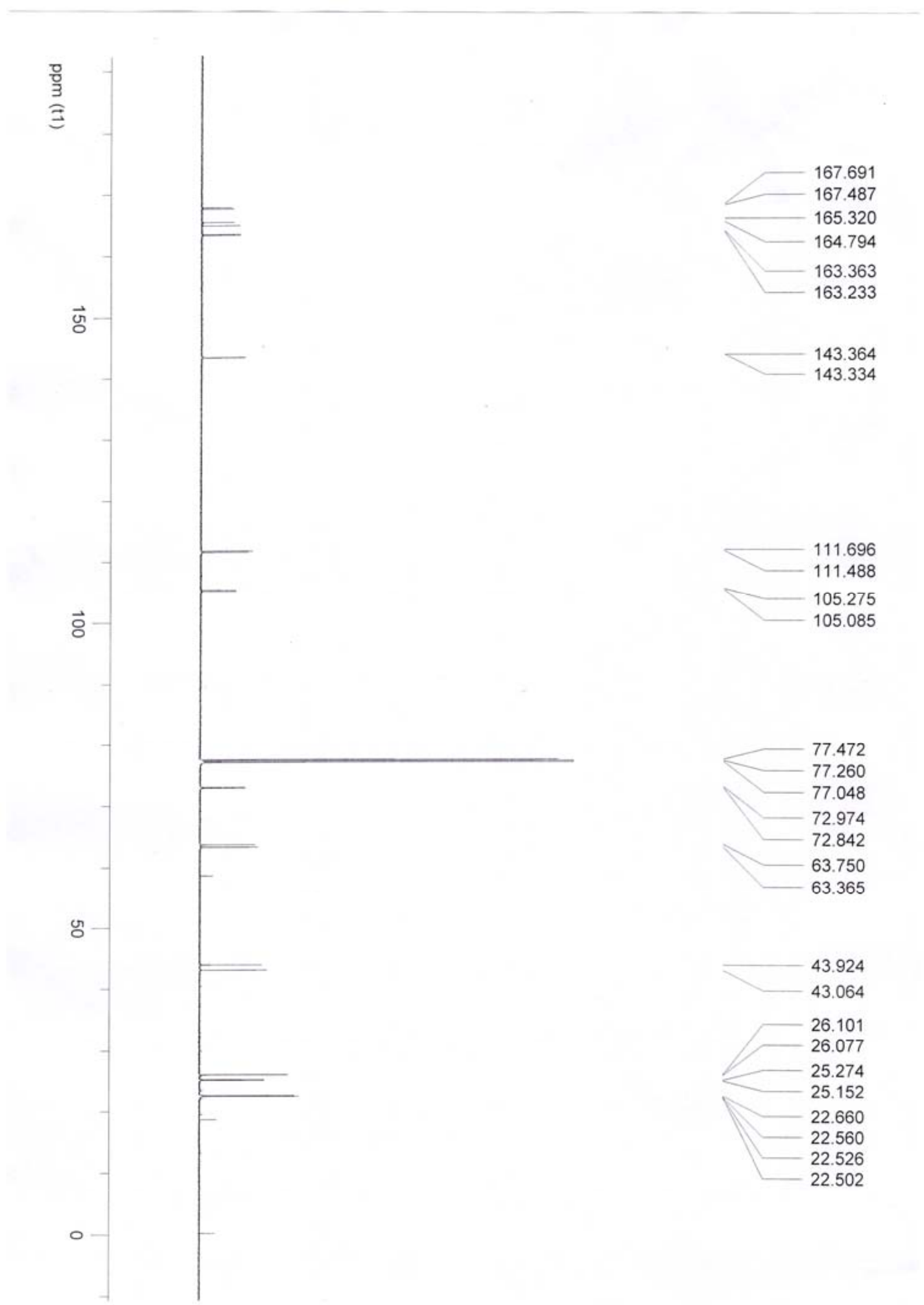
ppm (t1)
150
100
50
0
167.691
167.487
165.320
164.794
163.363
163.233
143.364
143.334
111.696
111.488
105.275
105.085
77.472
77.260
77.048
72.974
72.842
63.750
63.365
43.924
43.064
26.101
26.077
25.274
25.152
22.660
22.560
22.526
22.502

Compound 15

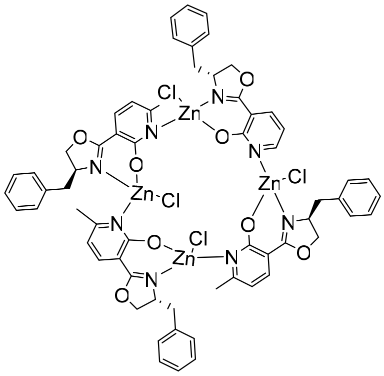
Cl
Zn
N
O
Zn
Cl
Zn
Cl
Cl
Zn

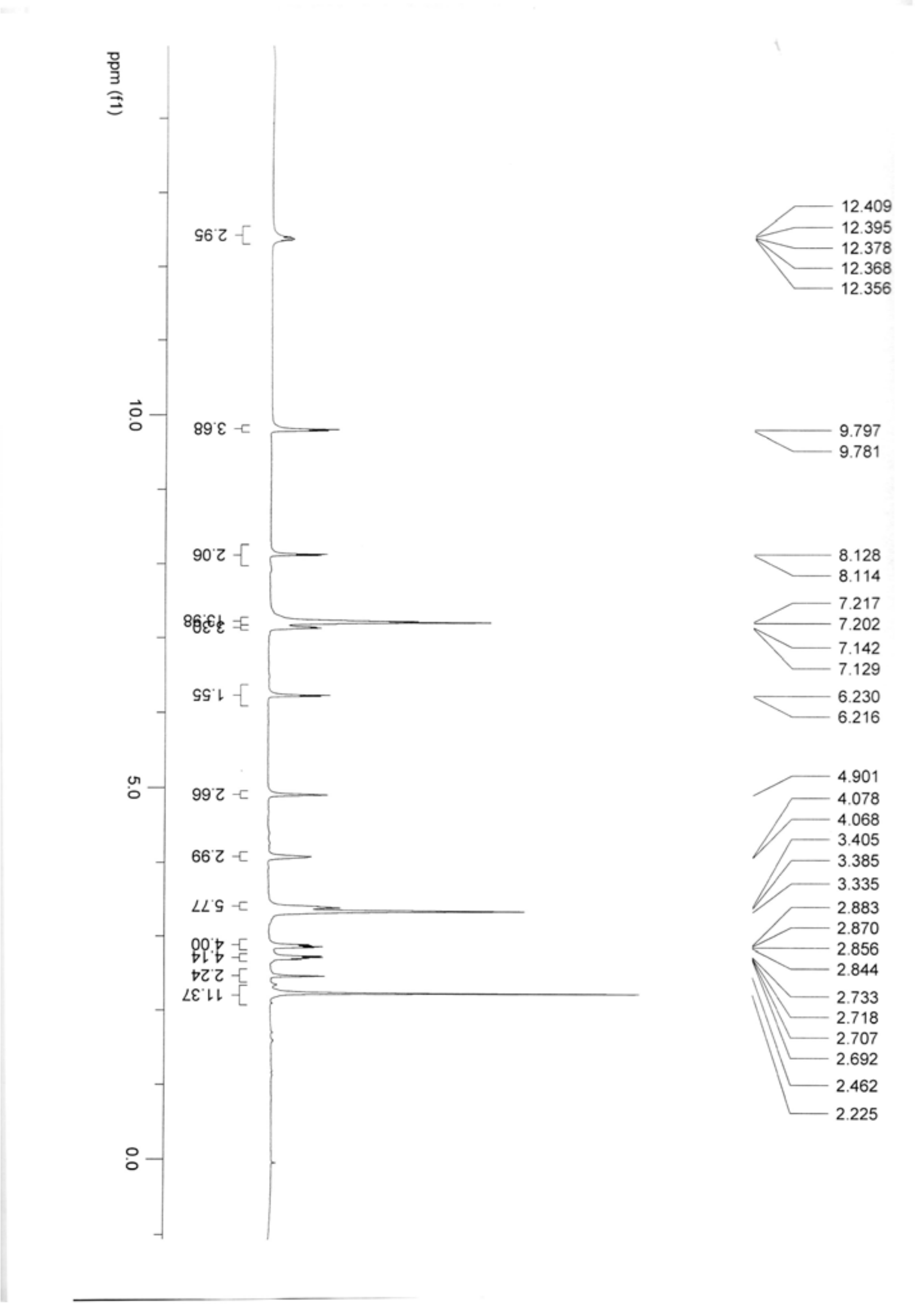
ppm (f1)
10.0
5.0
0.0
2.95
3.68
2.06
13.98
3.30
1.55
2.66
2.99
5.77
4.00
4.14
2.24
11.37
12.409
12.395
12.378
12.368
12.356
9.797
9.781
8.128
8.114
7.217
7.202
7.142
7.129
6.230
6.216
4.901
4.078
4.068
3.405
3.385
3.335
2.883
2.870
2.856
2.844
2.733
2.718
2.707
2.692
2.462
2.225

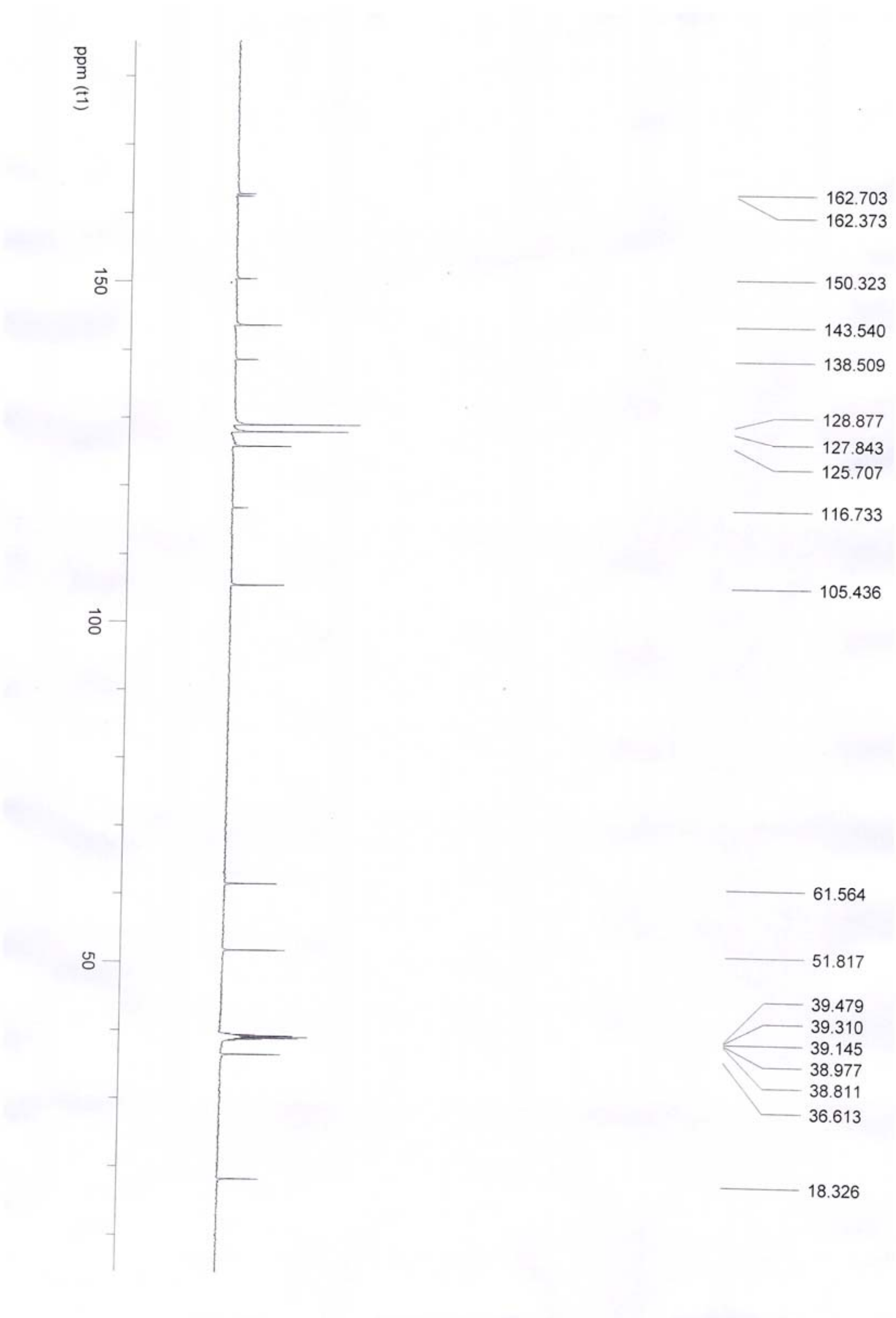
ppm (t1)
150
100
50
162.703
162.373
150.323
143.540
138.509
128.877
127.843
125.707
116.733
105.436
61.564
51.817
39.479
39.310
39.145
38.977
38.811
36.613
18.326
